# iPPBS-Opt: A Sequence-Based Ensemble Classifier for Identifying Protein-Protein Binding Sites by Optimizing Imbalanced Training Datasets

**DOI:** 10.3390/molecules21010095

**Published:** 2016-01-19

**Authors:** Jianhua Jia, Zi Liu, Xuan Xiao, Bingxiang Liu, Kuo-Chen Chou

**Affiliations:** 1 Computer Department, Jing-De-Zhen Ceramic Institute, Jing-De-Zhen 333403, China; liuzi189836@163.com (Z.L.); lbx1966@163.com (B.L.); 2 Gordon Life Science Institute, Boston, MA 02478, USA; kcchou@gordonlifescience.org; 3 Center of Excellence in Genomic Medicine Research (CEGMR), King Abdulaziz University, Jeddah 21589, Saudi Arabia

**Keywords:** protein-protein binding sites, physicochemical property, stationary wavelet transform, PseAAC, Optimize training dataset, KNNC, IHTS, target cross-validation

## Abstract

Knowledge of protein-protein interactions and their binding sites is indispensable for in-depth understanding of the networks in living cells. With the avalanche of protein sequences generated in the postgenomic age, it is critical to develop computational methods for identifying in a timely fashion the protein-protein binding sites (PPBSs) based on the sequence information alone because the information obtained by this way can be used for both biomedical research and drug development. To address such a challenge, we have proposed a new predictor, called **iPPBS-Opt**, in which we have used: (1) the K-Nearest Neighbors Cleaning (KNNC) and Inserting Hypothetical Training Samples (IHTS) treatments to optimize the training dataset; (2) the ensemble voting approach to select the most relevant features; and (3) the stationary wavelet transform to formulate the statistical samples. Cross-validation tests by targeting the experiment-confirmed results have demonstrated that the new predictor is very promising, implying that the aforementioned practices are indeed very effective. Particularly, the approach of using the wavelets to express protein/peptide sequences might be the key in grasping the problem’s essence, fully consistent with the findings that many important biological functions of proteins can be elucidated with their low-frequency internal motions. To maximize the convenience of most experimental scientists, we have provided a step-by-step guide on how to use the predictor’s web server (http://www.jci-bioinfo.cn/iPPBS-Opt) to get the desired results without the need to go through the complicated mathematical equations involved.

## 1. Introduction

Individual proteins rarely function alone. Most proteins whose functions are essential to life are associated with protein-protein interactions [1]. Actually, these kinds of interactions affect the biological processes in a living cell. To really understand protein-protein interactions, however, it is indispensable to acquire the information of protein-protein binding site (PPBS). Despite many studies on the binding site of a protein or DNA with its ligand (small molecule) have been made [2,3,4,5,6,7,8], relatively much less studies have been conducted on PPBS, particularly based on the sequence information alone. It is both time-consuming and expensive to determine PPBS purely based on biochemical experiments. Facing the enormous number of protein sequences generated in the postgenomic era, it is highly desired to develop computational methods to identify PPBSs for uncharacterized proteins so that they can be timely used for both basic research and drug development, such as conducting mutagenesis studies [9] and prioritize drug targets.

Given a protein sequence, how can one identify which of its constituent amino acid residues are located in the binding sites? Actually, considerable efforts were made to address this problem [10,11]. Although the aforementioned works each have their own merits and did play a role in stimulating the development of this area, further work is needed due to the following shortcomings: (1) The datasets used by these authors to train their prediction methods were highly imbalanced or with a strong bias; *i.e.*, the number of non-PPBS samples was significantly larger than that of PPBS samples; (2) None of their prediction methods has a publicly accessible web server, and hence their practical application value is quite limited, particularly for the majority of experimental scientists.

The present study is initiated in an attempt to develop a new PPBS predictor by addressing the aforementioned shortcomings. According to the Chou’s 5-step rule [12] and the demonstrations in a series of recent publications [13,14,15,16,17,18,19,20], to establish a really useful sequence-based statistical predictor for a biological system, we should make the following five aspects crystal clear: (1) how to construct or select a valid benchmark dataset to train and test the predictor; (2) how to formulate the biological sequence samples with an effective mathematical expression that can truly reflect their intrinsic correlation with the target to be predicted; (3) how to introduce or develop a powerful algorithm (or engine) to operate the prediction; (4) how to properly perform cross-validation tests to objectively evaluate its anticipated accuracy; (5) how to establish a user-friendly web-server that is accessible to the public. Below, we are to address the five procedures one-by-one.

## 2. Materials and Methods

### 2.1. Benchmark Dataset

Two benchmark datasets were used for the current study. One is the “surface-residue” dataset and the other is “all-residue” dataset, as described below. The protein-protein interfaces are usually formed by those residues, which are exposed to the solvent after the two counterparts are separated from each other [21]. Given a protein sample with *L* residues as expressed by:

(1) $$P=R_{1}R_{2}R_{3}R_{4}R_{5}R_{6}R_{7}\cdots R_{L}$$

where R_1_ represents the 1st amino acid residue of the protein P, R_2_ the 2nd residue, and so forth. The residue $R_{i\;}\left(i=1,2,\cdots ,L\right)\;$is deemed as a surface residue if it satisfies the following condition:

(2) $$\phi \left(R_{i}\right)=\frac{\text{ASA}(R_{i}|P)}{\text{ASA}\left(R_{i}\right)}>25\%$$

where ASA(R*_i_*|P) is the accessible surface area of R*_i_* when it is a part of protein P, ASA(R*_i_*) is the accessible surface area of the free R*_i_* that is actually its maximal accessible surface area as given in Table 1 [22], and $\phi \left(R_{i}\right)$ is the ratio of the two.

**Table 1:** Maximum accessible surface area (ASA) of different amino acids ^a^.

AA	A	B	C	D	E	F	G	H	I	K	L	M
MaxASA	106	160	135	163	194	197	84	184	169	205	164	188
AA	N	P	Q	R	S	T	V	W	X	Y	Z	
MaxASA	157	136	198	248	130	142	142	227	180	222	196	

^a^ Amino acids are represented by their one-letter codes. Here, B stands for D or N; Z for E or Q, and X for an undetermined amino acid.

Furthermore, the surface residue $R_{i}$ is deemed as interfacial residue [23] if:

(3) $$\text{ASA}\left(R_{i}|P\right)-\text{ASA}\left(R_{i}|PP\right)>1{Å}^{2}$$

where ASA(R*_i_*|**PP**) is the accessible surface area of $R_{i}$ when it is a part of protein-protein complex.

For a given protein, we can use DSSP program [24] to find out all its surface residues based on Equation (2), and use PSAIA program [25] to find all its interfacial residues based on Equation (3).

If only considering the surface residues as done in [26] for the 99 polypeptide chains extracted by Deng *et al.* [10] from the 54 heterocomplexes in the Protein Data Bank, we have obtained the results that can be formulated as follows:

(4) $$S_{\text{surf}}=S_{\text{surf}}^{+}\cup^{\text{​}}S_{\text{surf}}^{-}$$

where $S_{\text{surf}}\;$ is called the “surface-residue dataset” that contains a total of 13,771 surfaces residues, of which 2828 are interfacial residues belonging to the positive subset $S_{\text{surf}}^{+}$ while 10,943 are non-interfacial residues belonging the negative subset $S_{\text{surf}}^{-}$, and $\cup^{\text{​}}$ is the symbol of union in the set theory.

If considering all the residues as done in [11], however, the corresponding benchmark dataset can be expressed by:

(5) $$S_{\text{all}}=S_{\text{all}}^{+}\cup^{\text{​}}\;S_{\text{all}}^{-}$$

where $S_{\text{all}}\;$is called the “all-residue dataset” that contains a total of 27,442 residues, of which 2828 are interfacial residues belonging to the positive subset $S_{\text{all}}^{+}$ while 24,614 are non-interfacial residues belonging the negative subset $S_{\text{all}}^{-}$.

For readers’ convenience, given in S1 Dataset (List of the 99 proteins and their residues’ attributions associated with the protein-protein binding sites is in Supplementary Materials) is a combination of the two benchmark datasets, where those labeled in column 3 are all the residues determined by experiments, those in column 4 are of surface and non-surface residues, and those in column 5 are of interface and non-interface residues.

As pointed out in a comprehensive review [27] there is no need to separate a benchmark dataset into a training dataset and a testing dataset for examining the quality of a prediction method if it is tested by the jackknife test or subsampling (K-fold) cross-validation test because the outcome obtained via this kind of approach is actually from a combination of many different independent dataset tests.

### 2.2. Flexible Sliding Window Approach

**Figure 1:**
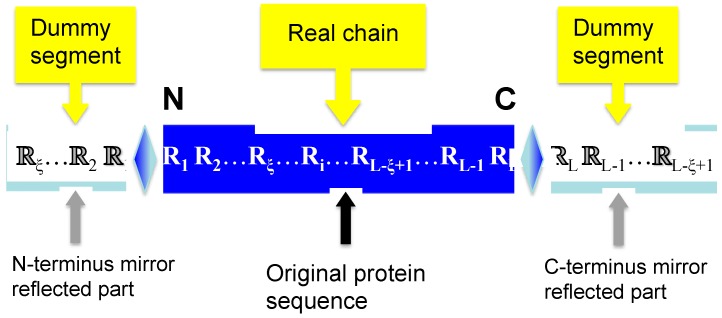
A schematic drawing to show how to use the extended chain of Equation (7) to define the working segments of Equation (6) for those sites when their sequence positions in the protein are less than ξ or greater L−ξ , where the left dummy segment stands for the mirror image of $R_{1}R_{2}\cdots R_{\xi }$ at N-terminus and the right dummy segment for that of $R_{L-\xi +1}\cdots R_{L-1}R_{L}$ at the C-terminus.

Given a protein chain as expressed in Equation (1), the sliding window approach [28] and flexible sliding window approach [29] are often used to investigate its various posttranslational modification (PTM) sites [16,30,31,32,33,34] and HIV (human immunodeficiency virus) protease cleavage sites [35]. Here, we also use it to study protein-protein binding sites. In the sliding window approach, a scaled window is denoted by [–ξ, +ξ] [28], and its width is 2ξ+1, where ξ is an integer. When sliding it along a protein chain **P**, one can see through the window a series of consecutive peptide segments as formulated by:

(6) $$P_{\xi }\left({\mathbb{R}}_{0}\right)=R_{-\xi }R_{-\left(\xi -1\right)}\cdots R_{-2}R_{-1}{\mathbb{R}}_{0}R_{+1}R_{+2}\cdots R_{+\left(\xi -1\right)}R_{+\xi }$$

where $R_{-\xi }$ represents the ξ-th upstream amino acid residue from the center, $R_{+\xi }$ the ξ-th downstream amino acid residue, and so forth. The amino acid residue ${\mathbb{R}}_{0}$ at the center is the targeted residue. When its sequence position in **P** (*cf.* Equation (1) is less than ξ or greater L−ξ, the corresponding $P_{\xi }\left({\mathbb{R}}_{0}\right)\;$ is defined, rather than by **P** of Equation (1), but by the following dummy protein chain:

(7) $$P\left(\text{dummy}\right)={\mathbb{R}}_{\xi }\cdots {\mathbb{R}}_{2}{\mathbb{R}}_{1}⇕R_{1}R_{2}\cdots R_{\xi }\cdots R_{i}\cdots R_{L-\xi +1}\cdots R_{L-1}R_{L}\;⇕{\mathbb{R}}_{L}{\mathbb{R}}_{L-1}\cdots {\mathbb{R}}_{L-\xi +1}$$

where the symbol ⇕ stands for a mirror, the dummy segment ${\mathbb{R}}_{\xi }\cdots {\mathbb{R}}_{2}{\mathbb{R}}_{1}$ stands for the image of $R_{1}R_{2}\cdots R_{\xi }$ reflected by the mirror, and the dummy segment ${\mathbb{R}}_{L}{\mathbb{R}}_{L-1}\cdots {\mathbb{R}}_{L-\xi +1}$ for the mirror image of $R_{L-\xi +1}\cdots R_{L-1}R_{L}$ (Figure 1). Accordingly, **P**(dummy) of Equation (7) is also called the mirror-extended chain of protein **P**.

Thus, for each of the *L* amino acid residues in protein **P**, we have a working protein segment as defined by Equation (6). In the current study, the (2ξ+1)-tuple peptide $P_{\xi }\left({\mathbb{R}}_{0}\right)$ can be further classified into the following categories:

(8) $$P_{\xi }\left({\mathbb{R}}_{0}\right)\{\begin{array}{c} P_{\xi }^{+}\left({\mathbb{R}}_{0}\right),\;\mathrm{if}\;\mathrm{its}\;\mathrm{center}\;\mathrm{is}\;a\;\mathrm{PPBS}\\ P_{\xi }^{-}\left({\mathbb{R}}_{0}\right),\;\;\;\;\;\;\;\;\;\;\;\;\;\;\;\;\;\;\;\;\mathrm{otherwise}\; \end{array}$$

where ∈ represents “a member of” in the set theory.

### 2.3. Using Pseudo Amino Acid Composition to Represent Peptide Chains

One of the most challenging problems in computational biology today is how to effectively formulate the sequence of a biological sample (such as protein/peptide, and DNA/RNA) with a discrete model or a vector that can considerably keep its sequence order information or capture its key features. The reasons are as follows: (1) If using the sequential model, *i.e.*, the model in which all the samples are represented by their original sequences, it is hardly able to train a machine that can cover all the possible cases concerned, as elaborated in [36]; (2) All the existing computational algorithms, such as optimization approach [37], correlation-angle approach [38], covariance discriminant (CD) [39], neural network [40], K-nearest neighbor (KNN) [41], OET-KNN [42], SLLE algorithm [43], random forest [44], Fuzzy K-nearest neighbor [45], and ML-KNN algorithm [46], can only handle vector but not sequence samples.

However, a vector defined in a discrete model may completely lose the sequence-order information as elaborated in [47,48]. To cope with such a dilemma, the approach of pseudo amino acid composition [36,49] or Chou’s PseAAC [50,51] was proposed. Ever since it was introduced in 2001 [36], the concept of PseAAC has been penetrating into nearly all the areas of computational biology (see, e.g., [52,53,54,55,56] as well as a long list of references cited in [48,57] and a recent review [58]). It has also been selected as a special topic for a special issue on “drug development and biomedicine” [59]. Recently, the concept of PseAAC was further extended to represent the feature vectors of DNA and nucleotides [60,61,62,63,64]. Because of its being widely and increasingly used, three types of open access soft-ware, called “PseAAC-Builder” [65], “propy” [50], and “PseAAC-General” [57], were established: the former two are for generating various modes of Chou’s special PseAAC; while the 3rd one for those of Chou’s general PseAAC.

According to [12], PseAAC can be generally formulated as:

(9) $$P={\left[\Psi_{1}\;\Psi_{2}\;\cdots \;\Psi_{u}\;\cdots \;\Psi_{\Omega }\right]}^{T}$$

where T is the transpose operator, while Ω an integer to reflect the vector’s dimension. The value of Ω as well as the components $\Psi_{u}=\left(u=1,2,\cdots ,\text{ Ω}\right)$ in Equation (9) will depend on how to extract the desired information from a peptide sequence. Below, we are to describe how to extract the useful information from the aforementioned benchmark datasets (*cf.* Equations (4) and (5)) to define the working protein segments via Equation (9). For the convenience of formulation below, we convert the (2ξ+1)-tuple peptide in Equation (6) to:

(10) $$P_{\xi }=R_{1}R_{2}R_{3}R_{4}R_{5}R_{6}R_{7}\cdots R_{\left(2\xi +1\right)}$$

#### 2.3.1. Physicochemical Properties

Different types of amino acid in the above equation may have different physicochemical properties. In this study, we considered the following seven physicochemical properties: (1) hydrophobicity [66] or $\Phi^{\left(1\right)}$; (2) hydrophicility [67] or $\;\Phi^{\left(2\right)}$; (3) side-chain volume [68] or $\Phi^{\left(3\right)}$; (4) polarity [69] or $\Phi^{\left(4\right)}$; (5) polarizability [70] or $\Phi^{\left(5\right)}$; (6) solvent-accessible surface area (SASA) [71] or $\Phi^{\left(6\right)}$; and (7) side-chain net charge index (NCI) [72] or $\Phi^{\left(7\right)}$. Their numerical values are given in Table 2.

**Table 2:** The original values of the seven physicochemical properties for each amino acid.

Amino Acid Code	Physicochemical Property (*cf.* Equation (11)) ^a^						
	$\Phi^{\left(1\right)}$	$\Phi^{\left(2\right)}$	$\Phi^{\left(3\right)}$	$\Phi^{\left(4\right)}$	$\Phi^{\left(5\right)}$	$\Phi^{\left(6\right)}$	$\Phi^{\left(7\right)}$
	H1	H2	V	P1	P2	SASA	NCI
A	0.62	−0.5	27.5	8.1	0.046	1.181	0.007187
C	0.29	−1	44.6	5.5	0.128	1.461	−0.03661
D	−0.9	3	40	13	0.105	1.587	−0.02382
E	−0.74	3	62	12.3	0.151	1.862	0.006802
F	1.19	−2.5	115.5	5.2	0.29	2.228	0.037552
G	0.48	0	0	9	0	0.881	0.179052
H	−0.4	−0.5	79	10.4	0.23	2.025	−0.01069
I	1.38	−1.8	93.5	5.2	0.186	1.81	0.021631
K	−1.5	3	100	11.3	0.219	2.258	0.017708
L	1.06	−1.8	93.5	4.9	0.186	1.931	0.051672
M	0.64	−1.3	94.1	5.7	0.221	2.034	0.002683
N	−0.78	2	58.7	11.6	0.134	1.655	0.005392
P	0.12	0	41.9	8	0.131	1.468	0.239531
Q	−0.85	0.2	80.7	10.5	0.18	1.932	0.049211
R	−2.53	3	105	10.5	0.291	2.56	0.043587
S	−0.18	0.3	29.3	9.2	0.062	1.298	0.004627
T	−0.05	−0.4	51.3	8.6	0.108	1.525	0.003352
V	1.08	−1.5	71.5	5.9	0.14	1.645	0.057004
W	0.81	−3.4	145.5	5.4	0.409	2.663	0.037977
Y	0.26	−2.3	117.3	6.2	0.298	2.368	0.023599

^a^ H1, hydrophobicity; H2, hydrophilicity; V, volume of side chains; P1, polarity; P2, polarizability; SASA, solvent accessible surface area; NCI, net charge index of side chains.

Thus, the peptide segment $P_{\xi }$ of Equation (10) can be encoded into seven different numerical series, as formulated by:

(11) $$P_{\xi }=\{\begin{array}{c} \Phi_{1}^{\left(1\right)}\Phi_{2}^{\left(1\right)}\Phi_{3}^{\left(1\right)}\Phi_{4}^{\left(1\right)}\Phi_{5}^{\left(1\right)}\Phi_{6}^{\left(1\right)}\Phi_{7}^{\left(1\right)}\cdots \Phi_{2\xi +1}^{\left(1\right)}\\ \Phi_{1}^{\left(2\right)}\Phi_{2}^{\left(2\right)}\Phi_{3}^{\left(2\right)}\Phi_{4}^{\left(2\right)}\Phi_{5}^{\left(2\right)}\Phi_{6}^{\left(2\right)}\Phi_{7}^{\left(2\right)}\cdots \Phi_{2\xi +1}^{\left(2\right)}\\ \Phi_{1}^{\left(3\right)}\Phi_{2}^{\left(3\right)}\Phi_{3}^{\left(3\right)}\Phi_{4}^{\left(3\right)}\Phi_{5}^{\left(3\right)}\Phi_{6}^{\left(3\right)}\Phi_{7}^{\left(3\right)}\cdots \Phi_{2\xi +1}^{\left(3\right)}\\ \Phi_{1}^{\left(4\right)}\Phi_{2}^{\left(4\right)}\Phi_{3}^{\left(4\right)}\Phi_{4}^{\left(4\right)}\Phi_{5}^{\left(4\right)}\Phi_{6}^{\left(4\right)}\Phi_{7}^{\left(4\right)}\cdots \Phi_{2\xi +1}^{\left(4\right)}\\ \Phi_{1}^{\left(5\right)}\Phi_{2}^{\left(5\right)}\Phi_{3}^{\left(5\right)}\Phi_{4}^{\left(5\right)}\Phi_{5}^{\left(5\right)}\Phi_{6}^{\left(5\right)}\Phi_{7}^{\left(5\right)}\cdots \Phi_{2\xi +1}^{\left(5\right)}\\ \Phi_{1}^{\left(6\right)}\Phi_{2}^{\left(6\right)}\Phi_{3}^{\left(6\right)}\Phi_{4}^{\left(6\right)}\Phi_{5}^{\left(6\right)}\Phi_{6}^{\left(6\right)}\Phi_{7}^{\left(6\right)}\cdots \Phi_{2\xi +1}^{\left(6\right)}\\ \Phi_{1}^{\left(7\right)}\Phi_{2}^{\left(7\right)}\Phi_{3}^{\left(7\right)}\Phi_{4}^{\left(7\right)}\Phi_{5}^{\left(7\right)}\Phi_{6}^{\left(7\right)}\Phi_{7}^{\left(7\right)}\cdots \Phi_{2\xi +1}^{\left(7\right)} \end{array}$$

where $\Phi_{1}^{\left(1\right)}$ is the hydrophobicity value of $R_{1}$ in Equation (10), $\Phi_{2}^{\left(2\right)}$ the hydrophilicity value of $R_{2}$, and so forth. Note that before substituting the physicochemical values of Table 2 into Equation (10), they all are subjected to the following standard conversion:

(12) $$\Phi_{i}^{\left(\xi \right)}⇐\;\frac{\Phi_{i}^{\varphi }-\langle \Phi_{i}^{\varphi }\rangle }{\text{SD}\left(\Phi_{i}^{\varphi }\right)}\;\left(\varphi =1,\;2,\;\cdots ,7;\;i=1,\;2,\;\cdots ,2\xi +1\right)$$

where the symbol 〈 〉 means taking the average for the quantity therein over the 20 amino acid types, and SD means the corresponding standard deviation. The converted values via Equation (12) will have zero mean value over the 20 amino acid types, and will remain unchanged if they go through the same standard conversion procedure again.

#### 2.3.2. Stationary Wavelet Transform Approach

The low-frequency internal motion is a very important feature of biomacromolecules (see, e.g., [73,74,75]. Many marvelous biological functions in proteins and DNA and their profound dynamic mechanisms, such as switch between active and inactive states [76,77], cooperative effects [78], allosteric transition [79,80,81], intercalation of drugs into DNA [82], and assembly of microtubules [83], can be revealed by studying their low-frequency internal motions as summarized in a comprehensive review [84]. Low frequency Fourier spectrum was also used by Liu *et al.* [85] to develop a sequence-based method for predicting membrane protein types. In view of this, it would be intriguing to introduce the stationary wavelet transform into the current study.

**Figure 2:**
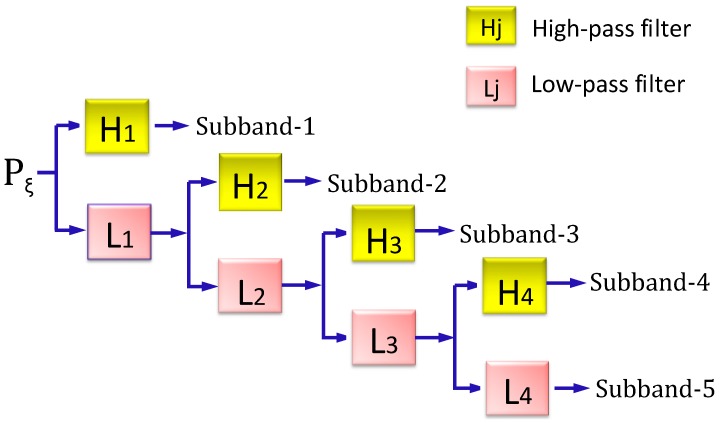
A schematic drawing to illustrate the procedure of multi-level SWT (stationary wavelets transform). See Equations (10)–(12) as well as the relevant text for further explanation.

The stationary wavelet transform (SWT) [86] is a wavelet transform algorithm designed to overcome the lack of shift-invariance of the discrete wavelet transform (DWT) [87]. Shift-invariance is achieved by removing the downsamplers and upsamplers in the DWT and upsampling (insert zero) the filter coefficients by a factor of $2^{j-1}\;$in the *j*-th level of the algorithm. The SWT is an inherently redundant scheme as the output of each level of SWT contains the same number of samples as the input-so for a decomposition of N levels there is a redundancy of N in the wavelet coefficients. Shown in Figure 2 is the block diagram depicting the digital implementation of SWT. As we can see from the figure, the input peptide segment is decomposed recursively in the low-frequency part.

The concrete procedure of using the SWT to denote the (2ξ+1)-tuple peptides is as follows. For each of the (2ξ+1)-tuple peptides generated by sliding the scaled window[−ξ, +ξ] along the protein chain concerned, the SWT was used to decompose it based on the amino acid values encoded by the seven physicochemical properties as given in Equation (11). Daubechies of number 1 (Db1) wavelet was selected because its wavelet possesses a lower vanish moment and easily generates non-zero coefficients for the ensemble learning framework that will be introduced later.

Preliminary tests indicated that, when ξ=7, *i.e.*, the working segments are 15-tuple peptides, the outcomes thus obtained were most promising. Accordingly, we only consider the case of ξ=7 hereafter.

Using the SWT approach, we have generated five sub-bands (Figure 2), each of which has four coefficients: (1) $\alpha_{i}$, the maximum of the wavelet coefficients in the sub-band i (1,2, ⋯5); (2) $\beta_{i},$ the corresponding mean of the wavelet coefficients; (3) $\gamma_{i}$, the corresponding minimum of the wavelet coefficients; (4) $\delta_{i}$, the corresponding standard deviation of the wavelet coefficients. Therefore, for each working segment, we can get a feature vector that contains Ω=5×4=20 components by using each of the seven physicochemical properties of Equation (11). In other words, we have seven different modes of PseAAC as given below:

(13) $$P^{\left(k\right)}={\left[\Psi_{1}^{\left(k\right)}\;\Psi_{2}^{\left(k\right)}\;\Psi_{3}^{\left(k\right)}\;\cdots \;\Psi_{u}^{\left(k\right)}\;\cdots \;\Psi_{20}^{\left(k\right)}\right]}^{T}\;\left(k=1,\;2,\;\cdots ,\;7\right)$$

where:

(14) $$\Psi_{\mu }^{\left(k\right)}=\{\begin{array}{cc} \alpha_{\mu }^{\left(k\right)} & \mathrm{when}\;1\leq \mu \leq 5\\ \beta_{\mu -5}^{\left(k\right)} & \mathrm{when}\;6\leq \mu \leq 10\\ \lambda_{\mu -10}^{\left(k\right)} & \mathrm{when}\;11\leq \mu \leq 15\\ \delta_{\mu -15}^{\left(k\right)} & \mathrm{when}\;11\leq \mu \leq 20 \end{array}$$

### 2.4. Optimizing Imbalanced Training Datasets

In the current benchmark dataset $S_{\text{surf}}$ or $S_{\text{all }}$, the negative subset $S_{\text{all}}^{-}\;\text{or}\;S_{\text{surf}}^{-}\;$ is much larger than the corresponding positive subset $S_{\text{all}}^{+}\;\text{or}\;S_{\text{surf}}^{+}$ as can be seen by the following equation:

(15) $$\{\begin{array}{cc} S_{\text{surf}}\left(13771\right)=S_{\text{surf}}^{+}\left(2828\right)\cup^{\text{​}}S_{\text{surf}}^{-}\left(10943\right) & \mathrm{for}\;\mathrm{surface}\;\mathrm{residuces}\;\\ S_{\text{all }}\left(27442\right)=S_{\text{all}}^{+}\left(2828\right)\cup^{\text{​}}S_{\text{all}}^{-}\left(24614\right) & \text{for all residues} \end{array}$$

where the figures in the parentheses denote the sample numbers taken from Section 2.1. As we can see from Equation (15), the numbers of the negative samples are nearly nine and four times the sizes of the corresponding positive samples for the all-residue and surface-residue benchmark datasets, respectively.

Although this might reflect the real world in which the non-binding sites are always the majority compared with the binding ones, a predictor trained by such a highly skewed benchmark dataset would inevitably have the bias consequence that many binding sites might be mispredicted as non-binding ones [88]. Actually, what is really the most intriguing information for us is the information about the binding sites. Therefore, it is important to find an effective approach to optimize the unbalanced training dataset and minimize this kind of bias consequence. To realize this, we took the following procedures.

First, we used the K-Nearest Neighbors Cleaning (KNNC) treatment to remove some redundant negative samples from the negative subset so as to reduce its statistical noise. The detailed process can be described below: (i) for each of the samples in the negative subset $S^{-}$ find its *K* nearest neighbors, where *K* may be any integer (such as 3 or 8), and its final value will be discussed later; (ii) if one of its *K* nearest neighbors belongs to the positive subset$\;S^{+}$, remove the negative sample from $S^{-}$. A similar method, called the Neighborhood Cleaning Rule (NCR), was also been used by Laurikkala *et al.* [89], Xiao *et al.* [90], and Liu *et al.* [91] although their details are different with the current practice. Also, the current KNNC approach is more flexible because it contains a variable *K* and hence can be used to deal with various different training datasets.

Second, we used the Inserting Hypothetical Training Samples (IHTS) treatment to add some hypothetical positive samples into the positive subset so as to enhance the ability in identifying the interactive pairs. For the details of how to generate the hypothetical training samples, see the Monte Calo samples expanding approach in [92,93], or seed-propagation approach in [94], or the SMOTE (synthetic minority over-sampling technique) approach in [95].

After the above two treatments, we can change an original highly skewed training dataset to a balanced training dataset with its positive subset and negative subset having exactly the same size.

It is instructive to point out that the hypothetical samples generated via the IHTS treatment can only be expressed by their feature vectors as defined in Equation (13), but not the real peptide segment samples as given by Equations (6) or (10). Nevertheless, it would be perfectly reasonable to do so because the data directly used to train a predictor were actually the samples’ feature vectors but not their sequence codes. This is the key to optimize an imbalanced benchmark dataset in the current study, and the rationale of such an interesting approach will be further elucidated later.

### 2.5. Fusing Multiple Physicochemical Properties

The random forest (RF) algorithm is a powerful algorithm, which has been used in many areas of computational biology (see, e.g., [44,96,97]). The detailed procedures and formulation of RF have been very clearly described in [98], and hence there is no need to repeat here.

As shown in Equations (11)–(13), a peptide segment concerned in the current study can be formulated with seven different PseAAC modes, each of which can be used to train the random forest predictor after the KNNC and IHTS procedures. Accordingly, we have a total of seven individual predictors for identifying PPBS, as formulated by:

(16) PPBS individual predictor=ℝF(k) (k=1, 2, ⋯, 7)

where ℝF(k) represents the random forest predictor based on the *k*-th physicochemical property (*cf.* Equation (13)).

**Figure 3:**
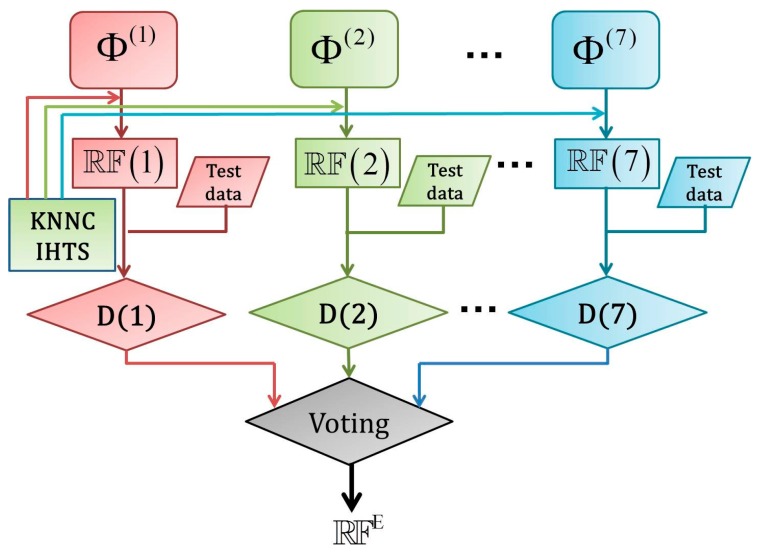
A flowchart to illustrate the ensemble classifier of Equation (17) that exploits all the different groups of features, where D(1) means the decision made by ℝF(1), D(2) means the decision made by ℝF(2), and so forth. See the text as well as Equations (11) and (16) for further explanation.

Now, the problem is how to combine the results from the seven individual predictors to maximize the prediction quality. As indicated by a series of previous studies, using the ensemble classifier formed by fusing many individual classifiers can remarkably enhance the success rates in predicting protein subcellular localization [99,100] and protein quaternary structural attribute [101]. Encouraged by the previous investigators’ studies, here we are also developing an ensemble classifier by fusing the seven individual predictors ℝF(k) (k=1,2,⋯,7) through a voting system, as formulated by:

(17) $$\mathbb{R}F^{E}=\mathbb{R}F\left(1\right)\forall \cdots \forall \mathbb{R}F\left(7\right)=\forall_{k=1}^{7}\mathbb{R}F\left(k\right)$$

where $\mathbb{R}F^{E}$ stands for the ensemble classifier, and the symbol ∀ for the fusing operator. For the detailed procedures of how to fuse the results from the seven individual predictors to reach a final outcome via the voting system, see Equations (30)–(35) in [27], where a crystal clear and elegant derivation was elaborated and hence there is no need to repeat here. To provide an intuitive picture, a flowchart is given in Figure 3 to illustrate how the seven individual RF predictors are fused into the ensemble classifier.

The final predictor thus obtained is called “**iPPBS-Opt**”, where “i” stands for “identify”, “PPBS” for “protein-protein binding site”, and “Opt” for “optimizing” training datasets. Note that the **iPPBS-Opt** predictor contains a parameter *K*, reflecting how many nearest neighbors should be considered in removing the redundant negative samples from the training dataset during the KNNC treatment (*cf.* Section 2.4). Its final value is determined by maximizing the overall success rate via cross-validation, as will be described later.

## 3. Result and Discussion

As pointed out in the Introduction section, one of the important procedures in developing a predictor is how to properly and objectively evaluate its anticipated success rates [12]. Towards this, we need to consider the following two aspects: one is what kind of metrics should be used to quantitatively measure the prediction accuracy; the other is what kind of test method should be adopted to derive the metrics values, as elaborated below.

### 3.1. Metrics for Measuring Success Rates

For measuring the success rates in identifying PPBS, a set of four metrics are usually used in literature. They are: (1) overall accuracy or Acc; (2) Mathew’s correlation coefficient or MCC; (3) sensitivity or Sn; and (4) specificity or Sp (see, e.g., [102]). Unfortunately, the conventional formulations for the four metrics are not quite intuitive for most experimental scientists, particularly the one for MCC. Interestingly, by using the symbols and derivation as used in [103] for studying signal peptides, the aforementioned four metrics can be formulated by a set of equations given below [14,30,60,61,104]:

(18) $$\{\begin{array}{ll} \mathrm{Sn}=1-\frac{N_{-}^{+}}{N^{+}} & 0\leq \mathrm{Sn}\leq 1\\ \mathrm{Sp}=1-\frac{N_{+}^{-}}{N^{-}} & 0\leq \mathrm{Sp}\leq 1\\ \mathrm{Acc}=\Lambda =1-\frac{N_{-}^{+}+N_{+}^{-}}{N^{+}+N^{-}} & 0\leq \mathrm{Acc}\leq 1\\ \mathrm{Mcc}=\frac{1-(\frac{N_{-}^{+}+N_{+}^{-}}{N^{+}+N^{-}})}{\sqrt{\left(1+\frac{N_{+}^{-}-N_{-}^{+}}{N^{+}}\right)\left(1+\frac{N_{-}^{+}-N_{+}^{-}}{N^{-}}\right)}} & -1\leq \mathrm{Mcc}\leq 1 \end{array}$$

where $N^{+}$ represents the total number of PPBSs investigated whereas $N_{-}^{+}$ the number of true PPBSs incorrectly predicted to be of non-PPBS; $N^{-}$ the total number of the non-PPBSs investigated whereas $N_{+}^{-}$ the number of non-PPBSs incorrectly predicted to be of PPBS.

According to Equation (18), it is crystal clear to see the following. When $N_{-}^{+}=0$ meaning none of the true PPBSs are incorrectly predicted to be of non-PPBS, we have the sensitivity Sn=1. When $N_{-}^{+}=N^{+}$ meaning that all the PPBSs are incorrectly predicted to be of non-PPBS, we have the sensitivity Sn=0. Likewise, when $N_{+}^{-}$ = 0 meaning none of the non-PPBSs are incorrectly predicted to be of PPBS, we have the specificity Sp=1; whereas $N_{+}^{-}\;$=$\;N^{-}$ meaning that all the non-PPBSs are incorrectly predicted to be of PPBS, we have the specificity Sp=0. When $N_{-}^{+}=N_{+}^{-}=0$ meaning that none of PPBSs in the positive dataset and none of the non-PPBSs in the negative dataset are incorrectly predicted, we have the overall accuracy Acc=1 and MCC=1; when $N_{-}^{+}=N^{+}$ and $N_{+}^{-}\;$=$\;N^{-}$ meaning that all the PPBSs in the positive dataset and all the non-PPBSs in the negative dataset are incorrectly predicted, we have the overall accuracy Acc=0 and MCC=−1; whereas when $N_{-}^{+}=N^{+}/2$ and $N_{+}^{-}$ = $N^{-}/2$ we have Acc=0.5 and MCC=0 meaning no better than random guess. As we can see from the above discussion, it would make the meanings of sensitivity, specificity, overall accuracy, and Mathew’s correlation coefficient much more intuitive and easier-to-understand by using Equation (18), particularly for the meaning of MCC.

It should be pointed out, however, the set of metrics as defined in Equation (18) is valid only for the single-label systems. For the multi-label systems whose emergence has become more frequent in system biology [46,105,106] and system medicine [107], a completely different set of metrics as defined in [108] is needed.

### 3.2. Cross-Validation and Target Cross-Validation

Once established the evaluation metrics, the next issue is the selection of the most appropriate validation method should be used to derive the values of these metrics. Three cross-validation methods are often used to derive metrics values in statistical prediction: the independent dataset test, subsampling (or K-fold cross-validation) test, and jackknife test [109]. Of the three the jackknife test is deemed the least arbitrary as it can always yield a unique outcome for a given benchmark dataset, as elucidated in [12] and demonstrated by Equations (28)–(32) therein. Accordingly, the jackknife test has been widely recognized and increasingly used by investigators to examine the quality of various predictors (see, e.g., [46,53,54,110,111,112,113,114,115]. However, to reduce the computational time, in this study we adopted the 10-fold cross-validation, as done by most investigators with SVM and random forests algorithms as the prediction engine.

When conducting the 10-fold cross-validation for the current predictor **iPPBS-Opt,** however, some special consideration is needed. This is because a dataset, after optimized by the KNNC and ITHTS treatments, may miss many experimental negative samples and contain some hypothetical positive samples. It would be fine to use such a dataset to train a predictor, but not for validation. Since the validation should be conducted based on all the experimental data in the benchmark dataset but not on the added hypothetical samples nor only on the data in the reduced negative subset, a special cross-validation, the so-called target cross-validation, has been introduced here. During the target cross-validation process for the positive samples, only the experiment-confirmed samples are singled out as the targets (or test samples) for validation; but during the target cross-validation process for the negative samples, even all the excluded experimental data are taken into account. The detailed procedures of the target 10-fold cross-validation are as follows:

*Step 1*. Before optimizing the original benchmark dataset, both its positive and negative subsets were randomly divided into 10 parts with about the same size. For example, for the all-residue benchmark dataset $S_{\text{all }}$, after such evenly division we have:

(19) $$S_{\text{all }}=S_{\text{all}\left(1\right)}\cup^{\text{​}}S_{\text{all}\left(2\right)}\cup^{\text{​}}\cdots \cup^{\text{​}}S_{\text{all}\left(10\right)}=\;{\cup^{\text{​}}}_{i=1}^{10}S_{\text{all}\left(i\right)}$$

and:

(20) $$S_{\text{all}\left(1\right)}≜\;S_{\text{all}\left(2\right)}≜\;\cdots ≜\;=S_{\text{all}\left(10\right)}\;$$

where the symbol ≜ means that the divided 10 datasets are about the same in size, and so are their subsets.

*Step 2*. One of the 10 sets, say $S_{\text{all}\left(1\right)},$ was singled out as the testing dataset and the remaining nine sets as the training dataset.

*Step 3*. The training set was optimized using the KNNC and IHTS treatments as described in Section 2.4. After such a process, the original imbalanced training dataset would become a balanced one; *i.e.*, its positive subset and negative subset would contain a same number of samples. Note that although the starting value for K in the KNNC treatment could be arbitrary, the following empirical approach might be of help to reduce the time for finally finding its optimal value. Suppose the starting value for *K* is K(0), then we have according to our experience

(21) $$K\left(0\right)=\text{Int}\left[\frac{N^{-}}{N^{+}}\right]$$

where $\;N^{+}$ and $N^{-}\;$ are the numbers of the total positive and negative samples in the benchmark dataset, respectively, and Int is the “integer truncation operator” meaning to take the integer part for the number in the brackets right after it [116]. Substituting the data of Equation (15) into Equation (21), we obtained K(0)=3 or 8 for the surface-residue case or of all-residue case, respectively.

*Step 4*. Use the aforementioned balanced dataset to train the operation engine, followed by applying the **iPPBS-Opt** predictor to calculate the prediction scores for the testing dataset, which had been singled out in Step 2 before the optimized treatment and hence contained the experiment-confirmed samples only.

*Step 5*. Repeat Steps 2–4 until all the 10 divided sets had been singled out one-by-one for testing validation.

*Step 6*. Substituting the scores obtained from the above 10-round tests into Equation (18) to calculate Sn, Sp, Acc, and MCC. The metrics values thus obtained should be a function of *K*; for instance, the overall accuracy Acc can be expressed as Acc(*K*).

**Figure 4:**
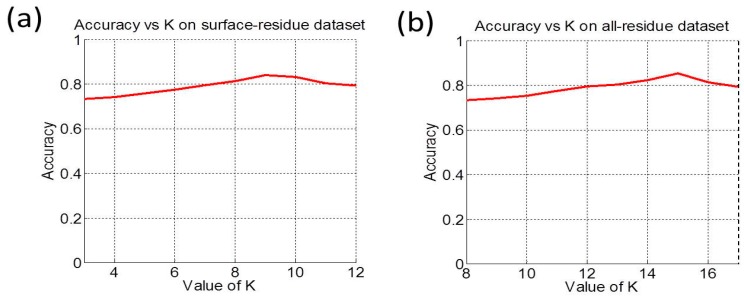
A plot of Acc *vs.* *K* for (**a**) the surface-residue benchmark dataset (*cf.* Equation (4)); and (**b**) the all-residue benchmark dataset (*cf.* Equation (5)). It can be seen from panel (**a**) that the overall accuracy reaches its peak at K=9, and from panel (**b**) that the overall accuracy reaches its peak at K=15 .

*Step 7*. Repeat Steps 2–6 by increasing *K* with a gap of 1, we consecutively obtained Acc(3), Acc(4), …., Acc(12) for the surface-residue case or Acc(8), Acc(9), …., Acc(17) for the all-residue case, respectively (Figure 4). The value of *K* that maximized Acc would be taken for **iPPBS-Opt** in the current study, as given in the footnote c of Table 3.

It is instructive to emphasize again that it is absolutely fair to use the above 10-fold cross-validation steps to compare the current predictor with the existing ones. This is because all the predictors concerned were tested using exactly the same experiment-confirmed samples and that all the added hypothetical samples had been completely excluded from the testing datasets.

**Table 3:** Comparison of the **iPPBS-Opt** with the other existing methods via the 10-fold cross-validation on the surface-residue benchmark dataset (Equation (4)) and the all-residue benchmark dataset (Equation (5)).

Benchmark Dataset	Method	Acc (%)	MCC	Sn (%)	Sp (%)	AUC
Surface-residue	Deng ^a^	N/A	0.3456	76.77	63.16	0.7976
	Chen ^b^	75.09	0.4248	43.81	92.12	0.8004
	iPPBS-PseAAC ^c^	**84.04**	**0.5821**	58.26	94.14	**0.8934**
All-residue	Deng ^a^	N/A	0.3763	76.33	78.61	0.8465
	Chen ^b^	73.77	0.3286	24.95	96.52	0.8001
	iPPBS-PseAAC ^c^	**85.45**	**0.4662**	39.14	96.66	**0.8820**

^a^ Results reported by Deng *et al.* [10]; ^b^ Results reported by Chen *et al.* [11]; ^c^ Results obtained on the same testing dataset by the current predictor **iPPBS-Opt** with its parameter K=9 for the surface-residue benchmark dataset $S_{\text{surf}}$ (*cf.* Equation (4)) and K=15  for the all-residue benchmark dataset $S_{\text{all}}$ (*cf.* Equation (5)). Also see Figure 4 for the details.

### 3.3. Comparison with the Existing Methods

Listed in Table 3 are the values of the four metrics (*cf.* Equation (18)) obtained by the current **iPPBS-Opt** predictor using the target 10-fold cross-validation on the surface-residue benchmark dataset $S_{\text{surf}}$ (Equation (4)) and the all-residue benchmark dataset $S_{\text{all}}$ (Equation (5)), respectively. See S1 Dataset for the details of the two benchmark datasets. For facilitating comparison, the corresponding results obtained by the existing methods [10,11] are also given there.

As we can see from the table, the new predictor **iPPBS-Opt** proposed in this paper remarkably outperformed its counterparts, particularly in Acc and MCC; the former stands for the overall accuracy, and the latter for the stability. At the first glance, although the value of Sn by Deng *et al.*’s method [10] is higher than that of the current predictor when tested by the surface-residue benchmark dataset, its corresponding Sp value is more than 30% lower than that of the latter, indicating the method [10] is very unstable with extremely high noise.

**Figure 5:**
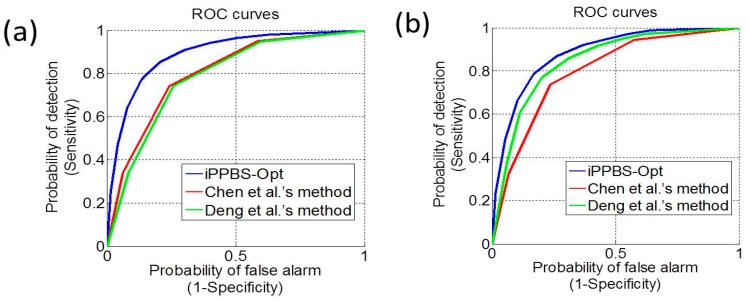
The ROC (Receiver Operating Characteristic) curves to show the 10-fold cross validation by **iPPBS-Opt**, Deng *et al.*’s method [10], and Chen *et al.*’s method [11] on (**a**) surface-residue benchmark dataset; and (**b**) the all-residue benchmark dataset. As shown on the figure, the area under the ROC curve for **iPPBS-Opt** is obviously larger than those of their counterparts, indicating a clear improvement of the new predictor in comparison with the existing ones.

Because graphic approaches can provide useful intuitive insights (see, e.g., [117,118,119,120,121,122]), here we also provide a graphic comparison of the current predictor with their counterparts via the Receiver Operating Characteristic (ROC) plot [123], as shown in (Figure 5). According to ROC [123], the larger the area under the curve (AUC), the better the corresponding predictor is. As we can see from the figure, the area under the ROC curve of the new predictor is remarkably greater than those of their counterparts fully consistent with the AUC values listed on Table 3, once again indicating a clear improvement of the new predictor in comparison with the existing ones.

All the above facts have shown that **iPPBS-Opt** is really a very promising predictor for identifying protein-protein binding sites. Or at the very least, it can play a complementary role to the existing prediction methods in this area. Particularly, none of the existing predictors has provided a web server. In contrast to this, a user-friendly and publically accessible web server has been established for **iPPBS-Opt** at http://www.jci-bioinfo.cn/iPPBS-Opt, which is no doubt very useful for the majority of experimental scientist in this or related areas without the need to follow the complicated mathematical equations.

Why could the proposed method be so powerful? The reasons are as follows: First, the KNNC and IHTS treatments have been introduced to optimize the training datasets, so as to avoid many misprediction events caused by the highly imbalanced training datasets used in previous studies. Second, the ensemble technique has been utilized in this study to select the most relevant one from seven classes of different physicochemical properties. Third, the wavelets transform technique has been applied to extract some important key features, which are deeply hidden in complicated protein sequences. This is just like the studies in dealing with the extremely complicated internal motions of proteins, it is the key to grasp the low-frequency collective motion [74,75] for in-depth understanding or revealing the dynamic mechanisms of their various important biological functions [84], such as cooperative effects [78], allosteric transition [80,81], assembly of microtubules [83], and switch between active and inactive states [76]. Fourth, the PseAAC approach has been introduced to formulate the statistical samples, which has been proved very useful not only in dealing with protein/peptide sequences, but also in dealing with DNA/RNA sequences, as elaborated in a recent review paper [124].

### 3.4. Web Server and User Guide

**Figure 6:**
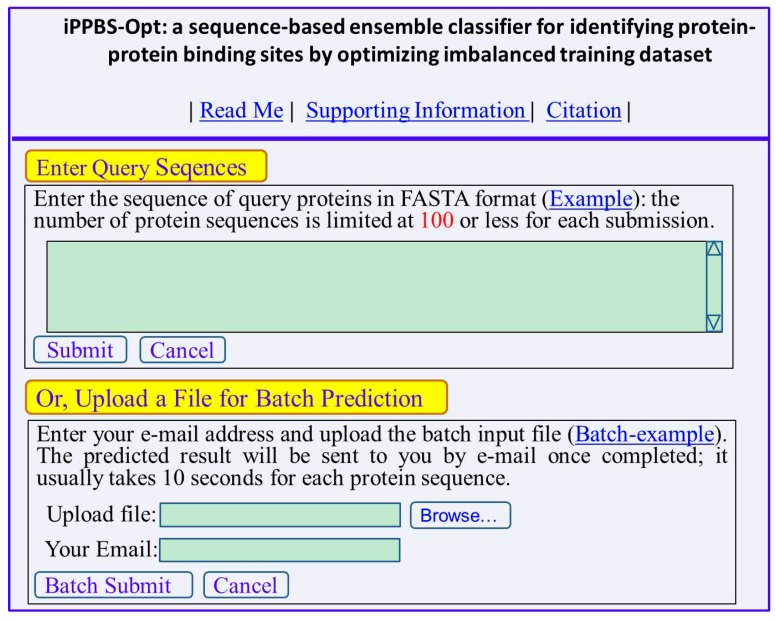
A semi-screenshot of the top page for the web server **iPPBS-Opt** at http://www.jci-bioinfo.cn/iPPBS-Opt.

To enhance the value of its practical applications, a web-server for **iPPBS-Opt** has been established at http://www.jci-bioinfo.cn/iPPBS-Opt. Furthermore, to maximize the convenience for the majority of experimental scientists, a step-to-step guide is provided below:

*Step 1*. Opening the web-server at http://www.jci-bioinfo.cn/iPPBS-Opt, you will see the top page of **iPPBS-Opt** on your computer screen, as shown in Figure 6. Click on the Read Me button to see a brief introduction about the i**PPBS-Opt** predictor.

*Step 2*. Either type or copy/paste the query protein sequences into the input box at the center of Figure 6. The input sequence should be in the FASTA format. For the examples of sequences in FASTA format, click the Example button right above the input box.

*Step 3.* Click on the Submit button to see the predicted result. For example, if you use the two query protein sequences in the Example window as the input, after 20 s or so, you will see the following on the screen of your computer: (1) Sequence-1 contains 109 amino acid residues, of which 11 are highlighted with red, meaning belonging to binding site; (2) Sequence-2 contains 275 residues, of which 25 are highlighted with red, belonging binding site. All these predicted results are fully consistent with experimental observations except for residues 53 in sequence-1 and residues 62 and 249 in sequence-2 that are overpredicted.

*Step 4.* As shown on the lower panel of Figure 6, batch prediction can also be selected by entering an e-mail address and the desired batch input file (in FASTA format naturally) via the Browse button. To see the sample of batch input file, click on the button Batch-example.

*Step 5*. Click on the Citation button to find the relevant papers that document the detailed development and algorithm of **iPPBS-Opt**.

*Step 6.* Click the Supporting Information button to download the benchmark dataset used in this study.

## 4. Conclusions

It is a very effective approach to optimize the training dataset via the KNNC treatment and IHTS treatment to enhance the prediction quality in identifying the protein-protein binding sites. This is because the training datasets constructed in this area without undergoing such an optimization procedure are usually extremely skewed and unbalanced, with the negative subset being overwhelmingly larger than the positive one. It is anticipated that the **iPPBS-Opt** web server presented in this paper will become a very useful high throughput tool for identifying protein-protein binding sites, or at the very least, a complementary tool to the existing prediction methods in this area.

## Supplementary Materials

Supplementary materials can be accessed at: http://www.mdpi.com/1420-3049/21/1/95/s1.
