# Supplementary material for: iPPBS-Opt: A Sequence-Based Ensemble Classifier for Identifying Protein-Protein Binding Sites by Optimizing Imbalanced Training Datasets
Source: Molecules. 2016 Jan 19;21(1):95. doi: 10.3390/molecules21010095 (PMC6274413; doi:10.3390/molecules21010095)
Supplement: Supplementary file 1 [file molecules-21-00095-s001.pdf]

Supplementary Materials: iPPBS−Opt: A Sequence−Based Ensemble Classifier for Identifying Protein−Protein Binding Sites by Optimizing Imbalanced Training Dataset

Jianhua Jia [1,2,*], Zi Liu [1], Xuan Xiao [1,2,*], Bingxiang Liu [1] and Kuo-Chen Chou [2,3]

Table S1. Dataset. List of the 99 proteins and their residues' attributions associated with the protein−protein binding sites. (1) Those labeled with 1 and 0 in column 4 are of surface and non−surface residues, respectively; (2) Those labeled with 1 and −1 in column 5 are of interface and non−interface residues, respectively.

Protein ID	Chain ID	Residue Position	Surface	Interface
1ACB	E	1	1	−1
1ACB	E	2	0	−1
1ACB	E	3	1	−1
1ACB	E	4	0	−1
1ACB	E	5	1	−1
1ACB	E	6	0	−1
1ACB	E	7	1	−1
1ACB	E	8	0	−1
1ACB	E	9	1	−1
1ACB	E	10	0	−1
1ACB	E	11	1	−1
1ACB	E	12	1	−1
1ACB	E	13	1	−1
1ACB	E	16	0	−1
1ACB	E	17	0	−1
1ACB	E	18	1	−1
1ACB	E	19	1	−1
1ACB	E	20	1	−1
1ACB	E	21	1	−1
1ACB	E	22	0	−1
1ACB	E	23	1	−1
1ACB	E	24	1	−1
1ACB	E	25	0	−1
1ACB	E	26	0	−1
1ACB	E	27	0	−1
1ACB	E	28	0	−1
1ACB	E	29	0	−1
1ACB	E	30	0	−1
1ACB	E	31	0	−1
1ACB	E	32	0	−1
1ACB	E	33	0	−1
1ACB	E	34	0	−1
1ACB	E	35	1	−1
1ACB	E	36	1	−1
1ACB	E	37	1	−1
1ACB	E	38	1	−1
1ACB	E	39	1	1
1ACB	E	40	0	−1

1ACB	E	41	1	1
1ACB	E	42	0	−1
1ACB	E	43	0	−1
1ACB	E	44	0	−1
1ACB	E	45	0	−1
1ACB	E	46	0	−1
1ACB	E	47	0	−1
1ACB	E	48	1	−1
1ACB	E	49	1	−1
1ACB	E	50	1	−1
1ACB	E	51	0	−1
1ACB	E	52	0	−1
1ACB	E	53	0	−1
1ACB	E	54	0	−1
1ACB	E	55	0	−1
1ACB	E	56	0	−1
1ACB	E	57	1	1
1ACB	E	58	0	−1
1ACB	E	59	1	1
1ACB	E	60	0	−1
1ACB	E	61	1	−1
1ACB	E	62	1	−1
1ACB	E	63	1	−1
1ACB	E	64	0	−1
1ACB	E	65	0	−1
1ACB	E	66	0	−1
1ACB	E	67	0	−1
1ACB	E	68	0	−1
1ACB	E	69	0	−1
1ACB	E	70	0	−1
1ACB	E	71	0	−1
1ACB	E	72	0	−1
1ACB	E	73	1	−1
1ACB	E	74	1	−1
1ACB	E	75	1	−1
1ACB	E	76	1	−1
1ACB	E	77	1	−1
1ACB	E	78	0	−1
1ACB	E	79	1	−1
1ACB	E	80	1	−1
1ACB	E	81	0	−1
1ACB	E	82	1	−1
1ACB	E	83	0	−1
1ACB	E	84	1	−1
1ACB	E	85	0	−1
1ACB	E	86	1	−1
1ACB	E	87	1	−1
1ACB	E	88	1	−1
1ACB	E	89	0	−1
1ACB	E	90	1	−1
1ACB	E	91	0	−1
1ACB	E	92	1	−1

1ACB	E	93	1	−1
1ACB	E	94	0	−1
1ACB	E	95	1	−1
1ACB	E	96	1	−1
1ACB	E	97	1	−1
1ACB	E	98	1	−1
1ACB	E	99	0	−1
1ACB	E	100	0	−1
1ACB	E	101	0	−1
1ACB	E	102	0	−1
1ACB	E	103	0	−1
1ACB	E	104	0	−1
1ACB	E	105	0	−1
1ACB	E	106	0	−1
1ACB	E	107	1	−1
1ACB	E	108	0	−1
1ACB	E	109	1	−1
1ACB	E	110	1	−1
1ACB	E	111	1	−1
1ACB	E	112	0	−1
1ACB	E	113	1	−1
1ACB	E	114	1	−1
1ACB	E	115	1	−1
1ACB	E	116	1	−1
1ACB	E	117	0	−1
1ACB	E	118	0	−1
1ACB	E	119	0	−1
1ACB	E	120	0	−1
1ACB	E	121	0	−1
1ACB	E	122	0	−1
1ACB	E	123	1	−1
1ACB	E	124	0	−1
1ACB	E	125	1	−1
1ACB	E	126	1	−1
1ACB	E	127	1	−1
1ACB	E	128	1	−1
1ACB	E	129	1	−1
1ACB	E	130	0	−1
1ACB	E	131	1	−1
1ACB	E	132	1	−1
1ACB	E	133	1	−1
1ACB	E	134	1	−1
1ACB	E	135	1	−1
1ACB	E	136	0	−1
1ACB	E	137	0	−1
1ACB	E	138	0	−1
1ACB	E	139	0	−1
1ACB	E	140	0	−1
1ACB	E	141	0	−1
1ACB	E	142	0	−1
1ACB	E	143	0	−1
1ACB	E	144	1	−1

1ACB	E	145	1	−1
1ACB	E	146	1	1
1ACB	E	149	1	1
1ACB	E	150	1	−1
1ACB	E	151	1	1
1ACB	E	152	0	−1
1ACB	E	153	1	−1
1ACB	E	154	1	−1
1ACB	E	155	0	−1
1ACB	E	156	0	−1
1ACB	E	157	0	−1
1ACB	E	158	0	−1
1ACB	E	159	0	−1
1ACB	E	160	0	−1
1ACB	E	161	1	−1
1ACB	E	162	0	−1
1ACB	E	163	0	−1
1ACB	E	164	1	−1
1ACB	E	165	1	−1
1ACB	E	166	1	−1
1ACB	E	167	1	−1
1ACB	E	168	0	−1
1ACB	E	169	1	−1
1ACB	E	170	1	−1
1ACB	E	171	1	−1
1ACB	E	172	0	−1
1ACB	E	173	1	−1
1ACB	E	174	1	−1
1ACB	E	175	1	1
1ACB	E	176	0	−1
1ACB	E	177	1	−1
1ACB	E	178	1	−1
1ACB	E	179	0	−1
1ACB	E	180	0	−1
1ACB	E	181	0	−1
1ACB	E	182	0	−1
1ACB	E	183	0	−1
1ACB	E	184	0	−1
1ACB	E	185	1	−1
1ACB	E	186	1	−1
1ACB	E	187	1	−1
1ACB	E	188	0	−1
1ACB	E	189	0	−1
1ACB	E	190	0	−1
1ACB	E	191	0	−1
1ACB	E	192	1	1
1ACB	E	193	0	−1
1ACB	E	194	0	−1
1ACB	E	195	0	−1
1ACB	E	196	0	−1
1ACB	E	197	0	−1
1ACB	E	198	0	−1

1ACB	E	199	0	−1
1ACB	E	200	0	−1
1ACB	E	201	0	−1
1ACB	E	202	1	−1
1ACB	E	203	1	−1
1ACB	E	204	1	−1
1ACB	E	205	1	−1
1ACB	E	206	0	−1
1ACB	E	207	0	−1
1ACB	E	208	0	−1
1ACB	E	209	0	−1
1ACB	E	210	0	−1
1ACB	E	211	0	−1
1ACB	E	212	0	−1
1ACB	E	213	0	−1
1ACB	E	214	0	−1
1ACB	E	215	0	−1
1ACB	E	216	1	1
1ACB	E	217	0	−1
1ACB	E	218	1	1
1ACB	E	219	1	−1
1ACB	E	220	0	−1
1ACB	E	221	0	−1
1ACB	E	222	1	−1
1ACB	E	223	1	−1
1ACB	E	224	0	−1
1ACB	E	225	0	−1
1ACB	E	226	0	−1
1ACB	E	227	0	−1
1ACB	E	228	0	−1
1ACB	E	229	0	−1
1ACB	E	230	0	−1
1ACB	E	231	0	−1
1ACB	E	232	0	−1
1ACB	E	233	1	−1
1ACB	E	234	0	−1
1ACB	E	235	0	−1
1ACB	E	236	1	−1
1ACB	E	237	0	−1
1ACB	E	238	0	−1
1ACB	E	239	1	−1
1ACB	E	240	1	−1
1ACB	E	241	0	−1
1ACB	E	242	0	−1
1ACB	E	243	1	−1
1ACB	E	244	1	−1
1ACB	E	245	1	−1
1ACB	I	8	1	−1
1ACB	I	9	1	−1
1ACB	I	10	0	−1
1ACB	I	11	1	−1
1ACB	I	12	1	−1

1ACB	I	13	0	−1
1ACB	I	14	1	−1
1ACB	I	15	1	−1
1ACB	I	16	1	−1
1ACB	I	17	1	−1
1ACB	I	18	0	−1
1ACB	I	19	1	−1
1ACB	I	20	1	−1
1ACB	I	21	0	−1
1ACB	I	22	1	−1
1ACB	I	23	1	−1
1ACB	I	24	1	−1
1ACB	I	25	0	−1
1ACB	I	26	1	−1
1ACB	I	27	1	−1
1ACB	I	28	1	−1
1ACB	I	29	0	−1
1ACB	I	30	1	−1
1ACB	I	31	1	−1
1ACB	I	32	0	−1
1ACB	I	33	1	−1
1ACB	I	34	0	−1
1ACB	I	35	1	−1
1ACB	I	36	0	−1
1ACB	I	37	0	−1
1ACB	I	38	1	−1
1ACB	I	39	1	−1
1ACB	I	40	1	1
1ACB	I	41	1	1
1ACB	I	42	1	1
1ACB	I	43	1	1
1ACB	I	44	1	1
1ACB	I	45	1	1
1ACB	I	46	1	1
1ACB	I	47	1	1
1ACB	I	48	1	1
1ACB	I	49	1	1
1ACB	I	50	1	−1
1ACB	I	51	0	−1
1ACB	I	52	0	−1
1ACB	I	53	0	−1
1ACB	I	54	0	−1
1ACB	I	55	1	1
1ACB	I	56	0	−1
1ACB	I	57	1	−1
1ACB	I	58	1	−1
1ACB	I	59	1	−1
1ACB	I	60	1	−1
1ACB	I	61	1	−1
1ACB	I	62	0	−1
1ACB	I	63	0	−1
1ACB	I	64	1	−1

1ACB	I	65	1	1
1ACB	I	66	1	1
1ACB	I	67	0	−1
1ACB	I	68	1	1
1ACB	I	69	0	−1
1ACB	I	70	0	−1
1AGR	A	4	1	−1
1AGR	A	5	1	−1
1AGR	A	6	1	−1
1AGR	A	7	1	−1
1AGR	A	8	1	−1
1AGR	A	9	1	−1
1AGR	A	10	1	−1
1AGR	A	11	1	−1
1AGR	A	12	1	−1
1AGR	A	13	1	−1
1AGR	A	14	1	−1
1AGR	A	15	1	−1
1AGR	A	16	1	−1
1AGR	A	17	1	−1
1AGR	A	18	1	−1
1AGR	A	19	1	−1
1AGR	A	20	1	−1
1AGR	A	21	1	−1
1AGR	A	22	1	−1
1AGR	A	23	1	−1
1AGR	A	24	1	−1
1AGR	A	25	1	−1
1AGR	A	26	0	−1
1AGR	A	27	1	−1
1AGR	A	28	1	−1
1AGR	A	29	1	−1
1AGR	A	30	0	−1
1AGR	A	31	0	−1
1AGR	A	32	1	−1
1AGR	A	33	0	−1
1AGR	A	34	0	−1
1AGR	A	35	0	−1
1AGR	A	36	0	−1
1AGR	A	37	0	−1
1AGR	A	38	0	−1
1AGR	A	39	0	−1
1AGR	A	40	0	−1
1AGR	A	41	0	−1
1AGR	A	42	0	−1
1AGR	A	43	0	−1
1AGR	A	44	0	−1
1AGR	A	45	0	−1
1AGR	A	46	0	−1
1AGR	A	47	0	−1
1AGR	A	48	0	−1
1AGR	A	49	0	−1

1AGR	A	50	0	−1
1AGR	A	51	0	−1
1AGR	A	52	0	−1
1AGR	A	53	1	−1
1AGR	A	54	0	−1
1AGR	A	55	0	−1
1AGR	A	56	0	−1
1AGR	A	57	1	−1
1AGR	A	58	1	−1
1AGR	A	59	0	−1
1AGR	A	60	0	−1
1AGR	A	61	1	−1
1AGR	A	62	1	−1
1AGR	A	63	1	−1
1AGR	A	64	1	1
1AGR	A	65	0	−1
1AGR	A	66	1	−1
1AGR	A	67	1	1
1AGR	A	68	1	−1
1AGR	A	69	0	−1
1AGR	A	70	0	−1
1AGR	A	71	0	−1
1AGR	A	72	0	−1
1AGR	A	73	0	−1
1AGR	A	74	1	1
1AGR	A	75	0	−1
1AGR	A	76	0	−1
1AGR	A	77	0	−1
1AGR	A	78	1	−1
1AGR	A	79	0	−1
1AGR	A	80	0	−1
1AGR	A	81	0	−1
1AGR	A	82	0	−1
1AGR	A	83	0	−1
1AGR	A	84	0	−1
1AGR	A	85	1	−1
1AGR	A	86	0	−1
1AGR	A	87	0	−1
1AGR	A	88	1	−1
1AGR	A	89	1	−1
1AGR	A	90	1	−1
1AGR	A	91	1	−1
1AGR	A	92	0	−1
1AGR	A	93	1	−1
1AGR	A	94	1	−1
1AGR	A	95	1	−1
1AGR	A	96	1	−1
1AGR	A	97	1	−1
1AGR	A	98	1	−1
1AGR	A	99	0	−1
1AGR	A	100	1	−1
1AGR	A	101	1	−1

1AGR	A	102	0	−1
1AGR	A	103	0	−1
1AGR	A	104	1	−1
1AGR	A	105	1	−1
1AGR	A	106	0	−1
1AGR	A	107	1	−1
1AGR	A	108	1	−1
1AGR	A	109	0	−1
1AGR	A	110	0	−1
1AGR	A	111	1	−1
1AGR	A	112	1	−1
1AGR	A	113	1	−1
1AGR	A	114	1	−1
1AGR	A	115	1	1
1AGR	A	116	1	−1
1AGR	A	117	1	−1
1AGR	A	118	0	−1
1AGR	A	119	1	−1
1AGR	A	120	1	−1
1AGR	A	121	1	−1
1AGR	A	122	0	−1
1AGR	A	123	0	−1
1AGR	A	124	0	−1
1AGR	A	125	0	−1
1AGR	A	126	0	−1
1AGR	A	127	1	−1
1AGR	A	128	1	−1
1AGR	A	129	0	−1
1AGR	A	130	0	−1
1AGR	A	131	1	−1
1AGR	A	132	0	−1
1AGR	A	133	1	−1
1AGR	A	134	0	−1
1AGR	A	135	0	−1
1AGR	A	136	1	−1
1AGR	A	137	1	−1
1AGR	A	138	0	−1
1AGR	A	139	0	−1
1AGR	A	140	1	−1
1AGR	A	141	1	−1
1AGR	A	142	1	−1
1AGR	A	143	1	−1
1AGR	A	144	1	−1
1AGR	A	145	0	−1
1AGR	A	146	1	−1
1AGR	A	147	0	−1
1AGR	A	148	1	−1
1AGR	A	149	1	−1
1AGR	A	150	0	−1
1AGR	A	151	0	−1
1AGR	A	152	0	−1
1AGR	A	153	0	−1

1AGR	A	154	0	−1
1AGR	A	155	0	−1
1AGR	A	156	1	−1
1AGR	A	157	1	−1
1AGR	A	158	0	−1
1AGR	A	159	1	−1
1AGR	A	160	1	−1
1AGR	A	161	0	−1
1AGR	A	162	0	−1
1AGR	A	163	1	−1
1AGR	A	164	1	−1
1AGR	A	165	1	−1
1AGR	A	166	0	−1
1AGR	A	167	1	−1
1AGR	A	168	0	−1
1AGR	A	169	1	−1
1AGR	A	170	1	−1
1AGR	A	171	1	−1
1AGR	A	172	0	−1
1AGR	A	173	0	−1
1AGR	A	174	0	−1
1AGR	A	175	0	−1
1AGR	A	176	0	−1
1AGR	A	177	1	−1
1AGR	A	178	1	1
1AGR	A	179	1	1
1AGR	A	180	0	−1
1AGR	A	181	1	1
1AGR	A	182	0	−1
1AGR	A	183	1	1
1AGR	A	184	1	1
1AGR	A	185	1	−1
1AGR	A	186	1	−1
1AGR	A	187	1	−1
1AGR	A	188	0	−1
1AGR	A	189	1	−1
1AGR	A	190	0	−1
1AGR	A	191	1	−1
1AGR	A	192	1	−1
1AGR	A	193	0	−1
1AGR	A	194	1	−1
1AGR	A	195	0	−1
1AGR	A	196	1	−1
1AGR	A	197	0	−1
1AGR	A	198	0	−1
1AGR	A	199	0	−1
1AGR	A	200	0	−1
1AGR	A	201	0	−1
1AGR	A	202	0	−1
1AGR	A	203	0	−1
1AGR	A	204	1	1
1AGR	A	205	1	1

1AGR	A	206	0	−1
1AGR	A	207	0	−1
1AGR	A	208	1	1
1AGR	A	209	1	1
1AGR	A	210	0	−1
1AGR	A	211	1	−1
1AGR	A	212	1	1
1AGR	A	213	0	−1
1AGR	A	214	0	−1
1AGR	A	215	1	−1
1AGR	A	216	1	−1
1AGR	A	217	0	−1
1AGR	A	218	0	−1
1AGR	A	219	0	−1
1AGR	A	220	0	−1
1AGR	A	221	0	−1
1AGR	A	222	0	−1
1AGR	A	223	0	−1
1AGR	A	224	0	−1
1AGR	A	225	0	−1
1AGR	A	226	0	−1
1AGR	A	227	0	−1
1AGR	A	228	0	−1
1AGR	A	229	0	−1
1AGR	A	230	0	−1
1AGR	A	231	0	−1
1AGR	A	232	0	−1
1AGR	A	233	0	−1
1AGR	A	234	1	1
1AGR	A	235	1	1
1AGR	A	236	0	−1
1AGR	A	237	1	1
1AGR	A	238	1	1
1AGR	A	239	0	−1
1AGR	A	240	0	−1
1AGR	A	241	0	−1
1AGR	A	242	0	−1
1AGR	A	243	1	−1
1AGR	A	244	0	−1
1AGR	A	245	0	−1
1AGR	A	246	0	−1
1AGR	A	247	1	−1
1AGR	A	248	0	−1
1AGR	A	249	0	−1
1AGR	A	250	1	−1
1AGR	A	251	1	−1
1AGR	A	252	0	−1
1AGR	A	253	0	−1
1AGR	A	254	0	−1
1AGR	A	255	1	−1
1AGR	A	256	1	−1
1AGR	A	257	1	−1

1AGR	A	258	0	−1
1AGR	A	259	1	−1
1AGR	A	260	1	−1
1AGR	A	261	0	−1
1AGR	A	262	0	−1
1AGR	A	263	0	−1
1AGR	A	264	0	−1
1AGR	A	265	0	−1
1AGR	A	266	0	−1
1AGR	A	267	0	−1
1AGR	A	268	0	−1
1AGR	A	269	0	−1
1AGR	A	270	0	−1
1AGR	A	271	1	−1
1AGR	A	272	0	−1
1AGR	A	273	0	−1
1AGR	A	274	1	−1
1AGR	A	275	1	−1
1AGR	A	276	0	−1
1AGR	A	277	0	−1
1AGR	A	278	1	−1
1AGR	A	279	1	−1
1AGR	A	280	0	−1
1AGR	A	281	1	−1
1AGR	A	282	0	−1
1AGR	A	283	1	−1
1AGR	A	284	1	−1
1AGR	A	285	0	−1
1AGR	A	286	0	−1
1AGR	A	287	1	−1
1AGR	A	288	1	−1
1AGR	A	289	0	−1
1AGR	A	290	1	−1
1AGR	A	291	1	−1
1AGR	A	292	1	−1
1AGR	A	293	1	−1
1AGR	A	294	0	−1
1AGR	A	295	0	−1
1AGR	A	296	0	−1
1AGR	A	297	0	−1
1AGR	A	298	0	−1
1AGR	A	299	0	−1
1AGR	A	300	0	−1
1AGR	A	301	1	−1
1AGR	A	302	0	−1
1AGR	A	303	0	−1
1AGR	A	304	1	−1
1AGR	A	305	0	−1
1AGR	A	306	0	−1
1AGR	A	307	1	−1
1AGR	A	308	1	−1
1AGR	A	309	0	−1

1AGR	A	310	0	−1
1AGR	A	311	1	−1
1AGR	A	312	1	−1
1AGR	A	313	1	−1
1AGR	A	314	1	−1
1AGR	A	315	1	−1
1AGR	A	316	0	−1
1AGR	A	317	1	−1
1AGR	A	318	0	−1
1AGR	A	319	0	−1
1AGR	A	320	0	−1
1AGR	A	321	0	−1
1AGR	A	322	0	−1
1AGR	A	323	0	−1
1AGR	A	324	0	−1
1AGR	A	325	0	−1
1AGR	A	326	0	−1
1AGR	A	327	1	−1
1AGR	A	328	1	−1
1AGR	A	329	1	−1
1AGR	A	330	0	−1
1AGR	A	331	0	−1
1AGR	A	332	1	−1
1AGR	A	333	1	−1
1AGR	A	334	0	−1
1AGR	A	335	0	−1
1AGR	A	336	1	−1
1AGR	A	337	1	−1
1AGR	A	338	0	−1
1AGR	A	339	0	−1
1AGR	A	340	1	−1
1AGR	A	341	0	−1
1AGR	A	342	0	−1
1AGR	A	343	0	−1
1AGR	A	344	1	−1
1AGR	A	345	0	−1
1AGR	A	346	0	−1
1AGR	A	347	0	−1
1AGR	A	348	1	−1
1AGR	A	349	1	−1
1AGR	A	350	1	−1
1AGR	A	351	1	−1
1AGR	A	352	1	−1
1AGR	D	10	1	−1
1AGR	D	11	1	−1
1AGR	D	12	1	−1
1AGR	D	13	1	−1
1AGR	D	14	1	−1
1AGR	D	15	1	−1
1AGR	D	16	1	−1
1AGR	D	17	1	−1
1AGR	D	18	1	−1

1AGR	D	19	1	−1
1AGR	D	20	1	−1
1AGR	D	21	1	−1
1AGR	D	22	1	−1
1AGR	D	23	1	−1
1AGR	D	24	1	−1
1AGR	D	25	1	−1
1AGR	D	26	0	−1
1AGR	D	27	1	−1
1AGR	D	28	1	−1
1AGR	D	29	1	−1
1AGR	D	30	0	−1
1AGR	D	31	1	−1
1AGR	D	32	1	−1
1AGR	D	33	0	−1
1AGR	D	34	0	−1
1AGR	D	35	0	−1
1AGR	D	36	0	−1
1AGR	D	37	0	−1
1AGR	D	38	0	−1
1AGR	D	39	0	−1
1AGR	D	40	0	−1
1AGR	D	41	0	−1
1AGR	D	42	0	−1
1AGR	D	43	0	−1
1AGR	D	44	0	−1
1AGR	D	45	0	−1
1AGR	D	46	0	−1
1AGR	D	47	0	−1
1AGR	D	48	0	−1
1AGR	D	49	0	−1
1AGR	D	50	0	−1
1AGR	D	51	0	−1
1AGR	D	52	0	−1
1AGR	D	53	1	−1
1AGR	D	54	0	−1
1AGR	D	55	0	−1
1AGR	D	56	0	−1
1AGR	D	57	1	−1
1AGR	D	58	1	−1
1AGR	D	59	0	−1
1AGR	D	60	0	−1
1AGR	D	61	1	−1
1AGR	D	62	1	−1
1AGR	D	63	1	−1
1AGR	D	64	1	1
1AGR	D	65	0	−1
1AGR	D	66	1	−1
1AGR	D	67	1	1
1AGR	D	68	1	−1
1AGR	D	69	0	−1
1AGR	D	70	0	−1

1AGR	D	71	0	−1
1AGR	D	72	0	−1
1AGR	D	73	0	−1
1AGR	D	74	1	1
1AGR	D	75	0	−1
1AGR	D	76	0	−1
1AGR	D	77	0	−1
1AGR	D	78	1	−1
1AGR	D	79	0	−1
1AGR	D	80	0	−1
1AGR	D	81	0	−1
1AGR	D	82	0	−1
1AGR	D	83	0	−1
1AGR	D	84	0	−1
1AGR	D	85	1	−1
1AGR	D	86	0	−1
1AGR	D	87	0	−1
1AGR	D	88	1	−1
1AGR	D	89	1	1
1AGR	D	90	0	−1
1AGR	D	91	1	−1
1AGR	D	92	0	−1
1AGR	D	93	1	−1
1AGR	D	94	0	−1
1AGR	D	95	1	−1
1AGR	D	96	1	−1
1AGR	D	97	1	−1
1AGR	D	98	1	−1
1AGR	D	99	0	−1
1AGR	D	100	1	−1
1AGR	D	101	1	−1
1AGR	D	102	0	−1
1AGR	D	103	0	−1
1AGR	D	104	1	−1
1AGR	D	105	1	−1
1AGR	D	106	0	−1
1AGR	D	107	1	−1
1AGR	D	108	1	−1
1AGR	D	109	0	−1
1AGR	D	110	0	−1
1AGR	D	111	1	−1
1AGR	D	112	1	−1
1AGR	D	113	1	−1
1AGR	D	114	1	−1
1AGR	D	115	1	1
1AGR	D	116	1	−1
1AGR	D	117	1	−1
1AGR	D	118	0	−1
1AGR	D	119	1	−1
1AGR	D	120	1	−1
1AGR	D	121	1	−1
1AGR	D	122	0	−1

1AGR	D	123	0	−1
1AGR	D	124	0	−1
1AGR	D	125	0	−1
1AGR	D	126	0	−1
1AGR	D	127	1	−1
1AGR	D	128	1	−1
1AGR	D	129	0	−1
1AGR	D	130	0	−1
1AGR	D	131	1	−1
1AGR	D	132	0	−1
1AGR	D	133	1	−1
1AGR	D	134	0	−1
1AGR	D	135	0	−1
1AGR	D	136	1	−1
1AGR	D	137	1	−1
1AGR	D	138	0	−1
1AGR	D	139	0	−1
1AGR	D	140	1	−1
1AGR	D	141	1	−1
1AGR	D	142	1	−1
1AGR	D	143	1	−1
1AGR	D	144	1	−1
1AGR	D	145	0	−1
1AGR	D	146	1	−1
1AGR	D	147	0	−1
1AGR	D	148	1	−1
1AGR	D	149	1	−1
1AGR	D	150	0	−1
1AGR	D	151	0	−1
1AGR	D	152	0	−1
1AGR	D	153	0	−1
1AGR	D	154	0	−1
1AGR	D	155	0	−1
1AGR	D	156	1	−1
1AGR	D	157	1	−1
1AGR	D	158	0	−1
1AGR	D	159	1	−1
1AGR	D	160	1	−1
1AGR	D	161	0	−1
1AGR	D	162	0	−1
1AGR	D	163	1	−1
1AGR	D	164	1	−1
1AGR	D	165	1	−1
1AGR	D	166	0	−1
1AGR	D	167	1	−1
1AGR	D	168	0	−1
1AGR	D	169	1	−1
1AGR	D	170	1	−1
1AGR	D	171	1	−1
1AGR	D	172	0	−1
1AGR	D	173	0	−1
1AGR	D	174	0	−1

1AGR	D	175	0	−1
1AGR	D	176	0	−1
1AGR	D	177	1	1
1AGR	D	178	1	1
1AGR	D	179	1	1
1AGR	D	180	0	−1
1AGR	D	181	1	1
1AGR	D	182	0	−1
1AGR	D	183	1	1
1AGR	D	184	1	1
1AGR	D	185	1	−1
1AGR	D	186	1	−1
1AGR	D	187	1	−1
1AGR	D	188	0	−1
1AGR	D	189	1	−1
1AGR	D	190	0	−1
1AGR	D	191	1	−1
1AGR	D	192	1	−1
1AGR	D	193	0	−1
1AGR	D	194	1	−1
1AGR	D	195	0	−1
1AGR	D	196	1	−1
1AGR	D	197	0	−1
1AGR	D	198	0	−1
1AGR	D	199	0	−1
1AGR	D	200	0	−1
1AGR	D	201	0	−1
1AGR	D	202	0	−1
1AGR	D	203	0	−1
1AGR	D	204	1	1
1AGR	D	205	1	1
1AGR	D	206	0	−1
1AGR	D	207	0	−1
1AGR	D	208	1	1
1AGR	D	209	1	1
1AGR	D	210	0	−1
1AGR	D	211	1	−1
1AGR	D	212	1	1
1AGR	D	213	0	−1
1AGR	D	214	0	−1
1AGR	D	215	1	−1
1AGR	D	216	1	−1
1AGR	D	217	0	−1
1AGR	D	218	0	−1
1AGR	D	219	0	−1
1AGR	D	220	0	−1
1AGR	D	221	0	−1
1AGR	D	222	0	−1
1AGR	D	223	0	−1
1AGR	D	224	0	−1
1AGR	D	225	0	−1
1AGR	D	226	0	−1

1AGR	D	227	0	−1
1AGR	D	228	0	−1
1AGR	D	229	0	−1
1AGR	D	230	0	−1
1AGR	D	231	0	−1
1AGR	D	232	1	−1
1AGR	D	233	0	−1
1AGR	D	234	1	1
1AGR	D	235	1	1
1AGR	D	236	0	−1
1AGR	D	237	1	1
1AGR	D	238	1	1
1AGR	D	239	0	−1
1AGR	D	240	0	−1
1AGR	D	241	0	−1
1AGR	D	242	0	−1
1AGR	D	243	1	−1
1AGR	D	244	0	−1
1AGR	D	245	0	−1
1AGR	D	246	0	−1
1AGR	D	247	1	−1
1AGR	D	248	0	−1
1AGR	D	249	0	−1
1AGR	D	250	1	−1
1AGR	D	251	1	−1
1AGR	D	252	0	−1
1AGR	D	253	0	−1
1AGR	D	254	0	−1
1AGR	D	255	1	−1
1AGR	D	256	1	−1
1AGR	D	257	1	−1
1AGR	D	258	0	−1
1AGR	D	259	1	−1
1AGR	D	260	1	−1
1AGR	D	261	0	−1
1AGR	D	262	0	−1
1AGR	D	263	0	−1
1AGR	D	264	0	−1
1AGR	D	265	0	−1
1AGR	D	266	0	−1
1AGR	D	267	0	−1
1AGR	D	268	0	−1
1AGR	D	269	0	−1
1AGR	D	270	0	−1
1AGR	D	271	1	−1
1AGR	D	272	0	−1
1AGR	D	273	0	−1
1AGR	D	274	1	−1
1AGR	D	275	1	−1
1AGR	D	276	0	−1
1AGR	D	277	0	−1
1AGR	D	278	1	−1

1AGR	D	279	1	−1
1AGR	D	280	0	−1
1AGR	D	281	1	−1
1AGR	D	282	0	−1
1AGR	D	283	1	−1
1AGR	D	284	1	−1
1AGR	D	285	0	−1
1AGR	D	286	0	−1
1AGR	D	287	1	−1
1AGR	D	288	1	−1
1AGR	D	289	0	−1
1AGR	D	290	1	−1
1AGR	D	291	1	−1
1AGR	D	292	1	−1
1AGR	D	293	1	−1
1AGR	D	294	0	−1
1AGR	D	295	0	−1
1AGR	D	296	0	−1
1AGR	D	297	0	−1
1AGR	D	298	0	−1
1AGR	D	299	0	−1
1AGR	D	300	0	−1
1AGR	D	301	1	−1
1AGR	D	302	0	−1
1AGR	D	303	0	−1
1AGR	D	304	1	−1
1AGR	D	305	0	−1
1AGR	D	306	0	−1
1AGR	D	307	1	−1
1AGR	D	308	1	−1
1AGR	D	309	0	−1
1AGR	D	310	0	−1
1AGR	D	311	1	−1
1AGR	D	312	1	−1
1AGR	D	313	1	−1
1AGR	D	314	1	−1
1AGR	D	315	1	−1
1AGR	D	316	0	−1
1AGR	D	317	1	−1
1AGR	D	318	0	−1
1AGR	D	319	0	−1
1AGR	D	320	0	−1
1AGR	D	321	0	−1
1AGR	D	322	0	−1
1AGR	D	323	0	−1
1AGR	D	324	0	−1
1AGR	D	325	0	−1
1AGR	D	326	0	−1
1AGR	D	327	1	−1
1AGR	D	328	1	−1
1AGR	D	329	1	−1
1AGR	D	330	0	−1

1AGR	D	331	0	−1
1AGR	D	332	1	−1
1AGR	D	333	1	−1
1AGR	D	334	0	−1
1AGR	D	335	0	−1
1AGR	D	336	1	−1
1AGR	D	337	1	−1
1AGR	D	338	0	−1
1AGR	D	339	0	−1
1AGR	D	340	1	−1
1AGR	D	341	0	−1
1AGR	D	342	0	−1
1AGR	D	343	1	−1
1AGR	D	344	1	−1
1AGR	D	345	0	−1
1AGR	D	346	0	−1
1AGR	D	347	1	−1
1AGR	D	348	1	−1
1AGR	D	349	1	−1
1AGR	D	350	1	−1
1AGR	D	351	1	−1
1AGR	D	352	1	−1
1AGR	E	51	1	−1
1AGR	E	52	1	−1
1AGR	E	53	1	−1
1AGR	E	54	1	−1
1AGR	E	55	1	−1
1AGR	E	56	0	−1
1AGR	E	57	1	−1
1AGR	E	58	1	−1
1AGR	E	59	0	−1
1AGR	E	60	1	−1
1AGR	E	61	1	−1
1AGR	E	62	0	−1
1AGR	E	63	0	−1
1AGR	E	64	1	−1
1AGR	E	65	1	−1
1AGR	E	66	0	−1
1AGR	E	67	0	−1
1AGR	E	68	1	−1
1AGR	E	69	0	−1
1AGR	E	70	1	−1
1AGR	E	71	1	−1
1AGR	E	72	0	−1
1AGR	E	73	0	−1
1AGR	E	74	1	−1
1AGR	E	75	0	−1
1AGR	E	76	0	−1
1AGR	E	77	1	−1
1AGR	E	78	1	−1
1AGR	E	79	0	−1
1AGR	E	80	0	−1

1AGR	E	81	1	−1
1AGR	E	82	1	−1
1AGR	E	83	1	1
1AGR	E	84	1	1
1AGR	E	85	0	−1
1AGR	E	86	1	−1
1AGR	E	87	0	−1
1AGR	E	88	0	−1
1AGR	E	89	0	−1
1AGR	E	90	0	−1
1AGR	E	91	0	−1
1AGR	E	92	0	−1
1AGR	E	93	1	−1
1AGR	E	94	0	−1
1AGR	E	95	0	−1
1AGR	E	96	1	−1
1AGR	E	97	1	−1
1AGR	E	98	0	−1
1AGR	E	99	1	−1
1AGR	E	100	1	−1
1AGR	E	101	0	−1
1AGR	E	102	1	−1
1AGR	E	103	1	−1
1AGR	E	104	1	−1
1AGR	E	105	1	−1
1AGR	E	106	1	−1
1AGR	E	107	0	−1
1AGR	E	108	1	−1
1AGR	E	109	1	−1
1AGR	E	110	0	−1
1AGR	E	111	0	−1
1AGR	E	112	1	−1
1AGR	E	113	1	−1
1AGR	E	114	0	−1
1AGR	E	115	0	−1
1AGR	E	116	1	−1
1AGR	E	117	1	−1
1AGR	E	118	0	−1
1AGR	E	119	0	−1
1AGR	E	120	0	−1
1AGR	E	121	1	−1
1AGR	E	122	1	−1
1AGR	E	123	0	−1
1AGR	E	124	1	1
1AGR	E	125	1	1
1AGR	E	126	1	1
1AGR	E	127	0	−1
1AGR	E	128	1	1
1AGR	E	129	0	−1
1AGR	E	130	1	1
1AGR	E	131	1	1
1AGR	E	132	1	−1

1AGR	E	133	0	−1
1AGR	E	134	0	−1
1AGR	E	135	1	1
1AGR	E	136	1	−1
1AGR	E	137	0	−1
1AGR	E	138	1	−1
1AGR	E	139	1	−1
1AGR	E	140	1	−1
1AGR	E	141	0	−1
1AGR	E	142	1	−1
1AGR	E	143	1	−1
1AGR	E	144	0	−1
1AGR	E	145	1	−1
1AGR	E	146	1	−1
1AGR	E	147	1	−1
1AGR	E	148	0	−1
1AGR	E	149	0	−1
1AGR	E	150	1	−1
1AGR	E	151	1	−1
1AGR	E	152	0	−1
1AGR	E	153	0	−1
1AGR	E	154	1	−1
1AGR	E	155	1	−1
1AGR	E	156	0	−1
1AGR	E	157	0	−1
1AGR	E	158	1	−1
1AGR	E	159	1	1
1AGR	E	160	0	−1
1AGR	E	161	0	−1
1AGR	E	162	1	1
1AGR	E	163	1	1
1AGR	E	164	0	−1
1AGR	E	165	0	−1
1AGR	E	166	1	1
1AGR	E	167	1	1
1AGR	E	168	0	−1
1AGR	E	169	0	−1
1AGR	E	170	1	1
1AGR	E	171	0	−1
1AGR	E	172	1	−1
1AGR	E	173	1	−1
1AGR	E	174	0	−1
1AGR	E	175	1	−1
1AGR	E	176	1	−1
1AGR	E	177	1	−1
1AGR	E	178	1	−1
1AGR	H	60	1	−1
1AGR	H	61	1	−1
1AGR	H	62	0	−1
1AGR	H	63	0	−1
1AGR	H	64	1	−1
1AGR	H	65	1	−1

1AGR	H	66	0	−1
1AGR	H	67	0	−1
1AGR	H	68	1	−1
1AGR	H	69	1	−1
1AGR	H	70	1	−1
1AGR	H	71	1	−1
1AGR	H	72	0	−1
1AGR	H	73	0	−1
1AGR	H	74	1	−1
1AGR	H	75	1	−1
1AGR	H	76	0	−1
1AGR	H	77	1	−1
1AGR	H	78	1	−1
1AGR	H	79	0	−1
1AGR	H	80	0	−1
1AGR	H	81	1	−1
1AGR	H	82	1	1
1AGR	H	83	1	1
1AGR	H	84	1	1
1AGR	H	85	0	−1
1AGR	H	86	0	−1
1AGR	H	87	0	−1
1AGR	H	88	0	−1
1AGR	H	89	0	−1
1AGR	H	90	0	−1
1AGR	H	91	0	−1
1AGR	H	92	0	−1
1AGR	H	93	0	−1
1AGR	H	94	0	−1
1AGR	H	95	0	−1
1AGR	H	96	1	−1
1AGR	H	97	1	−1
1AGR	H	98	0	−1
1AGR	H	99	1	−1
1AGR	H	100	1	−1
1AGR	H	101	0	−1
1AGR	H	102	1	−1
1AGR	H	103	1	−1
1AGR	H	104	1	−1
1AGR	H	105	1	−1
1AGR	H	106	1	−1
1AGR	H	107	0	−1
1AGR	H	108	1	−1
1AGR	H	109	1	−1
1AGR	H	110	0	−1
1AGR	H	111	0	−1
1AGR	H	112	1	−1
1AGR	H	113	1	−1
1AGR	H	114	0	−1
1AGR	H	115	0	−1
1AGR	H	116	1	−1
1AGR	H	117	1	−1

1AGR	H	118	0	−1
1AGR	H	119	0	−1
1AGR	H	120	0	−1
1AGR	H	121	1	−1
1AGR	H	122	1	−1
1AGR	H	123	0	−1
1AGR	H	124	1	1
1AGR	H	125	1	1
1AGR	H	126	1	1
1AGR	H	127	0	−1
1AGR	H	128	1	1
1AGR	H	129	0	−1
1AGR	H	130	1	1
1AGR	H	131	1	1
1AGR	H	132	1	1
1AGR	H	133	0	−1
1AGR	H	134	0	−1
1AGR	H	135	1	1
1AGR	H	136	1	−1
1AGR	H	137	0	−1
1AGR	H	138	1	−1
1AGR	H	139	1	−1
1AGR	H	140	1	−1
1AGR	H	141	0	−1
1AGR	H	142	1	−1
1AGR	H	143	1	−1
1AGR	H	144	0	−1
1AGR	H	145	1	−1
1AGR	H	146	1	−1
1AGR	H	147	1	−1
1AGR	H	148	0	−1
1AGR	H	149	0	−1
1AGR	H	150	1	−1
1AGR	H	151	0	−1
1AGR	H	152	0	−1
1AGR	H	153	0	−1
1AGR	H	154	1	−1
1AGR	H	155	1	−1
1AGR	H	156	0	−1
1AGR	H	157	0	−1
1AGR	H	158	1	−1
1AGR	H	159	1	1
1AGR	H	160	0	−1
1AGR	H	161	0	−1
1AGR	H	162	1	1
1AGR	H	163	1	1
1AGR	H	164	0	−1
1AGR	H	165	0	−1
1AGR	H	166	1	1
1AGR	H	167	1	1
1AGR	H	168	0	−1
1AGR	H	169	0	−1

1AGR	H	170	1	1
1AGR	H	171	0	−1
1AGR	H	172	1	−1
1AGR	H	173	1	−1
1AGR	H	174	1	−1
1AGR	H	175	1	−1
1AK4	A	1	1	−1
1AK4	A	2	1	−1
1AK4	A	3	0	−1
1AK4	A	4	0	−1
1AK4	A	5	0	−1
1AK4	A	6	0	−1
1AK4	A	7	0	−1
1AK4	A	8	0	−1
1AK4	A	9	0	−1
1AK4	A	10	0	−1
1AK4	A	11	0	−1
1AK4	A	12	0	−1
1AK4	A	13	1	−1
1AK4	A	14	1	−1
1AK4	A	15	1	−1
1AK4	A	16	1	−1
1AK4	A	17	1	−1
1AK4	A	18	1	−1
1AK4	A	19	1	−1
1AK4	A	20	0	−1
1AK4	A	21	0	−1
1AK4	A	22	0	−1
1AK4	A	23	0	−1
1AK4	A	24	0	−1
1AK4	A	25	0	−1
1AK4	A	26	0	−1
1AK4	A	27	1	−1
1AK4	A	28	1	−1
1AK4	A	29	0	−1
1AK4	A	30	1	−1
1AK4	A	31	1	−1
1AK4	A	32	0	−1
1AK4	A	33	0	−1
1AK4	A	34	1	−1
1AK4	A	35	0	−1
1AK4	A	36	0	−1
1AK4	A	37	0	−1
1AK4	A	38	0	−1
1AK4	A	39	0	−1
1AK4	A	40	0	−1
1AK4	A	41	1	−1
1AK4	A	42	1	−1
1AK4	A	43	1	−1
1AK4	A	44	1	−1
1AK4	A	45	1	−1
1AK4	A	46	1	−1

1AK4	A	47	0	−1
1AK4	A	48	0	−1
1AK4	A	49	1	−1
1AK4	A	50	1	−1
1AK4	A	51	0	−1
1AK4	A	52	0	−1
1AK4	A	53	0	−1
1AK4	A	54	0	−1
1AK4	A	55	1	1
1AK4	A	56	0	−1
1AK4	A	57	0	−1
1AK4	A	58	1	−1
1AK4	A	59	1	−1
1AK4	A	60	0	−1
1AK4	A	61	0	−1
1AK4	A	62	0	−1
1AK4	A	63	0	−1
1AK4	A	64	0	−1
1AK4	A	65	0	−1
1AK4	A	66	0	−1
1AK4	A	67	0	−1
1AK4	A	68	1	−1
1AK4	A	69	1	1
1AK4	A	70	1	−1
1AK4	A	71	1	1
1AK4	A	72	1	1
1AK4	A	73	1	1
1AK4	A	74	0	−1
1AK4	A	75	0	−1
1AK4	A	76	1	−1
1AK4	A	77	0	−1
1AK4	A	78	0	−1
1AK4	A	79	1	−1
1AK4	A	80	1	−1
1AK4	A	81	1	−1
1AK4	A	82	1	−1
1AK4	A	83	0	−1
1AK4	A	84	1	−1
1AK4	A	85	0	−1
1AK4	A	86	1	−1
1AK4	A	87	0	−1
1AK4	A	88	1	−1
1AK4	A	89	1	−1
1AK4	A	90	1	−1
1AK4	A	91	1	−1
1AK4	A	92	0	−1
1AK4	A	93	1	−1
1AK4	A	94	0	−1
1AK4	A	95	1	−1
1AK4	A	96	0	−1
1AK4	A	97	0	−1
1AK4	A	98	0	−1

1AK4	A	99	0	−1
1AK4	A	100	0	−1
1AK4	A	101	0	−1
1AK4	A	102	0	−1
1AK4	A	103	1	1
1AK4	A	104	1	−1
1AK4	A	105	1	−1
1AK4	A	106	1	−1
1AK4	A	107	1	−1
1AK4	A	108	0	−1
1AK4	A	109	0	−1
1AK4	A	110	0	−1
1AK4	A	111	0	−1
1AK4	A	112	0	−1
1AK4	A	113	0	−1
1AK4	A	114	0	−1
1AK4	A	115	0	−1
1AK4	A	116	0	−1
1AK4	A	117	0	−1
1AK4	A	118	1	−1
1AK4	A	119	0	−1
1AK4	A	120	1	−1
1AK4	A	121	1	1
1AK4	A	122	0	−1
1AK4	A	123	1	−1
1AK4	A	124	1	−1
1AK4	A	125	1	−1
1AK4	A	126	0	−1
1AK4	A	127	0	−1
1AK4	A	128	0	−1
1AK4	A	129	0	−1
1AK4	A	130	0	−1
1AK4	A	131	1	−1
1AK4	A	132	0	−1
1AK4	A	133	1	−1
1AK4	A	134	1	−1
1AK4	A	135	0	−1
1AK4	A	136	0	−1
1AK4	A	137	1	−1
1AK4	A	138	0	−1
1AK4	A	139	0	−1
1AK4	A	140	1	−1
1AK4	A	141	0	−1
1AK4	A	142	0	−1
1AK4	A	143	0	−1
1AK4	A	144	1	−1
1AK4	A	145	0	−1
1AK4	A	146	0	−1
1AK4	A	147	1	−1
1AK4	A	148	1	1
1AK4	A	149	1	1
1AK4	A	150	0	−1

1AK4	A	151	1	−1
1AK4	A	152	0	−1
1AK4	A	153	1	−1
1AK4	A	154	1	−1
1AK4	A	155	1	−1
1AK4	A	156	0	−1
1AK4	A	157	0	−1
1AK4	A	158	0	−1
1AK4	A	159	1	−1
1AK4	A	160	1	−1
1AK4	A	161	0	−1
1AK4	A	162	0	−1
1AK4	A	163	1	−1
1AK4	A	164	1	−1
1AK4	A	165	1	−1
1AK4	B	3	1	−1
1AK4	B	4	0	−1
1AK4	B	5	0	−1
1AK4	B	6	0	−1
1AK4	B	7	0	−1
1AK4	B	8	0	−1
1AK4	B	9	0	−1
1AK4	B	10	0	−1
1AK4	B	11	0	−1
1AK4	B	12	0	−1
1AK4	B	13	1	−1
1AK4	B	14	1	−1
1AK4	B	15	1	−1
1AK4	B	16	1	−1
1AK4	B	17	1	−1
1AK4	B	18	1	−1
1AK4	B	19	1	−1
1AK4	B	20	0	−1
1AK4	B	21	0	−1
1AK4	B	22	0	−1
1AK4	B	23	1	−1
1AK4	B	24	0	−1
1AK4	B	25	0	−1
1AK4	B	26	1	−1
1AK4	B	27	1	−1
1AK4	B	28	1	−1
1AK4	B	29	0	−1
1AK4	B	30	1	−1
1AK4	B	31	1	−1
1AK4	B	32	0	−1
1AK4	B	33	0	−1
1AK4	B	34	1	−1
1AK4	B	35	0	−1
1AK4	B	36	0	−1
1AK4	B	37	0	−1
1AK4	B	38	0	−1
1AK4	B	39	0	−1

1AK4	B	40	0	−1
1AK4	B	41	1	−1
1AK4	B	42	1	−1
1AK4	B	43	1	−1
1AK4	B	44	1	−1
1AK4	B	45	1	−1
1AK4	B	46	1	−1
1AK4	B	47	0	−1
1AK4	B	48	0	−1
1AK4	B	49	1	−1
1AK4	B	50	1	−1
1AK4	B	51	0	−1
1AK4	B	52	0	−1
1AK4	B	53	0	−1
1AK4	B	54	0	−1
1AK4	B	55	1	1
1AK4	B	56	0	−1
1AK4	B	57	0	−1
1AK4	B	58	1	−1
1AK4	B	59	1	−1
1AK4	B	60	0	−1
1AK4	B	61	0	−1
1AK4	B	62	0	−1
1AK4	B	63	0	−1
1AK4	B	64	0	−1
1AK4	B	65	0	−1
1AK4	B	66	0	−1
1AK4	B	67	0	−1
1AK4	B	68	1	−1
1AK4	B	69	1	1
1AK4	B	70	1	−1
1AK4	B	71	1	1
1AK4	B	72	1	1
1AK4	B	73	1	1
1AK4	B	74	0	−1
1AK4	B	75	0	−1
1AK4	B	76	1	−1
1AK4	B	77	0	−1
1AK4	B	78	0	−1
1AK4	B	79	1	−1
1AK4	B	80	1	−1
1AK4	B	81	1	−1
1AK4	B	82	1	−1
1AK4	B	83	0	−1
1AK4	B	84	1	−1
1AK4	B	85	0	−1
1AK4	B	86	1	−1
1AK4	B	87	0	−1
1AK4	B	88	1	−1
1AK4	B	89	1	−1
1AK4	B	90	1	−1
1AK4	B	91	1	−1

1AK4	B	92	0	−1
1AK4	B	93	1	−1
1AK4	B	94	0	−1
1AK4	B	95	1	−1
1AK4	B	96	0	−1
1AK4	B	97	0	−1
1AK4	B	98	0	−1
1AK4	B	99	0	−1
1AK4	B	100	0	−1
1AK4	B	101	0	−1
1AK4	B	102	0	−1
1AK4	B	103	1	1
1AK4	B	104	1	−1
1AK4	B	105	1	−1
1AK4	B	106	1	−1
1AK4	B	107	0	−1
1AK4	B	108	0	−1
1AK4	B	109	0	−1
1AK4	B	110	0	−1
1AK4	B	111	0	−1
1AK4	B	112	0	−1
1AK4	B	113	0	−1
1AK4	B	114	0	−1
1AK4	B	115	0	−1
1AK4	B	116	0	−1
1AK4	B	117	1	−1
1AK4	B	118	1	−1
1AK4	B	119	0	−1
1AK4	B	120	1	−1
1AK4	B	121	1	1
1AK4	B	122	0	−1
1AK4	B	123	1	−1
1AK4	B	124	1	−1
1AK4	B	125	1	1
1AK4	B	126	0	−1
1AK4	B	127	0	−1
1AK4	B	128	0	−1
1AK4	B	129	0	−1
1AK4	B	130	0	−1
1AK4	B	131	1	−1
1AK4	B	132	0	−1
1AK4	B	133	1	−1
1AK4	B	134	1	−1
1AK4	B	135	0	−1
1AK4	B	136	0	−1
1AK4	B	137	1	−1
1AK4	B	138	0	−1
1AK4	B	139	0	−1
1AK4	B	140	1	−1
1AK4	B	141	0	−1
1AK4	B	142	0	−1
1AK4	B	143	0	−1

1AK4	B	144	1	−1
1AK4	B	145	0	−1
1AK4	B	146	0	−1
1AK4	B	147	1	−1
1AK4	B	148	1	1
1AK4	B	149	1	1
1AK4	B	150	0	−1
1AK4	B	151	1	1
1AK4	B	152	0	−1
1AK4	B	153	1	−1
1AK4	B	154	1	−1
1AK4	B	155	1	−1
1AK4	B	156	0	−1
1AK4	B	157	0	−1
1AK4	B	158	0	−1
1AK4	B	159	1	−1
1AK4	B	160	1	−1
1AK4	B	161	0	−1
1AK4	B	162	0	−1
1AK4	B	163	1	−1
1AK4	B	164	1	−1
1AK4	B	165	1	−1
1AO7	A	1	1	−1
1AO7	A	2	1	−1
1AO7	A	3	0	−1
1AO7	A	4	0	−1
1AO7	A	5	0	−1
1AO7	A	6	0	−1
1AO7	A	7	0	−1
1AO7	A	8	1	1
1AO7	A	9	0	−1
1AO7	A	10	1	1
1AO7	A	11	0	−1
1AO7	A	12	1	1
1AO7	A	13	0	−1
1AO7	A	14	1	−1
1AO7	A	15	1	−1
1AO7	A	16	1	−1
1AO7	A	17	1	−1
1AO7	A	18	1	−1
1AO7	A	19	1	−1
1AO7	A	20	0	−1
1AO7	A	21	1	1
1AO7	A	22	0	−1
1AO7	A	23	0	−1
1AO7	A	24	0	−1
1AO7	A	25	0	−1
1AO7	A	26	0	−1
1AO7	A	27	1	1
1AO7	A	28	0	−1
1AO7	A	29	0	−1
1AO7	A	30	1	−1

1AO7	A	31	0	−1
1AO7	A	32	1	1
1AO7	A	33	0	−1
1AO7	A	34	0	−1
1AO7	A	35	0	−1
1AO7	A	36	0	−1
1AO7	A	37	0	−1
1AO7	A	38	0	−1
1AO7	A	39	1	−1
1AO7	A	40	0	−1
1AO7	A	41	1	−1
1AO7	A	42	1	−1
1AO7	A	43	1	−1
1AO7	A	44	1	−1
1AO7	A	45	0	−1
1AO7	A	46	0	−1
1AO7	A	47	1	−1
1AO7	A	48	1	1
1AO7	A	49	0	−1
1AO7	A	50	1	−1
1AO7	A	51	0	−1
1AO7	A	52	0	−1
1AO7	A	53	1	−1
1AO7	A	54	1	−1
1AO7	A	55	0	−1
1AO7	A	56	1	−1
1AO7	A	57	1	−1
1AO7	A	58	1	1
1AO7	A	59	0	−1
1AO7	A	60	0	−1
1AO7	A	61	1	−1
1AO7	A	62	0	−1
1AO7	A	63	0	−1
1AO7	A	64	0	−1
1AO7	A	65	1	1
1AO7	A	66	1	1
1AO7	A	67	0	−1
1AO7	A	68	1	1
1AO7	A	69	1	1
1AO7	A	70	0	−1
1AO7	A	71	0	−1
1AO7	A	72	1	1
1AO7	A	73	1	1
1AO7	A	74	0	−1
1AO7	A	75	1	−1
1AO7	A	76	1	1
1AO7	A	77	0	−1
1AO7	A	78	0	−1
1AO7	A	79	1	−1
1AO7	A	80	1	1
1AO7	A	81	0	−1
1AO7	A	82	1	−1

1AO7	A	83	1	−1
1AO7	A	84	1	1
1AO7	A	85	1	−1
1AO7	A	86	1	−1
1AO7	A	87	1	−1
1AO7	A	88	1	−1
1AO7	A	89	1	−1
1AO7	A	90	1	−1
1AO7	A	91	1	−1
1AO7	A	92	1	1
1AO7	A	93	0	−1
1AO7	A	94	1	1
1AO7	A	95	0	−1
1AO7	A	96	1	1
1AO7	A	97	0	−1
1AO7	A	98	0	−1
1AO7	A	99	0	−1
1AO7	A	100	0	−1
1AO7	A	101	0	−1
1AO7	A	102	0	−1
1AO7	A	103	0	−1
1AO7	A	104	0	−1
1AO7	A	105	1	−1
1AO7	A	106	1	−1
1AO7	A	107	0	−1
1AO7	A	108	1	−1
1AO7	A	109	1	−1
1AO7	A	110	1	−1
1AO7	A	111	1	−1
1AO7	A	112	0	−1
1AO7	A	113	1	−1
1AO7	A	114	0	−1
1AO7	A	115	1	1
1AO7	A	116	0	−1
1AO7	A	117	0	−1
1AO7	A	118	0	−1
1AO7	A	119	1	1
1AO7	A	120	1	1
1AO7	A	121	1	1
1AO7	A	122	1	1
1AO7	A	123	0	−1
1AO7	A	124	0	−1
1AO7	A	125	0	−1
1AO7	A	126	0	−1
1AO7	A	127	1	−1
1AO7	A	128	1	−1
1AO7	A	129	1	−1
1AO7	A	130	0	−1
1AO7	A	131	1	−1
1AO7	A	132	1	−1
1AO7	A	133	0	−1
1AO7	A	134	1	−1

1AO7	A	135	0	−1
1AO7	A	136	1	−1
1AO7	A	137	1	−1
1AO7	A	138	1	−1
1AO7	A	139	0	−1
1AO7	A	140	0	−1
1AO7	A	141	1	−1
1AO7	A	142	1	−1
1AO7	A	143	0	−1
1AO7	A	144	0	−1
1AO7	A	145	1	−1
1AO7	A	146	1	1
1AO7	A	147	0	−1
1AO7	A	148	1	−1
1AO7	A	149	1	1
1AO7	A	150	1	1
1AO7	A	151	1	1
1AO7	A	152	1	1
1AO7	A	153	0	−1
1AO7	A	154	1	1
1AO7	A	155	1	1
1AO7	A	156	0	−1
1AO7	A	157	0	−1
1AO7	A	158	1	1
1AO7	A	159	1	1
1AO7	A	160	0	−1
1AO7	A	161	1	−1
1AO7	A	162	0	−1
1AO7	A	163	1	1
1AO7	A	164	0	−1
1AO7	A	165	0	−1
1AO7	A	166	1	1
1AO7	A	167	1	1
1AO7	A	168	0	−1
1AO7	A	169	1	−1
1AO7	A	170	1	1
1AO7	A	171	0	−1
1AO7	A	172	0	−1
1AO7	A	173	1	−1
1AO7	A	174	1	−1
1AO7	A	175	0	−1
1AO7	A	176	1	−1
1AO7	A	177	1	−1
1AO7	A	178	1	−1
1AO7	A	179	0	−1
1AO7	A	180	1	−1
1AO7	A	181	1	−1
1AO7	A	182	1	−1
1AO7	A	183	1	−1
1AO7	A	184	1	−1
1AO7	A	185	0	−1
1AO7	A	186	1	−1

1AO7	A	187	0	−1
1AO7	A	188	1	−1
1AO7	A	189	0	−1
1AO7	A	190	1	−1
1AO7	A	191	1	−1
1AO7	A	192	1	1
1AO7	A	193	1	−1
1AO7	A	194	1	−1
1AO7	A	195	1	−1
1AO7	A	196	1	−1
1AO7	A	197	1	−1
1AO7	A	198	1	−1
1AO7	A	199	0	−1
1AO7	A	200	0	−1
1AO7	A	201	0	−1
1AO7	A	202	1	1
1AO7	A	203	0	−1
1AO7	A	204	0	−1
1AO7	A	205	0	−1
1AO7	A	206	1	1
1AO7	A	207	1	1
1AO7	A	208	0	−1
1AO7	A	209	0	−1
1AO7	A	210	0	−1
1AO7	A	211	0	−1
1AO7	A	212	1	−1
1AO7	A	213	0	−1
1AO7	A	214	1	−1
1AO7	A	215	0	−1
1AO7	A	216	1	−1
1AO7	A	217	0	−1
1AO7	A	218	1	−1
1AO7	A	219	0	−1
1AO7	A	220	1	−1
1AO7	A	221	1	−1
1AO7	A	222	1	−1
1AO7	A	223	1	−1
1AO7	A	224	0	−1
1AO7	A	225	1	−1
1AO7	A	226	1	−1
1AO7	A	227	1	−1
1AO7	A	228	0	−1
1AO7	A	229	1	−1
1AO7	A	230	1	−1
1AO7	A	231	1	1
1AO7	A	232	1	1
1AO7	A	233	1	1
1AO7	A	234	1	1
1AO7	A	235	1	1
1AO7	A	236	1	1
1AO7	A	237	1	1
1AO7	A	238	1	1

1AO7	A	239	1	−1
1AO7	A	240	0	−1
1AO7	A	241	0	−1
1AO7	A	242	0	−1
1AO7	A	243	0	−1
1AO7	A	244	0	−1
1AO7	A	245	0	−1
1AO7	A	246	0	−1
1AO7	A	247	0	−1
1AO7	A	248	1	−1
1AO7	A	249	0	−1
1AO7	A	250	1	−1
1AO7	A	251	1	−1
1AO7	A	252	1	−1
1AO7	A	253	1	−1
1AO7	A	254	0	−1
1AO7	A	255	1	−1
1AO7	A	256	1	−1
1AO7	A	257	0	−1
1AO7	A	258	0	−1
1AO7	A	259	0	−1
1AO7	A	260	0	−1
1AO7	A	261	0	−1
1AO7	A	262	1	−1
1AO7	A	263	0	−1
1AO7	A	264	1	−1
1AO7	A	265	1	−1
1AO7	A	266	0	−1
1AO7	A	267	1	−1
1AO7	A	268	1	−1
1AO7	A	269	1	−1
1AO7	A	270	1	−1
1AO7	A	271	1	−1
1AO7	A	272	0	−1
1AO7	A	273	1	−1
1AO7	A	274	1	−1
1AO7	B	1	1	1
1AO7	B	2	1	1
1AO7	B	3	1	−1
1AO7	B	4	1	1
1AO7	B	5	1	−1
1AO7	B	6	0	−1
1AO7	B	7	1	1
1AO7	B	8	0	−1
1AO7	B	9	1	1
1AO7	B	10	0	−1
1AO7	B	11	1	1
1AO7	B	12	0	−1
1AO7	B	13	1	1
1AO7	B	14	1	1
1AO7	B	15	1	1
1AO7	B	16	0	−1

1AO7	B	17	1	−1
1AO7	B	18	1	−1
1AO7	B	19	1	−1
1AO7	B	20	1	−1
1AO7	B	21	1	−1
1AO7	B	22	0	−1
1AO7	B	23	1	−1
1AO7	B	24	0	−1
1AO7	B	25	0	−1
1AO7	B	26	0	−1
1AO7	B	27	1	1
1AO7	B	28	0	−1
1AO7	B	29	0	−1
1AO7	B	30	1	1
1AO7	B	31	0	−1
1AO7	B	32	1	1
1AO7	B	33	1	1
1AO7	B	34	1	1
1AO7	B	35	1	1
1AO7	B	36	0	−1
1AO7	B	37	1	−1
1AO7	B	38	0	−1
1AO7	B	39	0	−1
1AO7	B	40	0	−1
1AO7	B	41	0	−1
1AO7	B	42	1	−1
1AO7	B	43	1	−1
1AO7	B	44	1	−1
1AO7	B	45	1	−1
1AO7	B	46	1	−1
1AO7	B	47	0	−1
1AO7	B	48	1	−1
1AO7	B	49	1	−1
1AO7	B	50	0	−1
1AO7	B	51	1	−1
1AO7	B	52	1	1
1AO7	B	53	1	1
1AO7	B	54	1	1
1AO7	B	55	1	1
1AO7	B	56	1	1
1AO7	B	57	1	1
1AO7	B	58	1	1
1AO7	B	59	1	−1
1AO7	B	60	1	−1
1AO7	B	61	1	1
1AO7	B	62	0	−1
1AO7	B	63	0	−1
1AO7	B	64	1	1
1AO7	B	65	0	−1
1AO7	B	66	1	1
1AO7	B	67	0	−1
1AO7	B	68	0	−1

1AO7	B	69	0	−1
1AO7	B	70	1	−1
1AO7	B	71	0	−1
1AO7	B	72	1	−1
1AO7	B	73	0	−1
1AO7	B	74	1	−1
1AO7	B	75	1	−1
1AO7	B	76	1	−1
1AO7	B	77	0	−1
1AO7	B	78	1	−1
1AO7	B	79	0	−1
1AO7	B	80	0	−1
1AO7	B	81	0	−1
1AO7	B	82	1	−1
1AO7	B	83	0	−1
1AO7	B	84	1	−1
1AO7	B	85	0	−1
1AO7	B	86	1	−1
1AO7	B	87	1	−1
1AO7	B	88	0	−1
1AO7	B	89	1	−1
1AO7	B	90	1	−1
1AO7	B	91	1	−1
1AO7	B	92	1	−1
1AO7	B	93	1	−1
1AO7	B	94	1	−1
1AO7	B	95	1	−1
1AO7	B	96	0	−1
1AO7	B	97	1	−1
1AO7	B	98	1	−1
1AO7	B	99	1	1
1AO7	B	100	1	1
1AO7	D	1	1	1
1AO7	D	2	1	−1
1AO7	D	3	0	−1
1AO7	D	4	1	−1
1AO7	D	5	0	−1
1AO7	D	6	1	−1
1AO7	D	7	1	−1
1AO7	D	8	1	−1
1AO7	D	9	1	−1
1AO7	D	10	1	−1
1AO7	D	11	1	−1
1AO7	D	12	0	−1
1AO7	D	13	1	−1
1AO7	D	14	1	−1
1AO7	D	15	1	−1
1AO7	D	16	1	−1
1AO7	D	17	1	−1
1AO7	D	18	0	−1
1AO7	D	19	1	−1
1AO7	D	20	0	−1

1AO7	D	21	1	−1
1AO7	D	22	0	−1
1AO7	D	23	1	−1
1AO7	D	24	0	−1
1AO7	D	25	1	1
1AO7	D	26	1	1
1AO7	D	27	1	1
1AO7	D	28	1	1
1AO7	D	29	0	−1
1AO7	D	30	1	1
1AO7	D	31	0	−1
1AO7	D	32	0	−1
1AO7	D	33	0	−1
1AO7	D	34	0	−1
1AO7	D	35	0	−1
1AO7	D	36	0	−1
1AO7	D	37	1	1
1AO7	D	38	1	−1
1AO7	D	39	1	1
1AO7	D	40	1	1
1AO7	D	41	1	1
1AO7	D	42	1	1
1AO7	D	43	1	1
1AO7	D	44	1	−1
1AO7	D	45	1	1
1AO7	D	46	0	−1
1AO7	D	47	0	−1
1AO7	D	48	0	−1
1AO7	D	49	0	−1
1AO7	D	50	1	1
1AO7	D	51	1	1
1AO7	D	52	1	1
1AO7	D	53	1	−1
1AO7	D	54	1	−1
1AO7	D	55	1	−1
1AO7	D	56	1	−1
1AO7	D	57	1	−1
1AO7	D	58	1	−1
1AO7	D	59	1	−1
1AO7	D	60	0	−1
1AO7	D	61	0	−1
1AO7	D	62	0	−1
1AO7	D	63	1	−1
1AO7	D	64	0	−1
1AO7	D	65	1	−1
1AO7	D	66	1	1
1AO7	D	67	1	−1
1AO7	D	68	1	−1
1AO7	D	69	0	−1
1AO7	D	70	1	−1
1AO7	D	71	0	−1
1AO7	D	72	0	−1

1AO7	D	73	0	−1
1AO7	D	74	1	−1
1AO7	D	75	0	−1
1AO7	D	76	1	−1
1AO7	D	77	1	−1
1AO7	D	78	0	−1
1AO7	D	79	1	−1
1AO7	D	80	1	−1
1AO7	D	81	1	−1
1AO7	D	82	0	−1
1AO7	D	83	1	−1
1AO7	D	84	0	−1
1AO7	D	85	0	−1
1AO7	D	86	0	−1
1AO7	D	87	0	−1
1AO7	D	88	0	−1
1AO7	D	89	0	−1
1AO7	D	90	0	−1
1AO7	D	91	0	−1
1AO7	D	92	0	−1
1AO7	D	93	1	1
1AO7	D	94	1	1
1AO7	D	95	1	1
1AO7	D	96	1	1
1AO7	D	97	1	1
1AO7	D	98	1	1
1AO7	D	99	0	−1
1AO7	D	100	1	1
1AO7	D	101	0	−1
1AO7	D	102	1	1
1AO7	D	103	0	−1
1AO7	D	104	0	−1
1AO7	D	105	1	−1
1AO7	D	106	0	−1
1AO7	D	107	1	−1
1AO7	D	108	0	−1
1AO7	D	109	1	−1
1AO7	D	110	1	−1
1AO7	D	111	1	−1
1AO7	D	112	1	−1
1AO7	D	113	1	−1
1AO7	D	114	1	−1
1AO7	D	115	1	−1
1AO7	E	3	1	−1
1AO7	E	4	0	−1
1AO7	E	5	1	−1
1AO7	E	6	0	−1
1AO7	E	7	1	−1
1AO7	E	8	0	−1
1AO7	E	9	1	−1
1AO7	E	10	0	−1
1AO7	E	11	1	−1

1AO7	E	12	0	−1
1AO7	E	13	0	−1
1AO7	E	14	1	−1
1AO7	E	15	1	−1
1AO7	E	16	1	−1
1AO7	E	17	1	−1
1AO7	E	18	1	−1
1AO7	E	19	0	−1
1AO7	E	20	1	−1
1AO7	E	21	0	−1
1AO7	E	22	1	−1
1AO7	E	23	0	−1
1AO7	E	24	0	−1
1AO7	E	25	0	−1
1AO7	E	26	1	−1
1AO7	E	27	1	−1
1AO7	E	28	1	−1
1AO7	E	29	0	−1
1AO7	E	30	1	1
1AO7	E	31	0	−1
1AO7	E	32	0	−1
1AO7	E	33	0	−1
1AO7	E	34	0	−1
1AO7	E	35	0	−1
1AO7	E	36	0	−1
1AO7	E	37	1	1
1AO7	E	38	0	−1
1AO7	E	39	1	−1
1AO7	E	40	1	−1
1AO7	E	41	1	1
1AO7	E	42	1	1
1AO7	E	43	1	1
1AO7	E	44	0	−1
1AO7	E	45	0	−1
1AO7	E	46	0	−1
1AO7	E	47	0	−1
1AO7	E	48	1	1
1AO7	E	49	0	−1
1AO7	E	50	1	1
1AO7	E	51	0	−1
1AO7	E	52	1	−1
1AO7	E	53	1	−1
1AO7	E	54	1	1
1AO7	E	55	1	−1
1AO7	E	56	1	−1
1AO7	E	57	1	1
1AO7	E	58	1	1
1AO7	E	59	1	1
1AO7	E	60	0	−1
1AO7	E	61	0	−1
1AO7	E	62	1	−1
1AO7	E	63	1	−1

1AO7	E	64	0	−1
1AO7	E	65	1	−1
1AO7	E	66	0	−1
1AO7	E	67	1	−1
1AO7	E	68	0	−1
1AO7	E	69	1	−1
1AO7	E	70	1	−1
1AO7	E	71	0	−1
1AO7	E	72	1	−1
1AO7	E	73	1	−1
1AO7	E	74	0	−1
1AO7	E	75	0	−1
1AO7	E	76	0	−1
1AO7	E	77	1	−1
1AO7	E	78	0	−1
1AO7	E	79	1	−1
1AO7	E	80	1	−1
1AO7	E	81	0	−1
1AO7	E	82	1	−1
1AO7	E	83	1	−1
1AO7	E	84	1	−1
1AO7	E	85	0	−1
1AO7	E	86	1	−1
1AO7	E	87	0	−1
1AO7	E	88	0	−1
1AO7	E	89	0	−1
1AO7	E	90	0	−1
1AO7	E	91	0	−1
1AO7	E	92	0	−1
1AO7	E	93	0	−1
1AO7	E	94	1	1
1AO7	E	95	0	−1
1AO7	E	96	1	1
1AO7	E	97	1	1
1AO7	E	98	1	1
1AO7	E	99	1	1
1AO7	E	100	1	1
1AO7	E	101	1	1
1AO7	E	102	1	1
1AO7	E	103	1	1
1AO7	E	104	0	−1
1AO7	E	105	1	1
1AO7	E	106	1	1
1AO7	E	107	0	−1
1AO7	E	108	1	1
1AO7	E	109	0	−1
1AO7	E	110	0	−1
1AO7	E	111	1	1
1AO7	E	112	0	−1
1AO7	E	113	0	−1
1AO7	E	114	0	−1
1AO7	E	115	0	−1

1AO7	E	116	1	−1
1AO7	E	117	1	−1
1AO7	E	118	1	−1
1AO7	E	119	1	−1
1AO7	E	120	1	−1
1AO7	E	121	0	−1
1AO7	E	122	1	−1
1AO7	E	123	1	−1
1AO7	E	124	0	−1
1AO7	E	125	1	−1
1AO7	E	126	0	−1
1AO7	E	127	1	−1
1AO7	E	128	0	−1
1AO7	E	129	1	−1
1AO7	E	144	1	−1
1AO7	E	145	1	−1
1AO7	E	146	0	−1
1AO7	E	147	1	−1
1AO7	E	148	0	−1
1AO7	E	149	1	−1
1AO7	E	150	1	−1
1AO7	E	151	0	−1
1AO7	E	152	1	−1
1AO7	E	153	0	−1
1AO7	E	154	0	−1
1AO7	E	155	0	−1
1AO7	E	156	0	−1
1AO7	E	157	1	−1
1AO7	E	158	0	−1
1AO7	E	159	0	−1
1AO7	E	160	0	−1
1AO7	E	161	1	−1
1AO7	E	162	0	−1
1AO7	E	163	1	−1
1AO7	E	164	1	−1
1AO7	E	165	1	−1
1AO7	E	166	1	−1
1AO7	E	167	0	−1
1AO7	E	168	1	−1
1AO7	E	169	1	−1
1AO7	E	170	1	−1
1AO7	E	171	0	−1
1AO7	E	172	1	−1
1AO7	E	173	1	−1
1AO7	E	174	0	−1
1AO7	E	175	1	1
1AO7	E	176	1	1
1AO7	E	177	0	−1
1AO7	E	178	1	−1
1AO7	E	189	1	−1
1AO7	E	190	0	−1
1AO7	E	191	0	−1

1AO7	E	192	0	−1
1AO7	E	193	0	−1
1AO7	E	194	1	−1
1AO7	E	195	0	−1
1AO7	E	196	1	−1
1AO7	E	197	1	−1
1AO7	E	198	1	−1
1AO7	E	199	1	−1
1AO7	E	200	1	−1
1AO7	E	201	1	−1
1AO7	E	202	1	−1
1AO7	E	203	1	−1
1AO7	E	204	1	−1
1AO7	E	205	1	−1
1AO7	E	206	1	−1
1AO7	E	207	0	−1
1AO7	E	208	0	−1
1AO7	E	209	0	−1
1AO7	E	210	0	−1
1AO7	E	211	0	−1
1AO7	E	212	0	−1
1AO7	E	213	0	−1
1AO7	E	214	1	−1
1AO7	E	215	0	−1
1AO7	E	216	0	−1
1AO7	E	217	0	−1
1AO7	E	218	1	−1
1AO7	E	229	1	−1
1AO7	E	230	1	−1
1AO7	E	231	0	−1
1AO7	E	232	1	−1
1AO7	E	233	1	−1
1AO7	E	234	1	−1
1AO7	E	235	1	−1
1AO7	E	236	0	−1
1AO7	E	237	1	−1
1AO7	E	238	0	−1
1AO7	E	239	1	−1
1AO7	E	240	1	−1
1AO7	E	241	1	−1
1AO7	E	242	0	−1
1AO7	E	243	1	−1
1AO7	E	244	1	−1
1AO7	E	245	1	−1
1ATN	A	2	1	−1
1ATN	A	3	1	−1
1ATN	A	4	1	−1
1ATN	A	5	1	−1
1ATN	A	6	1	−1
1ATN	A	7	0	−1
1ATN	A	8	0	−1
1ATN	A	9	0	−1

1ATN	A	10	0	−1
1ATN	A	11	0	−1
1ATN	A	12	0	−1
1ATN	A	13	0	−1
1ATN	A	14	0	−1
1ATN	A	15	0	−1
1ATN	A	16	0	−1
1ATN	A	17	1	−1
1ATN	A	18	0	−1
1ATN	A	19	0	−1
1ATN	A	20	0	−1
1ATN	A	21	0	−1
1ATN	A	22	0	−1
1ATN	A	23	0	−1
1ATN	A	24	1	−1
1ATN	A	25	1	−1
1ATN	A	26	1	−1
1ATN	A	27	1	−1
1ATN	A	28	0	−1
1ATN	A	29	1	−1
1ATN	A	30	0	−1
1ATN	A	31	1	−1
1ATN	A	32	0	−1
1ATN	A	33	0	−1
1ATN	A	34	0	−1
1ATN	A	35	0	−1
1ATN	A	36	0	−1
1ATN	A	37	0	−1
1ATN	A	38	1	−1
1ATN	A	39	1	1
1ATN	A	40	1	1
1ATN	A	41	1	1
1ATN	A	42	1	1
1ATN	A	43	1	1
1ATN	A	44	1	1
1ATN	A	45	1	1
1ATN	A	46	1	1
1ATN	A	47	1	1
1ATN	A	48	1	1
1ATN	A	49	1	−1
1ATN	A	50	1	−1
1ATN	A	51	1	−1
1ATN	A	52	1	−1
1ATN	A	53	0	−1
1ATN	A	54	0	−1
1ATN	A	55	0	−1
1ATN	A	56	0	−1
1ATN	A	57	1	−1
1ATN	A	58	1	1
1ATN	A	59	0	−1
1ATN	A	60	0	−1
1ATN	A	61	1	1

1ATN	A	62	0	−1
1ATN	A	63	0	−1
1ATN	A	64	1	1
1ATN	A	65	1	1
1ATN	A	66	0	−1
1ATN	A	67	1	−1
1ATN	A	68	1	−1
1ATN	A	69	1	−1
1ATN	A	70	1	−1
1ATN	A	71	0	−1
1ATN	A	72	0	−1
1ATN	A	73	1	−1
1ATN	A	75	0	−1
1ATN	A	76	0	−1
1ATN	A	77	0	−1
1ATN	A	78	0	−1
1ATN	A	79	1	−1
1ATN	A	80	1	−1
1ATN	A	81	0	−1
1ATN	A	82	1	−1
1ATN	A	83	0	−1
1ATN	A	84	0	−1
1ATN	A	85	1	−1
1ATN	A	86	0	−1
1ATN	A	87	0	−1
1ATN	A	88	0	−1
1ATN	A	89	1	−1
1ATN	A	90	0	−1
1ATN	A	91	0	−1
1ATN	A	92	0	−1
1ATN	A	93	1	−1
1ATN	A	94	1	−1
1ATN	A	95	1	−1
1ATN	A	96	0	−1
1ATN	A	97	1	−1
1ATN	A	98	0	−1
1ATN	A	99	1	−1
1ATN	A	100	0	−1
1ATN	A	101	1	−1
1ATN	A	102	1	−1
1ATN	A	103	0	−1
1ATN	A	104	0	−1
1ATN	A	105	0	−1
1ATN	A	106	0	−1
1ATN	A	107	0	−1
1ATN	A	108	0	−1
1ATN	A	109	0	−1
1ATN	A	110	0	−1
1ATN	A	111	0	−1
1ATN	A	112	1	−1
1ATN	A	113	0	−1
1ATN	A	114	1	−1

1ATN	A	115	1	−1
1ATN	A	116	1	−1
1ATN	A	117	0	−1
1ATN	A	118	0	−1
1ATN	A	119	1	−1
1ATN	A	120	1	−1
1ATN	A	121	0	−1
1ATN	A	122	0	−1
1ATN	A	123	0	−1
1ATN	A	124	0	−1
1ATN	A	125	0	−1
1ATN	A	126	0	−1
1ATN	A	127	1	−1
1ATN	A	128	1	−1
1ATN	A	129	0	−1
1ATN	A	130	1	−1
1ATN	A	131	0	−1
1ATN	A	132	1	−1
1ATN	A	133	0	−1
1ATN	A	134	0	−1
1ATN	A	135	0	−1
1ATN	A	136	0	−1
1ATN	A	137	0	−1
1ATN	A	138	0	−1
1ATN	A	139	0	−1
1ATN	A	140	0	−1
1ATN	A	141	0	−1
1ATN	A	142	0	−1
1ATN	A	143	0	−1
1ATN	A	144	0	−1
1ATN	A	145	1	−1
1ATN	A	146	0	−1
1ATN	A	147	0	−1
1ATN	A	148	1	−1
1ATN	A	149	1	−1
1ATN	A	150	1	−1
1ATN	A	151	0	−1
1ATN	A	152	0	−1
1ATN	A	153	0	−1
1ATN	A	154	0	−1
1ATN	A	155	0	−1
1ATN	A	156	0	−1
1ATN	A	157	0	−1
1ATN	A	158	1	−1
1ATN	A	159	1	−1
1ATN	A	160	0	−1
1ATN	A	161	0	−1
1ATN	A	162	0	−1
1ATN	A	163	0	−1
1ATN	A	164	0	−1
1ATN	A	165	0	−1
1ATN	A	166	0	−1

1ATN	A	167	0	−1
1ATN	A	168	1	−1
1ATN	A	169	1	−1
1ATN	A	170	1	−1
1ATN	A	171	1	−1
1ATN	A	172	0	−1
1ATN	A	173	0	−1
1ATN	A	174	1	−1
1ATN	A	175	1	−1
1ATN	A	176	0	−1
1ATN	A	177	0	−1
1ATN	A	178	1	−1
1ATN	A	179	1	−1
1ATN	A	180	0	−1
1ATN	A	181	1	−1
1ATN	A	182	0	−1
1ATN	A	183	0	−1
1ATN	A	184	0	−1
1ATN	A	185	0	−1
1ATN	A	186	1	−1
1ATN	A	187	0	−1
1ATN	A	188	0	−1
1ATN	A	189	1	−1
1ATN	A	190	0	−1
1ATN	A	191	0	−1
1ATN	A	192	0	−1
1ATN	A	193	1	−1
1ATN	A	194	0	−1
1ATN	A	195	0	−1
1ATN	A	196	1	−1
1ATN	A	197	1	−1
1ATN	A	198	1	−1
1ATN	A	199	1	−1
1ATN	A	200	0	−1
1ATN	A	201	1	−1
1ATN	A	202	0	−1
1ATN	A	203	1	1
1ATN	A	204	1	1
1ATN	A	205	1	1
1ATN	A	206	1	1
1ATN	A	207	0	−1
1ATN	A	208	1	−1
1ATN	A	209	1	1
1ATN	A	210	0	−1
1ATN	A	211	0	−1
1ATN	A	212	1	−1
1ATN	A	213	0	−1
1ATN	A	214	0	−1
1ATN	A	215	0	−1
1ATN	A	216	1	−1
1ATN	A	217	1	−1
1ATN	A	218	0	−1

1ATN	A	219	0	−1
1ATN	A	220	1	−1
1ATN	A	221	0	−1
1ATN	A	222	0	−1
1ATN	A	223	1	−1
1ATN	A	224	1	−1
1ATN	A	225	0	−1
1ATN	A	226	1	−1
1ATN	A	227	1	−1
1ATN	A	228	1	−1
1ATN	A	229	1	−1
1ATN	A	230	1	−1
1ATN	A	231	1	−1
1ATN	A	232	0	−1
1ATN	A	233	1	−1
1ATN	A	234	1	−1
1ATN	A	235	1	−1
1ATN	A	236	1	−1
1ATN	A	237	1	−1
1ATN	A	238	0	−1
1ATN	A	239	1	−1
1ATN	A	240	1	−1
1ATN	A	241	1	−1
1ATN	A	242	0	−1
1ATN	A	243	1	−1
1ATN	A	244	0	−1
1ATN	A	245	1	−1
1ATN	A	246	1	−1
1ATN	A	247	1	−1
1ATN	A	248	1	−1
1ATN	A	249	1	−1
1ATN	A	250	0	−1
1ATN	A	251	1	−1
1ATN	A	252	0	−1
1ATN	A	253	0	−1
1ATN	A	254	0	−1
1ATN	A	255	0	−1
1ATN	A	256	0	−1
1ATN	A	257	0	−1
1ATN	A	258	0	−1
1ATN	A	259	0	−1
1ATN	A	260	0	−1
1ATN	A	261	0	−1
1ATN	A	262	0	−1
1ATN	A	263	0	−1
1ATN	A	264	0	−1
1ATN	A	265	0	−1
1ATN	A	266	0	−1
1ATN	A	267	1	−1
1ATN	A	268	1	−1
1ATN	A	269	1	−1
1ATN	A	270	1	−1

1ATN	A	271	1	−1
1ATN	A	272	1	−1
1ATN	A	273	1	−1
1ATN	A	274	1	−1
1ATN	A	275	0	−1
1ATN	A	276	0	−1
1ATN	A	277	0	−1
1ATN	A	278	1	−1
1ATN	A	279	0	−1
1ATN	A	280	0	−1
1ATN	A	281	0	−1
1ATN	A	282	1	−1
1ATN	A	283	0	−1
1ATN	A	284	0	−1
1ATN	A	285	1	−1
1ATN	A	286	1	−1
1ATN	A	287	0	−1
1ATN	A	288	1	−1
1ATN	A	289	1	−1
1ATN	A	290	1	−1
1ATN	A	291	0	−1
1ATN	A	292	0	−1
1ATN	A	293	1	−1
1ATN	A	294	1	−1
1ATN	A	295	0	−1
1ATN	A	296	0	−1
1ATN	A	297	0	−1
1ATN	A	298	0	−1
1ATN	A	299	0	−1
1ATN	A	300	0	−1
1ATN	A	301	0	−1
1ATN	A	302	0	−1
1ATN	A	303	0	−1
1ATN	A	304	1	−1
1ATN	A	305	0	−1
1ATN	A	306	0	−1
1ATN	A	307	1	−1
1ATN	A	308	0	−1
1ATN	A	309	1	−1
1ATN	A	310	0	−1
1ATN	A	311	0	−1
1ATN	A	312	0	−1
1ATN	A	313	1	−1
1ATN	A	314	0	−1
1ATN	A	315	0	−1
1ATN	A	316	1	−1
1ATN	A	317	1	−1
1ATN	A	318	0	−1
1ATN	A	319	0	−1
1ATN	A	320	1	−1
1ATN	A	321	1	−1
1ATN	A	322	1	−1

1ATN	A	323	0	−1
1ATN	A	324	1	−1
1ATN	A	325	1	−1
1ATN	A	326	1	−1
1ATN	A	327	1	−1
1ATN	A	328	1	−1
1ATN	A	329	0	−1
1ATN	A	330	1	−1
1ATN	A	331	0	−1
1ATN	A	332	0	−1
1ATN	A	333	1	−1
1ATN	A	334	0	−1
1ATN	A	335	1	−1
1ATN	A	336	1	−1
1ATN	A	337	0	−1
1ATN	A	338	1	−1
1ATN	A	339	0	−1
1ATN	A	340	0	−1
1ATN	A	341	0	−1
1ATN	A	342	0	−1
1ATN	A	343	0	−1
1ATN	A	344	0	−1
1ATN	A	345	0	−1
1ATN	A	346	0	−1
1ATN	A	347	1	−1
1ATN	A	348	0	−1
1ATN	A	349	0	−1
1ATN	A	350	1	−1
1ATN	A	351	1	−1
1ATN	A	352	1	−1
1ATN	A	353	1	−1
1ATN	A	354	0	−1
1ATN	A	355	1	−1
1ATN	A	356	1	−1
1ATN	A	357	1	−1
1ATN	A	358	0	−1
1ATN	A	359	0	−1
1ATN	A	360	1	−1
1ATN	A	361	1	−1
1ATN	A	362	1	−1
1ATN	A	363	1	−1
1ATN	A	364	0	−1
1ATN	A	365	1	−1
1ATN	A	366	1	−1
1ATN	A	367	1	−1
1ATN	A	368	1	−1
1ATN	A	369	1	−1
1ATN	A	370	1	−1
1ATN	A	371	1	−1
1ATN	A	372	0	−1
1ATN	A	373	0	−1
1ATN	D	1	0	−1

1ATN	D	2	1	−1
1ATN	D	3	0	−1
1ATN	D	4	0	−1
1ATN	D	5	0	−1
1ATN	D	6	0	−1
1ATN	D	7	0	−1
1ATN	D	8	0	−1
1ATN	D	9	1	−1
1ATN	D	10	1	−1
1ATN	D	11	0	−1
1ATN	D	12	0	−1
1ATN	D	13	1	1
1ATN	D	14	1	1
1ATN	D	15	0	−1
1ATN	D	16	0	−1
1ATN	D	17	1	−1
1ATN	D	18	1	−1
1ATN	D	19	1	−1
1ATN	D	20	1	−1
1ATN	D	21	0	−1
1ATN	D	22	0	−1
1ATN	D	23	1	−1
1ATN	D	24	0	−1
1ATN	D	25	0	−1
1ATN	D	26	0	−1
1ATN	D	27	1	−1
1ATN	D	28	0	−1
1ATN	D	29	0	−1
1ATN	D	30	1	−1
1ATN	D	31	1	−1
1ATN	D	32	0	−1
1ATN	D	33	0	−1
1ATN	D	34	0	−1
1ATN	D	35	0	−1
1ATN	D	36	0	−1
1ATN	D	37	0	−1
1ATN	D	38	0	−1
1ATN	D	39	0	−1
1ATN	D	40	0	−1
1ATN	D	41	0	−1
1ATN	D	42	0	−1
1ATN	D	43	1	1
1ATN	D	44	1	1
1ATN	D	45	1	1
1ATN	D	46	1	1
1ATN	D	47	0	−1
1ATN	D	48	0	−1
1ATN	D	49	1	1
1ATN	D	50	1	−1
1ATN	D	51	0	−1
1ATN	D	52	0	−1
1ATN	D	53	1	1

1ATN	D	54	1	−1
1ATN	D	55	0	−1
1ATN	D	56	0	−1
1ATN	D	57	1	−1
1ATN	D	58	1	−1
1ATN	D	59	1	−1
1ATN	D	60	1	1
1ATN	D	61	1	1
1ATN	D	62	0	−1
1ATN	D	63	0	−1
1ATN	D	64	1	1
1ATN	D	65	1	1
1ATN	D	66	0	−1
1ATN	D	67	1	1
1ATN	D	68	0	−1
1ATN	D	69	1	1
1ATN	D	70	1	−1
1ATN	D	71	0	−1
1ATN	D	72	0	−1
1ATN	D	73	1	−1
1ATN	D	74	1	−1
1ATN	D	75	1	−1
1ATN	D	76	1	−1
1ATN	D	77	1	−1
1ATN	D	78	0	−1
1ATN	D	79	0	−1
1ATN	D	80	0	−1
1ATN	D	81	0	−1
1ATN	D	82	0	−1
1ATN	D	83	0	−1
1ATN	D	84	0	−1
1ATN	D	85	0	−1
1ATN	D	86	1	−1
1ATN	D	87	1	−1
1ATN	D	88	1	−1
1ATN	D	89	0	−1
1ATN	D	90	1	−1
1ATN	D	91	1	1
1ATN	D	92	1	−1
1ATN	D	93	1	−1
1ATN	D	94	1	1
1ATN	D	95	0	−1
1ATN	D	96	1	1
1ATN	D	97	0	−1
1ATN	D	98	1	−1
1ATN	D	99	0	−1
1ATN	D	100	1	−1
1ATN	D	101	1	−1
1ATN	D	104	1	−1
1ATN	D	105	1	−1
1ATN	D	106	1	−1
1ATN	D	107	0	−1

1ATN	D	108	1	−1
1ATN	D	109	0	−1
1ATN	D	110	0	−1
1ATN	D	111	0	−1
1ATN	D	112	0	−1
1ATN	D	113	0	−1
1ATN	D	114	0	−1
1ATN	D	115	0	−1
1ATN	D	116	0	−1
1ATN	D	117	0	−1
1ATN	D	118	0	−1
1ATN	D	119	0	−1
1ATN	D	120	0	−1
1ATN	D	121	1	−1
1ATN	D	122	1	−1
1ATN	D	123	0	−1
1ATN	D	124	1	−1
1ATN	D	125	0	−1
1ATN	D	126	1	−1
1ATN	D	127	1	−1
1ATN	D	128	0	−1
1ATN	D	129	0	−1
1ATN	D	130	0	−1
1ATN	D	131	0	−1
1ATN	D	132	0	−1
1ATN	D	133	0	−1
1ATN	D	134	0	−1
1ATN	D	135	0	−1
1ATN	D	136	0	−1
1ATN	D	137	1	−1
1ATN	D	138	1	−1
1ATN	D	139	1	−1
1ATN	D	140	0	−1
1ATN	D	141	0	−1
1ATN	D	142	1	−1
1ATN	D	143	0	−1
1ATN	D	144	0	−1
1ATN	D	145	0	−1
1ATN	D	146	0	−1
1ATN	D	147	0	−1
1ATN	D	148	0	−1
1ATN	D	149	1	−1
1ATN	D	150	0	−1
1ATN	D	151	0	−1
1ATN	D	152	1	−1
1ATN	D	153	0	−1
1ATN	D	154	0	−1
1ATN	D	155	0	−1
1ATN	D	156	1	−1
1ATN	D	157	1	−1
1ATN	D	158	0	−1
1ATN	D	159	1	−1

1ATN	D	160	0	−1
1ATN	D	161	1	−1
1ATN	D	162	0	−1
1ATN	D	163	0	−1
1ATN	D	164	0	−1
1ATN	D	165	0	−1
1ATN	D	166	0	−1
1ATN	D	167	0	−1
1ATN	D	168	0	−1
1ATN	D	169	0	−1
1ATN	D	170	0	−1
1ATN	D	171	0	−1
1ATN	D	172	0	−1
1ATN	D	173	1	−1
1ATN	D	174	1	−1
1ATN	D	175	1	−1
1ATN	D	176	0	−1
1ATN	D	177	1	−1
1ATN	D	178	1	−1
1ATN	D	179	1	−1
1ATN	D	180	1	−1
1ATN	D	181	0	−1
1ATN	D	182	1	−1
1ATN	D	183	1	−1
1ATN	D	184	0	−1
1ATN	D	185	1	−1
1ATN	D	186	0	−1
1ATN	D	187	1	−1
1ATN	D	188	1	−1
1ATN	D	189	0	−1
1ATN	D	190	1	−1
1ATN	D	191	1	−1
1ATN	D	192	0	−1
1ATN	D	193	1	−1
1ATN	D	194	0	−1
1ATN	D	195	0	−1
1ATN	D	196	0	−1
1ATN	D	197	1	−1
1ATN	D	198	1	−1
1ATN	D	199	1	−1
1ATN	D	200	0	−1
1ATN	D	201	1	−1
1ATN	D	202	0	−1
1ATN	D	203	0	−1
1ATN	D	204	0	−1
1ATN	D	205	1	−1
1ATN	D	206	1	−1
1ATN	D	207	1	−1
1ATN	D	208	1	−1
1ATN	D	209	0	−1
1ATN	D	210	0	−1
1ATN	D	211	0	−1

1ATN	D	212	0	−1
1ATN	D	213	0	−1
1ATN	D	214	0	−1
1ATN	D	215	0	−1
1ATN	D	216	0	−1
1ATN	D	217	0	−1
1ATN	D	218	0	−1
1ATN	D	219	1	−1
1ATN	D	220	1	−1
1ATN	D	221	0	−1
1ATN	D	222	1	−1
1ATN	D	223	1	−1
1ATN	D	224	0	−1
1ATN	D	225	0	−1
1ATN	D	226	1	−1
1ATN	D	227	1	−1
1ATN	D	228	1	−1
1ATN	D	229	0	−1
1ATN	D	230	0	−1
1ATN	D	231	1	−1
1ATN	D	232	1	−1
1ATN	D	233	0	−1
1ATN	D	234	1	−1
1ATN	D	235	0	−1
1ATN	D	236	0	−1
1ATN	D	237	1	−1
1ATN	D	238	1	−1
1ATN	D	239	0	−1
1ATN	D	240	1	−1
1ATN	D	241	0	−1
1ATN	D	242	1	−1
1ATN	D	243	1	−1
1ATN	D	244	1	−1
1ATN	D	245	0	−1
1ATN	D	246	0	−1
1ATN	D	247	1	−1
1ATN	D	248	1	−1
1ATN	D	249	0	−1
1ATN	D	250	0	−1
1ATN	D	251	0	−1
1ATN	D	252	0	−1
1ATN	D	253	0	−1
1ATN	D	254	0	−1
1ATN	D	255	0	−1
1ATN	D	256	0	−1
1ATN	D	257	0	−1
1ATN	D	258	0	−1
1ATN	D	259	0	−1
1ATN	D	260	1	−1
1AVW	A	1	0	−1
1AVW	A	2	0	−1
1AVW	A	3	1	−1

1AVW	A	4	1	−1
1AVW	A	5	1	−1
1AVW	A	6	1	−1
1AVW	A	7	0	−1
1AVW	A	8	1	−1
1AVW	A	9	1	−1
1AVW	A	10	0	−1
1AVW	A	11	1	−1
1AVW	A	12	0	−1
1AVW	A	13	0	−1
1AVW	A	14	0	−1
1AVW	A	15	0	−1
1AVW	A	16	0	−1
1AVW	A	17	0	−1
1AVW	A	18	0	−1
1AVW	A	19	0	−1
1AVW	A	20	1	1
1AVW	A	21	1	−1
1AVW	A	22	1	1
1AVW	A	23	0	−1
1AVW	A	24	1	1
1AVW	A	25	0	−1
1AVW	A	26	0	−1
1AVW	A	27	0	−1
1AVW	A	28	0	−1
1AVW	A	29	0	−1
1AVW	A	30	0	−1
1AVW	A	31	1	−1
1AVW	A	32	1	−1
1AVW	A	33	1	−1
1AVW	A	34	0	−1
1AVW	A	35	0	−1
1AVW	A	36	0	−1
1AVW	A	37	0	−1
1AVW	A	38	0	−1
1AVW	A	39	0	−1
1AVW	A	40	1	1
1AVW	A	41	0	−1
1AVW	A	42	1	−1
1AVW	A	43	1	1
1AVW	A	44	1	1
1AVW	A	45	1	−1
1AVW	A	46	0	−1
1AVW	A	47	0	−1
1AVW	A	48	0	−1
1AVW	A	49	0	−1
1AVW	A	50	0	−1
1AVW	A	51	0	−1
1AVW	A	52	0	−1
1AVW	A	53	0	−1
1AVW	A	54	0	−1
1AVW	A	55	0	−1

1AVW	A	56	1	−1
1AVW	A	57	1	−1
1AVW	A	58	1	−1
1AVW	A	59	1	−1
1AVW	A	60	1	−1
1AVW	A	61	1	−1
1AVW	A	62	0	−1
1AVW	A	63	0	−1
1AVW	A	64	1	−1
1AVW	A	65	0	−1
1AVW	A	66	1	−1
1AVW	A	67	0	−1
1AVW	A	68	1	−1
1AVW	A	69	1	−1
1AVW	A	70	0	−1
1AVW	A	71	1	−1
1AVW	A	72	1	−1
1AVW	A	73	0	−1
1AVW	A	74	1	−1
1AVW	A	75	1	−1
1AVW	A	76	1	1
1AVW	A	77	1	−1
1AVW	A	78	1	1
1AVW	A	79	1	1
1AVW	A	80	1	−1
1AVW	A	81	1	1
1AVW	A	82	0	−1
1AVW	A	83	0	−1
1AVW	A	84	0	−1
1AVW	A	85	0	−1
1AVW	A	86	0	−1
1AVW	A	87	0	−1
1AVW	A	88	0	−1
1AVW	A	89	0	−1
1AVW	A	90	0	−1
1AVW	A	91	1	−1
1AVW	A	92	1	−1
1AVW	A	93	1	−1
1AVW	A	94	0	−1
1AVW	A	95	1	−1
1AVW	A	96	1	−1
1AVW	A	97	1	−1
1AVW	A	98	1	−1
1AVW	A	99	0	−1
1AVW	A	100	0	−1
1AVW	A	101	1	−1
1AVW	A	102	1	−1
1AVW	A	103	0	−1
1AVW	A	104	1	−1
1AVW	A	105	0	−1
1AVW	A	106	0	−1
1AVW	A	107	1	−1

1AVW	A	108	1	−1
1AVW	A	109	1	−1
1AVW	A	110	1	−1
1AVW	A	111	1	−1
1AVW	A	112	1	−1
1AVW	A	113	1	−1
1AVW	A	114	0	−1
1AVW	A	115	1	−1
1AVW	A	116	0	−1
1AVW	A	117	0	−1
1AVW	A	118	0	−1
1AVW	A	119	0	−1
1AVW	A	120	0	−1
1AVW	A	121	0	−1
1AVW	A	122	0	−1
1AVW	A	123	0	−1
1AVW	A	124	1	−1
1AVW	A	125	1	−1
1AVW	A	126	1	−1
1AVW	A	127	1	−1
1AVW	A	128	1	−1
1AVW	A	129	1	1
1AVW	A	130	1	−1
1AVW	A	131	1	1
1AVW	A	132	0	−1
1AVW	A	133	1	−1
1AVW	A	134	1	−1
1AVW	A	135	0	−1
1AVW	A	136	0	−1
1AVW	A	137	0	−1
1AVW	A	138	0	−1
1AVW	A	139	1	−1
1AVW	A	140	0	−1
1AVW	A	141	0	−1
1AVW	A	142	0	−1
1AVW	A	143	0	−1
1AVW	A	144	1	−1
1AVW	A	145	1	−1
1AVW	A	146	1	−1
1AVW	A	147	1	−1
1AVW	A	148	0	−1
1AVW	A	149	1	−1
1AVW	A	150	1	−1
1AVW	A	151	0	−1
1AVW	A	152	0	−1
1AVW	A	153	1	−1
1AVW	A	154	1	−1
1AVW	A	155	1	1
1AVW	A	156	0	−1
1AVW	A	157	1	−1
1AVW	A	158	1	−1
1AVW	A	159	0	−1

1AVW	A	160	0	−1
1AVW	A	161	0	−1
1AVW	A	162	0	−1
1AVW	A	163	0	−1
1AVW	A	164	0	−1
1AVW	A	165	0	−1
1AVW	A	166	1	−1
1AVW	A	167	1	−1
1AVW	A	168	0	−1
1AVW	A	169	1	−1
1AVW	A	170	1	−1
1AVW	A	171	0	−1
1AVW	A	172	0	−1
1AVW	A	173	0	−1
1AVW	A	174	1	1
1AVW	A	175	0	−1
1AVW	A	176	0	−1
1AVW	A	177	0	−1
1AVW	A	178	0	−1
1AVW	A	179	0	−1
1AVW	A	180	0	−1
1AVW	A	181	0	−1
1AVW	A	182	0	−1
1AVW	A	183	0	−1
1AVW	A	184	1	−1
1AVW	A	185	1	−1
1AVW	A	186	0	−1
1AVW	A	187	0	−1
1AVW	A	188	0	−1
1AVW	A	189	0	−1
1AVW	A	190	0	−1
1AVW	A	191	0	−1
1AVW	A	192	0	−1
1AVW	A	193	0	−1
1AVW	A	194	1	1
1AVW	A	195	1	1
1AVW	A	196	1	1
1AVW	A	197	0	−1
1AVW	A	198	0	−1
1AVW	A	199	1	−1
1AVW	A	200	1	−1
1AVW	A	201	1	−1
1AVW	A	202	1	−1
1AVW	A	203	0	−1
1AVW	A	204	0	−1
1AVW	A	205	0	−1
1AVW	A	206	0	−1
1AVW	A	207	0	−1
1AVW	A	208	1	−1
1AVW	A	209	0	−1
1AVW	A	210	0	−1
1AVW	A	211	1	−1

1AVW	A	212	0	−1
1AVW	A	213	0	−1
1AVW	A	214	1	−1
1AVW	A	215	0	−1
1AVW	A	216	0	−1
1AVW	A	217	1	−1
1AVW	A	218	1	−1
1AVW	A	219	0	−1
1AVW	A	220	0	−1
1AVW	A	221	1	−1
1AVW	A	222	1	−1
1AVW	A	223	1	−1
1AVW	B	1	1	1
1AVW	B	2	1	1
1AVW	B	3	0	−1
1AVW	B	4	0	−1
1AVW	B	5	0	−1
1AVW	B	6	1	−1
1AVW	B	7	1	−1
1AVW	B	8	1	−1
1AVW	B	9	1	−1
1AVW	B	10	1	−1
1AVW	B	11	0	−1
1AVW	B	12	1	1
1AVW	B	13	0	−1
1AVW	B	14	1	−1
1AVW	B	15	0	−1
1AVW	B	16	0	−1
1AVW	B	17	0	−1
1AVW	B	18	1	−1
1AVW	B	19	0	−1
1AVW	B	20	0	−1
1AVW	B	21	0	−1
1AVW	B	22	1	−1
1AVW	B	23	1	−1
1AVW	B	24	1	−1
1AVW	B	25	1	−1
1AVW	B	26	1	−1
1AVW	B	27	0	−1
1AVW	B	28	0	−1
1AVW	B	29	0	−1
1AVW	B	30	1	−1
1AVW	B	31	0	−1
1AVW	B	32	0	−1
1AVW	B	33	1	−1
1AVW	B	34	0	−1
1AVW	B	35	1	−1
1AVW	B	36	1	−1
1AVW	B	37	0	−1
1AVW	B	38	1	−1
1AVW	B	39	0	−1
1AVW	B	40	0	−1

1AVW	B	41	0	−1
1AVW	B	42	0	−1
1AVW	B	43	0	−1
1AVW	B	44	0	−1
1AVW	B	45	0	−1
1AVW	B	46	0	−1
1AVW	B	47	1	−1
1AVW	B	48	1	−1
1AVW	B	49	1	−1
1AVW	B	50	1	−1
1AVW	B	51	0	−1
1AVW	B	52	1	−1
1AVW	B	53	0	−1
1AVW	B	54	0	−1
1AVW	B	55	0	−1
1AVW	B	56	0	−1
1AVW	B	57	0	−1
1AVW	B	58	0	−1
1AVW	B	59	1	−1
1AVW	B	60	0	−1
1AVW	B	61	1	1
1AVW	B	62	1	1
1AVW	B	63	1	1
1AVW	B	64	1	1
1AVW	B	65	1	1
1AVW	B	66	0	−1
1AVW	B	67	0	−1
1AVW	B	68	0	−1
1AVW	B	69	1	−1
1AVW	B	70	1	1
1AVW	B	71	1	1
1AVW	B	72	0	−1
1AVW	B	73	0	−1
1AVW	B	74	1	−1
1AVW	B	75	0	−1
1AVW	B	76	1	−1
1AVW	B	77	0	−1
1AVW	B	78	1	−1
1AVW	B	79	1	−1
1AVW	B	80	0	−1
1AVW	B	81	1	−1
1AVW	B	82	1	−1
1AVW	B	83	1	−1
1AVW	B	84	1	−1
1AVW	B	85	1	−1
1AVW	B	86	0	−1
1AVW	B	87	1	−1
1AVW	B	88	1	−1
1AVW	B	89	1	−1
1AVW	B	90	1	−1
1AVW	B	91	0	−1
1AVW	B	92	1	−1

1AVW	B	93	0	−1
1AVW	B	94	0	−1
1AVW	B	95	0	−1
1AVW	B	96	0	−1
1AVW	B	97	1	−1
1AVW	B	98	1	−1
1AVW	B	99	1	−1
1AVW	B	100	1	−1
1AVW	B	101	0	−1
1AVW	B	102	1	−1
1AVW	B	103	1	−1
1AVW	B	104	0	−1
1AVW	B	105	0	−1
1AVW	B	106	0	−1
1AVW	B	107	0	−1
1AVW	B	108	0	−1
1AVW	B	109	1	−1
1AVW	B	110	1	−1
1AVW	B	111	1	−1
1AVW	B	112	1	−1
1AVW	B	113	1	−1
1AVW	B	114	0	−1
1AVW	B	115	1	−1
1AVW	B	116	1	−1
1AVW	B	117	1	1
1AVW	B	118	0	−1
1AVW	B	119	0	−1
1AVW	B	120	0	−1
1AVW	B	121	1	−1
1AVW	B	122	1	−1
1AVW	B	123	1	−1
1AVW	B	124	1	−1
1AVW	B	127	1	−1
1AVW	B	128	1	−1
1AVW	B	129	0	−1
1AVW	B	130	0	−1
1AVW	B	131	0	−1
1AVW	B	132	0	−1
1AVW	B	133	0	−1
1AVW	B	134	0	−1
1AVW	B	135	0	−1
1AVW	B	136	0	−1
1AVW	B	137	1	−1
1AVW	B	138	1	−1
1AVW	B	143	1	1
1AVW	B	144	1	−1
1AVW	B	145	1	−1
1AVW	B	146	1	−1
1AVW	B	147	0	−1
1AVW	B	148	0	−1
1AVW	B	149	0	−1
1AVW	B	150	0	−1

1AVW	B	151	0	−1
1AVW	B	152	1	−1
1AVW	B	153	0	−1
1AVW	B	154	1	−1
1AVW	B	155	1	−1
1AVW	B	156	1	−1
1AVW	B	157	1	−1
1AVW	B	158	0	−1
1AVW	B	159	0	−1
1AVW	B	160	0	−1
1AVW	B	161	0	−1
1AVW	B	162	0	−1
1AVW	B	163	1	−1
1AVW	B	164	0	−1
1AVW	B	165	1	−1
1AVW	B	166	1	−1
1AVW	B	167	0	−1
1AVW	B	168	1	−1
1AVW	B	169	0	−1
1AVW	B	170	0	−1
1AVW	B	171	0	−1
1AVW	B	172	0	−1
1AVW	B	173	0	−1
1AVW	B	174	0	−1
1AVW	B	175	1	−1
1AVW	B	176	1	−1
1AVW	B	177	1	−1
1BRS	A	3	1	−1
1BRS	A	4	1	−1
1BRS	A	5	0	−1
1BRS	A	6	1	−1
1BRS	A	7	0	−1
1BRS	A	8	1	−1
1BRS	A	9	1	−1
1BRS	A	10	0	−1
1BRS	A	11	0	−1
1BRS	A	12	1	−1
1BRS	A	13	0	−1
1BRS	A	14	0	−1
1BRS	A	15	1	−1
1BRS	A	16	1	−1
1BRS	A	17	1	−1
1BRS	A	18	1	−1
1BRS	A	19	1	−1
1BRS	A	20	0	−1
1BRS	A	21	0	−1
1BRS	A	22	1	−1
1BRS	A	23	0	−1
1BRS	A	24	0	−1
1BRS	A	25	0	−1
1BRS	A	26	0	−1
1BRS	A	27	1	1

1BRS	A	28	1	−1
1BRS	A	29	1	−1
1BRS	A	30	0	−1
1BRS	A	31	1	1
1BRS	A	32	1	−1
1BRS	A	33	1	−1
1BRS	A	34	1	−1
1BRS	A	35	0	−1
1BRS	A	36	1	−1
1BRS	A	37	1	1
1BRS	A	38	1	1
1BRS	A	39	1	−1
1BRS	A	40	0	−1
1BRS	A	41	0	−1
1BRS	A	42	0	−1
1BRS	A	43	0	−1
1BRS	A	44	1	−1
1BRS	A	45	1	−1
1BRS	A	46	0	−1
1BRS	A	47	1	−1
1BRS	A	48	1	−1
1BRS	A	49	1	−1
1BRS	A	50	0	−1
1BRS	A	51	0	−1
1BRS	A	52	0	−1
1BRS	A	53	0	−1
1BRS	A	54	0	−1
1BRS	A	55	1	1
1BRS	A	56	0	−1
1BRS	A	57	1	1
1BRS	A	58	0	−1
1BRS	A	59	1	1
1BRS	A	60	1	1
1BRS	A	61	1	−1
1BRS	A	62	1	1
1BRS	A	63	0	−1
1BRS	A	64	1	−1
1BRS	A	65	1	−1
1BRS	A	66	1	−1
1BRS	A	67	1	−1
1BRS	A	68	1	−1
1BRS	A	69	0	−1
1BRS	A	70	1	−1
1BRS	A	71	0	−1
1BRS	A	72	1	−1
1BRS	A	73	0	−1
1BRS	A	74	0	−1
1BRS	A	75	0	−1
1BRS	A	76	0	−1
1BRS	A	77	1	−1
1BRS	A	78	0	−1
1BRS	A	79	1	−1

1BRS	A	80	1	−1
1BRS	A	81	1	−1
1BRS	A	82	1	1
1BRS	A	83	1	1
1BRS	A	84	1	1
1BRS	A	85	1	1
1BRS	A	86	0	−1
1BRS	A	87	0	−1
1BRS	A	88	0	−1
1BRS	A	89	0	−1
1BRS	A	90	0	−1
1BRS	A	91	0	−1
1BRS	A	92	1	−1
1BRS	A	93	1	−1
1BRS	A	94	1	−1
1BRS	A	95	0	−1
1BRS	A	96	0	−1
1BRS	A	97	0	−1
1BRS	A	98	0	−1
1BRS	A	99	0	−1
1BRS	A	100	1	−1
1BRS	A	101	1	1
1BRS	A	102	1	1
1BRS	A	103	0	−1
1BRS	A	104	1	1
1BRS	A	105	1	−1
1BRS	A	106	0	−1
1BRS	A	107	1	−1
1BRS	A	108	0	−1
1BRS	A	109	0	−1
1BRS	A	110	0	−1
1BRS	B	1	1	−1
1BRS	B	2	1	−1
1BRS	B	3	1	−1
1BRS	B	4	1	−1
1BRS	B	5	0	−1
1BRS	B	6	1	−1
1BRS	B	7	0	−1
1BRS	B	8	1	−1
1BRS	B	9	1	−1
1BRS	B	10	0	−1
1BRS	B	11	0	−1
1BRS	B	12	1	−1
1BRS	B	13	0	−1
1BRS	B	14	0	−1
1BRS	B	15	1	−1
1BRS	B	16	1	−1
1BRS	B	17	1	−1
1BRS	B	18	1	−1
1BRS	B	19	1	−1
1BRS	B	20	0	−1
1BRS	B	21	0	−1

1BRS	B	22	1	−1
1BRS	B	23	0	−1
1BRS	B	24	0	−1
1BRS	B	25	0	−1
1BRS	B	26	0	−1
1BRS	B	27	1	1
1BRS	B	28	1	−1
1BRS	B	29	1	−1
1BRS	B	30	0	−1
1BRS	B	31	1	1
1BRS	B	32	1	−1
1BRS	B	33	1	−1
1BRS	B	34	1	−1
1BRS	B	35	0	−1
1BRS	B	36	1	−1
1BRS	B	37	1	1
1BRS	B	38	1	1
1BRS	B	39	1	−1
1BRS	B	40	0	−1
1BRS	B	41	0	−1
1BRS	B	42	0	−1
1BRS	B	43	1	−1
1BRS	B	44	1	−1
1BRS	B	45	1	−1
1BRS	B	46	0	−1
1BRS	B	47	1	−1
1BRS	B	48	1	−1
1BRS	B	49	1	−1
1BRS	B	50	0	−1
1BRS	B	51	0	−1
1BRS	B	52	0	−1
1BRS	B	53	0	−1
1BRS	B	54	0	−1
1BRS	B	55	1	1
1BRS	B	56	0	−1
1BRS	B	57	1	1
1BRS	B	58	0	−1
1BRS	B	59	1	1
1BRS	B	60	1	1
1BRS	B	61	1	−1
1BRS	B	62	1	1
1BRS	B	63	0	−1
1BRS	B	64	1	−1
1BRS	B	65	1	−1
1BRS	B	66	1	−1
1BRS	B	67	1	−1
1BRS	B	68	1	−1
1BRS	B	69	0	−1
1BRS	B	70	1	−1
1BRS	B	71	0	−1
1BRS	B	72	1	−1
1BRS	B	73	0	−1

1BRS	B	74	0	−1
1BRS	B	75	0	−1
1BRS	B	76	0	−1
1BRS	B	77	1	−1
1BRS	B	78	0	−1
1BRS	B	79	1	−1
1BRS	B	80	1	−1
1BRS	B	81	1	−1
1BRS	B	82	1	1
1BRS	B	83	1	1
1BRS	B	84	1	1
1BRS	B	85	1	1
1BRS	B	86	0	−1
1BRS	B	87	0	−1
1BRS	B	88	0	−1
1BRS	B	89	0	−1
1BRS	B	90	0	−1
1BRS	B	91	0	−1
1BRS	B	92	1	−1
1BRS	B	93	1	−1
1BRS	B	94	1	−1
1BRS	B	95	0	−1
1BRS	B	96	0	−1
1BRS	B	97	0	−1
1BRS	B	98	0	−1
1BRS	B	99	0	−1
1BRS	B	100	1	−1
1BRS	B	101	1	1
1BRS	B	102	1	1
1BRS	B	103	0	−1
1BRS	B	104	1	1
1BRS	B	105	1	−1
1BRS	B	106	0	−1
1BRS	B	107	1	−1
1BRS	B	108	0	−1
1BRS	B	109	0	−1
1BRS	B	110	0	−1
1BRS	C	3	1	−1
1BRS	C	4	1	−1
1BRS	C	5	0	−1
1BRS	C	6	1	−1
1BRS	C	7	0	−1
1BRS	C	8	1	−1
1BRS	C	9	1	−1
1BRS	C	10	0	−1
1BRS	C	11	0	−1
1BRS	C	12	1	−1
1BRS	C	13	1	−1
1BRS	C	14	0	−1
1BRS	C	15	1	−1
1BRS	C	16	1	−1
1BRS	C	17	1	−1

1BRS	C	18	1	−1
1BRS	C	19	0	−1
1BRS	C	20	0	−1
1BRS	C	21	0	−1
1BRS	C	22	1	−1
1BRS	C	23	0	−1
1BRS	C	24	0	−1
1BRS	C	25	0	−1
1BRS	C	26	0	−1
1BRS	C	27	1	1
1BRS	C	28	1	−1
1BRS	C	29	1	−1
1BRS	C	30	0	−1
1BRS	C	31	1	−1
1BRS	C	32	1	−1
1BRS	C	33	1	−1
1BRS	C	34	1	−1
1BRS	C	35	0	−1
1BRS	C	36	1	−1
1BRS	C	37	1	1
1BRS	C	38	1	1
1BRS	C	39	1	−1
1BRS	C	40	0	−1
1BRS	C	41	0	−1
1BRS	C	42	0	−1
1BRS	C	43	0	−1
1BRS	C	44	1	−1
1BRS	C	45	1	−1
1BRS	C	46	0	−1
1BRS	C	47	1	−1
1BRS	C	48	1	−1
1BRS	C	49	0	−1
1BRS	C	50	0	−1
1BRS	C	51	0	−1
1BRS	C	52	0	−1
1BRS	C	53	0	−1
1BRS	C	54	0	−1
1BRS	C	55	1	1
1BRS	C	56	0	−1
1BRS	C	57	1	1
1BRS	C	58	0	−1
1BRS	C	59	1	1
1BRS	C	60	1	1
1BRS	C	61	1	−1
1BRS	C	62	1	1
1BRS	C	63	0	−1
1BRS	C	64	1	−1
1BRS	C	65	1	−1
1BRS	C	66	1	−1
1BRS	C	67	1	−1
1BRS	C	68	1	−1
1BRS	C	69	0	−1

1BRS	C	70	1	−1
1BRS	C	71	0	−1
1BRS	C	72	1	−1
1BRS	C	73	0	−1
1BRS	C	74	0	−1
1BRS	C	75	0	−1
1BRS	C	76	0	−1
1BRS	C	77	1	−1
1BRS	C	78	0	−1
1BRS	C	79	1	−1
1BRS	C	80	1	−1
1BRS	C	81	1	−1
1BRS	C	82	1	1
1BRS	C	83	1	1
1BRS	C	84	1	1
1BRS	C	85	1	1
1BRS	C	86	0	−1
1BRS	C	87	0	−1
1BRS	C	88	0	−1
1BRS	C	89	0	−1
1BRS	C	90	0	−1
1BRS	C	91	0	−1
1BRS	C	92	1	−1
1BRS	C	93	1	−1
1BRS	C	94	1	−1
1BRS	C	95	0	−1
1BRS	C	96	0	−1
1BRS	C	97	0	−1
1BRS	C	98	1	−1
1BRS	C	99	0	−1
1BRS	C	100	1	−1
1BRS	C	101	1	−1
1BRS	C	102	1	1
1BRS	C	103	0	−1
1BRS	C	104	1	1
1BRS	C	105	1	−1
1BRS	C	106	0	−1
1BRS	C	107	1	−1
1BRS	C	108	1	−1
1BRS	C	109	1	−1
1BRS	D	1	1	−1
1BRS	D	2	1	−1
1BRS	D	3	0	−1
1BRS	D	4	0	−1
1BRS	D	5	0	−1
1BRS	D	6	1	−1
1BRS	D	7	0	−1
1BRS	D	8	1	−1
1BRS	D	9	1	−1
1BRS	D	10	0	−1
1BRS	D	11	1	−1
1BRS	D	12	1	−1

1BRS	D	13	1	−1
1BRS	D	14	1	−1
1BRS	D	15	1	−1
1BRS	D	16	0	−1
1BRS	D	17	0	−1
1BRS	D	18	1	−1
1BRS	D	19	1	−1
1BRS	D	20	0	−1
1BRS	D	21	0	−1
1BRS	D	22	1	−1
1BRS	D	23	1	−1
1BRS	D	24	0	−1
1BRS	D	25	1	−1
1BRS	D	26	0	−1
1BRS	D	27	0	−1
1BRS	D	28	1	−1
1BRS	D	29	1	1
1BRS	D	30	0	−1
1BRS	D	31	1	1
1BRS	D	32	1	1
1BRS	D	33	1	1
1BRS	D	34	1	1
1BRS	D	35	1	1
1BRS	D	36	0	−1
1BRS	D	37	0	−1
1BRS	D	38	1	1
1BRS	D	39	1	1
1BRS	D	40	0	−1
1BRS	D	41	0	−1
1BRS	D	42	1	1
1BRS	D	43	1	1
1BRS	D	44	1	1
1BRS	D	45	0	−1
1BRS	D	46	1	1
1BRS	D	47	1	1
1BRS	D	48	1	−1
1BRS	D	49	0	−1
1BRS	D	50	1	−1
1BRS	D	51	0	−1
1BRS	D	52	0	−1
1BRS	D	53	0	−1
1BRS	D	54	1	−1
1BRS	D	55	1	−1
1BRS	D	56	0	−1
1BRS	D	57	1	−1
1BRS	D	58	1	−1
1BRS	D	59	0	−1
1BRS	D	60	1	−1
1BRS	D	61	1	−1
1BRS	D	62	1	−1
1BRS	D	63	1	−1
1BRS	D	66	1	−1

1BRS	D	67	0	−1
1BRS	D	68	1	−1
1BRS	D	69	1	−1
1BRS	D	70	0	−1
1BRS	D	71	0	−1
1BRS	D	72	1	−1
1BRS	D	73	0	−1
1BRS	D	74	0	−1
1BRS	D	75	1	−1
1BRS	D	76	1	1
1BRS	D	77	0	−1
1BRS	D	78	1	−1
1BRS	D	79	1	−1
1BRS	D	80	1	−1
1BRS	D	81	1	−1
1BRS	D	82	0	−1
1BRS	D	83	1	−1
1BRS	D	84	0	−1
1BRS	D	85	1	−1
1BRS	D	86	0	−1
1BRS	D	87	1	−1
1BRS	D	88	1	−1
1BRS	D	89	1	−1
1BRS	E	2	1	−1
1BRS	E	3	0	−1
1BRS	E	4	1	−1
1BRS	E	5	0	−1
1BRS	E	6	1	−1
1BRS	E	7	0	−1
1BRS	E	8	1	−1
1BRS	E	9	1	−1
1BRS	E	10	0	−1
1BRS	E	11	1	−1
1BRS	E	12	1	−1
1BRS	E	13	1	−1
1BRS	E	14	1	−1
1BRS	E	15	1	−1
1BRS	E	16	0	−1
1BRS	E	17	0	−1
1BRS	E	18	0	−1
1BRS	E	19	1	−1
1BRS	E	20	0	−1
1BRS	E	21	0	−1
1BRS	E	22	1	−1
1BRS	E	23	1	−1
1BRS	E	24	0	−1
1BRS	E	25	1	−1
1BRS	E	26	0	−1
1BRS	E	27	0	−1
1BRS	E	28	1	−1
1BRS	E	29	1	1
1BRS	E	30	0	−1

1BRS	E	31	1	1
1BRS	E	32	1	1
1BRS	E	33	1	1
1BRS	E	34	1	1
1BRS	E	35	1	1
1BRS	E	36	0	−1
1BRS	E	37	0	−1
1BRS	E	38	1	1
1BRS	E	39	1	1
1BRS	E	40	0	−1
1BRS	E	41	0	−1
1BRS	E	42	1	1
1BRS	E	43	1	1
1BRS	E	44	1	1
1BRS	E	45	0	−1
1BRS	E	46	1	1
1BRS	E	47	1	−1
1BRS	E	48	1	−1
1BRS	E	49	0	−1
1BRS	E	50	1	−1
1BRS	E	51	0	−1
1BRS	E	52	0	−1
1BRS	E	53	0	−1
1BRS	E	54	0	−1
1BRS	E	55	1	−1
1BRS	E	56	0	−1
1BRS	E	57	1	−1
1BRS	E	58	0	−1
1BRS	E	59	0	−1
1BRS	E	60	0	−1
1BRS	E	61	1	−1
1BRS	E	62	1	−1
1BRS	E	63	1	−1
1BRS	E	66	1	−1
1BRS	E	67	0	−1
1BRS	E	68	1	−1
1BRS	E	69	1	−1
1BRS	E	70	0	−1
1BRS	E	71	0	−1
1BRS	E	72	1	−1
1BRS	E	73	0	−1
1BRS	E	74	0	−1
1BRS	E	75	1	−1
1BRS	E	76	1	1
1BRS	E	77	0	−1
1BRS	E	78	1	−1
1BRS	E	79	1	−1
1BRS	E	80	1	1
1BRS	E	81	1	−1
1BRS	E	82	0	−1
1BRS	E	83	1	−1
1BRS	E	84	0	−1

1BRS	E	85	1	−1
1BRS	E	86	0	−1
1BRS	E	87	1	−1
1BRS	E	88	1	−1
1BRS	E	89	1	−1
1BRS	F	1	1	−1
1BRS	F	2	1	−1
1BRS	F	3	0	−1
1BRS	F	4	0	−1
1BRS	F	5	0	−1
1BRS	F	6	1	−1
1BRS	F	7	0	−1
1BRS	F	8	1	−1
1BRS	F	9	1	−1
1BRS	F	10	0	−1
1BRS	F	11	1	−1
1BRS	F	12	1	−1
1BRS	F	13	1	−1
1BRS	F	14	1	−1
1BRS	F	15	1	−1
1BRS	F	16	0	−1
1BRS	F	17	0	−1
1BRS	F	18	1	−1
1BRS	F	19	1	−1
1BRS	F	20	0	−1
1BRS	F	21	0	−1
1BRS	F	22	1	−1
1BRS	F	23	1	−1
1BRS	F	24	0	−1
1BRS	F	25	1	−1
1BRS	F	26	0	−1
1BRS	F	27	0	−1
1BRS	F	28	1	−1
1BRS	F	29	1	1
1BRS	F	30	0	−1
1BRS	F	31	1	1
1BRS	F	32	1	1
1BRS	F	33	1	1
1BRS	F	34	1	1
1BRS	F	35	1	1
1BRS	F	36	0	−1
1BRS	F	37	0	−1
1BRS	F	38	1	1
1BRS	F	39	1	1
1BRS	F	40	0	−1
1BRS	F	41	0	−1
1BRS	F	42	1	1
1BRS	F	43	1	1
1BRS	F	44	1	1
1BRS	F	45	0	−1
1BRS	F	46	1	1
1BRS	F	47	1	−1

1BRS	F	48	1	−1
1BRS	F	49	0	−1
1BRS	F	50	1	−1
1BRS	F	51	0	−1
1BRS	F	52	0	−1
1BRS	F	53	0	−1
1BRS	F	54	1	−1
1BRS	F	55	1	−1
1BRS	F	56	0	−1
1BRS	F	57	1	−1
1BRS	F	58	1	−1
1BRS	F	59	0	−1
1BRS	F	60	1	−1
1BRS	F	61	1	−1
1BRS	F	62	1	−1
1BRS	F	63	0	−1
1BRS	F	64	1	−1
1BRS	F	65	1	−1
1BRS	F	66	0	−1
1BRS	F	67	0	−1
1BRS	F	68	1	−1
1BRS	F	69	1	−1
1BRS	F	70	0	−1
1BRS	F	71	0	−1
1BRS	F	72	1	1
1BRS	F	73	0	−1
1BRS	F	74	0	−1
1BRS	F	75	1	−1
1BRS	F	76	1	1
1BRS	F	77	0	−1
1BRS	F	78	1	−1
1BRS	F	79	1	−1
1BRS	F	80	1	−1
1BRS	F	81	1	−1
1BRS	F	82	0	−1
1BRS	F	83	1	−1
1BRS	F	84	0	−1
1BRS	F	85	1	−1
1BRS	F	86	0	−1
1BRS	F	87	1	−1
1BRS	F	88	1	−1
1BTH	P	1	1	−1
1BTH	P	2	1	−1
1BTH	P	3	1	−1
1BTH	P	4	0	−1
1BTH	P	5	0	−1
1BTH	P	6	1	−1
1BTH	P	7	1	−1
1BTH	P	8	1	−1
1BTH	P	9	1	1
1BTH	P	10	1	−1
1BTH	P	11	1	1

1BTH	P	12	1	1
1BTH	P	13	1	1
1BTH	P	14	1	1
1BTH	P	15	1	1
1BTH	P	16	1	1
1BTH	P	17	1	1
1BTH	P	18	1	1
1BTH	P	19	1	1
1BTH	P	20	1	1
1BTH	P	21	1	−1
1BTH	P	22	0	−1
1BTH	P	23	0	−1
1BTH	P	24	1	−1
1BTH	P	25	1	−1
1BTH	P	26	1	−1
1BTH	P	27	1	−1
1BTH	P	28	1	−1
1BTH	P	29	1	−1
1BTH	P	30	0	−1
1BTH	P	31	1	−1
1BTH	P	32	1	1
1BTH	P	33	0	−1
1BTH	P	34	1	1
1BTH	P	35	0	−1
1BTH	P	36	0	−1
1BTH	P	37	1	1
1BTH	P	38	0	−1
1BTH	P	39	1	1
1BTH	P	40	1	1
1BTH	P	41	1	1
1BTH	P	42	1	1
1BTH	P	43	0	−1
1BTH	P	44	0	−1
1BTH	P	45	0	−1
1BTH	P	46	1	1
1BTH	P	47	0	−1
1BTH	P	48	1	−1
1BTH	P	49	1	−1
1BTH	P	50	1	−1
1BTH	P	51	0	−1
1BTH	P	52	1	−1
1BTH	P	53	1	−1
1BTH	P	54	1	−1
1BTH	P	55	0	−1
1BTH	P	56	1	−1
1BTH	P	57	1	−1
1BTH	P	58	1	−1
1BTH	Q	1	1	−1
1BTH	Q	2	1	−1
1BTH	Q	3	1	−1
1BTH	Q	4	0	−1
1BTH	Q	5	0	−1

1BTH	Q	6	1	−1
1BTH	Q	7	1	−1
1BTH	Q	8	1	−1
1BTH	Q	9	1	1
1BTH	Q	10	1	−1
1BTH	Q	11	1	1
1BTH	Q	12	1	1
1BTH	Q	13	1	1
1BTH	Q	14	1	1
1BTH	Q	15	1	1
1BTH	Q	16	1	1
1BTH	Q	17	1	1
1BTH	Q	18	1	1
1BTH	Q	19	1	1
1BTH	Q	20	1	1
1BTH	Q	21	1	−1
1BTH	Q	22	0	−1
1BTH	Q	23	0	−1
1BTH	Q	24	1	−1
1BTH	Q	25	1	−1
1BTH	Q	26	1	−1
1BTH	Q	27	1	−1
1BTH	Q	28	1	−1
1BTH	Q	29	1	−1
1BTH	Q	30	0	−1
1BTH	Q	31	1	−1
1BTH	Q	32	1	1
1BTH	Q	33	0	−1
1BTH	Q	34	1	1
1BTH	Q	35	0	−1
1BTH	Q	36	0	−1
1BTH	Q	37	1	1
1BTH	Q	38	0	−1
1BTH	Q	39	1	1
1BTH	Q	40	1	1
1BTH	Q	41	1	1
1BTH	Q	42	1	1
1BTH	Q	43	0	−1
1BTH	Q	44	0	−1
1BTH	Q	45	0	−1
1BTH	Q	46	1	1
1BTH	Q	47	0	−1
1BTH	Q	48	1	−1
1BTH	Q	49	1	−1
1BTH	Q	50	1	−1
1BTH	Q	51	0	−1
1BTH	Q	52	1	−1
1BTH	Q	53	1	−1
1BTH	Q	54	1	−1
1BTH	Q	55	0	−1
1BTH	Q	56	1	−1
1BTH	Q	57	1	−1

1BTH	Q	58	1	−1
1CHO	I	4	1	−1
1CHO	I	5	1	−1
1CHO	I	6	1	−1
1CHO	I	7	1	−1
1CHO	I	8	0	−1
1CHO	I	9	1	−1
1CHO	I	10	1	−1
1CHO	I	11	0	−1
1CHO	I	12	1	−1
1CHO	I	13	1	1
1CHO	I	14	1	1
1CHO	I	15	1	1
1CHO	I	16	1	1
1CHO	I	17	1	1
1CHO	I	18	1	1
1CHO	I	19	1	1
1CHO	I	20	1	1
1CHO	I	21	1	1
1CHO	I	22	0	−1
1CHO	I	23	0	−1
1CHO	I	24	0	−1
1CHO	I	25	0	−1
1CHO	I	26	1	−1
1CHO	I	27	1	−1
1CHO	I	28	1	−1
1CHO	I	29	1	−1
1CHO	I	30	1	−1
1CHO	I	31	0	−1
1CHO	I	32	0	−1
1CHO	I	33	0	−1
1CHO	I	34	1	−1
1CHO	I	35	0	−1
1CHO	I	36	1	1
1CHO	I	37	0	−1
1CHO	I	38	0	−1
1CHO	I	39	0	−1
1CHO	I	40	0	−1
1CHO	I	41	0	−1
1CHO	I	42	1	−1
1CHO	I	43	1	−1
1CHO	I	44	0	−1
1CHO	I	45	1	−1
1CHO	I	46	1	−1
1CHO	I	47	1	−1
1CHO	I	48	0	−1
1CHO	I	49	1	−1
1CHO	I	50	1	−1
1CHO	I	51	1	−1
1CHO	I	52	1	−1
1CHO	I	53	1	−1
1CHO	I	54	1	−1

1CHO	I	55	1	1
1CHO	I	56	1	−1
1DAN	L	1	1	−1
1DAN	L	2	1	−1
1DAN	L	3	1	−1
1DAN	L	4	1	−1
1DAN	L	5	1	−1
1DAN	L	8	1	−1
1DAN	L	9	1	−1
1DAN	L	10	1	−1
1DAN	L	11	1	−1
1DAN	L	12	1	−1
1DAN	L	13	1	1
1DAN	L	15	0	−1
1DAN	L	17	0	−1
1DAN	L	18	1	1
1DAN	L	21	1	−1
1DAN	L	22	0	−1
1DAN	L	23	1	−1
1DAN	L	24	1	−1
1DAN	L	27	0	−1
1DAN	L	28	1	−1
1DAN	L	30	1	−1
1DAN	L	31	1	1
1DAN	L	32	1	−1
1DAN	L	33	1	−1
1DAN	L	34	1	−1
1DAN	L	36	1	1
1DAN	L	37	0	−1
1DAN	L	38	1	−1
1DAN	L	39	1	1
1DAN	L	40	0	−1
1DAN	L	41	1	−1
1DAN	L	42	1	1
1DAN	L	43	1	1
1DAN	L	44	1	−1
1DAN	L	45	1	−1
1DAN	L	46	0	−1
1DAN	L	47	1	−1
1DAN	L	48	1	−1
1DAN	L	49	1	−1
1DAN	L	50	0	−1
1DAN	L	51	1	−1
1DAN	L	52	1	−1
1DAN	L	53	1	−1
1DAN	L	54	0	−1
1DAN	L	55	0	−1
1DAN	L	56	1	−1
1DAN	L	57	1	−1
1DAN	L	58	1	−1
1DAN	L	59	0	−1
1DAN	L	60	1	1

1DAN	L	61	1	−1
1DAN	L	62	1	1
1DAN	L	63	1	1
1DAN	L	64	1	1
1DAN	L	65	1	1
1DAN	L	66	1	−1
1DAN	L	67	1	1
1DAN	L	68	1	−1
1DAN	L	69	1	1
1DAN	L	70	0	−1
1DAN	L	71	1	1
1DAN	L	72	1	1
1DAN	L	73	1	−1
1DAN	L	74	1	1
1DAN	L	75	1	−1
1DAN	L	76	0	−1
1DAN	L	77	1	1
1DAN	L	78	1	1
1DAN	L	79	1	1
1DAN	L	80	0	−1
1DAN	L	81	0	−1
1DAN	L	82	1	1
1DAN	L	83	1	−1
1DAN	L	84	1	−1
1DAN	L	85	1	1
1DAN	L	86	1	−1
1DAN	L	87	1	−1
1DAN	L	88	1	1
1DAN	L	89	1	−1
1DAN	L	90	1	1
1DAN	L	91	0	−1
1DAN	L	92	1	1
1DAN	L	93	1	1
1DAN	L	94	1	1
1DAN	L	95	0	−1
1DAN	L	96	0	−1
1DAN	L	97	1	−1
1DAN	L	98	0	−1
1DAN	L	99	0	−1
1DAN	L	100	0	−1
1DAN	L	101	0	−1
1DAN	L	102	0	−1
1DAN	L	103	0	−1
1DAN	L	104	1	1
1DAN	L	105	1	−1
1DAN	L	106	1	−1
1DAN	L	107	1	−1
1DAN	L	108	1	−1
1DAN	L	109	1	−1
1DAN	L	110	0	−1
1DAN	L	111	1	−1
1DAN	L	112	0	−1

1DAN	L	113	1	−1
1DAN	L	114	0	−1
1DAN	L	115	0	−1
1DAN	L	116	1	−1
1DAN	L	117	0	−1
1DAN	L	118	0	−1
1DAN	L	119	1	−1
1DAN	L	120	1	−1
1DAN	L	121	1	−1
1DAN	L	122	1	−1
1DAN	L	123	1	−1
1DAN	L	124	1	−1
1DAN	L	125	0	−1
1DAN	L	126	0	−1
1DAN	L	127	0	−1
1DAN	L	128	1	−1
1DAN	L	129	1	−1
1DAN	L	130	1	−1
1DAN	L	131	1	−1
1DAN	L	132	1	−1
1DAN	L	133	0	−1
1DAN	L	134	0	−1
1DAN	L	135	0	−1
1DAN	L	136	0	−1
1DAN	L	137	0	−1
1DAN	L	138	0	−1
1DAN	L	139	0	−1
1DAN	L	140	1	−1
1DAN	L	141	1	−1
1DAN	L	142	1	−1
1DAN	H	1	0	−1
1DAN	H	2	0	−1
1DAN	H	3	1	−1
1DAN	H	4	0	−1
1DAN	H	5	1	−1
1DAN	H	6	1	−1
1DAN	H	7	0	−1
1DAN	H	8	1	−1
1DAN	H	9	1	−1
1DAN	H	10	0	−1
1DAN	H	11	1	−1
1DAN	H	12	0	−1
1DAN	H	13	0	−1
1DAN	H	14	0	−1
1DAN	H	15	0	−1
1DAN	H	16	0	−1
1DAN	H	17	0	−1
1DAN	H	18	0	−1
1DAN	H	19	0	−1
1DAN	H	20	0	−1
1DAN	H	21	1	−1
1DAN	H	22	1	−1

1DAN	H	23	1	−1
1DAN	H	24	1	−1
1DAN	H	25	1	−1
1DAN	H	26	0	−1
1DAN	H	27	0	−1
1DAN	H	28	0	−1
1DAN	H	29	0	−1
1DAN	H	30	0	−1
1DAN	H	31	0	−1
1DAN	H	32	1	−1
1DAN	H	33	1	−1
1DAN	H	34	0	−1
1DAN	H	35	0	−1
1DAN	H	36	0	−1
1DAN	H	37	0	−1
1DAN	H	38	0	−1
1DAN	H	39	0	−1
1DAN	H	40	0	−1
1DAN	H	41	1	1
1DAN	H	42	0	−1
1DAN	H	43	0	−1
1DAN	H	44	1	−1
1DAN	H	45	1	−1
1DAN	H	46	0	−1
1DAN	H	47	1	−1
1DAN	H	48	1	−1
1DAN	H	49	0	−1
1DAN	H	50	1	−1
1DAN	H	51	1	−1
1DAN	H	52	0	−1
1DAN	H	53	1	−1
1DAN	H	54	0	−1
1DAN	H	55	0	−1
1DAN	H	56	0	−1
1DAN	H	57	0	−1
1DAN	H	58	0	−1
1DAN	H	59	0	−1
1DAN	H	60	0	−1
1DAN	H	61	1	−1
1DAN	H	62	1	−1
1DAN	H	63	1	−1
1DAN	H	64	1	−1
1DAN	H	65	1	−1
1DAN	H	66	1	−1
1DAN	H	67	1	−1
1DAN	H	68	0	−1
1DAN	H	69	0	−1
1DAN	H	70	1	−1
1DAN	H	71	0	−1
1DAN	H	72	1	−1
1DAN	H	73	0	−1
1DAN	H	74	1	−1

1DAN	H	75	0	−1
1DAN	H	76	0	−1
1DAN	H	77	0	−1
1DAN	H	78	0	−1
1DAN	H	79	0	−1
1DAN	H	80	1	−1
1DAN	H	81	1	−1
1DAN	H	82	0	−1
1DAN	H	83	1	−1
1DAN	H	84	1	−1
1DAN	H	85	1	1
1DAN	H	86	1	1
1DAN	H	87	0	−1
1DAN	H	88	1	−1
1DAN	H	89	0	−1
1DAN	H	90	0	−1
1DAN	H	91	0	−1
1DAN	H	92	0	−1
1DAN	H	93	0	−1
1DAN	H	94	0	−1
1DAN	H	95	0	−1
1DAN	H	96	0	−1
1DAN	H	97	1	−1
1DAN	H	98	1	−1
1DAN	H	99	1	−1
1DAN	H	100	0	−1
1DAN	H	101	1	−1
1DAN	H	102	1	−1
1DAN	H	103	1	−1
1DAN	H	104	1	−1
1DAN	H	105	0	−1
1DAN	H	106	0	−1
1DAN	H	107	0	−1
1DAN	H	108	0	−1
1DAN	H	109	0	−1
1DAN	H	110	0	−1
1DAN	H	111	0	−1
1DAN	H	112	0	−1
1DAN	H	113	1	−1
1DAN	H	114	1	−1
1DAN	H	115	1	−1
1DAN	H	116	0	−1
1DAN	H	117	0	−1
1DAN	H	118	1	−1
1DAN	H	119	1	1
1DAN	H	120	0	−1
1DAN	H	121	0	−1
1DAN	H	122	0	−1
1DAN	H	123	1	1
1DAN	H	124	0	−1
1DAN	H	125	1	1
1DAN	H	126	1	1

1DAN	H	127	0	−1
1DAN	H	128	1	−1
1DAN	H	129	0	−1
1DAN	H	130	0	−1
1DAN	H	131	0	−1
1DAN	H	132	0	−1
1DAN	H	133	0	−1
1DAN	H	134	0	−1
1DAN	H	135	1	−1
1DAN	H	136	1	−1
1DAN	H	137	1	−1
1DAN	H	138	1	−1
1DAN	H	139	1	−1
1DAN	H	140	1	−1
1DAN	H	141	1	−1
1DAN	H	142	0	−1
1DAN	H	143	1	−1
1DAN	H	144	1	−1
1DAN	H	145	0	−1
1DAN	H	146	0	−1
1DAN	H	147	0	−1
1DAN	H	148	0	−1
1DAN	H	149	1	−1
1DAN	H	150	0	−1
1DAN	H	151	0	−1
1DAN	H	152	0	−1
1DAN	H	153	0	−1
1DAN	H	154	1	1
1DAN	H	155	0	−1
1DAN	H	156	1	1
1DAN	H	157	1	1
1DAN	H	158	0	−1
1DAN	H	159	1	−1
1DAN	H	160	1	1
1DAN	H	161	1	−1
1DAN	H	162	1	−1
1DAN	H	163	1	−1
1DAN	H	164	1	−1
1DAN	H	165	1	−1
1DAN	H	166	1	−1
1DAN	H	167	1	−1
1DAN	H	168	1	−1
1DAN	H	169	1	−1
1DAN	H	170	1	−1
1DAN	H	171	0	−1
1DAN	H	172	0	−1
1DAN	H	173	1	1
1DAN	H	174	0	−1
1DAN	H	175	0	−1
1DAN	H	176	0	−1
1DAN	H	177	0	−1
1DAN	H	178	0	−1

1DAN	H	179	0	−1
1DAN	H	180	1	−1
1DAN	H	181	1	−1
1DAN	H	182	1	−1
1DAN	H	183	1	−1
1DAN	H	184	1	−1
1DAN	H	185	1	−1
1DAN	H	186	0	−1
1DAN	H	187	0	−1
1DAN	H	188	0	−1
1DAN	H	189	1	1
1DAN	H	190	1	1
1DAN	H	191	0	−1
1DAN	H	192	0	−1
1DAN	H	193	0	−1
1DAN	H	194	0	−1
1DAN	H	195	0	−1
1DAN	H	196	0	−1
1DAN	H	197	0	−1
1DAN	H	198	0	−1
1DAN	H	199	1	−1
1DAN	H	200	0	−1
1DAN	H	201	1	−1
1DAN	H	202	1	−1
1DAN	H	203	0	−1
1DAN	H	204	0	−1
1DAN	H	205	0	−1
1DAN	H	206	0	−1
1DAN	H	207	0	−1
1DAN	H	208	0	−1
1DAN	H	209	0	−1
1DAN	H	210	0	−1
1DAN	H	211	0	−1
1DAN	H	212	0	−1
1DAN	H	213	0	−1
1DAN	H	214	1	−1
1DAN	H	215	0	−1
1DAN	H	216	0	−1
1DAN	H	217	0	−1
1DAN	H	218	1	−1
1DAN	H	219	1	−1
1DAN	H	220	0	−1
1DAN	H	221	0	−1
1DAN	H	222	0	−1
1DAN	H	223	0	−1
1DAN	H	224	0	−1
1DAN	H	225	0	−1
1DAN	H	226	0	−1
1DAN	H	227	0	−1
1DAN	H	228	0	−1
1DAN	H	229	0	−1
1DAN	H	230	0	−1

1DAN	H	231	0	−1
1DAN	H	232	0	−1
1DAN	H	233	1	−1
1DAN	H	234	0	−1
1DAN	H	235	0	−1
1DAN	H	236	1	−1
1DAN	H	237	1	−1
1DAN	H	238	0	−1
1DAN	H	239	0	−1
1DAN	H	240	1	−1
1DAN	H	241	1	−1
1DAN	H	242	1	−1
1DAN	H	243	1	−1
1DAN	H	244	1	−1
1DAN	H	245	1	−1
1DAN	H	246	1	−1
1DAN	H	247	1	−1
1DAN	H	248	1	−1
1DAN	H	249	1	−1
1DAN	H	250	1	−1
1DAN	H	251	0	−1
1DAN	H	252	1	−1
1DAN	H	253	0	−1
1DAN	H	254	1	−1
1DAN	T	2	1	−1
1DAN	T	3	1	−1
1DAN	T	4	1	−1
1DAN	T	5	0	−1
1DAN	T	6	1	−1
1DAN	T	7	1	−1
1DAN	T	8	0	−1
1DAN	T	9	1	−1
1DAN	T	10	0	−1
1DAN	T	11	1	1
1DAN	T	12	0	−1
1DAN	T	13	1	1
1DAN	T	14	0	−1
1DAN	T	15	0	−1
1DAN	T	16	1	1
1DAN	T	17	0	−1
1DAN	T	18	0	−1
1DAN	T	19	0	−1
1DAN	T	20	1	1
1DAN	T	21	0	−1
1DAN	T	22	1	−1
1DAN	T	23	0	−1
1DAN	T	24	1	−1
1DAN	T	25	1	−1
1DAN	T	26	1	−1
1DAN	T	27	1	−1
1DAN	T	28	0	−1
1DAN	T	29	0	−1

1DAN	T	30	0	−1
1DAN	T	31	0	−1
1DAN	T	32	0	−1
1DAN	T	33	0	−1
1DAN	T	34	0	−1
1DAN	T	35	0	−1
1DAN	T	36	0	−1
1DAN	T	37	1	1
1DAN	T	38	1	1
1DAN	T	39	1	1
1DAN	T	40	1	1
1DAN	T	41	1	1
1DAN	T	42	1	1
1DAN	T	43	1	1
1DAN	T	44	0	−1
1DAN	T	45	1	1
1DAN	T	46	1	1
1DAN	T	47	1	1
1DAN	T	48	0	−1
1DAN	T	49	1	−1
1DAN	T	50	1	1
1DAN	T	51	1	−1
1DAN	T	52	1	1
1DAN	T	53	0	−1
1DAN	T	54	1	1
1DAN	T	55	0	−1
1DAN	T	56	0	−1
1DAN	T	57	1	1
1DAN	T	58	0	−1
1DAN	T	59	0	−1
1DAN	T	60	0	−1
1DAN	T	61	1	−1
1DAN	T	62	1	−1
1DAN	T	63	0	−1
1DAN	T	64	1	−1
1DAN	T	65	1	−1
1DAN	T	66	0	−1
1DAN	T	67	0	−1
1DAN	T	68	0	−1
1DAN	T	69	0	−1
1DAN	T	70	0	−1
1DAN	T	71	0	−1
1DAN	T	72	0	−1
1DAN	T	73	0	−1
1DAN	T	74	1	1
1DAN	T	75	1	−1
1DAN	T	76	1	−1
1DAN	U	2	1	1
1DAN	U	3	1	1
1DAN	U	4	1	1
1DAN	U	5	1	1
1DAN	U	6	1	1

1DAN	U	7	1	1
1DAN	U	8	0	−1
1DAN	U	9	1	−1
1DAN	U	10	1	−1
1DAN	U	11	0	−1
1DAN	U	12	0	−1
1DAN	U	13	0	−1
1DAN	U	14	0	−1
1DAN	U	15	1	−1
1DAN	U	16	1	−1
1DAN	U	17	0	−1
1DAN	U	18	1	−1
1DAN	U	19	0	−1
1DAN	U	20	0	−1
1DAN	U	21	1	1
1DAN	U	22	0	−1
1DAN	U	23	1	1
1DAN	U	24	0	−1
1DAN	U	25	1	−1
1DAN	U	26	1	−1
1DAN	U	27	1	−1
1DAN	U	28	1	−1
1DAN	U	29	1	−1
1DAN	U	30	1	−1
1DAN	U	31	1	−1
1DAN	U	32	1	−1
1DAN	U	33	1	−1
1DAN	U	34	0	−1
1DAN	U	35	0	−1
1DAN	U	36	0	−1
1DAN	U	37	0	−1
1DAN	U	38	0	−1
1DAN	U	39	1	1
1DAN	U	40	1	−1
1DAN	U	41	1	1
1DAN	U	42	1	1
1DAN	U	43	0	−1
1DAN	U	44	1	1
1DAN	U	45	0	−1
1DAN	U	46	1	1
1DAN	U	47	1	−1
1DAN	U	48	1	−1
1DAN	U	49	1	1
1DAN	U	50	1	−1
1DAN	U	51	1	1
1DAN	U	52	0	−1
1DAN	U	53	0	−1
1DAN	U	54	0	−1
1DAN	U	55	0	−1
1DAN	U	56	1	−1
1DAN	U	57	0	−1
1DAN	U	58	0	−1

1DAN	U	59	1	−1
1DAN	U	60	1	−1
1DAN	U	61	1	−1
1DAN	U	62	0	−1
1DAN	U	63	0	−1
1DAN	U	64	0	−1
1DAN	U	65	0	−1
1DAN	U	66	0	−1
1DAN	U	67	0	−1
1DAN	U	68	0	−1
1DAN	U	69	1	1
1DAN	U	74	1	1
1DAN	U	75	1	1
1DAN	U	76	1	1
1DAN	U	77	1	−1
1DAN	U	78	1	−1
1DAN	U	79	0	−1
1DAN	U	80	1	−1
1DAN	U	81	0	−1
1DAN	U	82	1	−1
1DAN	U	83	1	−1
1DAN	U	84	0	−1
1DAN	U	85	1	−1
1DAN	U	86	0	−1
1DAN	U	87	1	−1
1DAN	U	88	0	−1
1DAN	U	89	1	−1
1DAN	U	90	0	−1
1DAN	U	91	1	−1
1DAN	U	92	1	−1
1DAN	U	93	1	−1
1DAN	U	94	1	−1
1DAN	U	95	1	−1
1DAN	U	96	0	−1
1DAN	U	97	0	−1
1DAN	U	98	0	−1
1DAN	U	99	0	−1
1DAN	U	100	0	−1
1DAN	U	101	0	−1
1DAN	U	102	0	−1
1DAN	U	103	0	−1
1DAN	U	104	0	−1
1DAN	U	105	1	−1
1DAN	U	106	1	−1
1DAN	U	107	0	−1
1DAN	U	108	1	−1
1DAN	U	109	1	−1
1DAN	U	110	1	−1
1DAN	U	111	1	−1
1DAN	U	112	1	−1
1DAN	U	113	0	−1
1DAN	U	114	1	1

1DAN	U	115	1	1
1DAN	U	116	0	−1
1DAN	U	117	1	1
1DAN	U	118	1	1
1DAN	U	119	1	1
1DAN	U	120	1	1
1DAN	U	121	1	−1
1DFJ	E	1	1	−1
1DFJ	E	2	1	1
1DFJ	E	3	1	−1
1DFJ	E	4	1	1
1DFJ	E	5	0	−1
1DFJ	E	6	0	−1
1DFJ	E	7	1	1
1DFJ	E	8	0	−1
1DFJ	E	9	1	−1
1DFJ	E	10	1	1
1DFJ	E	11	0	−1
1DFJ	E	12	0	−1
1DFJ	E	13	0	−1
1DFJ	E	14	0	−1
1DFJ	E	15	1	−1
1DFJ	E	16	1	−1
1DFJ	E	17	0	−1
1DFJ	E	18	1	−1
1DFJ	E	19	1	−1
1DFJ	E	20	0	−1
1DFJ	E	21	1	−1
1DFJ	E	22	1	−1
1DFJ	E	23	1	1
1DFJ	E	24	1	1
1DFJ	E	25	0	−1
1DFJ	E	26	0	−1
1DFJ	E	27	1	1
1DFJ	E	28	1	1
1DFJ	E	29	0	−1
1DFJ	E	30	0	−1
1DFJ	E	31	1	1
1DFJ	E	32	1	1
1DFJ	E	33	0	−1
1DFJ	E	34	1	1
1DFJ	E	35	0	−1
1DFJ	E	36	0	−1
1DFJ	E	37	1	1
1DFJ	E	38	1	1
1DFJ	E	39	1	1
1DFJ	E	40	0	−1
1DFJ	E	41	0	−1
1DFJ	E	42	1	1
1DFJ	E	43	1	1
1DFJ	E	44	0	−1
1DFJ	E	45	0	−1

1DFJ	E	46	0	−1
1DFJ	E	47	0	−1
1DFJ	E	48	0	−1
1DFJ	E	49	1	−1
1DFJ	E	50	1	−1
1DFJ	E	51	1	−1
1DFJ	E	52	1	−1
1DFJ	E	53	1	−1
1DFJ	E	54	0	−1
1DFJ	E	55	1	−1
1DFJ	E	56	1	−1
1DFJ	E	57	0	−1
1DFJ	E	58	0	−1
1DFJ	E	59	1	−1
1DFJ	E	60	0	−1
1DFJ	E	61	1	−1
1DFJ	E	62	1	−1
1DFJ	E	63	1	−1
1DFJ	E	64	1	−1
1DFJ	E	65	0	−1
1DFJ	E	66	1	1
1DFJ	E	67	1	1
1DFJ	E	68	1	−1
1DFJ	E	69	1	1
1DFJ	E	70	1	−1
1DFJ	E	71	1	1
1DFJ	E	72	0	−1
1DFJ	E	73	0	−1
1DFJ	E	74	0	−1
1DFJ	E	75	0	−1
1DFJ	E	76	1	−1
1DFJ	E	77	1	−1
1DFJ	E	78	1	−1
1DFJ	E	79	0	−1
1DFJ	E	80	0	−1
1DFJ	E	81	0	−1
1DFJ	E	82	0	−1
1DFJ	E	83	0	−1
1DFJ	E	84	0	−1
1DFJ	E	85	1	−1
1DFJ	E	86	1	1
1DFJ	E	87	1	1
1DFJ	E	88	1	1
1DFJ	E	89	1	1
1DFJ	E	90	0	−1
1DFJ	E	91	1	1
1DFJ	E	92	1	−1
1DFJ	E	93	1	−1
1DFJ	E	94	1	1
1DFJ	E	95	0	−1
1DFJ	E	96	0	−1
1DFJ	E	97	0	−1

1DFJ	E	98	1	−1
1DFJ	E	99	0	−1
1DFJ	E	100	1	−1
1DFJ	E	101	1	−1
1DFJ	E	102	1	−1
1DFJ	E	103	1	−1
1DFJ	E	104	1	−1
1DFJ	E	105	1	−1
1DFJ	E	106	0	−1
1DFJ	E	107	0	−1
1DFJ	E	108	0	−1
1DFJ	E	109	0	−1
1DFJ	E	110	0	−1
1DFJ	E	111	1	1
1DFJ	E	112	1	−1
1DFJ	E	113	1	−1
1DFJ	E	114	1	−1
1DFJ	E	115	1	−1
1DFJ	E	116	0	−1
1DFJ	E	117	0	−1
1DFJ	E	118	0	−1
1DFJ	E	119	1	1
1DFJ	E	120	0	−1
1DFJ	E	121	0	−1
1DFJ	E	122	0	−1
1DFJ	E	123	0	−1
1DFJ	E	124	1	−1
1DFJ	I	2	1	−1
1DFJ	I	3	1	−1
1DFJ	I	4	0	−1
1DFJ	I	5	1	−1
1DFJ	I	6	0	−1
1DFJ	I	7	1	1
1DFJ	I	8	1	1
1DFJ	I	9	1	−1
1DFJ	I	10	1	−1
1DFJ	I	11	0	−1
1DFJ	I	12	1	−1
1DFJ	I	13	1	−1
1DFJ	I	14	1	−1
1DFJ	I	15	1	−1
1DFJ	I	16	0	−1
1DFJ	I	17	1	−1
1DFJ	I	18	1	−1
1DFJ	I	19	0	−1
1DFJ	I	20	0	−1
1DFJ	I	21	1	−1
1DFJ	I	22	1	−1
1DFJ	I	23	0	−1
1DFJ	I	24	1	−1
1DFJ	I	25	1	−1
1DFJ	I	26	0	−1

1DFJ	I	27	1	−1
1DFJ	I	28	0	−1
1DFJ	I	29	0	−1
1DFJ	I	30	1	−1
1DFJ	I	31	0	−1
1DFJ	I	32	0	−1
1DFJ	I	33	1	1
1DFJ	I	34	0	−1
1DFJ	I	35	1	−1
1DFJ	I	36	0	−1
1DFJ	I	37	0	−1
1DFJ	I	38	1	−1
1DFJ	I	39	1	−1
1DFJ	I	40	0	−1
1DFJ	I	41	0	−1
1DFJ	I	42	1	−1
1DFJ	I	43	1	−1
1DFJ	I	44	0	−1
1DFJ	I	45	0	−1
1DFJ	I	46	1	−1
1DFJ	I	47	0	−1
1DFJ	I	48	0	−1
1DFJ	I	49	1	−1
1DFJ	I	50	1	−1
1DFJ	I	51	0	−1
1DFJ	I	52	1	−1
1DFJ	I	53	1	−1
1DFJ	I	54	0	−1
1DFJ	I	55	1	−1
1DFJ	I	56	0	−1
1DFJ	I	57	0	−1
1DFJ	I	58	0	−1
1DFJ	I	59	0	−1
1DFJ	I	60	1	1
1DFJ	I	61	1	1
1DFJ	I	62	0	−1
1DFJ	I	63	1	−1
1DFJ	I	64	0	−1
1DFJ	I	65	0	−1
1DFJ	I	66	1	−1
1DFJ	I	67	1	−1
1DFJ	I	68	0	−1
1DFJ	I	69	0	−1
1DFJ	I	70	1	−1
1DFJ	I	71	1	−1
1DFJ	I	72	0	−1
1DFJ	I	73	0	−1
1DFJ	I	74	1	−1
1DFJ	I	75	0	−1
1DFJ	I	76	0	−1
1DFJ	I	77	1	−1
1DFJ	I	78	0	−1

1DFJ	I	79	1	−1
1DFJ	I	80	1	−1
1DFJ	I	81	0	−1
1DFJ	I	82	1	−1
1DFJ	I	83	0	−1
1DFJ	I	84	1	−1
1DFJ	I	85	1	−1
1DFJ	I	86	0	−1
1DFJ	I	87	0	−1
1DFJ	I	88	0	−1
1DFJ	I	89	0	−1
1DFJ	I	90	1	1
1DFJ	I	91	0	−1
1DFJ	I	92	1	−1
1DFJ	I	93	0	−1
1DFJ	I	94	1	−1
1DFJ	I	95	1	−1
1DFJ	I	96	1	−1
1DFJ	I	97	0	−1
1DFJ	I	98	0	−1
1DFJ	I	99	1	−1
1DFJ	I	100	0	−1
1DFJ	I	101	0	−1
1DFJ	I	102	0	−1
1DFJ	I	103	1	−1
1DFJ	I	104	0	−1
1DFJ	I	105	0	−1
1DFJ	I	106	1	−1
1DFJ	I	107	1	−1
1DFJ	I	108	0	−1
1DFJ	I	109	1	−1
1DFJ	I	110	1	−1
1DFJ	I	111	0	−1
1DFJ	I	112	1	−1
1DFJ	I	113	0	−1
1DFJ	I	114	0	−1
1DFJ	I	115	0	−1
1DFJ	I	116	0	−1
1DFJ	I	117	0	−1
1DFJ	I	118	1	1
1DFJ	I	119	0	−1
1DFJ	I	120	1	−1
1DFJ	I	121	0	−1
1DFJ	I	122	0	−1
1DFJ	I	123	0	−1
1DFJ	I	124	1	−1
1DFJ	I	125	0	−1
1DFJ	I	126	0	−1
1DFJ	I	127	1	−1
1DFJ	I	128	1	−1
1DFJ	I	129	0	−1
1DFJ	I	130	0	−1

1DFJ	I	131	1	−1
1DFJ	I	132	0	−1
1DFJ	I	133	0	−1
1DFJ	I	134	1	−1
1DFJ	I	135	0	−1
1DFJ	I	136	1	−1
1DFJ	I	137	1	−1
1DFJ	I	138	0	−1
1DFJ	I	139	1	−1
1DFJ	I	140	0	−1
1DFJ	I	141	1	−1
1DFJ	I	142	0	−1
1DFJ	I	143	0	−1
1DFJ	I	144	0	−1
1DFJ	I	145	0	−1
1DFJ	I	146	1	−1
1DFJ	I	147	1	1
1DFJ	I	148	0	−1
1DFJ	I	149	1	−1
1DFJ	I	150	0	−1
1DFJ	I	151	1	−1
1DFJ	I	152	1	−1
1DFJ	I	153	1	−1
1DFJ	I	154	0	−1
1DFJ	I	155	0	−1
1DFJ	I	156	1	−1
1DFJ	I	157	0	−1
1DFJ	I	158	0	−1
1DFJ	I	159	0	−1
1DFJ	I	160	1	−1
1DFJ	I	161	0	−1
1DFJ	I	162	0	−1
1DFJ	I	163	1	−1
1DFJ	I	164	1	−1
1DFJ	I	165	0	−1
1DFJ	I	166	1	−1
1DFJ	I	167	1	−1
1DFJ	I	168	0	−1
1DFJ	I	169	1	−1
1DFJ	I	170	0	−1
1DFJ	I	171	0	−1
1DFJ	I	172	0	−1
1DFJ	I	173	0	−1
1DFJ	I	174	0	−1
1DFJ	I	175	0	−1
1DFJ	I	176	0	−1
1DFJ	I	177	1	−1
1DFJ	I	178	0	−1
1DFJ	I	179	0	−1
1DFJ	I	180	1	−1
1DFJ	I	181	1	−1
1DFJ	I	182	0	−1

1DFJ	I	183	0	−1
1DFJ	I	184	1	−1
1DFJ	I	185	1	−1
1DFJ	I	186	0	−1
1DFJ	I	187	0	−1
1DFJ	I	188	1	−1
1DFJ	I	189	0	−1
1DFJ	I	190	0	−1
1DFJ	I	191	1	−1
1DFJ	I	192	1	−1
1DFJ	I	193	0	−1
1DFJ	I	194	1	−1
1DFJ	I	195	0	−1
1DFJ	I	196	1	−1
1DFJ	I	197	0	−1
1DFJ	I	198	1	−1
1DFJ	I	199	0	−1
1DFJ	I	200	0	−1
1DFJ	I	201	0	−1
1DFJ	I	202	0	−1
1DFJ	I	203	1	1
1DFJ	I	204	1	−1
1DFJ	I	205	0	−1
1DFJ	I	206	1	−1
1DFJ	I	207	0	−1
1DFJ	I	208	1	−1
1DFJ	I	209	1	−1
1DFJ	I	210	1	−1
1DFJ	I	211	0	−1
1DFJ	I	212	0	−1
1DFJ	I	213	1	−1
1DFJ	I	214	1	−1
1DFJ	I	215	0	−1
1DFJ	I	216	0	−1
1DFJ	I	217	1	−1
1DFJ	I	218	0	−1
1DFJ	I	219	0	−1
1DFJ	I	220	0	−1
1DFJ	I	221	1	−1
1DFJ	I	222	0	−1
1DFJ	I	223	0	−1
1DFJ	I	224	0	−1
1DFJ	I	225	0	−1
1DFJ	I	226	1	−1
1DFJ	I	227	0	−1
1DFJ	I	228	0	−1
1DFJ	I	229	0	−1
1DFJ	I	230	0	−1
1DFJ	I	231	0	−1
1DFJ	I	232	0	−1
1DFJ	I	233	0	−1
1DFJ	I	234	1	−1

1DFJ	I	235	0	−1
1DFJ	I	236	0	−1
1DFJ	I	237	1	−1
1DFJ	I	238	1	−1
1DFJ	I	239	0	−1
1DFJ	I	240	0	−1
1DFJ	I	241	1	−1
1DFJ	I	242	1	−1
1DFJ	I	243	0	−1
1DFJ	I	244	0	−1
1DFJ	I	245	1	−1
1DFJ	I	246	0	−1
1DFJ	I	247	0	−1
1DFJ	I	248	1	−1
1DFJ	I	249	0	−1
1DFJ	I	250	1	−1
1DFJ	I	251	1	−1
1DFJ	I	252	0	−1
1DFJ	I	253	1	−1
1DFJ	I	254	0	−1
1DFJ	I	255	1	−1
1DFJ	I	256	0	−1
1DFJ	I	257	0	−1
1DFJ	I	258	0	−1
1DFJ	I	259	0	−1
1DFJ	I	260	0	−1
1DFJ	I	261	1	1
1DFJ	I	262	0	−1
1DFJ	I	263	1	−1
1DFJ	I	264	0	−1
1DFJ	I	265	1	−1
1DFJ	I	266	1	−1
1DFJ	I	267	1	−1
1DFJ	I	268	0	−1
1DFJ	I	269	0	−1
1DFJ	I	270	1	−1
1DFJ	I	271	0	−1
1DFJ	I	272	0	−1
1DFJ	I	273	0	−1
1DFJ	I	274	1	−1
1DFJ	I	275	0	−1
1DFJ	I	276	0	−1
1DFJ	I	277	1	−1
1DFJ	I	278	1	−1
1DFJ	I	279	0	−1
1DFJ	I	280	1	−1
1DFJ	I	281	1	−1
1DFJ	I	282	0	−1
1DFJ	I	283	1	−1
1DFJ	I	284	1	1
1DFJ	I	285	0	−1
1DFJ	I	286	0	−1

1DFJ	I	287	0	−1
1DFJ	I	288	0	−1
1DFJ	I	289	0	−1
1DFJ	I	290	0	−1
1DFJ	I	291	1	−1
1DFJ	I	292	0	−1
1DFJ	I	293	0	−1
1DFJ	I	294	1	−1
1DFJ	I	295	1	−1
1DFJ	I	296	0	−1
1DFJ	I	297	0	−1
1DFJ	I	298	1	−1
1DFJ	I	299	0	−1
1DFJ	I	300	0	−1
1DFJ	I	301	0	−1
1DFJ	I	302	1	−1
1DFJ	I	303	0	−1
1DFJ	I	304	0	−1
1DFJ	I	305	1	−1
1DFJ	I	306	1	−1
1DFJ	I	307	1	−1
1DFJ	I	308	1	−1
1DFJ	I	309	0	−1
1DFJ	I	310	1	−1
1DFJ	I	311	0	−1
1DFJ	I	312	1	−1
1DFJ	I	313	0	−1
1DFJ	I	314	0	−1
1DFJ	I	315	1	1
1DFJ	I	316	0	−1
1DFJ	I	317	1	1
1DFJ	I	318	1	−1
1DFJ	I	319	0	−1
1DFJ	I	320	1	−1
1DFJ	I	321	0	−1
1DFJ	I	322	1	−1
1DFJ	I	323	1	−1
1DFJ	I	324	1	−1
1DFJ	I	325	0	−1
1DFJ	I	326	0	−1
1DFJ	I	327	1	−1
1DFJ	I	328	1	−1
1DFJ	I	329	0	−1
1DFJ	I	330	0	−1
1DFJ	I	331	1	−1
1DFJ	I	332	0	−1
1DFJ	I	333	0	−1
1DFJ	I	334	1	−1
1DFJ	I	335	1	−1
1DFJ	I	336	0	−1
1DFJ	I	337	1	−1
1DFJ	I	338	1	−1

1DFJ	I	339	0	−1
1DFJ	I	340	1	−1
1DFJ	I	341	0	−1
1DFJ	I	342	0	−1
1DFJ	I	343	0	−1
1DFJ	I	344	0	−1
1DFJ	I	345	0	−1
1DFJ	I	346	1	−1
1DFJ	I	347	0	−1
1DFJ	I	348	1	−1
1DFJ	I	349	0	−1
1DFJ	I	350	0	−1
1DFJ	I	351	1	−1
1DFJ	I	352	1	−1
1DFJ	I	353	0	−1
1DFJ	I	354	0	−1
1DFJ	I	355	1	−1
1DFJ	I	356	1	−1
1DFJ	I	357	0	−1
1DFJ	I	358	0	−1
1DFJ	I	359	1	−1
1DFJ	I	360	0	−1
1DFJ	I	361	0	−1
1DFJ	I	362	1	−1
1DFJ	I	363	1	−1
1DFJ	I	364	1	−1
1DFJ	I	365	1	−1
1DFJ	I	366	0	−1
1DFJ	I	367	0	−1
1DFJ	I	368	0	−1
1DFJ	I	369	1	−1
1DFJ	I	370	0	−1
1DFJ	I	371	0	−1
1DFJ	I	372	0	−1
1DFJ	I	373	0	−1
1DFJ	I	374	0	−1
1DFJ	I	375	1	−1
1DFJ	I	376	0	−1
1DFJ	I	377	1	−1
1DFJ	I	378	0	−1
1DFJ	I	379	1	−1
1DFJ	I	380	1	1
1DFJ	I	381	1	−1
1DFJ	I	382	0	−1
1DFJ	I	383	0	−1
1DFJ	I	384	1	−1
1DFJ	I	385	0	−1
1DFJ	I	386	0	−1
1DFJ	I	387	0	−1
1DFJ	I	388	1	−1
1DFJ	I	389	0	−1
1DFJ	I	390	0	−1

1DFJ	I	391	1	−1
1DFJ	I	392	1	−1
1DFJ	I	393	0	−1
1DFJ	I	394	1	−1
1DFJ	I	395	0	−1
1DFJ	I	396	0	−1
1DFJ	I	397	1	−1
1DFJ	I	398	1	1
1DFJ	I	399	0	−1
1DFJ	I	400	0	−1
1DFJ	I	401	0	−1
1DFJ	I	402	0	−1
1DFJ	I	403	1	1
1DFJ	I	404	0	−1
1DFJ	I	405	1	1
1DFJ	I	406	0	−1
1DFJ	I	407	0	−1
1DFJ	I	408	1	1
1DFJ	I	409	1	1
1DFJ	I	410	0	−1
1DFJ	I	411	0	−1
1DFJ	I	412	1	−1
1DFJ	I	413	1	−1
1DFJ	I	414	0	−1
1DFJ	I	415	0	−1
1DFJ	I	416	1	−1
1DFJ	I	417	0	−1
1DFJ	I	418	0	−1
1DFJ	I	419	1	−1
1DFJ	I	420	1	−1
1DFJ	I	421	1	−1
1DFJ	I	422	1	−1
1DFJ	I	423	0	−1
1DFJ	I	424	1	−1
1DFJ	I	425	0	−1
1DFJ	I	426	1	−1
1DFJ	I	427	1	1
1DFJ	I	428	0	−1
1DFJ	I	429	0	−1
1DFJ	I	430	0	−1
1DFJ	I	431	1	1
1DFJ	I	432	1	1
1DFJ	I	433	0	−1
1DFJ	I	434	1	1
1DFJ	I	435	0	−1
1DFJ	I	436	1	1
1DFJ	I	437	1	1
1DFJ	I	438	1	−1
1DFJ	I	439	0	−1
1DFJ	I	440	1	1
1DFJ	I	441	1	−1
1DFJ	I	442	1	−1

1DFJ	I	443	0	−1
1DFJ	I	444	1	−1
1DFJ	I	445	1	−1
1DFJ	I	446	0	−1
1DFJ	I	447	1	−1
1DFJ	I	448	1	−1
1DFJ	I	449	1	−1
1DFJ	I	450	1	−1
1DFJ	I	451	1	−1
1DFJ	I	452	1	−1
1DFJ	I	453	0	−1
1DFJ	I	454	1	1
1DFJ	I	455	0	−1
1DFJ	I	456	0	−1
1DFJ	I	457	1	1
1DHK	A	2	1	−1
1DHK	A	3	1	−1
1DHK	A	4	0	−1
1DHK	A	5	0	−1
1DHK	A	6	0	−1
1DHK	A	7	1	−1
1DHK	A	8	1	−1
1DHK	A	9	1	−1
1DHK	A	10	0	−1
1DHK	A	11	0	−1
1DHK	A	12	0	−1
1DHK	A	13	0	−1
1DHK	A	14	0	−1
1DHK	A	15	0	−1
1DHK	A	16	0	−1
1DHK	A	17	0	−1
1DHK	A	18	0	−1
1DHK	A	19	0	−1
1DHK	A	20	0	−1
1DHK	A	21	0	−1
1DHK	A	22	1	−1
1DHK	A	23	0	−1
1DHK	A	24	0	−1
1DHK	A	25	0	−1
1DHK	A	26	1	−1
1DHK	A	27	0	−1
1DHK	A	28	0	−1
1DHK	A	29	1	−1
1DHK	A	30	1	−1
1DHK	A	31	0	−1
1DHK	A	32	0	−1
1DHK	A	33	0	−1
1DHK	A	34	1	−1
1DHK	A	35	1	−1
1DHK	A	36	0	−1
1DHK	A	37	0	−1
1DHK	A	38	0	−1

1DHK	A	39	0	−1
1DHK	A	40	0	−1
1DHK	A	41	0	−1
1DHK	A	42	0	−1
1DHK	A	43	0	−1
1DHK	A	44	0	−1
1DHK	A	45	0	−1
1DHK	A	46	0	−1
1DHK	A	47	0	−1
1DHK	A	48	0	−1
1DHK	A	49	0	−1
1DHK	A	50	1	−1
1DHK	A	51	0	−1
1DHK	A	52	1	−1
1DHK	A	53	1	−1
1DHK	A	54	0	−1
1DHK	A	55	1	−1
1DHK	A	56	0	−1
1DHK	A	57	0	−1
1DHK	A	58	0	−1
1DHK	A	59	1	1
1DHK	A	60	0	−1
1DHK	A	61	0	−1
1DHK	A	62	0	−1
1DHK	A	63	0	−1
1DHK	A	64	0	−1
1DHK	A	65	0	−1
1DHK	A	66	0	−1
1DHK	A	67	0	−1
1DHK	A	68	1	−1
1DHK	A	69	0	−1
1DHK	A	70	0	−1
1DHK	A	71	0	−1
1DHK	A	72	0	−1
1DHK	A	73	0	−1
1DHK	A	74	0	−1
1DHK	A	75	1	−1
1DHK	A	76	1	−1
1DHK	A	77	1	−1
1DHK	A	78	1	−1
1DHK	A	79	0	−1
1DHK	A	80	1	−1
1DHK	A	81	1	−1
1DHK	A	82	0	−1
1DHK	A	83	0	−1
1DHK	A	84	1	−1
1DHK	A	85	0	−1
1DHK	A	86	0	−1
1DHK	A	87	0	−1
1DHK	A	88	1	−1
1DHK	A	89	1	−1
1DHK	A	90	0	−1

1DHK	A	91	0	−1
1DHK	A	92	0	−1
1DHK	A	93	0	−1
1DHK	A	94	0	−1
1DHK	A	95	0	−1
1DHK	A	96	0	−1
1DHK	A	97	0	−1
1DHK	A	98	0	−1
1DHK	A	99	0	−1
1DHK	A	100	0	−1
1DHK	A	101	0	−1
1DHK	A	102	0	−1
1DHK	A	103	0	−1
1DHK	A	104	0	−1
1DHK	A	105	1	−1
1DHK	A	106	1	−1
1DHK	A	107	1	−1
1DHK	A	108	1	−1
1DHK	A	109	1	−1
1DHK	A	110	1	−1
1DHK	A	111	1	−1
1DHK	A	112	1	−1
1DHK	A	113	0	−1
1DHK	A	114	0	−1
1DHK	A	115	1	−1
1DHK	A	116	1	−1
1DHK	A	117	0	−1
1DHK	A	118	1	−1
1DHK	A	119	0	−1
1DHK	A	120	0	−1
1DHK	A	121	0	−1
1DHK	A	122	1	−1
1DHK	A	123	1	−1
1DHK	A	124	0	−1
1DHK	A	125	1	−1
1DHK	A	126	0	−1
1DHK	A	127	1	−1
1DHK	A	128	1	−1
1DHK	A	129	0	−1
1DHK	A	130	1	−1
1DHK	A	131	0	−1
1DHK	A	132	0	−1
1DHK	A	133	1	−1
1DHK	A	134	1	−1
1DHK	A	135	0	−1
1DHK	A	136	0	−1
1DHK	A	137	0	−1
1DHK	A	138	1	−1
1DHK	A	139	1	−1
1DHK	A	140	1	−1
1DHK	A	141	0	−1
1DHK	A	142	1	1

1DHK	A	143	0	−1
1DHK	A	144	1	1
1DHK	A	145	1	1
1DHK	A	146	1	−1
1DHK	A	147	0	−1
1DHK	A	148	0	−1
1DHK	A	149	1	1
1DHK	A	150	1	1
1DHK	A	151	1	1
1DHK	A	152	1	1
1DHK	A	153	0	−1
1DHK	A	154	1	−1
1DHK	A	155	1	−1
1DHK	A	156	0	−1
1DHK	A	157	0	−1
1DHK	A	158	0	−1
1DHK	A	159	0	−1
1DHK	A	160	0	−1
1DHK	A	161	0	−1
1DHK	A	162	0	−1
1DHK	A	163	1	1
1DHK	A	164	1	1
1DHK	A	165	0	−1
1DHK	A	166	0	−1
1DHK	A	167	0	−1
1DHK	A	168	0	−1
1DHK	A	169	0	−1
1DHK	A	170	0	−1
1DHK	A	171	1	−1
1DHK	A	172	1	−1
1DHK	A	173	1	−1
1DHK	A	174	1	−1
1DHK	A	175	0	−1
1DHK	A	176	0	−1
1DHK	A	177	0	−1
1DHK	A	178	0	−1
1DHK	A	179	0	−1
1DHK	A	180	0	−1
1DHK	A	181	1	−1
1DHK	A	182	0	−1
1DHK	A	183	0	−1
1DHK	A	184	0	−1
1DHK	A	185	1	−1
1DHK	A	186	0	−1
1DHK	A	187	0	−1
1DHK	A	188	1	−1
1DHK	A	189	0	−1
1DHK	A	190	0	−1
1DHK	A	191	0	−1
1DHK	A	192	0	−1
1DHK	A	193	0	−1
1DHK	A	194	0	−1

1DHK	A	195	0	−1
1DHK	A	196	0	−1
1DHK	A	197	0	−1
1DHK	A	198	0	−1
1DHK	A	199	0	−1
1DHK	A	200	0	−1
1DHK	A	201	0	−1
1DHK	A	202	0	−1
1DHK	A	203	1	−1
1DHK	A	204	0	−1
1DHK	A	205	1	−1
1DHK	A	206	0	−1
1DHK	A	207	0	−1
1DHK	A	208	1	−1
1DHK	A	209	0	−1
1DHK	A	210	0	−1
1DHK	A	211	0	−1
1DHK	A	212	1	−1
1DHK	A	213	1	−1
1DHK	A	214	0	−1
1DHK	A	215	1	−1
1DHK	A	216	1	−1
1DHK	A	217	0	−1
1DHK	A	218	0	−1
1DHK	A	219	1	−1
1DHK	A	220	1	−1
1DHK	A	221	0	−1
1DHK	A	222	0	−1
1DHK	A	223	1	−1
1DHK	A	224	1	−1
1DHK	A	225	1	−1
1DHK	A	226	0	−1
1DHK	A	227	1	−1
1DHK	A	228	0	−1
1DHK	A	229	0	−1
1DHK	A	230	0	−1
1DHK	A	231	0	−1
1DHK	A	232	0	−1
1DHK	A	233	0	−1
1DHK	A	234	0	−1
1DHK	A	235	1	1
1DHK	A	236	0	−1
1DHK	A	237	1	1
1DHK	A	238	1	1
1DHK	A	239	1	1
1DHK	A	240	0	−1
1DHK	A	241	1	−1
1DHK	A	242	0	−1
1DHK	A	243	1	−1
1DHK	A	244	0	−1
1DHK	A	245	1	−1
1DHK	A	246	1	−1

1DHK	A	247	0	−1
1DHK	A	248	0	−1
1DHK	A	249	1	−1
1DHK	A	250	0	−1
1DHK	A	251	0	−1
1DHK	A	252	0	−1
1DHK	A	253	0	−1
1DHK	A	254	0	−1
1DHK	A	255	0	−1
1DHK	A	256	0	−1
1DHK	A	257	1	−1
1DHK	A	258	0	−1
1DHK	A	259	0	−1
1DHK	A	260	1	1
1DHK	A	261	1	−1
1DHK	A	262	0	−1
1DHK	A	263	0	−1
1DHK	A	264	0	−1
1DHK	A	265	0	−1
1DHK	A	266	0	−1
1DHK	A	267	0	−1
1DHK	A	268	1	−1
1DHK	A	269	1	1
1DHK	A	270	1	1
1DHK	A	271	1	−1
1DHK	A	272	1	−1
1DHK	A	273	1	−1
1DHK	A	274	0	−1
1DHK	A	275	0	−1
1DHK	A	276	1	−1
1DHK	A	277	0	−1
1DHK	A	278	1	−1
1DHK	A	279	1	−1
1DHK	A	280	0	−1
1DHK	A	281	0	−1
1DHK	A	282	1	−1
1DHK	A	283	1	−1
1DHK	A	284	0	−1
1DHK	A	285	1	−1
1DHK	A	286	0	−1
1DHK	A	287	0	−1
1DHK	A	288	1	−1
1DHK	A	289	0	−1
1DHK	A	290	1	−1
1DHK	A	291	0	−1
1DHK	A	292	0	−1
1DHK	A	293	0	−1
1DHK	A	294	0	−1
1DHK	A	295	0	−1
1DHK	A	296	0	−1
1DHK	A	297	0	−1
1DHK	A	298	0	−1

1DHK	A	299	0	−1
1DHK	A	300	1	1
1DHK	A	301	0	−1
1DHK	A	302	0	−1
1DHK	A	303	0	−1
1DHK	A	304	1	1
1DHK	A	305	1	1
1DHK	A	306	0	−1
1DHK	A	307	1	1
1DHK	A	308	1	1
1DHK	A	309	1	1
1DHK	A	310	0	−1
1DHK	A	311	0	−1
1DHK	A	312	0	−1
1DHK	A	313	0	−1
1DHK	A	314	0	−1
1DHK	A	315	0	−1
1DHK	A	316	1	−1
1DHK	A	317	1	−1
1DHK	A	318	1	−1
1DHK	A	319	1	−1
1DHK	A	320	1	−1
1DHK	A	321	0	−1
1DHK	A	322	0	−1
1DHK	A	323	0	−1
1DHK	A	324	0	−1
1DHK	A	325	0	−1
1DHK	A	326	0	−1
1DHK	A	327	0	−1
1DHK	A	328	0	−1
1DHK	A	329	0	−1
1DHK	A	330	0	−1
1DHK	A	331	0	−1
1DHK	A	332	1	−1
1DHK	A	333	0	−1
1DHK	A	334	0	−1
1DHK	A	335	0	−1
1DHK	A	336	0	−1
1DHK	A	337	0	−1
1DHK	A	338	0	−1
1DHK	A	339	0	−1
1DHK	A	340	0	−1
1DHK	A	341	0	−1
1DHK	A	342	0	−1
1DHK	A	343	1	−1
1DHK	A	344	0	−1
1DHK	A	345	1	−1
1DHK	A	346	1	−1
1DHK	A	347	1	−1
1DHK	A	348	1	−1
1DHK	A	349	1	−1
1DHK	A	350	1	−1

1DHK	A	351	1	1
1DHK	A	352	1	1
1DHK	A	353	0	−1
1DHK	A	354	1	−1
1DHK	A	355	0	−1
1DHK	A	356	1	1
1DHK	A	357	1	−1
1DHK	A	358	0	−1
1DHK	A	359	0	−1
1DHK	A	360	0	−1
1DHK	A	361	0	−1
1DHK	A	362	1	−1
1DHK	A	363	1	−1
1DHK	A	364	1	−1
1DHK	A	365	0	−1
1DHK	A	366	1	−1
1DHK	A	367	0	−1
1DHK	A	368	0	−1
1DHK	A	369	1	−1
1DHK	A	370	0	−1
1DHK	A	371	1	−1
1DHK	A	372	1	−1
1DHK	A	373	1	−1
1DHK	A	374	1	−1
1DHK	A	375	1	−1
1DHK	A	376	1	−1
1DHK	A	377	1	−1
1DHK	A	378	0	−1
1DHK	A	379	0	−1
1DHK	A	380	1	−1
1DHK	A	381	1	−1
1DHK	A	382	0	−1
1DHK	A	383	0	−1
1DHK	A	384	0	−1
1DHK	A	385	0	−1
1DHK	A	386	0	−1
1DHK	A	387	0	−1
1DHK	A	388	1	−1
1DHK	A	389	1	−1
1DHK	A	390	0	−1
1DHK	A	391	0	−1
1DHK	A	392	0	−1
1DHK	A	393	0	−1
1DHK	A	394	0	−1
1DHK	A	395	0	−1
1DHK	A	396	1	−1
1DHK	A	397	0	−1
1DHK	A	398	0	−1
1DHK	A	399	0	−1
1DHK	A	400	0	−1
1DHK	A	401	0	−1
1DHK	A	402	1	−1

1DHK	A	403	1	−1
1DHK	A	404	1	−1
1DHK	A	405	1	−1
1DHK	A	406	1	−1
1DHK	A	407	1	−1
1DHK	A	408	1	−1
1DHK	A	409	0	−1
1DHK	A	410	1	−1
1DHK	A	411	1	−1
1DHK	A	412	1	−1
1DHK	A	413	1	−1
1DHK	A	414	0	−1
1DHK	A	415	0	−1
1DHK	A	416	0	−1
1DHK	A	417	0	−1
1DHK	A	418	0	−1
1DHK	A	419	0	−1
1DHK	A	420	0	−1
1DHK	A	421	0	−1
1DHK	A	422	0	−1
1DHK	A	423	1	−1
1DHK	A	424	1	−1
1DHK	A	425	0	−1
1DHK	A	426	0	−1
1DHK	A	427	0	−1
1DHK	A	428	0	−1
1DHK	A	429	0	−1
1DHK	A	430	0	−1
1DHK	A	431	0	−1
1DHK	A	432	0	−1
1DHK	A	433	1	−1
1DHK	A	434	1	−1
1DHK	A	435	1	−1
1DHK	A	436	0	−1
1DHK	A	437	1	−1
1DHK	A	438	1	−1
1DHK	A	439	1	−1
1DHK	A	440	0	−1
1DHK	A	441	1	−1
1DHK	A	442	0	−1
1DHK	A	443	1	−1
1DHK	A	444	0	−1
1DHK	A	445	1	−1
1DHK	A	446	1	−1
1DHK	A	447	1	−1
1DHK	A	448	1	−1
1DHK	A	449	0	−1
1DHK	A	450	0	−1
1DHK	A	451	0	−1
1DHK	A	452	0	−1
1DHK	A	453	0	−1
1DHK	A	454	1	−1

1DHK	A	455	0	−1
1DHK	A	456	1	−1
1DHK	A	457	1	−1
1DHK	A	458	1	−1
1DHK	A	459	1	−1
1DHK	A	460	1	−1
1DHK	A	461	1	−1
1DHK	A	462	0	−1
1DHK	A	463	1	−1
1DHK	A	464	1	−1
1DHK	A	465	1	−1
1DHK	A	466	1	−1
1DHK	A	467	0	−1
1DHK	A	468	1	−1
1DHK	A	469	0	−1
1DHK	A	470	1	−1
1DHK	A	471	1	−1
1DHK	A	472	1	−1
1DHK	A	473	0	−1
1DHK	A	474	0	−1
1DHK	A	475	0	−1
1DHK	A	476	1	−1
1DHK	A	477	0	−1
1DHK	A	478	1	−1
1DHK	A	479	0	−1
1DHK	A	480	1	−1
1DHK	A	481	1	−1
1DHK	A	482	1	−1
1DHK	A	483	1	−1
1DHK	A	484	1	−1
1DHK	A	485	0	−1
1DHK	A	486	0	−1
1DHK	A	487	0	−1
1DHK	A	488	0	−1
1DHK	A	489	0	−1
1DHK	A	490	0	−1
1DHK	A	491	0	−1
1DHK	A	492	1	−1
1DHK	A	493	1	−1
1DHK	A	494	0	−1
1DHK	A	495	1	−1
1DHK	A	496	1	−1
1DHK	B	1	1	−1
1DHK	B	2	1	−1
1DHK	B	3	1	−1
1DHK	B	4	0	−1
1DHK	B	5	1	−1
1DHK	B	6	0	−1
1DHK	B	7	1	−1
1DHK	B	8	0	−1
1DHK	B	9	1	−1
1DHK	B	10	1	−1

1DHK	B	11	0	−1
1DHK	B	12	1	−1
1DHK	B	13	1	1
1DHK	B	14	1	−1
1DHK	B	15	1	−1
1DHK	B	16	0	−1
1DHK	B	17	1	−1
1DHK	B	18	1	−1
1DHK	B	19	1	1
1DHK	B	20	1	1
1DHK	B	21	0	−1
1DHK	B	22	0	−1
1DHK	B	23	1	1
1DHK	B	24	0	−1
1DHK	B	25	0	−1
1DHK	B	26	1	1
1DHK	B	27	1	−1
1DHK	B	28	0	−1
1DHK	B	29	0	−1
1DHK	B	30	0	−1
1DHK	B	31	0	−1
1DHK	B	32	0	−1
1DHK	B	33	0	−1
1DHK	B	34	1	1
1DHK	B	35	1	1
1DHK	B	36	0	−1
1DHK	B	37	1	1
1DHK	B	38	1	1
1DHK	B	39	1	1
1DHK	B	40	1	1
1DHK	B	41	0	−1
1DHK	B	42	1	1
1DHK	B	43	0	−1
1DHK	B	44	0	−1
1DHK	B	45	0	−1
1DHK	B	46	1	−1
1DHK	B	47	1	−1
1DHK	B	48	1	−1
1DHK	B	49	0	−1
1DHK	B	50	0	−1
1DHK	B	51	0	−1
1DHK	B	52	0	−1
1DHK	B	53	0	−1
1DHK	B	54	1	−1
1DHK	B	55	1	−1
1DHK	B	56	1	−1
1DHK	B	57	1	−1
1DHK	B	58	1	−1
1DHK	B	59	0	−1
1DHK	B	60	0	−1
1DHK	B	61	1	−1
1DHK	B	62	0	−1

1DHK	B	63	1	−1
1DHK	B	64	0	−1
1DHK	B	65	1	−1
1DHK	B	66	0	−1
1DHK	B	67	0	−1
1DHK	B	68	0	−1
1DHK	B	69	0	−1
1DHK	B	70	0	−1
1DHK	B	71	1	1
1DHK	B	72	0	−1
1DHK	B	73	1	1
1DHK	B	74	1	−1
1DHK	B	78	0	−1
1DHK	B	79	1	1
1DHK	B	80	0	−1
1DHK	B	81	0	−1
1DHK	B	82	0	−1
1DHK	B	83	0	−1
1DHK	B	84	0	−1
1DHK	B	85	0	−1
1DHK	B	86	0	−1
1DHK	B	87	0	−1
1DHK	B	88	0	−1
1DHK	B	89	1	−1
1DHK	B	96	1	−1
1DHK	B	97	1	1
1DHK	B	98	0	−1
1DHK	B	99	0	−1
1DHK	B	100	0	−1
1DHK	B	101	0	−1
1DHK	B	102	0	−1
1DHK	B	103	0	−1
1DHK	B	104	0	−1
1DHK	B	105	1	1
1DHK	B	106	1	1
1DHK	B	107	1	−1
1DHK	B	108	0	−1
1DHK	B	109	0	−1
1DHK	B	110	0	−1
1DHK	B	111	0	−1
1DHK	B	112	0	−1
1DHK	B	113	0	−1
1DHK	B	114	1	−1
1DHK	B	115	1	1
1DHK	B	116	1	−1
1DHK	B	117	1	1
1DHK	B	118	1	−1
1DHK	B	119	1	−1
1DHK	B	120	1	−1
1DHK	B	121	0	−1
1DHK	B	122	1	−1
1DHK	B	123	0	−1

1DHK	B	124	1	−1
1DHK	B	125	0	−1
1DHK	B	126	1	−1
1DHK	B	127	1	−1
1DHK	B	128	0	−1
1DHK	B	129	1	1
1DHK	B	130	1	1
1DHK	B	131	1	−1
1DHK	B	132	1	−1
1DHK	B	133	0	−1
1DHK	B	134	1	−1
1DHK	B	135	0	−1
1DHK	B	136	1	−1
1DHK	B	137	0	−1
1DHK	B	138	1	−1
1DHK	B	139	0	−1
1DHK	B	140	1	−1
1DHK	B	141	0	−1
1DHK	B	142	1	−1
1DHK	B	143	1	−1
1DHK	B	144	0	−1
1DHK	B	145	1	−1
1DHK	B	146	0	−1
1DHK	B	147	1	−1
1DHK	B	148	0	−1
1DHK	B	149	1	−1
1DHK	B	150	0	−1
1DHK	B	151	1	−1
1DHK	B	152	0	−1
1DHK	B	153	1	−1
1DHK	B	154	1	−1
1DHK	B	155	1	−1
1DHK	B	156	1	−1
1DHK	B	157	1	−1
1DHK	B	158	1	−1
1DHK	B	159	0	−1
1DHK	B	160	1	−1
1DHK	B	161	0	−1
1DHK	B	162	1	−1
1DHK	B	163	0	−1
1DHK	B	164	1	−1
1DHK	B	165	0	−1
1DHK	B	166	1	−1
1DHK	B	167	0	−1
1DHK	B	168	1	−1
1DHK	B	169	1	−1
1DHK	B	170	1	−1
1DHK	B	171	1	−1
1DHK	B	172	0	−1
1DHK	B	173	0	−1
1DHK	B	174	1	−1
1DHK	B	175	0	−1

1DHK	B	176	0	−1
1DHK	B	177	0	−1
1DHK	B	178	0	−1
1DHK	B	179	0	−1
1DHK	B	180	0	−1
1DHK	B	181	0	−1
1DHK	B	182	0	−1
1DHK	B	183	0	−1
1DHK	B	184	0	−1
1DHK	B	185	0	−1
1DHK	B	186	1	1
1DHK	B	187	1	1
1DHK	B	188	1	1
1DHK	B	189	0	−1
1DHK	B	190	1	1
1DHK	B	191	0	−1
1DHK	B	192	0	−1
1DHK	B	193	0	−1
1DHK	B	194	0	−1
1DHK	B	195	0	−1
1DHK	B	196	0	−1
1DHK	B	197	1	−1
1DHK	B	198	0	−1
1DHK	B	199	0	−1
1DHK	B	200	0	−1
1DHK	B	201	1	−1
1DHK	B	202	0	−1
1DHK	B	203	1	−1
1DHK	B	204	1	−1
1DKG	A	34	1	−1
1DKG	A	35	1	−1
1DKG	A	36	1	−1
1DKG	A	37	1	−1
1DKG	A	38	1	−1
1DKG	A	39	1	−1
1DKG	A	40	1	−1
1DKG	A	41	1	−1
1DKG	A	42	1	−1
1DKG	A	43	1	−1
1DKG	A	44	0	−1
1DKG	A	45	0	−1
1DKG	A	46	1	−1
1DKG	A	47	0	−1
1DKG	A	48	1	−1
1DKG	A	49	1	−1
1DKG	A	50	1	−1
1DKG	A	51	0	−1
1DKG	A	52	1	−1
1DKG	A	53	1	−1
1DKG	A	54	0	−1
1DKG	A	55	1	−1
1DKG	A	56	1	−1

1DKG	A	57	1	−1
1DKG	A	58	0	−1
1DKG	A	59	1	−1
1DKG	A	60	1	−1
1DKG	A	61	0	−1
1DKG	A	62	0	−1
1DKG	A	63	1	−1
1DKG	A	64	1	−1
1DKG	A	65	0	−1
1DKG	A	66	1	−1
1DKG	A	67	1	−1
1DKG	A	68	1	−1
1DKG	A	69	0	−1
1DKG	A	70	1	−1
1DKG	A	71	1	1
1DKG	A	72	0	−1
1DKG	A	73	1	−1
1DKG	A	74	1	1
1DKG	A	75	0	−1
1DKG	A	76	0	−1
1DKG	A	77	1	−1
1DKG	A	78	1	1
1DKG	A	79	1	1
1DKG	A	80	1	−1
1DKG	A	81	1	−1
1DKG	A	82	1	1
1DKG	A	83	1	−1
1DKG	A	84	1	−1
1DKG	A	85	1	−1
1DKG	A	86	0	−1
1DKG	A	87	0	−1
1DKG	A	88	0	−1
1DKG	A	89	0	−1
1DKG	A	90	1	−1
1DKG	A	91	0	−1
1DKG	A	92	0	−1
1DKG	A	93	1	−1
1DKG	A	94	0	−1
1DKG	A	95	0	−1
1DKG	A	96	0	−1
1DKG	A	97	1	1
1DKG	A	98	0	−1
1DKG	A	99	0	−1
1DKG	A	100	0	−1
1DKG	A	101	0	−1
1DKG	A	102	0	−1
1DKG	A	103	0	−1
1DKG	A	104	1	1
1DKG	A	105	0	−1
1DKG	A	106	0	−1
1DKG	A	107	1	1
1DKG	A	108	1	1

1DKG	A	109	1	1
1DKG	A	116	1	−1
1DKG	A	117	1	−1
1DKG	A	118	1	−1
1DKG	A	119	0	−1
1DKG	A	120	1	−1
1DKG	A	121	1	−1
1DKG	A	122	0	−1
1DKG	A	123	0	−1
1DKG	A	124	1	−1
1DKG	A	125	1	−1
1DKG	A	126	0	−1
1DKG	A	127	0	−1
1DKG	A	128	1	−1
1DKG	A	129	0	−1
1DKG	A	130	0	−1
1DKG	A	131	0	−1
1DKG	A	132	1	−1
1DKG	A	133	0	−1
1DKG	A	134	0	−1
1DKG	A	135	1	−1
1DKG	A	136	1	−1
1DKG	A	137	0	−1
1DKG	A	138	1	−1
1DKG	A	139	0	−1
1DKG	A	140	1	−1
1DKG	A	141	0	−1
1DKG	A	142	0	−1
1DKG	A	143	1	−1
1DKG	A	144	1	−1
1DKG	A	145	1	−1
1DKG	A	146	1	−1
1DKG	A	147	0	−1
1DKG	A	148	1	−1
1DKG	A	149	1	1
1DKG	A	150	1	−1
1DKG	A	151	1	1
1DKG	A	152	1	1
1DKG	A	153	0	−1
1DKG	A	154	0	−1
1DKG	A	155	1	1
1DKG	A	156	1	1
1DKG	A	157	1	1
1DKG	A	158	1	1
1DKG	A	159	1	1
1DKG	A	160	1	1
1DKG	A	161	1	−1
1DKG	A	162	0	−1
1DKG	A	163	1	−1
1DKG	A	164	1	−1
1DKG	A	165	0	−1
1DKG	A	166	1	−1

1DKG	A	167	1	−1
1DKG	A	168	1	−1
1DKG	A	169	0	−1
1DKG	A	170	0	−1
1DKG	A	171	0	−1
1DKG	A	172	1	−1
1DKG	A	173	1	−1
1DKG	A	174	1	1
1DKG	A	175	1	−1
1DKG	A	176	0	−1
1DKG	A	177	0	−1
1DKG	A	178	0	−1
1DKG	A	179	0	−1
1DKG	A	180	0	−1
1DKG	A	181	1	−1
1DKG	A	182	1	−1
1DKG	A	183	1	1
1DKG	A	184	1	1
1DKG	A	185	0	−1
1DKG	A	186	0	−1
1DKG	A	187	1	1
1DKG	A	188	0	−1
1DKG	A	189	1	1
1DKG	A	190	0	−1
1DKG	A	191	0	−1
1DKG	A	192	0	−1
1DKG	A	193	0	−1
1DKG	A	194	0	−1
1DKG	A	195	1	−1
1DKG	A	196	1	−1
1DKG	A	197	1	−1
1DKG	B	38	1	−1
1DKG	B	39	1	−1
1DKG	B	40	1	−1
1DKG	B	41	1	−1
1DKG	B	42	1	−1
1DKG	B	43	1	−1
1DKG	B	44	0	−1
1DKG	B	45	1	−1
1DKG	B	46	1	−1
1DKG	B	47	0	−1
1DKG	B	48	1	−1
1DKG	B	49	1	−1
1DKG	B	50	1	−1
1DKG	B	51	0	−1
1DKG	B	52	1	−1
1DKG	B	53	1	−1
1DKG	B	54	0	−1
1DKG	B	55	1	−1
1DKG	B	56	1	−1
1DKG	B	57	1	−1
1DKG	B	58	0	−1

1DKG	B	59	1	−1
1DKG	B	60	1	−1
1DKG	B	61	1	−1
1DKG	B	62	0	−1
1DKG	B	63	1	−1
1DKG	B	64	0	−1
1DKG	B	65	0	−1
1DKG	B	66	1	−1
1DKG	B	67	1	−1
1DKG	B	68	1	−1
1DKG	B	69	0	−1
1DKG	B	70	1	−1
1DKG	B	71	1	−1
1DKG	B	72	0	−1
1DKG	B	73	1	1
1DKG	B	74	1	−1
1DKG	B	75	1	−1
1DKG	B	76	0	−1
1DKG	B	77	1	−1
1DKG	B	78	1	−1
1DKG	B	79	1	−1
1DKG	B	80	0	−1
1DKG	B	81	0	−1
1DKG	B	82	1	−1
1DKG	B	83	1	−1
1DKG	B	84	1	−1
1DKG	B	85	1	−1
1DKG	B	86	1	−1
1DKG	B	87	0	−1
1DKG	B	88	0	−1
1DKG	B	89	0	−1
1DKG	B	90	1	−1
1DKG	B	91	0	−1
1DKG	B	92	0	−1
1DKG	B	93	1	−1
1DKG	B	94	1	−1
1DKG	B	95	0	−1
1DKG	B	96	0	−1
1DKG	B	97	1	−1
1DKG	B	98	0	−1
1DKG	B	99	0	−1
1DKG	B	100	0	−1
1DKG	B	101	0	−1
1DKG	B	102	0	−1
1DKG	B	103	0	−1
1DKG	B	104	1	−1
1DKG	B	105	0	−1
1DKG	B	106	1	−1
1DKG	B	107	1	−1
1DKG	B	108	1	−1
1DKG	B	116	1	−1
1DKG	B	117	1	−1

1DKG	B	118	1	−1
1DKG	B	119	0	−1
1DKG	B	120	0	−1
1DKG	B	121	1	−1
1DKG	B	122	1	−1
1DKG	B	123	0	−1
1DKG	B	124	1	−1
1DKG	B	125	1	1
1DKG	B	126	0	−1
1DKG	B	127	0	−1
1DKG	B	128	1	−1
1DKG	B	129	0	−1
1DKG	B	130	0	−1
1DKG	B	131	0	−1
1DKG	B	132	1	−1
1DKG	B	133	0	−1
1DKG	B	134	0	−1
1DKG	B	135	1	−1
1DKG	B	136	1	−1
1DKG	B	137	0	−1
1DKG	B	138	0	−1
1DKG	B	139	0	−1
1DKG	B	140	1	−1
1DKG	B	141	1	−1
1DKG	B	142	0	−1
1DKG	B	143	1	−1
1DKG	B	144	1	−1
1DKG	B	145	1	−1
1DKG	B	146	1	−1
1DKG	B	147	0	−1
1DKG	B	148	1	−1
1DKG	B	149	1	−1
1DKG	B	150	1	−1
1DKG	B	151	1	−1
1DKG	B	152	1	−1
1DKG	B	153	0	−1
1DKG	B	154	0	−1
1DKG	B	155	1	−1
1DKG	B	156	1	−1
1DKG	B	157	1	−1
1DKG	B	158	1	−1
1DKG	B	159	1	−1
1DKG	B	160	1	−1
1DKG	B	161	1	−1
1DKG	B	162	0	−1
1DKG	B	163	1	−1
1DKG	B	164	1	−1
1DKG	B	165	0	−1
1DKG	B	166	1	−1
1DKG	B	167	1	−1
1DKG	B	168	1	−1
1DKG	B	169	0	−1

1DKG	B	170	0	−1
1DKG	B	171	0	−1
1DKG	B	172	1	−1
1DKG	B	173	1	−1
1DKG	B	174	1	−1
1DKG	B	175	1	−1
1DKG	B	176	1	−1
1DKG	B	177	0	−1
1DKG	B	178	0	−1
1DKG	B	179	0	−1
1DKG	B	180	0	−1
1DKG	B	181	1	−1
1DKG	B	182	1	−1
1DKG	B	183	1	−1
1DKG	B	184	1	−1
1DKG	B	185	0	−1
1DKG	B	186	0	−1
1DKG	B	187	0	−1
1DKG	B	188	0	−1
1DKG	B	189	1	−1
1DKG	B	190	0	−1
1DKG	B	191	0	−1
1DKG	B	192	0	−1
1DKG	B	193	0	−1
1DKG	B	194	1	−1
1DKG	B	195	1	−1
1DKG	D	3	1	−1
1DKG	D	4	0	−1
1DKG	D	5	0	−1
1DKG	D	6	0	−1
1DKG	D	7	0	−1
1DKG	D	8	0	−1
1DKG	D	9	0	−1
1DKG	D	10	0	−1
1DKG	D	11	0	−1
1DKG	D	12	1	−1
1DKG	D	13	1	−1
1DKG	D	14	0	−1
1DKG	D	15	0	−1
1DKG	D	16	0	−1
1DKG	D	17	0	−1
1DKG	D	18	0	−1
1DKG	D	19	0	−1
1DKG	D	20	1	1
1DKG	D	21	1	−1
1DKG	D	22	1	−1
1DKG	D	23	1	−1
1DKG	D	24	1	−1
1DKG	D	25	1	−1
1DKG	D	26	0	−1
1DKG	D	27	0	−1
1DKG	D	28	1	1

1DKG	D	29	0	−1
1DKG	D	30	1	1
1DKG	D	31	1	1
1DKG	D	32	1	1
1DKG	D	33	1	−1
1DKG	D	34	1	1
1DKG	D	35	1	−1
1DKG	D	36	0	−1
1DKG	D	37	0	−1
1DKG	D	38	0	−1
1DKG	D	39	0	−1
1DKG	D	40	0	−1
1DKG	D	41	0	−1
1DKG	D	42	0	−1
1DKG	D	43	0	−1
1DKG	D	44	1	−1
1DKG	D	45	1	−1
1DKG	D	46	1	−1
1DKG	D	47	1	−1
1DKG	D	48	1	−1
1DKG	D	49	0	−1
1DKG	D	50	0	−1
1DKG	D	51	0	−1
1DKG	D	52	0	−1
1DKG	D	53	1	1
1DKG	D	54	0	−1
1DKG	D	55	1	−1
1DKG	D	56	1	1
1DKG	D	57	0	−1
1DKG	D	58	1	−1
1DKG	D	59	1	1
1DKG	D	60	1	1
1DKG	D	61	0	−1
1DKG	D	62	1	−1
1DKG	D	63	1	−1
1DKG	D	64	0	−1
1DKG	D	65	0	−1
1DKG	D	66	0	−1
1DKG	D	67	1	−1
1DKG	D	68	0	−1
1DKG	D	69	0	−1
1DKG	D	70	0	−1
1DKG	D	71	0	−1
1DKG	D	72	0	−1
1DKG	D	73	0	−1
1DKG	D	74	0	−1
1DKG	D	75	0	−1
1DKG	D	76	1	−1
1DKG	D	77	1	−1
1DKG	D	78	1	−1
1DKG	D	79	1	−1
1DKG	D	80	1	−1

1DKG	D	81	0	−1
1DKG	D	82	0	−1
1DKG	D	83	1	−1
1DKG	D	84	0	−1
1DKG	D	85	0	−1
1DKG	D	86	1	−1
1DKG	D	87	1	−1
1DKG	D	88	1	−1
1DKG	D	89	0	−1
1DKG	D	90	1	−1
1DKG	D	91	0	−1
1DKG	D	92	1	−1
1DKG	D	93	0	−1
1DKG	D	94	0	−1
1DKG	D	95	1	−1
1DKG	D	96	0	−1
1DKG	D	97	1	−1
1DKG	D	98	1	−1
1DKG	D	99	1	−1
1DKG	D	100	1	−1
1DKG	D	101	0	−1
1DKG	D	102	1	−1
1DKG	D	103	0	−1
1DKG	D	104	1	−1
1DKG	D	105	0	−1
1DKG	D	106	1	−1
1DKG	D	107	1	−1
1DKG	D	108	1	−1
1DKG	D	109	1	−1
1DKG	D	110	0	−1
1DKG	D	111	0	−1
1DKG	D	112	0	−1
1DKG	D	113	0	−1
1DKG	D	114	1	−1
1DKG	D	115	0	−1
1DKG	D	116	0	−1
1DKG	D	117	0	−1
1DKG	D	118	1	−1
1DKG	D	119	0	−1
1DKG	D	120	0	−1
1DKG	D	121	1	−1
1DKG	D	122	0	−1
1DKG	D	123	0	−1
1DKG	D	124	0	−1
1DKG	D	125	1	−1
1DKG	D	126	0	−1
1DKG	D	127	0	−1
1DKG	D	128	0	−1
1DKG	D	129	1	1
1DKG	D	130	1	1
1DKG	D	131	1	1
1DKG	D	132	1	1

1DKG	D	133	1	1
1DKG	D	134	1	−1
1DKG	D	135	0	−1
1DKG	D	136	1	−1
1DKG	D	137	1	−1
1DKG	D	138	0	−1
1DKG	D	139	0	−1
1DKG	D	140	0	−1
1DKG	D	141	0	−1
1DKG	D	142	0	−1
1DKG	D	143	0	−1
1DKG	D	144	0	−1
1DKG	D	145	1	−1
1DKG	D	146	0	−1
1DKG	D	147	1	−1
1DKG	D	148	1	−1
1DKG	D	149	1	−1
1DKG	D	150	0	−1
1DKG	D	151	0	−1
1DKG	D	152	1	−1
1DKG	D	153	0	−1
1DKG	D	154	0	−1
1DKG	D	155	1	−1
1DKG	D	156	1	−1
1DKG	D	157	0	−1
1DKG	D	158	0	−1
1DKG	D	159	1	−1
1DKG	D	160	1	−1
1DKG	D	161	0	−1
1DKG	D	162	1	−1
1DKG	D	163	0	−1
1DKG	D	164	1	−1
1DKG	D	165	0	−1
1DKG	D	166	1	−1
1DKG	D	167	0	−1
1DKG	D	168	0	−1
1DKG	D	169	0	−1
1DKG	D	170	0	−1
1DKG	D	171	0	−1
1DKG	D	172	0	−1
1DKG	D	173	0	−1
1DKG	D	174	0	−1
1DKG	D	175	0	−1
1DKG	D	176	0	−1
1DKG	D	177	0	−1
1DKG	D	178	0	−1
1DKG	D	179	0	−1
1DKG	D	180	1	−1
1DKG	D	181	0	−1
1DKG	D	182	1	−1
1DKG	D	183	1	−1
1DKG	D	185	1	−1

1DKG	D	186	1	−1
1DKG	D	187	1	−1
1DKG	D	188	1	−1
1DKG	D	189	0	−1
1DKG	D	190	0	−1
1DKG	D	191	0	−1
1DKG	D	192	0	−1
1DKG	D	193	0	−1
1DKG	D	194	0	−1
1DKG	D	195	0	−1
1DKG	D	196	0	−1
1DKG	D	197	1	−1
1DKG	D	198	0	−1
1DKG	D	199	0	−1
1DKG	D	200	0	−1
1DKG	D	201	0	−1
1DKG	D	202	0	−1
1DKG	D	203	0	−1
1DKG	D	204	0	−1
1DKG	D	205	0	−1
1DKG	D	206	1	−1
1DKG	D	207	0	−1
1DKG	D	208	1	−1
1DKG	D	209	1	−1
1DKG	D	214	1	−1
1DKG	D	215	1	−1
1DKG	D	216	1	−1
1DKG	D	217	1	−1
1DKG	D	218	1	−1
1DKG	D	219	1	−1
1DKG	D	220	0	−1
1DKG	D	221	1	−1
1DKG	D	222	1	−1
1DKG	D	223	1	−1
1DKG	D	224	0	−1
1DKG	D	225	1	−1
1DKG	D	226	1	−1
1DKG	D	227	0	−1
1DKG	D	228	0	−1
1DKG	D	229	0	−1
1DKG	D	230	1	−1
1DKG	D	231	0	−1
1DKG	D	232	0	−1
1DKG	D	233	0	−1
1DKG	D	234	1	−1
1DKG	D	235	1	−1
1DKG	D	236	0	−1
1DKG	D	237	0	−1
1DKG	D	238	1	−1
1DKG	D	239	0	−1
1DKG	D	240	0	−1
1DKG	D	241	0	−1

1DKG	D	242	1	−1
1DKG	D	243	0	−1
1DKG	D	244	0	−1
1DKG	D	245	1	−1
1DKG	D	246	1	−1
1DKG	D	247	1	−1
1DKG	D	248	1	−1
1DKG	D	249	1	−1
1DKG	D	250	0	−1
1DKG	D	251	1	−1
1DKG	D	252	0	−1
1DKG	D	253	1	−1
1DKG	D	254	1	−1
1DKG	D	255	1	−1
1DKG	D	256	1	1
1DKG	D	257	1	1
1DKG	D	258	0	−1
1DKG	D	259	0	−1
1DKG	D	260	1	1
1DKG	D	261	1	1
1DKG	D	262	0	−1
1DKG	D	263	1	−1
1DKG	D	264	1	1
1DKG	D	265	0	−1
1DKG	D	266	0	−1
1DKG	D	267	1	−1
1DKG	D	268	1	1
1DKG	D	269	0	−1
1DKG	D	270	0	−1
1DKG	D	271	1	−1
1DKG	D	272	1	−1
1DKG	D	273	0	−1
1DKG	D	274	0	−1
1DKG	D	275	1	−1
1DKG	D	276	1	−1
1DKG	D	277	1	−1
1DKG	D	278	1	−1
1DKG	D	279	0	−1
1DKG	D	280	1	−1
1DKG	D	281	0	−1
1DKG	D	282	1	1
1DKG	D	283	0	−1
1DKG	D	284	1	1
1DKG	D	285	1	1
1DKG	D	286	0	−1
1DKG	D	287	0	−1
1DKG	D	288	1	−1
1DKG	D	289	1	−1
1DKG	D	290	1	−1
1DKG	D	291	1	−1
1DKG	D	292	1	−1
1DKG	D	293	1	−1

1DKG	D	294	0	−1
1DKG	D	295	1	1
1DKG	D	296	0	−1
1DKG	D	297	1	−1
1DKG	D	298	0	−1
1DKG	D	299	1	−1
1DKG	D	300	0	−1
1DKG	D	301	0	−1
1DKG	D	302	1	−1
1DKG	D	303	1	−1
1DKG	D	304	1	−1
1DKG	D	305	0	−1
1DKG	D	306	1	−1
1DKG	D	307	1	−1
1DKG	D	308	1	−1
1DKG	D	309	0	−1
1DKG	D	310	1	−1
1DKG	D	311	1	−1
1DKG	D	312	0	−1
1DKG	D	313	0	−1
1DKG	D	314	1	−1
1DKG	D	315	1	−1
1DKG	D	316	0	−1
1DKG	D	317	0	−1
1DKG	D	318	1	−1
1DKG	D	319	1	−1
1DKG	D	320	0	−1
1DKG	D	321	1	−1
1DKG	D	322	1	−1
1DKG	D	323	0	−1
1DKG	D	324	0	−1
1DKG	D	325	1	−1
1DKG	D	326	1	−1
1DKG	D	327	0	−1
1DKG	D	328	1	−1
1DKG	D	329	1	−1
1DKG	D	330	1	−1
1DKG	D	331	1	−1
1DKG	D	332	1	−1
1DKG	D	333	1	−1
1DKG	D	334	0	−1
1DKG	D	335	1	−1
1DKG	D	336	1	−1
1DKG	D	337	0	−1
1DKG	D	338	0	−1
1DKG	D	339	0	−1
1DKG	D	340	0	−1
1DKG	D	341	0	−1
1DKG	D	342	1	−1
1DKG	D	343	0	−1
1DKG	D	344	0	−1
1DKG	D	345	1	−1

1DKG	D	346	0	−1
1DKG	D	347	0	−1
1DKG	D	348	0	−1
1DKG	D	349	0	−1
1DKG	D	350	1	−1
1DKG	D	351	1	−1
1DKG	D	352	0	−1
1DKG	D	353	0	−1
1DKG	D	354	0	−1
1DKG	D	355	1	−1
1DKG	D	356	0	−1
1DKG	D	357	0	−1
1DKG	D	358	1	−1
1DKG	D	359	1	−1
1DKG	D	360	1	−1
1DKG	D	361	0	−1
1DKG	D	362	1	−1
1DKG	D	363	1	−1
1DKG	D	364	1	−1
1DKG	D	365	0	−1
1DKG	D	366	1	−1
1DKG	D	367	0	−1
1DKG	D	368	1	−1
1DKG	D	369	0	−1
1DKG	D	370	0	−1
1DKG	D	371	0	−1
1DKG	D	372	0	−1
1DKG	D	373	0	−1
1DKG	D	374	0	−1
1DKG	D	375	0	−1
1DKG	D	376	0	−1
1DKG	D	377	1	−1
1DKG	D	378	1	−1
1DKG	D	379	0	−1
1DKG	D	380	0	−1
1DKG	D	381	1	−1
1DKG	D	382	1	−1
1DKG	D	383	1	−1
1DVF	A	1	1	−1
1DVF	A	2	0	−1
1DVF	A	3	1	−1
1DVF	A	4	0	−1
1DVF	A	5	1	−1
1DVF	A	6	0	−1
1DVF	A	7	1	−1
1DVF	A	8	1	−1
1DVF	A	9	1	−1
1DVF	A	10	1	−1
1DVF	A	11	0	−1
1DVF	A	12	1	−1
1DVF	A	13	0	−1
1DVF	A	14	1	−1

1DVF	A	15	1	−1
1DVF	A	16	1	−1
1DVF	A	17	1	−1
1DVF	A	18	1	−1
1DVF	A	19	0	−1
1DVF	A	20	1	−1
1DVF	A	21	0	−1
1DVF	A	22	1	−1
1DVF	A	23	0	−1
1DVF	A	24	1	−1
1DVF	A	25	0	−1
1DVF	A	26	1	−1
1DVF	A	27	1	−1
1DVF	A	28	1	−1
1DVF	A	29	0	−1
1DVF	A	30	1	1
1DVF	A	31	1	−1
1DVF	A	32	1	1
1DVF	A	33	0	−1
1DVF	A	34	0	−1
1DVF	A	35	0	−1
1DVF	A	36	0	−1
1DVF	A	37	0	−1
1DVF	A	38	0	−1
1DVF	A	39	1	−1
1DVF	A	40	1	−1
1DVF	A	41	1	−1
1DVF	A	42	1	−1
1DVF	A	43	0	−1
1DVF	A	44	0	−1
1DVF	A	45	1	−1
1DVF	A	46	0	−1
1DVF	A	47	0	−1
1DVF	A	48	0	−1
1DVF	A	49	0	−1
1DVF	A	50	1	1
1DVF	A	51	0	−1
1DVF	A	52	1	−1
1DVF	A	53	1	1
1DVF	A	54	1	−1
1DVF	A	55	0	−1
1DVF	A	56	1	1
1DVF	A	57	1	−1
1DVF	A	58	0	−1
1DVF	A	59	1	−1
1DVF	A	60	1	−1
1DVF	A	61	0	−1
1DVF	A	62	0	−1
1DVF	A	63	1	−1
1DVF	A	64	0	−1
1DVF	A	65	1	−1
1DVF	A	66	1	−1

1DVF	A	67	1	−1
1DVF	A	68	1	−1
1DVF	A	69	1	−1
1DVF	A	70	1	−1
1DVF	A	71	0	−1
1DVF	A	72	0	−1
1DVF	A	73	0	−1
1DVF	A	74	1	−1
1DVF	A	75	0	−1
1DVF	A	76	1	−1
1DVF	A	77	1	−1
1DVF	A	78	0	−1
1DVF	A	79	1	−1
1DVF	A	80	1	−1
1DVF	A	81	1	−1
1DVF	A	82	0	−1
1DVF	A	83	0	−1
1DVF	A	84	0	−1
1DVF	A	85	0	−1
1DVF	A	86	0	−1
1DVF	A	87	0	−1
1DVF	A	88	0	−1
1DVF	A	89	0	−1
1DVF	A	90	0	−1
1DVF	A	91	0	−1
1DVF	A	92	1	1
1DVF	A	93	1	1
1DVF	A	94	1	1
1DVF	A	95	0	−1
1DVF	A	96	0	−1
1DVF	A	97	0	−1
1DVF	A	98	0	−1
1DVF	A	99	0	−1
1DVF	A	100	1	−1
1DVF	A	101	0	−1
1DVF	A	102	0	−1
1DVF	A	103	1	−1
1DVF	A	104	0	−1
1DVF	A	105	1	−1
1DVF	A	106	1	−1
1DVF	A	107	1	−1
1DVF	B	1	1	−1
1DVF	B	2	1	−1
1DVF	B	3	1	−1
1DVF	B	4	0	−1
1DVF	B	5	1	−1
1DVF	B	6	0	−1
1DVF	B	7	1	−1
1DVF	B	8	1	−1
1DVF	B	9	1	−1
1DVF	B	10	1	−1
1DVF	B	11	1	−1

1DVF	B	12	0	−1
1DVF	B	13	0	−1
1DVF	B	14	1	−1
1DVF	B	15	1	−1
1DVF	B	16	1	−1
1DVF	B	17	1	−1
1DVF	B	18	0	−1
1DVF	B	19	1	−1
1DVF	B	20	0	−1
1DVF	B	21	0	−1
1DVF	B	22	0	−1
1DVF	B	23	1	−1
1DVF	B	24	0	−1
1DVF	B	25	1	−1
1DVF	B	26	1	−1
1DVF	B	27	0	−1
1DVF	B	28	1	−1
1DVF	B	29	0	−1
1DVF	B	30	1	1
1DVF	B	31	1	1
1DVF	B	32	1	1
1DVF	B	33	0	−1
1DVF	B	34	0	−1
1DVF	B	35	0	−1
1DVF	B	36	0	−1
1DVF	B	37	0	−1
1DVF	B	38	0	−1
1DVF	B	39	0	−1
1DVF	B	40	0	−1
1DVF	B	41	1	−1
1DVF	B	42	1	−1
1DVF	B	43	1	−1
1DVF	B	44	1	−1
1DVF	B	45	0	−1
1DVF	B	46	1	−1
1DVF	B	47	0	−1
1DVF	B	48	0	−1
1DVF	B	49	0	−1
1DVF	B	50	0	−1
1DVF	B	51	0	−1
1DVF	B	52	1	1
1DVF	B	53	0	−1
1DVF	B	54	1	1
1DVF	B	55	1	−1
1DVF	B	56	1	1
1DVF	B	57	1	1
1DVF	B	58	1	1
1DVF	B	59	0	−1
1DVF	B	60	0	−1
1DVF	B	61	1	−1
1DVF	B	62	1	−1
1DVF	B	63	0	−1

1DVF	B	64	1	−1
1DVF	B	65	1	−1
1DVF	B	66	0	−1
1DVF	B	67	0	−1
1DVF	B	68	1	−1
1DVF	B	69	0	−1
1DVF	B	70	1	−1
1DVF	B	71	0	−1
1DVF	B	72	1	−1
1DVF	B	73	1	−1
1DVF	B	74	1	−1
1DVF	B	75	1	−1
1DVF	B	76	1	−1
1DVF	B	77	0	−1
1DVF	B	78	0	−1
1DVF	B	79	0	−1
1DVF	B	80	0	−1
1DVF	B	81	1	−1
1DVF	B	82	0	−1
1DVF	B	83	1	−1
1DVF	B	84	1	−1
1DVF	B	85	0	−1
1DVF	B	86	1	−1
1DVF	B	87	1	−1
1DVF	B	88	1	−1
1DVF	B	89	0	−1
1DVF	B	90	0	−1
1DVF	B	91	0	−1
1DVF	B	92	1	−1
1DVF	B	93	0	−1
1DVF	B	94	0	−1
1DVF	B	95	0	−1
1DVF	B	96	0	−1
1DVF	B	97	0	−1
1DVF	B	98	0	−1
1DVF	B	99	1	1
1DVF	B	100	1	1
1DVF	B	101	1	1
1DVF	B	102	0	−1
1DVF	B	103	0	−1
1DVF	B	104	0	−1
1DVF	B	105	1	−1
1DVF	B	106	0	−1
1DVF	B	107	0	−1
1DVF	B	108	1	−1
1DVF	B	109	0	−1
1DVF	B	110	0	−1
1DVF	B	111	1	−1
1DVF	B	112	0	−1
1DVF	B	113	1	−1
1DVF	B	114	0	−1
1DVF	B	115	1	−1

1DVF	B	116	1	−1
1DVF	C	1	1	−1
1DVF	C	2	0	−1
1DVF	C	3	1	−1
1DVF	C	4	0	−1
1DVF	C	5	1	−1
1DVF	C	6	0	−1
1DVF	C	7	1	−1
1DVF	C	8	1	−1
1DVF	C	9	1	−1
1DVF	C	10	1	−1
1DVF	C	11	0	−1
1DVF	C	12	1	−1
1DVF	C	13	0	−1
1DVF	C	14	0	−1
1DVF	C	15	1	−1
1DVF	C	16	1	−1
1DVF	C	17	1	−1
1DVF	C	18	1	−1
1DVF	C	19	0	−1
1DVF	C	20	1	−1
1DVF	C	21	0	−1
1DVF	C	22	1	−1
1DVF	C	23	0	−1
1DVF	C	24	1	−1
1DVF	C	25	0	−1
1DVF	C	26	1	−1
1DVF	C	27	1	−1
1DVF	C	28	1	−1
1DVF	C	29	0	−1
1DVF	C	30	1	−1
1DVF	C	31	1	−1
1DVF	C	32	0	−1
1DVF	C	33	0	−1
1DVF	C	34	0	−1
1DVF	C	35	0	−1
1DVF	C	36	0	−1
1DVF	C	37	0	−1
1DVF	C	38	0	−1
1DVF	C	39	1	−1
1DVF	C	40	1	−1
1DVF	C	41	1	−1
1DVF	C	42	0	−1
1DVF	C	43	0	−1
1DVF	C	44	0	−1
1DVF	C	45	1	−1
1DVF	C	46	0	−1
1DVF	C	47	0	−1
1DVF	C	48	0	−1
1DVF	C	49	0	−1
1DVF	C	50	1	1
1DVF	C	51	0	−1

1DVF	C	52	1	−1
1DVF	C	53	1	1
1DVF	C	54	1	−1
1DVF	C	55	0	−1
1DVF	C	56	1	−1
1DVF	C	57	1	−1
1DVF	C	58	0	−1
1DVF	C	59	1	−1
1DVF	C	60	1	−1
1DVF	C	61	0	−1
1DVF	C	62	0	−1
1DVF	C	63	1	−1
1DVF	C	64	0	−1
1DVF	C	65	1	−1
1DVF	C	66	1	−1
1DVF	C	67	1	−1
1DVF	C	68	0	−1
1DVF	C	69	1	−1
1DVF	C	70	1	−1
1DVF	C	71	0	−1
1DVF	C	72	0	−1
1DVF	C	73	0	−1
1DVF	C	74	0	−1
1DVF	C	75	0	−1
1DVF	C	76	1	−1
1DVF	C	77	1	−1
1DVF	C	78	0	−1
1DVF	C	79	1	−1
1DVF	C	80	1	−1
1DVF	C	81	1	−1
1DVF	C	82	0	−1
1DVF	C	83	1	−1
1DVF	C	84	0	−1
1DVF	C	85	1	−1
1DVF	C	86	0	−1
1DVF	C	87	0	−1
1DVF	C	88	0	−1
1DVF	C	89	0	−1
1DVF	C	90	0	−1
1DVF	C	91	0	−1
1DVF	C	92	0	−1
1DVF	C	93	1	1
1DVF	C	94	1	1
1DVF	C	95	0	−1
1DVF	C	96	0	−1
1DVF	C	97	0	−1
1DVF	C	98	0	−1
1DVF	C	99	0	−1
1DVF	C	100	1	−1
1DVF	C	101	0	−1
1DVF	C	102	0	−1
1DVF	C	103	1	−1

1DVF	C	104	0	−1
1DVF	C	105	1	−1
1DVF	C	106	0	−1
1DVF	C	107	1	−1
1DVF	D	1	1	−1
1DVF	D	2	1	−1
1DVF	D	3	1	−1
1DVF	D	4	0	−1
1DVF	D	5	1	−1
1DVF	D	6	0	−1
1DVF	D	7	1	−1
1DVF	D	8	1	−1
1DVF	D	9	1	−1
1DVF	D	10	1	−1
1DVF	D	11	1	−1
1DVF	D	12	0	−1
1DVF	D	13	1	−1
1DVF	D	14	1	−1
1DVF	D	15	1	−1
1DVF	D	16	1	−1
1DVF	D	17	1	−1
1DVF	D	18	0	−1
1DVF	D	19	1	−1
1DVF	D	20	0	−1
1DVF	D	21	1	−1
1DVF	D	22	0	−1
1DVF	D	23	1	−1
1DVF	D	24	0	−1
1DVF	D	25	1	−1
1DVF	D	26	1	−1
1DVF	D	27	0	−1
1DVF	D	28	1	−1
1DVF	D	29	0	−1
1DVF	D	30	1	1
1DVF	D	31	1	1
1DVF	D	32	0	−1
1DVF	D	33	1	1
1DVF	D	34	0	−1
1DVF	D	35	0	−1
1DVF	D	36	0	−1
1DVF	D	37	0	−1
1DVF	D	38	0	−1
1DVF	D	39	0	−1
1DVF	D	40	0	−1
1DVF	D	41	1	−1
1DVF	D	42	1	−1
1DVF	D	43	1	−1
1DVF	D	44	0	−1
1DVF	D	45	0	−1
1DVF	D	46	1	−1
1DVF	D	47	0	−1
1DVF	D	48	0	−1

1DVF	D	49	0	−1
1DVF	D	50	0	−1
1DVF	D	51	0	−1
1DVF	D	52	0	−1
1DVF	D	53	0	−1
1DVF	D	54	1	1
1DVF	D	55	1	1
1DVF	D	56	1	−1
1DVF	D	57	1	1
1DVF	D	58	1	−1
1DVF	D	59	1	1
1DVF	D	60	0	−1
1DVF	D	61	0	−1
1DVF	D	62	1	−1
1DVF	D	63	1	−1
1DVF	D	64	0	−1
1DVF	D	65	1	−1
1DVF	D	66	1	−1
1DVF	D	67	0	−1
1DVF	D	68	0	−1
1DVF	D	69	1	−1
1DVF	D	70	0	−1
1DVF	D	71	1	−1
1DVF	D	72	1	−1
1DVF	D	73	1	−1
1DVF	D	74	1	−1
1DVF	D	75	1	−1
1DVF	D	76	1	−1
1DVF	D	77	0	−1
1DVF	D	78	0	−1
1DVF	D	79	0	−1
1DVF	D	80	0	−1
1DVF	D	81	0	−1
1DVF	D	82	1	−1
1DVF	D	83	0	−1
1DVF	D	84	1	−1
1DVF	D	85	0	−1
1DVF	D	87	1	−1
1DVF	D	88	1	−1
1DVF	D	89	1	−1
1DVF	D	90	0	−1
1DVF	D	91	0	−1
1DVF	D	92	0	−1
1DVF	D	93	0	−1
1DVF	D	94	0	−1
1DVF	D	95	0	−1
1DVF	D	96	0	−1
1DVF	D	97	0	−1
1DVF	D	98	0	−1
1DVF	D	99	1	1
1DVF	D	100	1	1
1DVF	D	101	1	1

1DVF	D	102	1	1
1DVF	D	103	0	−1
1DVF	D	104	1	1
1DVF	D	105	1	1
1DVF	D	106	1	1
1DVF	D	107	0	−1
1DVF	D	108	0	−1
1DVF	D	109	0	−1
1DVF	D	110	0	−1
1DVF	D	111	1	−1
1DVF	D	112	0	−1
1DVF	D	113	0	−1
1DVF	D	114	1	−1
1DVF	D	115	1	−1
1DVF	D	116	0	−1
1DVF	D	117	1	−1
1DVF	D	118	0	−1
1DVF	D	119	1	−1
1DVF	D	120	0	−1
1DVF	D	121	1	−1
1FC2	C	5	1	1
1FC2	C	6	1	−1
1FC2	C	7	1	−1
1FC2	C	8	1	−1
1FC2	C	9	1	1
1FC2	C	10	1	1
1FC2	C	11	1	1
1FC2	C	12	0	−1
1FC2	C	13	1	1
1FC2	C	14	1	1
1FC2	C	15	1	1
1FC2	C	16	0	−1
1FC2	C	17	1	1
1FC2	C	18	1	1
1FC2	C	19	0	−1
1FC2	C	20	1	−1
1FC2	C	21	1	−1
1FC2	C	22	0	−1
1FC2	C	23	1	−1
1FC2	C	24	1	−1
1FC2	C	25	1	−1
1FC2	C	26	1	−1
1FC2	C	27	0	−1
1FC2	C	28	1	1
1FC2	C	29	1	−1
1FC2	C	30	0	−1
1FC2	C	31	0	−1
1FC2	C	32	1	1
1FC2	C	33	1	−1
1FC2	C	34	0	−1
1FC2	C	35	1	1
1FC2	C	36	1	−1

1FC2	C	37	1	−1
1FC2	C	38	0	−1
1FC2	C	39	1	−1
1FC2	C	40	1	−1
1FC2	C	41	0	−1
1FC2	C	42	1	−1
1FC2	C	43	1	−1
1FC2	C	44	1	−1
1FC2	C	45	1	−1
1FC2	C	46	1	−1
1FC2	C	47	1	−1
1FC2	D	16	1	−1
1FC2	D	17	1	−1
1FC2	D	18	0	−1
1FC2	D	19	1	−1
1FC2	D	20	0	−1
1FC2	D	21	1	−1
1FC2	D	22	1	−1
1FC2	D	23	0	−1
1FC2	D	24	1	−1
1FC2	D	25	1	−1
1FC2	D	26	0	−1
1FC2	D	27	0	−1
1FC2	D	28	0	−1
1FC2	D	29	0	−1
1FC2	D	30	0	−1
1FC2	D	31	1	1
1FC2	D	32	1	1
1FC2	D	33	1	1
1FC2	D	34	1	−1
1FC2	D	35	0	−1
1FC2	D	36	1	−1
1FC2	D	37	0	−1
1FC2	D	38	0	−1
1FC2	D	39	0	−1
1FC2	D	40	0	−1
1FC2	D	41	0	−1
1FC2	D	42	1	−1
1FC2	D	43	1	−1
1FC2	D	44	0	−1
1FC2	D	45	1	−1
1FC2	D	46	1	−1
1FC2	D	47	1	−1
1FC2	D	48	0	−1
1FC2	D	49	1	−1
1FC2	D	50	1	−1
1FC2	D	51	0	−1
1FC2	D	52	1	−1
1FC2	D	53	0	−1
1FC2	D	54	0	−1
1FC2	D	55	0	−1
1FC2	D	56	0	−1

1FC2	D	57	0	−1
1FC2	D	58	1	−1
1FC2	D	59	1	−1
1FC2	D	60	1	−1
1FC2	D	61	1	−1
1FC2	D	62	1	−1
1FC2	D	63	1	−1
1FC2	D	64	1	−1
1FC2	D	65	1	−1
1FC2	D	66	1	−1
1FC2	D	67	0	−1
1FC2	D	68	1	−1
1FC2	D	69	1	−1
1FC2	D	70	1	−1
1FC2	D	71	1	−1
1FC2	D	72	1	−1
1FC2	D	73	1	−1
1FC2	D	74	1	−1
1FC2	D	75	1	−1
1FC2	D	76	1	−1
1FC2	D	77	0	−1
1FC2	D	78	1	−1
1FC2	D	79	1	−1
1FC2	D	80	0	−1
1FC2	D	81	0	−1
1FC2	D	82	0	−1
1FC2	D	83	0	−1
1FC2	D	84	0	−1
1FC2	D	85	0	−1
1FC2	D	86	0	−1
1FC2	D	87	1	1
1FC2	D	88	0	−1
1FC2	D	89	1	1
1FC2	D	90	0	−1
1FC2	D	91	0	−1
1FC2	D	92	0	−1
1FC2	D	93	1	1
1FC2	D	94	1	−1
1FC2	D	95	1	1
1FC2	D	96	1	−1
1FC2	D	97	0	−1
1FC2	D	98	1	−1
1FC2	D	99	0	−1
1FC2	D	100	1	−1
1FC2	D	101	0	−1
1FC2	D	102	1	−1
1FC2	D	103	0	−1
1FC2	D	104	1	−1
1FC2	D	105	1	−1
1FC2	D	106	0	−1
1FC2	D	107	1	−1
1FC2	D	108	1	−1

1FC2	D	109	1	−1
1FC2	D	110	0	−1
1FC2	D	111	1	−1
1FC2	D	112	1	−1
1FC2	D	113	1	−1
1FC2	D	114	0	−1
1FC2	D	115	1	−1
1FC2	D	116	0	−1
1FC2	D	117	1	−1
1FC2	D	118	1	−1
1FC2	D	119	1	−1
1FC2	D	120	1	−1
1FC2	D	121	1	−1
1FC2	D	122	1	−1
1FC2	D	123	1	−1
1FC2	D	124	0	−1
1FC2	D	125	1	−1
1FC2	D	126	0	−1
1FC2	D	127	1	−1
1FC2	D	128	0	−1
1FC2	D	129	1	−1
1FC2	D	130	1	−1
1FC2	D	131	0	−1
1FC2	D	132	1	−1
1FC2	D	133	1	−1
1FC2	D	134	1	−1
1FC2	D	135	0	−1
1FC2	D	136	1	−1
1FC2	D	137	1	−1
1FC2	D	138	1	−1
1FC2	D	139	1	−1
1FC2	D	140	1	−1
1FC2	D	141	0	−1
1FC2	D	142	0	−1
1FC2	D	143	0	−1
1FC2	D	144	0	−1
1FC2	D	145	0	−1
1FC2	D	146	0	−1
1FC2	D	147	0	−1
1FC2	D	148	1	−1
1FC2	D	149	0	−1
1FC2	D	150	0	−1
1FC2	D	151	1	−1
1FC2	D	152	0	−1
1FC2	D	153	1	−1
1FC2	D	154	1	−1
1FC2	D	155	0	−1
1FC2	D	156	0	−1
1FC2	D	157	0	−1
1FC2	D	158	0	−1
1FC2	D	159	0	−1
1FC2	D	160	0	−1

1FC2	D	161	0	−1
1FC2	D	162	1	−1
1FC2	D	163	1	−1
1FC2	D	164	1	−1
1FC2	D	165	1	−1
1FC2	D	166	0	−1
1FC2	D	167	1	−1
1FC2	D	168	1	−1
1FC2	D	169	1	−1
1FC2	D	170	1	−1
1FC2	D	171	1	−1
1FC2	D	172	1	−1
1FC2	D	173	1	−1
1FC2	D	174	1	−1
1FC2	D	175	1	−1
1FC2	D	176	1	−1
1FC2	D	177	1	−1
1FC2	D	178	1	−1
1FC2	D	179	1	−1
1FC2	D	180	1	−1
1FC2	D	181	0	−1
1FC2	D	182	0	−1
1FC2	D	183	1	−1
1FC2	D	184	0	−1
1FC2	D	185	1	−1
1FC2	D	186	0	−1
1FC2	D	187	1	−1
1FC2	D	188	0	−1
1FC2	D	189	1	−1
1FC2	D	190	0	−1
1FC2	D	191	1	−1
1FC2	D	192	1	−1
1FC2	D	193	1	−1
1FC2	D	194	1	−1
1FC2	D	195	0	−1
1FC2	D	196	1	−1
1FC2	D	197	1	−1
1FC2	D	198	1	−1
1FC2	D	199	0	−1
1FC2	D	200	1	−1
1FC2	D	201	0	−1
1FC2	D	202	0	−1
1FC2	D	203	0	−1
1FC2	D	204	0	−1
1FC2	D	205	0	−1
1FC2	D	206	0	−1
1FC2	D	207	0	−1
1FC2	D	208	0	−1
1FC2	D	209	0	−1
1FC2	D	210	0	−1
1FC2	D	211	1	1
1FC2	D	212	1	1

1FC2	D	213	1	1
1FC2	D	214	1	1
1FC2	D	215	1	−1
1FC2	D	216	1	−1
1FC2	D	217	1	−1
1FC2	D	218	1	−1
1FC2	D	219	0	−1
1FC2	D	220	1	−1
1FC2	D	221	1	−1
1FLE	E	1	0	−1
1FLE	E	2	0	−1
1FLE	E	3	1	−1
1FLE	E	4	1	−1
1FLE	E	5	1	−1
1FLE	E	6	1	−1
1FLE	E	7	0	−1
1FLE	E	8	1	−1
1FLE	E	9	1	−1
1FLE	E	10	1	−1
1FLE	E	11	1	−1
1FLE	E	12	0	−1
1FLE	E	13	1	−1
1FLE	E	14	0	−1
1FLE	E	15	0	−1
1FLE	E	16	0	−1
1FLE	E	17	0	−1
1FLE	E	18	0	−1
1FLE	E	19	0	−1
1FLE	E	20	1	1
1FLE	E	21	1	−1
1FLE	E	22	1	−1
1FLE	E	23	1	−1
1FLE	E	24	1	−1
1FLE	E	25	1	−1
1FLE	E	26	1	−1
1FLE	E	27	0	−1
1FLE	E	28	0	−1
1FLE	E	29	0	−1
1FLE	E	30	0	−1
1FLE	E	31	0	−1
1FLE	E	32	0	−1
1FLE	E	33	0	−1
1FLE	E	34	0	−1
1FLE	E	35	0	−1
1FLE	E	36	1	−1
1FLE	E	37	1	−1
1FLE	E	38	0	−1
1FLE	E	39	0	−1
1FLE	E	40	0	−1
1FLE	E	41	0	−1
1FLE	E	42	0	−1
1FLE	E	43	0	−1

1FLE	E	44	0	−1
1FLE	E	45	1	1
1FLE	E	46	0	−1
1FLE	E	47	0	−1
1FLE	E	48	1	1
1FLE	E	49	1	1
1FLE	E	50	1	−1
1FLE	E	51	1	−1
1FLE	E	52	1	−1
1FLE	E	53	0	−1
1FLE	E	54	0	−1
1FLE	E	55	0	−1
1FLE	E	56	0	−1
1FLE	E	57	0	−1
1FLE	E	58	0	−1
1FLE	E	59	0	−1
1FLE	E	60	0	−1
1FLE	E	61	0	−1
1FLE	E	62	0	−1
1FLE	E	63	1	−1
1FLE	E	64	1	−1
1FLE	E	65	1	−1
1FLE	E	66	0	−1
1FLE	E	67	1	−1
1FLE	E	68	0	−1
1FLE	E	69	0	−1
1FLE	E	70	0	−1
1FLE	E	71	1	−1
1FLE	E	72	0	−1
1FLE	E	73	1	−1
1FLE	E	74	0	−1
1FLE	E	75	1	−1
1FLE	E	76	1	−1
1FLE	E	77	1	−1
1FLE	E	78	0	−1
1FLE	E	79	1	−1
1FLE	E	80	0	−1
1FLE	E	81	1	−1
1FLE	E	82	1	−1
1FLE	E	83	0	−1
1FLE	E	84	1	1
1FLE	E	85	1	1
1FLE	E	86	1	1
1FLE	E	87	1	−1
1FLE	E	88	1	1
1FLE	E	89	0	−1
1FLE	E	90	1	−1
1FLE	E	91	0	−1
1FLE	E	92	1	−1
1FLE	E	93	0	−1
1FLE	E	94	0	−1
1FLE	E	95	0	−1

1FLE	E	96	0	−1
1FLE	E	97	0	−1
1FLE	E	98	1	−1
1FLE	E	99	0	−1
1FLE	E	100	1	−1
1FLE	E	101	1	−1
1FLE	E	102	1	−1
1FLE	E	103	0	−1
1FLE	E	104	1	−1
1FLE	E	105	1	−1
1FLE	E	106	1	−1
1FLE	E	107	1	−1
1FLE	E	108	1	−1
1FLE	E	109	0	−1
1FLE	E	110	1	−1
1FLE	E	111	1	−1
1FLE	E	112	0	−1
1FLE	E	113	1	−1
1FLE	E	114	1	−1
1FLE	E	115	0	−1
1FLE	E	116	1	−1
1FLE	E	117	1	−1
1FLE	E	118	1	−1
1FLE	E	119	1	−1
1FLE	E	120	1	−1
1FLE	E	121	0	−1
1FLE	E	122	1	−1
1FLE	E	123	1	−1
1FLE	E	124	1	−1
1FLE	E	125	0	−1
1FLE	E	126	1	−1
1FLE	E	127	0	−1
1FLE	E	128	1	−1
1FLE	E	129	0	−1
1FLE	E	130	0	−1
1FLE	E	131	0	−1
1FLE	E	132	0	−1
1FLE	E	133	0	−1
1FLE	E	134	0	−1
1FLE	E	135	1	−1
1FLE	E	136	1	−1
1FLE	E	137	0	−1
1FLE	E	138	1	−1
1FLE	E	139	1	−1
1FLE	E	140	1	−1
1FLE	E	141	1	1
1FLE	E	142	0	−1
1FLE	E	143	1	−1
1FLE	E	144	0	−1
1FLE	E	145	0	−1
1FLE	E	146	0	−1
1FLE	E	147	0	−1

1FLE	E	148	0	−1
1FLE	E	149	1	−1
1FLE	E	150	0	−1
1FLE	E	151	0	−1
1FLE	E	152	0	−1
1FLE	E	153	0	−1
1FLE	E	154	1	−1
1FLE	E	155	1	−1
1FLE	E	156	1	−1
1FLE	E	157	1	−1
1FLE	E	158	0	−1
1FLE	E	159	0	−1
1FLE	E	160	1	−1
1FLE	E	161	1	−1
1FLE	E	162	1	−1
1FLE	E	163	0	−1
1FLE	E	164	0	−1
1FLE	E	165	0	−1
1FLE	E	166	1	−1
1FLE	E	167	1	1
1FLE	E	168	0	−1
1FLE	E	169	1	−1
1FLE	E	170	1	−1
1FLE	E	171	0	−1
1FLE	E	172	0	−1
1FLE	E	173	0	−1
1FLE	E	174	0	−1
1FLE	E	175	0	−1
1FLE	E	176	0	−1
1FLE	E	177	0	−1
1FLE	E	178	1	−1
1FLE	E	179	0	−1
1FLE	E	180	1	−1
1FLE	E	181	1	−1
1FLE	E	182	0	−1
1FLE	E	183	0	−1
1FLE	E	184	0	−1
1FLE	E	185	1	1
1FLE	E	186	0	−1
1FLE	E	187	0	−1
1FLE	E	188	0	−1
1FLE	E	189	0	−1
1FLE	E	190	0	−1
1FLE	E	191	0	−1
1FLE	E	192	0	−1
1FLE	E	193	0	−1
1FLE	E	194	0	−1
1FLE	E	195	1	−1
1FLE	E	196	1	−1
1FLE	E	197	1	−1
1FLE	E	198	1	−1
1FLE	E	199	1	−1

1FLE	E	200	1	−1
1FLE	E	201	0	−1
1FLE	E	202	0	−1
1FLE	E	203	0	−1
1FLE	E	204	0	−1
1FLE	E	205	0	−1
1FLE	E	206	0	−1
1FLE	E	207	0	−1
1FLE	E	208	0	−1
1FLE	E	209	0	−1
1FLE	E	210	0	−1
1FLE	E	211	1	1
1FLE	E	212	1	1
1FLE	E	213	0	−1
1FLE	E	214	0	−1
1FLE	E	215	1	−1
1FLE	E	216	1	−1
1FLE	E	217	1	−1
1FLE	E	218	1	−1
1FLE	E	219	1	1
1FLE	E	220	0	−1
1FLE	E	221	0	−1
1FLE	E	222	0	−1
1FLE	E	223	0	−1
1FLE	E	224	0	−1
1FLE	E	225	0	−1
1FLE	E	226	0	−1
1FLE	E	227	0	−1
1FLE	E	228	1	−1
1FLE	E	229	0	−1
1FLE	E	230	0	−1
1FLE	E	231	1	−1
1FLE	E	232	0	−1
1FLE	E	233	0	−1
1FLE	E	234	1	−1
1FLE	E	235	1	−1
1FLE	E	236	0	−1
1FLE	E	237	0	−1
1FLE	E	238	1	−1
1FLE	E	239	1	−1
1FLE	E	240	1	−1
1FLE	I	11	1	−1
1FLE	I	12	1	−1
1FLE	I	13	1	−1
1FLE	I	14	0	−1
1FLE	I	15	1	−1
1FLE	I	16	0	−1
1FLE	I	17	0	−1
1FLE	I	18	1	1
1FLE	I	19	1	1
1FLE	I	20	1	1
1FLE	I	21	1	1

1FLE	I	22	1	1
1FLE	I	23	1	1
1FLE	I	24	1	1
1FLE	I	25	1	1
1FLE	I	26	1	1
1FLE	I	27	1	1
1FLE	I	28	0	−1
1FLE	I	29	1	1
1FLE	I	30	1	−1
1FLE	I	31	1	1
1FLE	I	32	0	−1
1FLE	I	33	1	−1
1FLE	I	34	1	−1
1FLE	I	35	0	−1
1FLE	I	36	1	−1
1FLE	I	37	1	−1
1FLE	I	38	0	−1
1FLE	I	39	1	−1
1FLE	I	40	1	−1
1FLE	I	41	1	−1
1FLE	I	42	0	−1
1FLE	I	43	0	−1
1FLE	I	44	0	−1
1FLE	I	45	0	−1
1FLE	I	46	1	1
1FLE	I	47	0	−1
1FLE	I	48	1	1
1FLE	I	49	1	1
1FLE	I	50	0	−1
1FLE	I	51	0	−1
1FLE	I	52	0	−1
1FLE	I	53	1	1
1FLE	I	54	1	1
1FLE	I	55	1	−1
1FLE	I	56	0	−1
1FLE	I	57	1	−1
1FSS	A	4	1	−1
1FSS	A	5	0	−1
1FSS	A	6	0	−1
1FSS	A	7	1	−1
1FSS	A	8	0	−1
1FSS	A	9	1	−1
1FSS	A	10	0	−1
1FSS	A	11	1	−1
1FSS	A	12	0	−1
1FSS	A	13	0	−1
1FSS	A	14	1	−1
1FSS	A	15	0	−1
1FSS	A	16	1	−1
1FSS	A	17	0	−1
1FSS	A	18	1	−1
1FSS	A	19	1	−1

1FSS	A	20	0	−1
1FSS	A	21	1	−1
1FSS	A	22	0	−1
1FSS	A	23	1	−1
1FSS	A	24	1	−1
1FSS	A	25	1	−1
1FSS	A	26	1	−1
1FSS	A	27	0	−1
1FSS	A	28	0	−1
1FSS	A	29	0	−1
1FSS	A	30	0	−1
1FSS	A	31	0	−1
1FSS	A	32	0	−1
1FSS	A	33	0	−1
1FSS	A	34	0	−1
1FSS	A	35	0	−1
1FSS	A	36	0	−1
1FSS	A	37	1	−1
1FSS	A	38	0	−1
1FSS	A	39	0	−1
1FSS	A	40	1	−1
1FSS	A	41	1	−1
1FSS	A	42	1	−1
1FSS	A	43	1	−1
1FSS	A	44	0	−1
1FSS	A	45	0	−1
1FSS	A	46	1	−1
1FSS	A	47	1	−1
1FSS	A	48	0	−1
1FSS	A	49	1	−1
1FSS	A	50	1	−1
1FSS	A	51	0	−1
1FSS	A	52	1	−1
1FSS	A	53	1	−1
1FSS	A	54	0	−1
1FSS	A	55	1	−1
1FSS	A	56	1	−1
1FSS	A	57	1	−1
1FSS	A	58	1	−1
1FSS	A	59	1	−1
1FSS	A	60	0	−1
1FSS	A	61	0	−1
1FSS	A	62	1	−1
1FSS	A	63	0	−1
1FSS	A	64	0	−1
1FSS	A	65	1	−1
1FSS	A	66	0	−1
1FSS	A	67	0	−1
1FSS	A	68	0	−1
1FSS	A	69	0	−1
1FSS	A	70	1	1
1FSS	A	71	1	1

1FSS	A	72	0	−1
1FSS	A	73	1	1
1FSS	A	74	1	1
1FSS	A	75	0	−1
1FSS	A	76	1	1
1FSS	A	77	1	−1
1FSS	A	78	0	−1
1FSS	A	79	1	−1
1FSS	A	80	0	−1
1FSS	A	81	0	−1
1FSS	A	82	1	1
1FSS	A	83	1	−1
1FSS	A	84	1	−1
1FSS	A	85	0	−1
1FSS	A	86	0	−1
1FSS	A	87	1	1
1FSS	A	88	1	−1
1FSS	A	89	1	−1
1FSS	A	90	1	1
1FSS	A	91	1	−1
1FSS	A	92	0	−1
1FSS	A	93	1	−1
1FSS	A	94	0	−1
1FSS	A	95	0	−1
1FSS	A	96	0	−1
1FSS	A	97	0	−1
1FSS	A	98	0	−1
1FSS	A	99	0	−1
1FSS	A	100	0	−1
1FSS	A	101	0	−1
1FSS	A	102	0	−1
1FSS	A	103	1	−1
1FSS	A	104	1	−1
1FSS	A	105	1	−1
1FSS	A	106	0	−1
1FSS	A	107	1	−1
1FSS	A	108	1	−1
1FSS	A	109	0	−1
1FSS	A	110	0	−1
1FSS	A	111	0	−1
1FSS	A	112	0	−1
1FSS	A	113	0	−1
1FSS	A	114	0	−1
1FSS	A	115	0	−1
1FSS	A	116	0	−1
1FSS	A	117	0	−1
1FSS	A	118	0	−1
1FSS	A	119	0	−1
1FSS	A	120	0	−1
1FSS	A	121	0	−1
1FSS	A	122	0	−1
1FSS	A	123	0	−1

1FSS	A	124	0	−1
1FSS	A	125	0	−1
1FSS	A	126	0	−1
1FSS	A	127	0	−1
1FSS	A	128	1	−1
1FSS	A	129	0	−1
1FSS	A	130	0	−1
1FSS	A	131	0	−1
1FSS	A	132	0	−1
1FSS	A	133	0	−1
1FSS	A	134	0	−1
1FSS	A	135	0	−1
1FSS	A	136	0	−1
1FSS	A	137	1	−1
1FSS	A	138	0	−1
1FSS	A	139	0	−1
1FSS	A	140	1	−1
1FSS	A	141	0	−1
1FSS	A	142	0	−1
1FSS	A	143	0	−1
1FSS	A	144	0	−1
1FSS	A	145	0	−1
1FSS	A	146	0	−1
1FSS	A	147	0	−1
1FSS	A	148	0	−1
1FSS	A	149	0	−1
1FSS	A	150	0	−1
1FSS	A	151	0	−1
1FSS	A	152	0	−1
1FSS	A	153	0	−1
1FSS	A	154	0	−1
1FSS	A	155	0	−1
1FSS	A	156	0	−1
1FSS	A	157	0	−1
1FSS	A	158	0	−1
1FSS	A	159	1	−1
1FSS	A	160	1	−1
1FSS	A	161	0	−1
1FSS	A	162	1	−1
1FSS	A	163	0	−1
1FSS	A	164	0	−1
1FSS	A	165	0	−1
1FSS	A	166	0	−1
1FSS	A	167	0	−1
1FSS	A	168	0	−1
1FSS	A	169	0	−1
1FSS	A	170	0	−1
1FSS	A	171	0	−1
1FSS	A	172	0	−1
1FSS	A	173	0	−1
1FSS	A	174	1	−1
1FSS	A	175	0	−1

1FSS	A	176	0	−1
1FSS	A	177	0	−1
1FSS	A	178	1	−1
1FSS	A	179	0	−1
1FSS	A	180	0	−1
1FSS	A	181	1	−1
1FSS	A	182	1	−1
1FSS	A	183	0	−1
1FSS	A	184	0	−1
1FSS	A	185	1	−1
1FSS	A	186	1	−1
1FSS	A	187	0	−1
1FSS	A	188	0	−1
1FSS	A	189	0	−1
1FSS	A	190	0	−1
1FSS	A	191	0	−1
1FSS	A	192	1	−1
1FSS	A	193	0	−1
1FSS	A	194	0	−1
1FSS	A	195	0	−1
1FSS	A	196	0	−1
1FSS	A	197	0	−1
1FSS	A	198	0	−1
1FSS	A	199	0	−1
1FSS	A	200	0	−1
1FSS	A	201	0	−1
1FSS	A	202	0	−1
1FSS	A	203	0	−1
1FSS	A	204	0	−1
1FSS	A	205	0	−1
1FSS	A	206	0	−1
1FSS	A	207	0	−1
1FSS	A	208	0	−1
1FSS	A	209	0	−1
1FSS	A	210	0	−1
1FSS	A	211	0	−1
1FSS	A	212	0	−1
1FSS	A	213	1	−1
1FSS	A	214	1	−1
1FSS	A	215	0	−1
1FSS	A	216	0	−1
1FSS	A	217	1	−1
1FSS	A	218	0	−1
1FSS	A	219	0	−1
1FSS	A	220	1	−1
1FSS	A	221	0	−1
1FSS	A	222	0	−1
1FSS	A	223	0	−1
1FSS	A	224	0	−1
1FSS	A	225	0	−1
1FSS	A	226	0	−1
1FSS	A	227	0	−1

1FSS	A	228	0	−1
1FSS	A	229	0	−1
1FSS	A	230	0	−1
1FSS	A	231	0	−1
1FSS	A	232	1	−1
1FSS	A	233	0	−1
1FSS	A	234	0	−1
1FSS	A	235	0	−1
1FSS	A	236	0	−1
1FSS	A	237	1	−1
1FSS	A	238	1	−1
1FSS	A	239	1	−1
1FSS	A	240	0	−1
1FSS	A	241	0	−1
1FSS	A	242	1	−1
1FSS	A	243	1	−1
1FSS	A	244	0	−1
1FSS	A	245	0	−1
1FSS	A	246	1	−1
1FSS	A	247	1	−1
1FSS	A	248	0	−1
1FSS	A	249	0	−1
1FSS	A	250	1	−1
1FSS	A	251	0	−1
1FSS	A	252	0	−1
1FSS	A	253	1	−1
1FSS	A	254	0	−1
1FSS	A	255	1	−1
1FSS	A	256	1	−1
1FSS	A	257	1	−1
1FSS	A	258	1	−1
1FSS	A	259	0	−1
1FSS	A	260	1	−1
1FSS	A	261	1	−1
1FSS	A	262	0	−1
1FSS	A	263	0	−1
1FSS	A	264	1	−1
1FSS	A	265	0	−1
1FSS	A	266	0	−1
1FSS	A	267	0	−1
1FSS	A	268	1	−1
1FSS	A	269	1	−1
1FSS	A	270	1	−1
1FSS	A	271	0	−1
1FSS	A	272	1	1
1FSS	A	273	1	−1
1FSS	A	274	0	−1
1FSS	A	275	0	−1
1FSS	A	276	1	1
1FSS	A	277	0	−1
1FSS	A	278	0	−1
1FSS	A	279	1	1

1FSS	A	280	1	1
1FSS	A	281	0	−1
1FSS	A	282	1	1
1FSS	A	283	1	−1
1FSS	A	284	1	1
1FSS	A	285	1	1
1FSS	A	286	1	1
1FSS	A	287	0	−1
1FSS	A	288	0	−1
1FSS	A	289	0	−1
1FSS	A	290	0	−1
1FSS	A	291	0	−1
1FSS	A	292	0	−1
1FSS	A	293	0	−1
1FSS	A	294	0	−1
1FSS	A	295	0	−1
1FSS	A	296	0	−1
1FSS	A	297	0	−1
1FSS	A	298	1	−1
1FSS	A	299	1	−1
1FSS	A	300	0	−1
1FSS	A	301	0	−1
1FSS	A	302	1	−1
1FSS	A	303	1	−1
1FSS	A	304	1	−1
1FSS	A	305	0	−1
1FSS	A	306	1	−1
1FSS	A	307	1	−1
1FSS	A	308	0	−1
1FSS	A	309	0	−1
1FSS	A	310	1	−1
1FSS	A	311	1	−1
1FSS	A	312	0	−1
1FSS	A	313	1	−1
1FSS	A	314	0	−1
1FSS	A	315	0	−1
1FSS	A	316	1	−1
1FSS	A	317	0	−1
1FSS	A	318	0	−1
1FSS	A	319	0	−1
1FSS	A	320	0	−1
1FSS	A	321	0	−1
1FSS	A	322	0	−1
1FSS	A	323	0	−1
1FSS	A	324	0	−1
1FSS	A	325	1	−1
1FSS	A	326	0	−1
1FSS	A	327	0	−1
1FSS	A	328	0	−1
1FSS	A	329	0	−1
1FSS	A	330	0	−1
1FSS	A	331	0	−1

1FSS	A	332	0	−1
1FSS	A	333	0	−1
1FSS	A	334	1	1
1FSS	A	335	1	1
1FSS	A	336	0	−1
1FSS	A	337	1	−1
1FSS	A	338	1	−1
1FSS	A	339	0	−1
1FSS	A	340	1	−1
1FSS	A	341	0	−1
1FSS	A	342	1	1
1FSS	A	343	1	−1
1FSS	A	344	1	−1
1FSS	A	345	0	−1
1FSS	A	346	1	−1
1FSS	A	347	0	−1
1FSS	A	348	1	−1
1FSS	A	349	0	−1
1FSS	A	350	1	−1
1FSS	A	351	1	−1
1FSS	A	352	0	−1
1FSS	A	353	1	−1
1FSS	A	354	1	−1
1FSS	A	355	0	−1
1FSS	A	356	0	−1
1FSS	A	357	1	−1
1FSS	A	358	0	−1
1FSS	A	359	0	−1
1FSS	A	360	0	−1
1FSS	A	361	0	−1
1FSS	A	362	1	−1
1FSS	A	363	0	−1
1FSS	A	364	1	−1
1FSS	A	365	1	−1
1FSS	A	366	1	−1
1FSS	A	367	0	−1
1FSS	A	368	0	−1
1FSS	A	369	1	−1
1FSS	A	370	0	−1
1FSS	A	371	0	−1
1FSS	A	372	0	−1
1FSS	A	373	1	−1
1FSS	A	374	1	−1
1FSS	A	375	0	−1
1FSS	A	376	0	−1
1FSS	A	377	1	−1
1FSS	A	378	1	−1
1FSS	A	379	1	−1
1FSS	A	380	1	−1
1FSS	A	381	1	−1
1FSS	A	382	1	−1
1FSS	A	383	1	−1

1FSS	A	384	0	−1
1FSS	A	385	1	−1
1FSS	A	386	1	−1
1FSS	A	387	0	−1
1FSS	A	388	0	−1
1FSS	A	389	1	−1
1FSS	A	390	0	−1
1FSS	A	391	0	−1
1FSS	A	392	0	−1
1FSS	A	393	1	−1
1FSS	A	394	0	−1
1FSS	A	395	0	−1
1FSS	A	396	0	−1
1FSS	A	397	0	−1
1FSS	A	398	0	−1
1FSS	A	399	0	−1
1FSS	A	400	0	−1
1FSS	A	401	0	−1
1FSS	A	402	0	−1
1FSS	A	403	0	−1
1FSS	A	404	0	−1
1FSS	A	405	0	−1
1FSS	A	406	0	−1
1FSS	A	407	0	−1
1FSS	A	408	0	−1
1FSS	A	409	1	−1
1FSS	A	410	0	−1
1FSS	A	411	0	−1
1FSS	A	412	0	−1
1FSS	A	413	1	−1
1FSS	A	414	1	−1
1FSS	A	415	1	−1
1FSS	A	416	1	−1
1FSS	A	417	1	−1
1FSS	A	418	0	−1
1FSS	A	419	0	−1
1FSS	A	420	0	−1
1FSS	A	421	0	−1
1FSS	A	422	0	−1
1FSS	A	423	0	−1
1FSS	A	424	0	−1
1FSS	A	425	0	−1
1FSS	A	426	1	−1
1FSS	A	427	0	−1
1FSS	A	428	1	−1
1FSS	A	429	1	−1
1FSS	A	430	0	−1
1FSS	A	431	1	−1
1FSS	A	432	0	−1
1FSS	A	433	0	−1
1FSS	A	434	1	−1
1FSS	A	435	0	−1

1FSS	A	436	0	−1
1FSS	A	437	0	−1
1FSS	A	438	0	−1
1FSS	A	439	0	−1
1FSS	A	440	0	−1
1FSS	A	441	0	−1
1FSS	A	442	0	−1
1FSS	A	443	0	−1
1FSS	A	444	0	−1
1FSS	A	445	0	−1
1FSS	A	446	0	−1
1FSS	A	447	0	−1
1FSS	A	448	0	−1
1FSS	A	449	0	−1
1FSS	A	450	0	−1
1FSS	A	451	0	−1
1FSS	A	452	0	−1
1FSS	A	453	0	−1
1FSS	A	454	1	−1
1FSS	A	455	1	−1
1FSS	A	456	1	−1
1FSS	A	457	1	−1
1FSS	A	458	0	−1
1FSS	A	459	1	−1
1FSS	A	460	1	−1
1FSS	A	461	1	−1
1FSS	A	462	0	−1
1FSS	A	463	1	−1
1FSS	A	464	1	−1
1FSS	A	465	0	−1
1FSS	A	466	0	−1
1FSS	A	467	1	−1
1FSS	A	468	1	−1
1FSS	A	469	0	−1
1FSS	A	470	0	−1
1FSS	A	471	1	−1
1FSS	A	472	0	−1
1FSS	A	473	0	−1
1FSS	A	474	0	−1
1FSS	A	475	0	−1
1FSS	A	476	0	−1
1FSS	A	477	0	−1
1FSS	A	478	1	−1
1FSS	A	479	1	−1
1FSS	A	480	0	−1
1FSS	A	481	1	−1
1FSS	A	482	0	−1
1FSS	A	483	0	−1
1FSS	A	484	1	−1
1FSS	A	485	1	−1
1FSS	A	486	1	−1
1FSS	A	487	1	−1

1FSS	A	488	1	−1
1FSS	A	489	1	−1
1FSS	A	490	1	−1
1FSS	A	491	1	−1
1FSS	A	492	0	−1
1FSS	A	493	0	−1
1FSS	A	494	1	−1
1FSS	A	495	0	−1
1FSS	A	496	1	−1
1FSS	A	497	1	−1
1FSS	A	498	1	−1
1FSS	A	499	1	−1
1FSS	A	500	0	−1
1FSS	A	501	1	−1
1FSS	A	502	0	−1
1FSS	A	503	0	−1
1FSS	A	504	0	−1
1FSS	A	505	0	−1
1FSS	A	506	0	−1
1FSS	A	507	0	−1
1FSS	A	508	1	−1
1FSS	A	509	1	−1
1FSS	A	510	1	−1
1FSS	A	511	1	−1
1FSS	A	512	1	−1
1FSS	A	513	1	−1
1FSS	A	514	1	−1
1FSS	A	515	1	−1
1FSS	A	516	0	−1
1FSS	A	517	1	−1
1FSS	A	518	1	−1
1FSS	A	519	1	−1
1FSS	A	520	0	−1
1FSS	A	521	0	−1
1FSS	A	522	1	−1
1FSS	A	523	0	−1
1FSS	A	524	0	−1
1FSS	A	525	1	−1
1FSS	A	526	1	−1
1FSS	A	527	1	−1
1FSS	A	528	0	−1
1FSS	A	529	1	−1
1FSS	A	530	1	−1
1FSS	A	531	0	−1
1FSS	A	532	0	−1
1FSS	A	533	1	−1
1FSS	A	534	1	−1
1FSS	A	535	1	−1
1FSS	B	1	1	−1
1FSS	B	2	1	−1
1FSS	B	3	0	−1
1FSS	B	4	0	−1

1FSS	B	5	0	−1
1FSS	B	6	0	−1
1FSS	B	7	1	1
1FSS	B	8	1	1
1FSS	B	9	1	1
1FSS	B	10	1	1
1FSS	B	11	1	1
1FSS	B	12	1	1
1FSS	B	13	1	−1
1FSS	B	14	1	−1
1FSS	B	15	1	−1
1FSS	B	16	1	−1
1FSS	B	17	0	−1
1FSS	B	18	1	−1
1FSS	B	19	1	−1
1FSS	B	20	1	−1
1FSS	B	21	0	−1
1FSS	B	22	0	−1
1FSS	B	23	0	−1
1FSS	B	24	0	−1
1FSS	B	25	1	1
1FSS	B	26	0	−1
1FSS	B	27	1	1
1FSS	B	28	1	−1
1FSS	B	29	1	1
1FSS	B	30	1	1
1FSS	B	31	1	1
1FSS	B	32	1	1
1FSS	B	33	1	1
1FSS	B	34	1	1
1FSS	B	35	1	1
1FSS	B	36	0	−1
1FSS	B	37	0	−1
1FSS	B	38	0	−1
1FSS	B	39	1	−1
1FSS	B	40	0	−1
1FSS	B	41	1	−1
1FSS	B	42	1	−1
1FSS	B	43	1	−1
1FSS	B	44	1	−1
1FSS	B	45	1	−1
1FSS	B	46	1	−1
1FSS	B	47	1	1
1FSS	B	48	1	−1
1FSS	B	49	1	−1
1FSS	B	50	1	−1
1FSS	B	51	1	−1
1FSS	B	52	1	−1
1FSS	B	53	0	−1
1FSS	B	54	1	−1
1FSS	B	55	1	−1
1FSS	B	56	1	−1

1FSS	B	57	1	−1
1FSS	B	58	1	−1
1FSS	B	59	0	−1
1FSS	B	60	0	−1
1FSS	B	61	1	1
1GG2	A	4	1	1
1GG2	A	5	1	−1
1GG2	A	6	1	−1
1GG2	A	7	1	−1
1GG2	A	8	1	−1
1GG2	A	9	1	−1
1GG2	A	10	1	−1
1GG2	A	11	1	1
1GG2	A	12	1	1
1GG2	A	13	1	−1
1GG2	A	14	1	1
1GG2	A	15	1	1
1GG2	A	16	1	−1
1GG2	A	17	1	−1
1GG2	A	18	1	1
1GG2	A	19	1	1
1GG2	A	20	1	−1
1GG2	A	21	1	−1
1GG2	A	22	1	1
1GG2	A	23	1	1
1GG2	A	24	1	−1
1GG2	A	25	1	1
1GG2	A	26	1	1
1GG2	A	27	1	−1
1GG2	A	28	1	−1
1GG2	A	29	1	1
1GG2	A	30	1	−1
1GG2	A	31	1	−1
1GG2	A	32	0	−1
1GG2	A	33	0	−1
1GG2	A	34	1	1
1GG2	A	35	0	−1
1GG2	A	36	0	−1
1GG2	A	37	0	−1
1GG2	A	38	0	−1
1GG2	A	39	0	−1
1GG2	A	40	1	−1
1GG2	A	41	1	−1
1GG2	A	42	0	−1
1GG2	A	43	0	−1
1GG2	A	44	0	−1
1GG2	A	45	0	−1
1GG2	A	46	1	−1
1GG2	A	47	0	−1
1GG2	A	48	0	−1
1GG2	A	49	0	−1
1GG2	A	50	0	−1

1GG2	A	51	0	−1
1GG2	A	52	0	−1
1GG2	A	53	0	−1
1GG2	A	54	0	−1
1GG2	A	55	0	−1
1GG2	A	56	0	−1
1GG2	A	57	1	−1
1GG2	A	58	1	−1
1GG2	A	59	0	−1
1GG2	A	60	0	−1
1GG2	A	61	1	−1
1GG2	A	62	1	−1
1GG2	A	63	1	−1
1GG2	A	64	1	−1
1GG2	A	65	0	−1
1GG2	A	66	1	−1
1GG2	A	67	1	−1
1GG2	A	68	0	−1
1GG2	A	69	0	−1
1GG2	A	70	0	−1
1GG2	A	71	0	−1
1GG2	A	72	0	−1
1GG2	A	73	0	−1
1GG2	A	74	1	−1
1GG2	A	75	0	−1
1GG2	A	76	0	−1
1GG2	A	77	0	−1
1GG2	A	78	1	−1
1GG2	A	79	0	−1
1GG2	A	80	0	−1
1GG2	A	81	0	−1
1GG2	A	82	0	−1
1GG2	A	83	0	−1
1GG2	A	84	0	−1
1GG2	A	85	1	−1
1GG2	A	86	0	−1
1GG2	A	87	0	−1
1GG2	A	88	1	−1
1GG2	A	89	1	−1
1GG2	A	90	0	−1
1GG2	A	91	1	−1
1GG2	A	92	0	−1
1GG2	A	93	1	−1
1GG2	A	94	0	−1
1GG2	A	95	1	−1
1GG2	A	96	1	−1
1GG2	A	97	1	−1
1GG2	A	98	1	−1
1GG2	A	99	0	−1
1GG2	A	100	1	−1
1GG2	A	101	1	−1
1GG2	A	102	0	−1

1GG2	A	103	0	−1
1GG2	A	104	1	−1
1GG2	A	105	0	−1
1GG2	A	106	0	−1
1GG2	A	107	1	−1
1GG2	A	108	1	−1
1GG2	A	109	0	−1
1GG2	A	110	0	−1
1GG2	A	111	1	−1
1GG2	A	112	1	−1
1GG2	A	113	1	−1
1GG2	A	114	1	−1
1GG2	A	115	1	−1
1GG2	A	116	0	−1
1GG2	A	117	1	−1
1GG2	A	118	0	−1
1GG2	A	119	1	−1
1GG2	A	120	1	−1
1GG2	A	121	1	−1
1GG2	A	122	0	−1
1GG2	A	123	0	−1
1GG2	A	124	0	−1
1GG2	A	125	0	−1
1GG2	A	126	0	−1
1GG2	A	127	1	−1
1GG2	A	128	1	−1
1GG2	A	129	0	−1
1GG2	A	130	0	−1
1GG2	A	131	1	−1
1GG2	A	132	0	−1
1GG2	A	133	1	−1
1GG2	A	134	0	−1
1GG2	A	135	0	−1
1GG2	A	136	1	−1
1GG2	A	137	1	−1
1GG2	A	138	0	−1
1GG2	A	139	0	−1
1GG2	A	140	1	−1
1GG2	A	141	1	−1
1GG2	A	142	1	−1
1GG2	A	143	1	−1
1GG2	A	144	1	−1
1GG2	A	145	0	−1
1GG2	A	146	1	−1
1GG2	A	147	0	−1
1GG2	A	148	0	−1
1GG2	A	149	1	−1
1GG2	A	150	0	−1
1GG2	A	151	0	−1
1GG2	A	152	0	−1
1GG2	A	153	0	−1
1GG2	A	154	0	−1

1GG2	A	155	0	−1
1GG2	A	156	1	−1
1GG2	A	157	1	−1
1GG2	A	158	0	−1
1GG2	A	159	1	−1
1GG2	A	160	1	−1
1GG2	A	161	0	−1
1GG2	A	162	0	−1
1GG2	A	163	1	−1
1GG2	A	164	1	−1
1GG2	A	165	1	−1
1GG2	A	166	0	−1
1GG2	A	167	1	−1
1GG2	A	168	0	−1
1GG2	A	169	1	−1
1GG2	A	170	1	−1
1GG2	A	171	1	−1
1GG2	A	172	0	−1
1GG2	A	173	0	−1
1GG2	A	174	0	−1
1GG2	A	175	0	−1
1GG2	A	176	0	−1
1GG2	A	177	1	−1
1GG2	A	178	1	−1
1GG2	A	179	1	−1
1GG2	A	180	1	−1
1GG2	A	181	1	1
1GG2	A	182	1	1
1GG2	A	183	1	1
1GG2	A	184	1	−1
1GG2	A	185	1	1
1GG2	A	186	0	−1
1GG2	A	187	1	−1
1GG2	A	188	0	−1
1GG2	A	189	1	−1
1GG2	A	190	0	−1
1GG2	A	191	1	−1
1GG2	A	192	1	−1
1GG2	A	193	0	−1
1GG2	A	194	1	−1
1GG2	A	195	0	−1
1GG2	A	196	0	−1
1GG2	A	197	0	−1
1GG2	A	198	0	−1
1GG2	A	199	0	−1
1GG2	A	200	0	−1
1GG2	A	201	1	−1
1GG2	A	202	1	−1
1GG2	A	203	1	1
1GG2	A	204	1	1
1GG2	A	205	1	1
1GG2	A	206	1	1

1GG2	A	207	1	−1
1GG2	A	208	1	1
1GG2	A	209	1	1
1GG2	A	210	0	−1
1GG2	A	211	0	−1
1GG2	A	212	1	1
1GG2	A	213	1	1
1GG2	A	214	0	−1
1GG2	A	215	1	1
1GG2	A	216	1	−1
1GG2	A	217	0	−1
1GG2	A	218	0	−1
1GG2	A	219	0	−1
1GG2	A	220	0	−1
1GG2	A	221	0	−1
1GG2	A	222	0	−1
1GG2	A	223	0	−1
1GG2	A	224	0	−1
1GG2	A	225	0	−1
1GG2	A	226	0	−1
1GG2	A	227	0	−1
1GG2	A	228	0	−1
1GG2	A	229	0	−1
1GG2	A	230	0	−1
1GG2	A	231	0	−1
1GG2	A	232	1	−1
1GG2	A	233	0	−1
1GG2	A	234	1	−1
1GG2	A	235	1	−1
1GG2	A	236	1	−1
1GG2	A	237	1	−1
1GG2	A	238	1	−1
1GG2	A	239	1	−1
1GG2	A	240	0	−1
1GG2	A	241	1	−1
1GG2	A	242	0	−1
1GG2	A	243	1	−1
1GG2	A	244	1	−1
1GG2	A	245	0	−1
1GG2	A	246	0	−1
1GG2	A	247	1	−1
1GG2	A	248	0	−1
1GG2	A	249	0	−1
1GG2	A	250	1	−1
1GG2	A	251	1	−1
1GG2	A	252	0	−1
1GG2	A	253	0	−1
1GG2	A	254	0	−1
1GG2	A	255	0	−1
1GG2	A	256	1	−1
1GG2	A	257	1	1
1GG2	A	258	0	−1

1GG2	A	259	1	−1
1GG2	A	260	1	−1
1GG2	A	261	0	−1
1GG2	A	262	0	−1
1GG2	A	263	0	−1
1GG2	A	264	0	−1
1GG2	A	265	0	−1
1GG2	A	266	0	−1
1GG2	A	267	0	−1
1GG2	A	268	0	−1
1GG2	A	269	0	−1
1GG2	A	270	1	−1
1GG2	A	271	1	−1
1GG2	A	272	1	−1
1GG2	A	273	0	−1
1GG2	A	274	1	−1
1GG2	A	275	1	−1
1GG2	A	276	0	−1
1GG2	A	277	0	−1
1GG2	A	278	1	−1
1GG2	A	279	1	−1
1GG2	A	280	0	−1
1GG2	A	281	1	−1
1GG2	A	282	0	−1
1GG2	A	283	1	−1
1GG2	A	284	1	−1
1GG2	A	285	0	−1
1GG2	A	286	0	−1
1GG2	A	287	1	−1
1GG2	A	288	1	−1
1GG2	A	289	0	−1
1GG2	A	290	1	−1
1GG2	A	291	0	−1
1GG2	A	292	1	−1
1GG2	A	293	1	−1
1GG2	A	294	1	−1
1GG2	A	295	0	−1
1GG2	A	296	1	−1
1GG2	A	297	1	−1
1GG2	A	298	0	−1
1GG2	A	299	0	−1
1GG2	A	300	1	−1
1GG2	A	301	1	−1
1GG2	A	302	0	−1
1GG2	A	303	1	−1
1GG2	A	304	1	−1
1GG2	A	305	0	−1
1GG2	A	306	0	−1
1GG2	A	307	0	−1
1GG2	A	308	1	−1
1GG2	A	309	0	−1
1GG2	A	310	0	−1

1GG2	A	311	1	−1
1GG2	A	312	1	−1
1GG2	A	313	1	−1
1GG2	A	314	1	−1
1GG2	A	315	1	−1
1GG2	A	316	0	−1
1GG2	A	317	1	−1
1GG2	A	318	0	−1
1GG2	A	319	0	−1
1GG2	A	320	0	−1
1GG2	A	321	0	−1
1GG2	A	322	0	−1
1GG2	A	323	0	−1
1GG2	A	324	0	−1
1GG2	A	325	0	−1
1GG2	A	326	0	−1
1GG2	A	327	1	−1
1GG2	A	328	1	−1
1GG2	A	329	1	−1
1GG2	A	330	0	−1
1GG2	A	331	0	−1
1GG2	A	332	1	−1
1GG2	A	333	1	−1
1GG2	A	334	0	−1
1GG2	A	335	0	−1
1GG2	A	336	0	−1
1GG2	A	337	0	−1
1GG2	A	338	0	−1
1GG2	A	339	0	−1
1GG2	A	340	1	−1
1GG2	A	341	0	−1
1GG2	A	342	0	−1
1GG2	A	343	1	−1
1GG2	A	344	1	−1
1GG2	A	345	1	−1
1GG2	A	346	1	−1
1GG2	A	347	1	−1
1GG2	B	2	1	−1
1GG2	B	3	1	1
1GG2	B	4	1	1
1GG2	B	5	1	−1
1GG2	B	6	1	−1
1GG2	B	7	1	1
1GG2	B	8	1	1
1GG2	B	9	1	−1
1GG2	B	10	1	1
1GG2	B	11	1	1
1GG2	B	12	1	−1
1GG2	B	13	1	−1
1GG2	B	14	1	1
1GG2	B	15	1	1
1GG2	B	16	1	−1

1GG2	B	17	1	1
1GG2	B	18	1	1
1GG2	B	19	1	−1
1GG2	B	20	1	−1
1GG2	B	21	1	1
1GG2	B	22	1	1
1GG2	B	23	1	−1
1GG2	B	24	1	1
1GG2	B	25	1	1
1GG2	B	26	1	1
1GG2	B	27	1	1
1GG2	B	28	1	1
1GG2	B	29	1	1
1GG2	B	30	0	−1
1GG2	B	31	1	−1
1GG2	B	32	1	−1
1GG2	B	33	1	1
1GG2	B	34	0	−1
1GG2	B	35	1	−1
1GG2	B	36	1	−1
1GG2	B	37	1	1
1GG2	B	38	1	−1
1GG2	B	39	1	−1
1GG2	B	40	0	−1
1GG2	B	41	1	−1
1GG2	B	42	1	−1
1GG2	B	43	1	1
1GG2	B	44	1	−1
1GG2	B	45	1	1
1GG2	B	46	1	−1
1GG2	B	47	1	−1
1GG2	B	48	1	1
1GG2	B	49	1	1
1GG2	B	50	1	−1
1GG2	B	51	0	−1
1GG2	B	52	1	1
1GG2	B	53	1	1
1GG2	B	54	0	−1
1GG2	B	55	1	1
1GG2	B	56	1	−1
1GG2	B	57	1	1
1GG2	B	58	0	−1
1GG2	B	59	1	1
1GG2	B	60	0	−1
1GG2	B	61	0	−1
1GG2	B	62	1	−1
1GG2	B	63	0	−1
1GG2	B	64	0	−1
1GG2	B	65	1	−1
1GG2	B	66	1	−1
1GG2	B	67	0	−1
1GG2	B	68	1	−1

1GG2	B	69	0	−1
1GG2	B	70	0	−1
1GG2	B	71	0	−1
1GG2	B	72	0	−1
1GG2	B	73	0	−1
1GG2	B	74	0	−1
1GG2	B	75	0	−1
1GG2	B	76	0	−1
1GG2	B	77	0	−1
1GG2	B	78	1	1
1GG2	B	79	0	−1
1GG2	B	80	0	−1
1GG2	B	81	0	−1
1GG2	B	82	0	−1
1GG2	B	83	0	−1
1GG2	B	84	0	−1
1GG2	B	85	1	1
1GG2	B	86	1	−1
1GG2	B	87	1	−1
1GG2	B	88	1	1
1GG2	B	89	1	1
1GG2	B	90	1	1
1GG2	B	91	0	−1
1GG2	B	92	1	1
1GG2	B	93	0	−1
1GG2	B	94	1	−1
1GG2	B	95	0	−1
1GG2	B	96	1	−1
1GG2	B	97	0	−1
1GG2	B	98	1	1
1GG2	B	99	1	1
1GG2	B	100	0	−1
1GG2	B	101	1	1
1GG2	B	102	0	−1
1GG2	B	103	0	−1
1GG2	B	104	0	−1
1GG2	B	105	0	−1
1GG2	B	106	0	−1
1GG2	B	107	1	−1
1GG2	B	108	1	−1
1GG2	B	109	0	−1
1GG2	B	110	1	−1
1GG2	B	111	0	−1
1GG2	B	112	0	−1
1GG2	B	113	0	−1
1GG2	B	114	0	−1
1GG2	B	115	0	−1
1GG2	B	116	0	−1
1GG2	B	117	1	1
1GG2	B	118	0	−1
1GG2	B	119	1	1
1GG2	B	120	0	−1

1GG2	B	121	0	−1
1GG2	B	122	0	−1
1GG2	B	123	0	−1
1GG2	B	124	0	−1
1GG2	B	125	0	−1
1GG2	B	126	0	−1
1GG2	B	127	1	−1
1GG2	B	128	0	−1
1GG2	B	129	1	−1
1GG2	B	130	1	−1
1GG2	B	131	1	−1
1GG2	B	132	1	1
1GG2	B	133	0	−1
1GG2	B	134	1	−1
1GG2	B	135	1	−1
1GG2	B	136	1	−1
1GG2	B	137	1	−1
1GG2	B	138	1	−1
1GG2	B	139	0	−1
1GG2	B	140	1	−1
1GG2	B	141	1	−1
1GG2	B	142	0	−1
1GG2	B	143	1	1
1GG2	B	144	0	−1
1GG2	B	145	0	−1
1GG2	B	146	0	−1
1GG2	B	147	0	−1
1GG2	B	148	0	−1
1GG2	B	149	0	−1
1GG2	B	150	0	−1
1GG2	B	151	0	−1
1GG2	B	152	0	−1
1GG2	B	153	1	−1
1GG2	B	154	0	−1
1GG2	B	155	1	−1
1GG2	B	156	0	−1
1GG2	B	157	0	−1
1GG2	B	158	0	−1
1GG2	B	159	0	−1
1GG2	B	160	0	−1
1GG2	B	161	0	−1
1GG2	B	162	0	−1
1GG2	B	163	0	−1
1GG2	B	164	1	−1
1GG2	B	165	0	−1
1GG2	B	166	0	−1
1GG2	B	167	0	−1
1GG2	B	168	0	−1
1GG2	B	169	0	−1
1GG2	B	170	1	−1
1GG2	B	171	0	−1
1GG2	B	172	1	−1

1GG2	B	173	1	−1
1GG2	B	174	1	−1
1GG2	B	175	1	−1
1GG2	B	176	1	−1
1GG2	B	177	1	−1
1GG2	B	178	1	−1
1GG2	B	179	1	−1
1GG2	B	180	0	−1
1GG2	B	181	1	−1
1GG2	B	182	1	−1
1GG2	B	183	0	−1
1GG2	B	184	1	−1
1GG2	B	185	0	−1
1GG2	B	186	1	1
1GG2	B	187	0	−1
1GG2	B	188	0	−1
1GG2	B	189	0	−1
1GG2	B	190	0	−1
1GG2	B	191	0	−1
1GG2	B	192	0	−1
1GG2	B	193	0	−1
1GG2	B	194	1	−1
1GG2	B	195	1	−1
1GG2	B	196	1	−1
1GG2	B	197	1	−1
1GG2	B	198	0	−1
1GG2	B	199	0	−1
1GG2	B	200	0	−1
1GG2	B	201	0	−1
1GG2	B	202	0	−1
1GG2	B	203	0	−1
1GG2	B	204	1	1
1GG2	B	205	1	−1
1GG2	B	206	0	−1
1GG2	B	207	0	−1
1GG2	B	208	0	−1
1GG2	B	209	0	−1
1GG2	B	210	0	−1
1GG2	B	211	0	−1
1GG2	B	212	0	−1
1GG2	B	213	0	−1
1GG2	B	214	1	−1
1GG2	B	215	1	1
1GG2	B	216	0	−1
1GG2	B	217	1	1
1GG2	B	218	1	1
1GG2	B	219	1	1
1GG2	B	220	0	−1
1GG2	B	221	1	1
1GG2	B	222	0	−1
1GG2	B	223	1	−1
1GG2	B	224	1	−1

1GG2	B	225	0	−1
1GG2	B	226	1	−1
1GG2	B	227	1	1
1GG2	B	228	1	1
1GG2	B	229	0	−1
1GG2	B	230	0	−1
1GG2	B	231	0	−1
1GG2	B	232	0	−1
1GG2	B	233	0	−1
1GG2	B	234	0	−1
1GG2	B	235	0	−1
1GG2	B	236	1	1
1GG2	B	237	1	1
1GG2	B	238	0	−1
1GG2	B	239	1	−1
1GG2	B	240	0	−1
1GG2	B	241	0	−1
1GG2	B	242	0	−1
1GG2	B	243	0	−1
1GG2	B	244	0	−1
1GG2	B	245	0	−1
1GG2	B	246	1	1
1GG2	B	247	0	−1
1GG2	B	248	0	−1
1GG2	B	249	0	−1
1GG2	B	250	0	−1
1GG2	B	251	0	−1
1GG2	B	252	0	−1
1GG2	B	253	0	−1
1GG2	B	254	0	−1
1GG2	B	255	0	−1
1GG2	B	256	1	1
1GG2	B	257	1	1
1GG2	B	258	0	−1
1GG2	B	259	0	−1
1GG2	B	260	1	−1
1GG2	B	261	1	1
1GG2	B	262	0	−1
1GG2	B	263	1	−1
1GG2	B	264	0	−1
1GG2	B	265	1	−1
1GG2	B	266	1	−1
1GG2	B	267	1	−1
1GG2	B	268	1	−1
1GG2	B	269	0	−1
1GG2	B	270	1	−1
1GG2	B	271	0	−1
1GG2	B	272	0	−1
1GG2	B	273	0	−1
1GG2	B	274	0	−1
1GG2	B	275	0	−1
1GG2	B	276	0	−1

1GG2	B	277	0	−1
1GG2	B	278	0	−1
1GG2	B	279	0	−1
1GG2	B	280	1	1
1GG2	B	281	1	1
1GG2	B	282	0	−1
1GG2	B	283	0	−1
1GG2	B	284	0	−1
1GG2	B	285	0	−1
1GG2	B	286	0	−1
1GG2	B	287	0	−1
1GG2	B	288	0	−1
1GG2	B	289	0	−1
1GG2	B	290	0	−1
1GG2	B	291	1	−1
1GG2	B	292	1	−1
1GG2	B	293	1	−1
1GG2	B	294	0	−1
1GG2	B	295	0	−1
1GG2	B	296	0	−1
1GG2	B	297	0	−1
1GG2	B	298	0	−1
1GG2	B	299	0	−1
1GG2	B	300	1	1
1GG2	B	301	0	−1
1GG2	B	302	0	−1
1GG2	B	303	1	−1
1GG2	B	304	1	−1
1GG2	B	305	0	−1
1GG2	B	306	0	−1
1GG2	B	307	1	−1
1GG2	B	308	0	−1
1GG2	B	309	1	−1
1GG2	B	310	1	−1
1GG2	B	311	0	−1
1GG2	B	312	1	−1
1GG2	B	313	1	1
1GG2	B	314	1	1
1GG2	B	315	0	−1
1GG2	B	316	0	−1
1GG2	B	317	0	−1
1GG2	B	318	0	−1
1GG2	B	319	0	−1
1GG2	B	320	0	−1
1GG2	B	321	0	−1
1GG2	B	322	1	−1
1GG2	B	323	1	1
1GG2	B	324	1	1
1GG2	B	325	1	1
1GG2	B	326	0	−1
1GG2	B	327	0	−1
1GG2	B	328	0	−1

1GG2	B	329	0	−1
1GG2	B	330	0	−1
1GG2	B	331	0	−1
1GG2	B	332	0	−1
1GG2	B	333	0	−1
1GG2	B	334	0	−1
1GG2	B	335	1	−1
1GG2	B	336	0	−1
1GG2	B	337	0	−1
1GG2	B	338	0	−1
1GG2	B	339	0	−1
1GG2	B	340	1	1
1GG2	G	8	1	1
1GG2	G	9	1	1
1GG2	G	10	1	−1
1GG2	G	11	1	1
1GG2	G	12	1	1
1GG2	G	13	1	1
1GG2	G	14	1	−1
1GG2	G	15	1	1
1GG2	G	16	1	1
1GG2	G	17	1	−1
1GG2	G	18	1	1
1GG2	G	19	1	1
1GG2	G	20	1	1
1GG2	G	21	1	1
1GG2	G	22	1	1
1GG2	G	23	1	1
1GG2	G	24	1	1
1GG2	G	25	1	1
1GG2	G	26	1	1
1GG2	G	27	1	1
1GG2	G	28	1	1
1GG2	G	29	1	1
1GG2	G	30	1	1
1GG2	G	31	1	1
1GG2	G	32	1	1
1GG2	G	33	1	1
1GG2	G	34	1	1
1GG2	G	35	1	1
1GG2	G	36	1	1
1GG2	G	37	1	1
1GG2	G	38	1	1
1GG2	G	39	1	−1
1GG2	G	40	1	1
1GG2	G	41	1	1
1GG2	G	42	1	1
1GG2	G	43	1	−1
1GG2	G	44	1	1
1GG2	G	45	1	1
1GG2	G	46	1	−1
1GG2	G	47	1	1

1GG2	G	48	1	1
1GG2	G	49	1	1
1GG2	G	50	1	1
1GG2	G	51	1	1
1GG2	G	52	0	−1
1GG2	G	53	1	1
1GG2	G	54	1	1
1GG2	G	55	1	−1
1GG2	G	56	1	−1
1GG2	G	57	1	−1
1GG2	G	58	1	1
1GG2	G	59	1	1
1GG2	G	60	1	1
1GG2	G	61	1	1
1GLA	F	1	1	−1
1GLA	F	2	1	−1
1GLA	F	3	1	−1
1GLA	F	4	1	−1
1GLA	F	5	1	−1
1GLA	F	6	1	−1
1GLA	F	7	1	−1
1GLA	F	8	1	−1
1GLA	F	9	1	−1
1GLA	F	10	1	−1
1GLA	F	11	1	−1
1GLA	F	19	1	−1
1GLA	F	20	0	−1
1GLA	F	21	1	−1
1GLA	F	22	0	−1
1GLA	F	23	0	−1
1GLA	F	24	0	−1
1GLA	F	25	0	−1
1GLA	F	26	0	−1
1GLA	F	27	0	−1
1GLA	F	28	0	−1
1GLA	F	29	1	−1
1GLA	F	30	0	−1
1GLA	F	31	1	−1
1GLA	F	32	1	−1
1GLA	F	33	0	−1
1GLA	F	34	1	−1
1GLA	F	35	1	−1
1GLA	F	36	0	−1
1GLA	F	37	1	−1
1GLA	F	38	1	1
1GLA	F	39	1	1
1GLA	F	40	1	1
1GLA	F	41	0	−1
1GLA	F	42	0	−1
1GLA	F	43	1	1
1GLA	F	44	1	−1
1GLA	F	45	1	1

1GLA	F	46	1	1
1GLA	F	47	0	−1
1GLA	F	48	0	−1
1GLA	F	49	0	−1
1GLA	F	50	0	−1
1GLA	F	51	0	−1
1GLA	F	52	0	−1
1GLA	F	53	1	−1
1GLA	F	54	0	−1
1GLA	F	55	1	−1
1GLA	F	56	1	−1
1GLA	F	57	1	−1
1GLA	F	58	1	−1
1GLA	F	59	0	−1
1GLA	F	60	0	−1
1GLA	F	61	0	−1
1GLA	F	62	0	−1
1GLA	F	63	0	−1
1GLA	F	64	1	−1
1GLA	F	65	0	−1
1GLA	F	66	1	−1
1GLA	F	67	0	−1
1GLA	F	68	1	−1
1GLA	F	69	1	1
1GLA	F	70	0	−1
1GLA	F	71	1	1
1GLA	F	72	1	1
1GLA	F	73	0	−1
1GLA	F	74	0	−1
1GLA	F	75	0	−1
1GLA	F	76	0	−1
1GLA	F	77	0	−1
1GLA	F	78	0	−1
1GLA	F	79	0	−1
1GLA	F	80	1	−1
1GLA	F	81	0	−1
1GLA	F	82	1	−1
1GLA	F	83	1	−1
1GLA	F	84	1	−1
1GLA	F	85	0	−1
1GLA	F	86	1	−1
1GLA	F	87	0	−1
1GLA	F	88	0	−1
1GLA	F	89	0	−1
1GLA	F	90	0	−1
1GLA	F	91	0	−1
1GLA	F	92	0	−1
1GLA	F	93	0	−1
1GLA	F	94	1	1
1GLA	F	95	0	−1
1GLA	F	96	1	1
1GLA	F	97	1	1

1GLA	F	98	0	−1
1GLA	F	99	1	1
1GLA	F	100	0	−1
1GLA	F	101	1	−1
1GLA	F	102	0	−1
1GLA	F	103	0	−1
1GLA	F	104	1	−1
1GLA	F	105	1	−1
1GLA	F	106	1	−1
1GLA	F	107	0	−1
1GLA	F	108	1	−1
1GLA	F	109	1	−1
1GLA	F	110	1	−1
1GLA	F	111	1	−1
1GLA	F	112	1	−1
1GLA	F	113	0	−1
1GLA	F	114	0	−1
1GLA	F	115	1	−1
1GLA	F	116	0	−1
1GLA	F	117	1	−1
1GLA	F	118	1	−1
1GLA	F	119	0	−1
1GLA	F	120	0	−1
1GLA	F	121	1	−1
1GLA	F	122	0	−1
1GLA	F	123	1	−1
1GLA	F	124	0	−1
1GLA	F	125	1	−1
1GLA	F	126	1	−1
1GLA	F	127	0	−1
1GLA	F	128	1	−1
1GLA	F	129	1	−1
1GLA	F	130	1	−1
1GLA	F	131	0	−1
1GLA	F	132	1	−1
1GLA	F	133	0	−1
1GLA	F	134	0	−1
1GLA	F	135	0	−1
1GLA	F	136	0	−1
1GLA	F	137	0	−1
1GLA	F	138	0	−1
1GLA	F	139	0	−1
1GLA	F	140	0	−1
1GLA	F	141	1	1
1GLA	F	142	0	−1
1GLA	F	143	1	−1
1GLA	F	144	1	−1
1GLA	F	145	1	−1
1GLA	F	146	0	−1
1GLA	F	147	1	−1
1GLA	F	148	1	−1
1GLA	F	149	0	−1

1GLA	F	150	1	−1
1GLA	F	151	1	−1
1GLA	F	152	1	−1
1GLA	F	153	1	−1
1GLA	F	154	1	−1
1GLA	F	155	1	−1
1GLA	F	156	0	−1
1GLA	F	157	1	−1
1GLA	F	158	0	−1
1GLA	F	159	0	−1
1GLA	F	160	1	−1
1GLA	F	161	1	−1
1GLA	F	162	1	−1
1GLA	F	163	0	−1
1GLA	F	164	0	−1
1GLA	F	165	1	−1
1GLA	F	166	0	−1
1GLA	F	167	1	−1
1GLA	F	168	1	−1
1GLA	G	4	1	−1
1GLA	G	5	1	−1
1GLA	G	6	0	−1
1GLA	G	7	0	−1
1GLA	G	8	0	−1
1GLA	G	9	0	−1
1GLA	G	10	0	−1
1GLA	G	11	0	−1
1GLA	G	12	0	−1
1GLA	G	13	0	−1
1GLA	G	14	0	−1
1GLA	G	15	0	−1
1GLA	G	16	0	−1
1GLA	G	17	0	−1
1GLA	G	18	0	−1
1GLA	G	19	0	−1
1GLA	G	20	0	−1
1GLA	G	21	0	−1
1GLA	G	22	1	−1
1GLA	G	23	1	−1
1GLA	G	24	1	−1
1GLA	G	25	0	−1
1GLA	G	26	1	−1
1GLA	G	27	1	−1
1GLA	G	28	1	−1
1GLA	G	29	0	−1
1GLA	G	30	1	−1
1GLA	G	31	0	−1
1GLA	G	32	1	−1
1GLA	G	33	1	−1
1GLA	G	34	1	−1
1GLA	G	35	0	−1
1GLA	G	36	1	−1

1GLA	G	37	0	−1
1GLA	G	38	0	−1
1GLA	G	39	1	−1
1GLA	G	40	1	−1
1GLA	G	41	1	−1
1GLA	G	42	1	−1
1GLA	G	43	0	−1
1GLA	G	44	0	−1
1GLA	G	45	0	−1
1GLA	G	46	0	−1
1GLA	G	47	0	−1
1GLA	G	48	1	−1
1GLA	G	49	0	−1
1GLA	G	50	1	−1
1GLA	G	51	1	−1
1GLA	G	52	0	−1
1GLA	G	53	1	−1
1GLA	G	54	1	−1
1GLA	G	55	0	−1
1GLA	G	56	0	−1
1GLA	G	57	0	−1
1GLA	G	58	1	−1
1GLA	G	59	0	−1
1GLA	G	60	0	−1
1GLA	G	61	1	−1
1GLA	G	62	1	−1
1GLA	G	63	0	−1
1GLA	G	64	0	−1
1GLA	G	65	1	−1
1GLA	G	66	1	−1
1GLA	G	67	1	−1
1GLA	G	68	1	−1
1GLA	G	69	0	−1
1GLA	G	70	1	−1
1GLA	G	71	1	−1
1GLA	G	72	1	−1
1GLA	G	73	1	−1
1GLA	G	74	0	−1
1GLA	G	75	1	−1
1GLA	G	76	0	−1
1GLA	G	77	0	−1
1GLA	G	78	0	−1
1GLA	G	79	0	−1
1GLA	G	80	0	−1
1GLA	G	81	0	−1
1GLA	G	82	0	−1
1GLA	G	83	0	−1
1GLA	G	84	0	−1
1GLA	G	85	0	−1
1GLA	G	86	0	−1
1GLA	G	87	0	−1
1GLA	G	88	0	−1

1GLA	G	89	0	−1
1GLA	G	90	1	−1
1GLA	G	91	0	−1
1GLA	G	92	1	−1
1GLA	G	93	1	−1
1GLA	G	94	0	−1
1GLA	G	95	1	−1
1GLA	G	96	0	−1
1GLA	G	97	0	−1
1GLA	G	98	1	−1
1GLA	G	99	1	−1
1GLA	G	100	0	−1
1GLA	G	101	0	−1
1GLA	G	102	0	−1
1GLA	G	103	0	−1
1GLA	G	104	0	−1
1GLA	G	105	0	−1
1GLA	G	106	0	−1
1GLA	G	107	0	−1
1GLA	G	108	0	−1
1GLA	G	109	0	−1
1GLA	G	110	1	−1
1GLA	G	111	0	−1
1GLA	G	112	0	−1
1GLA	G	113	1	−1
1GLA	G	114	1	−1
1GLA	G	115	0	−1
1GLA	G	116	1	−1
1GLA	G	117	1	−1
1GLA	G	118	1	−1
1GLA	G	119	1	−1
1GLA	G	120	0	−1
1GLA	G	121	1	−1
1GLA	G	122	1	−1
1GLA	G	123	1	−1
1GLA	G	124	0	−1
1GLA	G	125	1	−1
1GLA	G	126	1	−1
1GLA	G	127	0	−1
1GLA	G	128	0	−1
1GLA	G	129	0	−1
1GLA	G	130	0	−1
1GLA	G	131	0	−1
1GLA	G	132	0	−1
1GLA	G	133	0	−1
1GLA	G	134	0	−1
1GLA	G	135	0	−1
1GLA	G	136	0	−1
1GLA	G	137	0	−1
1GLA	G	138	0	−1
1GLA	G	139	0	−1
1GLA	G	140	0	−1

1GLA	G	141	0	−1
1GLA	G	142	0	−1
1GLA	G	143	0	−1
1GLA	G	144	0	−1
1GLA	G	145	0	−1
1GLA	G	146	1	−1
1GLA	G	147	1	−1
1GLA	G	148	1	−1
1GLA	G	149	1	−1
1GLA	G	150	1	−1
1GLA	G	151	0	−1
1GLA	G	152	0	−1
1GLA	G	153	1	−1
1GLA	G	154	1	−1
1GLA	G	155	0	−1
1GLA	G	156	1	−1
1GLA	G	157	1	−1
1GLA	G	158	1	−1
1GLA	G	159	0	−1
1GLA	G	160	0	−1
1GLA	G	161	0	−1
1GLA	G	162	0	−1
1GLA	G	163	0	−1
1GLA	G	164	0	−1
1GLA	G	165	0	−1
1GLA	G	166	0	−1
1GLA	G	167	0	−1
1GLA	G	168	0	−1
1GLA	G	169	0	−1
1GLA	G	170	0	−1
1GLA	G	171	0	−1
1GLA	G	172	0	−1
1GLA	G	173	0	−1
1GLA	G	174	0	−1
1GLA	G	175	1	−1
1GLA	G	176	1	−1
1GLA	G	177	1	−1
1GLA	G	178	0	−1
1GLA	G	179	0	−1
1GLA	G	180	0	−1
1GLA	G	181	0	−1
1GLA	G	182	0	−1
1GLA	G	183	0	−1
1GLA	G	184	0	−1
1GLA	G	185	0	−1
1GLA	G	186	0	−1
1GLA	G	187	0	−1
1GLA	G	188	0	−1
1GLA	G	189	0	−1
1GLA	G	190	0	−1
1GLA	G	191	0	−1
1GLA	G	192	0	−1

1GLA	G	193	0	−1
1GLA	G	194	0	−1
1GLA	G	195	1	−1
1GLA	G	196	1	−1
1GLA	G	197	1	−1
1GLA	G	198	1	−1
1GLA	G	199	0	−1
1GLA	G	200	0	−1
1GLA	G	201	1	−1
1GLA	G	202	0	−1
1GLA	G	203	0	−1
1GLA	G	204	0	−1
1GLA	G	205	1	−1
1GLA	G	206	1	−1
1GLA	G	207	0	−1
1GLA	G	208	1	−1
1GLA	G	209	0	−1
1GLA	G	210	0	−1
1GLA	G	211	1	−1
1GLA	G	212	1	−1
1GLA	G	213	0	−1
1GLA	G	214	0	−1
1GLA	G	215	0	−1
1GLA	G	216	1	−1
1GLA	G	217	0	−1
1GLA	G	218	1	−1
1GLA	G	219	1	−1
1GLA	G	220	0	−1
1GLA	G	221	0	−1
1GLA	G	222	1	−1
1GLA	G	223	1	−1
1GLA	G	224	0	−1
1GLA	G	225	0	−1
1GLA	G	226	1	−1
1GLA	G	227	0	−1
1GLA	G	228	1	−1
1GLA	G	229	1	−1
1GLA	G	237	0	−1
1GLA	G	238	0	−1
1GLA	G	239	0	−1
1GLA	G	240	0	−1
1GLA	G	241	0	−1
1GLA	G	242	0	−1
1GLA	G	243	0	−1
1GLA	G	244	0	−1
1GLA	G	245	0	−1
1GLA	G	246	0	−1
1GLA	G	247	0	−1
1GLA	G	248	0	−1
1GLA	G	249	0	−1
1GLA	G	250	0	−1
1GLA	G	251	0	−1

1GLA	G	252	0	−1
1GLA	G	253	0	−1
1GLA	G	254	0	−1
1GLA	G	255	0	−1
1GLA	G	256	0	−1
1GLA	G	257	1	−1
1GLA	G	258	1	−1
1GLA	G	259	1	−1
1GLA	G	260	1	−1
1GLA	G	261	0	−1
1GLA	G	262	0	−1
1GLA	G	263	0	−1
1GLA	G	264	0	−1
1GLA	G	265	0	−1
1GLA	G	266	1	−1
1GLA	G	267	0	−1
1GLA	G	268	0	−1
1GLA	G	269	0	−1
1GLA	G	270	0	−1
1GLA	G	271	0	−1
1GLA	G	272	0	−1
1GLA	G	273	0	−1
1GLA	G	274	0	−1
1GLA	G	275	0	−1
1GLA	G	276	0	−1
1GLA	G	277	1	−1
1GLA	G	278	1	−1
1GLA	G	279	1	−1
1GLA	G	280	1	−1
1GLA	G	281	1	−1
1GLA	G	282	0	−1
1GLA	G	283	1	−1
1GLA	G	284	1	−1
1GLA	G	285	1	−1
1GLA	G	286	0	−1
1GLA	G	287	0	−1
1GLA	G	288	0	−1
1GLA	G	289	0	−1
1GLA	G	290	0	−1
1GLA	G	291	0	−1
1GLA	G	292	0	−1
1GLA	G	293	0	−1
1GLA	G	294	1	−1
1GLA	G	295	1	−1
1GLA	G	296	0	−1
1GLA	G	297	1	−1
1GLA	G	298	1	−1
1GLA	G	299	0	−1
1GLA	G	300	0	−1
1GLA	G	301	0	−1
1GLA	G	302	0	−1
1GLA	G	303	0	−1

1GLA	G	304	0	−1
1GLA	G	305	0	−1
1GLA	G	306	0	−1
1GLA	G	307	0	−1
1GLA	G	308	0	−1
1GLA	G	309	0	−1
1GLA	G	310	0	−1
1GLA	G	311	0	−1
1GLA	G	312	0	−1
1GLA	G	313	0	−1
1GLA	G	314	0	−1
1GLA	G	315	0	−1
1GLA	G	316	0	−1
1GLA	G	317	1	−1
1GLA	G	318	1	−1
1GLA	G	319	1	−1
1GLA	G	320	1	−1
1GLA	G	321	1	−1
1GLA	G	322	1	−1
1GLA	G	323	0	−1
1GLA	G	324	1	−1
1GLA	G	325	1	−1
1GLA	G	326	1	−1
1GLA	G	327	1	−1
1GLA	G	328	1	−1
1GLA	G	329	0	−1
1GLA	G	330	1	−1
1GLA	G	331	1	−1
1GLA	G	332	1	−1
1GLA	G	333	0	−1
1GLA	G	334	1	−1
1GLA	G	335	1	−1
1GLA	G	336	0	−1
1GLA	G	337	1	1
1GLA	G	338	1	1
1GLA	G	339	0	−1
1GLA	G	340	1	−1
1GLA	G	341	1	−1
1GLA	G	342	0	−1
1GLA	G	343	0	−1
1GLA	G	344	0	−1
1GLA	G	345	0	−1
1GLA	G	346	0	−1
1GLA	G	347	0	−1
1GLA	G	348	1	−1
1GLA	G	349	0	−1
1GLA	G	350	0	−1
1GLA	G	351	0	−1
1GLA	G	352	0	−1
1GLA	G	353	0	−1
1GLA	G	354	0	−1
1GLA	G	355	0	−1

1GLA	G	356	0	−1
1GLA	G	357	0	−1
1GLA	G	358	0	−1
1GLA	G	359	0	−1
1GLA	G	360	0	−1
1GLA	G	361	0	−1
1GLA	G	362	1	−1
1GLA	G	363	0	−1
1GLA	G	364	1	−1
1GLA	G	365	0	−1
1GLA	G	366	1	−1
1GLA	G	367	1	−1
1GLA	G	368	1	−1
1GLA	G	369	1	−1
1GLA	G	370	1	−1
1GLA	G	371	0	−1
1GLA	G	372	1	−1
1GLA	G	373	1	−1
1GLA	G	374	0	−1
1GLA	G	375	0	−1
1GLA	G	376	0	−1
1GLA	G	377	0	−1
1GLA	G	378	0	−1
1GLA	G	379	0	−1
1GLA	G	380	0	−1
1GLA	G	381	0	−1
1GLA	G	382	0	−1
1GLA	G	383	0	−1
1GLA	G	384	0	−1
1GLA	G	385	0	−1
1GLA	G	386	0	−1
1GLA	G	387	0	−1
1GLA	G	388	0	−1
1GLA	G	389	0	−1
1GLA	G	390	0	−1
1GLA	G	391	0	−1
1GLA	G	392	0	−1
1GLA	G	393	1	−1
1GLA	G	394	0	−1
1GLA	G	395	0	−1
1GLA	G	396	0	−1
1GLA	G	397	1	−1
1GLA	G	398	0	−1
1GLA	G	399	1	−1
1GLA	G	400	1	−1
1GLA	G	401	1	−1
1GLA	G	402	1	1
1GLA	G	403	0	−1
1GLA	G	404	1	1
1GLA	G	405	0	−1
1GLA	G	406	0	−1
1GLA	G	407	0	−1

1GLA	G	408	0	−1
1GLA	G	409	0	−1
1GLA	G	410	0	−1
1GLA	G	411	1	−1
1GLA	G	412	0	−1
1GLA	G	413	0	−1
1GLA	G	414	1	−1
1GLA	G	415	0	−1
1GLA	G	416	1	−1
1GLA	G	417	0	−1
1GLA	G	418	0	−1
1GLA	G	419	0	−1
1GLA	G	420	1	−1
1GLA	G	421	0	−1
1GLA	G	422	0	−1
1GLA	G	423	0	−1
1GLA	G	424	0	−1
1GLA	G	425	0	−1
1GLA	G	426	0	−1
1GLA	G	427	1	1
1GLA	G	428	0	−1
1GLA	G	429	1	1
1GLA	G	430	0	−1
1GLA	G	431	0	−1
1GLA	G	432	0	−1
1GLA	G	433	0	−1
1GLA	G	434	1	−1
1GLA	G	435	1	−1
1GLA	G	436	0	−1
1GLA	G	437	0	−1
1GLA	G	438	0	−1
1GLA	G	439	0	−1
1GLA	G	440	0	−1
1GLA	G	441	0	−1
1GLA	G	442	0	−1
1GLA	G	443	0	−1
1GLA	G	444	0	−1
1GLA	G	445	0	−1
1GLA	G	446	0	−1
1GLA	G	447	0	−1
1GLA	G	448	0	−1
1GLA	G	449	0	−1
1GLA	G	450	1	−1
1GLA	G	451	1	−1
1GLA	G	452	1	−1
1GLA	G	453	0	−1
1GLA	G	454	0	−1
1GLA	G	455	1	−1
1GLA	G	456	1	−1
1GLA	G	457	0	−1
1GLA	G	458	1	−1
1GLA	G	459	1	−1

1GLA	G	460	0	−1
1GLA	G	461	1	−1
1GLA	G	462	1	−1
1GLA	G	463	0	−1
1GLA	G	464	1	−1
1GLA	G	465	1	−1
1GLA	G	466	1	−1
1GLA	G	467	1	−1
1GLA	G	468	1	−1
1GLA	G	469	1	−1
1GLA	G	470	0	−1
1GLA	G	471	0	−1
1GLA	G	472	1	1
1GLA	G	473	1	1
1GLA	G	474	1	1
1GLA	G	475	1	1
1GLA	G	476	0	−1
1GLA	G	477	1	1
1GLA	G	478	1	1
1GLA	G	479	1	1
1GLA	G	480	1	1
1GLA	G	481	1	1
1GLA	G	482	0	−1
1GLA	G	483	0	−1
1GLA	G	484	1	−1
1GLA	G	485	0	−1
1GLA	G	486	0	−1
1GLA	G	487	1	−1
1GLA	G	488	1	−1
1GLA	G	489	0	−1
1GLA	G	490	0	−1
1GLA	G	491	1	−1
1GLA	G	492	1	−1
1GLA	G	493	0	−1
1GLA	G	494	1	−1
1GLA	G	495	1	−1
1GLA	G	496	1	−1
1GLA	G	497	1	−1
1GLA	G	498	1	−1
1GLA	G	499	1	−1
1GOT	A	6	1	−1
1GOT	A	7	1	−1
1GOT	A	8	1	1
1GOT	A	9	1	1
1GOT	A	10	1	−1
1GOT	A	11	1	1
1GOT	A	12	1	1
1GOT	A	13	1	1
1GOT	A	14	1	−1
1GOT	A	15	1	1
1GOT	A	16	1	1
1GOT	A	17	1	−1

1GOT	A	18	1	1
1GOT	A	19	1	1
1GOT	A	20	1	−1
1GOT	A	21	1	−1
1GOT	A	22	1	1
1GOT	A	23	1	1
1GOT	A	24	1	−1
1GOT	A	25	1	−1
1GOT	A	26	1	1
1GOT	A	27	1	−1
1GOT	A	28	1	−1
1GOT	A	29	0	−1
1GOT	A	30	0	−1
1GOT	A	31	0	−1
1GOT	A	32	0	−1
1GOT	A	33	0	−1
1GOT	A	34	0	−1
1GOT	A	35	0	−1
1GOT	A	36	0	−1
1GOT	A	37	0	−1
1GOT	A	38	1	−1
1GOT	A	39	1	−1
1GOT	A	40	0	−1
1GOT	A	41	0	−1
1GOT	A	42	0	−1
1GOT	A	43	1	−1
1GOT	A	44	0	−1
1GOT	A	45	0	−1
1GOT	A	46	0	−1
1GOT	A	47	0	−1
1GOT	A	48	0	−1
1GOT	A	50	0	−1
1GOT	A	51	0	−1
1GOT	A	52	0	−1
1GOT	A	53	0	−1
1GOT	A	54	1	−1
1GOT	A	55	1	−1
1GOT	A	56	0	−1
1GOT	A	57	0	−1
1GOT	A	58	1	−1
1GOT	A	59	1	−1
1GOT	A	60	1	−1
1GOT	A	61	1	−1
1GOT	A	62	0	−1
1GOT	A	63	1	−1
1GOT	A	64	1	−1
1GOT	A	65	0	−1
1GOT	A	66	0	−1
1GOT	A	67	1	−1
1GOT	A	68	0	−1
1GOT	A	69	0	−1
1GOT	A	70	0	−1

1GOT	A	71	1	−1
1GOT	A	72	0	−1
1GOT	A	73	0	−1
1GOT	A	74	0	−1
1GOT	A	75	1	−1
1GOT	A	76	0	−1
1GOT	A	77	0	−1
1GOT	A	78	0	−1
1GOT	A	79	0	−1
1GOT	A	80	0	−1
1GOT	A	81	0	−1
1GOT	A	82	1	−1
1GOT	A	83	0	−1
1GOT	A	85	1	−1
1GOT	A	86	1	−1
1GOT	A	87	0	−1
1GOT	A	88	1	−1
1GOT	A	89	0	−1
1GOT	A	90	1	−1
1GOT	A	91	0	−1
1GOT	A	92	1	−1
1GOT	A	93	1	−1
1GOT	A	94	1	−1
1GOT	A	95	1	−1
1GOT	A	96	0	−1
1GOT	A	97	1	−1
1GOT	A	98	1	−1
1GOT	A	99	0	−1
1GOT	A	100	0	−1
1GOT	A	101	1	−1
1GOT	A	102	1	−1
1GOT	A	103	0	−1
1GOT	A	105	1	−1
1GOT	A	107	0	−1
1GOT	A	108	1	−1
1GOT	A	109	1	−1
1GOT	A	110	0	−1
1GOT	A	111	1	−1
1GOT	A	112	1	−1
1GOT	A	113	0	−1
1GOT	A	114	1	−1
1GOT	A	116	1	−1
1GOT	A	117	1	−1
1GOT	A	118	1	−1
1GOT	A	120	0	−1
1GOT	A	121	1	−1
1GOT	A	122	0	−1
1GOT	A	123	0	−1
1GOT	A	124	0	−1
1GOT	A	125	1	−1
1GOT	A	126	0	−1
1GOT	A	127	0	−1

1GOT	A	128	1	−1
1GOT	A	129	0	−1
1GOT	A	130	1	−1
1GOT	A	131	0	−1
1GOT	A	132	0	−1
1GOT	A	133	1	−1
1GOT	A	134	1	−1
1GOT	A	135	0	−1
1GOT	A	136	1	−1
1GOT	A	137	1	−1
1GOT	A	138	1	−1
1GOT	A	139	1	−1
1GOT	A	140	0	−1
1GOT	A	141	1	−1
1GOT	A	142	0	−1
1GOT	A	143	1	−1
1GOT	A	144	0	−1
1GOT	A	145	0	−1
1GOT	A	146	1	−1
1GOT	A	147	0	−1
1GOT	A	148	0	−1
1GOT	A	149	0	−1
1GOT	A	150	0	−1
1GOT	A	151	0	−1
1GOT	A	152	0	−1
1GOT	A	153	1	−1
1GOT	A	154	1	−1
1GOT	A	155	0	−1
1GOT	A	156	1	−1
1GOT	A	157	1	−1
1GOT	A	158	0	−1
1GOT	A	159	0	−1
1GOT	A	160	1	−1
1GOT	A	161	1	−1
1GOT	A	162	1	−1
1GOT	A	163	0	−1
1GOT	A	164	1	−1
1GOT	A	165	0	−1
1GOT	A	166	1	−1
1GOT	A	167	1	−1
1GOT	A	168	1	−1
1GOT	A	169	0	−1
1GOT	A	170	0	−1
1GOT	A	171	0	−1
1GOT	A	172	0	−1
1GOT	A	173	0	−1
1GOT	A	174	1	−1
1GOT	A	175	1	−1
1GOT	A	176	1	−1
1GOT	A	177	1	−1
1GOT	A	178	1	1
1GOT	A	179	1	1

1GOT	A	180	1	1
1GOT	A	181	1	−1
1GOT	A	182	1	1
1GOT	A	183	0	−1
1GOT	A	184	1	1
1GOT	A	185	0	−1
1GOT	A	186	1	−1
1GOT	A	187	0	−1
1GOT	A	188	1	−1
1GOT	A	189	1	−1
1GOT	A	190	0	−1
1GOT	A	191	0	−1
1GOT	A	192	0	−1
1GOT	A	193	1	1
1GOT	A	195	0	−1
1GOT	A	196	0	−1
1GOT	A	197	0	−1
1GOT	A	198	1	−1
1GOT	A	199	1	−1
1GOT	A	200	1	1
1GOT	A	201	1	1
1GOT	A	202	1	1
1GOT	A	203	1	1
1GOT	A	204	1	−1
1GOT	A	205	1	1
1GOT	A	206	1	1
1GOT	A	207	0	−1
1GOT	A	208	0	−1
1GOT	A	209	1	1
1GOT	A	210	1	1
1GOT	A	211	0	−1
1GOT	A	212	1	1
1GOT	A	213	1	−1
1GOT	A	214	0	−1
1GOT	A	215	0	−1
1GOT	A	216	0	−1
1GOT	A	217	0	−1
1GOT	A	218	0	−1
1GOT	A	219	0	−1
1GOT	A	220	0	−1
1GOT	A	221	0	−1
1GOT	A	222	0	−1
1GOT	A	223	0	−1
1GOT	A	224	0	−1
1GOT	A	225	0	−1
1GOT	A	226	0	−1
1GOT	A	227	0	−1
1GOT	A	228	0	−1
1GOT	A	229	1	−1
1GOT	A	230	0	−1
1GOT	A	231	1	−1
1GOT	A	232	1	−1

1GOT	A	233	1	−1
1GOT	A	234	1	−1
1GOT	A	235	1	−1
1GOT	A	237	0	−1
1GOT	A	238	0	−1
1GOT	A	240	1	−1
1GOT	A	241	1	−1
1GOT	A	242	0	−1
1GOT	A	244	1	−1
1GOT	A	245	0	−1
1GOT	A	246	0	−1
1GOT	A	247	1	−1
1GOT	A	248	1	−1
1GOT	A	249	0	−1
1GOT	A	250	0	−1
1GOT	A	251	1	−1
1GOT	A	252	0	−1
1GOT	A	253	1	1
1GOT	A	254	1	1
1GOT	A	255	0	−1
1GOT	A	256	1	−1
1GOT	A	257	1	−1
1GOT	A	258	0	−1
1GOT	A	259	0	−1
1GOT	A	260	0	−1
1GOT	A	261	0	−1
1GOT	A	262	0	−1
1GOT	A	263	0	−1
1GOT	A	264	0	−1
1GOT	A	265	0	−1
1GOT	A	266	0	−1
1GOT	A	267	0	−1
1GOT	A	268	1	−1
1GOT	A	269	1	−1
1GOT	A	270	0	−1
1GOT	A	271	1	−1
1GOT	A	272	1	−1
1GOT	A	273	0	−1
1GOT	A	274	0	−1
1GOT	A	275	1	−1
1GOT	A	276	1	−1
1GOT	A	277	0	−1
1GOT	A	278	1	−1
1GOT	A	279	0	−1
1GOT	A	280	1	−1
1GOT	A	281	1	−1
1GOT	A	282	0	−1
1GOT	A	283	0	−1
1GOT	A	284	1	−1
1GOT	A	285	1	−1
1GOT	A	286	0	−1
1GOT	A	287	1	−1

1GOT	A	288	1	−1
1GOT	A	289	1	−1
1GOT	A	290	1	−1
1GOT	A	291	1	−1
1GOT	A	292	0	−1
1GOT	A	293	1	−1
1GOT	A	294	1	−1
1GOT	A	295	0	−1
1GOT	A	296	0	−1
1GOT	A	297	1	−1
1GOT	A	298	1	−1
1GOT	A	299	0	−1
1GOT	A	300	0	−1
1GOT	A	301	1	−1
1GOT	A	302	0	−1
1GOT	A	303	0	−1
1GOT	A	304	1	−1
1GOT	A	305	1	−1
1GOT	A	306	0	−1
1GOT	A	307	0	−1
1GOT	A	309	0	−1
1GOT	A	310	1	−1
1GOT	A	311	1	−1
1GOT	A	312	1	−1
1GOT	A	313	1	−1
1GOT	A	314	1	−1
1GOT	A	315	0	−1
1GOT	A	316	1	−1
1GOT	A	317	0	−1
1GOT	A	318	0	−1
1GOT	A	320	0	−1
1GOT	A	321	0	−1
1GOT	A	322	0	−1
1GOT	A	323	0	−1
1GOT	A	324	1	−1
1GOT	A	325	0	−1
1GOT	A	326	1	−1
1GOT	A	327	0	−1
1GOT	A	328	0	−1
1GOT	A	329	1	−1
1GOT	A	330	1	−1
1GOT	A	331	0	−1
1GOT	A	332	0	−1
1GOT	A	333	1	−1
1GOT	A	334	1	−1
1GOT	A	335	0	−1
1GOT	A	336	0	−1
1GOT	A	337	1	−1
1GOT	A	338	1	−1
1GOT	A	339	0	−1
1GOT	A	340	1	−1
1GOT	A	341	1	−1

1GOT	A	342	1	−1
1GOT	A	343	1	−1
1GOT	B	2	1	−1
1GOT	B	3	1	1
1GOT	B	4	1	1
1GOT	B	5	1	−1
1GOT	B	6	1	−1
1GOT	B	7	1	1
1GOT	B	8	1	1
1GOT	B	9	1	−1
1GOT	B	10	1	1
1GOT	B	11	1	1
1GOT	B	12	1	−1
1GOT	B	13	1	−1
1GOT	B	14	1	1
1GOT	B	15	1	1
1GOT	B	16	1	−1
1GOT	B	17	1	1
1GOT	B	18	1	1
1GOT	B	19	1	−1
1GOT	B	20	1	−1
1GOT	B	21	1	1
1GOT	B	22	0	−1
1GOT	B	23	1	−1
1GOT	B	24	1	1
1GOT	B	25	1	1
1GOT	B	26	1	1
1GOT	B	27	1	1
1GOT	B	28	1	1
1GOT	B	29	1	1
1GOT	B	30	0	−1
1GOT	B	31	1	−1
1GOT	B	32	1	−1
1GOT	B	33	1	1
1GOT	B	34	0	−1
1GOT	B	35	1	−1
1GOT	B	36	1	1
1GOT	B	37	1	1
1GOT	B	38	1	−1
1GOT	B	39	1	−1
1GOT	B	40	1	1
1GOT	B	41	1	−1
1GOT	B	42	1	−1
1GOT	B	43	0	−1
1GOT	B	44	1	−1
1GOT	B	45	0	−1
1GOT	B	46	1	1
1GOT	B	47	1	−1
1GOT	B	48	1	1
1GOT	B	49	1	1
1GOT	B	50	1	−1
1GOT	B	51	0	−1

1GOT	B	52	1	−1
1GOT	B	53	1	1
1GOT	B	54	0	−1
1GOT	B	55	1	1
1GOT	B	56	1	−1
1GOT	B	57	1	1
1GOT	B	58	0	−1
1GOT	B	59	1	1
1GOT	B	60	0	−1
1GOT	B	61	0	−1
1GOT	B	62	1	−1
1GOT	B	63	0	−1
1GOT	B	64	0	−1
1GOT	B	65	1	−1
1GOT	B	66	1	−1
1GOT	B	67	0	−1
1GOT	B	68	1	−1
1GOT	B	69	1	−1
1GOT	B	70	0	−1
1GOT	B	71	0	−1
1GOT	B	72	0	−1
1GOT	B	73	0	−1
1GOT	B	74	0	−1
1GOT	B	75	0	−1
1GOT	B	76	0	−1
1GOT	B	77	0	−1
1GOT	B	78	1	1
1GOT	B	79	0	−1
1GOT	B	80	0	−1
1GOT	B	81	0	−1
1GOT	B	82	0	−1
1GOT	B	83	0	−1
1GOT	B	84	0	−1
1GOT	B	85	1	1
1GOT	B	86	1	−1
1GOT	B	87	1	1
1GOT	B	88	1	1
1GOT	B	89	1	1
1GOT	B	90	1	1
1GOT	B	91	0	−1
1GOT	B	92	1	1
1GOT	B	93	0	−1
1GOT	B	94	1	−1
1GOT	B	95	0	−1
1GOT	B	96	1	1
1GOT	B	97	0	−1
1GOT	B	98	1	1
1GOT	B	99	1	1
1GOT	B	100	0	−1
1GOT	B	101	1	1
1GOT	B	102	0	−1
1GOT	B	103	0	−1

1GOT	B	104	0	−1
1GOT	B	105	0	−1
1GOT	B	106	0	−1
1GOT	B	107	1	−1
1GOT	B	108	1	−1
1GOT	B	109	1	−1
1GOT	B	110	1	−1
1GOT	B	111	0	−1
1GOT	B	112	0	−1
1GOT	B	113	0	−1
1GOT	B	114	0	−1
1GOT	B	115	0	−1
1GOT	B	116	0	−1
1GOT	B	117	1	1
1GOT	B	118	1	1
1GOT	B	119	1	1
1GOT	B	120	0	−1
1GOT	B	121	0	−1
1GOT	B	122	0	−1
1GOT	B	123	0	−1
1GOT	B	124	0	−1
1GOT	B	125	0	−1
1GOT	B	126	0	−1
1GOT	B	127	1	−1
1GOT	B	128	1	−1
1GOT	B	129	1	−1
1GOT	B	130	1	1
1GOT	B	131	1	1
1GOT	B	132	1	1
1GOT	B	133	0	−1
1GOT	B	134	1	−1
1GOT	B	135	1	−1
1GOT	B	136	1	−1
1GOT	B	137	1	−1
1GOT	B	138	1	−1
1GOT	B	139	0	−1
1GOT	B	140	1	−1
1GOT	B	141	1	−1
1GOT	B	142	0	−1
1GOT	B	143	1	1
1GOT	B	144	0	−1
1GOT	B	145	0	−1
1GOT	B	146	0	−1
1GOT	B	147	0	−1
1GOT	B	148	0	−1
1GOT	B	149	0	−1
1GOT	B	150	0	−1
1GOT	B	151	0	−1
1GOT	B	152	0	−1
1GOT	B	153	1	−1
1GOT	B	154	1	−1
1GOT	B	155	1	−1

1GOT	B	156	1	−1
1GOT	B	157	0	−1
1GOT	B	158	0	−1
1GOT	B	159	0	−1
1GOT	B	160	0	−1
1GOT	B	161	0	−1
1GOT	B	162	0	−1
1GOT	B	163	0	−1
1GOT	B	164	1	−1
1GOT	B	165	0	−1
1GOT	B	166	0	−1
1GOT	B	167	0	−1
1GOT	B	168	0	−1
1GOT	B	169	0	−1
1GOT	B	170	0	−1
1GOT	B	171	0	−1
1GOT	B	172	1	−1
1GOT	B	173	1	−1
1GOT	B	174	0	−1
1GOT	B	175	1	−1
1GOT	B	176	1	−1
1GOT	B	177	1	−1
1GOT	B	178	0	−1
1GOT	B	179	1	−1
1GOT	B	180	0	−1
1GOT	B	181	1	1
1GOT	B	182	1	1
1GOT	B	183	0	−1
1GOT	B	184	1	1
1GOT	B	185	0	−1
1GOT	B	186	1	1
1GOT	B	187	0	−1
1GOT	B	188	0	−1
1GOT	B	189	0	−1
1GOT	B	190	0	−1
1GOT	B	191	0	−1
1GOT	B	192	0	−1
1GOT	B	193	0	−1
1GOT	B	194	1	−1
1GOT	B	195	1	−1
1GOT	B	196	1	−1
1GOT	B	197	1	−1
1GOT	B	198	0	−1
1GOT	B	199	0	−1
1GOT	B	200	0	−1
1GOT	B	201	0	−1
1GOT	B	202	0	−1
1GOT	B	203	0	−1
1GOT	B	204	1	1
1GOT	B	205	0	−1
1GOT	B	206	0	−1
1GOT	B	207	0	−1

1GOT	B	208	0	−1
1GOT	B	209	0	−1
1GOT	B	210	0	−1
1GOT	B	211	0	−1
1GOT	B	212	0	−1
1GOT	B	213	0	−1
1GOT	B	214	1	−1
1GOT	B	215	1	−1
1GOT	B	216	0	−1
1GOT	B	217	1	1
1GOT	B	218	1	1
1GOT	B	219	1	1
1GOT	B	220	0	−1
1GOT	B	221	1	1
1GOT	B	222	0	−1
1GOT	B	223	1	−1
1GOT	B	224	1	−1
1GOT	B	225	0	−1
1GOT	B	226	1	−1
1GOT	B	227	1	−1
1GOT	B	228	1	1
1GOT	B	229	0	−1
1GOT	B	230	0	−1
1GOT	B	231	0	−1
1GOT	B	232	0	−1
1GOT	B	233	0	−1
1GOT	B	234	0	−1
1GOT	B	235	0	−1
1GOT	B	236	1	1
1GOT	B	237	1	1
1GOT	B	238	0	−1
1GOT	B	239	1	1
1GOT	B	240	0	−1
1GOT	B	241	0	−1
1GOT	B	242	0	−1
1GOT	B	243	0	−1
1GOT	B	244	0	−1
1GOT	B	245	0	−1
1GOT	B	246	1	1
1GOT	B	247	0	−1
1GOT	B	248	0	−1
1GOT	B	249	0	−1
1GOT	B	250	0	−1
1GOT	B	251	0	−1
1GOT	B	252	0	−1
1GOT	B	253	0	−1
1GOT	B	254	0	−1
1GOT	B	255	0	−1
1GOT	B	256	1	1
1GOT	B	257	1	1
1GOT	B	258	1	1
1GOT	B	259	0	−1

1GOT	B	260	1	−1
1GOT	B	261	1	1
1GOT	B	262	0	−1
1GOT	B	263	1	−1
1GOT	B	264	0	−1
1GOT	B	265	1	−1
1GOT	B	266	1	−1
1GOT	B	267	1	−1
1GOT	B	268	1	−1
1GOT	B	269	0	−1
1GOT	B	270	1	−1
1GOT	B	271	0	−1
1GOT	B	272	0	−1
1GOT	B	273	0	−1
1GOT	B	274	0	−1
1GOT	B	275	0	−1
1GOT	B	276	0	−1
1GOT	B	277	0	−1
1GOT	B	278	0	−1
1GOT	B	279	0	−1
1GOT	B	280	1	1
1GOT	B	281	1	1
1GOT	B	282	0	−1
1GOT	B	283	0	−1
1GOT	B	284	0	−1
1GOT	B	285	0	−1
1GOT	B	286	0	−1
1GOT	B	287	0	−1
1GOT	B	288	0	−1
1GOT	B	289	0	−1
1GOT	B	290	0	−1
1GOT	B	291	1	−1
1GOT	B	292	1	−1
1GOT	B	293	0	−1
1GOT	B	294	0	−1
1GOT	B	295	0	−1
1GOT	B	296	0	−1
1GOT	B	297	0	−1
1GOT	B	298	0	−1
1GOT	B	299	0	−1
1GOT	B	300	1	1
1GOT	B	301	0	−1
1GOT	B	302	1	−1
1GOT	B	303	1	−1
1GOT	B	304	1	−1
1GOT	B	305	0	−1
1GOT	B	306	0	−1
1GOT	B	307	1	−1
1GOT	B	308	0	−1
1GOT	B	309	1	−1
1GOT	B	310	1	−1
1GOT	B	311	0	−1

1GOT	B	312	1	−1
1GOT	B	313	1	1
1GOT	B	314	1	1
1GOT	B	315	0	−1
1GOT	B	316	0	−1
1GOT	B	317	0	−1
1GOT	B	318	0	−1
1GOT	B	319	0	−1
1GOT	B	320	0	−1
1GOT	B	321	0	−1
1GOT	B	322	1	−1
1GOT	B	323	1	1
1GOT	B	324	1	1
1GOT	B	325	1	1
1GOT	B	326	0	−1
1GOT	B	327	0	−1
1GOT	B	328	0	−1
1GOT	B	329	0	−1
1GOT	B	330	0	−1
1GOT	B	331	0	−1
1GOT	B	332	1	1
1GOT	B	333	0	−1
1GOT	B	334	0	−1
1GOT	B	335	0	−1
1GOT	B	336	0	−1
1GOT	B	337	0	−1
1GOT	B	338	0	−1
1GOT	B	339	0	−1
1GOT	B	340	1	1
1GOT	G	16	1	1
1GOT	G	17	1	−1
1GOT	G	18	1	1
1GOT	G	19	1	1
1GOT	G	20	1	−1
1GOT	G	21	1	1
1GOT	G	22	1	1
1GOT	G	23	1	1
1GOT	G	24	1	1
1GOT	G	25	1	1
1GOT	G	26	1	1
1GOT	G	27	1	−1
1GOT	G	28	1	1
1GOT	G	29	1	1
1GOT	G	30	1	1
1GOT	G	31	1	1
1GOT	G	32	1	1
1GOT	G	33	1	1
1GOT	G	34	1	−1
1GOT	G	35	1	1
1GOT	G	36	1	1
1GOT	G	37	1	1
1GOT	G	38	1	1

1GOT	G	39	1	1
1GOT	G	40	1	1
1GOT	G	41	1	1
1GOT	G	42	1	−1
1GOT	G	43	1	1
1GOT	G	44	1	1
1GOT	G	45	1	1
1GOT	G	46	1	1
1GOT	G	47	1	1
1GOT	G	48	1	1
1GOT	G	49	1	−1
1GOT	G	50	1	1
1GOT	G	51	1	1
1GOT	G	52	1	1
1GOT	G	53	1	−1
1GOT	G	54	1	1
1GOT	G	55	0	−1
1GOT	G	56	1	−1
1GOT	G	57	1	1
1GOT	G	58	1	1
1GOT	G	59	1	1
1GOT	G	60	1	1
1GOT	G	61	1	1
1GOT	G	62	1	−1
1GOT	G	63	1	1
1GOT	G	64	1	1
1GOT	G	65	1	−1
1GOT	G	66	1	1
1GOT	G	67	1	−1
1GOT	G	68	1	1
1GOT	G	69	0	−1
1GOT	G	70	1	1
1GOT	G	71	1	1
1GOT	G	72	1	1
1GOT	G	73	1	1
1GUA	A	1	1	−1
1GUA	A	2	1	−1
1GUA	A	3	1	1
1GUA	A	4	0	−1
1GUA	A	5	1	−1
1GUA	A	6	0	−1
1GUA	A	7	0	−1
1GUA	A	8	0	−1
1GUA	A	9	0	−1
1GUA	A	10	0	−1
1GUA	A	11	0	−1
1GUA	A	12	1	−1
1GUA	A	13	1	−1
1GUA	A	14	0	−1
1GUA	A	15	0	−1
1GUA	A	16	0	−1
1GUA	A	17	0	−1

1GUA	A	18	0	−1
1GUA	A	19	0	−1
1GUA	A	20	0	−1
1GUA	A	21	0	−1
1GUA	A	22	0	−1
1GUA	A	23	0	−1
1GUA	A	24	0	−1
1GUA	A	25	1	1
1GUA	A	26	1	−1
1GUA	A	27	1	1
1GUA	A	28	1	−1
1GUA	A	29	1	1
1GUA	A	30	1	−1
1GUA	A	31	1	1
1GUA	A	32	1	−1
1GUA	A	33	1	1
1GUA	A	34	1	1
1GUA	A	35	0	−1
1GUA	A	36	1	1
1GUA	A	37	1	1
1GUA	A	38	1	1
1GUA	A	39	1	1
1GUA	A	40	1	1
1GUA	A	41	1	1
1GUA	A	42	1	1
1GUA	A	43	1	−1
1GUA	A	44	0	−1
1GUA	A	45	1	−1
1GUA	A	46	0	−1
1GUA	A	47	1	−1
1GUA	A	48	1	−1
1GUA	A	49	1	−1
1GUA	A	50	1	−1
1GUA	A	51	0	−1
1GUA	A	52	0	−1
1GUA	A	53	0	−1
1GUA	A	54	0	−1
1GUA	A	55	0	−1
1GUA	A	56	0	−1
1GUA	A	57	0	−1
1GUA	A	58	0	−1
1GUA	A	59	0	−1
1GUA	A	60	1	−1
1GUA	A	61	1	−1
1GUA	A	62	1	−1
1GUA	A	63	1	−1
1GUA	A	64	1	−1
1GUA	A	65	1	−1
1GUA	A	66	1	−1
1GUA	A	67	1	−1
1GUA	A	68	0	−1
1GUA	A	69	1	−1

1GUA	A	70	1	−1
1GUA	A	71	1	−1
1GUA	A	72	0	−1
1GUA	A	73	1	−1
1GUA	A	74	1	−1
1GUA	A	75	0	−1
1GUA	A	76	1	−1
1GUA	A	77	0	−1
1GUA	A	78	0	−1
1GUA	A	79	0	−1
1GUA	A	80	0	−1
1GUA	A	81	0	−1
1GUA	A	82	0	−1
1GUA	A	83	0	−1
1GUA	A	84	0	−1
1GUA	A	85	1	−1
1GUA	A	86	1	−1
1GUA	A	87	1	−1
1GUA	A	88	1	−1
1GUA	A	89	0	−1
1GUA	A	90	0	−1
1GUA	A	91	1	−1
1GUA	A	92	1	−1
1GUA	A	93	0	−1
1GUA	A	94	1	−1
1GUA	A	95	1	−1
1GUA	A	96	0	−1
1GUA	A	97	0	−1
1GUA	A	98	1	−1
1GUA	A	99	0	−1
1GUA	A	100	0	−1
1GUA	A	101	0	−1
1GUA	A	102	1	−1
1GUA	A	103	0	−1
1GUA	A	104	1	−1
1GUA	A	105	1	−1
1GUA	A	106	1	−1
1GUA	A	107	1	−1
1GUA	A	108	1	−1
1GUA	A	109	0	−1
1GUA	A	110	0	−1
1GUA	A	111	0	−1
1GUA	A	112	0	−1
1GUA	A	113	0	−1
1GUA	A	114	0	−1
1GUA	A	115	0	−1
1GUA	A	116	0	−1
1GUA	A	117	1	−1
1GUA	A	118	0	−1
1GUA	A	119	1	−1
1GUA	A	120	1	−1
1GUA	A	121	1	−1

1GUA	A	122	1	−1
1GUA	A	123	1	−1
1GUA	A	124	0	−1
1GUA	A	125	1	−1
1GUA	A	126	0	−1
1GUA	A	127	1	−1
1GUA	A	128	1	−1
1GUA	A	129	1	−1
1GUA	A	130	1	−1
1GUA	A	131	0	−1
1GUA	A	132	1	−1
1GUA	A	133	1	−1
1GUA	A	134	0	−1
1GUA	A	135	0	−1
1GUA	A	136	1	−1
1GUA	A	137	1	−1
1GUA	A	138	0	−1
1GUA	A	139	1	−1
1GUA	A	140	1	−1
1GUA	A	141	0	−1
1GUA	A	142	1	−1
1GUA	A	143	0	−1
1GUA	A	144	0	−1
1GUA	A	145	0	−1
1GUA	A	146	0	−1
1GUA	A	147	0	−1
1GUA	A	148	0	−1
1GUA	A	149	1	−1
1GUA	A	150	1	−1
1GUA	A	151	1	−1
1GUA	A	152	1	−1
1GUA	A	153	1	−1
1GUA	A	154	0	−1
1GUA	A	155	1	−1
1GUA	A	156	1	−1
1GUA	A	157	0	−1
1GUA	A	158	0	−1
1GUA	A	159	0	−1
1GUA	A	160	1	−1
1GUA	A	161	0	−1
1GUA	A	162	0	−1
1GUA	A	163	1	−1
1GUA	A	164	0	−1
1GUA	A	165	0	−1
1GUA	A	166	1	−1
1GUA	A	167	1	−1
1GUA	B	6	1	−1
1GUA	B	7	1	1
1GUA	B	8	0	−1
1GUA	B	9	1	1
1GUA	B	10	0	−1
1GUA	B	11	1	−1

1GUA	B	12	0	−1
1GUA	B	13	0	−1
1GUA	B	14	1	1
1GUA	B	15	1	1
1GUA	B	16	1	1
1GUA	B	17	1	1
1GUA	B	18	0	−1
1GUA	B	19	1	1
1GUA	B	20	0	−1
1GUA	B	21	1	1
1GUA	B	22	0	−1
1GUA	B	23	1	1
1GUA	B	24	1	−1
1GUA	B	25	1	−1
1GUA	B	26	0	−1
1GUA	B	27	0	−1
1GUA	B	28	0	−1
1GUA	B	29	1	−1
1GUA	B	30	1	−1
1GUA	B	31	0	−1
1GUA	B	32	0	−1
1GUA	B	33	1	−1
1GUA	B	34	1	1
1GUA	B	35	0	−1
1GUA	B	36	0	−1
1GUA	B	37	1	1
1GUA	B	38	1	1
1GUA	B	39	1	1
1GUA	B	40	1	1
1GUA	B	41	0	−1
1GUA	B	42	1	−1
1GUA	B	43	0	−1
1GUA	B	44	1	−1
1GUA	B	45	1	−1
1GUA	B	46	0	−1
1GUA	B	47	0	−1
1GUA	B	48	0	−1
1GUA	B	49	0	−1
1GUA	B	50	1	−1
1GUA	B	51	0	−1
1GUA	B	52	0	−1
1GUA	B	53	1	−1
1GUA	B	54	1	−1
1GUA	B	55	1	−1
1GUA	B	56	1	−1
1GUA	B	57	1	−1
1GUA	B	58	1	−1
1GUA	B	59	1	−1
1GUA	B	60	1	−1
1GUA	B	61	1	−1
1GUA	B	62	0	−1
1GUA	B	63	1	−1

1GUA	B	64	0	−1
1GUA	B	65	1	−1
1GUA	B	66	1	−1
1GUA	B	67	1	−1
1GUA	B	68	0	−1
1GUA	B	69	0	−1
1GUA	B	70	1	−1
1GUA	B	71	0	−1
1GUA	B	72	1	−1
1GUA	B	73	1	−1
1GUA	B	74	1	−1
1GUA	B	75	1	−1
1GUA	B	76	0	−1
1GUA	B	77	0	−1
1GUA	B	78	0	−1
1GUA	B	79	1	−1
1GUA	B	80	1	−1
1GUA	B	81	1	−1
1HIA	Y	1	0	−1
1HIA	Y	2	0	−1
1HIA	Y	3	1	−1
1HIA	Y	4	1	−1
1HIA	Y	5	1	−1
1HIA	Y	6	1	−1
1HIA	Y	7	0	−1
1HIA	Y	8	1	−1
1HIA	Y	9	1	−1
1HIA	Y	10	1	−1
1HIA	Y	11	1	−1
1HIA	Y	12	0	−1
1HIA	Y	13	0	−1
1HIA	Y	14	0	−1
1HIA	Y	15	0	−1
1HIA	Y	16	0	−1
1HIA	Y	17	0	−1
1HIA	Y	18	0	−1
1HIA	Y	19	0	−1
1HIA	Y	20	0	−1
1HIA	Y	21	1	−1
1HIA	Y	22	1	1
1HIA	Y	23	1	1
1HIA	Y	24	0	−1
1HIA	Y	25	1	1
1HIA	Y	26	0	−1
1HIA	Y	27	0	−1
1HIA	Y	28	0	−1
1HIA	Y	29	0	−1
1HIA	Y	30	0	−1
1HIA	Y	31	0	−1
1HIA	Y	32	1	−1
1HIA	Y	33	1	−1
1HIA	Y	34	0	−1

1HIA	Y	35	0	−1
1HIA	Y	36	0	−1
1HIA	Y	37	0	−1
1HIA	Y	38	0	−1
1HIA	Y	39	0	−1
1HIA	Y	40	0	−1
1HIA	Y	41	1	1
1HIA	Y	42	0	−1
1HIA	Y	43	1	−1
1HIA	Y	44	1	−1
1HIA	Y	45	1	−1
1HIA	Y	46	1	−1
1HIA	Y	47	0	−1
1HIA	Y	48	1	−1
1HIA	Y	49	0	−1
1HIA	Y	50	0	−1
1HIA	Y	51	0	−1
1HIA	Y	52	0	−1
1HIA	Y	53	0	−1
1HIA	Y	54	0	−1
1HIA	Y	55	0	−1
1HIA	Y	56	0	−1
1HIA	Y	57	1	1
1HIA	Y	58	1	−1
1HIA	Y	59	1	−1
1HIA	Y	60	0	−1
1HIA	Y	61	1	−1
1HIA	Y	62	1	−1
1HIA	Y	63	0	−1
1HIA	Y	64	0	−1
1HIA	Y	65	1	−1
1HIA	Y	66	0	−1
1HIA	Y	67	0	−1
1HIA	Y	68	0	−1
1HIA	Y	69	1	−1
1HIA	Y	70	0	−1
1HIA	Y	71	1	−1
1HIA	Y	72	0	−1
1HIA	Y	73	1	−1
1HIA	Y	74	0	−1
1HIA	Y	75	1	−1
1HIA	Y	76	0	−1
1HIA	Y	77	0	−1
1HIA	Y	78	1	−1
1HIA	Y	79	1	1
1HIA	Y	80	1	−1
1HIA	Y	3	1	1
1HIA	Y	4	1	−1
1HIA	Y	5	0	−1
1HIA	Y	6	0	−1
1HIA	Y	7	0	−1
1HIA	Y	8	0	−1

1HIA	Y	9	0	−1
1HIA	Y	10	0	−1
1HIA	Y	11	0	−1
1HIA	Y	12	0	−1
1HIA	Y	13	0	−1
1HIA	Y	14	0	−1
1HIA	Y	15	0	−1
1HIA	Y	16	1	−1
1HIA	Y	17	1	−1
1HIA	Y	18	1	−1
1HIA	Y	19	0	−1
1HIA	Y	20	1	−1
1HIA	Y	21	1	−1
1HIA	Y	22	1	−1
1HIA	Y	23	1	−1
1HIA	Y	24	1	−1
1HIA	Y	25	0	−1
1HIA	Y	26	1	−1
1HIA	Y	27	1	−1
1HIA	Y	28	0	−1
1HIA	Y	29	1	−1
1HIA	Y	30	1	−1
1HIA	Y	31	0	−1
1HIA	Y	32	1	−1
1HIA	Y	33	1	−1
1HIA	Y	34	1	−1
1HIA	Y	35	0	−1
1HIA	Y	36	1	−1
1HIA	Y	37	1	−1
1HIA	Y	38	1	−1
1HIA	Y	39	0	−1
1HIA	Y	40	1	−1
1HIA	Y	41	0	−1
1HIA	Y	42	1	−1
1HIA	Y	43	0	−1
1HIA	Y	44	0	−1
1HIA	Y	45	0	−1
1HIA	Y	46	0	−1
1HIA	Y	47	0	−1
1HIA	Y	48	0	−1
1HIA	Y	49	1	−1
1HIA	Y	50	1	−1
1HIA	Y	51	1	−1
1HIA	Y	52	0	−1
1HIA	Y	53	1	1
1HIA	Y	54	1	−1
1HIA	Y	55	1	−1
1HIA	Y	56	1	1
1HIA	Y	57	1	−1
1HIA	Y	58	1	1
1HIA	Y	59	0	−1
1HIA	Y	60	1	−1

1HIA	Y	61	1	−1
1HIA	Y	62	0	−1
1HIA	Y	63	0	−1
1HIA	Y	64	0	−1
1HIA	Y	65	0	−1
1HIA	Y	66	1	−1
1HIA	Y	67	0	−1
1HIA	Y	68	1	−1
1HIA	Y	69	0	−1
1HIA	Y	70	0	−1
1HIA	Y	71	1	−1
1HIA	Y	72	1	−1
1HIA	Y	73	1	−1
1HIA	Y	74	1	−1
1HIA	Y	75	0	−1
1HIA	Y	76	1	−1
1HIA	Y	77	1	−1
1HIA	Y	78	0	−1
1HIA	Y	79	0	−1
1HIA	Y	80	1	1
1HIA	Y	81	0	−1
1HIA	Y	82	0	−1
1HIA	Y	83	0	−1
1HIA	Y	84	0	−1
1HIA	Y	85	0	−1
1HIA	Y	86	0	−1
1HIA	Y	87	0	−1
1HIA	Y	88	0	−1
1HIA	Y	89	0	−1
1HIA	Y	90	0	−1
1HIA	Y	91	0	−1
1HIA	Y	92	1	−1
1HIA	Y	93	1	−1
1HIA	Y	94	1	−1
1HIA	Y	95	1	−1
1HIA	Y	96	1	−1
1HIA	Y	97	1	−1
1HIA	Y	98	0	−1
1HIA	Y	99	0	−1
1HIA	Y	100	0	−1
1HIA	Y	101	1	1
1HIA	Y	102	0	−1
1HIA	Y	103	0	−1
1HIA	Y	104	0	−1
1HIA	Y	105	0	−1
1HIA	Y	106	0	−1
1HIA	Y	107	0	−1
1HIA	Y	108	0	−1
1HIA	Y	109	0	−1
1HIA	Y	110	0	−1
1HIA	Y	111	1	−1
1HIA	Y	112	1	−1

1HIA	Y	113	1	−1
1HIA	Y	114	0	−1
1HIA	Y	115	0	−1
1HIA	Y	116	0	−1
1HIA	Y	117	0	−1
1HIA	Y	118	0	−1
1HIA	Y	119	0	−1
1HIA	Y	120	0	−1
1HIA	Y	121	1	1
1HIA	Y	122	1	1
1HIA	Y	123	1	1
1HIA	Y	124	1	1
1HIA	Y	125	0	−1
1HIA	Y	126	0	−1
1HIA	Y	127	0	−1
1HIA	Y	128	1	−1
1HIA	Y	129	1	−1
1HIA	Y	130	1	1
1HIA	Y	131	0	−1
1HIA	Y	132	0	−1
1HIA	Y	133	0	−1
1HIA	Y	134	0	−1
1HIA	Y	135	0	−1
1HIA	Y	136	0	−1
1HIA	Y	137	0	−1
1HIA	Y	138	0	−1
1HIA	Y	139	0	−1
1HIA	Y	140	0	−1
1HIA	Y	141	1	−1
1HIA	Y	142	1	−1
1HIA	Y	143	0	−1
1HIA	Y	144	0	−1
1HIA	Y	145	1	−1
1HIA	Y	146	0	−1
1HIA	Y	147	0	−1
1HIA	Y	148	0	−1
1HIA	Y	149	1	−1
1HIA	Y	150	1	−1
1HIA	Y	151	1	−1
1HIA	Y	152	1	−1
1HIA	Y	1	0	−1
1HIA	Y	2	0	−1
1HIA	Y	3	0	−1
1HIA	Y	4	0	−1
1HIA	Y	5	0	−1
1HIA	Y	6	0	−1
1HIA	Y	7	0	−1
1HIA	Y	8	1	−1
1HIA	Y	9	1	−1
1HIA	Y	10	1	−1
1HIA	Y	11	1	−1
1HIA	Y	12	0	−1

1HIA	Y	13	0	−1
1HIA	Y	14	0	−1
1HIA	Y	15	0	−1
1HIA	Y	16	0	−1
1HIA	Y	17	0	−1
1HIA	Y	18	0	−1
1HIA	Y	19	0	−1
1HIA	Y	20	0	−1
1HIA	Y	21	1	−1
1HIA	Y	22	1	1
1HIA	Y	23	1	1
1HIA	Y	24	0	−1
1HIA	Y	25	1	1
1HIA	Y	26	0	−1
1HIA	Y	27	0	−1
1HIA	Y	28	0	−1
1HIA	Y	29	0	−1
1HIA	Y	30	0	−1
1HIA	Y	31	0	−1
1HIA	Y	32	1	−1
1HIA	Y	33	1	−1
1HIA	Y	34	0	−1
1HIA	Y	35	0	−1
1HIA	Y	36	0	−1
1HIA	Y	37	0	−1
1HIA	Y	38	0	−1
1HIA	Y	39	0	−1
1HIA	Y	40	0	−1
1HIA	Y	41	1	1
1HIA	Y	42	0	−1
1HIA	Y	43	1	−1
1HIA	Y	44	1	−1
1HIA	Y	45	1	−1
1HIA	Y	46	1	−1
1HIA	Y	47	0	−1
1HIA	Y	48	1	−1
1HIA	Y	49	0	−1
1HIA	Y	50	0	−1
1HIA	Y	51	0	−1
1HIA	Y	52	0	−1
1HIA	Y	53	0	−1
1HIA	Y	54	0	−1
1HIA	Y	55	0	−1
1HIA	Y	56	0	−1
1HIA	Y	57	1	1
1HIA	Y	58	1	−1
1HIA	Y	59	1	−1
1HIA	Y	60	0	−1
1HIA	Y	61	1	−1
1HIA	Y	62	1	−1
1HIA	Y	63	1	−1
1HIA	Y	64	0	−1

1HIA	Y	65	1	−1
1HIA	Y	66	0	−1
1HIA	Y	67	0	−1
1HIA	Y	68	0	−1
1HIA	Y	69	1	−1
1HIA	Y	70	0	−1
1HIA	Y	71	1	−1
1HIA	Y	72	0	−1
1HIA	Y	73	1	−1
1HIA	Y	74	0	−1
1HIA	Y	75	1	−1
1HIA	Y	76	1	−1
1HIA	Y	77	0	−1
1HIA	Y	78	1	−1
1HIA	Y	79	1	1
1HIA	Y	80	1	−1
1HIA	Y	3	1	−1
1HIA	Y	4	1	1
1HIA	Y	5	1	−1
1HIA	Y	6	1	1
1HIA	Y	7	0	−1
1HIA	Y	8	1	−1
1HIA	Y	9	0	−1
1HIA	Y	10	0	−1
1HIA	Y	11	0	−1
1HIA	Y	12	0	−1
1HIA	Y	13	0	−1
1HIA	Y	14	0	−1
1HIA	Y	15	0	−1
1HIA	Y	16	1	−1
1HIA	Y	17	1	−1
1HIA	Y	18	1	−1
1HIA	Y	19	0	−1
1HIA	Y	20	1	−1
1HIA	Y	21	1	−1
1HIA	Y	22	0	−1
1HIA	Y	23	1	−1
1HIA	Y	24	1	−1
1HIA	Y	25	0	−1
1HIA	Y	26	1	−1
1HIA	Y	27	1	−1
1HIA	Y	28	0	−1
1HIA	Y	29	1	−1
1HIA	Y	30	1	−1
1HIA	Y	31	0	−1
1HIA	Y	32	1	−1
1HIA	Y	33	1	−1
1HIA	Y	34	1	−1
1HIA	Y	35	0	−1
1HIA	Y	36	1	−1
1HIA	Y	37	1	−1
1HIA	Y	38	1	−1

1HIA	Y	39	0	−1
1HIA	Y	40	1	−1
1HIA	Y	41	0	−1
1HIA	Y	42	1	−1
1HIA	Y	43	0	−1
1HIA	Y	44	0	−1
1HIA	Y	45	0	−1
1HIA	Y	46	0	−1
1HIA	Y	47	0	−1
1HIA	Y	48	0	−1
1HIA	Y	49	0	−1
1HIA	Y	50	0	−1
1HIA	Y	51	0	−1
1HIA	Y	52	0	−1
1HIA	Y	53	1	1
1HIA	Y	54	1	1
1HIA	Y	55	1	−1
1HIA	Y	56	1	1
1HIA	Y	57	0	−1
1HIA	Y	58	1	1
1HIA	Y	59	0	−1
1HIA	Y	60	1	−1
1HIA	Y	61	0	−1
1HIA	Y	62	0	−1
1HIA	Y	63	0	−1
1HIA	Y	64	0	−1
1HIA	Y	65	0	−1
1HIA	Y	66	1	−1
1HIA	Y	67	0	−1
1HIA	Y	68	1	−1
1HIA	Y	69	0	−1
1HIA	Y	70	0	−1
1HIA	Y	71	1	−1
1HIA	Y	72	1	−1
1HIA	Y	73	1	−1
1HIA	Y	74	1	−1
1HIA	Y	75	0	−1
1HIA	Y	76	1	−1
1HIA	Y	77	1	−1
1HIA	Y	78	0	−1
1HIA	Y	79	0	−1
1HIA	Y	80	1	1
1HIA	Y	81	0	−1
1HIA	Y	82	1	−1
1HIA	Y	83	0	−1
1HIA	Y	84	0	−1
1HIA	Y	85	1	−1
1HIA	Y	86	0	−1
1HIA	Y	87	0	−1
1HIA	Y	88	0	−1
1HIA	Y	89	0	−1
1HIA	Y	90	0	−1

1HIA	Y	91	0	−1
1HIA	Y	92	1	−1
1HIA	Y	93	1	−1
1HIA	Y	94	1	−1
1HIA	Y	95	0	−1
1HIA	Y	96	0	−1
1HIA	Y	97	0	−1
1HIA	Y	98	0	−1
1HIA	Y	99	0	−1
1HIA	Y	100	0	−1
1HIA	Y	101	1	1
1HIA	Y	102	0	−1
1HIA	Y	103	0	−1
1HIA	Y	104	0	−1
1HIA	Y	105	0	−1
1HIA	Y	106	0	−1
1HIA	Y	107	0	−1
1HIA	Y	108	0	−1
1HIA	Y	109	0	−1
1HIA	Y	110	0	−1
1HIA	Y	111	1	−1
1HIA	Y	112	1	−1
1HIA	Y	113	0	−1
1HIA	Y	114	0	−1
1HIA	Y	115	0	−1
1HIA	Y	116	0	−1
1HIA	Y	117	0	−1
1HIA	Y	118	0	−1
1HIA	Y	119	0	−1
1HIA	Y	120	0	−1
1HIA	Y	121	1	1
1HIA	Y	122	1	1
1HIA	Y	123	1	1
1HIA	Y	124	0	−1
1HIA	Y	125	0	−1
1HIA	Y	126	0	−1
1HIA	Y	127	0	−1
1HIA	Y	128	0	−1
1HIA	Y	129	1	−1
1HIA	Y	130	1	1
1HIA	Y	131	0	−1
1HIA	Y	132	0	−1
1HIA	Y	133	0	−1
1HIA	Y	134	0	−1
1HIA	Y	135	0	−1
1HIA	Y	136	0	−1
1HIA	Y	137	0	−1
1HIA	Y	138	0	−1
1HIA	Y	139	1	−1
1HIA	Y	140	0	−1
1HIA	Y	141	1	−1
1HIA	Y	142	1	−1

1HIA	Y	143	0	−1
1HIA	Y	144	0	−1
1HIA	Y	145	1	−1
1HIA	Y	146	1	−1
1HIA	Y	147	0	−1
1HIA	Y	148	0	−1
1HIA	Y	149	1	−1
1HIA	Y	150	1	−1
1HIA	Y	151	1	−1
1HIA	Y	152	1	−1
1HIA	I	1	1	−1
1HIA	I	2	0	−1
1HIA	I	3	1	−1
1HIA	I	4	1	−1
1HIA	I	5	1	−1
1HIA	I	6	1	−1
1HIA	I	7	1	−1
1HIA	I	8	0	−1
1HIA	I	9	1	−1
1HIA	I	10	0	−1
1HIA	I	11	0	−1
1HIA	I	12	1	1
1HIA	I	13	1	−1
1HIA	I	14	1	1
1HIA	I	15	1	−1
1HIA	I	16	1	−1
1HIA	I	17	1	−1
1HIA	I	18	0	−1
1HIA	I	19	1	−1
1HIA	I	20	1	−1
1HIA	I	21	0	−1
1HIA	I	22	1	1
1HIA	I	23	1	1
1HIA	I	24	1	1
1HIA	I	25	1	1
1HIA	I	26	1	1
1HIA	I	27	1	1
1HIA	I	28	1	1
1HIA	I	29	0	−1
1HIA	I	30	1	1
1HIA	I	31	1	−1
1HIA	I	32	1	−1
1HIA	I	33	0	−1
1HIA	I	34	1	−1
1HIA	I	35	1	−1
1HIA	I	36	0	−1
1HIA	I	37	1	−1
1HIA	I	38	1	−1
1HIA	I	39	1	−1
1HIA	I	40	0	−1
1HIA	I	41	1	1
1HIA	I	42	0	−1

1HIA	I	43	1	1
1HIA	I	44	1	1
1HIA	I	45	1	1
1HIA	I	46	1	1
1HIA	I	47	1	−1
1HIA	I	48	1	−1
1HIA	J	1	1	−1
1HIA	J	2	0	−1
1HIA	J	3	1	−1
1HIA	J	4	1	−1
1HIA	J	5	1	−1
1HIA	J	6	1	−1
1HIA	J	7	0	−1
1HIA	J	8	0	−1
1HIA	J	9	1	−1
1HIA	J	10	0	−1
1HIA	J	11	0	−1
1HIA	J	12	1	1
1HIA	J	13	1	−1
1HIA	J	14	1	1
1HIA	J	15	1	−1
1HIA	J	16	1	−1
1HIA	J	17	1	−1
1HIA	J	18	0	−1
1HIA	J	19	1	−1
1HIA	J	20	1	−1
1HIA	J	21	1	1
1HIA	J	22	1	1
1HIA	J	23	1	1
1HIA	J	24	0	−1
1HIA	J	25	1	1
1HIA	J	26	1	1
1HIA	J	27	1	1
1HIA	J	28	1	1
1HIA	J	29	0	−1
1HIA	J	30	1	1
1HIA	J	31	1	−1
1HIA	J	32	1	−1
1HIA	J	33	0	−1
1HIA	J	34	1	−1
1HIA	J	35	1	1
1HIA	J	36	0	−1
1HIA	J	37	1	−1
1HIA	J	38	1	−1
1HIA	J	39	1	−1
1HIA	J	40	0	−1
1HIA	J	41	1	1
1HIA	J	42	1	−1
1HIA	J	43	1	1
1HIA	J	44	1	1
1HIA	J	45	1	1
1HIA	J	46	1	1

1HIA	J	47	1	−1
1HIA	J	48	1	−1
1HWG	A	1	1	1
1HWG	A	2	1	1
1HWG	A	3	1	1
1HWG	A	4	1	1
1HWG	A	5	1	−1
1HWG	A	6	0	−1
1HWG	A	7	1	−1
1HWG	A	8	1	1
1HWG	A	9	0	−1
1HWG	A	10	0	−1
1HWG	A	11	1	1
1HWG	A	12	1	1
1HWG	A	13	0	−1
1HWG	A	14	0	−1
1HWG	A	15	1	1
1HWG	A	16	1	1
1HWG	A	17	0	−1
1HWG	A	18	1	1
1HWG	A	19	0	−1
1HWG	A	20	0	−1
1HWG	A	21	0	−1
1HWG	A	22	1	1
1HWG	A	23	0	−1
1HWG	A	24	0	−1
1HWG	A	25	1	1
1HWG	A	26	1	1
1HWG	A	27	0	−1
1HWG	A	28	0	−1
1HWG	A	29	1	1
1HWG	A	30	1	−1
1HWG	A	31	0	−1
1HWG	A	32	0	−1
1HWG	A	33	1	−1
1HWG	A	34	1	−1
1HWG	A	35	1	−1
1HWG	A	36	0	−1
1HWG	A	37	1	−1
1HWG	A	38	1	−1
1HWG	A	39	1	−1
1HWG	A	40	1	−1
1HWG	A	41	1	1
1HWG	A	42	1	1
1HWG	A	43	1	−1
1HWG	A	44	0	−1
1HWG	A	45	1	1
1HWG	A	46	1	1
1HWG	A	47	1	1
1HWG	A	48	1	1
1HWG	A	49	1	1
1HWG	A	50	0	−1

1HWG	A	51	0	−1
1HWG	A	52	1	1
1HWG	A	53	0	−1
1HWG	A	54	0	−1
1HWG	A	55	0	−1
1HWG	A	56	1	1
1HWG	A	57	1	−1
1HWG	A	58	0	−1
1HWG	A	59	1	−1
1HWG	A	60	1	−1
1HWG	A	61	0	−1
1HWG	A	62	1	1
1HWG	A	63	1	1
1HWG	A	64	1	1
1HWG	A	65	1	1
1HWG	A	66	1	−1
1HWG	A	67	0	−1
1HWG	A	68	1	1
1HWG	A	69	1	−1
1HWG	A	70	1	−1
1HWG	A	71	1	−1
1HWG	A	72	0	−1
1HWG	A	73	0	−1
1HWG	A	74	1	−1
1HWG	A	75	0	−1
1HWG	A	76	0	−1
1HWG	A	77	0	−1
1HWG	A	78	0	−1
1HWG	A	79	0	−1
1HWG	A	80	0	−1
1HWG	A	81	0	−1
1HWG	A	82	0	−1
1HWG	A	83	0	−1
1HWG	A	84	0	−1
1HWG	A	85	0	−1
1HWG	A	86	0	−1
1HWG	A	87	0	−1
1HWG	A	88	1	−1
1HWG	A	89	0	−1
1HWG	A	90	0	−1
1HWG	A	91	1	−1
1HWG	A	92	1	−1
1HWG	A	93	0	−1
1HWG	A	94	0	−1
1HWG	A	95	1	−1
1HWG	A	96	0	−1
1HWG	A	97	0	−1
1HWG	A	98	1	−1
1HWG	A	99	1	−1
1HWG	A	100	0	−1
1HWG	A	101	1	−1
1HWG	A	102	0	−1

1HWG	A	103	1	1
1HWG	A	104	0	−1
1HWG	A	105	0	−1
1HWG	A	106	1	−1
1HWG	A	107	1	−1
1HWG	A	108	1	−1
1HWG	A	109	1	−1
1HWG	A	110	0	−1
1HWG	A	111	1	−1
1HWG	A	112	1	−1
1HWG	A	113	0	−1
1HWG	A	114	0	−1
1HWG	A	115	1	−1
1HWG	A	116	1	1
1HWG	A	117	0	−1
1HWG	A	118	0	−1
1HWG	A	119	1	1
1HWG	A	120	1	1
1HWG	A	121	0	−1
1HWG	A	122	1	−1
1HWG	A	123	1	1
1HWG	A	124	0	−1
1HWG	A	125	0	−1
1HWG	A	126	1	−1
1HWG	A	127	1	−1
1HWG	A	128	0	−1
1HWG	A	129	1	−1
1HWG	A	130	1	−1
1HWG	A	131	1	−1
1HWG	A	132	0	−1
1HWG	A	133	1	−1
1HWG	A	134	1	−1
1HWG	A	135	1	−1
1HWG	A	136	1	−1
1HWG	A	137	1	−1
1HWG	A	138	0	−1
1HWG	A	139	0	−1
1HWG	A	140	1	−1
1HWG	A	141	0	−1
1HWG	A	142	1	−1
1HWG	A	143	1	−1
1HWG	A	144	0	−1
1HWG	A	145	1	−1
1HWG	A	146	1	−1
1HWG	A	147	1	−1
1HWG	A	154	1	−1
1HWG	A	155	1	−1
1HWG	A	156	1	−1
1HWG	A	157	1	−1
1HWG	A	158	1	−1
1HWG	A	159	0	−1
1HWG	A	160	0	−1

1HWG	A	161	0	−1
1HWG	A	162	0	−1
1HWG	A	163	0	−1
1HWG	A	164	0	−1
1HWG	A	165	0	−1
1HWG	A	166	0	−1
1HWG	A	167	1	1
1HWG	A	168	0	−1
1HWG	A	169	0	−1
1HWG	A	170	0	−1
1HWG	A	171	1	1
1HWG	A	172	0	−1
1HWG	A	173	0	−1
1HWG	A	174	0	−1
1HWG	A	175	1	1
1HWG	A	176	0	−1
1HWG	A	177	0	−1
1HWG	A	178	1	1
1HWG	A	179	0	−1
1HWG	A	180	0	−1
1HWG	A	181	0	−1
1HWG	A	182	1	1
1HWG	A	183	1	−1
1HWG	A	184	0	−1
1HWG	A	185	0	−1
1HWG	A	186	1	−1
1HWG	A	187	1	−1
1HWG	A	188	1	−1
1HWG	A	189	1	1
1HWG	A	190	1	1
1HWG	B	32	1	−1
1HWG	B	33	0	−1
1HWG	B	34	0	−1
1HWG	B	35	0	−1
1HWG	B	36	1	−1
1HWG	B	37	1	−1
1HWG	B	38	0	−1
1HWG	B	39	0	−1
1HWG	B	40	0	−1
1HWG	B	41	0	−1
1HWG	B	42	0	−1
1HWG	B	43	0	−1
1HWG	B	44	1	1
1HWG	B	45	0	−1
1HWG	B	46	0	−1
1HWG	B	47	0	−1
1HWG	B	48	0	−1
1HWG	B	49	1	−1
1HWG	B	50	0	−1
1HWG	B	51	1	−1
1HWG	B	52	1	−1
1HWG	B	53	1	−1

1HWG	B	63	1	−1
1HWG	B	64	1	−1
1HWG	B	65	1	−1
1HWG	B	66	0	−1
1HWG	B	67	0	−1
1HWG	B	68	0	−1
1HWG	B	69	0	−1
1HWG	B	70	0	−1
1HWG	B	71	1	1
1HWG	B	72	1	−1
1HWG	B	73	1	−1
1HWG	B	74	1	−1
1HWG	B	75	1	−1
1HWG	B	76	0	−1
1HWG	B	77	1	1
1HWG	B	78	1	−1
1HWG	B	79	0	−1
1HWG	B	80	1	1
1HWG	B	81	1	−1
1HWG	B	82	1	−1
1HWG	B	83	0	−1
1HWG	B	84	1	−1
1HWG	B	85	0	−1
1HWG	B	86	1	−1
1HWG	B	87	1	−1
1HWG	B	88	1	−1
1HWG	B	89	0	−1
1HWG	B	90	1	−1
1HWG	B	91	1	−1
1HWG	B	92	1	−1
1HWG	B	93	0	−1
1HWG	B	94	0	−1
1HWG	B	95	1	−1
1HWG	B	96	0	−1
1HWG	B	97	1	−1
1HWG	B	98	1	1
1HWG	B	99	1	1
1HWG	B	100	1	−1
1HWG	B	101	0	−1
1HWG	B	102	1	1
1HWG	B	103	0	−1
1HWG	B	104	1	1
1HWG	B	105	1	1
1HWG	B	106	1	1
1HWG	B	107	0	−1
1HWG	B	108	0	−1
1HWG	B	109	0	−1
1HWG	B	110	1	−1
1HWG	B	111	0	−1
1HWG	B	112	0	−1
1HWG	B	113	1	−1
1HWG	B	114	1	−1

1HWG	B	115	1	−1
1HWG	B	116	1	−1
1HWG	B	117	1	−1
1HWG	B	118	1	−1
1HWG	B	119	1	−1
1HWG	B	120	1	1
1HWG	B	121	1	1
1HWG	B	122	1	1
1HWG	B	123	0	−1
1HWG	B	124	0	−1
1HWG	B	125	0	−1
1HWG	B	126	1	1
1HWG	B	127	1	1
1HWG	B	128	0	−1
1HWG	B	129	0	−1
1HWG	B	130	1	−1
1HWG	B	131	0	−1
1HWG	B	132	1	−1
1HWG	B	133	1	−1
1HWG	B	134	0	−1
1HWG	B	135	1	−1
1HWG	B	136	1	−1
1HWG	B	137	0	−1
1HWG	B	138	1	−1
1HWG	B	139	1	−1
1HWG	B	140	1	−1
1HWG	B	141	1	−1
1HWG	B	142	1	−1
1HWG	B	143	1	−1
1HWG	B	144	1	−1
1HWG	B	145	0	−1
1HWG	B	146	1	−1
1HWG	B	147	0	−1
1HWG	B	148	1	−1
1HWG	B	149	0	−1
1HWG	B	150	0	−1
1HWG	B	151	0	−1
1HWG	B	152	0	−1
1HWG	B	153	0	−1
1HWG	B	154	0	−1
1HWG	B	155	0	−1
1HWG	B	156	0	−1
1HWG	B	157	0	−1
1HWG	B	158	1	−1
1HWG	B	159	0	−1
1HWG	B	160	0	−1
1HWG	B	161	1	−1
1HWG	B	162	1	−1
1HWG	B	163	0	−1
1HWG	B	164	0	−1
1HWG	B	165	1	1
1HWG	B	166	1	1

1HWG	B	167	1	1
1HWG	B	168	1	1
1HWG	B	169	1	1
1HWG	B	170	0	−1
1HWG	B	171	1	1
1HWG	B	172	0	−1
1HWG	B	173	0	−1
1HWG	B	174	0	−1
1HWG	B	175	0	−1
1HWG	B	176	0	−1
1HWG	B	177	0	−1
1HWG	B	178	0	−1
1HWG	B	179	0	−1
1HWG	B	180	0	−1
1HWG	B	181	1	−1
1HWG	B	182	1	−1
1HWG	B	183	0	−1
1HWG	B	184	1	−1
1HWG	B	185	1	−1
1HWG	B	186	0	−1
1HWG	B	187	1	−1
1HWG	B	188	1	−1
1HWG	B	189	0	−1
1HWG	B	190	1	−1
1HWG	B	191	1	−1
1HWG	B	192	0	−1
1HWG	B	193	1	−1
1HWG	B	194	1	−1
1HWG	B	195	1	−1
1HWG	B	196	1	−1
1HWG	B	197	0	−1
1HWG	B	198	1	−1
1HWG	B	199	0	−1
1HWG	B	200	0	−1
1HWG	B	201	0	−1
1HWG	B	202	0	−1
1HWG	B	203	1	−1
1HWG	B	204	0	−1
1HWG	B	205	1	−1
1HWG	B	206	1	−1
1HWG	B	207	1	−1
1HWG	B	208	0	−1
1HWG	B	209	0	−1
1HWG	B	210	0	−1
1HWG	B	211	0	−1
1HWG	B	212	0	−1
1HWG	B	213	0	−1
1HWG	B	214	0	−1
1HWG	B	215	0	−1
1HWG	B	216	0	−1
1HWG	B	217	1	1
1HWG	B	218	1	1

1HWG	B	219	0	−1
1HWG	B	220	1	1
1HWG	B	221	0	−1
1HWG	B	222	0	−1
1HWG	B	223	0	−1
1HWG	B	224	1	−1
1HWG	B	225	0	−1
1HWG	B	226	0	−1
1HWG	B	227	1	−1
1HWG	B	228	1	−1
1HWG	B	229	0	−1
1HWG	B	230	1	−1
1HWG	B	231	0	−1
1HWG	B	232	1	−1
1HWG	B	233	0	−1
1HWG	B	234	1	−1
1HWG	C	32	1	−1
1HWG	C	33	0	−1
1HWG	C	34	0	−1
1HWG	C	35	0	−1
1HWG	C	36	1	−1
1HWG	C	37	1	−1
1HWG	C	38	0	−1
1HWG	C	39	0	−1
1HWG	C	40	0	−1
1HWG	C	41	0	−1
1HWG	C	42	0	−1
1HWG	C	43	0	−1
1HWG	C	44	1	1
1HWG	C	45	0	−1
1HWG	C	46	0	−1
1HWG	C	47	0	−1
1HWG	C	48	0	−1
1HWG	C	49	1	−1
1HWG	C	50	0	−1
1HWG	C	51	1	−1
1HWG	C	52	1	−1
1HWG	C	63	1	−1
1HWG	C	64	0	−1
1HWG	C	65	1	−1
1HWG	C	66	0	−1
1HWG	C	67	0	−1
1HWG	C	68	0	−1
1HWG	C	69	0	−1
1HWG	C	70	1	1
1HWG	C	71	1	1
1HWG	C	72	1	1
1HWG	C	78	1	−1
1HWG	C	79	1	−1
1HWG	C	80	1	−1
1HWG	C	81	1	−1
1HWG	C	82	1	−1

1HWG	C	83	0	−1
1HWG	C	84	1	−1
1HWG	C	85	0	−1
1HWG	C	86	1	−1
1HWG	C	87	1	−1
1HWG	C	88	1	−1
1HWG	C	89	1	−1
1HWG	C	90	1	−1
1HWG	C	91	1	−1
1HWG	C	92	1	−1
1HWG	C	93	0	−1
1HWG	C	94	0	−1
1HWG	C	95	1	−1
1HWG	C	96	0	−1
1HWG	C	97	1	−1
1HWG	C	98	1	−1
1HWG	C	99	1	−1
1HWG	C	100	1	−1
1HWG	C	101	0	−1
1HWG	C	102	0	−1
1HWG	C	103	0	−1
1HWG	C	104	1	1
1HWG	C	105	1	1
1HWG	C	106	1	1
1HWG	C	107	0	−1
1HWG	C	108	0	−1
1HWG	C	109	0	−1
1HWG	C	110	0	−1
1HWG	C	111	0	−1
1HWG	C	112	0	−1
1HWG	C	113	1	−1
1HWG	C	114	1	−1
1HWG	C	115	1	−1
1HWG	C	116	1	−1
1HWG	C	117	1	−1
1HWG	C	118	1	−1
1HWG	C	119	1	−1
1HWG	C	120	1	−1
1HWG	C	121	1	−1
1HWG	C	122	1	1
1HWG	C	123	0	−1
1HWG	C	124	0	−1
1HWG	C	125	0	−1
1HWG	C	126	1	1
1HWG	C	127	1	1
1HWG	C	128	0	−1
1HWG	C	129	0	−1
1HWG	C	130	0	−1
1HWG	C	131	0	−1
1HWG	C	132	1	−1
1HWG	C	133	1	−1
1HWG	C	134	0	−1

1HWG	C	135	1	−1
1HWG	C	136	1	−1
1HWG	C	137	0	−1
1HWG	C	138	1	−1
1HWG	C	139	1	−1
1HWG	C	140	1	−1
1HWG	C	141	1	−1
1HWG	C	142	1	−1
1HWG	C	143	0	−1
1HWG	C	144	0	−1
1HWG	C	145	0	−1
1HWG	C	146	1	−1
1HWG	C	147	1	−1
1HWG	C	148	1	−1
1HWG	C	149	1	−1
1HWG	C	150	0	−1
1HWG	C	151	0	−1
1HWG	C	152	0	−1
1HWG	C	153	0	−1
1HWG	C	154	1	−1
1HWG	C	155	0	−1
1HWG	C	156	0	−1
1HWG	C	157	0	−1
1HWG	C	158	1	−1
1HWG	C	159	0	−1
1HWG	C	160	0	−1
1HWG	C	161	1	−1
1HWG	C	162	0	−1
1HWG	C	163	0	−1
1HWG	C	164	1	1
1HWG	C	165	1	1
1HWG	C	166	0	−1
1HWG	C	167	1	1
1HWG	C	168	1	1
1HWG	C	169	1	1
1HWG	C	170	0	−1
1HWG	C	171	1	1
1HWG	C	172	0	−1
1HWG	C	173	0	−1
1HWG	C	174	0	−1
1HWG	C	175	0	−1
1HWG	C	176	0	−1
1HWG	C	177	0	−1
1HWG	C	178	1	−1
1HWG	C	179	0	−1
1HWG	C	180	0	−1
1HWG	C	181	1	−1
1HWG	C	182	1	−1
1HWG	C	183	1	−1
1HWG	C	184	1	−1
1HWG	C	185	1	−1
1HWG	C	186	1	−1

1HWG	C	187	0	−1
1HWG	C	188	1	−1
1HWG	C	189	0	−1
1HWG	C	190	1	−1
1HWG	C	191	1	−1
1HWG	C	192	0	−1
1HWG	C	193	1	−1
1HWG	C	194	1	−1
1HWG	C	195	1	−1
1HWG	C	196	1	−1
1HWG	C	197	0	−1
1HWG	C	198	0	−1
1HWG	C	199	0	−1
1HWG	C	200	0	−1
1HWG	C	201	1	−1
1HWG	C	202	0	−1
1HWG	C	203	1	−1
1HWG	C	204	0	−1
1HWG	C	205	1	−1
1HWG	C	206	1	−1
1HWG	C	207	1	−1
1HWG	C	208	0	−1
1HWG	C	209	0	−1
1HWG	C	210	0	−1
1HWG	C	211	0	−1
1HWG	C	212	0	−1
1HWG	C	213	0	−1
1HWG	C	214	0	−1
1HWG	C	215	0	−1
1HWG	C	216	0	−1
1HWG	C	217	1	−1
1HWG	C	218	1	1
1HWG	C	219	0	−1
1HWG	C	220	0	−1
1HWG	C	221	1	−1
1HWG	C	222	0	−1
1HWG	C	223	0	−1
1HWG	C	224	1	−1
1HWG	C	225	1	−1
1HWG	C	226	0	−1
1HWG	C	227	1	−1
1HWG	C	228	1	−1
1HWG	C	229	0	−1
1HWG	C	230	1	−1
1HWG	C	231	0	−1
1HWG	C	232	1	−1
1HWG	C	233	0	−1
1HWG	C	234	1	−1
1HWG	C	235	1	−1
1HWG	C	236	1	−1
1HWG	C	237	1	−1
1IAI	L	1	1	−1

1IAI	L	2	0	−1
1IAI	L	3	1	−1
1IAI	L	4	0	−1
1IAI	L	5	1	−1
1IAI	L	6	0	−1
1IAI	L	7	1	−1
1IAI	L	8	1	−1
1IAI	L	9	1	−1
1IAI	L	10	1	−1
1IAI	L	11	1	−1
1IAI	L	12	1	−1
1IAI	L	13	0	−1
1IAI	L	14	1	−1
1IAI	L	15	1	−1
1IAI	L	16	1	−1
1IAI	L	17	1	−1
1IAI	L	18	1	−1
1IAI	L	19	0	−1
1IAI	L	20	1	−1
1IAI	L	21	0	−1
1IAI	L	22	1	−1
1IAI	L	23	0	−1
1IAI	L	24	1	−1
1IAI	L	25	0	−1
1IAI	L	26	1	−1
1IAI	L	27	1	1
1IAI	L	28	1	1
1IAI	L	29	0	−1
1IAI	L	30	0	−1
1IAI	L	31	1	−1
1IAI	L	32	0	−1
1IAI	L	33	0	−1
1IAI	L	34	0	−1
1IAI	L	35	0	−1
1IAI	L	36	0	−1
1IAI	L	37	0	−1
1IAI	L	38	0	−1
1IAI	L	39	1	−1
1IAI	L	40	1	−1
1IAI	L	41	1	−1
1IAI	L	42	1	−1
1IAI	L	43	0	−1
1IAI	L	44	0	−1
1IAI	L	45	1	−1
1IAI	L	46	0	−1
1IAI	L	47	0	−1
1IAI	L	48	0	−1
1IAI	L	49	0	−1
1IAI	L	50	0	−1
1IAI	L	51	0	−1
1IAI	L	52	1	−1
1IAI	L	53	1	−1

1IAI	L	54	1	−1
1IAI	L	55	0	−1
1IAI	L	56	1	−1
1IAI	L	57	1	−1
1IAI	L	58	0	−1
1IAI	L	59	1	−1
1IAI	L	60	1	−1
1IAI	L	61	0	−1
1IAI	L	62	0	−1
1IAI	L	63	1	−1
1IAI	L	64	0	−1
1IAI	L	65	1	−1
1IAI	L	66	1	−1
1IAI	L	67	1	−1
1IAI	L	68	1	1
1IAI	L	69	1	−1
1IAI	L	70	1	−1
1IAI	L	71	0	−1
1IAI	L	72	0	−1
1IAI	L	73	0	−1
1IAI	L	74	0	−1
1IAI	L	75	0	−1
1IAI	L	76	1	−1
1IAI	L	77	1	−1
1IAI	L	78	0	−1
1IAI	L	79	1	−1
1IAI	L	80	0	−1
1IAI	L	81	1	−1
1IAI	L	82	0	−1
1IAI	L	83	0	−1
1IAI	L	84	0	−1
1IAI	L	85	0	−1
1IAI	L	86	0	−1
1IAI	L	87	0	−1
1IAI	L	88	0	−1
1IAI	L	89	0	−1
1IAI	L	90	0	−1
1IAI	L	91	0	−1
1IAI	L	92	1	1
1IAI	L	93	1	1
1IAI	L	94	1	1
1IAI	L	95	0	−1
1IAI	L	96	0	−1
1IAI	L	97	0	−1
1IAI	L	98	0	−1
1IAI	L	99	0	−1
1IAI	L	100	1	−1
1IAI	L	101	0	−1
1IAI	L	102	0	−1
1IAI	L	103	1	−1
1IAI	L	104	0	−1
1IAI	L	105	1	−1

1IAI	L	106	0	−1
1IAI	L	107	1	−1
1IAI	L	108	1	−1
1IAI	L	109	1	−1
1IAI	L	110	1	−1
1IAI	L	111	0	−1
1IAI	L	112	1	−1
1IAI	L	113	0	−1
1IAI	L	114	1	−1
1IAI	L	115	0	−1
1IAI	L	116	0	−1
1IAI	L	117	0	−1
1IAI	L	118	0	−1
1IAI	L	119	0	−1
1IAI	L	120	0	−1
1IAI	L	121	0	−1
1IAI	L	122	1	−1
1IAI	L	123	1	−1
1IAI	L	124	0	−1
1IAI	L	125	0	−1
1IAI	L	126	1	−1
1IAI	L	127	1	−1
1IAI	L	128	1	−1
1IAI	L	129	0	−1
1IAI	L	130	0	−1
1IAI	L	131	0	−1
1IAI	L	132	0	−1
1IAI	L	133	0	−1
1IAI	L	134	0	−1
1IAI	L	135	0	−1
1IAI	L	136	0	−1
1IAI	L	137	0	−1
1IAI	L	138	1	−1
1IAI	L	139	0	−1
1IAI	L	140	0	−1
1IAI	L	141	0	−1
1IAI	L	142	1	−1
1IAI	L	143	1	−1
1IAI	L	144	0	−1
1IAI	L	145	1	−1
1IAI	L	146	0	−1
1IAI	L	147	1	−1
1IAI	L	148	0	−1
1IAI	L	149	1	−1
1IAI	L	150	0	−1
1IAI	L	151	1	−1
1IAI	L	152	1	−1
1IAI	L	153	1	−1
1IAI	L	154	1	−1
1IAI	L	155	1	−1
1IAI	L	156	1	−1
1IAI	L	157	1	−1

1IAI	L	158	1	−1
1IAI	L	159	1	−1
1IAI	L	160	0	−1
1IAI	L	161	1	−1
1IAI	L	162	0	−1
1IAI	L	163	1	−1
1IAI	L	164	0	−1
1IAI	L	165	1	−1
1IAI	L	166	0	−1
1IAI	L	167	1	−1
1IAI	L	168	1	−1
1IAI	L	169	1	−1
1IAI	L	170	1	−1
1IAI	L	171	0	−1
1IAI	L	172	0	−1
1IAI	L	173	0	−1
1IAI	L	174	0	−1
1IAI	L	175	0	−1
1IAI	L	176	0	−1
1IAI	L	177	0	−1
1IAI	L	178	0	−1
1IAI	L	179	0	−1
1IAI	L	180	1	−1
1IAI	L	181	0	−1
1IAI	L	182	1	−1
1IAI	L	183	1	−1
1IAI	L	184	1	−1
1IAI	L	185	1	−1
1IAI	L	186	0	−1
1IAI	L	187	1	−1
1IAI	L	188	1	−1
1IAI	L	189	0	−1
1IAI	L	190	1	−1
1IAI	L	191	1	−1
1IAI	L	192	0	−1
1IAI	L	193	0	−1
1IAI	L	194	0	−1
1IAI	L	195	0	−1
1IAI	L	196	0	−1
1IAI	L	197	1	−1
1IAI	L	198	0	−1
1IAI	L	199	1	−1
1IAI	L	200	1	−1
1IAI	L	201	1	−1
1IAI	L	202	1	−1
1IAI	L	203	1	−1
1IAI	L	204	1	−1
1IAI	L	205	1	−1
1IAI	L	206	1	−1
1IAI	L	207	1	−1
1IAI	L	208	1	−1
1IAI	L	209	0	−1

1IAI	L	210	1	−1
1IAI	L	211	1	−1
1IAI	L	212	1	−1
1IAI	L	213	1	−1
1IAI	L	214	1	−1
1IAI	H	1	1	−1
1IAI	H	2	0	−1
1IAI	H	3	1	−1
1IAI	H	4	0	−1
1IAI	H	5	1	−1
1IAI	H	6	0	−1
1IAI	H	7	0	−1
1IAI	H	8	1	−1
1IAI	H	9	1	−1
1IAI	H	10	1	−1
1IAI	H	11	0	−1
1IAI	H	12	1	−1
1IAI	H	13	1	−1
1IAI	H	14	1	−1
1IAI	H	15	1	−1
1IAI	H	16	1	−1
1IAI	H	17	1	−1
1IAI	H	18	0	−1
1IAI	H	19	1	−1
1IAI	H	20	0	−1
1IAI	H	21	1	−1
1IAI	H	22	0	−1
1IAI	H	23	1	−1
1IAI	H	24	0	−1
1IAI	H	25	1	−1
1IAI	H	26	1	−1
1IAI	H	27	0	−1
1IAI	H	28	1	−1
1IAI	H	29	0	−1
1IAI	H	30	1	1
1IAI	H	31	1	1
1IAI	H	32	1	1
1IAI	H	33	0	−1
1IAI	H	34	0	−1
1IAI	H	35	0	−1
1IAI	H	36	0	−1
1IAI	H	37	0	−1
1IAI	H	38	0	−1
1IAI	H	39	0	−1
1IAI	H	40	0	−1
1IAI	H	41	1	−1
1IAI	H	42	1	−1
1IAI	H	43	1	−1
1IAI	H	44	1	−1
1IAI	H	45	0	−1
1IAI	H	46	1	−1
1IAI	H	47	0	−1

1IAI	H	48	0	−1
1IAI	H	49	0	−1
1IAI	H	50	1	1
1IAI	H	51	0	−1
1IAI	H	52	1	1
1IAI	H	53	0	−1
1IAI	H	54	1	1
1IAI	H	55	1	1
1IAI	H	56	1	−1
1IAI	H	57	1	1
1IAI	H	58	0	−1
1IAI	H	59	1	1
1IAI	H	60	0	−1
1IAI	H	61	0	−1
1IAI	H	62	1	−1
1IAI	H	63	1	−1
1IAI	H	64	0	−1
1IAI	H	65	1	−1
1IAI	H	66	1	−1
1IAI	H	67	1	−1
1IAI	H	68	0	−1
1IAI	H	69	1	−1
1IAI	H	70	0	−1
1IAI	H	71	1	−1
1IAI	H	72	1	−1
1IAI	H	73	1	−1
1IAI	H	74	1	−1
1IAI	H	75	1	−1
1IAI	H	76	1	−1
1IAI	H	77	0	−1
1IAI	H	78	0	−1
1IAI	H	79	0	−1
1IAI	H	80	0	−1
1IAI	H	81	0	−1
1IAI	H	82	1	−1
1IAI	H	83	0	−1
1IAI	H	84	1	−1
1IAI	H	85	1	−1
1IAI	H	86	0	−1
1IAI	H	87	1	−1
1IAI	H	88	1	−1
1IAI	H	89	1	−1
1IAI	H	90	0	−1
1IAI	H	91	0	−1
1IAI	H	92	0	−1
1IAI	H	93	1	−1
1IAI	H	94	0	−1
1IAI	H	95	0	−1
1IAI	H	96	0	−1
1IAI	H	97	0	−1
1IAI	H	98	0	−1
1IAI	H	99	0	−1

1IAI	H	100	0	−1
1IAI	H	101	1	1
1IAI	H	102	0	−1
1IAI	H	103	1	−1
1IAI	H	104	1	1
1IAI	H	105	1	1
1IAI	H	106	1	1
1IAI	H	107	0	−1
1IAI	H	108	0	−1
1IAI	H	109	0	−1
1IAI	H	110	1	−1
1IAI	H	111	0	−1
1IAI	H	112	0	−1
1IAI	H	113	1	−1
1IAI	H	114	0	−1
1IAI	H	115	0	−1
1IAI	H	116	0	−1
1IAI	H	117	0	−1
1IAI	H	118	0	−1
1IAI	H	119	0	−1
1IAI	H	120	0	−1
1IAI	H	121	1	−1
1IAI	H	122	0	−1
1IAI	H	123	1	−1
1IAI	H	124	1	−1
1IAI	H	125	1	−1
1IAI	H	126	1	−1
1IAI	H	127	0	−1
1IAI	H	128	1	−1
1IAI	H	129	0	−1
1IAI	H	130	0	−1
1IAI	H	131	0	−1
1IAI	H	132	0	−1
1IAI	H	133	0	−1
1IAI	H	134	0	−1
1IAI	H	135	1	−1
1IAI	H	136	1	−1
1IAI	H	137	1	−1
1IAI	H	138	1	−1
1IAI	H	139	1	−1
1IAI	H	140	1	−1
1IAI	H	141	1	−1
1IAI	H	142	1	−1
1IAI	H	143	1	−1
1IAI	H	144	0	−1
1IAI	H	145	0	−1
1IAI	H	146	0	−1
1IAI	H	147	0	−1
1IAI	H	148	0	−1
1IAI	H	149	0	−1
1IAI	H	150	0	−1
1IAI	H	151	0	−1

1IAI	H	152	0	−1
1IAI	H	153	0	−1
1IAI	H	154	0	−1
1IAI	H	155	0	−1
1IAI	H	156	0	−1
1IAI	H	157	1	−1
1IAI	H	158	0	−1
1IAI	H	159	1	−1
1IAI	H	160	0	−1
1IAI	H	161	1	−1
1IAI	H	162	0	−1
1IAI	H	163	1	−1
1IAI	H	164	1	−1
1IAI	H	165	1	−1
1IAI	H	166	0	−1
1IAI	H	167	1	−1
1IAI	H	168	1	−1
1IAI	H	169	1	−1
1IAI	H	170	1	−1
1IAI	H	171	1	−1
1IAI	H	172	0	−1
1IAI	H	173	1	−1
1IAI	H	174	0	−1
1IAI	H	175	1	−1
1IAI	H	176	1	−1
1IAI	H	177	1	−1
1IAI	H	178	1	−1
1IAI	H	179	1	−1
1IAI	H	180	1	−1
1IAI	H	181	1	−1
1IAI	H	182	1	−1
1IAI	H	183	0	−1
1IAI	H	184	0	−1
1IAI	H	185	0	−1
1IAI	H	186	0	−1
1IAI	H	187	0	−1
1IAI	H	188	0	−1
1IAI	H	189	0	−1
1IAI	H	190	0	−1
1IAI	H	191	0	−1
1IAI	H	192	1	−1
1IAI	H	193	1	−1
1IAI	H	194	1	−1
1IAI	H	195	1	−1
1IAI	H	196	0	−1
1IAI	H	197	1	−1
1IAI	H	198	1	−1
1IAI	H	199	1	−1
1IAI	H	200	1	−1
1IAI	H	201	0	−1
1IAI	H	202	0	−1
1IAI	H	203	0	−1

1IAI	H	204	0	−1
1IAI	H	205	0	−1
1IAI	H	206	1	−1
1IAI	H	207	0	−1
1IAI	H	208	1	−1
1IAI	H	209	1	−1
1IAI	H	210	1	−1
1IAI	H	211	0	−1
1IAI	H	212	1	−1
1IAI	H	213	1	−1
1IAI	H	214	1	−1
1IAI	H	215	0	−1
1IAI	H	216	1	−1
1IAI	H	217	0	−1
1IAI	H	218	1	−1
1IAI	H	219	1	−1
1IAI	M	1	1	−1
1IAI	M	2	0	−1
1IAI	M	3	1	−1
1IAI	M	4	0	−1
1IAI	M	5	1	−1
1IAI	M	6	0	−1
1IAI	M	7	1	−1
1IAI	M	8	0	−1
1IAI	M	9	1	−1
1IAI	M	10	1	−1
1IAI	M	11	1	−1
1IAI	M	12	1	−1
1IAI	M	13	0	−1
1IAI	M	14	1	−1
1IAI	M	15	1	−1
1IAI	M	16	1	−1
1IAI	M	17	1	−1
1IAI	M	18	1	−1
1IAI	M	19	0	−1
1IAI	M	20	1	−1
1IAI	M	21	0	−1
1IAI	M	22	1	−1
1IAI	M	23	0	−1
1IAI	M	24	1	−1
1IAI	M	25	0	−1
1IAI	M	26	1	−1
1IAI	M	27	1	1
1IAI	M	28	1	1
1IAI	M	29	0	−1
1IAI	M	30	1	1
1IAI	M	31	1	1
1IAI	M	32	0	−1
1IAI	M	33	0	−1
1IAI	M	34	0	−1
1IAI	M	35	0	−1
1IAI	M	36	0	−1

1IAI	M	37	0	−1
1IAI	M	38	0	−1
1IAI	M	39	0	−1
1IAI	M	40	0	−1
1IAI	M	41	1	−1
1IAI	M	42	1	−1
1IAI	M	43	1	−1
1IAI	M	44	0	−1
1IAI	M	45	0	−1
1IAI	M	46	1	−1
1IAI	M	47	0	−1
1IAI	M	48	0	−1
1IAI	M	49	0	−1
1IAI	M	50	1	1
1IAI	M	51	0	−1
1IAI	M	52	0	−1
1IAI	M	53	1	1
1IAI	M	54	1	1
1IAI	M	55	1	−1
1IAI	M	56	0	−1
1IAI	M	57	1	−1
1IAI	M	58	1	−1
1IAI	M	59	0	−1
1IAI	M	60	1	−1
1IAI	M	61	1	−1
1IAI	M	62	0	−1
1IAI	M	63	0	−1
1IAI	M	64	1	−1
1IAI	M	65	0	−1
1IAI	M	66	1	−1
1IAI	M	67	1	−1
1IAI	M	68	1	1
1IAI	M	69	1	1
1IAI	M	70	1	−1
1IAI	M	71	1	−1
1IAI	M	72	0	−1
1IAI	M	73	1	−1
1IAI	M	74	0	−1
1IAI	M	75	1	−1
1IAI	M	76	0	−1
1IAI	M	77	1	−1
1IAI	M	78	1	−1
1IAI	M	79	0	−1
1IAI	M	80	1	−1
1IAI	M	81	1	−1
1IAI	M	82	1	−1
1IAI	M	83	0	−1
1IAI	M	84	0	−1
1IAI	M	85	0	−1
1IAI	M	86	0	−1
1IAI	M	87	0	−1
1IAI	M	88	0	−1

1IAI	M	89	0	−1
1IAI	M	90	0	−1
1IAI	M	91	0	−1
1IAI	M	92	0	−1
1IAI	M	93	1	1
1IAI	M	94	1	1
1IAI	M	95	1	1
1IAI	M	96	1	−1
1IAI	M	97	0	−1
1IAI	M	98	0	−1
1IAI	M	99	0	−1
1IAI	M	100	0	−1
1IAI	M	101	1	−1
1IAI	M	102	0	−1
1IAI	M	103	0	−1
1IAI	M	104	1	−1
1IAI	M	105	0	−1
1IAI	M	106	1	−1
1IAI	M	107	0	−1
1IAI	M	108	1	−1
1IAI	M	109	1	−1
1IAI	M	110	1	−1
1IAI	M	111	1	−1
1IAI	M	112	0	−1
1IAI	M	113	1	−1
1IAI	M	114	0	−1
1IAI	M	115	0	−1
1IAI	M	116	0	−1
1IAI	M	117	0	−1
1IAI	M	118	0	−1
1IAI	M	119	0	−1
1IAI	M	120	1	−1
1IAI	M	121	0	−1
1IAI	M	122	0	−1
1IAI	M	123	1	−1
1IAI	M	124	1	−1
1IAI	M	125	0	−1
1IAI	M	126	0	−1
1IAI	M	127	1	−1
1IAI	M	128	1	−1
1IAI	M	129	0	−1
1IAI	M	130	1	−1
1IAI	M	131	0	−1
1IAI	M	132	0	−1
1IAI	M	133	0	−1
1IAI	M	134	0	−1
1IAI	M	135	0	−1
1IAI	M	136	0	−1
1IAI	M	137	0	−1
1IAI	M	138	0	−1
1IAI	M	139	1	−1
1IAI	M	140	0	−1

1IAI	M	141	0	−1
1IAI	M	142	0	−1
1IAI	M	143	1	−1
1IAI	M	144	1	−1
1IAI	M	145	0	−1
1IAI	M	146	1	−1
1IAI	M	147	0	−1
1IAI	M	148	1	−1
1IAI	M	149	0	−1
1IAI	M	150	1	−1
1IAI	M	151	0	−1
1IAI	M	152	1	−1
1IAI	M	153	1	−1
1IAI	M	154	1	−1
1IAI	M	155	1	−1
1IAI	M	156	1	−1
1IAI	M	157	1	−1
1IAI	M	158	1	−1
1IAI	M	159	1	−1
1IAI	M	160	1	−1
1IAI	M	161	0	−1
1IAI	M	162	0	−1
1IAI	M	163	0	−1
1IAI	M	164	1	−1
1IAI	M	165	0	−1
1IAI	M	166	1	−1
1IAI	M	167	0	−1
1IAI	M	168	1	−1
1IAI	M	169	1	−1
1IAI	M	170	1	−1
1IAI	M	171	1	−1
1IAI	M	172	0	−1
1IAI	M	173	0	−1
1IAI	M	174	0	−1
1IAI	M	175	0	−1
1IAI	M	176	0	−1
1IAI	M	177	0	−1
1IAI	M	178	0	−1
1IAI	M	179	0	−1
1IAI	M	180	0	−1
1IAI	M	181	1	−1
1IAI	M	182	0	−1
1IAI	M	183	1	−1
1IAI	M	184	1	−1
1IAI	M	185	1	−1
1IAI	M	186	1	−1
1IAI	M	187	0	−1
1IAI	M	188	0	−1
1IAI	M	189	1	−1
1IAI	M	190	1	−1
1IAI	M	191	1	−1
1IAI	M	192	0	−1

1IAI	M	193	0	−1
1IAI	M	194	0	−1
1IAI	M	195	0	−1
1IAI	M	196	0	−1
1IAI	M	197	0	−1
1IAI	M	198	1	−1
1IAI	M	199	0	−1
1IAI	M	200	1	−1
1IAI	M	201	1	−1
1IAI	M	202	1	−1
1IAI	M	203	1	−1
1IAI	M	204	1	−1
1IAI	M	205	1	−1
1IAI	M	206	1	−1
1IAI	M	207	1	−1
1IAI	M	208	0	−1
1IAI	M	209	1	−1
1IAI	M	210	0	−1
1IAI	M	211	1	−1
1IAI	M	212	1	−1
1IAI	M	213	1	−1
1IAI	M	214	1	−1
1IAI	M	215	1	−1
1IAI	I	1	1	1
1IAI	I	2	0	−1
1IAI	I	3	1	−1
1IAI	I	4	0	−1
1IAI	I	5	1	−1
1IAI	I	6	0	−1
1IAI	I	7	1	−1
1IAI	I	8	1	−1
1IAI	I	9	1	−1
1IAI	I	10	1	−1
1IAI	I	11	0	−1
1IAI	I	12	0	−1
1IAI	I	13	1	−1
1IAI	I	14	1	−1
1IAI	I	15	1	−1
1IAI	I	16	0	−1
1IAI	I	17	1	−1
1IAI	I	18	0	−1
1IAI	I	19	1	−1
1IAI	I	20	0	−1
1IAI	I	21	0	−1
1IAI	I	22	0	−1
1IAI	I	23	1	−1
1IAI	I	24	0	−1
1IAI	I	25	1	−1
1IAI	I	26	1	1
1IAI	I	27	0	−1
1IAI	I	28	1	1
1IAI	I	29	1	1

1IAI	I	30	1	1
1IAI	I	31	1	1
1IAI	I	32	0	−1
1IAI	I	33	0	−1
1IAI	I	34	0	−1
1IAI	I	35	0	−1
1IAI	I	36	0	−1
1IAI	I	37	0	−1
1IAI	I	38	0	−1
1IAI	I	39	0	−1
1IAI	I	40	0	−1
1IAI	I	41	1	−1
1IAI	I	42	1	−1
1IAI	I	43	1	−1
1IAI	I	44	1	−1
1IAI	I	45	0	−1
1IAI	I	46	0	−1
1IAI	I	47	0	−1
1IAI	I	48	0	−1
1IAI	I	49	0	−1
1IAI	I	50	0	−1
1IAI	I	51	0	−1
1IAI	I	52	0	−1
1IAI	I	53	0	−1
1IAI	I	54	1	1
1IAI	I	55	1	1
1IAI	I	56	1	−1
1IAI	I	57	1	−1
1IAI	I	58	1	−1
1IAI	I	59	0	−1
1IAI	I	60	1	−1
1IAI	I	61	1	−1
1IAI	I	62	1	−1
1IAI	I	63	0	−1
1IAI	I	64	1	−1
1IAI	I	65	0	−1
1IAI	I	66	0	−1
1IAI	I	67	1	−1
1IAI	I	68	1	−1
1IAI	I	69	0	−1
1IAI	I	70	0	−1
1IAI	I	71	1	−1
1IAI	I	72	0	−1
1IAI	I	73	1	−1
1IAI	I	74	0	−1
1IAI	I	75	1	−1
1IAI	I	76	1	−1
1IAI	I	77	1	−1
1IAI	I	78	1	−1
1IAI	I	79	0	−1
1IAI	I	80	1	−1
1IAI	I	81	0	−1

1IAI	I	82	0	−1
1IAI	I	83	0	−1
1IAI	I	84	1	−1
1IAI	I	85	0	−1
1IAI	I	86	1	−1
1IAI	I	87	1	−1
1IAI	I	88	0	−1
1IAI	I	89	1	−1
1IAI	I	90	1	−1
1IAI	I	91	1	−1
1IAI	I	92	0	−1
1IAI	I	93	1	−1
1IAI	I	94	0	−1
1IAI	I	95	1	−1
1IAI	I	96	0	−1
1IAI	I	97	0	−1
1IAI	I	98	0	−1
1IAI	I	99	0	−1
1IAI	I	100	0	−1
1IAI	I	101	0	−1
1IAI	I	102	0	−1
1IAI	I	103	1	1
1IAI	I	104	1	1
1IAI	I	105	1	1
1IAI	I	106	1	1
1IAI	I	107	0	−1
1IAI	I	108	0	−1
1IAI	I	109	0	−1
1IAI	I	110	0	−1
1IAI	I	111	0	−1
1IAI	I	112	0	−1
1IAI	I	113	1	−1
1IAI	I	114	0	−1
1IAI	I	115	0	−1
1IAI	I	116	0	−1
1IAI	I	117	0	−1
1IAI	I	118	0	−1
1IAI	I	119	0	−1
1IAI	I	120	0	−1
1IAI	I	121	1	−1
1IAI	I	122	0	−1
1IAI	I	123	1	−1
1IAI	I	124	1	−1
1IAI	I	125	1	−1
1IAI	I	126	1	−1
1IAI	I	127	0	−1
1IAI	I	128	1	−1
1IAI	I	129	0	−1
1IAI	I	130	0	−1
1IAI	I	131	0	−1
1IAI	I	132	0	−1
1IAI	I	133	0	−1

1IAI	I	134	0	−1
1IAI	I	135	1	−1
1IAI	I	136	1	−1
1IAI	I	137	0	−1
1IAI	I	138	0	−1
1IAI	I	139	1	−1
1IAI	I	140	1	−1
1IAI	I	141	1	−1
1IAI	I	142	1	−1
1IAI	I	143	1	−1
1IAI	I	144	0	−1
1IAI	I	145	0	−1
1IAI	I	146	0	−1
1IAI	I	147	0	−1
1IAI	I	148	0	−1
1IAI	I	149	0	−1
1IAI	I	150	0	−1
1IAI	I	151	0	−1
1IAI	I	152	0	−1
1IAI	I	153	0	−1
1IAI	I	154	0	−1
1IAI	I	155	0	−1
1IAI	I	156	1	−1
1IAI	I	157	1	−1
1IAI	I	158	1	−1
1IAI	I	159	1	−1
1IAI	I	160	0	−1
1IAI	I	161	1	−1
1IAI	I	162	0	−1
1IAI	I	163	1	−1
1IAI	I	164	1	−1
1IAI	I	165	1	−1
1IAI	I	166	1	−1
1IAI	I	167	1	−1
1IAI	I	168	1	−1
1IAI	I	169	1	−1
1IAI	I	170	1	−1
1IAI	I	171	1	−1
1IAI	I	172	0	−1
1IAI	I	173	1	−1
1IAI	I	174	0	−1
1IAI	I	175	1	−1
1IAI	I	176	0	−1
1IAI	I	177	1	−1
1IAI	I	178	1	−1
1IAI	I	179	1	−1
1IAI	I	180	1	−1
1IAI	I	181	1	−1
1IAI	I	182	1	−1
1IAI	I	183	0	−1
1IAI	I	184	0	−1
1IAI	I	185	0	−1

1IAI	I	186	0	−1
1IAI	I	187	0	−1
1IAI	I	188	0	−1
1IAI	I	189	0	−1
1IAI	I	190	0	−1
1IAI	I	191	0	−1
1IAI	I	192	1	−1
1IAI	I	193	1	−1
1IAI	I	194	1	−1
1IAI	I	195	1	−1
1IAI	I	196	1	−1
1IAI	I	197	0	−1
1IAI	I	198	0	−1
1IAI	I	199	1	−1
1IAI	I	200	1	−1
1IAI	I	201	0	−1
1IAI	I	202	1	−1
1IAI	I	203	0	−1
1IAI	I	204	0	−1
1IAI	I	205	0	−1
1IAI	I	206	1	−1
1IAI	I	207	0	−1
1IAI	I	208	1	−1
1IAI	I	209	1	−1
1IAI	I	210	1	−1
1IAI	I	211	0	−1
1IAI	I	212	1	−1
1IAI	I	213	1	−1
1IAI	I	214	1	−1
1IAI	I	215	0	−1
1IAI	I	216	1	−1
1IAI	I	217	0	−1
1IAI	I	218	1	−1
1IGC	A	4	1	−1
1IGC	A	5	1	−1
1IGC	A	6	1	−1
1IGC	A	7	1	−1
1IGC	A	8	0	−1
1IGC	A	9	1	−1
1IGC	A	10	0	−1
1IGC	A	11	1	−1
1IGC	A	12	0	−1
1IGC	A	13	1	−1
1IGC	A	14	0	−1
1IGC	A	15	1	1
1IGC	A	16	1	1
1IGC	A	17	1	1
1IGC	A	18	1	1
1IGC	A	19	1	1
1IGC	A	20	1	1
1IGC	A	21	1	1
1IGC	A	22	1	1

1IGC	A	23	1	−1
1IGC	A	24	1	1
1IGC	A	25	0	−1
1IGC	A	26	1	−1
1IGC	A	27	1	−1
1IGC	A	28	1	−1
1IGC	A	29	1	−1
1IGC	A	30	1	−1
1IGC	A	31	0	−1
1IGC	A	32	1	−1
1IGC	A	33	1	−1
1IGC	A	34	1	−1
1IGC	A	35	0	−1
1IGC	A	36	1	−1
1IGC	A	37	1	−1
1IGC	A	38	1	1
1IGC	A	39	0	−1
1IGC	A	40	1	1
1IGC	A	41	1	1
1IGC	A	42	1	1
1IGC	A	43	1	1
1IGC	A	44	0	−1
1IGC	A	45	1	−1
1IGC	A	46	1	−1
1IGC	A	47	1	−1
1IGC	A	48	0	−1
1IGC	A	49	1	−1
1IGC	A	50	1	−1
1IGC	A	51	1	−1
1IGC	A	52	1	−1
1IGC	A	53	1	−1
1IGC	A	54	1	−1
1IGC	A	55	1	−1
1IGC	A	56	0	−1
1IGC	A	57	0	−1
1IGC	A	58	0	−1
1IGC	A	59	0	−1
1IGC	A	60	1	−1
1IGC	A	61	1	−1
1IGC	L	1	1	−1
1IGC	L	2	0	−1
1IGC	L	3	1	−1
1IGC	L	4	0	−1
1IGC	L	5	1	−1
1IGC	L	6	0	−1
1IGC	L	7	1	−1
1IGC	L	8	1	−1
1IGC	L	9	1	−1
1IGC	L	10	1	−1
1IGC	L	11	0	−1
1IGC	L	12	1	−1
1IGC	L	13	0	−1

1IGC	L	14	1	−1
1IGC	L	15	1	−1
1IGC	L	16	1	−1
1IGC	L	17	1	−1
1IGC	L	18	1	−1
1IGC	L	19	0	−1
1IGC	L	20	1	−1
1IGC	L	21	0	−1
1IGC	L	22	0	−1
1IGC	L	23	0	−1
1IGC	L	24	1	−1
1IGC	L	25	0	−1
1IGC	L	26	1	−1
1IGC	L	27	1	−1
1IGC	L	28	1	−1
1IGC	L	29	0	−1
1IGC	L	30	1	−1
1IGC	L	31	1	−1
1IGC	L	32	1	−1
1IGC	L	33	0	−1
1IGC	L	34	0	−1
1IGC	L	35	0	−1
1IGC	L	36	0	−1
1IGC	L	37	0	−1
1IGC	L	38	0	−1
1IGC	L	39	0	−1
1IGC	L	40	1	−1
1IGC	L	41	1	−1
1IGC	L	42	1	−1
1IGC	L	43	0	−1
1IGC	L	44	0	−1
1IGC	L	45	1	−1
1IGC	L	46	0	−1
1IGC	L	47	0	−1
1IGC	L	48	0	−1
1IGC	L	49	0	−1
1IGC	L	50	0	−1
1IGC	L	51	0	−1
1IGC	L	52	1	−1
1IGC	L	53	1	−1
1IGC	L	54	1	−1
1IGC	L	55	0	−1
1IGC	L	56	1	−1
1IGC	L	57	1	−1
1IGC	L	58	0	−1
1IGC	L	59	1	−1
1IGC	L	60	1	−1
1IGC	L	61	0	−1
1IGC	L	62	0	−1
1IGC	L	63	1	−1
1IGC	L	64	0	−1
1IGC	L	65	1	−1

1IGC	L	66	1	−1
1IGC	L	67	1	−1
1IGC	L	68	0	−1
1IGC	L	69	1	−1
1IGC	L	70	1	−1
1IGC	L	71	0	−1
1IGC	L	72	1	−1
1IGC	L	73	0	−1
1IGC	L	74	0	−1
1IGC	L	75	0	−1
1IGC	L	76	1	−1
1IGC	L	77	1	−1
1IGC	L	78	0	−1
1IGC	L	79	1	−1
1IGC	L	80	0	−1
1IGC	L	81	1	−1
1IGC	L	82	0	−1
1IGC	L	83	0	−1
1IGC	L	84	0	−1
1IGC	L	85	0	−1
1IGC	L	86	0	−1
1IGC	L	87	0	−1
1IGC	L	88	0	−1
1IGC	L	89	0	−1
1IGC	L	90	0	−1
1IGC	L	91	0	−1
1IGC	L	92	1	−1
1IGC	L	93	1	−1
1IGC	L	94	1	−1
1IGC	L	95	0	−1
1IGC	L	96	0	−1
1IGC	L	97	0	−1
1IGC	L	98	0	−1
1IGC	L	99	0	−1
1IGC	L	100	1	−1
1IGC	L	101	0	−1
1IGC	L	102	0	−1
1IGC	L	103	0	−1
1IGC	L	104	0	−1
1IGC	L	105	1	−1
1IGC	L	106	0	−1
1IGC	L	107	1	−1
1IGC	L	108	1	−1
1IGC	L	109	1	−1
1IGC	L	110	1	−1
1IGC	L	111	0	−1
1IGC	L	112	1	−1
1IGC	L	113	0	−1
1IGC	L	114	1	−1
1IGC	L	115	0	−1
1IGC	L	116	0	−1
1IGC	L	117	0	−1

1IGC	L	118	0	−1
1IGC	L	119	0	−1
1IGC	L	120	0	−1
1IGC	L	121	0	−1
1IGC	L	122	1	−1
1IGC	L	123	1	1
1IGC	L	124	0	−1
1IGC	L	125	0	−1
1IGC	L	126	1	1
1IGC	L	127	1	1
1IGC	L	128	1	−1
1IGC	L	129	1	−1
1IGC	L	130	0	−1
1IGC	L	131	0	−1
1IGC	L	132	0	−1
1IGC	L	133	0	−1
1IGC	L	134	0	−1
1IGC	L	135	0	−1
1IGC	L	136	0	−1
1IGC	L	137	0	−1
1IGC	L	138	1	−1
1IGC	L	139	0	−1
1IGC	L	140	0	−1
1IGC	L	141	0	−1
1IGC	L	142	1	−1
1IGC	L	143	1	−1
1IGC	L	144	0	−1
1IGC	L	145	1	−1
1IGC	L	146	0	−1
1IGC	L	147	0	−1
1IGC	L	148	0	−1
1IGC	L	149	0	−1
1IGC	L	150	0	−1
1IGC	L	151	1	−1
1IGC	L	152	1	−1
1IGC	L	153	1	−1
1IGC	L	154	1	−1
1IGC	L	155	1	−1
1IGC	L	156	1	−1
1IGC	L	157	1	−1
1IGC	L	158	1	−1
1IGC	L	159	1	−1
1IGC	L	160	1	−1
1IGC	L	161	0	−1
1IGC	L	162	1	−1
1IGC	L	163	0	−1
1IGC	L	164	1	−1
1IGC	L	165	0	−1
1IGC	L	166	0	−1
1IGC	L	167	1	−1
1IGC	L	168	1	−1
1IGC	L	169	1	−1

1IGC	L	170	0	−1
1IGC	L	171	0	−1
1IGC	L	172	0	−1
1IGC	L	173	0	−1
1IGC	L	174	0	−1
1IGC	L	175	0	−1
1IGC	L	176	0	−1
1IGC	L	177	0	−1
1IGC	L	178	0	−1
1IGC	L	179	1	−1
1IGC	L	180	0	−1
1IGC	L	181	1	−1
1IGC	L	182	1	−1
1IGC	L	183	1	−1
1IGC	L	184	1	−1
1IGC	L	185	0	−1
1IGC	L	186	1	−1
1IGC	L	187	1	−1
1IGC	L	188	1	−1
1IGC	L	189	1	−1
1IGC	L	190	1	−1
1IGC	L	191	0	−1
1IGC	L	192	0	−1
1IGC	L	193	0	−1
1IGC	L	194	0	−1
1IGC	L	195	0	−1
1IGC	L	196	1	−1
1IGC	L	197	0	−1
1IGC	L	198	1	−1
1IGC	L	199	1	−1
1IGC	L	200	0	−1
1IGC	L	201	1	−1
1IGC	L	202	1	−1
1IGC	L	203	1	−1
1IGC	L	204	1	−1
1IGC	L	205	1	−1
1IGC	L	206	1	−1
1IGC	L	207	1	−1
1IGC	L	208	0	−1
1IGC	L	209	1	−1
1IGC	L	210	0	−1
1IGC	L	211	1	−1
1IGC	L	212	1	−1
1IGC	L	213	1	−1
1IGC	H	1	1	−1
1IGC	H	2	0	−1
1IGC	H	3	1	−1
1IGC	H	4	0	−1
1IGC	H	5	1	−1
1IGC	H	6	0	−1
1IGC	H	7	1	−1
1IGC	H	8	1	−1

1IGC	H	9	1	−1
1IGC	H	10	0	−1
1IGC	H	11	0	−1
1IGC	H	12	0	−1
1IGC	H	13	1	−1
1IGC	H	14	1	−1
1IGC	H	15	1	−1
1IGC	H	16	1	−1
1IGC	H	17	1	−1
1IGC	H	18	1	−1
1IGC	H	19	1	−1
1IGC	H	20	0	−1
1IGC	H	21	0	−1
1IGC	H	22	0	−1
1IGC	H	23	1	−1
1IGC	H	24	0	−1
1IGC	H	25	1	−1
1IGC	H	26	1	−1
1IGC	H	27	0	−1
1IGC	H	28	1	−1
1IGC	H	29	0	−1
1IGC	H	30	1	−1
1IGC	H	31	1	−1
1IGC	H	32	0	−1
1IGC	H	33	0	−1
1IGC	H	34	0	−1
1IGC	H	35	0	−1
1IGC	H	36	0	−1
1IGC	H	37	0	−1
1IGC	H	38	0	−1
1IGC	H	39	0	−1
1IGC	H	40	1	−1
1IGC	H	41	1	−1
1IGC	H	42	1	−1
1IGC	H	43	1	−1
1IGC	H	44	0	−1
1IGC	H	45	0	−1
1IGC	H	46	1	−1
1IGC	H	47	0	−1
1IGC	H	48	0	−1
1IGC	H	49	0	−1
1IGC	H	50	0	−1
1IGC	H	51	0	−1
1IGC	H	52	0	−1
1IGC	H	53	1	−1
1IGC	H	54	1	−1
1IGC	H	55	1	−1
1IGC	H	56	1	−1
1IGC	H	57	1	−1
1IGC	H	58	1	−1
1IGC	H	59	1	−1
1IGC	H	60	0	−1

1IGC	H	61	0	−1
1IGC	H	62	1	−1
1IGC	H	63	1	−1
1IGC	H	64	0	−1
1IGC	H	65	1	−1
1IGC	H	66	1	−1
1IGC	H	67	0	−1
1IGC	H	68	0	−1
1IGC	H	69	1	−1
1IGC	H	70	0	−1
1IGC	H	71	1	−1
1IGC	H	72	0	−1
1IGC	H	73	1	−1
1IGC	H	74	0	−1
1IGC	H	75	1	−1
1IGC	H	76	1	−1
1IGC	H	77	1	−1
1IGC	H	78	0	−1
1IGC	H	79	0	−1
1IGC	H	80	0	−1
1IGC	H	81	0	−1
1IGC	H	82	1	−1
1IGC	H	83	0	−1
1IGC	H	84	1	−1
1IGC	H	85	1	−1
1IGC	H	86	0	−1
1IGC	H	87	1	−1
1IGC	H	88	1	−1
1IGC	H	89	1	−1
1IGC	H	90	0	−1
1IGC	H	91	1	−1
1IGC	H	92	0	−1
1IGC	H	93	1	−1
1IGC	H	94	0	−1
1IGC	H	95	0	−1
1IGC	H	96	0	−1
1IGC	H	97	0	−1
1IGC	H	98	0	−1
1IGC	H	99	0	−1
1IGC	H	100	0	−1
1IGC	H	101	1	−1
1IGC	H	102	1	−1
1IGC	H	103	1	−1
1IGC	H	104	1	−1
1IGC	H	105	1	−1
1IGC	H	106	0	−1
1IGC	H	107	0	−1
1IGC	H	108	0	−1
1IGC	H	109	1	−1
1IGC	H	110	0	−1
1IGC	H	111	0	−1
1IGC	H	112	1	−1

1IGC	H	113	0	−1
1IGC	H	114	0	−1
1IGC	H	115	0	−1
1IGC	H	116	0	−1
1IGC	H	117	0	−1
1IGC	H	118	0	−1
1IGC	H	119	0	−1
1IGC	H	120	1	−1
1IGC	H	121	1	−1
1IGC	H	122	1	1
1IGC	H	123	1	−1
1IGC	H	124	1	−1
1IGC	H	125	1	1
1IGC	H	126	0	−1
1IGC	H	127	1	1
1IGC	H	128	0	−1
1IGC	H	129	0	−1
1IGC	H	130	0	−1
1IGC	H	131	0	−1
1IGC	H	132	0	−1
1IGC	H	133	0	−1
1IGC	H	134	0	−1
1IGC	H	135	1	−1
1IGC	H	136	1	−1
1IGC	H	137	1	−1
1IGC	H	138	0	−1
1IGC	H	139	1	−1
1IGC	H	140	1	−1
1IGC	H	141	1	−1
1IGC	H	142	1	−1
1IGC	H	143	0	−1
1IGC	H	144	0	−1
1IGC	H	145	0	−1
1IGC	H	146	0	−1
1IGC	H	147	0	−1
1IGC	H	148	0	−1
1IGC	H	149	0	−1
1IGC	H	150	0	−1
1IGC	H	151	0	−1
1IGC	H	152	0	−1
1IGC	H	153	0	−1
1IGC	H	154	0	−1
1IGC	H	155	1	−1
1IGC	H	156	1	−1
1IGC	H	157	0	−1
1IGC	H	158	1	−1
1IGC	H	159	0	−1
1IGC	H	160	1	−1
1IGC	H	161	0	−1
1IGC	H	162	1	−1
1IGC	H	163	1	−1
1IGC	H	164	1	−1

1IGC	H	165	1	−1
1IGC	H	166	0	−1
1IGC	H	167	1	−1
1IGC	H	168	1	−1
1IGC	H	169	1	−1
1IGC	H	170	1	−1
1IGC	H	171	0	−1
1IGC	H	172	1	−1
1IGC	H	173	0	−1
1IGC	H	174	1	−1
1IGC	H	175	0	−1
1IGC	H	176	0	−1
1IGC	H	177	1	−1
1IGC	H	178	1	−1
1IGC	H	179	1	−1
1IGC	H	180	1	−1
1IGC	H	181	1	−1
1IGC	H	182	0	−1
1IGC	H	183	0	−1
1IGC	H	184	0	−1
1IGC	H	185	0	−1
1IGC	H	186	0	−1
1IGC	H	187	0	−1
1IGC	H	188	0	−1
1IGC	H	189	0	−1
1IGC	H	190	0	−1
1IGC	H	191	1	−1
1IGC	H	192	1	−1
1IGC	H	193	1	−1
1IGC	H	194	0	−1
1IGC	H	195	0	−1
1IGC	H	196	1	−1
1IGC	H	197	1	−1
1IGC	H	198	1	−1
1IGC	H	199	1	−1
1IGC	H	200	0	−1
1IGC	H	201	0	−1
1IGC	H	202	0	−1
1IGC	H	203	0	−1
1IGC	H	204	0	−1
1IGC	H	205	1	−1
1IGC	H	206	0	−1
1IGC	H	207	1	−1
1IGC	H	208	1	−1
1IGC	H	209	0	−1
1IGC	H	210	1	1
1IGC	H	211	0	−1
1IGC	H	212	1	1
1IGC	H	213	1	1
1IGC	H	214	1	1
1IGC	H	215	0	−1
1IGC	H	216	1	1

1IGC	H	217	0	−1
1IGC	H	218	1	1
1IGC	H	219	0	−1
1IGC	H	220	1	−1
1IGC	H	221	1	−1
1IGC	H	222	1	−1
1JH1	A	1	1	−1
1JH1	A	2	1	−1
1JH1	A	3	1	−1
1JH1	A	4	0	−1
1JH1	A	5	1	−1
1JH1	A	6	1	−1
1JH1	A	7	1	−1
1JH1	A	8	1	−1
1JH1	A	9	0	−1
1JH1	A	10	1	−1
1JH1	A	11	0	−1
1JH1	A	12	0	−1
1JH1	A	13	0	−1
1JH1	A	14	1	−1
1JH1	A	15	1	−1
1JH1	A	16	1	−1
1JH1	A	17	0	−1
1JH1	A	18	1	−1
1JH1	A	19	1	−1
1JH1	A	20	0	−1
1JH1	A	21	1	−1
1JH1	A	22	1	−1
1JH1	A	23	1	−1
1JH1	A	24	1	−1
1JH1	A	25	0	−1
1JH1	A	26	0	−1
1JH1	A	27	1	−1
1JH1	A	28	0	−1
1JH1	A	29	0	−1
1JH1	A	30	1	−1
1JH1	A	31	1	−1
1JH1	A	32	0	−1
1JH1	A	33	0	−1
1JH1	A	34	1	−1
1JH1	A	35	0	−1
1JH1	A	36	0	−1
1JH1	A	37	0	−1
1JH1	A	38	1	−1
1JH1	A	39	0	−1
1JH1	A	40	0	−1
1JH1	A	41	1	−1
1JH1	A	42	0	−1
1JH1	A	43	1	−1
1JH1	A	44	0	−1
1JH1	A	45	1	−1
1JH1	A	46	1	−1

1JH1	A	47	1	−1
1JH1	A	48	1	−1
1JH1	A	49	1	−1
1JH1	A	50	1	−1
1JH1	A	51	1	−1
1JH1	A	52	0	−1
1JH1	A	53	0	−1
1JH1	A	54	0	−1
1JH1	A	55	0	−1
1JH1	A	56	0	−1
1JH1	A	57	0	−1
1JH1	A	58	0	−1
1JH1	A	59	1	−1
1JH1	A	60	1	−1
1JH1	A	61	1	−1
1JH1	A	62	1	−1
1JH1	A	63	0	−1
1JH1	A	64	1	−1
1JH1	A	65	0	−1
1JH1	A	66	1	−1
1JH1	A	67	1	−1
1JH1	A	68	1	−1
1JH1	A	69	0	−1
1JH1	A	70	1	−1
1JH1	A	71	0	−1
1JH1	A	72	1	−1
1JH1	A	73	1	−1
1JH1	A	74	1	−1
1JH1	A	75	1	−1
1JH1	A	76	0	−1
1JH1	A	77	0	−1
1JH1	A	78	0	−1
1JH1	A	79	0	−1
1JH1	A	80	0	−1
1JH1	A	81	1	−1
1JH1	A	82	0	−1
1JH1	A	83	1	−1
1JH1	A	84	1	−1
1JH1	A	85	1	−1
1JH1	A	86	1	−1
1JH1	A	87	0	−1
1JH1	A	88	0	−1
1JH1	A	89	0	−1
1JH1	A	90	0	−1
1JH1	A	91	0	−1
1JH1	A	92	0	−1
1JH1	A	93	0	−1
1JH1	A	94	0	−1
1JH1	A	95	0	−1
1JH1	A	96	0	−1
1JH1	A	97	1	−1
1JH1	A	98	0	−1

1JH1	A	99	0	−1
1JH1	A	100	1	−1
1JH1	A	101	1	−1
1JH1	A	102	1	−1
1JH1	A	103	1	−1
1JH1	A	104	1	−1
1JH1	A	105	0	−1
1JH1	A	106	0	−1
1JH1	A	107	0	−1
1JH1	A	108	0	−1
1JH1	A	109	0	−1
1JH1	A	110	0	−1
1JH1	A	111	0	−1
1JH1	A	112	0	−1
1JH1	A	113	0	−1
1JH1	A	114	0	−1
1JH1	A	115	0	−1
1JH1	A	116	0	−1
1JH1	A	117	0	−1
1JH1	A	118	0	−1
1JH1	A	119	0	−1
1JH1	A	120	0	−1
1JH1	A	121	0	−1
1JH1	A	122	1	−1
1JH1	A	123	1	−1
1JH1	A	124	0	−1
1JH1	A	125	1	−1
1JH1	A	126	1	−1
1JH1	A	127	1	−1
1JH1	A	128	1	−1
1JH1	A	129	0	−1
1JH1	A	130	0	−1
1JH1	A	131	0	−1
1JH1	A	132	1	−1
1JH1	A	133	1	−1
1JH1	A	134	1	−1
1JH1	A	135	1	−1
1JH1	A	136	1	−1
1JH1	A	137	1	−1
1JH1	A	138	1	−1
1JH1	A	139	1	−1
1JH1	A	140	1	−1
1JH1	A	141	1	−1
1JH1	A	142	1	−1
1JH1	A	143	0	−1
1JH1	A	144	1	−1
1JH1	A	145	0	−1
1JH1	A	146	0	−1
1JH1	A	147	1	−1
1JH1	A	148	1	−1
1JH1	A	149	0	−1
1JH1	A	150	0	−1

1JH1	A	151	1	−1
1JH1	A	152	0	−1
1JH1	A	153	0	−1
1JH1	A	154	1	−1
1JH1	A	155	1	−1
1JH1	A	156	0	−1
1JH1	A	157	0	−1
1JH1	A	158	1	−1
1KB5	A	1	1	1
1KB5	A	2	1	1
1KB5	A	3	0	−1
1KB5	A	4	0	−1
1KB5	A	5	0	−1
1KB5	A	6	1	−1
1KB5	A	7	1	−1
1KB5	A	8	1	−1
1KB5	A	9	1	−1
1KB5	A	10	0	−1
1KB5	A	11	1	−1
1KB5	A	12	0	−1
1KB5	A	13	1	−1
1KB5	A	14	1	−1
1KB5	A	15	1	−1
1KB5	A	16	1	−1
1KB5	A	17	1	−1
1KB5	A	18	0	−1
1KB5	A	19	1	−1
1KB5	A	20	0	−1
1KB5	A	21	1	−1
1KB5	A	22	0	−1
1KB5	A	23	1	−1
1KB5	A	24	0	−1
1KB5	A	25	1	1
1KB5	A	26	1	1
1KB5	A	27	1	−1
1KB5	A	28	1	−1
1KB5	A	29	0	−1
1KB5	A	30	0	−1
1KB5	A	31	0	−1
1KB5	A	32	0	−1
1KB5	A	33	0	−1
1KB5	A	34	0	−1
1KB5	A	35	0	−1
1KB5	A	36	0	−1
1KB5	A	37	0	−1
1KB5	A	38	1	−1
1KB5	A	39	1	−1
1KB5	A	40	1	−1
1KB5	A	41	1	−1
1KB5	A	42	0	−1
1KB5	A	43	0	−1
1KB5	A	44	1	−1

1KB5	A	45	1	−1
1KB5	A	46	0	−1
1KB5	A	47	0	−1
1KB5	A	48	0	−1
1KB5	A	49	0	−1
1KB5	A	50	1	−1
1KB5	A	51	0	−1
1KB5	A	52	1	−1
1KB5	A	53	1	−1
1KB5	A	54	1	−1
1KB5	A	55	1	−1
1KB5	A	56	1	−1
1KB5	A	57	1	−1
1KB5	A	58	1	−1
1KB5	A	59	1	−1
1KB5	A	60	0	−1
1KB5	A	61	0	−1
1KB5	A	62	0	−1
1KB5	A	63	0	−1
1KB5	A	64	1	−1
1KB5	A	65	0	−1
1KB5	A	66	1	−1
1KB5	A	67	1	−1
1KB5	A	68	1	−1
1KB5	A	69	1	−1
1KB5	A	70	1	−1
1KB5	A	71	1	−1
1KB5	A	72	0	−1
1KB5	A	73	0	−1
1KB5	A	74	0	−1
1KB5	A	75	0	−1
1KB5	A	76	0	−1
1KB5	A	77	1	−1
1KB5	A	78	1	−1
1KB5	A	79	0	−1
1KB5	A	80	1	−1
1KB5	A	81	1	−1
1KB5	A	82	1	−1
1KB5	A	83	0	−1
1KB5	A	84	0	−1
1KB5	A	85	0	−1
1KB5	A	86	0	−1
1KB5	A	87	0	−1
1KB5	A	88	0	−1
1KB5	A	89	0	−1
1KB5	A	90	0	−1
1KB5	A	91	0	−1
1KB5	A	92	0	−1
1KB5	A	93	1	1
1KB5	A	94	1	1
1KB5	A	95	0	−1
1KB5	A	96	0	−1

1KB5	A	97	1	1
1KB5	A	98	0	−1
1KB5	A	99	0	−1
1KB5	A	100	1	1
1KB5	A	101	0	−1
1KB5	A	102	0	−1
1KB5	A	103	0	−1
1KB5	A	104	0	−1
1KB5	A	105	0	−1
1KB5	A	106	1	−1
1KB5	A	107	0	−1
1KB5	A	108	0	−1
1KB5	A	109	0	−1
1KB5	A	110	1	−1
1KB5	A	111	0	−1
1KB5	A	112	1	−1
1KB5	A	113	1	−1
1KB5	A	114	1	−1
1KB5	A	115	1	−1
1KB5	B	1	1	−1
1KB5	B	2	1	−1
1KB5	B	3	1	−1
1KB5	B	4	0	−1
1KB5	B	5	1	−1
1KB5	B	6	0	−1
1KB5	B	7	1	−1
1KB5	B	8	0	−1
1KB5	B	9	1	−1
1KB5	B	10	1	−1
1KB5	B	11	1	−1
1KB5	B	12	1	−1
1KB5	B	13	0	−1
1KB5	B	14	1	−1
1KB5	B	15	1	−1
1KB5	B	16	1	−1
1KB5	B	17	1	−1
1KB5	B	18	1	−1
1KB5	B	19	0	−1
1KB5	B	20	1	−1
1KB5	B	21	0	−1
1KB5	B	22	1	−1
1KB5	B	23	0	−1
1KB5	B	24	0	−1
1KB5	B	25	0	−1
1KB5	B	26	1	−1
1KB5	B	27	1	−1
1KB5	B	28	1	−1
1KB5	B	29	1	−1
1KB5	B	30	0	−1
1KB5	B	31	0	−1
1KB5	B	32	0	−1
1KB5	B	33	0	−1

1KB5	B	34	0	−1
1KB5	B	35	0	−1
1KB5	B	36	0	−1
1KB5	B	37	0	−1
1KB5	B	38	0	−1
1KB5	B	39	0	−1
1KB5	B	40	1	−1
1KB5	B	41	1	−1
1KB5	B	42	1	−1
1KB5	B	43	1	1
1KB5	B	44	0	−1
1KB5	B	45	1	1
1KB5	B	46	1	1
1KB5	B	47	0	−1
1KB5	B	48	0	−1
1KB5	B	49	0	−1
1KB5	B	50	0	−1
1KB5	B	51	1	1
1KB5	B	52	1	1
1KB5	B	53	1	1
1KB5	B	54	1	−1
1KB5	B	55	1	1
1KB5	B	56	1	1
1KB5	B	57	1	1
1KB5	B	58	1	1
1KB5	B	59	1	1
1KB5	B	60	1	1
1KB5	B	61	0	−1
1KB5	B	62	1	1
1KB5	B	63	0	−1
1KB5	B	64	0	−1
1KB5	B	65	1	−1
1KB5	B	66	0	−1
1KB5	B	67	1	−1
1KB5	B	68	0	−1
1KB5	B	69	1	−1
1KB5	B	70	0	−1
1KB5	B	71	1	−1
1KB5	B	72	1	−1
1KB5	B	73	1	−1
1KB5	B	74	1	−1
1KB5	B	75	1	−1
1KB5	B	76	0	−1
1KB5	B	77	1	−1
1KB5	B	78	0	−1
1KB5	B	79	1	−1
1KB5	B	80	0	−1
1KB5	B	81	0	−1
1KB5	B	82	1	−1
1KB5	B	83	0	−1
1KB5	B	84	1	−1
1KB5	B	85	1	−1

1KB5	B	86	0	−1
1KB5	B	87	0	−1
1KB5	B	88	1	−1
1KB5	B	89	0	−1
1KB5	B	90	0	−1
1KB5	B	91	0	−1
1KB5	B	92	0	−1
1KB5	B	93	0	−1
1KB5	B	94	0	−1
1KB5	B	95	1	−1
1KB5	B	96	1	−1
1KB5	B	97	0	−1
1KB5	B	98	1	−1
1KB5	B	99	1	1
1KB5	B	100	1	−1
1KB5	B	101	1	−1
1KB5	B	102	0	−1
1KB5	B	103	0	−1
1KB5	B	104	0	−1
1KB5	B	105	1	−1
1KB5	B	106	0	−1
1KB5	B	107	1	−1
1KB5	B	108	0	−1
1KB5	B	109	0	−1
1KB5	B	110	1	−1
1KB5	B	111	0	−1
1KB5	B	112	0	−1
1KB5	B	113	1	−1
1KB5	B	114	0	−1
1KB5	B	115	0	−1
1KB5	B	116	1	−1
1KB5	B	117	1	−1
1KB5	L	1	1	−1
1KB5	L	2	0	−1
1KB5	L	3	1	−1
1KB5	L	4	0	−1
1KB5	L	5	1	−1
1KB5	L	6	0	−1
1KB5	L	7	1	−1
1KB5	L	8	1	−1
1KB5	L	9	1	−1
1KB5	L	10	1	−1
1KB5	L	11	0	−1
1KB5	L	12	1	−1
1KB5	L	13	0	−1
1KB5	L	14	1	−1
1KB5	L	15	1	−1
1KB5	L	16	1	−1
1KB5	L	17	1	−1
1KB5	L	18	1	−1
1KB5	L	19	1	−1
1KB5	L	20	1	−1

1KB5	L	21	0	−1
1KB5	L	22	0	−1
1KB5	L	23	0	−1
1KB5	L	24	1	−1
1KB5	L	25	0	−1
1KB5	L	26	1	−1
1KB5	L	27	1	1
1KB5	L	28	1	−1
1KB5	L	29	0	−1
1KB5	L	30	1	1
1KB5	L	31	1	1
1KB5	L	32	1	1
1KB5	L	33	0	−1
1KB5	L	34	0	−1
1KB5	L	35	0	−1
1KB5	L	36	0	−1
1KB5	L	37	0	−1
1KB5	L	38	0	−1
1KB5	L	39	1	−1
1KB5	L	40	1	−1
1KB5	L	41	1	−1
1KB5	L	42	1	−1
1KB5	L	43	0	−1
1KB5	L	44	0	−1
1KB5	L	45	1	−1
1KB5	L	46	0	−1
1KB5	L	47	0	−1
1KB5	L	48	0	−1
1KB5	L	49	0	−1
1KB5	L	50	1	1
1KB5	L	51	0	−1
1KB5	L	52	1	−1
1KB5	L	53	1	1
1KB5	L	54	1	−1
1KB5	L	55	0	−1
1KB5	L	56	1	−1
1KB5	L	57	1	−1
1KB5	L	58	0	−1
1KB5	L	59	1	−1
1KB5	L	60	1	−1
1KB5	L	61	0	−1
1KB5	L	62	0	−1
1KB5	L	63	1	−1
1KB5	L	64	0	−1
1KB5	L	65	1	−1
1KB5	L	66	1	−1
1KB5	L	67	1	−1
1KB5	L	68	0	−1
1KB5	L	69	1	−1
1KB5	L	70	1	−1
1KB5	L	71	0	−1
1KB5	L	72	0	−1

1KB5	L	73	0	−1
1KB5	L	74	1	−1
1KB5	L	75	0	−1
1KB5	L	76	1	−1
1KB5	L	77	1	−1
1KB5	L	78	0	−1
1KB5	L	79	1	−1
1KB5	L	80	0	−1
1KB5	L	81	1	−1
1KB5	L	82	0	−1
1KB5	L	83	0	−1
1KB5	L	84	0	−1
1KB5	L	85	0	−1
1KB5	L	86	0	−1
1KB5	L	87	0	−1
1KB5	L	88	0	−1
1KB5	L	89	0	−1
1KB5	L	90	0	−1
1KB5	L	91	0	−1
1KB5	L	92	1	1
1KB5	L	93	1	1
1KB5	L	94	1	1
1KB5	L	95	0	−1
1KB5	L	96	0	−1
1KB5	L	97	0	−1
1KB5	L	98	0	−1
1KB5	L	99	0	−1
1KB5	L	100	1	−1
1KB5	L	101	0	−1
1KB5	L	102	0	−1
1KB5	L	103	1	−1
1KB5	L	104	0	−1
1KB5	L	105	0	−1
1KB5	L	106	0	−1
1KB5	L	107	1	−1
1KB5	L	108	1	−1
1KB5	L	109	1	−1
1KB5	L	110	1	−1
1KB5	L	111	0	−1
1KB5	L	112	1	−1
1KB5	L	113	0	−1
1KB5	L	114	1	−1
1KB5	L	115	0	−1
1KB5	L	116	0	−1
1KB5	L	117	0	−1
1KB5	L	118	0	−1
1KB5	L	119	0	−1
1KB5	L	120	0	−1
1KB5	L	121	0	−1
1KB5	L	122	1	−1
1KB5	L	123	1	−1
1KB5	L	124	0	−1

1KB5	L	125	0	−1
1KB5	L	126	1	−1
1KB5	L	127	1	−1
1KB5	L	128	1	−1
1KB5	L	129	0	−1
1KB5	L	130	0	−1
1KB5	L	131	0	−1
1KB5	L	132	0	−1
1KB5	L	133	0	−1
1KB5	L	134	0	−1
1KB5	L	135	0	−1
1KB5	L	136	0	−1
1KB5	L	137	0	−1
1KB5	L	138	1	−1
1KB5	L	139	0	−1
1KB5	L	140	0	−1
1KB5	L	141	0	−1
1KB5	L	142	1	−1
1KB5	L	143	1	−1
1KB5	L	144	0	−1
1KB5	L	145	1	−1
1KB5	L	146	0	−1
1KB5	L	147	1	−1
1KB5	L	148	0	−1
1KB5	L	149	1	−1
1KB5	L	150	0	−1
1KB5	L	151	1	−1
1KB5	L	152	1	−1
1KB5	L	153	1	−1
1KB5	L	154	1	−1
1KB5	L	155	0	−1
1KB5	L	156	1	−1
1KB5	L	157	1	−1
1KB5	L	158	1	−1
1KB5	L	159	1	−1
1KB5	L	160	1	−1
1KB5	L	161	1	−1
1KB5	L	162	0	−1
1KB5	L	163	1	−1
1KB5	L	164	0	−1
1KB5	L	165	1	−1
1KB5	L	166	0	−1
1KB5	L	167	1	−1
1KB5	L	168	1	−1
1KB5	L	169	1	−1
1KB5	L	170	1	−1
1KB5	L	171	0	−1
1KB5	L	172	0	−1
1KB5	L	173	0	−1
1KB5	L	174	0	−1
1KB5	L	175	0	−1
1KB5	L	176	0	−1

1KB5	L	177	0	−1
1KB5	L	178	0	−1
1KB5	L	179	0	−1
1KB5	L	180	1	−1
1KB5	L	181	0	−1
1KB5	L	182	1	−1
1KB5	L	183	1	−1
1KB5	L	184	1	−1
1KB5	L	185	0	−1
1KB5	L	186	0	−1
1KB5	L	187	1	−1
1KB5	L	188	1	−1
1KB5	L	189	1	−1
1KB5	L	190	0	−1
1KB5	L	191	1	−1
1KB5	L	192	0	−1
1KB5	L	193	0	−1
1KB5	L	194	0	−1
1KB5	L	195	0	−1
1KB5	L	196	0	−1
1KB5	L	197	1	−1
1KB5	L	198	0	−1
1KB5	L	199	1	−1
1KB5	L	200	1	−1
1KB5	L	201	1	−1
1KB5	L	202	1	−1
1KB5	L	203	1	−1
1KB5	L	204	1	−1
1KB5	L	205	0	−1
1KB5	L	206	1	−1
1KB5	L	207	1	−1
1KB5	L	208	1	−1
1KB5	L	209	0	−1
1KB5	L	210	1	−1
1KB5	L	211	0	−1
1KB5	L	212	1	−1
1KB5	L	213	1	−1
1KB5	L	214	1	−1
1KB5	H	1	1	−1
1KB5	H	2	0	−1
1KB5	H	3	1	−1
1KB5	H	4	0	−1
1KB5	H	5	1	−1
1KB5	H	6	0	−1
1KB5	H	7	0	−1
1KB5	H	8	1	−1
1KB5	H	9	1	−1
1KB5	H	10	0	−1
1KB5	H	11	1	−1
1KB5	H	12	1	−1
1KB5	H	13	1	−1
1KB5	H	14	1	−1

1KB5	H	15	1	−1
1KB5	H	16	0	−1
1KB5	H	17	1	−1
1KB5	H	18	0	−1
1KB5	H	19	1	−1
1KB5	H	20	0	−1
1KB5	H	21	0	−1
1KB5	H	22	0	−1
1KB5	H	23	1	−1
1KB5	H	24	0	−1
1KB5	H	25	0	−1
1KB5	H	26	1	−1
1KB5	H	27	0	−1
1KB5	H	28	1	1
1KB5	H	29	0	−1
1KB5	H	30	1	1
1KB5	H	31	1	1
1KB5	H	32	1	1
1KB5	H	33	0	−1
1KB5	H	34	0	−1
1KB5	H	35	0	−1
1KB5	H	36	0	−1
1KB5	H	37	0	−1
1KB5	H	38	0	−1
1KB5	H	39	0	−1
1KB5	H	40	0	−1
1KB5	H	41	1	−1
1KB5	H	42	1	−1
1KB5	H	43	1	−1
1KB5	H	44	1	−1
1KB5	H	45	0	−1
1KB5	H	46	1	−1
1KB5	H	47	0	−1
1KB5	H	48	0	−1
1KB5	H	49	0	−1
1KB5	H	50	0	−1
1KB5	H	51	0	−1
1KB5	H	52	0	−1
1KB5	H	53	0	−1
1KB5	H	54	1	1
1KB5	H	55	1	1
1KB5	H	56	1	1
1KB5	H	57	1	1
1KB5	H	58	1	1
1KB5	H	59	1	1
1KB5	H	60	0	−1
1KB5	H	61	0	−1
1KB5	H	62	1	1
1KB5	H	63	1	−1
1KB5	H	64	0	−1
1KB5	H	65	1	1
1KB5	H	66	1	−1

1KB5	H	67	1	−1
1KB5	H	68	0	−1
1KB5	H	69	1	−1
1KB5	H	70	0	−1
1KB5	H	71	1	−1
1KB5	H	72	0	−1
1KB5	H	73	1	−1
1KB5	H	74	1	−1
1KB5	H	75	1	−1
1KB5	H	76	1	−1
1KB5	H	77	1	−1
1KB5	H	78	0	−1
1KB5	H	79	0	−1
1KB5	H	80	1	−1
1KB5	H	81	0	−1
1KB5	H	82	1	−1
1KB5	H	83	0	−1
1KB5	H	84	1	−1
1KB5	H	85	1	−1
1KB5	H	86	0	−1
1KB5	H	87	1	−1
1KB5	H	88	1	−1
1KB5	H	89	1	−1
1KB5	H	90	0	−1
1KB5	H	91	1	−1
1KB5	H	92	0	−1
1KB5	H	93	1	−1
1KB5	H	94	0	−1
1KB5	H	95	0	−1
1KB5	H	96	0	−1
1KB5	H	97	0	−1
1KB5	H	98	0	−1
1KB5	H	99	0	−1
1KB5	H	100	1	1
1KB5	H	101	1	1
1KB5	H	102	1	1
1KB5	H	103	0	−1
1KB5	H	104	0	−1
1KB5	H	105	0	−1
1KB5	H	106	0	−1
1KB5	H	107	0	−1
1KB5	H	108	1	−1
1KB5	H	109	0	−1
1KB5	H	110	0	−1
1KB5	H	111	1	−1
1KB5	H	112	0	−1
1KB5	H	113	0	−1
1KB5	H	114	1	−1
1KB5	H	115	0	−1
1KB5	H	116	0	−1
1KB5	H	117	0	−1
1KB5	H	118	0	−1

1KB5	H	119	1	−1
1KB5	H	120	0	−1
1KB5	H	121	1	−1
1KB5	H	122	1	−1
1KB5	H	123	1	−1
1KB5	H	124	1	−1
1KB5	H	125	0	−1
1KB5	H	126	1	−1
1KB5	H	127	0	−1
1KB5	H	128	0	−1
1KB5	H	129	0	−1
1KB5	H	130	0	−1
1KB5	H	131	0	−1
1KB5	H	132	1	−1
1KB5	H	133	1	−1
1KB5	H	134	1	−1
1KB5	H	135	1	−1
1KB5	H	136	1	−1
1KB5	H	137	1	−1
1KB5	H	138	1	−1
1KB5	H	139	1	−1
1KB5	H	140	1	−1
1KB5	H	141	1	−1
1KB5	H	142	0	−1
1KB5	H	143	0	−1
1KB5	H	144	0	−1
1KB5	H	145	0	−1
1KB5	H	146	0	−1
1KB5	H	147	0	−1
1KB5	H	148	0	−1
1KB5	H	149	0	−1
1KB5	H	150	0	−1
1KB5	H	151	0	−1
1KB5	H	152	0	−1
1KB5	H	153	0	−1
1KB5	H	154	1	−1
1KB5	H	155	0	−1
1KB5	H	156	0	−1
1KB5	H	157	1	−1
1KB5	H	158	0	−1
1KB5	H	159	1	−1
1KB5	H	160	0	−1
1KB5	H	161	0	−1
1KB5	H	162	1	−1
1KB5	H	163	1	−1
1KB5	H	164	1	−1
1KB5	H	165	0	−1
1KB5	H	166	1	−1
1KB5	H	167	1	−1
1KB5	H	168	1	−1
1KB5	H	169	1	−1
1KB5	H	170	0	−1

1KB5	H	171	1	−1
1KB5	H	172	0	−1
1KB5	H	173	1	−1
1KB5	H	174	1	−1
1KB5	H	175	1	−1
1KB5	H	176	1	−1
1KB5	H	177	0	−1
1KB5	H	178	1	−1
1KB5	H	179	1	−1
1KB5	H	180	1	−1
1KB5	H	181	0	−1
1KB5	H	182	0	−1
1KB5	H	183	0	−1
1KB5	H	184	0	−1
1KB5	H	185	0	−1
1KB5	H	186	0	−1
1KB5	H	187	0	−1
1KB5	H	188	1	−1
1KB5	H	189	0	−1
1KB5	H	190	1	−1
1KB5	H	191	1	−1
1KB5	H	192	1	−1
1KB5	H	193	1	−1
1KB5	H	194	0	−1
1KB5	H	195	1	−1
1KB5	H	196	1	−1
1KB5	H	197	1	−1
1KB5	H	198	1	−1
1KB5	H	199	0	−1
1KB5	H	200	0	−1
1KB5	H	201	0	−1
1KB5	H	202	0	−1
1KB5	H	203	0	−1
1KB5	H	204	1	−1
1KB5	H	205	0	−1
1KB5	H	206	1	−1
1KB5	H	207	1	−1
1KB5	H	208	1	−1
1KB5	H	209	1	−1
1KB5	H	210	0	−1
1KB5	H	211	1	−1
1KB5	H	212	0	−1
1KB5	H	213	1	−1
1KB5	H	214	1	−1
1KB5	H	215	1	−1
1KB5	H	216	0	−1
1KB5	H	217	1	−1
1KB5	H	218	1	−1
1KB5	H	219	1	−1
1MCT	A	1	0	−1
1MCT	A	2	0	−1
1MCT	A	3	1	−1

1MCT	A	4	1	−1
1MCT	A	5	1	−1
1MCT	A	6	1	−1
1MCT	A	7	0	−1
1MCT	A	8	1	−1
1MCT	A	9	1	−1
1MCT	A	10	1	−1
1MCT	A	11	1	−1
1MCT	A	12	0	−1
1MCT	A	13	0	−1
1MCT	A	14	0	−1
1MCT	A	15	0	−1
1MCT	A	16	0	−1
1MCT	A	17	0	−1
1MCT	A	18	0	−1
1MCT	A	19	0	−1
1MCT	A	20	0	−1
1MCT	A	21	1	−1
1MCT	A	22	1	1
1MCT	A	23	0	−1
1MCT	A	24	0	−1
1MCT	A	25	0	−1
1MCT	A	26	0	−1
1MCT	A	27	0	−1
1MCT	A	28	0	−1
1MCT	A	29	0	−1
1MCT	A	30	0	−1
1MCT	A	31	1	−1
1MCT	A	32	1	−1
1MCT	A	33	1	−1
1MCT	A	34	0	−1
1MCT	A	35	0	−1
1MCT	A	36	0	−1
1MCT	A	37	0	−1
1MCT	A	38	0	−1
1MCT	A	39	0	−1
1MCT	A	40	1	1
1MCT	A	41	0	−1
1MCT	A	42	1	−1
1MCT	A	43	1	1
1MCT	A	44	1	−1
1MCT	A	45	1	−1
1MCT	A	46	0	−1
1MCT	A	47	0	−1
1MCT	A	48	0	−1
1MCT	A	49	0	−1
1MCT	A	50	0	−1
1MCT	A	51	0	−1
1MCT	A	52	0	−1
1MCT	A	53	0	−1
1MCT	A	54	0	−1
1MCT	A	55	0	−1

1MCT	A	56	1	−1
1MCT	A	57	1	−1
1MCT	A	58	1	−1
1MCT	A	59	1	−1
1MCT	A	60	1	−1
1MCT	A	61	1	−1
1MCT	A	62	0	−1
1MCT	A	63	0	−1
1MCT	A	64	1	−1
1MCT	A	65	0	−1
1MCT	A	66	1	−1
1MCT	A	67	0	−1
1MCT	A	68	1	−1
1MCT	A	69	1	−1
1MCT	A	70	0	−1
1MCT	A	71	1	−1
1MCT	A	72	1	−1
1MCT	A	73	0	−1
1MCT	A	74	1	−1
1MCT	A	75	1	−1
1MCT	A	76	1	−1
1MCT	A	77	1	−1
1MCT	A	78	1	1
1MCT	A	79	1	1
1MCT	A	80	1	1
1MCT	A	81	0	−1
1MCT	A	82	0	−1
1MCT	A	83	0	−1
1MCT	A	84	0	−1
1MCT	A	85	0	−1
1MCT	A	86	0	−1
1MCT	A	87	0	−1
1MCT	A	88	0	−1
1MCT	A	89	0	−1
1MCT	A	90	0	−1
1MCT	A	91	1	−1
1MCT	A	92	1	−1
1MCT	A	93	1	−1
1MCT	A	94	0	−1
1MCT	A	95	1	−1
1MCT	A	96	1	−1
1MCT	A	97	1	−1
1MCT	A	98	1	−1
1MCT	A	99	0	−1
1MCT	A	100	0	−1
1MCT	A	101	1	−1
1MCT	A	102	1	−1
1MCT	A	103	0	−1
1MCT	A	104	1	−1
1MCT	A	105	0	−1
1MCT	A	106	0	−1
1MCT	A	107	1	−1

1MCT	A	108	1	−1
1MCT	A	109	1	−1
1MCT	A	110	1	−1
1MCT	A	111	1	−1
1MCT	A	112	1	−1
1MCT	A	113	1	−1
1MCT	A	114	0	−1
1MCT	A	115	1	−1
1MCT	A	116	0	−1
1MCT	A	117	0	−1
1MCT	A	118	0	−1
1MCT	A	119	0	−1
1MCT	A	120	0	−1
1MCT	A	121	0	−1
1MCT	A	122	0	−1
1MCT	A	123	0	−1
1MCT	A	124	1	−1
1MCT	A	125	1	−1
1MCT	A	126	1	−1
1MCT	A	127	1	−1
1MCT	A	128	1	−1
1MCT	A	129	1	1
1MCT	A	130	1	−1
1MCT	A	131	1	1
1MCT	A	132	0	−1
1MCT	A	133	1	−1
1MCT	A	134	1	−1
1MCT	A	135	0	−1
1MCT	A	136	0	−1
1MCT	A	137	0	−1
1MCT	A	138	0	−1
1MCT	A	139	1	−1
1MCT	A	140	0	−1
1MCT	A	141	1	−1
1MCT	A	142	0	−1
1MCT	A	143	0	−1
1MCT	A	144	1	−1
1MCT	A	145	1	−1
1MCT	A	146	1	−1
1MCT	A	147	1	−1
1MCT	A	148	0	−1
1MCT	A	149	1	−1
1MCT	A	150	1	−1
1MCT	A	151	0	−1
1MCT	A	152	0	−1
1MCT	A	153	1	−1
1MCT	A	154	1	−1
1MCT	A	155	1	1
1MCT	A	156	0	−1
1MCT	A	157	1	−1
1MCT	A	158	1	−1
1MCT	A	159	0	−1

1MCT	A	160	0	−1
1MCT	A	161	0	−1
1MCT	A	162	0	−1
1MCT	A	163	0	−1
1MCT	A	164	0	−1
1MCT	A	165	0	−1
1MCT	A	166	1	−1
1MCT	A	167	1	−1
1MCT	A	168	0	−1
1MCT	A	169	1	−1
1MCT	A	170	1	−1
1MCT	A	171	0	−1
1MCT	A	172	0	−1
1MCT	A	173	0	−1
1MCT	A	174	1	1
1MCT	A	175	0	−1
1MCT	A	176	0	−1
1MCT	A	177	0	−1
1MCT	A	178	0	−1
1MCT	A	179	0	−1
1MCT	A	180	0	−1
1MCT	A	181	0	−1
1MCT	A	182	0	−1
1MCT	A	183	0	−1
1MCT	A	184	1	−1
1MCT	A	185	1	−1
1MCT	A	186	0	−1
1MCT	A	187	0	−1
1MCT	A	188	0	−1
1MCT	A	189	0	−1
1MCT	A	190	0	−1
1MCT	A	191	0	−1
1MCT	A	192	0	−1
1MCT	A	193	0	−1
1MCT	A	194	1	1
1MCT	A	195	1	1
1MCT	A	196	1	1
1MCT	A	197	0	−1
1MCT	A	198	0	−1
1MCT	A	199	1	−1
1MCT	A	200	1	−1
1MCT	A	201	1	−1
1MCT	A	202	0	−1
1MCT	A	203	0	−1
1MCT	A	204	0	−1
1MCT	A	205	0	−1
1MCT	A	206	0	−1
1MCT	A	207	0	−1
1MCT	A	208	1	−1
1MCT	A	209	0	−1
1MCT	A	210	0	−1
1MCT	A	211	1	−1

1MCT	A	212	0	−1
1MCT	A	213	0	−1
1MCT	A	214	1	−1
1MCT	A	215	0	−1
1MCT	A	216	0	−1
1MCT	A	217	1	−1
1MCT	A	218	1	−1
1MCT	A	219	0	−1
1MCT	A	220	0	−1
1MCT	A	221	1	−1
1MCT	A	222	1	−1
1MCT	A	223	1	−1
1NCA	L	1	1	−1
1NCA	L	2	0	−1
1NCA	L	3	1	−1
1NCA	L	4	0	−1
1NCA	L	5	1	−1
1NCA	L	6	0	−1
1NCA	L	7	1	−1
1NCA	L	8	1	−1
1NCA	L	9	1	−1
1NCA	L	10	1	−1
1NCA	L	11	0	−1
1NCA	L	12	1	−1
1NCA	L	13	0	−1
1NCA	L	14	1	−1
1NCA	L	15	1	−1
1NCA	L	16	1	−1
1NCA	L	17	1	−1
1NCA	L	18	1	−1
1NCA	L	19	0	−1
1NCA	L	20	1	−1
1NCA	L	21	0	−1
1NCA	L	22	1	−1
1NCA	L	23	0	−1
1NCA	L	24	1	−1
1NCA	L	25	0	−1
1NCA	L	26	1	−1
1NCA	L	27	1	−1
1NCA	L	28	1	−1
1NCA	L	29	0	−1
1NCA	L	30	1	−1
1NCA	L	31	1	1
1NCA	L	32	0	−1
1NCA	L	33	0	−1
1NCA	L	34	0	−1
1NCA	L	35	0	−1
1NCA	L	36	0	−1
1NCA	L	37	0	−1
1NCA	L	38	0	−1
1NCA	L	39	0	−1
1NCA	L	40	1	−1

1NCA	L	41	1	−1
1NCA	L	42	1	−1
1NCA	L	43	1	−1
1NCA	L	44	0	−1
1NCA	L	45	1	−1
1NCA	L	46	0	−1
1NCA	L	47	0	−1
1NCA	L	48	0	−1
1NCA	L	49	1	1
1NCA	L	50	1	1
1NCA	L	51	0	−1
1NCA	L	52	1	1
1NCA	L	53	1	1
1NCA	L	54	1	1
1NCA	L	55	0	−1
1NCA	L	56	1	1
1NCA	L	57	1	−1
1NCA	L	58	0	−1
1NCA	L	59	1	−1
1NCA	L	60	1	−1
1NCA	L	61	0	−1
1NCA	L	62	0	−1
1NCA	L	63	1	−1
1NCA	L	64	0	−1
1NCA	L	65	1	−1
1NCA	L	66	1	−1
1NCA	L	67	1	−1
1NCA	L	68	1	−1
1NCA	L	69	1	−1
1NCA	L	70	1	−1
1NCA	L	71	0	−1
1NCA	L	72	1	−1
1NCA	L	73	0	−1
1NCA	L	74	0	−1
1NCA	L	75	0	−1
1NCA	L	76	1	−1
1NCA	L	77	1	−1
1NCA	L	78	0	−1
1NCA	L	79	1	−1
1NCA	L	80	0	−1
1NCA	L	81	1	−1
1NCA	L	82	0	−1
1NCA	L	83	0	−1
1NCA	L	84	0	−1
1NCA	L	85	1	−1
1NCA	L	86	0	−1
1NCA	L	87	0	−1
1NCA	L	88	0	−1
1NCA	L	89	0	−1
1NCA	L	90	0	−1
1NCA	L	91	0	−1
1NCA	L	92	1	1

1NCA	L	93	1	1
1NCA	L	94	1	1
1NCA	L	95	1	−1
1NCA	L	96	0	−1
1NCA	L	97	0	−1
1NCA	L	98	0	−1
1NCA	L	99	0	−1
1NCA	L	100	1	−1
1NCA	L	101	0	−1
1NCA	L	102	0	−1
1NCA	L	103	0	−1
1NCA	L	104	0	−1
1NCA	L	105	0	−1
1NCA	L	106	0	−1
1NCA	L	107	1	−1
1NCA	L	108	1	−1
1NCA	L	109	1	−1
1NCA	L	110	1	−1
1NCA	L	111	0	−1
1NCA	L	112	1	−1
1NCA	L	113	0	−1
1NCA	L	114	1	−1
1NCA	L	115	0	−1
1NCA	L	116	0	−1
1NCA	L	117	0	−1
1NCA	L	118	0	−1
1NCA	L	119	0	−1
1NCA	L	120	0	−1
1NCA	L	121	0	−1
1NCA	L	122	1	−1
1NCA	L	123	1	−1
1NCA	L	124	0	−1
1NCA	L	125	0	−1
1NCA	L	126	1	−1
1NCA	L	127	1	−1
1NCA	L	128	1	−1
1NCA	L	129	0	−1
1NCA	L	130	0	−1
1NCA	L	131	0	−1
1NCA	L	132	0	−1
1NCA	L	133	0	−1
1NCA	L	134	0	−1
1NCA	L	135	0	−1
1NCA	L	136	0	−1
1NCA	L	137	0	−1
1NCA	L	138	1	−1
1NCA	L	139	0	−1
1NCA	L	140	0	−1
1NCA	L	141	0	−1
1NCA	L	142	1	−1
1NCA	L	143	1	−1
1NCA	L	144	0	−1

1NCA	L	145	1	−1
1NCA	L	146	0	−1
1NCA	L	147	0	−1
1NCA	L	148	0	−1
1NCA	L	149	1	−1
1NCA	L	150	0	−1
1NCA	L	151	1	−1
1NCA	L	152	1	−1
1NCA	L	153	1	−1
1NCA	L	154	1	−1
1NCA	L	155	0	−1
1NCA	L	156	1	−1
1NCA	L	157	1	−1
1NCA	L	158	1	−1
1NCA	L	159	1	−1
1NCA	L	160	0	−1
1NCA	L	161	1	−1
1NCA	L	162	0	−1
1NCA	L	163	1	−1
1NCA	L	164	0	−1
1NCA	L	165	1	−1
1NCA	L	166	0	−1
1NCA	L	167	1	−1
1NCA	L	168	1	−1
1NCA	L	169	1	−1
1NCA	L	170	1	−1
1NCA	L	171	0	−1
1NCA	L	172	0	−1
1NCA	L	173	0	−1
1NCA	L	174	0	−1
1NCA	L	175	0	−1
1NCA	L	176	0	−1
1NCA	L	177	0	−1
1NCA	L	178	0	−1
1NCA	L	179	0	−1
1NCA	L	180	1	−1
1NCA	L	181	0	−1
1NCA	L	182	1	−1
1NCA	L	183	1	−1
1NCA	L	184	1	−1
1NCA	L	185	0	−1
1NCA	L	186	0	−1
1NCA	L	187	1	−1
1NCA	L	188	1	−1
1NCA	L	189	1	−1
1NCA	L	190	1	−1
1NCA	L	191	1	−1
1NCA	L	192	0	−1
1NCA	L	193	0	−1
1NCA	L	194	0	−1
1NCA	L	195	0	−1
1NCA	L	196	0	−1

1NCA	L	197	1	−1
1NCA	L	198	0	−1
1NCA	L	199	1	−1
1NCA	L	200	1	−1
1NCA	L	201	1	−1
1NCA	L	202	1	−1
1NCA	L	203	1	−1
1NCA	L	204	1	−1
1NCA	L	205	0	−1
1NCA	L	206	1	−1
1NCA	L	207	1	−1
1NCA	L	208	1	−1
1NCA	L	209	0	−1
1NCA	L	210	1	−1
1NCA	L	211	1	−1
1NCA	L	212	1	−1
1NCA	L	213	1	−1
1NCA	L	214	1	−1
1NCA	H	1	1	−1
1NCA	H	2	0	−1
1NCA	H	3	1	−1
1NCA	H	4	0	−1
1NCA	H	5	1	−1
1NCA	H	6	0	−1
1NCA	H	7	1	−1
1NCA	H	8	1	−1
1NCA	H	9	1	−1
1NCA	H	10	1	−1
1NCA	H	11	1	−1
1NCA	H	12	0	−1
1NCA	H	13	1	−1
1NCA	H	14	1	−1
1NCA	H	15	1	−1
1NCA	H	16	1	−1
1NCA	H	17	1	−1
1NCA	H	18	0	−1
1NCA	H	19	1	−1
1NCA	H	20	0	−1
1NCA	H	21	1	−1
1NCA	H	22	0	−1
1NCA	H	23	1	−1
1NCA	H	24	0	−1
1NCA	H	25	1	−1
1NCA	H	26	1	−1
1NCA	H	27	0	−1
1NCA	H	28	1	−1
1NCA	H	29	0	−1
1NCA	H	30	1	1
1NCA	H	31	1	1
1NCA	H	32	1	1
1NCA	H	33	0	−1
1NCA	H	34	0	−1

1NCA	H	35	0	−1
1NCA	H	36	0	−1
1NCA	H	37	0	−1
1NCA	H	38	0	−1
1NCA	H	39	0	−1
1NCA	H	40	0	−1
1NCA	H	41	1	−1
1NCA	H	42	1	−1
1NCA	H	43	1	−1
1NCA	H	44	0	−1
1NCA	H	45	0	−1
1NCA	H	46	1	−1
1NCA	H	47	0	−1
1NCA	H	48	0	−1
1NCA	H	49	0	−1
1NCA	H	50	0	−1
1NCA	H	51	0	−1
1NCA	H	52	0	−1
1NCA	H	53	0	−1
1NCA	H	54	1	1
1NCA	H	55	1	1
1NCA	H	56	1	−1
1NCA	H	57	1	1
1NCA	H	58	1	−1
1NCA	H	59	1	−1
1NCA	H	60	0	−1
1NCA	H	61	0	−1
1NCA	H	62	1	1
1NCA	H	63	1	−1
1NCA	H	64	0	−1
1NCA	H	65	1	1
1NCA	H	66	1	−1
1NCA	H	67	0	−1
1NCA	H	68	0	−1
1NCA	H	69	1	−1
1NCA	H	70	0	−1
1NCA	H	71	1	−1
1NCA	H	72	0	−1
1NCA	H	73	1	−1
1NCA	H	74	1	−1
1NCA	H	75	1	−1
1NCA	H	76	1	−1
1NCA	H	77	1	−1
1NCA	H	78	0	−1
1NCA	H	79	0	−1
1NCA	H	80	0	−1
1NCA	H	81	0	−1
1NCA	H	82	0	−1
1NCA	H	83	0	−1
1NCA	H	84	1	−1
1NCA	H	85	1	−1
1NCA	H	86	0	−1

1NCA	H	87	1	−1
1NCA	H	88	1	−1
1NCA	H	89	1	−1
1NCA	H	90	0	−1
1NCA	H	91	1	−1
1NCA	H	92	0	−1
1NCA	H	93	0	−1
1NCA	H	94	0	−1
1NCA	H	95	0	−1
1NCA	H	96	0	−1
1NCA	H	97	0	−1
1NCA	H	98	0	−1
1NCA	H	99	0	−1
1NCA	H	100	1	1
1NCA	H	101	1	1
1NCA	H	102	1	1
1NCA	H	103	1	1
1NCA	H	104	0	−1
1NCA	H	105	0	−1
1NCA	H	106	0	−1
1NCA	H	107	0	−1
1NCA	H	108	0	−1
1NCA	H	109	1	1
1NCA	H	110	0	−1
1NCA	H	111	0	−1
1NCA	H	112	1	−1
1NCA	H	113	0	−1
1NCA	H	114	0	−1
1NCA	H	115	1	−1
1NCA	H	116	0	−1
1NCA	H	117	1	−1
1NCA	H	118	0	−1
1NCA	H	119	1	−1
1NCA	H	120	1	−1
1NCA	H	121	0	−1
1NCA	H	122	1	−1
1NCA	H	123	1	−1
1NCA	H	124	1	−1
1NCA	H	125	1	−1
1NCA	H	126	0	−1
1NCA	H	127	1	−1
1NCA	H	128	0	−1
1NCA	H	129	0	−1
1NCA	H	130	0	−1
1NCA	H	131	0	−1
1NCA	H	132	0	−1
1NCA	H	133	0	−1
1NCA	H	134	0	−1
1NCA	H	135	1	−1
1NCA	H	136	1	−1
1NCA	H	137	1	−1
1NCA	H	138	0	−1

1NCA	H	139	1	−1
1NCA	H	140	1	−1
1NCA	H	141	1	−1
1NCA	H	142	0	−1
1NCA	H	143	0	−1
1NCA	H	144	0	−1
1NCA	H	145	0	−1
1NCA	H	146	0	−1
1NCA	H	147	0	−1
1NCA	H	148	0	−1
1NCA	H	149	0	−1
1NCA	H	150	0	−1
1NCA	H	151	0	−1
1NCA	H	152	0	−1
1NCA	H	153	0	−1
1NCA	H	154	0	−1
1NCA	H	155	1	−1
1NCA	H	156	1	−1
1NCA	H	157	0	−1
1NCA	H	158	1	−1
1NCA	H	159	0	−1
1NCA	H	160	1	−1
1NCA	H	161	0	−1
1NCA	H	162	0	−1
1NCA	H	163	1	−1
1NCA	H	164	1	−1
1NCA	H	165	1	−1
1NCA	H	166	0	−1
1NCA	H	167	1	−1
1NCA	H	168	1	−1
1NCA	H	169	1	−1
1NCA	H	170	1	−1
1NCA	H	171	0	−1
1NCA	H	172	1	−1
1NCA	H	173	0	−1
1NCA	H	174	1	−1
1NCA	H	175	0	−1
1NCA	H	176	1	−1
1NCA	H	177	1	−1
1NCA	H	178	1	−1
1NCA	H	179	1	−1
1NCA	H	180	1	−1
1NCA	H	181	1	−1
1NCA	H	182	0	−1
1NCA	H	183	0	−1
1NCA	H	184	0	−1
1NCA	H	185	0	−1
1NCA	H	186	0	−1
1NCA	H	187	0	−1
1NCA	H	188	0	−1
1NCA	H	189	1	−1
1NCA	H	190	0	−1

1NCA	H	191	1	−1
1NCA	H	192	1	−1
1NCA	H	193	1	−1
1NCA	H	194	1	−1
1NCA	H	195	0	−1
1NCA	H	196	1	−1
1NCA	H	197	1	−1
1NCA	H	198	1	−1
1NCA	H	199	1	−1
1NCA	H	200	0	−1
1NCA	H	201	0	−1
1NCA	H	202	0	−1
1NCA	H	203	0	−1
1NCA	H	204	0	−1
1NCA	H	205	0	−1
1NCA	H	206	0	−1
1NCA	H	207	1	−1
1NCA	H	208	1	−1
1NCA	H	209	1	−1
1NCA	H	210	1	−1
1NCA	H	211	0	−1
1NCA	H	212	1	−1
1NCA	H	213	1	−1
1NCA	H	214	1	−1
1NCA	H	215	1	−1
1NCA	H	216	1	−1
1NCA	H	217	0	−1
1NCA	H	218	1	−1
1NCA	H	219	1	−1
1NCA	H	220	1	−1
1NCA	H	221	1	−1
1NCA	N	1	1	−1
1NCA	N	2	1	−1
1NCA	N	3	1	−1
1NCA	N	4	0	−1
1NCA	N	5	0	−1
1NCA	N	6	1	−1
1NCA	N	7	0	−1
1NCA	N	8	1	−1
1NCA	N	9	0	−1
1NCA	N	10	1	−1
1NCA	N	11	0	−1
1NCA	N	12	0	−1
1NCA	N	13	1	−1
1NCA	N	14	0	−1
1NCA	N	15	0	−1
1NCA	N	16	0	−1
1NCA	N	17	0	−1
1NCA	N	18	1	−1
1NCA	N	19	1	−1
1NCA	N	20	1	−1
1NCA	N	21	0	−1

1NCA	N	22	0	−1
1NCA	N	23	0	−1
1NCA	N	24	1	−1
1NCA	N	25	0	−1
1NCA	N	26	0	−1
1NCA	N	27	1	−1
1NCA	N	28	1	−1
1NCA	N	29	0	−1
1NCA	N	30	1	−1
1NCA	N	31	1	−1
1NCA	N	32	1	−1
1NCA	N	33	1	−1
1NCA	N	34	0	−1
1NCA	N	35	0	−1
1NCA	N	36	0	−1
1NCA	N	37	0	−1
1NCA	N	38	0	−1
1NCA	N	39	0	−1
1NCA	N	40	0	−1
1NCA	N	41	0	−1
1NCA	N	42	0	−1
1NCA	N	43	0	−1
1NCA	N	44	0	−1
1NCA	N	45	0	−1
1NCA	N	46	1	−1
1NCA	N	47	1	−1
1NCA	N	48	1	−1
1NCA	N	49	0	−1
1NCA	N	50	0	−1
1NCA	N	51	0	−1
1NCA	N	52	0	−1
1NCA	N	53	0	−1
1NCA	N	54	0	−1
1NCA	N	55	0	−1
1NCA	N	56	0	−1
1NCA	N	57	1	−1
1NCA	N	58	0	−1
1NCA	N	59	0	−1
1NCA	N	60	0	−1
1NCA	N	61	1	−1
1NCA	N	62	1	−1
1NCA	N	63	1	−1
1NCA	N	64	1	−1
1NCA	N	65	0	−1
1NCA	N	66	1	−1
1NCA	N	67	1	−1
1NCA	N	68	0	−1
1NCA	N	69	1	1
1NCA	N	70	1	−1
1NCA	N	71	1	−1
1NCA	N	72	1	−1
1NCA	N	73	0	−1

1NCA	N	74	1	−1
1NCA	N	75	1	−1
1NCA	N	76	0	−1
1NCA	N	77	0	−1
1NCA	N	78	0	−1
1NCA	N	79	0	−1
1NCA	N	80	0	−1
1NCA	N	81	0	−1
1NCA	N	82	1	−1
1NCA	N	83	1	−1
1NCA	N	84	1	−1
1NCA	N	85	1	−1
1NCA	N	86	0	−1
1NCA	N	87	0	−1
1NCA	N	88	0	−1
1NCA	N	89	1	−1
1NCA	N	90	1	−1
1NCA	N	91	1	−1
1NCA	N	92	0	−1
1NCA	N	93	1	−1
1NCA	N	94	1	−1
1NCA	N	95	0	−1
1NCA	N	96	0	−1
1NCA	N	97	1	−1
1NCA	N	98	0	−1
1NCA	N	99	0	−1
1NCA	N	100	0	−1
1NCA	N	101	0	−1
1NCA	N	102	0	−1
1NCA	N	103	0	−1
1NCA	N	104	0	−1
1NCA	N	105	0	−1
1NCA	N	106	0	−1
1NCA	N	107	0	−1
1NCA	N	108	1	−1
1NCA	N	109	0	−1
1NCA	N	110	0	−1
1NCA	N	111	0	−1
1NCA	N	112	0	−1
1NCA	N	113	0	−1
1NCA	N	114	0	−1
1NCA	N	115	0	−1
1NCA	N	116	1	−1
1NCA	N	117	1	−1
1NCA	N	118	1	−1
1NCA	N	119	1	−1
1NCA	N	120	1	−1
1NCA	N	121	1	−1
1NCA	N	122	0	−1
1NCA	N	123	1	−1
1NCA	N	124	0	−1
1NCA	N	125	1	−1

1NCA	N	126	0	−1
1NCA	N	127	1	−1
1NCA	N	128	0	−1
1NCA	N	129	1	−1
1NCA	N	130	1	−1
1NCA	N	131	1	−1
1NCA	N	132	1	−1
1NCA	N	133	1	−1
1NCA	N	134	0	−1
1NCA	N	135	1	−1
1NCA	N	136	0	−1
1NCA	N	137	1	−1
1NCA	N	138	0	−1
1NCA	N	139	0	−1
1NCA	N	140	1	−1
1NCA	N	141	1	−1
1NCA	N	142	1	−1
1NCA	N	143	0	−1
1NCA	N	144	0	−1
1NCA	N	145	0	−1
1NCA	N	146	0	−1
1NCA	N	147	0	−1
1NCA	N	148	0	−1
1NCA	N	149	0	−1
1NCA	N	150	0	−1
1NCA	N	151	0	−1
1NCA	N	152	0	−1
1NCA	N	153	0	−1
1NCA	N	154	0	−1
1NCA	N	155	1	−1
1NCA	N	156	0	−1
1NCA	N	157	0	−1
1NCA	N	158	0	−1
1NCA	N	159	0	−1
1NCA	N	160	0	−1
1NCA	N	161	0	−1
1NCA	N	162	0	−1
1NCA	N	163	0	−1
1NCA	N	164	0	−1
1NCA	N	165	0	−1
1NCA	N	166	1	−1
1NCA	N	167	1	−1
1NCA	N	168	1	−1
1NCA	N	169	0	−1
1NCA	N	170	1	−1
1NCA	N	171	1	−1
1NCA	N	172	1	−1
1NCA	N	173	0	−1
1NCA	N	174	0	−1
1NCA	N	175	0	−1
1NCA	N	176	0	−1
1NCA	N	177	0	−1

1NCA	N	178	0	−1
1NCA	N	179	1	−1
1NCA	N	180	1	−1
1NCA	N	181	0	−1
1NCA	N	182	1	−1
1NCA	N	183	1	−1
1NCA	N	184	1	−1
1NCA	N	185	1	−1
1NCA	N	186	1	−1
1NCA	N	187	0	−1
1NCA	N	188	1	−1
1NCA	N	189	1	−1
1NCA	N	190	1	−1
1NCA	N	191	1	−1
1NCA	N	192	1	−1
1NCA	N	193	0	−1
1NCA	N	194	1	−1
1NCA	N	195	0	−1
1NCA	N	196	0	−1
1NCA	N	197	0	−1
1NCA	N	198	0	−1
1NCA	N	199	0	−1
1NCA	N	200	0	−1
1NCA	N	201	0	−1
1NCA	N	202	0	−1
1NCA	N	203	0	−1
1NCA	N	204	0	−1
1NCA	N	205	1	−1
1NCA	N	206	1	−1
1NCA	N	207	1	−1
1NCA	N	208	0	−1
1NCA	N	209	0	−1
1NCA	N	210	0	−1
1NCA	N	211	0	−1
1NCA	N	212	0	−1
1NCA	N	213	0	−1
1NCA	N	214	0	−1
1NCA	N	215	0	−1
1NCA	N	216	1	−1
1NCA	N	217	1	−1
1NCA	N	218	0	−1
1NCA	N	219	0	−1
1NCA	N	220	0	−1
1NCA	N	221	0	−1
1NCA	N	222	0	−1
1NCA	N	223	0	−1
1NCA	N	224	0	−1
1NCA	N	225	1	−1
1NCA	N	226	0	−1
1NCA	N	227	1	−1
1NCA	N	228	0	−1
1NCA	N	229	1	−1

1NCA	N	230	1	−1
1NCA	N	231	1	−1
1NCA	N	232	1	−1
1NCA	N	233	0	−1
1NCA	N	234	1	−1
1NCA	N	235	0	−1
1NCA	N	236	1	−1
1NCA	N	237	0	−1
1NCA	N	238	0	−1
1NCA	N	239	0	−1
1NCA	N	240	0	−1
1NCA	N	241	0	−1
1NCA	N	242	0	−1
1NCA	N	243	0	−1
1NCA	N	244	0	−1
1NCA	N	245	0	−1
1NCA	N	246	0	−1
1NCA	N	247	0	−1
1NCA	N	248	0	−1
1NCA	N	249	0	−1
1NCA	N	250	1	1
1NCA	N	251	1	1
1NCA	N	252	0	−1
1NCA	N	253	1	−1
1NCA	N	254	1	−1
1NCA	N	255	0	−1
1NCA	N	256	1	−1
1NCA	N	257	0	−1
1NCA	N	258	1	−1
1NCA	N	259	1	−1
1NCA	N	260	0	−1
1NCA	N	261	0	−1
1NCA	N	262	1	−1
1NCA	N	263	1	1
1NCA	N	264	1	1
1NCA	N	265	1	1
1NCA	N	266	1	−1
1NCA	N	267	1	1
1NCA	N	268	0	−1
1NCA	N	269	0	−1
1NCA	N	270	0	−1
1NCA	N	271	0	−1
1NCA	N	272	0	−1
1NCA	N	273	0	−1
1NCA	N	274	0	−1
1NCA	N	275	0	−1
1NCA	N	276	0	−1
1NCA	N	277	1	−1
1NCA	N	278	1	−1
1NCA	N	279	0	−1
1NCA	N	280	0	−1
1NCA	N	281	0	−1

1NCA	N	282	0	−1
1NCA	N	283	0	−1
1NCA	N	284	0	−1
1NCA	N	285	0	−1
1NCA	N	286	1	1
1NCA	N	287	0	−1
1NCA	N	288	1	1
1NCA	N	289	1	1
1NCA	N	290	0	−1
1NCA	N	291	1	−1
1NCA	N	292	0	−1
1NCA	N	293	0	−1
1NCA	N	294	0	−1
1NCA	N	295	0	−1
1NCA	N	296	0	−1
1NCA	N	297	0	−1
1NCA	N	298	0	−1
1NCA	N	299	0	−1
1NCA	N	300	0	−1
1NCA	N	301	1	−1
1NCA	N	302	0	−1
1NCA	N	303	0	−1
1NCA	N	304	1	−1
1NCA	N	305	1	−1
1NCA	N	306	1	−1
1NCA	N	307	1	−1
1NCA	N	308	0	−1
1NCA	N	309	1	−1
1NCA	N	310	1	−1
1NCA	N	311	1	−1
1NCA	N	312	1	−1
1NCA	N	313	1	−1
1NCA	N	314	0	−1
1NCA	N	315	1	1
1NCA	N	316	0	−1
1NCA	N	317	0	−1
1NCA	N	318	1	1
1NCA	N	319	1	1
1NCA	N	320	1	1
1NCA	N	321	1	1
1NCA	N	322	1	1
1NCA	N	323	0	−1
1NCA	N	324	0	−1
1NCA	N	325	0	−1
1NCA	N	326	0	−1
1NCA	N	327	0	−1
1NCA	N	328	0	−1
1NCA	N	329	0	−1
1NCA	N	330	0	−1
1NCA	N	331	1	−1
1NCA	N	332	0	−1
1NCA	N	333	0	−1

1NCA	N	334	1	−1
1NCA	N	335	1	−1
1NCA	N	336	1	−1
1NCA	N	337	1	−1
1NCA	N	338	1	−1
1NCA	N	339	0	−1
1NCA	N	340	1	−1
1NCA	N	341	0	−1
1NCA	N	342	0	−1
1NCA	N	343	0	−1
1NCA	N	344	0	−1
1NCA	N	345	0	−1
1NCA	N	346	0	−1
1NCA	N	347	0	−1
1NCA	N	348	0	−1
1NCA	N	349	0	−1
1NCA	N	350	0	−1
1NCA	N	351	1	−1
1NCA	N	352	1	1
1NCA	N	353	1	1
1NCA	N	354	0	−1
1NCA	N	355	1	1
1NCA	N	356	1	1
1NCA	N	357	0	−1
1NCA	N	358	1	−1
1NCA	N	359	0	−1
1NCA	N	360	0	−1
1NCA	N	361	0	−1
1NCA	N	362	0	−1
1NCA	N	363	0	−1
1NCA	N	364	0	−1
1NCA	N	365	0	−1
1NCA	N	366	0	−1
1NCA	N	367	0	−1
1NCA	N	368	0	−1
1NCA	N	369	0	−1
1NCA	N	370	0	−1
1NCA	N	371	1	−1
1NCA	N	372	1	−1
1NCA	N	373	1	−1
1NCA	N	374	1	−1
1NCA	N	375	1	−1
1NCA	N	376	1	−1
1NCA	N	377	1	−1
1NCA	N	378	1	1
1NCA	N	379	0	−1
1NCA	N	380	1	−1
1NCA	N	381	0	−1
1NCA	N	382	1	−1
1NCA	N	383	0	−1
1NCA	N	384	1	1
1NCA	N	385	0	−1

1NCA	N	386	1	1
1NCA	N	387	1	−1
1NCA	N	388	0	−1
1NCA	N	389	1	−1
1NFD	D	1	1	−1
1NFD	D	2	1	−1
1NFD	D	3	0	−1
1NFD	D	4	1	−1
1NFD	D	5	0	−1
1NFD	D	6	1	−1
1NFD	D	7	1	−1
1NFD	D	8	1	−1
1NFD	D	9	1	−1
1NFD	D	10	1	−1
1NFD	D	11	1	−1
1NFD	D	12	0	−1
1NFD	D	13	1	−1
1NFD	D	14	0	−1
1NFD	D	15	1	−1
1NFD	D	16	1	−1
1NFD	D	17	1	−1
1NFD	D	18	0	−1
1NFD	D	19	1	−1
1NFD	D	20	0	−1
1NFD	D	21	1	−1
1NFD	D	22	0	−1
1NFD	D	23	1	−1
1NFD	D	24	0	−1
1NFD	D	25	1	−1
1NFD	D	26	1	−1
1NFD	D	27	1	−1
1NFD	D	28	1	−1
1NFD	D	29	1	−1
1NFD	D	30	0	−1
1NFD	D	31	1	−1
1NFD	D	32	0	−1
1NFD	D	33	0	−1
1NFD	D	34	0	−1
1NFD	D	35	0	−1
1NFD	D	36	0	−1
1NFD	D	37	0	−1
1NFD	D	38	0	−1
1NFD	D	39	0	−1
1NFD	D	40	1	−1
1NFD	D	41	1	−1
1NFD	D	42	1	−1
1NFD	D	43	0	−1
1NFD	D	44	0	−1
1NFD	D	45	0	−1
1NFD	D	46	0	−1
1NFD	D	47	0	−1
1NFD	D	48	0	−1

1NFD	D	49	0	−1
1NFD	D	50	0	−1
1NFD	D	51	1	−1
1NFD	D	52	1	−1
1NFD	D	53	1	−1
1NFD	D	54	1	−1
1NFD	D	55	1	−1
1NFD	D	56	0	−1
1NFD	D	57	1	−1
1NFD	D	58	0	−1
1NFD	D	59	1	−1
1NFD	D	60	0	−1
1NFD	D	61	1	−1
1NFD	D	62	0	−1
1NFD	D	63	0	−1
1NFD	D	64	0	−1
1NFD	D	65	0	−1
1NFD	D	66	0	−1
1NFD	D	67	1	−1
1NFD	D	68	1	−1
1NFD	D	69	1	−1
1NFD	D	70	1	−1
1NFD	D	71	1	−1
1NFD	D	72	0	−1
1NFD	D	73	0	−1
1NFD	D	74	0	−1
1NFD	D	75	0	−1
1NFD	D	76	1	−1
1NFD	D	77	0	−1
1NFD	D	78	1	−1
1NFD	D	79	1	−1
1NFD	D	80	0	−1
1NFD	D	81	1	−1
1NFD	D	82	1	−1
1NFD	D	83	1	−1
1NFD	D	84	0	−1
1NFD	D	85	1	−1
1NFD	D	86	0	−1
1NFD	D	87	0	−1
1NFD	D	88	0	−1
1NFD	D	89	0	−1
1NFD	D	90	0	−1
1NFD	D	91	0	−1
1NFD	D	92	0	−1
1NFD	D	93	0	−1
1NFD	D	94	0	−1
1NFD	D	95	0	−1
1NFD	D	96	1	−1
1NFD	D	97	1	−1
1NFD	D	98	1	−1
1NFD	D	99	1	−1
1NFD	D	100	0	−1

1NFD	D	101	0	−1
1NFD	D	102	0	−1
1NFD	D	103	0	−1
1NFD	D	104	1	−1
1NFD	D	105	0	−1
1NFD	D	106	0	−1
1NFD	D	107	1	−1
1NFD	D	108	0	−1
1NFD	D	109	1	−1
1NFD	D	110	0	−1
1NFD	D	111	0	−1
1NFD	D	112	0	−1
1NFD	D	113	1	−1
1NFD	D	114	0	−1
1NFD	D	115	1	−1
1NFD	D	116	1	−1
1NFD	D	117	1	−1
1NFD	D	118	1	−1
1NFD	D	119	0	−1
1NFD	D	120	0	−1
1NFD	D	121	0	−1
1NFD	D	122	0	−1
1NFD	D	123	1	−1
1NFD	D	124	0	−1
1NFD	D	125	1	−1
1NFD	D	126	0	−1
1NFD	D	127	1	−1
1NFD	D	128	1	−1
1NFD	D	129	1	−1
1NFD	D	130	1	−1
1NFD	D	131	1	−1
1NFD	D	132	0	−1
1NFD	D	133	0	−1
1NFD	D	134	0	−1
1NFD	D	135	0	−1
1NFD	D	136	0	−1
1NFD	D	137	0	−1
1NFD	D	138	0	−1
1NFD	D	139	0	−1
1NFD	D	140	0	−1
1NFD	D	141	1	−1
1NFD	D	142	0	−1
1NFD	D	143	1	−1
1NFD	D	144	1	−1
1NFD	D	145	1	−1
1NFD	D	146	0	−1
1NFD	D	147	0	−1
1NFD	D	148	1	−1
1NFD	D	149	1	−1
1NFD	D	150	1	−1
1NFD	D	151	1	−1
1NFD	D	152	1	−1

1NFD	D	153	1	−1
1NFD	D	154	0	−1
1NFD	D	155	0	−1
1NFD	D	156	0	−1
1NFD	D	157	0	−1
1NFD	D	158	1	−1
1NFD	D	159	1	−1
1NFD	D	160	0	−1
1NFD	D	161	1	−1
1NFD	D	162	0	−1
1NFD	D	163	1	−1
1NFD	D	164	0	−1
1NFD	D	165	1	−1
1NFD	D	166	1	−1
1NFD	D	167	1	−1
1NFD	D	168	1	−1
1NFD	D	169	1	−1
1NFD	D	170	0	−1
1NFD	D	171	0	−1
1NFD	D	172	0	−1
1NFD	D	173	0	−1
1NFD	D	174	0	−1
1NFD	D	175	0	−1
1NFD	D	176	0	−1
1NFD	D	177	0	−1
1NFD	D	178	0	−1
1NFD	D	179	1	−1
1NFD	D	180	1	−1
1NFD	D	181	1	−1
1NFD	D	182	1	−1
1NFD	D	183	1	−1
1NFD	D	184	1	−1
1NFD	D	185	0	−1
1NFD	D	186	1	−1
1NFD	D	187	0	−1
1NFD	D	188	0	−1
1NFD	D	189	0	−1
1NFD	D	190	1	−1
1NFD	D	191	1	−1
1NFD	D	192	0	−1
1NFD	D	193	1	−1
1NFD	D	194	1	−1
1NFD	D	195	1	−1
1NFD	D	196	1	−1
1NFD	D	197	1	−1
1NFD	D	198	1	−1
1NFD	D	199	1	−1
1NFD	D	200	1	−1
1NFD	D	201	1	−1
1NFD	D	202	1	−1
1NFD	D	203	1	−1
1NFD	D	1	1	−1

1NFD	D	2	1	−1
1NFD	D	3	0	−1
1NFD	D	4	0	−1
1NFD	D	5	1	−1
1NFD	D	6	0	−1
1NFD	D	7	1	−1
1NFD	D	8	1	−1
1NFD	D	9	1	−1
1NFD	D	10	1	−1
1NFD	D	11	0	−1
1NFD	D	12	0	−1
1NFD	D	13	0	−1
1NFD	D	14	1	1
1NFD	D	15	1	1
1NFD	D	16	1	−1
1NFD	D	17	1	−1
1NFD	D	18	1	1
1NFD	D	19	0	−1
1NFD	D	20	1	−1
1NFD	D	21	0	−1
1NFD	D	22	0	−1
1NFD	D	23	0	−1
1NFD	D	24	1	−1
1NFD	D	25	0	−1
1NFD	D	26	0	−1
1NFD	D	27	0	−1
1NFD	D	28	0	−1
1NFD	D	29	0	−1
1NFD	D	30	0	−1
1NFD	D	31	0	−1
1NFD	D	32	0	−1
1NFD	D	33	0	−1
1NFD	D	34	0	−1
1NFD	D	35	0	−1
1NFD	D	36	0	−1
1NFD	D	37	0	−1
1NFD	D	38	1	−1
1NFD	D	39	1	−1
1NFD	D	40	1	−1
1NFD	D	41	1	−1
1NFD	D	42	1	−1
1NFD	D	43	0	−1
1NFD	D	44	1	−1
1NFD	D	45	0	−1
1NFD	D	46	0	−1
1NFD	D	47	0	−1
1NFD	D	48	0	−1
1NFD	D	49	0	−1
1NFD	D	50	0	−1
1NFD	D	51	1	−1
1NFD	D	52	1	−1
1NFD	D	53	1	−1

1NFD	D	54	1	−1
1NFD	D	55	1	−1
1NFD	D	56	0	−1
1NFD	D	57	1	−1
1NFD	D	58	1	−1
1NFD	D	59	1	−1
1NFD	D	60	0	−1
1NFD	D	61	0	−1
1NFD	D	62	1	−1
1NFD	D	63	1	−1
1NFD	D	64	0	−1
1NFD	D	65	1	−1
1NFD	D	66	0	−1
1NFD	D	67	1	−1
1NFD	D	68	0	−1
1NFD	D	69	1	−1
1NFD	D	70	1	−1
1NFD	D	71	1	−1
1NFD	D	72	0	−1
1NFD	D	73	0	−1
1NFD	D	74	0	−1
1NFD	D	75	1	−1
1NFD	D	76	0	−1
1NFD	D	77	1	−1
1NFD	D	78	0	−1
1NFD	D	79	0	−1
1NFD	D	80	1	−1
1NFD	D	81	0	−1
1NFD	D	82	1	−1
1NFD	D	83	1	−1
1NFD	D	84	1	−1
1NFD	D	85	0	−1
1NFD	D	86	1	−1
1NFD	D	87	0	−1
1NFD	D	88	1	−1
1NFD	D	89	0	−1
1NFD	D	90	0	−1
1NFD	D	91	0	−1
1NFD	D	92	0	−1
1NFD	D	93	0	−1
1NFD	D	94	0	−1
1NFD	D	95	0	−1
1NFD	D	96	0	−1
1NFD	D	97	1	−1
1NFD	D	98	0	−1
1NFD	D	99	0	−1
1NFD	D	100	0	−1
1NFD	D	101	0	−1
1NFD	D	102	0	−1
1NFD	D	103	0	−1
1NFD	D	104	0	−1
1NFD	D	105	0	−1

1NFD	D	106	0	−1
1NFD	D	107	0	−1
1NFD	D	108	1	−1
1NFD	D	109	0	−1
1NFD	D	110	0	−1
1NFD	D	111	0	−1
1NFD	D	112	0	−1
1NFD	D	113	1	−1
1NFD	D	114	1	1
1NFD	D	115	0	−1
1NFD	D	116	1	1
1NFD	D	117	0	−1
1NFD	D	118	0	−1
1NFD	D	119	1	1
1NFD	D	120	1	−1
1NFD	D	121	0	−1
1NFD	D	122	1	−1
1NFD	D	123	0	−1
1NFD	D	124	0	−1
1NFD	D	125	0	−1
1NFD	D	126	0	−1
1NFD	D	127	0	−1
1NFD	D	128	0	−1
1NFD	D	129	0	−1
1NFD	D	130	1	−1
1NFD	D	131	0	−1
1NFD	D	132	0	−1
1NFD	D	133	0	−1
1NFD	D	134	1	−1
1NFD	D	135	1	−1
1NFD	D	136	0	−1
1NFD	D	137	1	−1
1NFD	D	138	0	−1
1NFD	D	139	0	−1
1NFD	D	140	0	−1
1NFD	D	141	0	−1
1NFD	D	142	0	−1
1NFD	D	143	0	−1
1NFD	D	144	0	−1
1NFD	D	145	0	−1
1NFD	D	146	1	−1
1NFD	D	147	1	−1
1NFD	D	148	0	−1
1NFD	D	149	0	−1
1NFD	D	150	0	−1
1NFD	D	151	0	−1
1NFD	D	152	0	−1
1NFD	D	153	0	−1
1NFD	D	154	1	−1
1NFD	D	155	0	−1
1NFD	D	156	0	−1
1NFD	D	157	0	−1

1NFD	D	158	1	−1
1NFD	D	159	0	−1
1NFD	D	160	1	−1
1NFD	D	161	1	−1
1NFD	D	162	1	−1
1NFD	D	163	1	−1
1NFD	D	164	0	−1
1NFD	D	165	1	−1
1NFD	D	166	0	−1
1NFD	D	167	0	−1
1NFD	D	168	0	−1
1NFD	D	169	0	−1
1NFD	D	170	1	−1
1NFD	D	171	0	−1
1NFD	D	172	1	−1
1NFD	D	173	1	−1
1NFD	D	174	0	−1
1NFD	D	175	0	−1
1NFD	D	176	1	−1
1NFD	D	177	0	−1
1NFD	D	178	1	−1
1NFD	D	179	1	−1
1NFD	D	180	1	−1
1NFD	D	181	0	−1
1NFD	D	182	0	−1
1NFD	D	183	0	−1
1NFD	D	184	0	−1
1NFD	D	185	0	−1
1NFD	D	186	0	−1
1NFD	D	187	0	−1
1NFD	D	188	0	−1
1NFD	D	189	0	−1
1NFD	D	190	0	−1
1NFD	D	191	1	−1
1NFD	D	192	0	−1
1NFD	D	193	1	−1
1NFD	D	194	1	−1
1NFD	D	195	0	−1
1NFD	D	196	1	−1
1NFD	D	197	1	−1
1NFD	D	198	1	−1
1NFD	D	199	1	−1
1NFD	D	200	0	−1
1NFD	D	201	0	−1
1NFD	D	202	0	−1
1NFD	D	203	1	−1
1NFD	D	204	0	−1
1NFD	D	205	0	−1
1NFD	D	206	0	−1
1NFD	D	207	1	−1
1NFD	D	208	0	−1
1NFD	D	209	0	−1

1NFD	D	210	0	−1
1NFD	D	211	0	−1
1NFD	D	212	1	−1
1NFD	D	213	1	1
1NFD	D	214	1	1
1NFD	D	215	1	1
1NFD	D	216	1	1
1NFD	D	217	0	−1
1NFD	D	218	1	1
1NFD	D	219	1	1
1NFD	D	220	1	1
1NFD	D	221	1	1
1NFD	D	222	1	1
1NFD	D	223	1	1
1NFD	D	224	0	−1
1NFD	D	225	1	−1
1NFD	D	226	1	−1
1NFD	D	227	1	−1
1NFD	D	228	1	−1
1NFD	D	229	0	−1
1NFD	D	230	1	−1
1NFD	D	231	0	−1
1NFD	D	232	1	−1
1NFD	D	233	0	−1
1NFD	D	234	1	−1
1NFD	D	235	0	−1
1NFD	D	236	1	−1
1NFD	D	237	1	−1
1NFD	D	238	1	−1
1NFD	D	239	1	−1
1NFD	D	1	1	−1
1NFD	D	2	1	−1
1NFD	D	3	0	−1
1NFD	D	4	1	−1
1NFD	D	5	0	−1
1NFD	D	6	1	−1
1NFD	D	7	1	−1
1NFD	D	8	1	−1
1NFD	D	9	1	−1
1NFD	D	10	1	−1
1NFD	D	11	1	−1
1NFD	D	12	0	−1
1NFD	D	13	1	−1
1NFD	D	14	0	−1
1NFD	D	15	1	−1
1NFD	D	16	1	−1
1NFD	D	17	1	−1
1NFD	D	18	0	−1
1NFD	D	19	1	−1
1NFD	D	20	0	−1
1NFD	D	21	1	−1
1NFD	D	22	0	−1

1NFD	D	23	1	−1
1NFD	D	24	0	−1
1NFD	D	25	1	−1
1NFD	D	26	1	−1
1NFD	D	27	1	−1
1NFD	D	28	1	−1
1NFD	D	29	1	−1
1NFD	D	30	0	−1
1NFD	D	31	1	−1
1NFD	D	32	0	−1
1NFD	D	33	0	−1
1NFD	D	34	0	−1
1NFD	D	35	0	−1
1NFD	D	36	0	−1
1NFD	D	37	0	−1
1NFD	D	38	0	−1
1NFD	D	39	1	−1
1NFD	D	40	1	−1
1NFD	D	41	1	−1
1NFD	D	42	1	−1
1NFD	D	43	0	−1
1NFD	D	44	0	−1
1NFD	D	45	0	−1
1NFD	D	46	0	−1
1NFD	D	47	0	−1
1NFD	D	48	0	−1
1NFD	D	49	0	−1
1NFD	D	50	0	−1
1NFD	D	51	1	−1
1NFD	D	52	1	−1
1NFD	D	53	1	−1
1NFD	D	54	1	−1
1NFD	D	55	1	−1
1NFD	D	56	1	−1
1NFD	D	57	0	−1
1NFD	D	58	1	−1
1NFD	D	59	0	−1
1NFD	D	60	1	−1
1NFD	D	61	1	−1
1NFD	D	62	0	−1
1NFD	D	63	0	−1
1NFD	D	64	0	−1
1NFD	D	65	0	−1
1NFD	D	66	0	−1
1NFD	D	67	1	−1
1NFD	D	68	1	−1
1NFD	D	69	1	−1
1NFD	D	70	1	−1
1NFD	D	71	1	−1
1NFD	D	72	0	−1
1NFD	D	73	0	−1
1NFD	D	74	0	−1

1NFD	D	75	0	−1
1NFD	D	76	1	−1
1NFD	D	77	0	−1
1NFD	D	78	1	−1
1NFD	D	79	1	−1
1NFD	D	80	0	−1
1NFD	D	81	1	−1
1NFD	D	82	1	−1
1NFD	D	83	1	−1
1NFD	D	84	0	−1
1NFD	D	85	1	−1
1NFD	D	86	0	−1
1NFD	D	87	0	−1
1NFD	D	88	0	−1
1NFD	D	89	0	−1
1NFD	D	90	0	−1
1NFD	D	91	0	−1
1NFD	D	92	0	−1
1NFD	D	93	0	−1
1NFD	D	94	0	−1
1NFD	D	95	0	−1
1NFD	D	96	1	−1
1NFD	D	97	1	−1
1NFD	D	98	1	−1
1NFD	D	99	1	−1
1NFD	D	100	0	−1
1NFD	D	101	0	−1
1NFD	D	102	0	−1
1NFD	D	103	0	−1
1NFD	D	104	1	−1
1NFD	D	105	0	−1
1NFD	D	106	0	−1
1NFD	D	107	1	−1
1NFD	D	108	0	−1
1NFD	D	109	1	−1
1NFD	D	110	0	−1
1NFD	D	111	0	−1
1NFD	D	112	0	−1
1NFD	D	113	1	−1
1NFD	D	114	0	−1
1NFD	D	115	1	−1
1NFD	D	116	1	−1
1NFD	D	117	1	−1
1NFD	D	118	1	−1
1NFD	D	119	0	−1
1NFD	D	120	0	−1
1NFD	D	121	0	−1
1NFD	D	122	0	−1
1NFD	D	123	1	−1
1NFD	D	124	0	−1
1NFD	D	125	1	−1
1NFD	D	126	0	−1

1NFD	D	127	1	−1
1NFD	D	128	1	−1
1NFD	D	129	1	−1
1NFD	D	130	1	−1
1NFD	D	131	1	−1
1NFD	D	132	0	−1
1NFD	D	133	0	−1
1NFD	D	134	0	−1
1NFD	D	135	0	−1
1NFD	D	136	0	−1
1NFD	D	137	0	−1
1NFD	D	138	0	−1
1NFD	D	139	0	−1
1NFD	D	140	0	−1
1NFD	D	141	1	−1
1NFD	D	142	0	−1
1NFD	D	143	1	−1
1NFD	D	144	1	−1
1NFD	D	145	1	−1
1NFD	D	146	0	−1
1NFD	D	147	0	−1
1NFD	D	148	1	−1
1NFD	D	149	1	−1
1NFD	D	150	1	−1
1NFD	D	151	1	−1
1NFD	D	152	1	−1
1NFD	D	153	1	−1
1NFD	D	154	0	−1
1NFD	D	155	1	−1
1NFD	D	156	0	−1
1NFD	D	157	0	−1
1NFD	D	158	1	−1
1NFD	D	159	1	−1
1NFD	D	160	0	−1
1NFD	D	161	1	−1
1NFD	D	162	0	−1
1NFD	D	163	1	−1
1NFD	D	164	0	−1
1NFD	D	165	1	−1
1NFD	D	166	1	−1
1NFD	D	167	1	−1
1NFD	D	168	1	−1
1NFD	D	169	1	−1
1NFD	D	170	0	−1
1NFD	D	171	0	−1
1NFD	D	172	0	−1
1NFD	D	173	0	−1
1NFD	D	174	0	−1
1NFD	D	175	0	−1
1NFD	D	176	0	−1
1NFD	D	177	0	−1
1NFD	D	178	0	−1

1NFD	D	179	1	−1
1NFD	D	180	1	−1
1NFD	D	181	1	−1
1NFD	D	182	1	−1
1NFD	D	183	1	−1
1NFD	D	184	1	−1
1NFD	D	185	0	−1
1NFD	D	186	1	−1
1NFD	D	187	0	−1
1NFD	D	188	0	−1
1NFD	D	189	0	−1
1NFD	D	190	1	−1
1NFD	D	191	1	−1
1NFD	D	192	1	−1
1NFD	D	193	1	−1
1NFD	D	194	1	−1
1NFD	D	195	1	−1
1NFD	D	196	1	−1
1NFD	D	197	0	−1
1NFD	D	198	1	−1
1NFD	D	199	1	−1
1NFD	D	200	1	−1
1NFD	D	201	1	−1
1NFD	D	202	1	−1
1NFD	D	203	1	−1
1NFD	D	1	1	−1
1NFD	D	2	0	−1
1NFD	D	3	0	−1
1NFD	D	4	0	−1
1NFD	D	5	1	−1
1NFD	D	6	0	−1
1NFD	D	7	1	−1
1NFD	D	8	1	−1
1NFD	D	9	1	−1
1NFD	D	10	1	−1
1NFD	D	11	0	−1
1NFD	D	12	0	−1
1NFD	D	13	0	−1
1NFD	D	14	1	1
1NFD	D	15	1	−1
1NFD	D	16	1	−1
1NFD	D	17	1	−1
1NFD	D	18	1	−1
1NFD	D	19	0	−1
1NFD	D	20	1	−1
1NFD	D	21	0	−1
1NFD	D	22	0	−1
1NFD	D	23	0	−1
1NFD	D	24	1	−1
1NFD	D	25	0	−1
1NFD	D	26	0	−1
1NFD	D	27	0	−1

1NFD	D	28	0	−1
1NFD	D	29	0	−1
1NFD	D	30	0	−1
1NFD	D	31	0	−1
1NFD	D	32	0	−1
1NFD	D	33	0	−1
1NFD	D	34	0	−1
1NFD	D	35	0	−1
1NFD	D	36	0	−1
1NFD	D	37	0	−1
1NFD	D	38	1	−1
1NFD	D	39	1	−1
1NFD	D	40	1	−1
1NFD	D	41	0	−1
1NFD	D	42	1	−1
1NFD	D	43	0	−1
1NFD	D	44	1	−1
1NFD	D	45	0	−1
1NFD	D	46	0	−1
1NFD	D	47	0	−1
1NFD	D	48	0	−1
1NFD	D	49	0	−1
1NFD	D	50	1	−1
1NFD	D	51	0	−1
1NFD	D	52	1	−1
1NFD	D	53	1	−1
1NFD	D	54	1	−1
1NFD	D	55	1	−1
1NFD	D	56	1	−1
1NFD	D	57	1	−1
1NFD	D	58	1	−1
1NFD	D	59	1	−1
1NFD	D	60	0	−1
1NFD	D	61	0	−1
1NFD	D	62	1	−1
1NFD	D	63	1	−1
1NFD	D	64	0	−1
1NFD	D	65	1	−1
1NFD	D	66	0	−1
1NFD	D	67	1	−1
1NFD	D	68	0	−1
1NFD	D	69	1	−1
1NFD	D	70	1	−1
1NFD	D	71	1	−1
1NFD	D	72	0	−1
1NFD	D	73	0	−1
1NFD	D	74	0	−1
1NFD	D	75	1	−1
1NFD	D	76	0	−1
1NFD	D	77	1	−1
1NFD	D	78	0	−1
1NFD	D	79	0	−1

1NFD	D	80	1	−1
1NFD	D	81	0	−1
1NFD	D	82	1	−1
1NFD	D	83	1	−1
1NFD	D	84	1	−1
1NFD	D	85	0	−1
1NFD	D	86	1	−1
1NFD	D	87	0	−1
1NFD	D	88	1	−1
1NFD	D	89	0	−1
1NFD	D	90	0	−1
1NFD	D	91	0	−1
1NFD	D	92	0	−1
1NFD	D	93	0	−1
1NFD	D	94	0	−1
1NFD	D	95	0	−1
1NFD	D	96	0	−1
1NFD	D	97	1	−1
1NFD	D	98	0	−1
1NFD	D	99	0	−1
1NFD	D	100	0	−1
1NFD	D	101	0	−1
1NFD	D	102	0	−1
1NFD	D	103	0	−1
1NFD	D	104	0	−1
1NFD	D	105	0	−1
1NFD	D	106	0	−1
1NFD	D	107	0	−1
1NFD	D	108	1	−1
1NFD	D	109	0	−1
1NFD	D	110	0	−1
1NFD	D	111	0	−1
1NFD	D	112	0	−1
1NFD	D	113	1	1
1NFD	D	114	1	1
1NFD	D	115	0	−1
1NFD	D	116	1	1
1NFD	D	117	1	1
1NFD	D	118	0	−1
1NFD	D	119	1	1
1NFD	D	120	1	−1
1NFD	D	121	0	−1
1NFD	D	122	1	−1
1NFD	D	123	0	−1
1NFD	D	124	0	−1
1NFD	D	125	0	−1
1NFD	D	126	0	−1
1NFD	D	127	1	−1
1NFD	D	128	0	−1
1NFD	D	129	0	−1
1NFD	D	130	1	−1
1NFD	D	131	0	−1

1NFD	D	132	0	−1
1NFD	D	133	0	−1
1NFD	D	134	1	−1
1NFD	D	135	1	−1
1NFD	D	136	0	−1
1NFD	D	137	1	−1
1NFD	D	138	1	−1
1NFD	D	139	0	−1
1NFD	D	140	0	−1
1NFD	D	141	0	−1
1NFD	D	142	0	−1
1NFD	D	143	0	−1
1NFD	D	144	0	−1
1NFD	D	145	0	−1
1NFD	D	146	1	−1
1NFD	D	147	1	−1
1NFD	D	148	0	−1
1NFD	D	149	0	−1
1NFD	D	150	0	−1
1NFD	D	151	0	−1
1NFD	D	152	0	−1
1NFD	D	153	0	−1
1NFD	D	154	1	−1
1NFD	D	155	0	−1
1NFD	D	156	0	−1
1NFD	D	157	0	−1
1NFD	D	158	1	−1
1NFD	D	159	0	−1
1NFD	D	160	1	−1
1NFD	D	161	1	−1
1NFD	D	162	1	−1
1NFD	D	163	1	−1
1NFD	D	164	0	−1
1NFD	D	165	1	−1
1NFD	D	166	1	−1
1NFD	D	167	0	−1
1NFD	D	168	0	−1
1NFD	D	169	0	−1
1NFD	D	170	1	−1
1NFD	D	171	0	−1
1NFD	D	172	1	−1
1NFD	D	173	1	−1
1NFD	D	174	0	−1
1NFD	D	175	0	−1
1NFD	D	176	1	−1
1NFD	D	177	0	−1
1NFD	D	178	1	−1
1NFD	D	179	1	−1
1NFD	D	180	1	−1
1NFD	D	181	0	−1
1NFD	D	182	0	−1
1NFD	D	183	0	−1

1NFD	D	184	0	−1
1NFD	D	185	0	−1
1NFD	D	186	0	−1
1NFD	D	187	0	−1
1NFD	D	188	0	−1
1NFD	D	189	0	−1
1NFD	D	190	0	−1
1NFD	D	191	0	−1
1NFD	D	192	0	−1
1NFD	D	193	1	−1
1NFD	D	194	1	−1
1NFD	D	195	0	−1
1NFD	D	196	1	−1
1NFD	D	197	1	−1
1NFD	D	198	1	−1
1NFD	D	199	1	−1
1NFD	D	200	0	−1
1NFD	D	201	0	−1
1NFD	D	202	0	−1
1NFD	D	203	1	−1
1NFD	D	204	0	−1
1NFD	D	205	0	−1
1NFD	D	206	0	−1
1NFD	D	207	1	−1
1NFD	D	208	0	−1
1NFD	D	209	0	−1
1NFD	D	210	0	−1
1NFD	D	211	0	−1
1NFD	D	212	1	−1
1NFD	D	213	1	1
1NFD	D	214	1	1
1NFD	D	215	1	1
1NFD	D	216	1	1
1NFD	D	217	0	−1
1NFD	D	218	1	1
1NFD	D	219	1	1
1NFD	D	220	1	1
1NFD	D	221	1	1
1NFD	D	222	1	1
1NFD	D	223	1	1
1NFD	D	224	0	−1
1NFD	D	225	1	−1
1NFD	D	226	1	−1
1NFD	D	227	1	−1
1NFD	D	228	1	−1
1NFD	D	229	1	−1
1NFD	D	230	1	−1
1NFD	D	231	0	−1
1NFD	D	232	1	−1
1NFD	D	233	0	−1
1NFD	D	234	1	−1
1NFD	D	235	0	−1

1NFD	D	236	1	−1
1NFD	D	237	1	−1
1NFD	D	238	1	−1
1NFD	D	239	1	−1
1NFD	H	1	1	−1
1NFD	H	2	1	−1
1NFD	H	3	0	−1
1NFD	H	4	1	−1
1NFD	H	5	0	−1
1NFD	H	6	1	−1
1NFD	H	7	1	−1
1NFD	H	8	1	−1
1NFD	H	9	0	−1
1NFD	H	10	1	−1
1NFD	H	11	0	−1
1NFD	H	12	1	−1
1NFD	H	13	1	−1
1NFD	H	14	1	−1
1NFD	H	15	1	−1
1NFD	H	16	1	−1
1NFD	H	17	0	−1
1NFD	H	18	1	−1
1NFD	H	19	0	−1
1NFD	H	20	1	−1
1NFD	H	21	0	−1
1NFD	H	22	1	−1
1NFD	H	23	0	−1
1NFD	H	24	1	−1
1NFD	H	25	1	−1
1NFD	H	26	0	−1
1NFD	H	27	1	1
1NFD	H	28	1	1
1NFD	H	29	0	−1
1NFD	H	30	1	1
1NFD	H	31	0	−1
1NFD	H	32	0	−1
1NFD	H	33	0	−1
1NFD	H	34	0	−1
1NFD	H	35	0	−1
1NFD	H	36	0	−1
1NFD	H	37	1	−1
1NFD	H	38	1	−1
1NFD	H	39	1	−1
1NFD	H	40	1	−1
1NFD	H	41	1	−1
1NFD	H	42	0	−1
1NFD	H	43	1	−1
1NFD	H	44	0	−1
1NFD	H	45	0	−1
1NFD	H	46	0	−1
1NFD	H	47	0	−1
1NFD	H	48	0	−1

1NFD	H	49	0	−1
1NFD	H	50	1	1
1NFD	H	51	1	1
1NFD	H	52	1	−1
1NFD	H	53	0	−1
1NFD	H	54	1	−1
1NFD	H	55	1	−1
1NFD	H	56	0	−1
1NFD	H	57	1	−1
1NFD	H	58	1	−1
1NFD	H	59	0	−1
1NFD	H	60	0	−1
1NFD	H	61	1	−1
1NFD	H	62	0	−1
1NFD	H	63	1	−1
1NFD	H	64	1	−1
1NFD	H	65	1	1
1NFD	H	66	1	1
1NFD	H	67	1	−1
1NFD	H	68	1	−1
1NFD	H	69	0	−1
1NFD	H	70	0	−1
1NFD	H	71	0	−1
1NFD	H	72	0	−1
1NFD	H	73	0	−1
1NFD	H	74	1	−1
1NFD	H	75	1	−1
1NFD	H	76	0	−1
1NFD	H	77	1	−1
1NFD	H	78	0	−1
1NFD	H	79	1	−1
1NFD	H	80	0	−1
1NFD	H	81	1	−1
1NFD	H	82	0	−1
1NFD	H	83	0	−1
1NFD	H	84	0	−1
1NFD	H	85	0	−1
1NFD	H	86	0	−1
1NFD	H	87	0	−1
1NFD	H	88	0	−1
1NFD	H	89	0	−1
1NFD	H	90	0	−1
1NFD	H	91	1	−1
1NFD	H	92	1	−1
1NFD	H	93	1	−1
1NFD	H	94	1	1
1NFD	H	95	0	−1
1NFD	H	96	0	−1
1NFD	H	97	0	−1
1NFD	H	98	0	−1
1NFD	H	99	0	−1
1NFD	H	100	1	−1

1NFD	H	101	0	−1
1NFD	H	102	0	−1
1NFD	H	103	1	−1
1NFD	H	104	0	−1
1NFD	H	105	1	−1
1NFD	H	106	0	−1
1NFD	H	107	1	−1
1NFD	H	108	1	−1
1NFD	H	109	1	−1
1NFD	H	110	1	−1
1NFD	H	111	1	−1
1NFD	H	112	1	−1
1NFD	H	113	1	−1
1NFD	H	114	0	−1
1NFD	H	115	1	−1
1NFD	H	116	0	−1
1NFD	H	117	1	−1
1NFD	H	118	0	−1
1NFD	H	119	0	−1
1NFD	H	120	0	−1
1NFD	H	121	0	−1
1NFD	H	122	0	−1
1NFD	H	123	1	−1
1NFD	H	124	1	−1
1NFD	H	125	0	−1
1NFD	H	126	1	−1
1NFD	H	127	1	−1
1NFD	H	128	1	−1
1NFD	H	129	1	−1
1NFD	H	130	1	−1
1NFD	H	131	0	−1
1NFD	H	132	0	−1
1NFD	H	133	0	−1
1NFD	H	134	0	−1
1NFD	H	135	0	−1
1NFD	H	136	0	−1
1NFD	H	137	0	−1
1NFD	H	138	0	−1
1NFD	H	139	1	−1
1NFD	H	140	0	−1
1NFD	H	141	0	−1
1NFD	H	142	1	−1
1NFD	H	143	0	−1
1NFD	H	144	1	−1
1NFD	H	145	0	−1
1NFD	H	146	1	−1
1NFD	H	147	0	−1
1NFD	H	148	1	−1
1NFD	H	149	0	−1
1NFD	H	150	1	−1
1NFD	H	151	0	−1
1NFD	H	152	1	−1

1NFD	H	153	1	−1
1NFD	H	154	1	−1
1NFD	H	155	1	−1
1NFD	H	156	0	−1
1NFD	H	157	1	−1
1NFD	H	158	1	−1
1NFD	H	159	1	−1
1NFD	H	160	1	−1
1NFD	H	161	1	−1
1NFD	H	162	1	−1
1NFD	H	163	0	−1
1NFD	H	164	1	−1
1NFD	H	165	0	−1
1NFD	H	166	1	−1
1NFD	H	167	1	−1
1NFD	H	168	1	−1
1NFD	H	169	1	−1
1NFD	H	170	1	−1
1NFD	H	171	0	−1
1NFD	H	172	0	−1
1NFD	H	173	0	−1
1NFD	H	174	0	−1
1NFD	H	175	0	−1
1NFD	H	176	0	−1
1NFD	H	177	0	−1
1NFD	H	178	0	−1
1NFD	H	179	0	−1
1NFD	H	180	0	−1
1NFD	H	181	1	−1
1NFD	H	182	0	−1
1NFD	H	183	1	−1
1NFD	H	184	1	−1
1NFD	H	185	0	−1
1NFD	H	186	1	−1
1NFD	H	187	1	−1
1NFD	H	188	0	−1
1NFD	H	189	1	−1
1NFD	H	190	1	−1
1NFD	H	191	0	−1
1NFD	H	192	0	−1
1NFD	H	193	0	−1
1NFD	H	194	0	−1
1NFD	H	195	0	−1
1NFD	H	196	1	−1
1NFD	H	197	0	−1
1NFD	H	198	1	−1
1NFD	H	199	1	−1
1NFD	H	200	1	−1
1NFD	H	201	1	−1
1NFD	H	202	0	−1
1NFD	H	203	1	−1
1NFD	H	204	1	−1

1NFD	H	205	1	−1
1NFD	H	206	0	−1
1NFD	H	207	1	−1
1NFD	H	208	0	−1
1NFD	H	209	1	−1
1NFD	H	210	1	−1
1NFD	H	211	1	−1
1NFD	H	212	1	−1
1NFD	H	1	1	−1
1NFD	H	2	0	−1
1NFD	H	3	1	−1
1NFD	H	4	0	−1
1NFD	H	5	1	−1
1NFD	H	6	0	−1
1NFD	H	7	1	−1
1NFD	H	8	1	−1
1NFD	H	9	1	−1
1NFD	H	10	1	−1
1NFD	H	11	0	−1
1NFD	H	12	0	−1
1NFD	H	13	1	−1
1NFD	H	14	1	−1
1NFD	H	15	1	−1
1NFD	H	16	1	−1
1NFD	H	17	1	−1
1NFD	H	18	0	−1
1NFD	H	19	1	−1
1NFD	H	20	0	−1
1NFD	H	21	0	−1
1NFD	H	22	0	−1
1NFD	H	23	1	−1
1NFD	H	24	0	−1
1NFD	H	25	1	−1
1NFD	H	26	1	−1
1NFD	H	27	0	−1
1NFD	H	28	1	−1
1NFD	H	29	0	−1
1NFD	H	30	1	−1
1NFD	H	31	1	−1
1NFD	H	32	0	−1
1NFD	H	33	1	1
1NFD	H	34	0	−1
1NFD	H	35	0	−1
1NFD	H	36	0	−1
1NFD	H	37	0	−1
1NFD	H	38	0	−1
1NFD	H	39	0	−1
1NFD	H	40	0	−1
1NFD	H	41	1	−1
1NFD	H	42	1	−1
1NFD	H	43	1	−1
1NFD	H	44	1	−1

1NFD	H	45	0	−1
1NFD	H	46	1	−1
1NFD	H	47	0	−1
1NFD	H	48	0	−1
1NFD	H	49	0	−1
1NFD	H	50	0	−1
1NFD	H	51	0	−1
1NFD	H	52	1	−1
1NFD	H	53	0	−1
1NFD	H	54	1	−1
1NFD	H	55	1	−1
1NFD	H	56	1	1
1NFD	H	57	1	−1
1NFD	H	58	1	−1
1NFD	H	59	1	−1
1NFD	H	60	1	−1
1NFD	H	61	0	−1
1NFD	H	62	0	−1
1NFD	H	63	0	−1
1NFD	H	64	1	−1
1NFD	H	65	1	−1
1NFD	H	66	0	−1
1NFD	H	67	1	−1
1NFD	H	68	1	−1
1NFD	H	69	0	−1
1NFD	H	70	0	−1
1NFD	H	71	1	−1
1NFD	H	72	0	−1
1NFD	H	73	1	−1
1NFD	H	74	0	−1
1NFD	H	75	1	−1
1NFD	H	76	1	−1
1NFD	H	77	1	−1
1NFD	H	78	1	−1
1NFD	H	79	1	−1
1NFD	H	80	0	−1
1NFD	H	81	0	−1
1NFD	H	82	0	−1
1NFD	H	83	0	−1
1NFD	H	84	1	−1
1NFD	H	85	0	−1
1NFD	H	86	1	−1
1NFD	H	87	1	−1
1NFD	H	88	0	−1
1NFD	H	89	1	−1
1NFD	H	90	1	−1
1NFD	H	91	1	−1
1NFD	H	92	0	−1
1NFD	H	93	0	−1
1NFD	H	94	0	−1
1NFD	H	95	0	−1
1NFD	H	96	0	−1

1NFD	H	97	0	−1
1NFD	H	98	0	−1
1NFD	H	99	0	−1
1NFD	H	100	0	−1
1NFD	H	101	0	−1
1NFD	H	102	0	−1
1NFD	H	103	1	1
1NFD	H	104	1	1
1NFD	H	105	1	−1
1NFD	H	106	0	−1
1NFD	H	107	0	−1
1NFD	H	108	1	−1
1NFD	H	109	1	−1
1NFD	H	110	0	−1
1NFD	H	111	0	−1
1NFD	H	112	1	−1
1NFD	H	113	0	−1
1NFD	H	114	0	−1
1NFD	H	115	0	−1
1NFD	H	116	0	−1
1NFD	H	117	0	−1
1NFD	H	118	0	−1
1NFD	H	119	0	−1
1NFD	H	120	1	−1
1NFD	H	121	1	−1
1NFD	H	122	1	−1
1NFD	H	123	1	−1
1NFD	H	124	1	−1
1NFD	H	125	1	−1
1NFD	H	126	0	−1
1NFD	H	127	1	−1
1NFD	H	128	0	−1
1NFD	H	129	0	−1
1NFD	H	130	0	−1
1NFD	H	131	0	−1
1NFD	H	132	0	−1
1NFD	H	133	1	−1
1NFD	H	134	1	−1
1NFD	H	135	1	−1
1NFD	H	136	1	−1
1NFD	H	137	1	−1
1NFD	H	138	1	−1
1NFD	H	139	1	−1
1NFD	H	140	1	−1
1NFD	H	141	1	−1
1NFD	H	142	1	−1
1NFD	H	143	1	−1
1NFD	H	144	1	−1
1NFD	H	145	0	−1
1NFD	H	146	0	−1
1NFD	H	147	0	−1
1NFD	H	148	0	−1

1NFD	H	149	0	−1
1NFD	H	150	0	−1
1NFD	H	151	0	−1
1NFD	H	152	0	−1
1NFD	H	153	0	−1
1NFD	H	154	0	−1
1NFD	H	155	0	−1
1NFD	H	156	0	−1
1NFD	H	157	1	−1
1NFD	H	158	1	−1
1NFD	H	159	0	−1
1NFD	H	160	1	−1
1NFD	H	161	0	−1
1NFD	H	162	1	−1
1NFD	H	163	0	−1
1NFD	H	164	0	−1
1NFD	H	165	1	−1
1NFD	H	166	1	−1
1NFD	H	167	1	−1
1NFD	H	168	0	−1
1NFD	H	169	1	−1
1NFD	H	170	1	−1
1NFD	H	171	0	−1
1NFD	H	172	0	−1
1NFD	H	173	0	−1
1NFD	H	174	1	−1
1NFD	H	175	0	−1
1NFD	H	176	1	−1
1NFD	H	177	1	−1
1NFD	H	178	0	−1
1NFD	H	179	1	−1
1NFD	H	180	1	−1
1NFD	H	181	1	−1
1NFD	H	182	1	−1
1NFD	H	183	1	−1
1NFD	H	184	1	−1
1NFD	H	185	0	−1
1NFD	H	186	0	−1
1NFD	H	187	0	−1
1NFD	H	188	0	−1
1NFD	H	189	0	−1
1NFD	H	190	0	−1
1NFD	H	191	0	−1
1NFD	H	192	0	−1
1NFD	H	193	1	−1
1NFD	H	194	1	−1
1NFD	H	195	1	−1
1NFD	H	196	1	−1
1NFD	H	197	0	−1
1NFD	H	198	1	−1
1NFD	H	199	1	−1
1NFD	H	200	1	−1

1NFD	H	201	1	−1
1NFD	H	202	0	−1
1NFD	H	203	1	−1
1NFD	H	204	0	−1
1NFD	H	205	0	−1
1NFD	H	206	0	−1
1NFD	H	207	0	−1
1NFD	H	208	0	−1
1NFD	H	209	1	−1
1NFD	H	210	0	−1
1NFD	H	211	1	−1
1NFD	H	212	1	−1
1NFD	H	213	0	−1
1NFD	H	214	1	−1
1NFD	H	215	0	−1
1NFD	H	216	1	−1
1NFD	H	217	1	−1
1NFD	H	218	1	−1
1NFD	H	219	0	−1
1NFD	H	220	1	−1
1NFD	H	221	1	−1
1NFD	H	222	1	−1
1NFD	H	1	1	−1
1NFD	H	2	1	−1
1NFD	H	3	0	−1
1NFD	H	4	1	−1
1NFD	H	5	0	−1
1NFD	H	6	1	−1
1NFD	H	7	1	−1
1NFD	H	8	1	−1
1NFD	H	9	0	−1
1NFD	H	10	1	−1
1NFD	H	11	0	−1
1NFD	H	12	1	−1
1NFD	H	13	1	−1
1NFD	H	14	1	−1
1NFD	H	15	1	−1
1NFD	H	16	1	−1
1NFD	H	17	0	−1
1NFD	H	18	1	−1
1NFD	H	19	0	−1
1NFD	H	20	1	−1
1NFD	H	21	0	−1
1NFD	H	22	1	−1
1NFD	H	23	0	−1
1NFD	H	24	1	−1
1NFD	H	25	1	−1
1NFD	H	26	0	−1
1NFD	H	27	1	1
1NFD	H	28	1	1
1NFD	H	29	0	−1
1NFD	H	30	1	1

1NFD	H	31	0	−1
1NFD	H	32	0	−1
1NFD	H	33	0	−1
1NFD	H	34	0	−1
1NFD	H	35	0	−1
1NFD	H	36	0	−1
1NFD	H	37	1	−1
1NFD	H	38	1	−1
1NFD	H	39	1	−1
1NFD	H	40	1	−1
1NFD	H	41	1	−1
1NFD	H	42	0	−1
1NFD	H	43	1	−1
1NFD	H	44	0	−1
1NFD	H	45	0	−1
1NFD	H	46	0	−1
1NFD	H	47	0	−1
1NFD	H	48	1	1
1NFD	H	49	0	−1
1NFD	H	50	1	−1
1NFD	H	51	1	1
1NFD	H	52	1	−1
1NFD	H	53	0	−1
1NFD	H	54	1	−1
1NFD	H	55	1	−1
1NFD	H	56	0	−1
1NFD	H	57	1	−1
1NFD	H	58	1	−1
1NFD	H	59	0	−1
1NFD	H	60	0	−1
1NFD	H	61	1	−1
1NFD	H	62	0	−1
1NFD	H	63	1	−1
1NFD	H	64	1	1
1NFD	H	65	1	−1
1NFD	H	66	1	−1
1NFD	H	67	1	−1
1NFD	H	68	1	−1
1NFD	H	69	0	−1
1NFD	H	70	0	−1
1NFD	H	71	0	−1
1NFD	H	72	0	−1
1NFD	H	73	0	−1
1NFD	H	74	1	−1
1NFD	H	75	1	−1
1NFD	H	76	0	−1
1NFD	H	77	1	−1
1NFD	H	78	0	−1
1NFD	H	79	1	−1
1NFD	H	80	0	−1
1NFD	H	81	1	−1
1NFD	H	82	0	−1

1NFD	H	83	0	−1
1NFD	H	84	0	−1
1NFD	H	85	0	−1
1NFD	H	86	0	−1
1NFD	H	87	0	−1
1NFD	H	88	0	−1
1NFD	H	89	0	−1
1NFD	H	90	0	−1
1NFD	H	91	1	−1
1NFD	H	92	1	−1
1NFD	H	93	1	−1
1NFD	H	94	1	1
1NFD	H	95	0	−1
1NFD	H	96	0	−1
1NFD	H	97	0	−1
1NFD	H	98	0	−1
1NFD	H	99	0	−1
1NFD	H	100	1	−1
1NFD	H	101	0	−1
1NFD	H	102	0	−1
1NFD	H	103	1	−1
1NFD	H	104	0	−1
1NFD	H	105	1	−1
1NFD	H	106	0	−1
1NFD	H	107	1	−1
1NFD	H	108	1	−1
1NFD	H	109	1	−1
1NFD	H	110	1	−1
1NFD	H	111	1	−1
1NFD	H	112	1	−1
1NFD	H	113	1	−1
1NFD	H	114	0	−1
1NFD	H	115	1	−1
1NFD	H	116	0	−1
1NFD	H	117	0	−1
1NFD	H	118	0	−1
1NFD	H	119	0	−1
1NFD	H	120	1	−1
1NFD	H	121	0	−1
1NFD	H	122	0	−1
1NFD	H	123	1	−1
1NFD	H	124	1	−1
1NFD	H	125	0	−1
1NFD	H	126	0	−1
1NFD	H	127	1	−1
1NFD	H	128	1	−1
1NFD	H	129	1	−1
1NFD	H	130	1	−1
1NFD	H	131	0	−1
1NFD	H	132	0	−1
1NFD	H	133	0	−1
1NFD	H	134	0	−1

1NFD	H	135	0	−1
1NFD	H	136	0	−1
1NFD	H	137	0	−1
1NFD	H	138	0	−1
1NFD	H	139	1	−1
1NFD	H	140	0	−1
1NFD	H	141	0	−1
1NFD	H	142	1	−1
1NFD	H	143	0	−1
1NFD	H	144	1	−1
1NFD	H	145	0	−1
1NFD	H	146	1	−1
1NFD	H	147	0	−1
1NFD	H	148	1	−1
1NFD	H	149	0	−1
1NFD	H	150	1	−1
1NFD	H	151	0	−1
1NFD	H	152	1	−1
1NFD	H	153	1	−1
1NFD	H	154	1	−1
1NFD	H	155	1	−1
1NFD	H	156	0	−1
1NFD	H	157	1	−1
1NFD	H	158	1	−1
1NFD	H	159	1	−1
1NFD	H	160	1	−1
1NFD	H	161	1	−1
1NFD	H	162	1	−1
1NFD	H	163	1	−1
1NFD	H	164	1	−1
1NFD	H	165	0	−1
1NFD	H	166	1	−1
1NFD	H	167	1	−1
1NFD	H	168	1	−1
1NFD	H	169	1	−1
1NFD	H	170	1	−1
1NFD	H	171	0	−1
1NFD	H	172	0	−1
1NFD	H	173	0	−1
1NFD	H	174	0	−1
1NFD	H	175	0	−1
1NFD	H	176	0	−1
1NFD	H	177	0	−1
1NFD	H	178	0	−1
1NFD	H	179	0	−1
1NFD	H	180	0	−1
1NFD	H	181	1	−1
1NFD	H	182	0	−1
1NFD	H	183	1	−1
1NFD	H	184	1	−1
1NFD	H	185	0	−1
1NFD	H	186	1	−1

1NFD	H	187	1	−1
1NFD	H	188	1	−1
1NFD	H	189	1	−1
1NFD	H	190	1	−1
1NFD	H	191	0	−1
1NFD	H	192	0	−1
1NFD	H	193	0	−1
1NFD	H	194	0	−1
1NFD	H	195	0	−1
1NFD	H	196	1	−1
1NFD	H	197	0	−1
1NFD	H	198	1	−1
1NFD	H	199	1	−1
1NFD	H	200	1	−1
1NFD	H	201	1	−1
1NFD	H	202	0	−1
1NFD	H	203	1	−1
1NFD	H	204	1	−1
1NFD	H	205	1	−1
1NFD	H	206	1	−1
1NFD	H	207	1	−1
1NFD	H	208	0	−1
1NFD	H	209	0	−1
1NFD	H	210	1	−1
1NFD	H	211	1	−1
1NFD	H	212	1	−1
1NFD	H	1	1	−1
1NFD	H	2	0	−1
1NFD	H	3	1	−1
1NFD	H	4	0	−1
1NFD	H	5	1	−1
1NFD	H	6	0	−1
1NFD	H	7	1	−1
1NFD	H	8	1	−1
1NFD	H	9	1	−1
1NFD	H	10	1	−1
1NFD	H	11	0	−1
1NFD	H	12	0	−1
1NFD	H	13	1	−1
1NFD	H	14	1	−1
1NFD	H	15	1	−1
1NFD	H	16	1	−1
1NFD	H	17	1	−1
1NFD	H	18	0	−1
1NFD	H	19	1	−1
1NFD	H	20	0	−1
1NFD	H	21	0	−1
1NFD	H	22	0	−1
1NFD	H	23	1	−1
1NFD	H	24	0	−1
1NFD	H	25	1	−1
1NFD	H	26	1	−1

1NFD	H	27	0	−1
1NFD	H	28	1	−1
1NFD	H	29	0	−1
1NFD	H	30	1	−1
1NFD	H	31	1	−1
1NFD	H	32	0	−1
1NFD	H	33	1	1
1NFD	H	34	0	−1
1NFD	H	35	0	−1
1NFD	H	36	0	−1
1NFD	H	37	0	−1
1NFD	H	38	0	−1
1NFD	H	39	0	−1
1NFD	H	40	0	−1
1NFD	H	41	1	−1
1NFD	H	42	1	−1
1NFD	H	43	1	−1
1NFD	H	44	1	−1
1NFD	H	45	0	−1
1NFD	H	46	1	−1
1NFD	H	47	0	−1
1NFD	H	48	0	−1
1NFD	H	49	0	−1
1NFD	H	50	0	−1
1NFD	H	51	0	−1
1NFD	H	52	1	1
1NFD	H	53	0	−1
1NFD	H	54	1	−1
1NFD	H	55	1	−1
1NFD	H	56	1	1
1NFD	H	57	1	−1
1NFD	H	58	1	−1
1NFD	H	59	1	−1
1NFD	H	60	1	−1
1NFD	H	61	0	−1
1NFD	H	62	0	−1
1NFD	H	63	0	−1
1NFD	H	64	1	−1
1NFD	H	65	1	−1
1NFD	H	66	0	−1
1NFD	H	67	1	−1
1NFD	H	68	1	−1
1NFD	H	69	0	−1
1NFD	H	70	0	−1
1NFD	H	71	1	−1
1NFD	H	72	0	−1
1NFD	H	73	1	−1
1NFD	H	74	0	−1
1NFD	H	75	0	−1
1NFD	H	76	1	−1
1NFD	H	77	1	−1
1NFD	H	78	1	−1

1NFD	H	79	1	−1
1NFD	H	80	0	−1
1NFD	H	81	0	−1
1NFD	H	82	0	−1
1NFD	H	83	0	−1
1NFD	H	84	1	−1
1NFD	H	85	0	−1
1NFD	H	86	1	−1
1NFD	H	87	1	−1
1NFD	H	88	0	−1
1NFD	H	89	1	−1
1NFD	H	90	1	−1
1NFD	H	91	1	−1
1NFD	H	92	0	−1
1NFD	H	93	0	−1
1NFD	H	94	0	−1
1NFD	H	95	0	−1
1NFD	H	96	0	−1
1NFD	H	97	0	−1
1NFD	H	98	0	−1
1NFD	H	99	0	−1
1NFD	H	100	0	−1
1NFD	H	101	0	−1
1NFD	H	102	0	−1
1NFD	H	103	1	1
1NFD	H	104	1	1
1NFD	H	105	1	−1
1NFD	H	106	0	−1
1NFD	H	107	0	−1
1NFD	H	108	1	−1
1NFD	H	109	1	−1
1NFD	H	110	0	−1
1NFD	H	111	0	−1
1NFD	H	112	1	−1
1NFD	H	113	0	−1
1NFD	H	114	0	−1
1NFD	H	115	0	−1
1NFD	H	116	0	−1
1NFD	H	117	0	−1
1NFD	H	118	0	−1
1NFD	H	119	0	−1
1NFD	H	120	1	−1
1NFD	H	121	1	−1
1NFD	H	122	1	−1
1NFD	H	123	1	−1
1NFD	H	124	1	−1
1NFD	H	125	1	−1
1NFD	H	126	0	−1
1NFD	H	127	1	−1
1NFD	H	128	0	−1
1NFD	H	129	0	−1
1NFD	H	130	0	−1

1NFD	H	131	0	−1
1NFD	H	132	1	−1
1NFD	H	133	1	−1
1NFD	H	134	1	−1
1NFD	H	135	1	−1
1NFD	H	136	1	−1
1NFD	H	137	1	−1
1NFD	H	138	1	−1
1NFD	H	139	1	−1
1NFD	H	140	1	−1
1NFD	H	141	1	−1
1NFD	H	142	1	−1
1NFD	H	143	1	−1
1NFD	H	144	1	−1
1NFD	H	145	0	−1
1NFD	H	146	0	−1
1NFD	H	147	0	−1
1NFD	H	148	0	−1
1NFD	H	149	0	−1
1NFD	H	150	0	−1
1NFD	H	151	0	−1
1NFD	H	152	0	−1
1NFD	H	153	0	−1
1NFD	H	154	0	−1
1NFD	H	155	0	−1
1NFD	H	156	0	−1
1NFD	H	157	1	−1
1NFD	H	158	1	−1
1NFD	H	159	0	−1
1NFD	H	160	1	−1
1NFD	H	161	0	−1
1NFD	H	162	1	−1
1NFD	H	163	0	−1
1NFD	H	164	0	−1
1NFD	H	165	1	−1
1NFD	H	166	1	−1
1NFD	H	167	1	−1
1NFD	H	168	0	−1
1NFD	H	169	1	−1
1NFD	H	170	1	−1
1NFD	H	171	0	−1
1NFD	H	172	0	−1
1NFD	H	173	0	−1
1NFD	H	174	1	−1
1NFD	H	175	0	−1
1NFD	H	176	1	−1
1NFD	H	177	1	−1
1NFD	H	178	0	−1
1NFD	H	179	1	−1
1NFD	H	180	1	−1
1NFD	H	181	1	−1
1NFD	H	182	1	−1

1NFD	H	183	1	−1
1NFD	H	184	1	−1
1NFD	H	185	0	−1
1NFD	H	186	0	−1
1NFD	H	187	0	−1
1NFD	H	188	0	−1
1NFD	H	189	0	−1
1NFD	H	190	0	−1
1NFD	H	191	1	−1
1NFD	H	192	0	−1
1NFD	H	193	1	−1
1NFD	H	194	1	−1
1NFD	H	195	1	−1
1NFD	H	196	1	−1
1NFD	H	197	1	−1
1NFD	H	198	1	−1
1NFD	H	199	1	−1
1NFD	H	200	1	−1
1NFD	H	201	1	−1
1NFD	H	202	0	−1
1NFD	H	203	0	−1
1NFD	H	204	0	−1
1NFD	H	205	0	−1
1NFD	H	206	0	−1
1NFD	H	207	0	−1
1NFD	H	208	0	−1
1NFD	H	209	1	−1
1NFD	H	210	1	−1
1NFD	H	211	1	−1
1NFD	H	212	1	−1
1NFD	H	213	0	−1
1NFD	H	214	1	−1
1NFD	H	215	0	−1
1NFD	H	216	1	−1
1NFD	H	217	1	−1
1NFD	H	218	1	−1
1NFD	H	219	0	−1
1NFD	H	220	1	−1
1NFD	H	221	1	−1
1NFD	H	222	1	−1
1NMB	L	1	1	−1
1NMB	L	2	0	−1
1NMB	L	3	1	−1
1NMB	L	4	0	−1
1NMB	L	5	1	−1
1NMB	L	6	0	−1
1NMB	L	7	1	−1
1NMB	L	8	1	−1
1NMB	L	9	1	−1
1NMB	L	10	1	−1
1NMB	L	11	1	−1
1NMB	L	12	1	−1

1NMB	L	13	0	−1
1NMB	L	14	1	−1
1NMB	L	15	1	−1
1NMB	L	16	1	−1
1NMB	L	17	1	−1
1NMB	L	18	1	−1
1NMB	L	19	0	−1
1NMB	L	20	1	−1
1NMB	L	21	0	−1
1NMB	L	22	1	−1
1NMB	L	23	0	−1
1NMB	L	24	1	−1
1NMB	L	25	0	−1
1NMB	L	26	1	−1
1NMB	L	27	1	1
1NMB	L	28	1	1
1NMB	L	29	0	−1
1NMB	L	30	1	1
1NMB	L	31	1	1
1NMB	L	32	1	1
1NMB	L	33	0	−1
1NMB	L	34	0	−1
1NMB	L	35	0	−1
1NMB	L	36	0	−1
1NMB	L	37	0	−1
1NMB	L	38	0	−1
1NMB	L	39	1	−1
1NMB	L	40	1	−1
1NMB	L	41	1	−1
1NMB	L	42	0	−1
1NMB	L	43	1	−1
1NMB	L	44	0	−1
1NMB	L	45	1	−1
1NMB	L	46	0	−1
1NMB	L	47	0	−1
1NMB	L	48	0	−1
1NMB	L	49	1	−1
1NMB	L	50	1	1
1NMB	L	51	0	−1
1NMB	L	52	1	−1
1NMB	L	53	1	−1
1NMB	L	54	1	−1
1NMB	L	55	0	−1
1NMB	L	56	1	−1
1NMB	L	57	1	−1
1NMB	L	58	0	−1
1NMB	L	59	1	−1
1NMB	L	60	1	−1
1NMB	L	61	0	−1
1NMB	L	62	0	−1
1NMB	L	63	1	−1
1NMB	L	64	0	−1

1NMB	L	65	1	−1
1NMB	L	66	1	−1
1NMB	L	67	1	−1
1NMB	L	68	1	−1
1NMB	L	69	1	−1
1NMB	L	70	1	−1
1NMB	L	71	0	−1
1NMB	L	72	0	−1
1NMB	L	73	0	−1
1NMB	L	74	0	−1
1NMB	L	75	0	−1
1NMB	L	76	0	−1
1NMB	L	77	1	−1
1NMB	L	78	0	−1
1NMB	L	79	1	−1
1NMB	L	80	1	−1
1NMB	L	81	1	−1
1NMB	L	82	0	−1
1NMB	L	83	0	−1
1NMB	L	84	0	−1
1NMB	L	85	0	−1
1NMB	L	86	0	−1
1NMB	L	87	0	−1
1NMB	L	88	0	−1
1NMB	L	89	0	−1
1NMB	L	90	0	−1
1NMB	L	91	0	−1
1NMB	L	92	1	1
1NMB	L	93	1	1
1NMB	L	94	1	1
1NMB	L	95	0	−1
1NMB	L	96	0	−1
1NMB	L	97	0	−1
1NMB	L	98	0	−1
1NMB	L	99	0	−1
1NMB	L	100	1	−1
1NMB	L	101	0	−1
1NMB	L	102	0	−1
1NMB	L	103	1	−1
1NMB	L	104	0	−1
1NMB	L	105	1	−1
1NMB	L	106	1	−1
1NMB	L	107	1	−1
1NMB	L	108	1	−1
1NMB	L	109	1	−1
1NMB	H	1	1	−1
1NMB	H	2	0	−1
1NMB	H	3	1	−1
1NMB	H	4	0	−1
1NMB	H	5	1	−1
1NMB	H	6	0	−1
1NMB	H	7	1	−1

1NMB	H	8	1	−1
1NMB	H	9	1	−1
1NMB	H	10	1	−1
1NMB	H	11	1	−1
1NMB	H	12	0	−1
1NMB	H	13	1	−1
1NMB	H	14	1	−1
1NMB	H	15	1	−1
1NMB	H	16	1	−1
1NMB	H	17	1	−1
1NMB	H	18	0	−1
1NMB	H	19	1	−1
1NMB	H	20	0	−1
1NMB	H	21	1	−1
1NMB	H	22	0	−1
1NMB	H	23	1	−1
1NMB	H	24	0	−1
1NMB	H	25	1	−1
1NMB	H	26	1	−1
1NMB	H	27	0	−1
1NMB	H	28	1	−1
1NMB	H	29	0	−1
1NMB	H	30	1	−1
1NMB	H	31	1	−1
1NMB	H	32	0	−1
1NMB	H	33	0	−1
1NMB	H	34	0	−1
1NMB	H	35	0	−1
1NMB	H	36	0	−1
1NMB	H	37	0	−1
1NMB	H	38	0	−1
1NMB	H	39	0	−1
1NMB	H	40	0	−1
1NMB	H	41	1	−1
1NMB	H	42	1	−1
1NMB	H	43	1	−1
1NMB	H	44	1	−1
1NMB	H	45	0	−1
1NMB	H	46	0	−1
1NMB	H	47	0	−1
1NMB	H	48	0	−1
1NMB	H	49	0	−1
1NMB	H	50	0	−1
1NMB	H	51	0	−1
1NMB	H	52	0	−1
1NMB	H	53	0	−1
1NMB	H	54	1	1
1NMB	H	55	1	1
1NMB	H	56	1	−1
1NMB	H	57	1	1
1NMB	H	58	1	1
1NMB	H	59	1	1

1NMB	H	60	0	−1
1NMB	H	61	0	−1
1NMB	H	62	1	−1
1NMB	H	63	1	−1
1NMB	H	64	0	−1
1NMB	H	65	1	−1
1NMB	H	66	1	−1
1NMB	H	67	0	−1
1NMB	H	68	0	−1
1NMB	H	69	1	−1
1NMB	H	70	0	−1
1NMB	H	71	1	−1
1NMB	H	72	0	−1
1NMB	H	73	1	−1
1NMB	H	74	1	−1
1NMB	H	75	1	−1
1NMB	H	76	1	−1
1NMB	H	77	1	−1
1NMB	H	78	0	−1
1NMB	H	79	0	−1
1NMB	H	80	0	−1
1NMB	H	81	0	−1
1NMB	H	82	1	−1
1NMB	H	83	0	−1
1NMB	H	84	1	−1
1NMB	H	85	1	−1
1NMB	H	86	0	−1
1NMB	H	87	1	−1
1NMB	H	88	1	−1
1NMB	H	89	1	−1
1NMB	H	90	0	−1
1NMB	H	91	1	−1
1NMB	H	92	0	−1
1NMB	H	93	0	−1
1NMB	H	94	0	−1
1NMB	H	95	0	−1
1NMB	H	96	0	−1
1NMB	H	97	0	−1
1NMB	H	98	0	−1
1NMB	H	99	0	−1
1NMB	H	100	1	−1
1NMB	H	101	0	−1
1NMB	H	102	0	−1
1NMB	H	103	1	1
1NMB	H	104	1	−1
1NMB	H	105	1	1
1NMB	H	106	0	−1
1NMB	H	107	0	−1
1NMB	H	108	0	−1
1NMB	H	109	0	−1
1NMB	H	110	0	−1
1NMB	H	111	1	−1

1NMB	H	112	0	−1
1NMB	H	113	0	−1
1NMB	H	114	1	−1
1NMB	H	115	0	−1
1NMB	H	116	0	−1
1NMB	H	117	1	−1
1NMB	H	118	0	−1
1NMB	H	119	1	−1
1NMB	H	120	0	−1
1NMB	H	121	1	−1
1NMB	H	122	1	−1
1NMB	N	83	1	−1
1NMB	N	84	1	−1
1NMB	N	85	0	−1
1NMB	N	86	0	−1
1NMB	N	87	1	−1
1NMB	N	88	0	−1
1NMB	N	89	1	−1
1NMB	N	90	1	−1
1NMB	N	91	0	−1
1NMB	N	92	0	−1
1NMB	N	93	0	−1
1NMB	N	94	1	−1
1NMB	N	95	0	−1
1NMB	N	96	0	−1
1NMB	N	97	0	−1
1NMB	N	98	0	−1
1NMB	N	99	1	−1
1NMB	N	100	1	−1
1NMB	N	101	1	−1
1NMB	N	102	0	−1
1NMB	N	103	0	−1
1NMB	N	104	0	−1
1NMB	N	105	1	−1
1NMB	N	106	0	−1
1NMB	N	107	0	−1
1NMB	N	108	1	−1
1NMB	N	109	1	−1
1NMB	N	110	0	−1
1NMB	N	111	1	−1
1NMB	N	112	1	−1
1NMB	N	113	1	−1
1NMB	N	114	1	−1
1NMB	N	115	0	−1
1NMB	N	116	0	−1
1NMB	N	117	0	−1
1NMB	N	118	0	−1
1NMB	N	119	0	−1
1NMB	N	120	0	−1
1NMB	N	121	0	−1
1NMB	N	122	0	−1
1NMB	N	123	0	−1

1NMB	N	124	0	−1
1NMB	N	125	0	−1
1NMB	N	126	0	−1
1NMB	N	127	1	−1
1NMB	N	128	1	−1
1NMB	N	129	1	−1
1NMB	N	130	0	−1
1NMB	N	131	0	−1
1NMB	N	132	0	−1
1NMB	N	133	0	−1
1NMB	N	134	0	−1
1NMB	N	135	0	−1
1NMB	N	136	0	−1
1NMB	N	137	0	−1
1NMB	N	138	1	−1
1NMB	N	139	0	−1
1NMB	N	140	0	−1
1NMB	N	141	0	−1
1NMB	N	142	1	−1
1NMB	N	143	1	−1
1NMB	N	144	1	−1
1NMB	N	145	1	−1
1NMB	N	146	0	−1
1NMB	N	147	1	−1
1NMB	N	148	1	−1
1NMB	N	149	0	−1
1NMB	N	150	1	−1
1NMB	N	151	1	−1
1NMB	N	152	1	−1
1NMB	N	153	1	−1
1NMB	N	154	0	−1
1NMB	N	155	1	−1
1NMB	N	156	1	−1
1NMB	N	157	0	−1
1NMB	N	158	0	−1
1NMB	N	159	0	−1
1NMB	N	160	0	−1
1NMB	N	161	0	−1
1NMB	N	162	0	−1
1NMB	N	163	1	−1
1NMB	N	164	1	−1
1NMB	N	165	1	−1
1NMB	N	166	0	−1
1NMB	N	167	0	−1
1NMB	N	168	0	−1
1NMB	N	169	0	−1
1NMB	N	170	1	−1
1NMB	N	171	1	−1
1NMB	N	172	1	−1
1NMB	N	173	0	−1
1NMB	N	174	1	−1
1NMB	N	175	1	−1

1NMB	N	176	0	−1
1NMB	N	177	0	−1
1NMB	N	178	1	−1
1NMB	N	179	0	−1
1NMB	N	180	0	−1
1NMB	N	181	0	−1
1NMB	N	182	0	−1
1NMB	N	183	0	−1
1NMB	N	184	0	−1
1NMB	N	185	0	−1
1NMB	N	186	0	−1
1NMB	N	187	0	−1
1NMB	N	188	0	−1
1NMB	N	189	1	−1
1NMB	N	190	0	−1
1NMB	N	191	0	−1
1NMB	N	192	0	−1
1NMB	N	193	0	−1
1NMB	N	194	0	−1
1NMB	N	195	0	−1
1NMB	N	196	0	−1
1NMB	N	197	1	−1
1NMB	N	198	1	−1
1NMB	N	199	1	−1
1NMB	N	200	1	−1
1NMB	N	201	1	−1
1NMB	N	202	1	−1
1NMB	N	203	0	−1
1NMB	N	204	1	−1
1NMB	N	205	0	−1
1NMB	N	206	1	−1
1NMB	N	207	0	−1
1NMB	N	208	1	−1
1NMB	N	209	0	−1
1NMB	N	210	1	−1
1NMB	N	211	1	−1
1NMB	N	212	1	−1
1NMB	N	213	1	−1
1NMB	N	214	1	−1
1NMB	N	215	0	−1
1NMB	N	216	1	−1
1NMB	N	217	0	−1
1NMB	N	218	1	−1
1NMB	N	219	0	−1
1NMB	N	220	0	−1
1NMB	N	221	0	−1
1NMB	N	222	1	−1
1NMB	N	223	1	−1
1NMB	N	224	0	−1
1NMB	N	225	0	−1
1NMB	N	226	0	−1
1NMB	N	227	0	−1

1NMB	N	228	0	−1
1NMB	N	229	0	−1
1NMB	N	230	0	−1
1NMB	N	231	0	−1
1NMB	N	232	0	−1
1NMB	N	233	0	−1
1NMB	N	234	0	−1
1NMB	N	235	0	−1
1NMB	N	236	1	−1
1NMB	N	237	0	−1
1NMB	N	238	0	−1
1NMB	N	239	0	−1
1NMB	N	240	0	−1
1NMB	N	241	0	−1
1NMB	N	242	0	−1
1NMB	N	243	0	−1
1NMB	N	244	0	−1
1NMB	N	245	0	−1
1NMB	N	246	0	−1
1NMB	N	247	1	−1
1NMB	N	248	1	−1
1NMB	N	249	1	−1
1NMB	N	250	0	−1
1NMB	N	251	1	−1
1NMB	N	252	1	−1
1NMB	N	253	1	−1
1NMB	N	254	0	−1
1NMB	N	255	0	−1
1NMB	N	256	0	−1
1NMB	N	257	0	−1
1NMB	N	258	0	−1
1NMB	N	259	0	−1
1NMB	N	260	1	−1
1NMB	N	261	1	−1
1NMB	N	262	0	−1
1NMB	N	263	1	−1
1NMB	N	264	1	−1
1NMB	N	265	1	−1
1NMB	N	266	1	−1
1NMB	N	267	1	−1
1NMB	N	268	0	−1
1NMB	N	269	1	−1
1NMB	N	270	1	−1
1NMB	N	271	1	−1
1NMB	N	272	1	−1
1NMB	N	273	1	−1
1NMB	N	274	0	−1
1NMB	N	275	1	−1
1NMB	N	276	0	−1
1NMB	N	277	0	−1
1NMB	N	278	0	−1
1NMB	N	279	0	−1

1NMB	N	280	0	−1
1NMB	N	281	0	−1
1NMB	N	282	0	−1
1NMB	N	283	0	−1
1NMB	N	284	0	−1
1NMB	N	285	0	−1
1NMB	N	286	1	−1
1NMB	N	287	1	−1
1NMB	N	288	0	−1
1NMB	N	289	0	−1
1NMB	N	290	0	−1
1NMB	N	291	0	−1
1NMB	N	292	0	−1
1NMB	N	293	0	−1
1NMB	N	294	0	−1
1NMB	N	295	0	−1
1NMB	N	296	0	−1
1NMB	N	297	1	−1
1NMB	N	298	1	−1
1NMB	N	299	0	−1
1NMB	N	300	0	−1
1NMB	N	301	0	−1
1NMB	N	302	0	−1
1NMB	N	303	0	−1
1NMB	N	304	0	−1
1NMB	N	305	0	−1
1NMB	N	306	1	−1
1NMB	N	307	0	−1
1NMB	N	308	1	−1
1NMB	N	309	0	−1
1NMB	N	310	1	−1
1NMB	N	311	1	−1
1NMB	N	312	0	−1
1NMB	N	313	1	−1
1NMB	N	314	0	−1
1NMB	N	315	1	−1
1NMB	N	316	0	−1
1NMB	N	317	1	−1
1NMB	N	318	0	−1
1NMB	N	319	0	−1
1NMB	N	320	0	−1
1NMB	N	321	0	−1
1NMB	N	322	0	−1
1NMB	N	323	0	−1
1NMB	N	324	0	−1
1NMB	N	325	0	−1
1NMB	N	326	0	−1
1NMB	N	327	0	−1
1NMB	N	328	1	−1
1NMB	N	329	0	−1
1NMB	N	330	0	−1
1NMB	N	331	1	1

1NMB	N	332	1	1
1NMB	N	333	0	−1
1NMB	N	334	1	1
1NMB	N	335	1	−1
1NMB	N	336	0	−1
1NMB	N	337	1	−1
1NMB	N	338	0	−1
1NMB	N	339	1	−1
1NMB	N	340	1	−1
1NMB	N	341	0	−1
1NMB	N	342	0	−1
1NMB	N	343	1	1
1NMB	N	344	1	1
1NMB	N	345	1	1
1NMB	N	346	1	−1
1NMB	N	347	1	−1
1NMB	N	348	1	−1
1NMB	N	349	0	−1
1NMB	N	350	0	−1
1NMB	N	351	0	−1
1NMB	N	352	0	−1
1NMB	N	353	0	−1
1NMB	N	354	0	−1
1NMB	N	355	0	−1
1NMB	N	356	0	−1
1NMB	N	357	1	−1
1NMB	N	358	1	−1
1NMB	N	359	1	−1
1NMB	N	360	0	−1
1NMB	N	361	0	−1
1NMB	N	362	0	−1
1NMB	N	363	0	−1
1NMB	N	364	0	−1
1NMB	N	365	0	−1
1NMB	N	366	0	−1
1NMB	N	367	1	1
1NMB	N	368	1	1
1NMB	N	369	1	1
1NMB	N	370	1	1
1NMB	N	371	0	−1
1NMB	N	372	1	−1
1NMB	N	373	0	−1
1NMB	N	374	0	−1
1NMB	N	375	0	−1
1NMB	N	376	0	−1
1NMB	N	377	0	−1
1NMB	N	378	0	−1
1NMB	N	379	0	−1
1NMB	N	380	0	−1
1NMB	N	381	1	−1
1NMB	N	382	1	−1
1NMB	N	383	0	−1

1NMB	N	384	0	−1
1NMB	N	385	1	−1
1NMB	N	386	1	−1
1NMB	N	387	1	−1
1NMB	N	388	1	−1
1NMB	N	389	0	−1
1NMB	N	390	1	−1
1NMB	N	391	1	−1
1NMB	N	392	1	−1
1NMB	N	393	1	−1
1NMB	N	394	1	−1
1NMB	N	395	0	−1
1NMB	N	396	1	−1
1NMB	N	397	0	−1
1NMB	N	398	0	−1
1NMB	N	399	1	−1
1NMB	N	400	1	1
1NMB	N	401	1	1
1NMB	N	402	1	−1
1NMB	N	403	1	1
1NMB	N	404	0	−1
1NMB	N	405	0	−1
1NMB	N	406	0	−1
1NMB	N	407	0	−1
1NMB	N	408	0	−1
1NMB	N	409	0	−1
1NMB	N	410	0	−1
1NMB	N	411	0	−1
1NMB	N	412	1	−1
1NMB	N	413	0	−1
1NMB	N	414	1	−1
1NMB	N	415	1	−1
1NMB	N	416	1	−1
1NMB	N	417	1	−1
1NMB	N	418	1	−1
1NMB	N	419	1	−1
1NMB	N	420	0	−1
1NMB	N	421	1	−1
1NMB	N	422	0	−1
1NMB	N	423	0	−1
1NMB	N	424	0	−1
1NMB	N	425	0	−1
1NMB	N	426	0	−1
1NMB	N	427	0	−1
1NMB	N	428	0	−1
1NMB	N	429	0	−1
1NMB	N	430	0	−1
1NMB	N	431	0	−1
1NMB	N	432	0	−1
1NMB	N	433	1	−1
1NMB	N	434	1	1
1NMB	N	435	0	−1

1NMB	N	436	1	−1
1NMB	N	437	1	−1
1NMB	N	438	0	−1
1NMB	N	439	1	−1
1NMB	N	440	0	−1
1NMB	N	441	0	−1
1NMB	N	442	0	−1
1NMB	N	443	0	−1
1NMB	N	444	0	−1
1NMB	N	445	0	−1
1NMB	N	446	0	−1
1NMB	N	447	0	−1
1NMB	N	448	0	−1
1NMB	N	449	0	−1
1NMB	N	450	0	−1
1NMB	N	451	0	−1
1NMB	N	452	1	−1
1NMB	N	453	1	−1
1NMB	N	454	1	−1
1NMB	N	455	1	−1
1NMB	N	456	1	−1
1NMB	N	457	1	−1
1NMB	N	458	1	−1
1NMB	N	459	1	−1
1NMB	N	460	0	−1
1NMB	N	461	1	−1
1NMB	N	462	0	−1
1NMB	N	463	1	−1
1NMB	N	464	0	−1
1NMB	N	465	1	−1
1NMB	N	466	0	−1
1NMB	N	467	1	−1
1NMB	N	468	1	−1
1NMB	N	469	0	−1
1NMB	N	470	1	−1
1NSN	L	1	1	1
1NSN	L	2	0	−1
1NSN	L	3	1	−1
1NSN	L	4	0	−1
1NSN	L	5	1	−1
1NSN	L	6	0	−1
1NSN	L	7	1	−1
1NSN	L	8	1	−1
1NSN	L	9	1	−1
1NSN	L	10	1	−1
1NSN	L	11	0	−1
1NSN	L	12	1	−1
1NSN	L	13	0	−1
1NSN	L	14	1	−1
1NSN	L	15	1	−1
1NSN	L	16	1	−1
1NSN	L	17	1	−1

1NSN	L	18	1	−1
1NSN	L	19	0	−1
1NSN	L	20	1	−1
1NSN	L	21	0	−1
1NSN	L	22	1	−1
1NSN	L	23	0	−1
1NSN	L	24	1	−1
1NSN	L	25	0	−1
1NSN	L	26	1	−1
1NSN	L	27	1	−1
1NSN	L	28	1	−1
1NSN	L	29	0	−1
1NSN	L	30	0	−1
1NSN	L	31	1	1
1NSN	L	32	1	1
1NSN	L	33	1	1
1NSN	L	34	1	1
1NSN	L	35	1	−1
1NSN	L	36	0	−1
1NSN	L	37	0	−1
1NSN	L	38	0	−1
1NSN	L	39	0	−1
1NSN	L	40	0	−1
1NSN	L	41	0	−1
1NSN	L	42	0	−1
1NSN	L	43	0	−1
1NSN	L	44	1	−1
1NSN	L	45	1	−1
1NSN	L	46	1	−1
1NSN	L	47	0	−1
1NSN	L	48	0	−1
1NSN	L	49	1	−1
1NSN	L	50	0	−1
1NSN	L	51	0	−1
1NSN	L	52	0	−1
1NSN	L	53	1	1
1NSN	L	54	1	1
1NSN	L	55	0	−1
1NSN	L	56	1	−1
1NSN	L	57	1	1
1NSN	L	58	1	−1
1NSN	L	59	0	−1
1NSN	L	60	1	−1
1NSN	L	61	1	−1
1NSN	L	62	0	−1
1NSN	L	63	1	−1
1NSN	L	64	1	−1
1NSN	L	65	0	−1
1NSN	L	66	0	−1
1NSN	L	67	1	−1
1NSN	L	68	0	−1
1NSN	L	69	1	−1

1NSN	L	70	0	−1
1NSN	L	71	1	−1
1NSN	L	72	1	−1
1NSN	L	73	1	−1
1NSN	L	74	1	−1
1NSN	L	75	0	−1
1NSN	L	76	0	−1
1NSN	L	77	0	−1
1NSN	L	78	0	−1
1NSN	L	79	0	−1
1NSN	L	80	1	−1
1NSN	L	81	1	−1
1NSN	L	82	0	−1
1NSN	L	83	1	−1
1NSN	L	84	0	−1
1NSN	L	85	1	−1
1NSN	L	86	0	−1
1NSN	L	87	0	−1
1NSN	L	88	0	−1
1NSN	L	89	0	−1
1NSN	L	90	0	−1
1NSN	L	91	0	−1
1NSN	L	92	0	−1
1NSN	L	93	0	−1
1NSN	L	94	0	−1
1NSN	L	95	0	−1
1NSN	L	96	1	1
1NSN	L	97	1	1
1NSN	L	98	1	1
1NSN	L	99	0	−1
1NSN	L	100	1	1
1NSN	L	101	0	−1
1NSN	L	102	0	−1
1NSN	L	103	0	−1
1NSN	L	104	1	−1
1NSN	L	105	0	−1
1NSN	L	106	0	−1
1NSN	L	107	1	−1
1NSN	L	108	0	−1
1NSN	L	109	0	−1
1NSN	L	110	0	−1
1NSN	L	111	1	−1
1NSN	L	112	0	−1
1NSN	L	113	1	−1
1NSN	L	114	1	−1
1NSN	L	115	1	−1
1NSN	L	116	1	−1
1NSN	L	117	0	−1
1NSN	L	118	1	−1
1NSN	L	119	0	−1
1NSN	L	120	0	−1
1NSN	L	121	0	−1

1NSN	L	122	0	−1
1NSN	L	123	0	−1
1NSN	L	124	0	−1
1NSN	L	125	0	−1
1NSN	L	126	1	−1
1NSN	L	127	1	−1
1NSN	L	128	0	−1
1NSN	L	129	0	−1
1NSN	L	130	1	−1
1NSN	L	131	1	−1
1NSN	L	132	1	−1
1NSN	L	133	0	−1
1NSN	L	134	0	−1
1NSN	L	135	0	−1
1NSN	L	136	0	−1
1NSN	L	137	0	−1
1NSN	L	138	0	−1
1NSN	L	139	0	−1
1NSN	L	140	0	−1
1NSN	L	141	0	−1
1NSN	L	142	1	−1
1NSN	L	143	0	−1
1NSN	L	144	0	−1
1NSN	L	145	0	−1
1NSN	L	146	1	−1
1NSN	L	147	1	−1
1NSN	L	148	0	−1
1NSN	L	149	1	−1
1NSN	L	150	0	−1
1NSN	L	151	1	−1
1NSN	L	152	0	−1
1NSN	L	153	0	−1
1NSN	L	154	0	−1
1NSN	L	155	1	−1
1NSN	L	156	1	−1
1NSN	L	157	1	−1
1NSN	L	158	1	−1
1NSN	L	159	0	−1
1NSN	L	160	1	−1
1NSN	L	161	1	−1
1NSN	L	162	1	−1
1NSN	L	163	1	−1
1NSN	L	164	0	−1
1NSN	L	165	1	−1
1NSN	L	166	0	−1
1NSN	L	167	1	−1
1NSN	L	168	0	−1
1NSN	L	169	1	−1
1NSN	L	170	0	−1
1NSN	L	171	1	−1
1NSN	L	172	1	−1
1NSN	L	173	1	−1

1NSN	L	174	1	−1
1NSN	L	175	0	−1
1NSN	L	176	0	−1
1NSN	L	177	0	−1
1NSN	L	178	0	−1
1NSN	L	179	0	−1
1NSN	L	180	0	−1
1NSN	L	181	0	−1
1NSN	L	182	0	−1
1NSN	L	183	0	−1
1NSN	L	184	1	−1
1NSN	L	185	0	−1
1NSN	L	186	1	−1
1NSN	L	187	1	−1
1NSN	L	188	1	−1
1NSN	L	189	0	−1
1NSN	L	190	0	−1
1NSN	L	191	1	−1
1NSN	L	192	1	−1
1NSN	L	193	0	−1
1NSN	L	194	1	−1
1NSN	L	195	1	−1
1NSN	L	196	0	−1
1NSN	L	197	0	−1
1NSN	L	198	0	−1
1NSN	L	199	0	−1
1NSN	L	200	0	−1
1NSN	L	201	1	−1
1NSN	L	202	0	−1
1NSN	L	203	1	−1
1NSN	L	204	0	−1
1NSN	L	205	1	−1
1NSN	L	206	1	−1
1NSN	L	207	1	−1
1NSN	L	208	1	−1
1NSN	L	209	0	−1
1NSN	L	210	1	−1
1NSN	L	211	1	−1
1NSN	L	212	1	−1
1NSN	L	213	0	−1
1NSN	L	214	1	−1
1NSN	L	215	0	−1
1NSN	L	216	1	−1
1NSN	L	217	1	−1
1NSN	H	1	1	1
1NSN	H	2	1	−1
1NSN	H	3	1	−1
1NSN	H	4	0	−1
1NSN	H	5	1	−1
1NSN	H	6	0	−1
1NSN	H	7	1	−1
1NSN	H	8	1	−1

1NSN	H	9	1	−1
1NSN	H	10	0	−1
1NSN	H	11	0	−1
1NSN	H	12	0	−1
1NSN	H	13	1	−1
1NSN	H	14	1	−1
1NSN	H	15	1	−1
1NSN	H	16	1	−1
1NSN	H	17	1	−1
1NSN	H	18	0	−1
1NSN	H	19	1	−1
1NSN	H	20	0	−1
1NSN	H	21	0	−1
1NSN	H	22	0	−1
1NSN	H	23	1	−1
1NSN	H	24	0	−1
1NSN	H	25	1	−1
1NSN	H	26	1	−1
1NSN	H	27	1	1
1NSN	H	28	1	−1
1NSN	H	29	0	−1
1NSN	H	30	1	1
1NSN	H	31	1	1
1NSN	H	32	0	−1
1NSN	H	33	0	−1
1NSN	H	34	0	−1
1NSN	H	35	0	−1
1NSN	H	36	0	−1
1NSN	H	37	0	−1
1NSN	H	38	0	−1
1NSN	H	39	0	−1
1NSN	H	40	0	−1
1NSN	H	41	1	−1
1NSN	H	42	1	−1
1NSN	H	43	1	−1
1NSN	H	44	1	−1
1NSN	H	45	1	−1
1NSN	H	46	0	−1
1NSN	H	47	1	−1
1NSN	H	48	0	−1
1NSN	H	49	0	−1
1NSN	H	50	0	−1
1NSN	H	51	1	1
1NSN	H	52	0	−1
1NSN	H	53	1	1
1NSN	H	54	1	1
1NSN	H	55	1	1
1NSN	H	56	1	−1
1NSN	H	57	1	1
1NSN	H	58	0	−1
1NSN	H	59	1	1
1NSN	H	60	1	−1

1NSN	H	61	0	−1
1NSN	H	62	1	−1
1NSN	H	63	1	−1
1NSN	H	64	1	−1
1NSN	H	65	1	−1
1NSN	H	66	1	−1
1NSN	H	67	1	−1
1NSN	H	68	0	−1
1NSN	H	69	0	−1
1NSN	H	70	0	−1
1NSN	H	71	1	−1
1NSN	H	72	1	−1
1NSN	H	73	1	−1
1NSN	H	74	0	−1
1NSN	H	75	1	−1
1NSN	H	76	1	−1
1NSN	H	77	0	−1
1NSN	H	78	0	−1
1NSN	H	79	0	−1
1NSN	H	80	0	−1
1NSN	H	81	0	−1
1NSN	H	82	1	−1
1NSN	H	83	0	−1
1NSN	H	84	1	−1
1NSN	H	85	1	−1
1NSN	H	86	0	−1
1NSN	H	87	1	−1
1NSN	H	88	1	−1
1NSN	H	89	1	−1
1NSN	H	90	0	−1
1NSN	H	91	0	−1
1NSN	H	92	0	−1
1NSN	H	93	0	−1
1NSN	H	94	0	−1
1NSN	H	95	0	−1
1NSN	H	96	0	−1
1NSN	H	97	0	−1
1NSN	H	98	0	−1
1NSN	H	99	1	1
1NSN	H	100	1	1
1NSN	H	101	0	−1
1NSN	H	102	1	−1
1NSN	H	103	0	−1
1NSN	H	104	0	−1
1NSN	H	105	1	−1
1NSN	H	106	0	−1
1NSN	H	107	0	−1
1NSN	H	108	1	−1
1NSN	H	109	0	−1
1NSN	H	110	0	−1
1NSN	H	111	0	−1
1NSN	H	112	1	−1

1NSN	H	113	1	−1
1NSN	H	114	0	−1
1NSN	H	115	1	−1
1NSN	H	116	1	−1
1NSN	H	117	1	−1
1NSN	H	118	1	−1
1NSN	H	119	0	−1
1NSN	H	120	1	−1
1NSN	H	121	0	−1
1NSN	H	122	0	−1
1NSN	H	123	0	−1
1NSN	H	124	0	−1
1NSN	H	125	1	−1
1NSN	H	126	1	−1
1NSN	H	127	1	−1
1NSN	H	128	1	−1
1NSN	H	129	1	−1
1NSN	H	130	1	−1
1NSN	H	131	1	−1
1NSN	H	132	1	−1
1NSN	H	133	1	−1
1NSN	H	134	1	−1
1NSN	H	135	1	−1
1NSN	H	136	0	−1
1NSN	H	137	0	−1
1NSN	H	138	0	−1
1NSN	H	139	0	−1
1NSN	H	140	0	−1
1NSN	H	141	0	−1
1NSN	H	142	0	−1
1NSN	H	143	0	−1
1NSN	H	144	0	−1
1NSN	H	145	0	−1
1NSN	H	146	0	−1
1NSN	H	147	0	−1
1NSN	H	148	1	−1
1NSN	H	149	1	−1
1NSN	H	150	0	−1
1NSN	H	151	1	−1
1NSN	H	152	0	−1
1NSN	H	153	1	−1
1NSN	H	154	0	−1
1NSN	H	155	1	−1
1NSN	H	156	1	−1
1NSN	H	157	1	−1
1NSN	H	158	1	−1
1NSN	H	159	0	−1
1NSN	H	160	1	−1
1NSN	H	161	1	−1
1NSN	H	162	1	−1
1NSN	H	163	1	−1
1NSN	H	164	0	−1

1NSN	H	165	1	−1
1NSN	H	166	0	−1
1NSN	H	167	1	−1
1NSN	H	168	0	−1
1NSN	H	169	1	−1
1NSN	H	170	1	−1
1NSN	H	171	0	−1
1NSN	H	172	1	−1
1NSN	H	173	1	−1
1NSN	H	174	1	−1
1NSN	H	175	0	−1
1NSN	H	176	0	−1
1NSN	H	177	0	−1
1NSN	H	178	0	−1
1NSN	H	179	0	−1
1NSN	H	180	0	−1
1NSN	H	181	0	−1
1NSN	H	182	0	−1
1NSN	H	183	0	−1
1NSN	H	184	1	−1
1NSN	H	185	0	−1
1NSN	H	186	1	−1
1NSN	H	187	0	−1
1NSN	H	188	0	−1
1NSN	H	189	1	−1
1NSN	H	190	1	−1
1NSN	H	191	1	−1
1NSN	H	192	1	−1
1NSN	H	193	0	−1
1NSN	H	194	1	−1
1NSN	H	195	0	−1
1NSN	H	196	0	−1
1NSN	H	197	0	−1
1NSN	H	198	1	−1
1NSN	H	199	0	−1
1NSN	H	200	1	−1
1NSN	H	201	1	−1
1NSN	H	202	0	−1
1NSN	H	203	1	−1
1NSN	H	204	0	−1
1NSN	H	205	1	−1
1NSN	H	206	1	−1
1NSN	H	207	1	−1
1NSN	H	208	0	−1
1NSN	H	209	1	−1
1NSN	H	210	0	−1
1NSN	S	4	1	−1
1NSN	S	5	1	−1
1NSN	S	6	1	−1
1NSN	S	7	1	−1
1NSN	S	8	1	−1
1NSN	S	9	1	1

1NSN	S	10	0	−1
1NSN	S	11	1	−1
1NSN	S	12	0	−1
1NSN	S	13	1	−1
1NSN	S	14	1	−1
1NSN	S	15	1	−1
1NSN	S	16	1	−1
1NSN	S	17	0	−1
1NSN	S	18	1	−1
1NSN	S	19	0	−1
1NSN	S	20	0	−1
1NSN	S	21	0	−1
1NSN	S	22	0	−1
1NSN	S	23	0	−1
1NSN	S	24	0	−1
1NSN	S	25	0	−1
1NSN	S	26	1	−1
1NSN	S	27	0	−1
1NSN	S	28	1	−1
1NSN	S	29	1	−1
1NSN	S	30	1	−1
1NSN	S	31	1	−1
1NSN	S	32	0	−1
1NSN	S	33	1	−1
1NSN	S	34	0	−1
1NSN	S	35	0	−1
1NSN	S	36	0	−1
1NSN	S	37	0	−1
1NSN	S	38	0	−1
1NSN	S	39	0	−1
1NSN	S	40	0	−1
1NSN	S	41	0	−1
1NSN	S	42	0	−1
1NSN	S	43	0	−1
1NSN	S	44	1	−1
1NSN	S	45	1	−1
1NSN	S	46	1	−1
1NSN	S	47	1	−1
1NSN	S	48	1	−1
1NSN	S	49	1	−1
1NSN	S	50	1	−1
1NSN	S	51	1	−1
1NSN	S	52	1	−1
1NSN	S	53	1	−1
1NSN	S	54	0	−1
1NSN	S	55	0	−1
1NSN	S	56	1	1
1NSN	S	57	1	1
1NSN	S	58	0	−1
1NSN	S	59	0	−1
1NSN	S	60	1	1
1NSN	S	61	1	1

1NSN	S	62	0	−1
1NSN	S	63	1	−1
1NSN	S	64	1	1
1NSN	S	65	0	−1
1NSN	S	66	0	−1
1NSN	S	67	1	−1
1NSN	S	68	1	1
1NSN	S	69	0	−1
1NSN	S	70	1	1
1NSN	S	71	1	−1
1NSN	S	72	0	−1
1NSN	S	73	1	1
1NSN	S	74	0	−1
1NSN	S	75	0	−1
1NSN	S	76	1	−1
1NSN	S	77	0	−1
1NSN	S	78	1	−1
1NSN	S	79	0	−1
1NSN	S	80	1	−1
1NSN	S	81	0	−1
1NSN	S	82	1	−1
1NSN	S	83	1	−1
1NSN	S	84	1	−1
1NSN	S	85	1	−1
1NSN	S	86	1	−1
1NSN	S	87	0	−1
1NSN	S	88	0	−1
1NSN	S	89	0	−1
1NSN	S	90	0	−1
1NSN	S	91	0	−1
1NSN	S	92	0	−1
1NSN	S	93	0	−1
1NSN	S	94	0	−1
1NSN	S	95	1	1
1NSN	S	96	1	1
1NSN	S	97	1	1
1NSN	S	98	0	−1
1NSN	S	99	0	−1
1NSN	S	100	0	−1
1NSN	S	101	0	−1
1NSN	S	102	0	−1
1NSN	S	103	0	−1
1NSN	S	104	0	−1
1NSN	S	105	1	1
1NSN	S	106	1	1
1NSN	S	107	0	−1
1NSN	S	108	0	−1
1NSN	S	109	0	−1
1NSN	S	110	1	−1
1NSN	S	111	0	−1
1NSN	S	112	0	−1
1NSN	S	113	1	−1

1NSN	S	114	1	−1
1NSN	S	115	1	−1
1NSN	S	116	1	−1
1NSN	S	117	1	−1
1NSN	S	118	0	−1
1NSN	S	119	1	−1
1NSN	S	120	1	1
1NSN	S	121	0	−1
1NSN	S	122	0	−1
1NSN	S	123	1	1
1NSN	S	124	1	1
1NSN	S	125	0	−1
1NSN	S	126	1	−1
1NSN	S	127	1	1
1NSN	S	128	0	−1
1NSN	S	129	0	−1
1NSN	S	130	1	−1
1NSN	S	131	1	1
1NSN	S	132	0	−1
1NSN	S	133	1	−1
1NSN	S	134	1	1
1NSN	S	135	1	1
1NSN	S	136	1	−1
1NSN	S	137	0	−1
1NSN	S	138	1	1
1NSN	S	139	1	−1
1NSN	S	140	1	−1
1NSN	S	141	1	−1
1OSP	L	1	1	−1
1OSP	L	2	0	−1
1OSP	L	3	1	−1
1OSP	L	4	0	−1
1OSP	L	5	1	−1
1OSP	L	6	0	−1
1OSP	L	7	1	−1
1OSP	L	8	0	−1
1OSP	L	9	1	−1
1OSP	L	10	1	−1
1OSP	L	11	1	−1
1OSP	L	12	1	−1
1OSP	L	13	0	−1
1OSP	L	14	1	−1
1OSP	L	15	1	−1
1OSP	L	16	1	−1
1OSP	L	17	1	−1
1OSP	L	18	1	−1
1OSP	L	19	0	−1
1OSP	L	20	1	−1
1OSP	L	21	0	−1
1OSP	L	22	1	−1
1OSP	L	23	0	−1
1OSP	L	24	1	−1

1OSP	L	25	0	−1
1OSP	L	26	1	−1
1OSP	L	27	1	−1
1OSP	L	28	1	1
1OSP	L	29	0	−1
1OSP	L	30	1	1
1OSP	L	31	0	−1
1OSP	L	32	1	1
1OSP	L	33	0	−1
1OSP	L	34	0	−1
1OSP	L	35	0	−1
1OSP	L	36	0	−1
1OSP	L	37	0	−1
1OSP	L	38	0	−1
1OSP	L	39	1	−1
1OSP	L	40	1	−1
1OSP	L	41	1	−1
1OSP	L	42	1	−1
1OSP	L	43	0	−1
1OSP	L	44	0	−1
1OSP	L	45	1	−1
1OSP	L	46	0	−1
1OSP	L	47	0	−1
1OSP	L	48	0	−1
1OSP	L	49	0	−1
1OSP	L	50	1	1
1OSP	L	51	0	−1
1OSP	L	52	1	1
1OSP	L	53	1	1
1OSP	L	54	1	−1
1OSP	L	55	0	−1
1OSP	L	56	1	−1
1OSP	L	57	1	−1
1OSP	L	58	0	−1
1OSP	L	59	0	−1
1OSP	L	60	1	−1
1OSP	L	61	0	−1
1OSP	L	62	0	−1
1OSP	L	63	1	−1
1OSP	L	64	0	−1
1OSP	L	65	1	−1
1OSP	L	66	1	1
1OSP	L	67	1	−1
1OSP	L	68	1	−1
1OSP	L	69	1	−1
1OSP	L	70	0	−1
1OSP	L	71	0	−1
1OSP	L	72	0	−1
1OSP	L	73	0	−1
1OSP	L	74	0	−1
1OSP	L	75	0	−1
1OSP	L	76	1	−1

1OSP	L	77	1	−1
1OSP	L	78	0	−1
1OSP	L	79	1	−1
1OSP	L	80	1	−1
1OSP	L	81	1	−1
1OSP	L	82	0	−1
1OSP	L	83	0	−1
1OSP	L	84	0	−1
1OSP	L	85	0	−1
1OSP	L	86	0	−1
1OSP	L	87	0	−1
1OSP	L	88	0	−1
1OSP	L	89	0	−1
1OSP	L	90	0	−1
1OSP	L	91	0	−1
1OSP	L	92	1	1
1OSP	L	93	1	−1
1OSP	L	94	1	−1
1OSP	L	95	0	−1
1OSP	L	96	0	−1
1OSP	L	97	0	−1
1OSP	L	98	0	−1
1OSP	L	99	0	−1
1OSP	L	100	1	−1
1OSP	L	101	0	−1
1OSP	L	102	0	−1
1OSP	L	103	1	−1
1OSP	L	104	0	−1
1OSP	L	105	0	−1
1OSP	L	106	0	−1
1OSP	L	107	1	−1
1OSP	L	108	1	−1
1OSP	L	109	1	−1
1OSP	L	110	1	−1
1OSP	L	111	0	−1
1OSP	L	112	1	−1
1OSP	L	113	0	−1
1OSP	L	114	1	−1
1OSP	L	115	0	−1
1OSP	L	116	0	−1
1OSP	L	117	0	−1
1OSP	L	118	0	−1
1OSP	L	119	0	−1
1OSP	L	120	0	−1
1OSP	L	121	0	−1
1OSP	L	122	1	−1
1OSP	L	123	1	−1
1OSP	L	124	0	−1
1OSP	L	125	0	−1
1OSP	L	126	1	−1
1OSP	L	127	1	−1
1OSP	L	128	1	−1

1OSP	L	129	0	−1
1OSP	L	130	0	−1
1OSP	L	131	0	−1
1OSP	L	132	0	−1
1OSP	L	133	0	−1
1OSP	L	134	0	−1
1OSP	L	135	0	−1
1OSP	L	136	0	−1
1OSP	L	137	0	−1
1OSP	L	138	1	−1
1OSP	L	139	0	−1
1OSP	L	140	0	−1
1OSP	L	141	0	−1
1OSP	L	142	1	−1
1OSP	L	143	1	−1
1OSP	L	144	0	−1
1OSP	L	145	1	−1
1OSP	L	146	0	−1
1OSP	L	147	1	−1
1OSP	L	148	0	−1
1OSP	L	149	0	−1
1OSP	L	150	0	−1
1OSP	L	151	1	−1
1OSP	L	152	1	−1
1OSP	L	153	1	−1
1OSP	L	154	1	−1
1OSP	L	155	0	−1
1OSP	L	156	1	−1
1OSP	L	157	1	−1
1OSP	L	158	1	−1
1OSP	L	159	1	−1
1OSP	L	160	0	−1
1OSP	L	161	1	−1
1OSP	L	162	0	−1
1OSP	L	163	1	−1
1OSP	L	164	0	−1
1OSP	L	165	1	−1
1OSP	L	166	0	−1
1OSP	L	167	0	−1
1OSP	L	168	1	−1
1OSP	L	169	1	−1
1OSP	L	170	1	−1
1OSP	L	171	0	−1
1OSP	L	172	0	−1
1OSP	L	173	0	−1
1OSP	L	174	0	−1
1OSP	L	175	0	−1
1OSP	L	176	0	−1
1OSP	L	177	0	−1
1OSP	L	178	0	−1
1OSP	L	179	0	−1
1OSP	L	180	1	−1

1OSP	L	181	0	−1
1OSP	L	182	1	−1
1OSP	L	183	1	−1
1OSP	L	184	1	−1
1OSP	L	185	0	−1
1OSP	L	186	0	−1
1OSP	L	187	1	−1
1OSP	L	188	1	−1
1OSP	L	189	1	−1
1OSP	L	190	1	−1
1OSP	L	191	0	−1
1OSP	L	192	0	−1
1OSP	L	193	0	−1
1OSP	L	194	0	−1
1OSP	L	195	0	−1
1OSP	L	196	0	−1
1OSP	L	197	1	−1
1OSP	L	198	0	−1
1OSP	L	199	1	−1
1OSP	L	200	1	−1
1OSP	L	201	1	−1
1OSP	L	202	1	−1
1OSP	L	203	1	−1
1OSP	L	204	1	−1
1OSP	L	205	1	−1
1OSP	L	206	1	−1
1OSP	L	207	1	−1
1OSP	L	208	1	−1
1OSP	L	209	0	−1
1OSP	L	210	1	−1
1OSP	L	211	1	−1
1OSP	L	212	1	−1
1OSP	L	213	1	−1
1OSP	L	214	1	−1
1OSP	H	1	1	−1
1OSP	H	2	0	−1
1OSP	H	3	1	−1
1OSP	H	4	0	−1
1OSP	H	5	1	−1
1OSP	H	6	0	−1
1OSP	H	7	1	−1
1OSP	H	8	1	−1
1OSP	H	9	1	−1
1OSP	H	10	1	−1
1OSP	H	11	0	−1
1OSP	H	12	0	−1
1OSP	H	13	1	−1
1OSP	H	14	1	−1
1OSP	H	15	1	−1
1OSP	H	16	1	−1
1OSP	H	17	1	−1
1OSP	H	18	0	−1

1OSP	H	19	1	−1
1OSP	H	20	0	−1
1OSP	H	21	0	−1
1OSP	H	22	0	−1
1OSP	H	23	0	−1
1OSP	H	24	0	−1
1OSP	H	25	1	−1
1OSP	H	26	1	−1
1OSP	H	27	0	−1
1OSP	H	28	1	−1
1OSP	H	29	0	−1
1OSP	H	30	1	−1
1OSP	H	31	1	1
1OSP	H	32	0	−1
1OSP	H	33	0	−1
1OSP	H	34	0	−1
1OSP	H	35	0	−1
1OSP	H	36	0	−1
1OSP	H	37	0	−1
1OSP	H	38	0	−1
1OSP	H	39	0	−1
1OSP	H	40	0	−1
1OSP	H	41	1	−1
1OSP	H	42	1	−1
1OSP	H	43	1	−1
1OSP	H	44	1	−1
1OSP	H	45	0	−1
1OSP	H	46	0	−1
1OSP	H	47	0	−1
1OSP	H	48	0	−1
1OSP	H	49	0	−1
1OSP	H	50	0	−1
1OSP	H	51	0	−1
1OSP	H	52	1	−1
1OSP	H	53	1	1
1OSP	H	54	1	−1
1OSP	H	55	1	−1
1OSP	H	56	1	−1
1OSP	H	57	1	−1
1OSP	H	58	1	−1
1OSP	H	59	1	−1
1OSP	H	60	0	−1
1OSP	H	61	1	−1
1OSP	H	62	1	−1
1OSP	H	63	0	−1
1OSP	H	64	1	−1
1OSP	H	65	0	−1
1OSP	H	66	1	−1
1OSP	H	67	0	−1
1OSP	H	68	1	−1
1OSP	H	69	0	−1
1OSP	H	70	1	−1

1OSP	H	71	1	−1
1OSP	H	72	1	−1
1OSP	H	73	1	−1
1OSP	H	74	1	−1
1OSP	H	75	1	−1
1OSP	H	76	0	−1
1OSP	H	77	1	−1
1OSP	H	78	0	−1
1OSP	H	79	0	−1
1OSP	H	80	0	−1
1OSP	H	81	1	−1
1OSP	H	82	0	−1
1OSP	H	83	1	−1
1OSP	H	84	1	−1
1OSP	H	85	0	−1
1OSP	H	86	1	−1
1OSP	H	87	1	−1
1OSP	H	88	1	−1
1OSP	H	89	0	−1
1OSP	H	90	0	−1
1OSP	H	91	0	−1
1OSP	H	92	0	−1
1OSP	H	93	0	−1
1OSP	H	94	0	−1
1OSP	H	95	0	−1
1OSP	H	96	0	−1
1OSP	H	97	0	−1
1OSP	H	98	0	−1
1OSP	H	99	1	1
1OSP	H	100	1	1
1OSP	H	101	1	1
1OSP	H	102	1	1
1OSP	H	103	1	1
1OSP	H	104	1	−1
1OSP	H	105	0	−1
1OSP	H	106	0	−1
1OSP	H	107	0	−1
1OSP	H	108	0	−1
1OSP	H	109	0	−1
1OSP	H	110	0	−1
1OSP	H	111	0	−1
1OSP	H	112	1	−1
1OSP	H	113	0	−1
1OSP	H	114	0	−1
1OSP	H	115	1	−1
1OSP	H	116	0	−1
1OSP	H	117	0	−1
1OSP	H	118	0	−1
1OSP	H	119	0	−1
1OSP	H	120	1	−1
1OSP	H	121	1	−1
1OSP	H	122	1	−1

1OSP	H	123	1	−1
1OSP	H	124	1	−1
1OSP	H	125	1	−1
1OSP	H	126	0	−1
1OSP	H	127	1	−1
1OSP	H	128	0	−1
1OSP	H	129	0	−1
1OSP	H	130	0	−1
1OSP	H	131	0	−1
1OSP	H	132	0	−1
1OSP	H	133	1	−1
1OSP	H	134	0	−1
1OSP	H	135	1	−1
1OSP	H	136	1	−1
1OSP	H	137	1	−1
1OSP	H	138	1	−1
1OSP	H	139	1	−1
1OSP	H	140	1	−1
1OSP	H	141	1	−1
1OSP	H	142	1	−1
1OSP	H	143	0	−1
1OSP	H	144	0	−1
1OSP	H	145	0	−1
1OSP	H	146	0	−1
1OSP	H	147	0	−1
1OSP	H	148	0	−1
1OSP	H	149	0	−1
1OSP	H	150	0	−1
1OSP	H	151	0	−1
1OSP	H	152	0	−1
1OSP	H	153	0	−1
1OSP	H	154	0	−1
1OSP	H	155	0	−1
1OSP	H	156	1	−1
1OSP	H	157	0	−1
1OSP	H	158	1	−1
1OSP	H	159	0	−1
1OSP	H	160	1	−1
1OSP	H	161	0	−1
1OSP	H	162	1	−1
1OSP	H	163	1	−1
1OSP	H	164	1	−1
1OSP	H	165	0	−1
1OSP	H	166	1	−1
1OSP	H	167	1	−1
1OSP	H	168	1	−1
1OSP	H	169	1	−1
1OSP	H	170	1	−1
1OSP	H	171	0	−1
1OSP	H	172	1	−1
1OSP	H	173	0	−1
1OSP	H	174	1	−1

1OSP	H	175	0	−1
1OSP	H	176	1	−1
1OSP	H	177	1	−1
1OSP	H	178	1	−1
1OSP	H	179	1	−1
1OSP	H	180	1	−1
1OSP	H	181	1	−1
1OSP	H	182	0	−1
1OSP	H	183	0	−1
1OSP	H	184	0	−1
1OSP	H	185	0	−1
1OSP	H	186	0	−1
1OSP	H	187	0	−1
1OSP	H	188	0	−1
1OSP	H	189	0	−1
1OSP	H	190	0	−1
1OSP	H	191	1	−1
1OSP	H	192	0	−1
1OSP	H	193	1	−1
1OSP	H	194	0	−1
1OSP	H	195	0	−1
1OSP	H	196	1	−1
1OSP	H	197	1	−1
1OSP	H	198	1	−1
1OSP	H	199	1	−1
1OSP	H	200	0	−1
1OSP	H	201	0	−1
1OSP	H	202	0	−1
1OSP	H	203	0	−1
1OSP	H	204	0	−1
1OSP	H	205	0	−1
1OSP	H	206	0	−1
1OSP	H	207	1	−1
1OSP	H	208	1	−1
1OSP	H	209	0	−1
1OSP	H	210	1	−1
1OSP	H	211	1	−1
1OSP	H	212	1	−1
1OSP	H	213	1	−1
1OSP	H	214	1	−1
1OSP	H	215	0	−1
1OSP	H	216	1	−1
1OSP	H	217	0	−1
1OSP	H	218	1	−1
1OSP	O	7	1	−1
1OSP	O	8	1	−1
1OSP	O	9	1	−1
1OSP	O	10	1	−1
1OSP	O	11	1	−1
1OSP	O	12	1	−1
1OSP	O	13	0	−1
1OSP	O	14	1	1

1OSP	O	15	1	−1
1OSP	O	16	0	−1
1OSP	O	17	1	1
1OSP	O	18	0	−1
1OSP	O	19	0	−1
1OSP	O	20	1	−1
1OSP	O	21	1	−1
1OSP	O	22	0	−1
1OSP	O	23	1	−1
1OSP	O	24	0	−1
1OSP	O	25	0	−1
1OSP	O	26	0	−1
1OSP	O	27	0	−1
1OSP	O	28	1	1
1OSP	O	29	1	1
1OSP	O	30	1	1
1OSP	O	31	0	−1
1OSP	O	32	1	−1
1OSP	O	33	1	−1
1OSP	O	34	1	−1
1OSP	O	35	1	−1
1OSP	O	36	0	−1
1OSP	O	37	1	−1
1OSP	O	38	0	−1
1OSP	O	39	0	−1
1OSP	O	40	0	−1
1OSP	O	41	1	−1
1OSP	O	42	0	−1
1OSP	O	43	1	−1
1OSP	O	44	1	−1
1OSP	O	45	1	−1
1OSP	O	46	1	−1
1OSP	O	47	0	−1
1OSP	O	48	1	−1
1OSP	O	49	0	−1
1OSP	O	50	1	−1
1OSP	O	51	0	−1
1OSP	O	52	1	1
1OSP	O	53	1	1
1OSP	O	54	1	1
1OSP	O	55	1	1
1OSP	O	56	0	−1
1OSP	O	57	0	−1
1OSP	O	58	0	−1
1OSP	O	59	1	−1
1OSP	O	60	0	−1
1OSP	O	61	1	−1
1OSP	O	62	0	−1
1OSP	O	63	1	−1
1OSP	O	64	1	−1
1OSP	O	65	1	−1
1OSP	O	66	1	−1

1OSP	O	67	1	−1
1OSP	O	68	0	−1
1OSP	O	69	1	−1
1OSP	O	70	0	−1
1OSP	O	71	1	−1
1OSP	O	72	0	−1
1OSP	O	73	1	−1
1OSP	O	74	0	−1
1OSP	O	75	0	−1
1OSP	O	76	1	1
1OSP	O	77	1	1
1OSP	O	78	1	1
1OSP	O	79	0	−1
1OSP	O	80	1	−1
1OSP	O	81	0	−1
1OSP	O	82	0	−1
1OSP	O	83	0	−1
1OSP	O	84	0	−1
1OSP	O	85	0	−1
1OSP	O	86	0	−1
1OSP	O	87	1	−1
1OSP	O	88	1	−1
1OSP	O	89	1	−1
1OSP	O	90	1	−1
1OSP	O	91	1	−1
1OSP	O	92	0	−1
1OSP	O	93	0	−1
1OSP	O	94	1	−1
1OSP	O	95	1	−1
1OSP	O	96	1	−1
1OSP	O	97	1	−1
1OSP	O	98	0	−1
1OSP	O	99	0	−1
1OSP	O	100	0	−1
1OSP	O	101	1	1
1OSP	O	102	1	1
1OSP	O	103	1	1
1OSP	O	104	0	−1
1OSP	O	105	0	−1
1OSP	O	106	1	−1
1OSP	O	107	1	−1
1OSP	O	108	0	−1
1OSP	O	109	1	−1
1OSP	O	110	0	−1
1OSP	O	111	1	−1
1OSP	O	112	1	−1
1OSP	O	113	1	−1
1OSP	O	114	1	−1
1OSP	O	115	0	−1
1OSP	O	116	0	−1
1OSP	O	117	0	−1
1OSP	O	118	1	−1

1OSP	O	119	0	−1
1OSP	O	120	1	−1
1OSP	O	121	0	−1
1OSP	O	122	0	−1
1OSP	O	123	0	−1
1OSP	O	124	1	−1
1OSP	O	125	1	−1
1OSP	O	126	1	−1
1OSP	O	127	0	−1
1OSP	O	128	1	−1
1OSP	O	129	0	−1
1OSP	O	130	1	−1
1OSP	O	131	0	−1
1OSP	O	132	1	−1
1OSP	O	133	1	−1
1OSP	O	134	0	−1
1OSP	O	135	1	−1
1OSP	O	136	1	−1
1OSP	O	137	1	−1
1OSP	O	138	1	−1
1OSP	O	139	0	−1
1OSP	O	140	0	−1
1OSP	O	141	1	−1
1OSP	O	142	0	−1
1OSP	O	143	1	−1
1OSP	O	144	0	−1
1OSP	O	145	0	−1
1OSP	O	146	0	−1
1OSP	O	147	1	−1
1OSP	O	148	1	−1
1OSP	O	149	0	−1
1OSP	O	150	1	−1
1OSP	O	151	0	−1
1OSP	O	152	1	−1
1OSP	O	153	0	−1
1OSP	O	154	1	−1
1OSP	O	155	0	−1
1OSP	O	156	1	−1
1OSP	O	157	1	−1
1OSP	O	158	1	−1
1OSP	O	159	0	−1
1OSP	O	160	0	−1
1OSP	O	161	0	−1
1OSP	O	162	0	−1
1OSP	O	163	1	−1
1OSP	O	164	0	−1
1OSP	O	165	1	−1
1OSP	O	166	1	−1
1OSP	O	167	1	−1
1OSP	O	168	1	−1
1OSP	O	169	0	−1
1OSP	O	170	0	−1

1OSP	O	171	0	−1
1OSP	O	172	0	−1
1OSP	O	173	0	−1
1OSP	O	174	0	−1
1OSP	O	175	0	−1
1OSP	O	176	0	−1
1OSP	O	177	1	−1
1OSP	O	178	1	−1
1OSP	O	179	1	−1
1OSP	O	180	1	−1
1OSP	O	181	1	−1
1OSP	O	182	1	−1
1OSP	O	183	0	−1
1OSP	O	184	1	−1
1OSP	O	185	0	−1
1OSP	O	186	1	−1
1OSP	O	187	0	−1
1OSP	O	188	1	−1
1OSP	O	189	0	−1
1OSP	O	190	1	−1
1OSP	O	191	1	−1
1OSP	O	192	1	−1
1OSP	O	193	1	−1
1OSP	O	194	1	−1
1OSP	O	195	1	−1
1OSP	O	196	0	−1
1OSP	O	197	1	−1
1OSP	O	198	1	−1
1OSP	O	199	1	−1
1OSP	O	200	1	−1
1OSP	O	201	1	−1
1OSP	O	202	1	−1
1OSP	O	203	1	−1
1OSP	O	204	1	−1
1OSP	O	205	1	−1
1OSP	O	206	0	−1
1OSP	O	207	0	−1
1OSP	O	208	0	−1
1OSP	O	209	0	−1
1OSP	O	210	0	−1
1OSP	O	211	0	−1
1OSP	O	212	1	−1
1OSP	O	213	1	−1
1OSP	O	214	1	−1
1OSP	O	215	1	−1
1OSP	O	216	1	−1
1OSP	O	217	0	−1
1OSP	O	218	0	−1
1OSP	O	219	0	−1
1OSP	O	220	1	−1
1OSP	O	221	0	−1
1OSP	O	222	0	−1

1OSP	O	223	1	−1
1OSP	O	224	1	−1
1OSP	O	225	0	−1
1OSP	O	226	0	−1
1OSP	O	227	0	−1
1OSP	O	228	0	−1
1OSP	O	229	0	−1
1OSP	O	230	0	−1
1OSP	O	231	1	−1
1OSP	O	232	0	−1
1OSP	O	233	0	−1
1OSP	O	234	1	−1
1OSP	O	235	1	−1
1OSP	O	236	0	−1
1OSP	O	237	1	−1
1OSP	O	238	1	−1
1OSP	O	239	1	−1
1OSP	O	240	1	−1
1OSP	O	241	1	−1
1OSP	O	242	1	−1
1OSP	O	243	1	−1
1OSP	O	244	1	−1
1OSP	O	245	1	−1
1OSP	O	246	0	−1
1OSP	O	247	1	−1
1OSP	O	248	1	−1
1OSP	O	249	1	−1
1OSP	O	250	1	−1
1OSP	O	251	1	−1
1OSP	O	252	0	−1
1OSP	O	253	1	−1
1OSP	O	254	1	−1
1OSP	O	255	0	−1
1OSP	O	256	0	−1
1OSP	O	257	1	−1
1QFU	A	9	1	1
1QFU	A	10	1	1
1QFU	A	11	1	1
1QFU	A	12	1	1
1QFU	A	13	1	1
1QFU	A	14	1	1
1QFU	A	15	1	1
1QFU	A	16	1	1
1QFU	A	17	1	1
1QFU	A	18	1	1
1QFU	A	19	1	1
1QFU	A	20	1	1
1QFU	A	21	1	1
1QFU	A	22	1	−1
1QFU	A	23	1	−1
1QFU	A	24	1	−1
1QFU	A	25	1	−1

1QFU	A	26	0	−1
1QFU	A	27	1	1
1QFU	A	28	1	1
1QFU	A	29	1	1
1QFU	A	30	1	1
1QFU	A	31	1	−1
1QFU	A	32	1	−1
1QFU	A	33	1	−1
1QFU	A	34	0	−1
1QFU	A	35	1	−1
1QFU	A	36	0	−1
1QFU	A	37	0	−1
1QFU	A	38	1	−1
1QFU	A	39	0	−1
1QFU	A	40	1	1
1QFU	A	41	1	−1
1QFU	A	42	0	−1
1QFU	A	43	0	−1
1QFU	A	44	0	−1
1QFU	A	45	1	−1
1QFU	A	46	1	−1
1QFU	A	47	1	−1
1QFU	A	48	1	1
1QFU	A	49	1	1
1QFU	A	50	1	1
1QFU	A	51	0	−1
1QFU	A	52	0	−1
1QFU	A	53	1	1
1QFU	A	54	1	−1
1QFU	A	55	1	−1
1QFU	A	56	0	−1
1QFU	A	57	1	−1
1QFU	A	58	0	−1
1QFU	A	59	0	−1
1QFU	A	60	1	1
1QFU	A	61	0	−1
1QFU	A	62	1	1
1QFU	A	63	1	1
1QFU	A	64	0	−1
1QFU	A	65	0	−1
1QFU	A	66	0	−1
1QFU	A	67	0	−1
1QFU	A	68	0	−1
1QFU	A	69	0	−1
1QFU	A	70	0	−1
1QFU	A	71	0	−1
1QFU	A	72	0	−1
1QFU	A	73	0	−1
1QFU	A	74	0	−1
1QFU	A	75	1	1
1QFU	A	76	0	−1
1QFU	A	77	0	−1

1QFU	A	78	1	1
1QFU	A	79	0	−1
1QFU	A	80	1	−1
1QFU	A	81	1	−1
1QFU	A	82	1	1
1QFU	A	83	1	−1
1QFU	A	84	0	−1
1QFU	A	85	0	−1
1QFU	A	86	0	−1
1QFU	A	87	0	−1
1QFU	A	88	0	−1
1QFU	A	89	0	−1
1QFU	A	90	0	−1
1QFU	A	91	1	−1
1QFU	A	92	1	1
1QFU	A	93	0	−1
1QFU	A	94	1	1
1QFU	A	95	1	1
1QFU	A	96	1	1
1QFU	A	97	0	−1
1QFU	A	98	0	−1
1QFU	A	99	0	−1
1QFU	A	100	0	−1
1QFU	A	101	1	−1
1QFU	A	102	0	−1
1QFU	A	103	1	−1
1QFU	A	104	1	−1
1QFU	A	105	1	−1
1QFU	A	106	1	1
1QFU	A	107	1	−1
1QFU	A	108	0	−1
1QFU	A	109	0	−1
1QFU	A	110	1	1
1QFU	A	111	1	−1
1QFU	A	112	0	−1
1QFU	A	113	0	−1
1QFU	A	114	1	1
1QFU	A	115	0	−1
1QFU	A	116	0	−1
1QFU	A	117	0	−1
1QFU	A	118	0	−1
1QFU	A	119	0	−1
1QFU	A	120	0	−1
1QFU	A	121	1	−1
1QFU	A	122	1	−1
1QFU	A	123	0	−1
1QFU	A	124	1	−1
1QFU	A	125	0	−1
1QFU	A	126	1	−1
1QFU	A	127	0	−1
1QFU	A	128	1	−1
1QFU	A	129	1	−1

1QFU	A	130	0	−1
1QFU	A	131	1	−1
1QFU	A	132	1	−1
1QFU	A	133	1	−1
1QFU	A	134	0	−1
1QFU	A	135	1	−1
1QFU	A	136	0	−1
1QFU	A	137	1	−1
1QFU	A	138	0	−1
1QFU	A	139	0	−1
1QFU	A	140	1	−1
1QFU	A	141	1	1
1QFU	A	142	1	1
1QFU	A	143	1	1
1QFU	A	144	1	−1
1QFU	A	145	1	−1
1QFU	A	146	0	−1
1QFU	A	147	0	−1
1QFU	A	148	0	−1
1QFU	A	149	0	−1
1QFU	A	150	0	−1
1QFU	A	151	0	−1
1QFU	A	152	0	−1
1QFU	A	153	0	−1
1QFU	A	154	0	−1
1QFU	A	155	0	−1
1QFU	A	156	1	−1
1QFU	A	157	1	−1
1QFU	A	158	1	−1
1QFU	A	159	1	−1
1QFU	A	160	1	−1
1QFU	A	161	0	−1
1QFU	A	162	1	−1
1QFU	A	163	1	−1
1QFU	A	164	0	−1
1QFU	A	165	1	−1
1QFU	A	166	0	−1
1QFU	A	167	1	−1
1QFU	A	168	0	−1
1QFU	A	169	1	−1
1QFU	A	170	0	−1
1QFU	A	171	1	−1
1QFU	A	172	1	−1
1QFU	A	173	1	−1
1QFU	A	174	0	−1
1QFU	A	175	1	−1
1QFU	A	176	0	−1
1QFU	A	177	0	−1
1QFU	A	178	0	−1
1QFU	A	179	0	−1
1QFU	A	180	0	−1
1QFU	A	181	0	−1

1QFU	A	182	0	−1
1QFU	A	183	0	−1
1QFU	A	184	0	−1
1QFU	A	185	0	−1
1QFU	A	186	0	−1
1QFU	A	187	1	−1
1QFU	A	188	1	−1
1QFU	A	189	1	−1
1QFU	A	190	0	−1
1QFU	A	191	0	−1
1QFU	A	192	1	−1
1QFU	A	193	1	−1
1QFU	A	194	0	−1
1QFU	A	195	0	−1
1QFU	A	196	1	−1
1QFU	A	197	1	−1
1QFU	A	198	1	−1
1QFU	A	199	1	−1
1QFU	A	200	0	−1
1QFU	A	201	1	−1
1QFU	A	202	0	−1
1QFU	A	203	0	−1
1QFU	A	204	0	−1
1QFU	A	205	0	−1
1QFU	A	206	0	−1
1QFU	A	207	1	−1
1QFU	A	208	1	−1
1QFU	A	209	1	−1
1QFU	A	210	1	−1
1QFU	A	211	1	−1
1QFU	A	212	1	−1
1QFU	A	213	0	−1
1QFU	A	214	1	−1
1QFU	A	215	0	−1
1QFU	A	216	1	−1
1QFU	A	217	1	−1
1QFU	A	218	1	−1
1QFU	A	219	1	−1
1QFU	A	220	1	−1
1QFU	A	221	1	−1
1QFU	A	222	1	−1
1QFU	A	223	1	−1
1QFU	A	224	1	−1
1QFU	A	225	1	−1
1QFU	A	226	0	−1
1QFU	A	227	0	−1
1QFU	A	228	0	−1
1QFU	A	229	0	−1
1QFU	A	230	0	−1
1QFU	A	231	0	−1
1QFU	A	232	0	−1
1QFU	A	233	0	−1

1QFU	A	234	0	−1
1QFU	A	235	0	−1
1QFU	A	236	1	−1
1QFU	A	237	0	−1
1QFU	A	238	1	−1
1QFU	A	239	1	−1
1QFU	A	240	1	−1
1QFU	A	241	0	−1
1QFU	A	242	0	−1
1QFU	A	243	0	−1
1QFU	A	244	1	−1
1QFU	A	245	0	−1
1QFU	A	246	0	−1
1QFU	A	247	0	−1
1QFU	A	248	0	−1
1QFU	A	249	0	−1
1QFU	A	250	0	−1
1QFU	A	251	0	−1
1QFU	A	252	0	−1
1QFU	A	253	0	−1
1QFU	A	254	0	−1
1QFU	A	255	0	−1
1QFU	A	256	0	−1
1QFU	A	257	0	−1
1QFU	A	258	0	−1
1QFU	A	259	0	−1
1QFU	A	260	0	−1
1QFU	A	261	1	−1
1QFU	A	262	1	−1
1QFU	A	263	1	−1
1QFU	A	264	1	1
1QFU	A	265	0	−1
1QFU	A	266	1	1
1QFU	A	267	0	−1
1QFU	A	268	0	−1
1QFU	A	269	1	1
1QFU	A	270	0	−1
1QFU	A	271	1	1
1QFU	A	272	0	−1
1QFU	A	273	1	1
1QFU	A	274	1	1
1QFU	A	275	1	−1
1QFU	A	276	1	1
1QFU	A	277	1	−1
1QFU	A	278	1	−1
1QFU	A	279	0	−1
1QFU	A	280	1	1
1QFU	A	281	0	−1
1QFU	A	282	0	−1
1QFU	A	283	0	−1
1QFU	A	284	0	−1
1QFU	A	285	1	−1

1QFU	A	286	0	−1
1QFU	A	287	0	−1
1QFU	A	288	0	−1
1QFU	A	289	1	−1
1QFU	A	290	1	1
1QFU	A	291	1	1
1QFU	A	292	1	1
1QFU	A	293	1	1
1QFU	A	294	1	1
1QFU	A	295	0	−1
1QFU	A	296	0	−1
1QFU	A	297	0	−1
1QFU	A	298	0	−1
1QFU	A	299	1	1
1QFU	A	300	1	1
1QFU	A	301	1	1
1QFU	A	302	0	−1
1QFU	A	303	1	1
1QFU	A	304	1	1
1QFU	A	305	1	1
1QFU	A	306	0	−1
1QFU	A	307	1	1
1QFU	A	308	1	1
1QFU	A	309	0	−1
1QFU	A	310	1	1
1QFU	A	311	1	1
1QFU	A	312	1	−1
1QFU	A	313	1	−1
1QFU	A	314	1	1
1QFU	A	315	1	1
1QFU	A	316	1	1
1QFU	A	317	1	1
1QFU	A	318	1	1
1QFU	A	319	1	1
1QFU	A	320	1	1
1QFU	A	321	1	1
1QFU	A	322	0	−1
1QFU	A	323	1	1
1QFU	A	324	1	1
1QFU	A	325	1	1
1QFU	B	1	1	1
1QFU	B	2	1	−1
1QFU	B	3	1	−1
1QFU	B	4	1	−1
1QFU	B	5	0	−1
1QFU	B	6	1	1
1QFU	B	7	1	1
1QFU	B	8	1	−1
1QFU	B	9	1	−1
1QFU	B	10	0	−1
1QFU	B	11	1	1
1QFU	B	12	1	1

1QFU	B	13	1	1
1QFU	B	14	1	1
1QFU	B	15	1	1
1QFU	B	16	1	1
1QFU	B	17	0	−1
1QFU	B	18	1	−1
1QFU	B	19	1	−1
1QFU	B	20	0	−1
1QFU	B	21	1	1
1QFU	B	22	0	−1
1QFU	B	23	0	−1
1QFU	B	24	0	−1
1QFU	B	25	1	1
1QFU	B	26	0	−1
1QFU	B	27	1	1
1QFU	B	28	0	−1
1QFU	B	29	1	1
1QFU	B	30	1	−1
1QFU	B	31	1	−1
1QFU	B	32	1	−1
1QFU	B	33	1	−1
1QFU	B	34	1	−1
1QFU	B	35	0	−1
1QFU	B	36	0	−1
1QFU	B	37	0	−1
1QFU	B	38	1	−1
1QFU	B	39	1	−1
1QFU	B	40	0	−1
1QFU	B	41	0	−1
1QFU	B	42	1	−1
1QFU	B	43	1	−1
1QFU	B	44	0	−1
1QFU	B	45	0	−1
1QFU	B	46	1	−1
1QFU	B	47	1	−1
1QFU	B	48	0	−1
1QFU	B	49	1	−1
1QFU	B	50	1	−1
1QFU	B	51	0	−1
1QFU	B	52	1	1
1QFU	B	53	1	−1
1QFU	B	54	1	−1
1QFU	B	55	1	1
1QFU	B	56	1	1
1QFU	B	57	1	1
1QFU	B	58	1	1
1QFU	B	59	1	1
1QFU	B	60	1	1
1QFU	B	61	1	1
1QFU	B	62	1	1
1QFU	B	63	1	1
1QFU	B	64	1	1

1QFU	B	65	1	1
1QFU	B	66	1	−1
1QFU	B	67	1	1
1QFU	B	68	1	1
1QFU	B	69	1	1
1QFU	B	70	1	−1
1QFU	B	71	1	1
1QFU	B	72	1	−1
1QFU	B	73	1	−1
1QFU	B	74	1	1
1QFU	B	75	1	−1
1QFU	B	76	1	−1
1QFU	B	77	1	−1
1QFU	B	78	0	−1
1QFU	B	79	1	−1
1QFU	B	80	1	−1
1QFU	B	81	1	−1
1QFU	B	82	1	−1
1QFU	B	83	1	−1
1QFU	B	84	1	−1
1QFU	B	85	0	−1
1QFU	B	86	1	−1
1QFU	B	87	1	−1
1QFU	B	88	1	−1
1QFU	B	89	1	1
1QFU	B	90	1	1
1QFU	B	91	1	−1
1QFU	B	92	1	1
1QFU	B	93	1	1
1QFU	B	94	1	−1
1QFU	B	95	1	−1
1QFU	B	96	1	1
1QFU	B	97	1	1
1QFU	B	98	1	1
1QFU	B	99	1	1
1QFU	B	100	1	1
1QFU	B	101	1	1
1QFU	B	102	1	1
1QFU	B	103	0	−1
1QFU	B	104	1	1
1QFU	B	105	1	1
1QFU	B	106	1	−1
1QFU	B	107	0	−1
1QFU	B	108	1	1
1QFU	B	109	0	−1
1QFU	B	110	0	−1
1QFU	B	111	0	−1
1QFU	B	112	0	−1
1QFU	B	113	0	−1
1QFU	B	114	0	−1
1QFU	B	115	0	−1
1QFU	B	116	0	−1

1QFU	B	117	1	−1
1QFU	B	118	0	−1
1QFU	B	119	0	−1
1QFU	B	120	1	−1
1QFU	B	121	1	−1
1QFU	B	122	0	−1
1QFU	B	123	1	−1
1QFU	B	124	1	−1
1QFU	B	125	0	−1
1QFU	B	126	0	−1
1QFU	B	127	1	−1
1QFU	B	128	1	−1
1QFU	B	129	0	−1
1QFU	B	130	0	−1
1QFU	B	131	1	−1
1QFU	B	132	1	−1
1QFU	B	133	1	1
1QFU	B	134	1	−1
1QFU	B	135	1	−1
1QFU	B	136	1	1
1QFU	B	137	1	1
1QFU	B	138	0	−1
1QFU	B	139	1	1
1QFU	B	140	0	−1
1QFU	B	141	0	−1
1QFU	B	142	0	−1
1QFU	B	143	1	1
1QFU	B	144	0	−1
1QFU	B	145	1	−1
1QFU	B	146	1	−1
1QFU	B	147	1	−1
1QFU	B	148	0	−1
1QFU	B	149	0	−1
1QFU	B	150	1	−1
1QFU	B	151	0	−1
1QFU	B	152	0	−1
1QFU	B	153	1	1
1QFU	B	154	1	−1
1QFU	B	155	1	−1
1QFU	B	156	1	−1
1QFU	B	157	0	−1
1QFU	B	158	1	−1
1QFU	B	159	0	−1
1QFU	B	160	1	−1
1QFU	B	161	1	−1
1QFU	B	162	0	−1
1QFU	B	163	1	−1
1QFU	B	164	1	−1
1QFU	B	165	1	1
1QFU	B	166	0	−1
1QFU	B	167	1	−1
1QFU	B	168	1	−1

1QFU	B	169	1	1
1QFU	B	170	0	−1
1QFU	B	171	1	−1
1QFU	B	172	1	−1
1QFU	B	173	1	−1
1QFU	H	1	1	1
1QFU	H	2	0	−1
1QFU	H	3	1	−1
1QFU	H	4	0	−1
1QFU	H	5	1	−1
1QFU	H	6	0	−1
1QFU	H	7	1	−1
1QFU	H	8	1	−1
1QFU	H	9	1	−1
1QFU	H	10	1	−1
1QFU	H	11	1	−1
1QFU	H	12	0	−1
1QFU	H	13	1	−1
1QFU	H	14	1	−1
1QFU	H	15	1	−1
1QFU	H	16	1	−1
1QFU	H	17	1	−1
1QFU	H	18	0	−1
1QFU	H	19	1	−1
1QFU	H	20	0	−1
1QFU	H	21	1	−1
1QFU	H	22	0	−1
1QFU	H	23	1	−1
1QFU	H	24	0	−1
1QFU	H	25	1	−1
1QFU	H	26	1	1
1QFU	H	27	0	−1
1QFU	H	28	1	1
1QFU	H	29	0	−1
1QFU	H	30	1	1
1QFU	H	31	1	1
1QFU	H	32	1	1
1QFU	H	33	0	−1
1QFU	H	34	0	−1
1QFU	H	35	0	−1
1QFU	H	36	0	−1
1QFU	H	37	0	−1
1QFU	H	38	0	−1
1QFU	H	39	1	1
1QFU	H	40	1	−1
1QFU	H	41	1	−1
1QFU	H	42	1	−1
1QFU	H	43	1	1
1QFU	H	44	0	−1
1QFU	H	45	1	1
1QFU	H	46	0	−1
1QFU	H	47	1	1

1QFU	H	48	0	−1
1QFU	H	49	0	−1
1QFU	H	50	0	−1
1QFU	H	51	0	−1
1QFU	H	52	0	−1
1QFU	H	53	0	−1
1QFU	H	54	1	1
1QFU	H	55	1	1
1QFU	H	56	1	−1
1QFU	H	57	1	−1
1QFU	H	58	1	−1
1QFU	H	59	1	1
1QFU	H	60	0	−1
1QFU	H	61	1	1
1QFU	H	62	1	1
1QFU	H	63	1	−1
1QFU	H	64	0	−1
1QFU	H	65	1	−1
1QFU	H	66	1	−1
1QFU	H	67	0	−1
1QFU	H	68	0	−1
1QFU	H	69	1	−1
1QFU	H	70	0	−1
1QFU	H	71	1	−1
1QFU	H	72	0	−1
1QFU	H	73	1	−1
1QFU	H	74	1	−1
1QFU	H	75	1	−1
1QFU	H	76	1	−1
1QFU	H	77	1	1
1QFU	H	78	0	−1
1QFU	H	79	0	−1
1QFU	H	80	0	−1
1QFU	H	81	0	−1
1QFU	H	82	1	−1
1QFU	H	83	0	−1
1QFU	H	84	1	−1
1QFU	H	85	1	−1
1QFU	H	86	0	−1
1QFU	H	87	1	−1
1QFU	H	88	1	−1
1QFU	H	89	1	−1
1QFU	H	90	0	−1
1QFU	H	91	1	−1
1QFU	H	92	0	−1
1QFU	H	93	1	−1
1QFU	H	94	0	−1
1QFU	H	95	0	−1
1QFU	H	96	0	−1
1QFU	H	97	0	−1
1QFU	H	98	0	−1
1QFU	H	99	0	−1

1QFU	H	100	0	−1
1QFU	H	101	0	−1
1QFU	H	102	1	1
1QFU	H	103	1	1
1QFU	H	104	1	1
1QFU	H	105	1	1
1QFU	H	106	1	1
1QFU	H	107	1	1
1QFU	H	108	0	−1
1QFU	H	109	0	−1
1QFU	H	110	1	1
1QFU	H	111	1	1
1QFU	H	112	1	1
1QFU	H	113	0	−1
1QFU	H	114	1	1
1QFU	H	115	0	−1
1QFU	H	116	0	−1
1QFU	H	117	1	−1
1QFU	H	118	0	−1
1QFU	H	119	0	−1
1QFU	H	120	0	−1
1QFU	H	121	0	−1
1QFU	H	122	1	−1
1QFU	H	123	0	−1
1QFU	H	124	1	−1
1QFU	H	125	1	−1
1QFU	H	126	1	−1
1QFU	H	127	1	−1
1QFU	H	128	0	−1
1QFU	H	129	1	−1
1QFU	H	130	0	−1
1QFU	H	131	1	1
1QFU	H	132	1	1
1QFU	H	133	1	1
1QFU	H	134	0	−1
1QFU	H	135	0	−1
1QFU	H	136	0	−1
1QFU	H	137	1	1
1QFU	H	138	1	−1
1QFU	H	139	1	−1
1QFU	H	140	1	1
1QFU	H	141	1	−1
1QFU	H	142	1	1
1QFU	H	143	1	−1
1QFU	H	144	1	−1
1QFU	H	145	0	−1
1QFU	H	146	1	1
1QFU	H	147	0	−1
1QFU	H	148	0	−1
1QFU	H	149	0	−1
1QFU	H	150	1	1
1QFU	H	151	0	−1

1QFU	H	152	1	1
1QFU	H	153	0	−1
1QFU	H	154	0	−1
1QFU	H	155	0	−1
1QFU	H	156	0	−1
1QFU	H	157	1	−1
1QFU	H	158	1	−1
1QFU	H	159	0	−1
1QFU	H	160	1	−1
1QFU	H	161	0	−1
1QFU	H	162	1	−1
1QFU	H	163	0	−1
1QFU	H	164	1	−1
1QFU	H	165	1	−1
1QFU	H	166	1	−1
1QFU	H	167	1	−1
1QFU	H	168	0	−1
1QFU	H	169	1	−1
1QFU	H	170	1	−1
1QFU	H	171	1	−1
1QFU	H	172	1	−1
1QFU	H	173	1	1
1QFU	H	174	1	1
1QFU	H	175	1	1
1QFU	H	176	1	1
1QFU	H	177	0	−1
1QFU	H	178	1	1
1QFU	H	179	1	−1
1QFU	H	180	1	1
1QFU	H	181	1	−1
1QFU	H	182	1	−1
1QFU	H	183	1	−1
1QFU	H	184	0	−1
1QFU	H	185	0	−1
1QFU	H	186	0	−1
1QFU	H	187	1	1
1QFU	H	188	0	−1
1QFU	H	189	0	−1
1QFU	H	190	0	−1
1QFU	H	191	1	1
1QFU	H	192	0	−1
1QFU	H	193	1	−1
1QFU	H	194	1	−1
1QFU	H	195	1	−1
1QFU	H	196	1	−1
1QFU	H	197	0	−1
1QFU	H	198	1	−1
1QFU	H	199	1	−1
1QFU	H	200	1	−1
1QFU	H	201	1	−1
1QFU	H	202	0	−1
1QFU	H	203	0	−1

1QFU	H	204	0	−1
1QFU	H	205	0	−1
1QFU	H	206	0	−1
1QFU	H	207	1	−1
1QFU	H	208	0	−1
1QFU	H	209	1	−1
1QFU	H	210	1	−1
1QFU	H	211	0	−1
1QFU	H	212	1	−1
1QFU	H	213	0	−1
1QFU	H	214	1	−1
1QFU	H	215	0	−1
1QFU	H	216	1	−1
1QFU	H	217	1	1
1QFU	H	218	1	−1
1QFU	H	219	0	−1
1QFU	H	220	1	−1
1QFU	H	221	1	−1
1QFU	H	222	1	1
1QFU	H	223	1	−1
1QFU	L	1	1	1
1QFU	L	2	0	−1
1QFU	L	3	1	−1
1QFU	L	4	0	−1
1QFU	L	5	1	−1
1QFU	L	6	0	−1
1QFU	L	7	1	−1
1QFU	L	8	1	−1
1QFU	L	9	1	1
1QFU	L	10	1	−1
1QFU	L	11	0	−1
1QFU	L	12	1	−1
1QFU	L	13	0	−1
1QFU	L	14	1	−1
1QFU	L	15	1	−1
1QFU	L	16	1	−1
1QFU	L	17	1	−1
1QFU	L	18	1	−1
1QFU	L	19	0	−1
1QFU	L	20	1	−1
1QFU	L	21	0	−1
1QFU	L	22	0	−1
1QFU	L	23	0	−1
1QFU	L	24	1	−1
1QFU	L	25	0	−1
1QFU	L	26	1	−1
1QFU	L	27	1	−1
1QFU	L	28	1	−1
1QFU	L	29	0	−1
1QFU	L	30	1	−1
1QFU	L	31	1	1
1QFU	L	32	1	−1

1QFU	L	33	1	1
1QFU	L	34	1	−1
1QFU	L	35	1	−1
1QFU	L	36	0	−1
1QFU	L	37	0	−1
1QFU	L	38	0	−1
1QFU	L	39	0	−1
1QFU	L	40	0	−1
1QFU	L	41	0	−1
1QFU	L	42	0	−1
1QFU	L	43	1	1
1QFU	L	44	1	−1
1QFU	L	45	1	−1
1QFU	L	46	1	−1
1QFU	L	47	1	1
1QFU	L	48	1	1
1QFU	L	49	1	1
1QFU	L	50	1	1
1QFU	L	51	1	1
1QFU	L	52	0	−1
1QFU	L	53	0	−1
1QFU	L	54	1	1
1QFU	L	55	1	1
1QFU	L	56	0	−1
1QFU	L	57	1	−1
1QFU	L	58	1	−1
1QFU	L	59	1	1
1QFU	L	60	1	1
1QFU	L	61	1	1
1QFU	L	62	1	1
1QFU	L	63	0	−1
1QFU	L	64	1	1
1QFU	L	65	1	−1
1QFU	L	66	0	−1
1QFU	L	67	0	−1
1QFU	L	68	1	−1
1QFU	L	69	0	−1
1QFU	L	70	1	−1
1QFU	L	71	1	−1
1QFU	L	72	1	−1
1QFU	L	73	0	−1
1QFU	L	74	1	−1
1QFU	L	75	1	−1
1QFU	L	76	0	−1
1QFU	L	77	1	−1
1QFU	L	78	0	−1
1QFU	L	79	1	−1
1QFU	L	80	0	−1
1QFU	L	81	1	−1
1QFU	L	82	1	−1
1QFU	L	83	0	−1
1QFU	L	84	0	−1

1QFU	L	85	0	−1
1QFU	L	86	1	1
1QFU	L	87	0	−1
1QFU	L	88	0	−1
1QFU	L	89	0	−1
1QFU	L	90	0	−1
1QFU	L	91	0	−1
1QFU	L	92	0	−1
1QFU	L	93	0	−1
1QFU	L	94	0	−1
1QFU	L	95	0	−1
1QFU	L	96	1	1
1QFU	L	97	0	−1
1QFU	L	98	1	−1
1QFU	L	99	1	1
1QFU	L	100	1	1
1QFU	L	101	1	1
1QFU	L	102	0	−1
1QFU	L	103	1	1
1QFU	L	104	0	−1
1QFU	L	105	1	1
1QFU	L	106	0	−1
1QFU	L	107	0	−1
1QFU	L	108	1	1
1QFU	L	109	0	−1
1QFU	L	110	0	−1
1QFU	L	111	0	−1
1QFU	L	112	1	−1
1QFU	L	113	1	−1
1QFU	L	114	1	−1
1QFU	L	115	1	−1
1QFU	L	116	0	−1
1QFU	L	117	1	−1
1QFU	L	118	0	−1
1QFU	L	119	1	1
1QFU	L	120	0	−1
1QFU	L	121	1	1
1QFU	L	122	0	−1
1QFU	L	123	1	1
1QFU	L	124	1	1
1QFU	L	125	0	−1
1QFU	L	126	1	1
1QFU	L	127	1	−1
1QFU	L	128	1	1
1QFU	L	129	1	1
1QFU	L	130	0	−1
1QFU	L	131	1	−1
1QFU	L	132	1	1
1QFU	L	133	1	−1
1QFU	L	134	0	−1
1QFU	L	135	0	−1
1QFU	L	136	0	−1

1QFU	L	137	0	−1
1QFU	L	138	0	−1
1QFU	L	139	0	−1
1QFU	L	140	1	1
1QFU	L	141	0	−1
1QFU	L	142	1	1
1QFU	L	143	1	1
1QFU	L	144	0	−1
1QFU	L	145	0	−1
1QFU	L	146	0	−1
1QFU	L	147	1	−1
1QFU	L	148	1	−1
1QFU	L	149	0	−1
1QFU	L	150	1	−1
1QFU	L	151	0	−1
1QFU	L	152	0	−1
1QFU	L	153	0	−1
1QFU	L	154	1	−1
1QFU	L	155	0	−1
1QFU	L	156	1	−1
1QFU	L	157	1	−1
1QFU	L	158	1	−1
1QFU	L	159	1	−1
1QFU	L	160	0	−1
1QFU	L	161	1	−1
1QFU	L	162	1	−1
1QFU	L	163	1	1
1QFU	L	164	0	−1
1QFU	L	165	1	1
1QFU	L	166	1	1
1QFU	L	167	1	1
1QFU	L	168	1	1
1QFU	L	169	1	1
1QFU	L	170	1	1
1QFU	L	171	0	−1
1QFU	L	172	1	1
1QFU	L	173	1	−1
1QFU	L	174	1	−1
1QFU	L	175	1	−1
1QFU	L	176	0	−1
1QFU	L	177	0	−1
1QFU	L	178	0	−1
1QFU	L	179	0	−1
1QFU	L	180	0	−1
1QFU	L	181	0	−1
1QFU	L	182	0	−1
1QFU	L	183	0	−1
1QFU	L	184	0	−1
1QFU	L	185	1	1
1QFU	L	186	0	−1
1QFU	L	187	1	−1
1QFU	L	188	1	−1

1QFU	L	189	1	−1
1QFU	L	190	1	−1
1QFU	L	191	0	−1
1QFU	L	192	1	−1
1QFU	L	193	1	−1
1QFU	L	194	1	−1
1QFU	L	195	1	−1
1QFU	L	196	0	−1
1QFU	L	197	0	−1
1QFU	L	198	0	−1
1QFU	L	199	0	−1
1QFU	L	200	0	−1
1QFU	L	201	0	−1
1QFU	L	202	1	−1
1QFU	L	203	0	−1
1QFU	L	204	1	−1
1QFU	L	205	1	−1
1QFU	L	206	1	−1
1QFU	L	207	1	−1
1QFU	L	208	1	−1
1QFU	L	209	1	−1
1QFU	L	210	1	−1
1QFU	L	211	1	−1
1QFU	L	212	1	1
1QFU	L	213	1	1
1QFU	L	214	0	−1
1QFU	L	215	1	1
1QFU	L	216	1	−1
1QFU	L	217	1	1
1SEB	F	1	1	−1
1SEB	F	2	1	−1
1SEB	F	3	1	−1
1SEB	F	4	1	−1
1SEB	F	5	0	−1
1SEB	F	6	0	−1
1SEB	F	7	0	−1
1SEB	F	8	0	−1
1SEB	F	9	0	−1
1SEB	F	10	0	−1
1SEB	F	11	0	−1
1SEB	F	12	0	−1
1SEB	F	13	0	−1
1SEB	F	14	0	−1
1SEB	F	15	0	−1
1SEB	F	16	0	−1
1SEB	F	17	1	1
1SEB	F	18	1	1
1SEB	F	19	1	1
1SEB	F	20	1	1
1SEB	F	21	0	−1
1SEB	F	22	0	−1
1SEB	F	23	0	−1

1SEB	F	24	0	−1
1SEB	F	25	0	−1
1SEB	F	26	0	−1
1SEB	F	27	1	−1
1SEB	F	28	0	−1
1SEB	F	29	0	−1
1SEB	F	30	0	−1
1SEB	F	31	0	−1
1SEB	F	32	0	−1
1SEB	F	33	0	−1
1SEB	F	34	0	−1
1SEB	F	35	0	−1
1SEB	F	36	1	1
1SEB	F	37	1	1
1SEB	F	38	1	1
1SEB	F	39	1	1
1SEB	F	40	1	−1
1SEB	F	41	0	−1
1SEB	F	42	1	−1
1SEB	F	43	0	−1
1SEB	F	44	0	−1
1SEB	F	45	0	−1
1SEB	F	46	1	−1
1SEB	F	47	1	−1
1SEB	F	48	0	−1
1SEB	F	49	1	−1
1SEB	F	50	1	−1
1SEB	F	51	1	1
1SEB	F	52	0	−1
1SEB	F	53	1	1
1SEB	F	54	1	1
1SEB	F	55	1	1
1SEB	F	56	0	−1
1SEB	F	57	1	1
1SEB	F	58	1	1
1SEB	F	59	0	−1
1SEB	F	60	1	1
1SEB	F	61	1	1
1SEB	F	62	1	1
1SEB	F	63	0	−1
1SEB	F	64	1	1
1SEB	F	65	1	1
1SEB	F	66	0	−1
1SEB	F	67	1	1
1SEB	F	68	1	1
1SEB	F	69	1	1
1SEB	F	70	0	−1
1SEB	F	71	1	1
1SEB	F	72	1	1
1SEB	F	73	0	−1
1SEB	F	74	0	−1
1SEB	F	75	1	−1

1SEB	F	76	1	1
1SEB	F	77	0	−1
1SEB	F	78	1	−1
1SEB	F	79	1	−1
1SEB	F	80	1	−1
1SEB	F	81	1	−1
1SEB	F	82	0	−1
1SEB	F	83	1	−1
1SEB	F	84	1	−1
1SEB	F	85	1	−1
1SEB	F	86	1	−1
1SEB	F	87	0	−1
1SEB	F	88	1	−1
1SEB	F	89	0	−1
1SEB	F	90	1	−1
1SEB	F	91	0	−1
1SEB	F	92	1	−1
1SEB	F	93	0	−1
1SEB	F	94	1	−1
1SEB	F	95	1	−1
1SEB	F	96	1	−1
1SEB	F	97	0	−1
1SEB	F	98	1	−1
1SEB	F	99	1	−1
1SEB	F	100	1	−1
1SEB	F	101	1	−1
1SEB	F	102	1	−1
1SEB	F	103	0	−1
1SEB	F	104	0	−1
1SEB	F	105	0	−1
1SEB	F	106	0	−1
1SEB	F	107	0	−1
1SEB	F	108	0	−1
1SEB	F	109	0	−1
1SEB	F	110	0	−1
1SEB	F	111	0	−1
1SEB	F	112	0	−1
1SEB	F	113	0	−1
1SEB	F	114	0	−1
1SEB	F	115	0	−1
1SEB	F	116	0	−1
1SEB	F	117	0	−1
1SEB	F	118	1	−1
1SEB	F	119	0	−1
1SEB	F	120	1	−1
1SEB	F	121	0	−1
1SEB	F	122	0	−1
1SEB	F	123	1	−1
1SEB	F	124	1	−1
1SEB	F	125	1	−1
1SEB	F	126	1	−1
1SEB	F	127	1	−1

1SEB	F	128	0	−1
1SEB	F	129	1	−1
1SEB	F	130	1	−1
1SEB	F	131	1	−1
1SEB	F	132	1	−1
1SEB	F	133	1	−1
1SEB	F	134	1	−1
1SEB	F	135	0	−1
1SEB	F	136	0	−1
1SEB	F	137	0	−1
1SEB	F	138	0	−1
1SEB	F	139	0	−1
1SEB	F	140	0	−1
1SEB	F	141	1	−1
1SEB	F	142	1	−1
1SEB	F	143	0	−1
1SEB	F	144	0	−1
1SEB	F	145	0	−1
1SEB	F	146	0	−1
1SEB	F	147	0	−1
1SEB	F	148	0	−1
1SEB	F	149	0	−1
1SEB	F	150	0	−1
1SEB	F	151	0	−1
1SEB	F	152	1	−1
1SEB	F	153	0	−1
1SEB	F	154	1	−1
1SEB	F	155	0	−1
1SEB	F	156	0	−1
1SEB	F	157	1	−1
1SEB	F	158	1	−1
1SEB	F	159	0	−1
1SEB	F	160	0	−1
1SEB	F	161	0	−1
1SEB	F	162	0	−1
1SEB	F	163	0	−1
1SEB	F	164	1	−1
1SEB	F	165	0	−1
1SEB	F	166	1	−1
1SEB	F	167	0	−1
1SEB	F	168	1	−1
1SEB	F	169	1	−1
1SEB	F	170	1	−1
1SEB	F	171	1	−1
1SEB	F	172	1	−1
1SEB	F	173	1	−1
1SEB	F	174	1	−1
1SEB	F	175	0	−1
1SEB	F	176	1	−1
1SEB	F	177	0	−1
1SEB	F	178	0	−1
1SEB	F	179	0	−1

1SEB	F	180	1	−1
1SEB	F	181	1	−1
1SEB	F	1	1	−1
1SEB	F	2	1	−1
1SEB	F	3	1	−1
1SEB	F	4	1	−1
1SEB	F	5	1	−1
1SEB	F	6	0	−1
1SEB	F	7	0	−1
1SEB	F	8	0	−1
1SEB	F	9	0	−1
1SEB	F	10	0	−1
1SEB	F	11	0	−1
1SEB	F	12	0	−1
1SEB	F	13	0	−1
1SEB	F	14	0	−1
1SEB	F	15	0	−1
1SEB	F	16	0	−1
1SEB	F	17	0	−1
1SEB	F	18	0	−1
1SEB	F	19	1	−1
1SEB	F	20	0	−1
1SEB	F	21	1	−1
1SEB	F	22	1	−1
1SEB	F	23	0	−1
1SEB	F	24	0	−1
1SEB	F	25	0	−1
1SEB	F	26	0	−1
1SEB	F	27	0	−1
1SEB	F	28	0	−1
1SEB	F	29	0	−1
1SEB	F	30	0	−1
1SEB	F	31	0	−1
1SEB	F	32	0	−1
1SEB	F	33	0	−1
1SEB	F	34	1	−1
1SEB	F	35	1	−1
1SEB	F	36	0	−1
1SEB	F	37	0	−1
1SEB	F	38	0	−1
1SEB	F	39	1	−1
1SEB	F	40	0	−1
1SEB	F	41	0	−1
1SEB	F	42	0	−1
1SEB	F	43	1	−1
1SEB	F	44	1	−1
1SEB	F	45	0	−1
1SEB	F	46	1	−1
1SEB	F	47	0	−1
1SEB	F	48	1	−1
1SEB	F	49	0	−1
1SEB	F	50	0	−1

1SEB	F	51	0	−1
1SEB	F	52	1	−1
1SEB	F	53	0	−1
1SEB	F	54	0	−1
1SEB	F	55	0	−1
1SEB	F	56	1	1
1SEB	F	57	0	−1
1SEB	F	58	0	−1
1SEB	F	59	1	−1
1SEB	F	60	1	1
1SEB	F	61	0	−1
1SEB	F	62	0	−1
1SEB	F	63	1	−1
1SEB	F	64	0	−1
1SEB	F	65	1	−1
1SEB	F	66	1	−1
1SEB	F	67	0	−1
1SEB	F	68	0	−1
1SEB	F	69	1	−1
1SEB	F	70	1	1
1SEB	F	71	0	−1
1SEB	F	72	1	−1
1SEB	F	73	1	−1
1SEB	F	74	0	−1
1SEB	F	75	0	−1
1SEB	F	76	1	−1
1SEB	F	77	1	1
1SEB	F	78	0	−1
1SEB	F	79	0	−1
1SEB	F	80	1	−1
1SEB	F	81	1	1
1SEB	F	82	0	−1
1SEB	F	83	0	−1
1SEB	F	84	1	−1
1SEB	F	85	1	1
1SEB	F	86	0	−1
1SEB	F	87	1	−1
1SEB	F	88	1	−1
1SEB	F	89	0	−1
1SEB	F	90	0	−1
1SEB	F	91	0	−1
1SEB	F	92	1	−1
1SEB	F	93	0	−1
1SEB	F	94	1	−1
1SEB	F	95	1	−1
1SEB	F	96	1	−1
1SEB	F	97	0	−1
1SEB	F	98	1	−1
1SEB	F	99	0	−1
1SEB	F	100	1	−1
1SEB	F	101	0	−1
1SEB	F	102	1	−1

1SEB	F	103	0	−1
1SEB	F	104	1	−1
1SEB	F	105	0	−1
1SEB	F	106	1	−1
1SEB	F	107	1	−1
1SEB	F	108	1	−1
1SEB	F	109	1	−1
1SEB	F	110	1	−1
1SEB	F	111	1	−1
1SEB	F	112	0	−1
1SEB	F	113	0	−1
1SEB	F	114	0	−1
1SEB	F	115	0	−1
1SEB	F	116	0	−1
1SEB	F	117	0	−1
1SEB	F	118	0	−1
1SEB	F	119	0	−1
1SEB	F	120	0	−1
1SEB	F	121	1	−1
1SEB	F	122	0	−1
1SEB	F	123	0	−1
1SEB	F	124	0	−1
1SEB	F	125	0	−1
1SEB	F	126	1	−1
1SEB	F	127	1	−1
1SEB	F	128	1	−1
1SEB	F	129	0	−1
1SEB	F	130	1	−1
1SEB	F	131	0	−1
1SEB	F	132	0	−1
1SEB	F	133	1	−1
1SEB	F	134	1	−1
1SEB	F	135	1	−1
1SEB	F	136	1	−1
1SEB	F	137	1	−1
1SEB	F	138	0	−1
1SEB	F	139	1	−1
1SEB	F	140	1	−1
1SEB	F	141	0	−1
1SEB	F	142	0	−1
1SEB	F	143	0	−1
1SEB	F	144	0	−1
1SEB	F	145	1	−1
1SEB	F	146	1	−1
1SEB	F	147	1	−1
1SEB	F	148	0	−1
1SEB	F	149	0	−1
1SEB	F	150	0	−1
1SEB	F	151	0	−1
1SEB	F	152	1	−1
1SEB	F	153	0	−1
1SEB	F	154	0	−1

1SEB	F	155	0	−1
1SEB	F	156	0	−1
1SEB	F	157	0	−1
1SEB	F	158	0	−1
1SEB	F	159	0	−1
1SEB	F	160	0	−1
1SEB	F	161	0	−1
1SEB	F	162	0	−1
1SEB	F	163	0	−1
1SEB	F	164	1	−1
1SEB	F	165	0	−1
1SEB	F	166	1	−1
1SEB	F	167	1	−1
1SEB	F	168	1	−1
1SEB	F	169	0	−1
1SEB	F	170	0	−1
1SEB	F	171	0	−1
1SEB	F	172	0	−1
1SEB	F	173	0	−1
1SEB	F	174	0	−1
1SEB	F	175	0	−1
1SEB	F	176	1	−1
1SEB	F	177	0	−1
1SEB	F	178	1	−1
1SEB	F	179	0	−1
1SEB	F	180	0	−1
1SEB	F	181	1	−1
1SEB	F	182	1	−1
1SEB	F	183	1	−1
1SEB	F	184	0	−1
1SEB	F	185	1	−1
1SEB	F	186	0	−1
1SEB	F	187	1	−1
1SEB	F	188	0	−1
1SEB	F	189	1	−1
1SEB	F	190	0	−1
1SEB	F	191	1	−1
1SEB	F	192	1	−1
1SEB	F	1	1	−1
1SEB	F	2	1	−1
1SEB	F	3	1	−1
1SEB	F	4	1	−1
1SEB	F	5	0	−1
1SEB	F	6	0	−1
1SEB	F	7	0	−1
1SEB	F	8	0	−1
1SEB	F	9	0	−1
1SEB	F	10	0	−1
1SEB	F	11	0	−1
1SEB	F	12	0	−1
1SEB	F	13	0	−1
1SEB	F	14	0	−1

1SEB	F	15	0	−1
1SEB	F	16	0	−1
1SEB	F	17	1	1
1SEB	F	18	1	1
1SEB	F	19	1	1
1SEB	F	20	1	1
1SEB	F	21	0	−1
1SEB	F	22	0	−1
1SEB	F	23	0	−1
1SEB	F	24	0	−1
1SEB	F	25	0	−1
1SEB	F	26	0	−1
1SEB	F	27	1	−1
1SEB	F	28	0	−1
1SEB	F	29	0	−1
1SEB	F	30	0	−1
1SEB	F	31	0	−1
1SEB	F	32	0	−1
1SEB	F	33	0	−1
1SEB	F	34	0	−1
1SEB	F	35	0	−1
1SEB	F	36	1	1
1SEB	F	37	1	1
1SEB	F	38	1	1
1SEB	F	39	1	1
1SEB	F	40	1	−1
1SEB	F	41	0	−1
1SEB	F	42	1	−1
1SEB	F	43	0	−1
1SEB	F	44	0	−1
1SEB	F	45	0	−1
1SEB	F	46	1	−1
1SEB	F	47	1	−1
1SEB	F	48	0	−1
1SEB	F	49	1	−1
1SEB	F	50	1	−1
1SEB	F	51	1	1
1SEB	F	52	0	−1
1SEB	F	53	1	1
1SEB	F	54	1	1
1SEB	F	55	1	1
1SEB	F	56	0	−1
1SEB	F	57	1	1
1SEB	F	58	1	1
1SEB	F	59	0	−1
1SEB	F	60	1	1
1SEB	F	61	1	1
1SEB	F	62	1	1
1SEB	F	63	0	−1
1SEB	F	64	1	1
1SEB	F	65	1	1
1SEB	F	66	0	−1

1SEB	F	67	1	1
1SEB	F	68	1	1
1SEB	F	69	1	1
1SEB	F	70	0	−1
1SEB	F	71	1	1
1SEB	F	72	1	1
1SEB	F	73	0	−1
1SEB	F	74	0	−1
1SEB	F	75	1	−1
1SEB	F	76	1	1
1SEB	F	77	0	−1
1SEB	F	78	1	−1
1SEB	F	79	1	−1
1SEB	F	80	1	−1
1SEB	F	81	1	−1
1SEB	F	82	0	−1
1SEB	F	83	1	−1
1SEB	F	84	1	−1
1SEB	F	85	0	−1
1SEB	F	86	1	−1
1SEB	F	87	0	−1
1SEB	F	88	1	−1
1SEB	F	89	0	−1
1SEB	F	90	1	−1
1SEB	F	91	0	−1
1SEB	F	92	1	−1
1SEB	F	93	0	−1
1SEB	F	94	1	−1
1SEB	F	95	1	−1
1SEB	F	96	0	−1
1SEB	F	97	0	−1
1SEB	F	98	1	−1
1SEB	F	99	1	−1
1SEB	F	100	1	−1
1SEB	F	101	1	−1
1SEB	F	102	1	−1
1SEB	F	103	0	−1
1SEB	F	104	0	−1
1SEB	F	105	0	−1
1SEB	F	106	0	−1
1SEB	F	107	0	−1
1SEB	F	108	0	−1
1SEB	F	109	0	−1
1SEB	F	110	0	−1
1SEB	F	111	0	−1
1SEB	F	112	0	−1
1SEB	F	113	0	−1
1SEB	F	114	0	−1
1SEB	F	115	0	−1
1SEB	F	116	0	−1
1SEB	F	117	0	−1
1SEB	F	118	1	−1

1SEB	F	119	0	−1
1SEB	F	120	1	−1
1SEB	F	121	0	−1
1SEB	F	122	0	−1
1SEB	F	123	1	−1
1SEB	F	124	1	−1
1SEB	F	125	1	−1
1SEB	F	126	1	−1
1SEB	F	127	1	−1
1SEB	F	128	0	−1
1SEB	F	129	1	−1
1SEB	F	130	1	−1
1SEB	F	131	1	−1
1SEB	F	132	1	−1
1SEB	F	133	1	−1
1SEB	F	134	1	−1
1SEB	F	135	0	−1
1SEB	F	136	0	−1
1SEB	F	137	0	−1
1SEB	F	138	0	−1
1SEB	F	139	0	−1
1SEB	F	140	0	−1
1SEB	F	141	1	−1
1SEB	F	142	1	−1
1SEB	F	143	0	−1
1SEB	F	144	0	−1
1SEB	F	145	0	−1
1SEB	F	146	0	−1
1SEB	F	147	0	−1
1SEB	F	148	0	−1
1SEB	F	149	0	−1
1SEB	F	150	0	−1
1SEB	F	151	0	−1
1SEB	F	152	1	−1
1SEB	F	153	0	−1
1SEB	F	154	1	−1
1SEB	F	155	0	−1
1SEB	F	156	0	−1
1SEB	F	157	1	−1
1SEB	F	158	1	−1
1SEB	F	159	1	−1
1SEB	F	160	0	−1
1SEB	F	161	0	−1
1SEB	F	162	0	−1
1SEB	F	163	0	−1
1SEB	F	164	1	−1
1SEB	F	165	0	−1
1SEB	F	166	1	−1
1SEB	F	167	0	−1
1SEB	F	168	1	−1
1SEB	F	169	1	−1
1SEB	F	170	1	−1

1SEB	F	171	1	−1
1SEB	F	172	1	−1
1SEB	F	173	1	−1
1SEB	F	174	0	−1
1SEB	F	175	0	−1
1SEB	F	176	1	−1
1SEB	F	177	0	−1
1SEB	F	178	0	−1
1SEB	F	179	0	−1
1SEB	F	180	1	−1
1SEB	F	181	1	−1
1SEB	F	1	1	−1
1SEB	F	2	1	−1
1SEB	F	3	1	−1
1SEB	F	4	1	−1
1SEB	F	5	1	−1
1SEB	F	6	0	−1
1SEB	F	7	0	−1
1SEB	F	8	0	−1
1SEB	F	9	0	−1
1SEB	F	10	0	−1
1SEB	F	11	0	−1
1SEB	F	12	0	−1
1SEB	F	13	0	−1
1SEB	F	14	0	−1
1SEB	F	15	0	−1
1SEB	F	16	0	−1
1SEB	F	17	0	−1
1SEB	F	18	0	−1
1SEB	F	19	1	−1
1SEB	F	20	0	−1
1SEB	F	21	1	−1
1SEB	F	22	1	−1
1SEB	F	23	0	−1
1SEB	F	24	0	−1
1SEB	F	25	0	−1
1SEB	F	26	0	−1
1SEB	F	27	0	−1
1SEB	F	28	0	−1
1SEB	F	29	0	−1
1SEB	F	30	0	−1
1SEB	F	31	0	−1
1SEB	F	32	0	−1
1SEB	F	33	0	−1
1SEB	F	34	1	−1
1SEB	F	35	1	−1
1SEB	F	36	0	−1
1SEB	F	37	0	−1
1SEB	F	38	0	−1
1SEB	F	39	1	−1
1SEB	F	40	0	−1
1SEB	F	41	0	−1

1SEB	F	42	0	−1
1SEB	F	43	1	−1
1SEB	F	44	1	−1
1SEB	F	45	0	−1
1SEB	F	46	1	−1
1SEB	F	47	0	−1
1SEB	F	48	1	−1
1SEB	F	49	0	−1
1SEB	F	50	1	−1
1SEB	F	51	0	−1
1SEB	F	52	1	−1
1SEB	F	53	0	−1
1SEB	F	54	0	−1
1SEB	F	55	0	−1
1SEB	F	56	1	1
1SEB	F	57	0	−1
1SEB	F	58	0	−1
1SEB	F	59	1	−1
1SEB	F	60	1	1
1SEB	F	61	0	−1
1SEB	F	62	0	−1
1SEB	F	63	1	−1
1SEB	F	64	0	−1
1SEB	F	65	1	−1
1SEB	F	66	1	−1
1SEB	F	67	0	−1
1SEB	F	68	0	−1
1SEB	F	69	1	−1
1SEB	F	70	1	1
1SEB	F	71	0	−1
1SEB	F	72	1	−1
1SEB	F	73	1	−1
1SEB	F	74	0	−1
1SEB	F	75	0	−1
1SEB	F	76	1	−1
1SEB	F	77	1	1
1SEB	F	78	0	−1
1SEB	F	79	0	−1
1SEB	F	80	1	−1
1SEB	F	81	1	1
1SEB	F	82	0	−1
1SEB	F	83	0	−1
1SEB	F	84	1	−1
1SEB	F	85	1	1
1SEB	F	86	0	−1
1SEB	F	87	1	−1
1SEB	F	88	1	−1
1SEB	F	89	0	−1
1SEB	F	90	0	−1
1SEB	F	91	0	−1
1SEB	F	92	1	−1
1SEB	F	93	0	−1

1SEB	F	94	1	−1
1SEB	F	95	1	−1
1SEB	F	96	1	−1
1SEB	F	97	0	−1
1SEB	F	98	1	−1
1SEB	F	99	0	−1
1SEB	F	100	1	−1
1SEB	F	101	0	−1
1SEB	F	102	1	−1
1SEB	F	103	0	−1
1SEB	F	104	1	−1
1SEB	F	105	0	−1
1SEB	F	106	1	−1
1SEB	F	107	1	−1
1SEB	F	108	1	−1
1SEB	F	109	1	−1
1SEB	F	110	1	−1
1SEB	F	111	1	−1
1SEB	F	112	0	−1
1SEB	F	113	0	−1
1SEB	F	114	0	−1
1SEB	F	115	0	−1
1SEB	F	116	0	−1
1SEB	F	117	0	−1
1SEB	F	118	0	−1
1SEB	F	119	0	−1
1SEB	F	120	0	−1
1SEB	F	121	1	−1
1SEB	F	122	0	−1
1SEB	F	123	0	−1
1SEB	F	124	1	−1
1SEB	F	125	0	−1
1SEB	F	126	1	−1
1SEB	F	127	0	−1
1SEB	F	128	1	−1
1SEB	F	129	0	−1
1SEB	F	130	1	−1
1SEB	F	131	0	−1
1SEB	F	132	0	−1
1SEB	F	133	1	−1
1SEB	F	134	1	−1
1SEB	F	135	1	−1
1SEB	F	136	1	−1
1SEB	F	137	1	−1
1SEB	F	138	0	−1
1SEB	F	139	1	−1
1SEB	F	140	1	−1
1SEB	F	141	0	−1
1SEB	F	142	1	−1
1SEB	F	143	0	−1
1SEB	F	144	0	−1
1SEB	F	145	1	−1

1SEB	F	146	1	−1
1SEB	F	147	1	−1
1SEB	F	148	0	−1
1SEB	F	149	0	−1
1SEB	F	150	0	−1
1SEB	F	151	0	−1
1SEB	F	152	0	−1
1SEB	F	153	0	−1
1SEB	F	154	0	−1
1SEB	F	155	0	−1
1SEB	F	156	0	−1
1SEB	F	157	0	−1
1SEB	F	158	0	−1
1SEB	F	159	0	−1
1SEB	F	160	0	−1
1SEB	F	161	0	−1
1SEB	F	162	0	−1
1SEB	F	163	0	−1
1SEB	F	164	1	−1
1SEB	F	165	0	−1
1SEB	F	166	1	−1
1SEB	F	167	1	−1
1SEB	F	168	1	−1
1SEB	F	169	0	−1
1SEB	F	170	0	−1
1SEB	F	171	0	−1
1SEB	F	172	0	−1
1SEB	F	173	0	−1
1SEB	F	174	0	−1
1SEB	F	175	0	−1
1SEB	F	176	1	−1
1SEB	F	177	0	−1
1SEB	F	178	1	−1
1SEB	F	179	0	−1
1SEB	F	180	0	−1
1SEB	F	181	1	−1
1SEB	F	182	1	−1
1SEB	F	183	1	−1
1SEB	F	184	0	−1
1SEB	F	185	1	−1
1SEB	F	186	0	−1
1SEB	F	187	1	−1
1SEB	F	188	0	−1
1SEB	F	189	1	−1
1SEB	F	190	0	−1
1SEB	F	191	1	−1
1SEB	F	192	1	−1
1STF	E	1	1	−1
1STF	E	2	1	−1
1STF	E	3	1	−1
1STF	E	4	1	−1
1STF	E	5	0	−1

1STF	E	6	0	−1
1STF	E	7	0	−1
1STF	E	8	1	−1
1STF	E	9	1	−1
1STF	E	10	1	−1
1STF	E	11	1	−1
1STF	E	12	0	−1
1STF	E	13	0	−1
1STF	E	14	0	−1
1STF	E	15	1	−1
1STF	E	16	0	−1
1STF	E	17	0	−1
1STF	E	18	1	1
1STF	E	19	0	−1
1STF	E	20	1	1
1STF	E	21	1	1
1STF	E	22	0	−1
1STF	E	23	1	1
1STF	E	24	0	−1
1STF	E	26	0	−1
1STF	E	27	0	−1
1STF	E	28	0	−1
1STF	E	29	0	−1
1STF	E	30	0	−1
1STF	E	31	0	−1
1STF	E	32	0	−1
1STF	E	33	0	−1
1STF	E	34	0	−1
1STF	E	35	0	−1
1STF	E	36	0	−1
1STF	E	37	0	−1
1STF	E	38	0	−1
1STF	E	39	0	−1
1STF	E	40	0	−1
1STF	E	41	1	−1
1STF	E	42	1	−1
1STF	E	43	1	−1
1STF	E	44	1	−1
1STF	E	45	1	−1
1STF	E	46	1	−1
1STF	E	47	1	−1
1STF	E	48	1	−1
1STF	E	49	0	−1
1STF	E	50	0	−1
1STF	E	51	0	−1
1STF	E	52	0	−1
1STF	E	53	0	−1
1STF	E	54	0	−1
1STF	E	55	0	−1
1STF	E	56	0	−1
1STF	E	57	0	−1
1STF	E	58	0	−1

1STF	E	59	1	−1
1STF	E	60	1	−1
1STF	E	61	0	−1
1STF	E	62	1	1
1STF	E	63	0	−1
1STF	E	64	1	1
1STF	E	65	1	1
1STF	E	66	1	1
1STF	E	67	1	1
1STF	E	68	1	1
1STF	E	69	0	−1
1STF	E	70	1	1
1STF	E	71	0	−1
1STF	E	72	0	−1
1STF	E	73	0	−1
1STF	E	74	1	−1
1STF	E	75	0	−1
1STF	E	76	0	−1
1STF	E	77	0	−1
1STF	E	78	1	−1
1STF	E	79	1	−1
1STF	E	80	0	−1
1STF	E	81	0	−1
1STF	E	82	0	−1
1STF	E	83	1	−1
1STF	E	84	0	−1
1STF	E	85	1	−1
1STF	E	86	1	−1
1STF	E	87	0	−1
1STF	E	88	1	−1
1STF	E	89	0	−1
1STF	E	90	1	−1
1STF	E	91	1	−1
1STF	E	92	1	−1
1STF	E	93	1	−1
1STF	E	94	1	−1
1STF	E	95	1	−1
1STF	E	96	1	−1
1STF	E	97	0	−1
1STF	E	98	0	−1
1STF	E	99	1	−1
1STF	E	100	1	−1
1STF	E	101	1	−1
1STF	E	102	1	−1
1STF	E	103	1	−1
1STF	E	104	1	−1
1STF	E	105	1	−1
1STF	E	106	0	−1
1STF	E	107	1	−1
1STF	E	108	0	−1
1STF	E	109	1	−1
1STF	E	110	0	−1

1STF	E	111	0	−1
1STF	E	112	1	−1
1STF	E	113	1	−1
1STF	E	114	0	−1
1STF	E	115	1	−1
1STF	E	116	1	−1
1STF	E	117	1	−1
1STF	E	118	1	−1
1STF	E	119	0	−1
1STF	E	120	1	−1
1STF	E	121	0	−1
1STF	E	122	0	−1
1STF	E	123	0	−1
1STF	E	124	1	−1
1STF	E	125	0	−1
1STF	E	126	0	−1
1STF	E	127	0	−1
1STF	E	128	1	−1
1STF	E	129	0	−1
1STF	E	130	0	−1
1STF	E	131	0	−1
1STF	E	132	0	−1
1STF	E	133	0	−1
1STF	E	134	0	−1
1STF	E	135	0	−1
1STF	E	136	0	−1
1STF	E	137	0	−1
1STF	E	138	1	1
1STF	E	139	1	−1
1STF	E	140	1	1
1STF	E	141	1	−1
1STF	E	142	0	−1
1STF	E	143	1	1
1STF	E	144	1	1
1STF	E	145	0	−1
1STF	E	146	1	−1
1STF	E	147	1	−1
1STF	E	148	1	−1
1STF	E	149	1	−1
1STF	E	150	0	−1
1STF	E	151	1	−1
1STF	E	152	0	−1
1STF	E	153	1	−1
1STF	E	154	1	−1
1STF	E	155	1	−1
1STF	E	156	1	−1
1STF	E	157	1	−1
1STF	E	158	1	1
1STF	E	159	1	1
1STF	E	160	0	−1
1STF	E	161	0	−1
1STF	E	162	0	−1

1STF	E	163	0	−1
1STF	E	164	0	−1
1STF	E	165	0	−1
1STF	E	166	0	−1
1STF	E	167	0	−1
1STF	E	168	0	−1
1STF	E	169	1	−1
1STF	E	170	1	−1
1STF	E	171	0	−1
1STF	E	172	0	−1
1STF	E	173	0	−1
1STF	E	174	0	−1
1STF	E	175	0	−1
1STF	E	176	0	−1
1STF	E	177	0	−1
1STF	E	178	1	1
1STF	E	179	0	−1
1STF	E	180	1	−1
1STF	E	181	1	1
1STF	E	182	0	−1
1STF	E	183	0	−1
1STF	E	184	0	−1
1STF	E	185	1	−1
1STF	E	186	0	−1
1STF	E	187	0	−1
1STF	E	188	0	−1
1STF	E	189	1	−1
1STF	E	190	0	−1
1STF	E	191	1	−1
1STF	E	192	0	−1
1STF	E	193	1	−1
1STF	E	194	1	−1
1STF	E	195	1	−1
1STF	E	196	1	−1
1STF	E	197	1	−1
1STF	E	198	1	−1
1STF	E	199	0	−1
1STF	E	200	0	−1
1STF	E	201	0	−1
1STF	E	202	0	−1
1STF	E	203	0	−1
1STF	E	204	0	−1
1STF	E	205	0	−1
1STF	E	206	1	1
1STF	E	207	0	−1
1STF	E	208	0	−1
1STF	E	209	0	−1
1STF	E	210	0	−1
1STF	E	211	0	−1
1STF	E	212	0	−1
1STF	I	1	1	1
1STF	I	2	1	1

1STF	I	3	1	1
1STF	I	4	1	1
1STF	I	5	1	1
1STF	I	6	1	1
1STF	I	7	1	−1
1STF	I	8	1	−1
1STF	I	9	1	−1
1STF	I	10	1	−1
1STF	I	11	1	−1
1STF	I	12	1	−1
1STF	I	13	1	−1
1STF	I	14	1	−1
1STF	I	15	1	−1
1STF	I	16	0	−1
1STF	I	17	0	−1
1STF	I	18	1	−1
1STF	I	19	0	−1
1STF	I	20	0	−1
1STF	I	21	1	−1
1STF	I	22	1	−1
1STF	I	23	0	−1
1STF	I	24	1	−1
1STF	I	25	1	−1
1STF	I	26	1	−1
1STF	I	27	0	−1
1STF	I	28	0	−1
1STF	I	29	1	−1
1STF	I	30	1	−1
1STF	I	31	1	−1
1STF	I	32	1	−1
1STF	I	33	1	−1
1STF	I	34	1	−1
1STF	I	35	0	−1
1STF	I	36	1	−1
1STF	I	37	1	−1
1STF	I	38	0	−1
1STF	I	39	1	−1
1STF	I	40	0	−1
1STF	I	41	1	−1
1STF	I	42	0	−1
1STF	I	43	0	−1
1STF	I	44	0	−1
1STF	I	45	0	−1
1STF	I	46	0	−1
1STF	I	47	1	1
1STF	I	48	1	1
1STF	I	49	1	1
1STF	I	50	0	−1
1STF	I	51	0	−1
1STF	I	52	0	−1
1STF	I	53	0	−1
1STF	I	54	0	−1

1STF	I	55	0	−1
1STF	I	56	0	−1
1STF	I	57	0	−1
1STF	I	58	1	−1
1STF	I	59	0	−1
1STF	I	60	0	−1
1STF	I	61	1	−1
1STF	I	62	1	−1
1STF	I	63	0	−1
1STF	I	64	0	−1
1STF	I	65	0	−1
1STF	I	66	0	−1
1STF	I	67	0	−1
1STF	I	68	0	−1
1STF	I	69	0	−1
1STF	I	70	1	1
1STF	I	71	1	1
1STF	I	72	0	−1
1STF	I	73	1	1
1STF	I	74	1	1
1STF	I	75	1	1
1STF	I	76	1	1
1STF	I	77	1	−1
1STF	I	78	1	−1
1STF	I	79	1	−1
1STF	I	80	0	−1
1STF	I	81	1	−1
1STF	I	82	0	−1
1STF	I	83	1	1
1STF	I	84	1	−1
1STF	I	85	0	−1
1STF	I	86	0	−1
1STF	I	87	0	−1
1STF	I	88	1	−1
1STF	I	89	0	−1
1STF	I	90	1	−1
1STF	I	91	1	−1
1STF	I	92	1	−1
1STF	I	93	1	−1
1STF	I	94	1	−1
1STF	I	95	0	−1
1STF	I	96	1	−1
1STF	I	97	1	1
1STF	I	98	1	−1
1TBQ	K	1	1	−1
1TBQ	K	2	1	−1
1TBQ	K	3	1	−1
1TBQ	K	4	1	−1
1TBQ	K	5	1	−1
1TBQ	K	6	0	−1
1TBQ	K	7	1	−1
1TBQ	K	8	1	−1

1TBQ	K	9	1	−1
1TBQ	K	10	0	−1
1TBQ	K	11	1	−1
1TBQ	K	12	1	−1
1TBQ	K	13	1	−1
1TBQ	K	14	0	−1
1TBQ	K	15	0	−1
1TBQ	K	16	0	−1
1TBQ	K	17	1	−1
1TBQ	K	18	0	−1
1TBQ	K	19	0	−1
1TBQ	K	20	0	−1
1TBQ	K	21	1	−1
1TBQ	K	22	0	−1
1TBQ	K	23	0	−1
1TBQ	K	24	0	−1
1TBQ	K	25	0	−1
1TBQ	K	26	0	−1
1TBQ	K	27	0	−1
1TBQ	K	28	0	−1
1TBQ	K	29	0	−1
1TBQ	K	30	1	−1
1TBQ	K	31	1	−1
1TBQ	K	32	1	−1
1TBQ	K	33	1	−1
1TBQ	K	34	1	−1
1TBQ	K	35	0	−1
1TBQ	K	36	1	−1
1TBQ	K	37	0	−1
1TBQ	K	38	0	−1
1TBQ	K	39	1	−1
1TBQ	K	40	1	−1
1TBQ	K	41	0	−1
1TBQ	K	42	1	−1
1TBQ	K	43	1	−1
1TBQ	K	44	0	−1
1TBQ	K	45	0	−1
1TBQ	K	46	1	−1
1TBQ	K	47	1	−1
1TBQ	K	48	0	−1
1TBQ	K	49	1	−1
1TBQ	K	1	0	−1
1TBQ	K	2	0	−1
1TBQ	K	3	1	−1
1TBQ	K	4	1	−1
1TBQ	K	5	1	−1
1TBQ	K	6	1	−1
1TBQ	K	7	0	−1
1TBQ	K	8	0	−1
1TBQ	K	9	1	−1
1TBQ	K	10	0	−1
1TBQ	K	11	0	−1

1TBQ	K	12	0	−1
1TBQ	K	13	0	−1
1TBQ	K	14	0	−1
1TBQ	K	15	0	−1
1TBQ	K	16	0	−1
1TBQ	K	17	0	−1
1TBQ	K	18	0	−1
1TBQ	K	19	0	−1
1TBQ	K	20	1	1
1TBQ	K	21	1	1
1TBQ	K	22	1	1
1TBQ	K	23	1	1
1TBQ	K	24	1	1
1TBQ	K	25	1	1
1TBQ	K	26	1	1
1TBQ	K	27	0	−1
1TBQ	K	28	0	−1
1TBQ	K	29	0	−1
1TBQ	K	30	0	−1
1TBQ	K	31	0	−1
1TBQ	K	32	0	−1
1TBQ	K	33	0	−1
1TBQ	K	34	0	−1
1TBQ	K	35	0	−1
1TBQ	K	36	0	−1
1TBQ	K	37	0	−1
1TBQ	K	38	0	−1
1TBQ	K	39	0	−1
1TBQ	K	40	0	−1
1TBQ	K	41	0	−1
1TBQ	K	42	0	−1
1TBQ	K	43	0	−1
1TBQ	K	44	0	−1
1TBQ	K	45	0	−1
1TBQ	K	46	0	−1
1TBQ	K	47	0	−1
1TBQ	K	48	1	−1
1TBQ	K	49	1	−1
1TBQ	K	50	1	1
1TBQ	K	51	1	−1
1TBQ	K	52	0	−1
1TBQ	K	53	1	−1
1TBQ	K	54	0	−1
1TBQ	K	55	1	−1
1TBQ	K	56	1	−1
1TBQ	K	57	1	−1
1TBQ	K	58	1	−1
1TBQ	K	59	0	−1
1TBQ	K	60	1	1
1TBQ	K	61	0	−1
1TBQ	K	62	0	−1
1TBQ	K	63	0	−1

1TBQ	K	64	0	−1
1TBQ	K	65	0	−1
1TBQ	K	66	0	−1
1TBQ	K	67	0	−1
1TBQ	K	68	0	−1
1TBQ	K	69	1	1
1TBQ	K	70	1	1
1TBQ	K	71	1	1
1TBQ	K	72	0	−1
1TBQ	K	73	1	1
1TBQ	K	74	1	1
1TBQ	K	75	0	−1
1TBQ	K	76	0	−1
1TBQ	K	77	1	−1
1TBQ	K	78	1	1
1TBQ	K	79	0	−1
1TBQ	K	80	1	1
1TBQ	K	81	0	−1
1TBQ	K	82	1	−1
1TBQ	K	83	1	−1
1TBQ	K	84	0	−1
1TBQ	K	85	1	−1
1TBQ	K	86	1	−1
1TBQ	K	87	0	−1
1TBQ	K	88	1	−1
1TBQ	K	89	1	−1
1TBQ	K	90	0	−1
1TBQ	K	91	0	−1
1TBQ	K	92	1	−1
1TBQ	K	93	1	−1
1TBQ	K	94	1	−1
1TBQ	K	95	0	−1
1TBQ	K	96	0	−1
1TBQ	K	97	0	−1
1TBQ	K	98	0	−1
1TBQ	K	99	0	−1
1TBQ	K	100	0	−1
1TBQ	K	101	0	−1
1TBQ	K	102	0	−1
1TBQ	K	103	0	−1
1TBQ	K	104	0	−1
1TBQ	K	105	0	−1
1TBQ	K	106	1	1
1TBQ	K	107	1	1
1TBQ	K	108	1	−1
1TBQ	K	109	0	−1
1TBQ	K	110	1	−1
1TBQ	K	111	1	−1
1TBQ	K	112	1	−1
1TBQ	K	113	1	−1
1TBQ	K	114	1	−1
1TBQ	K	115	0	−1

1TBQ	K	116	0	−1
1TBQ	K	117	0	−1
1TBQ	K	118	0	−1
1TBQ	K	119	0	−1
1TBQ	K	120	0	−1
1TBQ	K	121	0	−1
1TBQ	K	122	0	−1
1TBQ	K	123	1	−1
1TBQ	K	124	1	−1
1TBQ	K	125	0	−1
1TBQ	K	126	0	−1
1TBQ	K	127	1	−1
1TBQ	K	128	1	−1
1TBQ	K	129	0	−1
1TBQ	K	130	0	−1
1TBQ	K	131	1	−1
1TBQ	K	132	1	−1
1TBQ	K	133	0	−1
1TBQ	K	134	0	−1
1TBQ	K	135	0	−1
1TBQ	K	136	0	−1
1TBQ	K	137	0	−1
1TBQ	K	138	0	−1
1TBQ	K	139	0	−1
1TBQ	K	140	0	−1
1TBQ	K	141	0	−1
1TBQ	K	142	0	−1
1TBQ	K	143	0	−1
1TBQ	K	144	1	−1
1TBQ	K	145	0	−1
1TBQ	K	146	0	−1
1TBQ	K	147	1	1
1TBQ	K	148	1	1
1TBQ	K	149	1	1
1TBQ	K	150	1	−1
1TBQ	K	151	1	−1
1TBQ	K	152	1	−1
1TBQ	K	153	1	−1
1TBQ	K	154	1	1
1TBQ	K	155	0	−1
1TBQ	K	156	1	1
1TBQ	K	157	0	−1
1TBQ	K	158	1	−1
1TBQ	K	159	0	−1
1TBQ	K	160	0	−1
1TBQ	K	161	0	−1
1TBQ	K	162	0	−1
1TBQ	K	163	0	−1
1TBQ	K	164	0	−1
1TBQ	K	165	0	−1
1TBQ	K	166	0	−1
1TBQ	K	167	0	−1

1TBQ	K	168	0	−1
1TBQ	K	169	1	−1
1TBQ	K	170	1	−1
1TBQ	K	171	1	−1
1TBQ	K	172	0	−1
1TBQ	K	173	0	−1
1TBQ	K	174	1	−1
1TBQ	K	175	1	−1
1TBQ	K	176	0	−1
1TBQ	K	177	0	−1
1TBQ	K	178	1	1
1TBQ	K	179	1	1
1TBQ	K	180	1	−1
1TBQ	K	181	0	−1
1TBQ	K	182	0	−1
1TBQ	K	183	1	−1
1TBQ	K	184	0	−1
1TBQ	K	185	0	−1
1TBQ	K	186	0	−1
1TBQ	K	187	0	−1
1TBQ	K	188	0	−1
1TBQ	K	189	0	−1
1TBQ	K	190	0	−1
1TBQ	K	191	1	−1
1TBQ	K	192	1	−1
1TBQ	K	193	1	−1
1TBQ	K	194	1	−1
1TBQ	K	195	1	−1
1TBQ	K	196	1	−1
1TBQ	K	197	1	−1
1TBQ	K	198	0	−1
1TBQ	K	199	0	−1
1TBQ	K	200	0	−1
1TBQ	K	201	0	−1
1TBQ	K	202	1	1
1TBQ	K	203	0	−1
1TBQ	K	204	0	−1
1TBQ	K	205	0	−1
1TBQ	K	206	0	−1
1TBQ	K	207	0	−1
1TBQ	K	208	0	−1
1TBQ	K	209	0	−1
1TBQ	K	210	0	−1
1TBQ	K	211	0	−1
1TBQ	K	212	0	−1
1TBQ	K	213	0	−1
1TBQ	K	214	1	−1
1TBQ	K	215	1	−1
1TBQ	K	216	1	−1
1TBQ	K	217	1	−1
1TBQ	K	218	0	−1
1TBQ	K	219	0	−1

1TBQ	K	220	0	−1
1TBQ	K	221	0	−1
1TBQ	K	222	0	−1
1TBQ	K	223	0	−1
1TBQ	K	224	0	−1
1TBQ	K	225	0	−1
1TBQ	K	226	0	−1
1TBQ	K	227	0	−1
1TBQ	K	228	1	1
1TBQ	K	229	1	1
1TBQ	K	230	1	1
1TBQ	K	231	0	−1
1TBQ	K	232	0	−1
1TBQ	K	233	1	1
1TBQ	K	234	1	−1
1TBQ	K	235	1	−1
1TBQ	K	236	1	1
1TBQ	K	237	0	−1
1TBQ	K	238	0	−1
1TBQ	K	239	0	−1
1TBQ	K	240	0	−1
1TBQ	K	241	0	−1
1TBQ	K	242	0	−1
1TBQ	K	243	0	−1
1TBQ	K	244	1	−1
1TBQ	K	245	1	−1
1TBQ	K	246	0	−1
1TBQ	K	247	0	−1
1TBQ	K	248	1	−1
1TBQ	K	249	0	−1
1TBQ	K	250	0	−1
1TBQ	K	251	1	−1
1TBQ	K	252	1	−1
1TBQ	K	253	0	−1
1TBQ	K	254	0	−1
1TBQ	K	255	1	−1
1TBQ	K	256	1	−1
1TBQ	K	257	1	−1
1TBQ	K	258	1	−1
1TBQ	K	259	1	−1
1TBQ	K	1	1	−1
1TBQ	K	2	1	−1
1TBQ	K	3	1	−1
1TBQ	K	4	1	−1
1TBQ	K	5	1	−1
1TBQ	K	6	0	−1
1TBQ	K	7	1	−1
1TBQ	K	8	1	−1
1TBQ	K	9	1	−1
1TBQ	K	10	0	−1
1TBQ	K	11	1	−1
1TBQ	K	12	1	−1

1TBQ	K	13	1	−1
1TBQ	K	14	0	−1
1TBQ	K	15	0	−1
1TBQ	K	16	0	−1
1TBQ	K	17	1	−1
1TBQ	K	18	0	−1
1TBQ	K	19	0	−1
1TBQ	K	20	1	−1
1TBQ	K	21	1	−1
1TBQ	K	22	0	−1
1TBQ	K	23	0	−1
1TBQ	K	24	0	−1
1TBQ	K	25	0	−1
1TBQ	K	26	0	−1
1TBQ	K	27	0	−1
1TBQ	K	28	0	−1
1TBQ	K	29	0	−1
1TBQ	K	30	1	−1
1TBQ	K	31	1	−1
1TBQ	K	32	1	−1
1TBQ	K	33	1	−1
1TBQ	K	34	1	−1
1TBQ	K	35	0	−1
1TBQ	K	36	1	−1
1TBQ	K	37	0	−1
1TBQ	K	38	0	−1
1TBQ	K	39	1	−1
1TBQ	K	40	0	−1
1TBQ	K	41	0	−1
1TBQ	K	42	1	−1
1TBQ	K	43	1	−1
1TBQ	K	44	0	−1
1TBQ	K	45	0	−1
1TBQ	K	46	1	−1
1TBQ	K	47	1	−1
1TBQ	K	48	1	−1
1TBQ	K	49	1	−1
1TBQ	K	1	0	−1
1TBQ	K	2	0	−1
1TBQ	K	3	1	−1
1TBQ	K	4	1	−1
1TBQ	K	5	1	−1
1TBQ	K	6	1	−1
1TBQ	K	7	0	−1
1TBQ	K	8	0	−1
1TBQ	K	9	1	−1
1TBQ	K	10	0	−1
1TBQ	K	11	0	−1
1TBQ	K	12	0	−1
1TBQ	K	13	0	−1
1TBQ	K	14	0	−1
1TBQ	K	15	0	−1

1TBQ	K	16	0	−1
1TBQ	K	17	0	−1
1TBQ	K	18	0	−1
1TBQ	K	19	0	−1
1TBQ	K	20	1	1
1TBQ	K	21	1	1
1TBQ	K	22	1	1
1TBQ	K	23	1	−1
1TBQ	K	24	1	1
1TBQ	K	25	1	1
1TBQ	K	26	0	−1
1TBQ	K	27	0	−1
1TBQ	K	28	0	−1
1TBQ	K	29	0	−1
1TBQ	K	30	0	−1
1TBQ	K	31	0	−1
1TBQ	K	32	0	−1
1TBQ	K	33	0	−1
1TBQ	K	34	0	−1
1TBQ	K	35	0	−1
1TBQ	K	36	0	−1
1TBQ	K	37	0	−1
1TBQ	K	38	0	−1
1TBQ	K	39	0	−1
1TBQ	K	40	0	−1
1TBQ	K	41	0	−1
1TBQ	K	42	0	−1
1TBQ	K	43	0	−1
1TBQ	K	44	0	−1
1TBQ	K	45	0	−1
1TBQ	K	46	1	−1
1TBQ	K	47	0	−1
1TBQ	K	48	1	−1
1TBQ	K	49	1	−1
1TBQ	K	50	1	1
1TBQ	K	51	1	−1
1TBQ	K	52	0	−1
1TBQ	K	53	1	−1
1TBQ	K	54	0	−1
1TBQ	K	55	1	−1
1TBQ	K	56	1	−1
1TBQ	K	57	1	−1
1TBQ	K	58	1	−1
1TBQ	K	59	0	−1
1TBQ	K	60	1	1
1TBQ	K	61	0	−1
1TBQ	K	62	0	−1
1TBQ	K	63	0	−1
1TBQ	K	64	0	−1
1TBQ	K	65	0	−1
1TBQ	K	66	0	−1
1TBQ	K	67	0	−1

1TBQ	K	68	0	−1
1TBQ	K	69	1	1
1TBQ	K	70	1	1
1TBQ	K	71	1	1
1TBQ	K	72	0	−1
1TBQ	K	73	1	1
1TBQ	K	74	1	1
1TBQ	K	75	0	−1
1TBQ	K	76	0	−1
1TBQ	K	77	1	1
1TBQ	K	78	1	1
1TBQ	K	79	0	−1
1TBQ	K	80	1	1
1TBQ	K	81	0	−1
1TBQ	K	82	1	−1
1TBQ	K	83	1	−1
1TBQ	K	84	0	−1
1TBQ	K	85	0	−1
1TBQ	K	86	1	−1
1TBQ	K	87	0	−1
1TBQ	K	88	1	−1
1TBQ	K	89	1	−1
1TBQ	K	90	0	−1
1TBQ	K	91	1	−1
1TBQ	K	92	1	−1
1TBQ	K	93	1	−1
1TBQ	K	94	1	−1
1TBQ	K	95	0	−1
1TBQ	K	96	0	−1
1TBQ	K	97	0	−1
1TBQ	K	98	0	−1
1TBQ	K	99	0	−1
1TBQ	K	100	0	−1
1TBQ	K	101	0	−1
1TBQ	K	102	0	−1
1TBQ	K	103	0	−1
1TBQ	K	104	0	−1
1TBQ	K	105	0	−1
1TBQ	K	106	1	1
1TBQ	K	107	1	−1
1TBQ	K	108	1	−1
1TBQ	K	109	0	−1
1TBQ	K	110	1	−1
1TBQ	K	111	1	−1
1TBQ	K	112	1	−1
1TBQ	K	113	1	−1
1TBQ	K	114	1	−1
1TBQ	K	115	0	−1
1TBQ	K	116	0	−1
1TBQ	K	117	0	−1
1TBQ	K	118	0	−1
1TBQ	K	119	0	−1

1TBQ	K	120	0	−1
1TBQ	K	121	0	−1
1TBQ	K	122	1	−1
1TBQ	K	123	1	−1
1TBQ	K	124	1	−1
1TBQ	K	125	0	−1
1TBQ	K	126	0	−1
1TBQ	K	127	1	−1
1TBQ	K	128	1	−1
1TBQ	K	129	0	−1
1TBQ	K	130	1	−1
1TBQ	K	131	1	−1
1TBQ	K	132	0	−1
1TBQ	K	133	1	−1
1TBQ	K	134	0	−1
1TBQ	K	135	0	−1
1TBQ	K	136	0	−1
1TBQ	K	137	0	−1
1TBQ	K	138	0	−1
1TBQ	K	139	0	−1
1TBQ	K	140	0	−1
1TBQ	K	141	0	−1
1TBQ	K	142	0	−1
1TBQ	K	143	0	−1
1TBQ	K	144	1	−1
1TBQ	K	145	1	−1
1TBQ	K	146	1	1
1TBQ	K	147	1	1
1TBQ	K	148	1	1
1TBQ	K	149	1	−1
1TBQ	K	150	1	1
1TBQ	K	151	1	−1
1TBQ	K	152	1	−1
1TBQ	K	153	1	−1
1TBQ	K	154	1	1
1TBQ	K	155	0	−1
1TBQ	K	156	1	1
1TBQ	K	157	0	−1
1TBQ	K	158	1	−1
1TBQ	K	159	0	−1
1TBQ	K	160	0	−1
1TBQ	K	161	0	−1
1TBQ	K	162	0	−1
1TBQ	K	163	0	−1
1TBQ	K	164	0	−1
1TBQ	K	165	0	−1
1TBQ	K	166	0	−1
1TBQ	K	167	0	−1
1TBQ	K	168	0	−1
1TBQ	K	169	1	−1
1TBQ	K	170	1	−1
1TBQ	K	171	1	−1

1TBQ	K	172	1	−1
1TBQ	K	173	0	−1
1TBQ	K	174	1	−1
1TBQ	K	175	1	−1
1TBQ	K	176	0	−1
1TBQ	K	177	0	−1
1TBQ	K	178	1	1
1TBQ	K	179	1	1
1TBQ	K	180	1	−1
1TBQ	K	181	0	−1
1TBQ	K	182	0	−1
1TBQ	K	183	1	−1
1TBQ	K	184	0	−1
1TBQ	K	185	0	−1
1TBQ	K	186	0	−1
1TBQ	K	187	0	−1
1TBQ	K	188	0	−1
1TBQ	K	189	0	−1
1TBQ	K	190	0	−1
1TBQ	K	191	1	−1
1TBQ	K	192	1	−1
1TBQ	K	193	1	−1
1TBQ	K	194	1	−1
1TBQ	K	195	1	−1
1TBQ	K	196	1	−1
1TBQ	K	197	1	−1
1TBQ	K	198	0	−1
1TBQ	K	199	0	−1
1TBQ	K	200	0	−1
1TBQ	K	201	0	−1
1TBQ	K	202	1	1
1TBQ	K	203	1	1
1TBQ	K	204	0	−1
1TBQ	K	205	0	−1
1TBQ	K	206	0	−1
1TBQ	K	207	0	−1
1TBQ	K	208	0	−1
1TBQ	K	209	0	−1
1TBQ	K	210	0	−1
1TBQ	K	211	0	−1
1TBQ	K	212	0	−1
1TBQ	K	213	0	−1
1TBQ	K	214	1	−1
1TBQ	K	215	1	−1
1TBQ	K	216	1	−1
1TBQ	K	217	1	−1
1TBQ	K	218	0	−1
1TBQ	K	219	0	−1
1TBQ	K	220	0	−1
1TBQ	K	221	0	−1
1TBQ	K	222	0	−1
1TBQ	K	223	0	−1

1TBQ	K	224	0	−1
1TBQ	K	225	0	−1
1TBQ	K	226	0	−1
1TBQ	K	227	0	−1
1TBQ	K	228	1	1
1TBQ	K	229	1	1
1TBQ	K	230	1	1
1TBQ	K	231	0	−1
1TBQ	K	232	0	−1
1TBQ	K	233	1	1
1TBQ	K	234	1	−1
1TBQ	K	235	1	−1
1TBQ	K	236	1	1
1TBQ	K	237	0	−1
1TBQ	K	238	0	−1
1TBQ	K	239	0	−1
1TBQ	K	240	0	−1
1TBQ	K	241	0	−1
1TBQ	K	242	0	−1
1TBQ	K	243	0	−1
1TBQ	K	244	0	−1
1TBQ	K	245	1	−1
1TBQ	K	246	0	−1
1TBQ	K	247	0	−1
1TBQ	K	248	1	−1
1TBQ	K	249	0	−1
1TBQ	K	250	0	−1
1TBQ	K	251	1	−1
1TBQ	K	252	1	−1
1TBQ	K	253	0	−1
1TBQ	K	254	0	−1
1TBQ	K	255	0	−1
1TBQ	K	256	1	−1
1TBQ	K	257	1	−1
1TBQ	K	258	0	−1
1TBQ	K	259	1	−1
1TBQ	R	1	1	1
1TBQ	R	2	1	1
1TBQ	R	3	1	1
1TBQ	R	4	1	1
1TBQ	R	5	1	−1
1TBQ	R	6	1	1
1TBQ	R	7	1	1
1TBQ	R	8	1	1
1TBQ	R	9	1	1
1TBQ	R	10	1	1
1TBQ	R	11	1	1
1TBQ	R	12	1	1
1TBQ	R	13	1	1
1TBQ	R	14	1	1
1TBQ	R	15	0	−1
1TBQ	R	16	0	−1

1TBQ	R	17	0	−1
1TBQ	R	18	1	−1
1TBQ	R	19	1	−1
1TBQ	R	20	1	−1
1TBQ	R	21	1	1
1TBQ	R	22	1	1
1TBQ	R	23	0	−1
1TBQ	R	24	1	1
1TBQ	R	25	0	−1
1TBQ	R	26	1	1
1TBQ	R	27	1	1
1TBQ	R	28	1	1
1TBQ	R	29	0	−1
1TBQ	R	30	1	−1
1TBQ	R	31	0	−1
1TBQ	R	32	0	−1
1TBQ	R	33	1	−1
1TBQ	R	34	1	−1
1TBQ	R	35	1	1
1TBQ	R	36	1	−1
1TBQ	R	37	1	−1
1TBQ	R	38	1	−1
1TBQ	R	39	1	−1
1TBQ	R	40	0	−1
1TBQ	R	41	1	−1
1TBQ	R	42	1	−1
1TBQ	R	43	1	−1
1TBQ	R	44	1	−1
1TBQ	R	45	1	1
1TBQ	R	46	1	1
1TBQ	R	47	0	−1
1TBQ	R	48	1	−1
1TBQ	R	49	1	−1
1TBQ	R	50	1	−1
1TBQ	R	51	1	1
1TBQ	R	52	1	1
1TBQ	R	53	1	−1
1TBQ	R	54	1	1
1TBQ	R	55	1	1
1TBQ	R	56	1	1
1TBQ	R	57	0	−1
1TBQ	R	58	1	1
1TBQ	R	59	1	1
1TBQ	R	60	0	−1
1TBQ	R	61	1	−1
1TBQ	R	62	1	−1
1TBQ	R	63	1	1
1TBQ	R	64	1	−1
1TBQ	R	65	1	−1
1TBQ	R	66	1	−1
1TBQ	R	67	0	−1
1TBQ	R	68	0	−1

1TBQ	R	69	0	−1
1TBQ	R	70	0	−1
1TBQ	R	71	1	−1
1TBQ	R	72	1	−1
1TBQ	R	73	1	−1
1TBQ	R	74	1	−1
1TBQ	R	75	1	−1
1TBQ	R	76	0	−1
1TBQ	R	77	1	−1
1TBQ	R	78	0	−1
1TBQ	R	79	1	−1
1TBQ	R	80	1	1
1TBQ	R	81	1	−1
1TBQ	R	82	0	−1
1TBQ	R	83	1	1
1TBQ	R	84	0	−1
1TBQ	R	85	0	−1
1TBQ	R	86	1	1
1TBQ	R	87	1	1
1TBQ	R	88	1	1
1TBQ	R	89	1	−1
1TBQ	R	90	0	−1
1TBQ	R	91	1	1
1TBQ	R	92	1	1
1TBQ	R	93	0	−1
1TBQ	R	94	1	1
1TBQ	R	95	1	−1
1TBQ	R	96	1	−1
1TBQ	R	97	0	−1
1TBQ	R	98	1	−1
1TBQ	R	99	0	−1
1TBQ	R	100	1	−1
1TBQ	R	101	0	−1
1TBQ	R	102	1	−1
1TBQ	R	103	1	−1
1TBQ	S	1	1	1
1TBQ	S	2	1	1
1TBQ	S	3	1	1
1TBQ	S	4	1	1
1TBQ	S	5	0	−1
1TBQ	S	6	1	1
1TBQ	S	7	1	1
1TBQ	S	8	1	1
1TBQ	S	9	1	1
1TBQ	S	10	1	1
1TBQ	S	11	1	1
1TBQ	S	12	1	1
1TBQ	S	13	1	1
1TBQ	S	14	1	1
1TBQ	S	15	0	−1
1TBQ	S	16	0	−1
1TBQ	S	17	0	−1

1TBQ	S	18	1	−1
1TBQ	S	19	1	−1
1TBQ	S	20	1	−1
1TBQ	S	21	1	1
1TBQ	S	22	1	1
1TBQ	S	23	0	−1
1TBQ	S	24	1	1
1TBQ	S	25	0	−1
1TBQ	S	26	1	1
1TBQ	S	27	1	1
1TBQ	S	28	1	1
1TBQ	S	29	0	−1
1TBQ	S	30	1	−1
1TBQ	S	31	0	−1
1TBQ	S	32	0	−1
1TBQ	S	33	1	−1
1TBQ	S	34	1	−1
1TBQ	S	35	1	1
1TBQ	S	36	1	−1
1TBQ	S	37	1	−1
1TBQ	S	38	1	−1
1TBQ	S	39	1	−1
1TBQ	S	40	0	−1
1TBQ	S	41	1	−1
1TBQ	S	42	1	−1
1TBQ	S	43	1	−1
1TBQ	S	44	1	−1
1TBQ	S	45	1	1
1TBQ	S	46	1	1
1TBQ	S	47	0	−1
1TBQ	S	48	1	−1
1TBQ	S	49	1	−1
1TBQ	S	50	1	−1
1TBQ	S	51	1	1
1TBQ	S	52	1	1
1TBQ	S	53	1	−1
1TBQ	S	54	1	1
1TBQ	S	55	1	1
1TBQ	S	56	1	1
1TBQ	S	57	0	−1
1TBQ	S	58	1	1
1TBQ	S	59	1	1
1TBQ	S	60	0	−1
1TBQ	S	61	1	−1
1TBQ	S	62	1	−1
1TBQ	S	63	1	1
1TBQ	S	64	1	−1
1TBQ	S	65	1	−1
1TBQ	S	66	1	−1
1TBQ	S	67	0	−1
1TBQ	S	68	0	−1
1TBQ	S	69	0	−1

1TBQ	S	70	0	−1
1TBQ	S	71	1	−1
1TBQ	S	72	1	−1
1TBQ	S	73	1	−1
1TBQ	S	74	1	−1
1TBQ	S	75	1	−1
1TBQ	S	76	0	−1
1TBQ	S	77	1	−1
1TBQ	S	78	0	−1
1TBQ	S	79	1	−1
1TBQ	S	80	1	1
1TBQ	S	81	1	−1
1TBQ	S	82	0	−1
1TBQ	S	83	1	1
1TBQ	S	84	0	−1
1TBQ	S	85	0	−1
1TBQ	S	86	0	−1
1TBQ	S	87	1	1
1TBQ	S	88	1	1
1TBQ	S	89	1	−1
1TBQ	S	90	0	−1
1TBQ	S	91	1	1
1TBQ	S	92	1	1
1TBQ	S	93	0	−1
1TBQ	S	94	1	1
1TBQ	S	95	1	−1
1TBQ	S	96	1	−1
1TBQ	S	97	1	−1
1TBQ	S	98	1	−1
1TBQ	S	99	0	−1
1TBQ	S	100	1	−1
1TBQ	S	101	0	−1
1TBQ	S	102	1	−1
1TBQ	S	103	1	−1
1TGS	I	1	1	−1
1TGS	I	2	1	−1
1TGS	I	3	1	−1
1TGS	I	4	1	−1
1TGS	I	5	1	−1
1TGS	I	6	1	−1
1TGS	I	7	0	−1
1TGS	I	8	1	−1
1TGS	I	9	1	−1
1TGS	I	10	1	1
1TGS	I	11	1	1
1TGS	I	12	1	1
1TGS	I	13	1	1
1TGS	I	14	1	1
1TGS	I	15	1	1
1TGS	I	16	1	1
1TGS	I	17	1	1
1TGS	I	18	1	1

1TGS	I	19	1	1
1TGS	I	20	1	1
1TGS	I	21	1	1
1TGS	I	22	0	−1
1TGS	I	23	0	−1
1TGS	I	24	0	−1
1TGS	I	25	0	−1
1TGS	I	26	1	−1
1TGS	I	27	1	−1
1TGS	I	28	1	−1
1TGS	I	29	1	1
1TGS	I	30	1	1
1TGS	I	31	0	−1
1TGS	I	32	1	1
1TGS	I	33	0	−1
1TGS	I	34	0	−1
1TGS	I	35	0	−1
1TGS	I	36	1	1
1TGS	I	37	0	−1
1TGS	I	38	0	−1
1TGS	I	39	1	−1
1TGS	I	40	1	−1
1TGS	I	41	0	−1
1TGS	I	42	1	−1
1TGS	I	43	1	1
1TGS	I	44	1	−1
1TGS	I	45	1	−1
1TGS	I	46	1	−1
1TGS	I	47	1	−1
1TGS	I	48	0	−1
1TGS	I	49	0	−1
1TGS	I	50	0	−1
1TGS	I	51	1	−1
1TGS	I	52	1	−1
1TGS	I	53	1	−1
1TGS	I	54	1	−1
1TGS	I	55	1	−1
1TGS	I	56	1	−1
1TGS	Z	5	1	−1
1TGS	Z	6	1	−1
1TGS	Z	7	0	−1
1TGS	Z	8	0	−1
1TGS	Z	9	0	−1
1TGS	Z	10	0	−1
1TGS	Z	11	1	−1
1TGS	Z	12	1	−1
1TGS	Z	13	0	−1
1TGS	Z	14	1	−1
1TGS	Z	15	0	−1
1TGS	Z	16	1	−1
1TGS	Z	17	1	−1
1TGS	Z	18	0	−1

1TGS	Z	19	0	−1
1TGS	Z	20	0	−1
1TGS	Z	21	0	−1
1TGS	Z	22	0	−1
1TGS	Z	23	0	−1
1TGS	Z	24	0	−1
1TGS	Z	25	0	−1
1TGS	Z	26	1	−1
1TGS	Z	27	1	−1
1TGS	Z	28	1	1
1TGS	Z	29	0	−1
1TGS	Z	30	0	−1
1TGS	Z	31	0	−1
1TGS	Z	32	0	−1
1TGS	Z	33	0	−1
1TGS	Z	34	0	−1
1TGS	Z	35	0	−1
1TGS	Z	36	0	−1
1TGS	Z	37	1	−1
1TGS	Z	38	1	−1
1TGS	Z	39	1	−1
1TGS	Z	40	0	−1
1TGS	Z	41	0	−1
1TGS	Z	42	0	−1
1TGS	Z	43	0	−1
1TGS	Z	44	0	−1
1TGS	Z	45	0	−1
1TGS	Z	46	1	1
1TGS	Z	47	0	−1
1TGS	Z	48	1	−1
1TGS	Z	49	1	1
1TGS	Z	50	1	−1
1TGS	Z	51	1	−1
1TGS	Z	52	0	−1
1TGS	Z	53	1	−1
1TGS	Z	54	0	−1
1TGS	Z	55	0	−1
1TGS	Z	56	0	−1
1TGS	Z	57	0	−1
1TGS	Z	58	0	−1
1TGS	Z	59	0	−1
1TGS	Z	60	1	−1
1TGS	Z	61	0	−1
1TGS	Z	62	1	−1
1TGS	Z	63	1	−1
1TGS	Z	64	1	−1
1TGS	Z	65	1	−1
1TGS	Z	66	1	−1
1TGS	Z	67	1	−1
1TGS	Z	68	0	−1
1TGS	Z	69	0	−1
1TGS	Z	70	1	−1

1TGS	Z	71	0	−1
1TGS	Z	72	1	−1
1TGS	Z	73	0	−1
1TGS	Z	74	1	−1
1TGS	Z	75	1	−1
1TGS	Z	76	1	−1
1TGS	Z	77	0	−1
1TGS	Z	78	1	−1
1TGS	Z	79	0	−1
1TGS	Z	80	1	−1
1TGS	Z	81	1	−1
1TGS	Z	82	0	−1
1TGS	Z	83	1	−1
1TGS	Z	84	1	1
1TGS	Z	85	1	1
1TGS	Z	86	1	1
1TGS	Z	87	0	−1
1TGS	Z	88	0	−1
1TGS	Z	89	0	−1
1TGS	Z	90	0	−1
1TGS	Z	91	0	−1
1TGS	Z	92	0	−1
1TGS	Z	93	0	−1
1TGS	Z	94	0	−1
1TGS	Z	95	0	−1
1TGS	Z	96	0	−1
1TGS	Z	97	1	−1
1TGS	Z	98	1	−1
1TGS	Z	99	1	−1
1TGS	Z	100	0	−1
1TGS	Z	101	1	−1
1TGS	Z	102	1	−1
1TGS	Z	103	1	−1
1TGS	Z	104	1	−1
1TGS	Z	105	1	−1
1TGS	Z	106	0	−1
1TGS	Z	107	1	−1
1TGS	Z	108	1	−1
1TGS	Z	109	0	−1
1TGS	Z	110	1	−1
1TGS	Z	111	0	−1
1TGS	Z	112	0	−1
1TGS	Z	113	1	−1
1TGS	Z	114	1	−1
1TGS	Z	115	1	−1
1TGS	Z	116	1	−1
1TGS	Z	117	1	−1
1TGS	Z	118	1	−1
1TGS	Z	119	1	−1
1TGS	Z	120	1	−1
1TGS	Z	121	1	−1
1TGS	Z	122	0	−1

1TGS	Z	123	0	−1
1TGS	Z	124	0	−1
1TGS	Z	125	0	−1
1TGS	Z	126	0	−1
1TGS	Z	127	0	−1
1TGS	Z	128	0	−1
1TGS	Z	129	0	−1
1TGS	Z	130	0	−1
1TGS	Z	131	1	−1
1TGS	Z	132	1	−1
1TGS	Z	133	1	1
1TGS	Z	134	1	−1
1TGS	Z	135	1	1
1TGS	Z	136	1	1
1TGS	Z	137	1	1
1TGS	Z	138	1	1
1TGS	Z	139	0	−1
1TGS	Z	140	1	−1
1TGS	Z	141	0	−1
1TGS	Z	142	0	−1
1TGS	Z	143	0	−1
1TGS	Z	144	0	−1
1TGS	Z	145	1	−1
1TGS	Z	146	0	−1
1TGS	Z	147	0	−1
1TGS	Z	148	0	−1
1TGS	Z	149	0	−1
1TGS	Z	150	1	−1
1TGS	Z	151	1	−1
1TGS	Z	152	1	−1
1TGS	Z	153	0	−1
1TGS	Z	154	0	−1
1TGS	Z	155	1	−1
1TGS	Z	156	1	−1
1TGS	Z	157	0	−1
1TGS	Z	158	0	−1
1TGS	Z	159	1	−1
1TGS	Z	160	1	−1
1TGS	Z	161	1	1
1TGS	Z	162	0	−1
1TGS	Z	163	0	−1
1TGS	Z	164	1	−1
1TGS	Z	165	0	−1
1TGS	Z	166	0	−1
1TGS	Z	167	0	−1
1TGS	Z	168	0	−1
1TGS	Z	169	0	−1
1TGS	Z	170	0	−1
1TGS	Z	171	1	−1
1TGS	Z	172	1	−1
1TGS	Z	173	1	−1
1TGS	Z	174	0	−1

1TGS	Z	175	1	−1
1TGS	Z	176	1	−1
1TGS	Z	177	0	−1
1TGS	Z	178	0	−1
1TGS	Z	179	0	−1
1TGS	Z	180	1	1
1TGS	Z	181	0	−1
1TGS	Z	182	0	−1
1TGS	Z	183	0	−1
1TGS	Z	184	0	−1
1TGS	Z	185	0	−1
1TGS	Z	186	0	−1
1TGS	Z	187	0	−1
1TGS	Z	188	0	−1
1TGS	Z	189	0	−1
1TGS	Z	190	1	−1
1TGS	Z	191	1	−1
1TGS	Z	192	1	−1
1TGS	Z	193	0	−1
1TGS	Z	194	0	−1
1TGS	Z	195	0	−1
1TGS	Z	196	0	−1
1TGS	Z	197	0	−1
1TGS	Z	198	0	−1
1TGS	Z	199	0	−1
1TGS	Z	200	1	1
1TGS	Z	201	1	1
1TGS	Z	202	1	1
1TGS	Z	203	0	−1
1TGS	Z	204	0	−1
1TGS	Z	205	1	1
1TGS	Z	206	1	−1
1TGS	Z	207	1	−1
1TGS	Z	208	1	−1
1TGS	Z	209	0	−1
1TGS	Z	210	0	−1
1TGS	Z	211	0	−1
1TGS	Z	212	0	−1
1TGS	Z	213	0	−1
1TGS	Z	214	1	−1
1TGS	Z	215	0	−1
1TGS	Z	216	0	−1
1TGS	Z	217	1	−1
1TGS	Z	218	0	−1
1TGS	Z	219	0	−1
1TGS	Z	220	1	−1
1TGS	Z	221	0	−1
1TGS	Z	222	0	−1
1TGS	Z	223	1	−1
1TGS	Z	224	1	−1
1TGS	Z	225	0	−1
1TGS	Z	226	0	−1

1TGS	Z	227	1	−1
1TGS	Z	228	1	−1
1TGS	Z	229	1	−1
1TOC	D	20	1	−1
1TOC	D	21	1	−1
1TOC	D	22	0	−1
1TOC	D	23	0	−1
1TOC	D	24	0	−1
1TOC	D	25	0	−1
1TOC	D	26	0	−1
1TOC	D	27	0	−1
1TOC	D	28	0	−1
1TOC	D	29	0	−1
1TOC	D	30	1	−1
1TOC	D	31	1	−1
1TOC	D	32	1	−1
1TOC	D	33	1	−1
1TOC	D	34	1	−1
1TOC	D	35	0	−1
1TOC	D	36	1	−1
1TOC	D	37	0	−1
1TOC	D	38	0	−1
1TOC	D	39	1	−1
1TOC	D	40	1	−1
1TOC	D	41	0	−1
1TOC	D	42	1	−1
1TOC	D	43	1	−1
1TOC	D	44	0	−1
1TOC	D	45	0	−1
1TOC	D	46	1	−1
1TOC	D	47	1	−1
1TOC	D	1	0	−1
1TOC	D	2	0	−1
1TOC	D	3	1	1
1TOC	D	4	1	−1
1TOC	D	5	1	−1
1TOC	D	6	1	−1
1TOC	D	7	0	−1
1TOC	D	8	1	−1
1TOC	D	9	1	−1
1TOC	D	10	0	−1
1TOC	D	11	0	−1
1TOC	D	12	0	−1
1TOC	D	13	0	−1
1TOC	D	14	0	−1
1TOC	D	15	0	−1
1TOC	D	16	0	−1
1TOC	D	17	0	−1
1TOC	D	18	0	−1
1TOC	D	19	0	−1
1TOC	D	20	1	−1
1TOC	D	21	1	1

1TOC	D	22	1	1
1TOC	D	23	1	−1
1TOC	D	24	1	1
1TOC	D	25	1	1
1TOC	D	26	1	1
1TOC	D	27	0	−1
1TOC	D	28	0	−1
1TOC	D	29	0	−1
1TOC	D	30	0	−1
1TOC	D	31	0	−1
1TOC	D	32	0	−1
1TOC	D	33	0	−1
1TOC	D	34	0	−1
1TOC	D	35	1	−1
1TOC	D	36	1	−1
1TOC	D	37	0	−1
1TOC	D	38	0	−1
1TOC	D	39	0	−1
1TOC	D	40	0	−1
1TOC	D	41	0	−1
1TOC	D	42	0	−1
1TOC	D	43	0	−1
1TOC	D	44	0	−1
1TOC	D	45	0	−1
1TOC	D	46	0	−1
1TOC	D	47	0	−1
1TOC	D	48	1	−1
1TOC	D	49	1	1
1TOC	D	50	1	1
1TOC	D	51	1	1
1TOC	D	52	0	−1
1TOC	D	53	1	−1
1TOC	D	54	0	−1
1TOC	D	55	1	−1
1TOC	D	56	1	−1
1TOC	D	57	1	−1
1TOC	D	58	1	−1
1TOC	D	59	0	−1
1TOC	D	60	0	−1
1TOC	D	61	0	−1
1TOC	D	62	0	−1
1TOC	D	63	0	−1
1TOC	D	64	0	−1
1TOC	D	65	0	−1
1TOC	D	66	0	−1
1TOC	D	67	0	−1
1TOC	D	68	0	−1
1TOC	D	69	1	1
1TOC	D	70	1	1
1TOC	D	71	1	1
1TOC	D	72	0	−1
1TOC	D	73	1	1

1TOC	D	74	1	−1
1TOC	D	75	0	−1
1TOC	D	76	0	−1
1TOC	D	77	1	1
1TOC	D	78	1	1
1TOC	D	79	0	−1
1TOC	D	80	1	1
1TOC	D	81	0	−1
1TOC	D	82	1	−1
1TOC	D	83	1	−1
1TOC	D	84	0	−1
1TOC	D	85	1	−1
1TOC	D	86	1	−1
1TOC	D	87	0	−1
1TOC	D	88	1	−1
1TOC	D	89	1	−1
1TOC	D	90	0	−1
1TOC	D	91	1	−1
1TOC	D	92	1	−1
1TOC	D	93	1	−1
1TOC	D	94	1	−1
1TOC	D	95	0	−1
1TOC	D	96	0	−1
1TOC	D	97	0	−1
1TOC	D	98	0	−1
1TOC	D	99	0	−1
1TOC	D	100	0	−1
1TOC	D	101	0	−1
1TOC	D	102	0	−1
1TOC	D	103	0	−1
1TOC	D	104	0	−1
1TOC	D	105	0	−1
1TOC	D	106	1	−1
1TOC	D	107	1	1
1TOC	D	108	1	−1
1TOC	D	109	0	−1
1TOC	D	110	1	−1
1TOC	D	111	1	−1
1TOC	D	112	1	−1
1TOC	D	113	1	−1
1TOC	D	114	1	−1
1TOC	D	115	0	−1
1TOC	D	116	0	−1
1TOC	D	117	0	−1
1TOC	D	118	0	−1
1TOC	D	119	0	−1
1TOC	D	120	0	−1
1TOC	D	121	0	−1
1TOC	D	122	1	−1
1TOC	D	123	1	−1
1TOC	D	124	1	−1
1TOC	D	125	0	−1

1TOC	D	126	0	−1
1TOC	D	127	1	−1
1TOC	D	128	1	−1
1TOC	D	129	0	−1
1TOC	D	130	1	−1
1TOC	D	131	1	−1
1TOC	D	132	1	−1
1TOC	D	133	1	−1
1TOC	D	134	0	−1
1TOC	D	135	0	−1
1TOC	D	136	0	−1
1TOC	D	137	0	−1
1TOC	D	138	0	−1
1TOC	D	139	0	−1
1TOC	D	140	0	−1
1TOC	D	141	0	−1
1TOC	D	142	0	−1
1TOC	D	143	0	−1
1TOC	D	144	0	−1
1TOC	D	145	1	1
1TOC	D	146	1	1
1TOC	D	147	1	1
1TOC	D	148	1	1
1TOC	D	149	1	−1
1TOC	D	150	1	1
1TOC	D	151	1	−1
1TOC	D	152	1	−1
1TOC	D	153	1	−1
1TOC	D	154	1	1
1TOC	D	155	0	−1
1TOC	D	156	1	1
1TOC	D	157	0	−1
1TOC	D	158	1	−1
1TOC	D	159	0	−1
1TOC	D	160	0	−1
1TOC	D	161	0	−1
1TOC	D	162	0	−1
1TOC	D	163	0	−1
1TOC	D	164	0	−1
1TOC	D	165	0	−1
1TOC	D	166	0	−1
1TOC	D	167	0	−1
1TOC	D	168	0	−1
1TOC	D	169	1	−1
1TOC	D	170	1	−1
1TOC	D	171	1	−1
1TOC	D	172	0	−1
1TOC	D	173	0	−1
1TOC	D	174	1	−1
1TOC	D	175	0	−1
1TOC	D	176	0	−1
1TOC	D	177	0	−1

1TOC	D	178	1	1
1TOC	D	179	1	1
1TOC	D	180	1	−1
1TOC	D	181	0	−1
1TOC	D	182	0	−1
1TOC	D	183	1	−1
1TOC	D	184	0	−1
1TOC	D	185	0	−1
1TOC	D	186	0	−1
1TOC	D	187	0	−1
1TOC	D	188	0	−1
1TOC	D	189	0	−1
1TOC	D	190	0	−1
1TOC	D	191	0	−1
1TOC	D	192	0	−1
1TOC	D	193	1	−1
1TOC	D	194	1	−1
1TOC	D	195	1	−1
1TOC	D	196	1	−1
1TOC	D	197	1	1
1TOC	D	198	0	−1
1TOC	D	199	0	−1
1TOC	D	200	0	−1
1TOC	D	201	0	−1
1TOC	D	202	1	1
1TOC	D	203	0	−1
1TOC	D	204	0	−1
1TOC	D	205	0	−1
1TOC	D	206	0	−1
1TOC	D	207	0	−1
1TOC	D	208	0	−1
1TOC	D	209	0	−1
1TOC	D	210	0	−1
1TOC	D	211	0	−1
1TOC	D	212	0	−1
1TOC	D	213	0	−1
1TOC	D	214	1	−1
1TOC	D	215	1	−1
1TOC	D	216	1	−1
1TOC	D	217	1	−1
1TOC	D	218	0	−1
1TOC	D	219	0	−1
1TOC	D	220	0	−1
1TOC	D	221	0	−1
1TOC	D	222	0	−1
1TOC	D	223	0	−1
1TOC	D	224	0	−1
1TOC	D	225	0	−1
1TOC	D	226	0	−1
1TOC	D	227	0	−1
1TOC	D	228	1	1
1TOC	D	229	0	−1

1TOC	D	230	1	1
1TOC	D	231	0	−1
1TOC	D	232	0	−1
1TOC	D	233	1	1
1TOC	D	234	1	−1
1TOC	D	235	1	−1
1TOC	D	236	1	1
1TOC	D	237	0	−1
1TOC	D	238	0	−1
1TOC	D	239	0	−1
1TOC	D	240	0	−1
1TOC	D	241	0	−1
1TOC	D	242	0	−1
1TOC	D	243	0	−1
1TOC	D	244	0	−1
1TOC	D	245	1	−1
1TOC	D	246	0	−1
1TOC	D	247	1	−1
1TOC	D	248	1	−1
1TOC	D	249	0	−1
1TOC	D	250	0	−1
1TOC	D	251	1	−1
1TOC	D	252	1	−1
1TOC	D	253	0	−1
1TOC	D	254	1	−1
1TOC	D	255	1	−1
1TOC	D	256	1	−1
1TOC	D	257	1	−1
1TOC	D	258	1	−1
1TOC	D	259	1	−1
1TOC	D	20	1	−1
1TOC	D	21	1	−1
1TOC	D	22	0	−1
1TOC	D	23	0	−1
1TOC	D	24	0	−1
1TOC	D	25	0	−1
1TOC	D	26	0	−1
1TOC	D	27	0	−1
1TOC	D	28	0	−1
1TOC	D	29	0	−1
1TOC	D	30	1	−1
1TOC	D	31	1	−1
1TOC	D	32	1	−1
1TOC	D	33	1	−1
1TOC	D	34	1	−1
1TOC	D	35	0	−1
1TOC	D	36	1	−1
1TOC	D	37	0	−1
1TOC	D	38	0	−1
1TOC	D	39	1	−1
1TOC	D	40	1	−1
1TOC	D	41	0	−1

1TOC	D	42	1	−1
1TOC	D	43	1	−1
1TOC	D	44	0	−1
1TOC	D	45	0	−1
1TOC	D	46	1	−1
1TOC	D	47	1	1
1TOC	D	1	0	−1
1TOC	D	2	0	−1
1TOC	D	3	1	1
1TOC	D	4	1	−1
1TOC	D	5	1	−1
1TOC	D	6	1	−1
1TOC	D	7	0	−1
1TOC	D	8	1	−1
1TOC	D	9	1	−1
1TOC	D	10	0	−1
1TOC	D	11	0	−1
1TOC	D	12	0	−1
1TOC	D	13	0	−1
1TOC	D	14	0	−1
1TOC	D	15	0	−1
1TOC	D	16	0	−1
1TOC	D	17	0	−1
1TOC	D	18	0	−1
1TOC	D	19	0	−1
1TOC	D	20	1	−1
1TOC	D	21	1	1
1TOC	D	22	1	1
1TOC	D	23	1	−1
1TOC	D	24	1	1
1TOC	D	25	1	1
1TOC	D	26	1	1
1TOC	D	27	0	−1
1TOC	D	28	0	−1
1TOC	D	29	0	−1
1TOC	D	30	0	−1
1TOC	D	31	0	−1
1TOC	D	32	0	−1
1TOC	D	33	0	−1
1TOC	D	34	0	−1
1TOC	D	35	1	−1
1TOC	D	36	1	−1
1TOC	D	37	0	−1
1TOC	D	38	0	−1
1TOC	D	39	0	−1
1TOC	D	40	0	−1
1TOC	D	41	0	−1
1TOC	D	42	0	−1
1TOC	D	43	0	−1
1TOC	D	44	0	−1
1TOC	D	45	0	−1
1TOC	D	46	0	−1

1TOC	D	47	0	−1
1TOC	D	48	1	−1
1TOC	D	49	1	1
1TOC	D	50	1	1
1TOC	D	51	1	1
1TOC	D	52	0	−1
1TOC	D	53	1	−1
1TOC	D	54	0	−1
1TOC	D	55	1	−1
1TOC	D	56	1	−1
1TOC	D	57	1	−1
1TOC	D	58	1	−1
1TOC	D	59	0	−1
1TOC	D	60	0	−1
1TOC	D	61	0	−1
1TOC	D	62	0	−1
1TOC	D	63	0	−1
1TOC	D	64	0	−1
1TOC	D	65	0	−1
1TOC	D	66	0	−1
1TOC	D	67	0	−1
1TOC	D	68	0	−1
1TOC	D	69	1	1
1TOC	D	70	1	1
1TOC	D	71	1	1
1TOC	D	72	0	−1
1TOC	D	73	1	1
1TOC	D	74	1	−1
1TOC	D	75	0	−1
1TOC	D	76	0	−1
1TOC	D	77	1	1
1TOC	D	78	1	1
1TOC	D	79	0	−1
1TOC	D	80	1	1
1TOC	D	81	0	−1
1TOC	D	82	1	−1
1TOC	D	83	1	−1
1TOC	D	84	0	−1
1TOC	D	85	0	−1
1TOC	D	86	1	−1
1TOC	D	87	0	−1
1TOC	D	88	1	−1
1TOC	D	89	1	−1
1TOC	D	90	0	−1
1TOC	D	91	1	−1
1TOC	D	92	1	−1
1TOC	D	93	1	−1
1TOC	D	94	1	−1
1TOC	D	95	0	−1
1TOC	D	96	0	−1
1TOC	D	97	0	−1
1TOC	D	98	0	−1

1TOC	D	99	0	−1
1TOC	D	100	0	−1
1TOC	D	101	0	−1
1TOC	D	102	0	−1
1TOC	D	103	0	−1
1TOC	D	104	0	−1
1TOC	D	105	0	−1
1TOC	D	106	1	−1
1TOC	D	107	1	1
1TOC	D	108	1	−1
1TOC	D	109	0	−1
1TOC	D	110	1	−1
1TOC	D	111	1	−1
1TOC	D	112	1	−1
1TOC	D	113	1	−1
1TOC	D	114	1	−1
1TOC	D	115	0	−1
1TOC	D	116	0	−1
1TOC	D	117	0	−1
1TOC	D	118	0	−1
1TOC	D	119	0	−1
1TOC	D	120	0	−1
1TOC	D	121	0	−1
1TOC	D	122	1	−1
1TOC	D	123	1	1
1TOC	D	124	1	1
1TOC	D	125	0	−1
1TOC	D	126	0	−1
1TOC	D	127	1	1
1TOC	D	128	0	−1
1TOC	D	129	0	−1
1TOC	D	130	1	−1
1TOC	D	131	1	1
1TOC	D	132	1	−1
1TOC	D	133	1	−1
1TOC	D	134	0	−1
1TOC	D	135	0	−1
1TOC	D	136	0	−1
1TOC	D	137	0	−1
1TOC	D	138	0	−1
1TOC	D	139	0	−1
1TOC	D	140	0	−1
1TOC	D	141	0	−1
1TOC	D	142	0	−1
1TOC	D	143	0	−1
1TOC	D	144	1	−1
1TOC	D	145	1	1
1TOC	D	146	1	1
1TOC	D	147	1	1
1TOC	D	148	1	1
1TOC	D	149	1	−1
1TOC	D	150	1	−1

1TOC	D	151	1	−1
1TOC	D	152	1	−1
1TOC	D	153	1	−1
1TOC	D	154	1	−1
1TOC	D	155	0	−1
1TOC	D	156	1	1
1TOC	D	157	0	−1
1TOC	D	158	1	−1
1TOC	D	159	0	−1
1TOC	D	160	0	−1
1TOC	D	161	0	−1
1TOC	D	162	0	−1
1TOC	D	163	0	−1
1TOC	D	164	0	−1
1TOC	D	165	0	−1
1TOC	D	166	0	−1
1TOC	D	167	0	−1
1TOC	D	168	0	−1
1TOC	D	169	1	−1
1TOC	D	170	1	−1
1TOC	D	171	1	−1
1TOC	D	172	0	−1
1TOC	D	173	0	−1
1TOC	D	174	1	−1
1TOC	D	175	0	−1
1TOC	D	176	0	−1
1TOC	D	177	0	−1
1TOC	D	178	1	1
1TOC	D	179	1	1
1TOC	D	180	1	−1
1TOC	D	181	0	−1
1TOC	D	182	0	−1
1TOC	D	183	1	−1
1TOC	D	184	0	−1
1TOC	D	185	0	−1
1TOC	D	186	0	−1
1TOC	D	187	0	−1
1TOC	D	188	0	−1
1TOC	D	189	0	−1
1TOC	D	190	0	−1
1TOC	D	191	0	−1
1TOC	D	192	0	−1
1TOC	D	193	1	−1
1TOC	D	194	1	−1
1TOC	D	195	1	−1
1TOC	D	196	1	−1
1TOC	D	197	1	1
1TOC	D	198	0	−1
1TOC	D	199	0	−1
1TOC	D	200	0	−1
1TOC	D	201	0	−1
1TOC	D	202	1	1

1TOC	D	203	0	−1
1TOC	D	204	0	−1
1TOC	D	205	0	−1
1TOC	D	206	0	−1
1TOC	D	207	0	−1
1TOC	D	208	0	−1
1TOC	D	209	0	−1
1TOC	D	210	0	−1
1TOC	D	211	0	−1
1TOC	D	212	0	−1
1TOC	D	213	0	−1
1TOC	D	214	1	−1
1TOC	D	215	1	1
1TOC	D	216	1	−1
1TOC	D	217	1	−1
1TOC	D	218	0	−1
1TOC	D	219	0	−1
1TOC	D	220	0	−1
1TOC	D	221	0	−1
1TOC	D	222	0	−1
1TOC	D	223	0	−1
1TOC	D	224	0	−1
1TOC	D	225	0	−1
1TOC	D	226	0	−1
1TOC	D	227	0	−1
1TOC	D	228	1	1
1TOC	D	229	0	−1
1TOC	D	230	1	1
1TOC	D	231	0	−1
1TOC	D	232	0	−1
1TOC	D	233	1	1
1TOC	D	234	1	1
1TOC	D	235	1	−1
1TOC	D	236	1	1
1TOC	D	237	0	−1
1TOC	D	238	0	−1
1TOC	D	239	0	−1
1TOC	D	240	0	−1
1TOC	D	241	0	−1
1TOC	D	242	0	−1
1TOC	D	243	0	−1
1TOC	D	244	1	−1
1TOC	D	245	1	−1
1TOC	D	246	0	−1
1TOC	D	247	1	−1
1TOC	D	248	1	−1
1TOC	D	249	0	−1
1TOC	D	250	0	−1
1TOC	D	251	1	−1
1TOC	D	252	1	−1
1TOC	D	253	0	−1
1TOC	D	254	1	−1

1TOC	D	255	1	−1
1TOC	D	256	1	−1
1TOC	D	257	1	−1
1TOC	D	258	1	−1
1TOC	D	259	1	−1
1TOC	D	20	1	−1
1TOC	D	21	1	−1
1TOC	D	22	0	−1
1TOC	D	23	0	−1
1TOC	D	24	0	−1
1TOC	D	25	0	−1
1TOC	D	26	0	−1
1TOC	D	27	0	−1
1TOC	D	28	0	−1
1TOC	D	29	0	−1
1TOC	D	30	1	−1
1TOC	D	31	1	−1
1TOC	D	32	1	−1
1TOC	D	33	1	−1
1TOC	D	34	1	−1
1TOC	D	35	0	−1
1TOC	D	36	1	−1
1TOC	D	37	0	−1
1TOC	D	38	0	−1
1TOC	D	39	1	−1
1TOC	D	40	1	−1
1TOC	D	41	0	−1
1TOC	D	42	1	−1
1TOC	D	43	1	−1
1TOC	D	44	0	−1
1TOC	D	45	0	−1
1TOC	D	46	1	−1
1TOC	D	47	1	−1
1TOC	D	1	0	−1
1TOC	D	2	0	−1
1TOC	D	3	1	1
1TOC	D	4	1	−1
1TOC	D	5	1	−1
1TOC	D	6	1	−1
1TOC	D	7	0	−1
1TOC	D	8	1	−1
1TOC	D	9	1	−1
1TOC	D	10	0	−1
1TOC	D	11	0	−1
1TOC	D	12	0	−1
1TOC	D	13	0	−1
1TOC	D	14	0	−1
1TOC	D	15	0	−1
1TOC	D	16	0	−1
1TOC	D	17	0	−1
1TOC	D	18	0	−1
1TOC	D	19	0	−1

1TOC	D	20	1	−1
1TOC	D	21	1	1
1TOC	D	22	1	1
1TOC	D	23	1	−1
1TOC	D	24	1	1
1TOC	D	25	1	1
1TOC	D	26	1	1
1TOC	D	27	0	−1
1TOC	D	28	0	−1
1TOC	D	29	0	−1
1TOC	D	30	0	−1
1TOC	D	31	0	−1
1TOC	D	32	0	−1
1TOC	D	33	0	−1
1TOC	D	34	0	−1
1TOC	D	35	1	−1
1TOC	D	36	0	−1
1TOC	D	37	0	−1
1TOC	D	38	0	−1
1TOC	D	39	0	−1
1TOC	D	40	0	−1
1TOC	D	41	0	−1
1TOC	D	42	0	−1
1TOC	D	43	0	−1
1TOC	D	44	0	−1
1TOC	D	45	0	−1
1TOC	D	46	0	−1
1TOC	D	47	0	−1
1TOC	D	48	1	−1
1TOC	D	49	1	1
1TOC	D	50	1	1
1TOC	D	51	1	1
1TOC	D	52	0	−1
1TOC	D	53	1	−1
1TOC	D	54	0	−1
1TOC	D	55	1	−1
1TOC	D	56	1	−1
1TOC	D	57	1	−1
1TOC	D	58	1	−1
1TOC	D	59	0	−1
1TOC	D	60	0	−1
1TOC	D	61	0	−1
1TOC	D	62	0	−1
1TOC	D	63	0	−1
1TOC	D	64	0	−1
1TOC	D	65	0	−1
1TOC	D	66	0	−1
1TOC	D	67	0	−1
1TOC	D	68	0	−1
1TOC	D	69	1	1
1TOC	D	70	1	1
1TOC	D	71	1	1

1TOC	D	72	0	−1
1TOC	D	73	1	1
1TOC	D	74	1	1
1TOC	D	75	1	−1
1TOC	D	76	0	−1
1TOC	D	77	1	1
1TOC	D	78	1	1
1TOC	D	79	0	−1
1TOC	D	80	1	1
1TOC	D	81	0	−1
1TOC	D	82	1	−1
1TOC	D	83	1	−1
1TOC	D	84	0	−1
1TOC	D	85	1	−1
1TOC	D	86	1	−1
1TOC	D	87	0	−1
1TOC	D	88	1	−1
1TOC	D	89	1	−1
1TOC	D	90	0	−1
1TOC	D	91	1	−1
1TOC	D	92	1	−1
1TOC	D	93	1	1
1TOC	D	94	1	−1
1TOC	D	95	0	−1
1TOC	D	96	0	−1
1TOC	D	97	0	−1
1TOC	D	98	0	−1
1TOC	D	99	0	−1
1TOC	D	100	0	−1
1TOC	D	101	0	−1
1TOC	D	102	0	−1
1TOC	D	103	0	−1
1TOC	D	104	0	−1
1TOC	D	105	0	−1
1TOC	D	106	1	−1
1TOC	D	107	1	−1
1TOC	D	108	1	−1
1TOC	D	109	0	−1
1TOC	D	110	1	−1
1TOC	D	111	1	−1
1TOC	D	112	1	−1
1TOC	D	113	1	−1
1TOC	D	114	1	−1
1TOC	D	115	0	−1
1TOC	D	116	0	−1
1TOC	D	117	0	−1
1TOC	D	118	0	−1
1TOC	D	119	0	−1
1TOC	D	120	0	−1
1TOC	D	121	0	−1
1TOC	D	122	1	−1
1TOC	D	123	1	−1

1TOC	D	124	1	−1
1TOC	D	125	0	−1
1TOC	D	126	0	−1
1TOC	D	127	1	−1
1TOC	D	128	1	−1
1TOC	D	129	0	−1
1TOC	D	130	1	−1
1TOC	D	131	1	−1
1TOC	D	132	1	−1
1TOC	D	133	1	−1
1TOC	D	134	0	−1
1TOC	D	135	0	−1
1TOC	D	136	0	−1
1TOC	D	137	0	−1
1TOC	D	138	0	−1
1TOC	D	139	0	−1
1TOC	D	140	0	−1
1TOC	D	141	0	−1
1TOC	D	142	0	−1
1TOC	D	143	0	−1
1TOC	D	144	1	−1
1TOC	D	145	1	1
1TOC	D	146	1	1
1TOC	D	147	1	1
1TOC	D	148	1	1
1TOC	D	149	1	−1
1TOC	D	150	1	−1
1TOC	D	151	1	−1
1TOC	D	152	1	−1
1TOC	D	153	1	−1
1TOC	D	154	1	−1
1TOC	D	155	0	−1
1TOC	D	156	1	1
1TOC	D	157	0	−1
1TOC	D	158	1	−1
1TOC	D	159	0	−1
1TOC	D	160	0	−1
1TOC	D	161	0	−1
1TOC	D	162	0	−1
1TOC	D	163	0	−1
1TOC	D	164	0	−1
1TOC	D	165	0	−1
1TOC	D	166	0	−1
1TOC	D	167	0	−1
1TOC	D	168	0	−1
1TOC	D	169	1	−1
1TOC	D	170	1	−1
1TOC	D	171	1	−1
1TOC	D	172	0	−1
1TOC	D	173	0	−1
1TOC	D	174	1	−1
1TOC	D	175	0	−1

1TOC	D	176	0	−1
1TOC	D	177	0	−1
1TOC	D	178	1	1
1TOC	D	179	1	1
1TOC	D	180	1	−1
1TOC	D	181	0	−1
1TOC	D	182	0	−1
1TOC	D	183	1	−1
1TOC	D	184	0	−1
1TOC	D	185	0	−1
1TOC	D	186	0	−1
1TOC	D	187	0	−1
1TOC	D	188	0	−1
1TOC	D	189	0	−1
1TOC	D	190	0	−1
1TOC	D	191	0	−1
1TOC	D	192	0	−1
1TOC	D	193	1	−1
1TOC	D	194	1	−1
1TOC	D	195	1	−1
1TOC	D	196	1	−1
1TOC	D	197	1	1
1TOC	D	198	0	−1
1TOC	D	199	0	−1
1TOC	D	200	0	−1
1TOC	D	201	0	−1
1TOC	D	202	1	1
1TOC	D	203	0	−1
1TOC	D	204	0	−1
1TOC	D	205	0	−1
1TOC	D	206	0	−1
1TOC	D	207	0	−1
1TOC	D	208	0	−1
1TOC	D	209	0	−1
1TOC	D	210	0	−1
1TOC	D	211	0	−1
1TOC	D	212	0	−1
1TOC	D	213	0	−1
1TOC	D	214	1	−1
1TOC	D	215	1	−1
1TOC	D	216	1	−1
1TOC	D	217	1	−1
1TOC	D	218	0	−1
1TOC	D	219	0	−1
1TOC	D	220	0	−1
1TOC	D	221	0	−1
1TOC	D	222	0	−1
1TOC	D	223	0	−1
1TOC	D	224	0	−1
1TOC	D	225	0	−1
1TOC	D	226	0	−1
1TOC	D	227	0	−1

1TOC	D	228	1	1
1TOC	D	229	0	−1
1TOC	D	230	1	1
1TOC	D	231	0	−1
1TOC	D	232	0	−1
1TOC	D	233	1	1
1TOC	D	234	1	1
1TOC	D	235	1	−1
1TOC	D	236	1	1
1TOC	D	237	0	−1
1TOC	D	238	0	−1
1TOC	D	239	0	−1
1TOC	D	240	0	−1
1TOC	D	241	0	−1
1TOC	D	242	0	−1
1TOC	D	243	0	−1
1TOC	D	244	0	−1
1TOC	D	245	1	−1
1TOC	D	246	0	−1
1TOC	D	247	1	−1
1TOC	D	248	1	−1
1TOC	D	249	0	−1
1TOC	D	250	0	−1
1TOC	D	251	1	−1
1TOC	D	252	1	−1
1TOC	D	253	0	−1
1TOC	D	254	1	−1
1TOC	D	255	1	−1
1TOC	D	256	1	−1
1TOC	D	257	1	−1
1TOC	D	258	1	−1
1TOC	D	259	1	−1
1TOC	D	20	1	−1
1TOC	D	21	1	−1
1TOC	D	22	0	−1
1TOC	D	23	0	−1
1TOC	D	24	0	−1
1TOC	D	25	0	−1
1TOC	D	26	0	−1
1TOC	D	27	0	−1
1TOC	D	28	0	−1
1TOC	D	29	0	−1
1TOC	D	30	1	−1
1TOC	D	31	1	−1
1TOC	D	32	1	−1
1TOC	D	33	1	−1
1TOC	D	34	1	−1
1TOC	D	35	0	−1
1TOC	D	36	1	−1
1TOC	D	37	0	−1
1TOC	D	38	0	−1
1TOC	D	39	1	−1

1TOC	D	40	1	−1
1TOC	D	41	0	−1
1TOC	D	42	1	−1
1TOC	D	43	1	−1
1TOC	D	44	0	−1
1TOC	D	45	0	−1
1TOC	D	46	1	−1
1TOC	D	47	1	−1
1TOC	D	1	0	−1
1TOC	D	2	0	−1
1TOC	D	3	1	1
1TOC	D	4	1	−1
1TOC	D	5	1	−1
1TOC	D	6	1	−1
1TOC	D	7	0	−1
1TOC	D	8	1	−1
1TOC	D	9	1	−1
1TOC	D	10	0	−1
1TOC	D	11	0	−1
1TOC	D	12	0	−1
1TOC	D	13	0	−1
1TOC	D	14	0	−1
1TOC	D	15	0	−1
1TOC	D	16	0	−1
1TOC	D	17	0	−1
1TOC	D	18	0	−1
1TOC	D	19	0	−1
1TOC	D	20	1	−1
1TOC	D	21	1	1
1TOC	D	22	1	1
1TOC	D	23	1	−1
1TOC	D	24	1	1
1TOC	D	25	1	1
1TOC	D	26	1	1
1TOC	D	27	0	−1
1TOC	D	28	0	−1
1TOC	D	29	0	−1
1TOC	D	30	0	−1
1TOC	D	31	0	−1
1TOC	D	32	0	−1
1TOC	D	33	0	−1
1TOC	D	34	0	−1
1TOC	D	35	1	−1
1TOC	D	36	1	−1
1TOC	D	37	0	−1
1TOC	D	38	0	−1
1TOC	D	39	0	−1
1TOC	D	40	0	−1
1TOC	D	41	0	−1
1TOC	D	42	0	−1
1TOC	D	43	0	−1
1TOC	D	44	0	−1

1TOC	D	45	0	−1
1TOC	D	46	0	−1
1TOC	D	47	0	−1
1TOC	D	48	1	−1
1TOC	D	49	1	1
1TOC	D	50	1	1
1TOC	D	51	1	1
1TOC	D	52	0	−1
1TOC	D	53	1	−1
1TOC	D	54	0	−1
1TOC	D	55	1	−1
1TOC	D	56	1	−1
1TOC	D	57	1	−1
1TOC	D	58	1	−1
1TOC	D	59	0	−1
1TOC	D	60	0	−1
1TOC	D	61	0	−1
1TOC	D	62	0	−1
1TOC	D	63	0	−1
1TOC	D	64	0	−1
1TOC	D	65	0	−1
1TOC	D	66	0	−1
1TOC	D	67	0	−1
1TOC	D	68	0	−1
1TOC	D	69	1	1
1TOC	D	70	1	1
1TOC	D	71	1	1
1TOC	D	72	0	−1
1TOC	D	73	1	1
1TOC	D	74	1	−1
1TOC	D	75	0	−1
1TOC	D	76	0	−1
1TOC	D	77	1	1
1TOC	D	78	1	1
1TOC	D	79	0	−1
1TOC	D	80	1	1
1TOC	D	81	0	−1
1TOC	D	82	1	−1
1TOC	D	83	1	−1
1TOC	D	84	0	−1
1TOC	D	85	1	−1
1TOC	D	86	1	−1
1TOC	D	87	0	−1
1TOC	D	88	1	−1
1TOC	D	89	1	−1
1TOC	D	90	0	−1
1TOC	D	91	1	−1
1TOC	D	92	1	−1
1TOC	D	93	1	−1
1TOC	D	94	1	1
1TOC	D	95	0	−1
1TOC	D	96	0	−1

1TOC	D	97	0	−1
1TOC	D	98	0	−1
1TOC	D	99	0	−1
1TOC	D	100	0	−1
1TOC	D	101	0	−1
1TOC	D	102	0	−1
1TOC	D	103	0	−1
1TOC	D	104	0	−1
1TOC	D	105	0	−1
1TOC	D	106	1	−1
1TOC	D	107	1	−1
1TOC	D	108	1	−1
1TOC	D	109	0	−1
1TOC	D	110	1	−1
1TOC	D	111	1	−1
1TOC	D	112	1	−1
1TOC	D	113	1	−1
1TOC	D	114	1	−1
1TOC	D	115	0	−1
1TOC	D	116	0	−1
1TOC	D	117	0	−1
1TOC	D	118	0	−1
1TOC	D	119	0	−1
1TOC	D	120	0	−1
1TOC	D	121	0	−1
1TOC	D	122	1	−1
1TOC	D	123	1	−1
1TOC	D	124	1	−1
1TOC	D	125	0	−1
1TOC	D	126	0	−1
1TOC	D	127	1	−1
1TOC	D	128	1	−1
1TOC	D	129	0	−1
1TOC	D	130	1	−1
1TOC	D	131	1	−1
1TOC	D	132	1	−1
1TOC	D	133	1	−1
1TOC	D	134	0	−1
1TOC	D	135	0	−1
1TOC	D	136	0	−1
1TOC	D	137	0	−1
1TOC	D	138	0	−1
1TOC	D	139	0	−1
1TOC	D	140	0	−1
1TOC	D	141	0	−1
1TOC	D	142	0	−1
1TOC	D	143	0	−1
1TOC	D	144	0	−1
1TOC	D	145	1	1
1TOC	D	146	1	1
1TOC	D	147	1	1
1TOC	D	148	1	1

1TOC	D	149	1	−1
1TOC	D	150	1	1
1TOC	D	151	1	−1
1TOC	D	152	1	−1
1TOC	D	153	1	−1
1TOC	D	154	1	1
1TOC	D	155	0	−1
1TOC	D	156	1	1
1TOC	D	157	0	−1
1TOC	D	158	1	−1
1TOC	D	159	0	−1
1TOC	D	160	0	−1
1TOC	D	161	0	−1
1TOC	D	162	0	−1
1TOC	D	163	0	−1
1TOC	D	164	0	−1
1TOC	D	165	0	−1
1TOC	D	166	0	−1
1TOC	D	167	0	−1
1TOC	D	168	0	−1
1TOC	D	169	1	−1
1TOC	D	170	1	−1
1TOC	D	171	1	−1
1TOC	D	172	0	−1
1TOC	D	173	0	−1
1TOC	D	174	1	−1
1TOC	D	175	0	−1
1TOC	D	176	0	−1
1TOC	D	177	0	−1
1TOC	D	178	1	1
1TOC	D	179	1	1
1TOC	D	180	1	−1
1TOC	D	181	0	−1
1TOC	D	182	0	−1
1TOC	D	183	1	−1
1TOC	D	184	0	−1
1TOC	D	185	0	−1
1TOC	D	186	0	−1
1TOC	D	187	0	−1
1TOC	D	188	0	−1
1TOC	D	189	0	−1
1TOC	D	190	0	−1
1TOC	D	191	0	−1
1TOC	D	192	0	−1
1TOC	D	193	1	−1
1TOC	D	194	1	−1
1TOC	D	195	1	−1
1TOC	D	196	1	−1
1TOC	D	197	1	1
1TOC	D	198	0	−1
1TOC	D	199	0	−1
1TOC	D	200	0	−1

1TOC	D	201	0	−1
1TOC	D	202	1	1
1TOC	D	203	0	−1
1TOC	D	204	0	−1
1TOC	D	205	0	−1
1TOC	D	206	0	−1
1TOC	D	207	0	−1
1TOC	D	208	0	−1
1TOC	D	209	0	−1
1TOC	D	210	0	−1
1TOC	D	211	0	−1
1TOC	D	212	0	−1
1TOC	D	213	0	−1
1TOC	D	214	1	−1
1TOC	D	215	1	−1
1TOC	D	216	1	−1
1TOC	D	217	1	−1
1TOC	D	218	0	−1
1TOC	D	219	0	−1
1TOC	D	220	0	−1
1TOC	D	221	0	−1
1TOC	D	222	0	−1
1TOC	D	223	0	−1
1TOC	D	224	0	−1
1TOC	D	225	0	−1
1TOC	D	226	0	−1
1TOC	D	227	0	−1
1TOC	D	228	1	1
1TOC	D	229	0	−1
1TOC	D	230	1	1
1TOC	D	231	0	−1
1TOC	D	232	0	−1
1TOC	D	233	1	1
1TOC	D	234	1	1
1TOC	D	235	1	−1
1TOC	D	236	1	1
1TOC	D	237	0	−1
1TOC	D	238	0	−1
1TOC	D	239	0	−1
1TOC	D	240	0	−1
1TOC	D	241	0	−1
1TOC	D	242	0	−1
1TOC	D	243	0	−1
1TOC	D	244	1	−1
1TOC	D	245	1	−1
1TOC	D	246	0	−1
1TOC	D	247	1	−1
1TOC	D	248	1	−1
1TOC	D	249	0	−1
1TOC	D	250	0	−1
1TOC	D	251	1	−1
1TOC	D	252	1	−1

1TOC	D	253	0	−1
1TOC	D	254	1	−1
1TOC	D	255	1	−1
1TOC	D	256	1	−1
1TOC	D	257	1	−1
1TOC	D	258	1	−1
1TOC	D	259	1	−1
1TOC	U	1	1	1
1TOC	U	2	1	1
1TOC	U	3	1	1
1TOC	U	4	1	1
1TOC	U	5	1	1
1TOC	U	6	0	−1
1TOC	U	7	1	1
1TOC	U	8	0	−1
1TOC	U	9	1	1
1TOC	U	10	1	−1
1TOC	U	11	1	−1
1TOC	U	12	1	−1
1TOC	U	13	1	−1
1TOC	U	14	0	−1
1TOC	U	15	1	1
1TOC	U	16	1	1
1TOC	U	17	1	−1
1TOC	U	18	1	−1
1TOC	U	19	1	−1
1TOC	U	20	1	−1
1TOC	U	21	1	−1
1TOC	U	22	0	−1
1TOC	U	23	1	−1
1TOC	U	24	0	−1
1TOC	U	25	1	1
1TOC	U	26	1	1
1TOC	U	27	1	1
1TOC	U	28	1	1
1TOC	U	29	1	−1
1TOC	U	30	0	−1
1TOC	U	31	1	−1
1TOC	U	32	1	−1
1TOC	U	33	0	−1
1TOC	U	34	1	−1
1TOC	U	35	0	−1
1TOC	U	36	0	−1
1TOC	U	37	1	−1
1TOC	U	38	1	1
1TOC	U	39	0	−1
1TOC	U	40	0	−1
1TOC	U	41	0	−1
1TOC	U	42	1	−1
1TOC	U	43	1	−1
1TOC	U	44	1	−1
1TOC	U	45	1	1

1TOC	U	46	1	1
1TOC	U	47	0	−1
1TOC	U	48	1	−1
1TOC	U	49	1	1
1TOC	U	50	1	1
1TOC	U	51	0	−1
1TOC	U	52	1	1
1TOC	U	53	1	1
1TOC	U	54	1	1
1TOC	U	55	1	1
1TOC	U	56	1	−1
1TOC	U	57	1	1
1TOC	U	58	1	1
1TOC	U	59	1	1
1TOC	U	60	1	1
1TOC	U	61	1	1
1TOC	U	62	1	−1
1TOC	U	63	1	1
1TOC	U	64	1	−1
1TOC	U	65	0	−1
1TOC	U	66	0	−1
1TOC	U	67	1	−1
1TOC	U	68	1	−1
1TOC	U	69	1	−1
1TOC	U	70	1	−1
1TOC	U	71	0	−1
1TOC	U	72	1	−1
1TOC	U	73	1	−1
1TOC	U	74	0	−1
1TOC	U	75	1	−1
1TOC	U	76	1	−1
1TOC	U	77	0	−1
1TOC	U	78	1	−1
1TOC	U	79	1	−1
1TOC	U	80	0	−1
1TOC	U	81	0	−1
1TOC	U	82	0	−1
1TOC	U	83	0	−1
1TOC	U	84	0	−1
1TOC	U	85	1	−1
1TOC	U	86	1	−1
1TOC	U	87	1	−1
1TOC	U	88	1	−1
1TOC	U	89	1	−1
1TOC	U	90	0	−1
1TOC	U	91	1	−1
1TOC	U	92	1	−1
1TOC	U	93	1	−1
1TOC	U	94	1	−1
1TOC	U	95	1	−1
1TOC	U	96	0	−1
1TOC	U	97	1	−1

1TOC	U	98	1	−1
1TOC	U	99	1	−1
1TOC	U	100	1	−1
1TOC	U	101	1	1
1TOC	U	102	0	−1
1TOC	U	103	1	1
1TOC	U	104	0	−1
1TOC	U	105	1	1
1TOC	U	106	1	1
1TOC	U	107	1	−1
1TOC	U	108	1	1
1TOC	U	109	1	1
1TOC	U	110	0	−1
1TOC	U	111	0	−1
1TOC	U	112	1	1
1TOC	U	113	1	1
1TOC	U	114	0	−1
1TOC	U	115	1	1
1TOC	U	116	0	−1
1TOC	U	117	1	1
1TOC	U	118	1	1
1TOC	U	119	1	1
1TOC	U	120	1	1
1TOC	U	1	1	1
1TOC	U	2	1	1
1TOC	U	3	1	1
1TOC	U	4	1	1
1TOC	U	5	1	1
1TOC	U	6	0	−1
1TOC	U	7	1	1
1TOC	U	8	0	−1
1TOC	U	9	1	1
1TOC	U	10	1	−1
1TOC	U	11	1	−1
1TOC	U	12	1	−1
1TOC	U	13	1	−1
1TOC	U	14	0	−1
1TOC	U	15	1	1
1TOC	U	16	1	1
1TOC	U	17	1	1
1TOC	U	18	1	−1
1TOC	U	19	1	−1
1TOC	U	20	1	−1
1TOC	U	21	1	−1
1TOC	U	22	0	−1
1TOC	U	23	1	−1
1TOC	U	24	0	−1
1TOC	U	25	1	1
1TOC	U	26	1	1
1TOC	U	27	1	1
1TOC	U	28	1	1
1TOC	U	29	1	−1

1TOC	U	30	0	−1
1TOC	U	31	1	−1
1TOC	U	32	1	−1
1TOC	U	33	0	−1
1TOC	U	34	1	−1
1TOC	U	35	0	−1
1TOC	U	36	0	−1
1TOC	U	37	1	−1
1TOC	U	38	1	1
1TOC	U	39	0	−1
1TOC	U	40	0	−1
1TOC	U	41	0	−1
1TOC	U	42	1	−1
1TOC	U	43	1	−1
1TOC	U	44	1	−1
1TOC	U	45	1	−1
1TOC	U	46	1	1
1TOC	U	47	0	−1
1TOC	U	48	1	−1
1TOC	U	49	1	1
1TOC	U	50	1	1
1TOC	U	51	0	−1
1TOC	U	52	1	1
1TOC	U	53	1	1
1TOC	U	54	1	1
1TOC	U	55	1	1
1TOC	U	56	1	−1
1TOC	U	57	1	1
1TOC	U	58	1	1
1TOC	U	59	1	1
1TOC	U	60	1	1
1TOC	U	61	1	1
1TOC	U	62	1	−1
1TOC	U	63	1	1
1TOC	U	64	1	−1
1TOC	U	65	0	−1
1TOC	U	66	0	−1
1TOC	U	67	1	−1
1TOC	U	68	1	−1
1TOC	U	69	1	−1
1TOC	U	70	1	−1
1TOC	U	71	0	−1
1TOC	U	72	1	−1
1TOC	U	73	1	−1
1TOC	U	74	1	−1
1TOC	U	75	1	−1
1TOC	U	76	1	−1
1TOC	U	77	0	−1
1TOC	U	78	1	−1
1TOC	U	79	1	−1
1TOC	U	80	0	−1
1TOC	U	81	0	−1

1TOC	U	82	0	−1
1TOC	U	83	0	−1
1TOC	U	84	0	−1
1TOC	U	85	1	−1
1TOC	U	86	1	−1
1TOC	U	87	1	−1
1TOC	U	88	1	−1
1TOC	U	89	1	−1
1TOC	U	90	0	−1
1TOC	U	91	1	−1
1TOC	U	92	1	−1
1TOC	U	93	1	−1
1TOC	U	94	1	−1
1TOC	U	95	1	−1
1TOC	U	96	0	−1
1TOC	U	97	0	−1
1TOC	U	98	1	−1
1TOC	U	99	1	−1
1TOC	U	100	1	−1
1TOC	U	101	1	1
1TOC	U	102	0	−1
1TOC	U	103	1	1
1TOC	U	104	0	−1
1TOC	U	105	1	1
1TOC	U	106	1	1
1TOC	U	107	1	−1
1TOC	U	108	1	1
1TOC	U	109	1	1
1TOC	U	110	0	−1
1TOC	U	111	0	−1
1TOC	U	112	1	1
1TOC	U	113	0	−1
1TOC	U	114	0	−1
1TOC	U	115	1	1
1TOC	U	116	0	−1
1TOC	U	117	1	1
1TOC	U	118	1	1
1TOC	U	119	1	1
1TOC	U	120	1	1
1TOC	U	1	1	1
1TOC	U	2	1	1
1TOC	U	3	1	1
1TOC	U	4	1	1
1TOC	U	5	1	1
1TOC	U	6	0	−1
1TOC	U	7	1	1
1TOC	U	8	0	−1
1TOC	U	9	1	−1
1TOC	U	10	1	1
1TOC	U	11	1	−1
1TOC	U	12	1	1
1TOC	U	13	1	1

1TOC	U	14	0	−1
1TOC	U	15	1	1
1TOC	U	16	1	1
1TOC	U	17	1	1
1TOC	U	18	1	1
1TOC	U	19	1	1
1TOC	U	20	1	1
1TOC	U	21	1	1
1TOC	U	22	0	−1
1TOC	U	23	1	−1
1TOC	U	24	0	−1
1TOC	U	25	1	1
1TOC	U	26	1	−1
1TOC	U	27	1	1
1TOC	U	28	1	1
1TOC	U	29	1	−1
1TOC	U	30	0	−1
1TOC	U	31	1	−1
1TOC	U	32	1	−1
1TOC	U	33	0	−1
1TOC	U	34	1	1
1TOC	U	35	0	−1
1TOC	U	36	0	−1
1TOC	U	37	1	−1
1TOC	U	38	1	1
1TOC	U	39	0	−1
1TOC	U	40	0	−1
1TOC	U	41	0	−1
1TOC	U	42	1	−1
1TOC	U	43	1	−1
1TOC	U	44	1	−1
1TOC	U	45	1	−1
1TOC	U	46	1	1
1TOC	U	47	0	−1
1TOC	U	48	1	−1
1TOC	U	49	1	1
1TOC	U	50	1	1
1TOC	U	51	0	−1
1TOC	U	52	1	1
1TOC	U	53	1	1
1TOC	U	54	1	1
1TOC	U	55	1	1
1TOC	U	56	1	−1
1TOC	U	57	1	1
1TOC	U	58	1	1
1TOC	U	59	1	1
1TOC	U	60	1	1
1TOC	U	61	1	1
1TOC	U	62	1	−1
1TOC	U	63	1	1
1TOC	U	64	1	−1
1TOC	U	65	0	−1

1TOC	U	66	0	−1
1TOC	U	67	1	−1
1TOC	U	68	1	−1
1TOC	U	69	1	−1
1TOC	U	70	1	−1
1TOC	U	71	0	−1
1TOC	U	72	1	−1
1TOC	U	73	1	−1
1TOC	U	74	1	−1
1TOC	U	75	1	−1
1TOC	U	76	1	−1
1TOC	U	77	0	−1
1TOC	U	78	1	−1
1TOC	U	79	1	−1
1TOC	U	80	0	−1
1TOC	U	81	0	−1
1TOC	U	82	0	−1
1TOC	U	83	0	−1
1TOC	U	84	0	−1
1TOC	U	85	1	−1
1TOC	U	86	1	−1
1TOC	U	87	1	−1
1TOC	U	88	1	−1
1TOC	U	89	1	−1
1TOC	U	90	0	−1
1TOC	U	91	1	−1
1TOC	U	92	1	−1
1TOC	U	93	1	−1
1TOC	U	94	1	−1
1TOC	U	95	1	−1
1TOC	U	96	0	−1
1TOC	U	97	0	−1
1TOC	U	98	1	−1
1TOC	U	99	1	−1
1TOC	U	100	1	−1
1TOC	U	101	1	1
1TOC	U	102	0	−1
1TOC	U	103	1	1
1TOC	U	104	0	−1
1TOC	U	105	1	1
1TOC	U	106	1	1
1TOC	U	107	1	−1
1TOC	U	108	1	1
1TOC	U	109	1	1
1TOC	U	110	0	−1
1TOC	U	111	0	−1
1TOC	U	112	1	1
1TOC	U	113	0	−1
1TOC	U	114	0	−1
1TOC	U	115	1	1
1TOC	U	116	0	−1
1TOC	U	117	1	1

1TOC	U	118	1	1
1TOC	U	119	1	1
1TOC	U	120	1	1
1TOC	U	1	1	1
1TOC	U	2	1	1
1TOC	U	3	1	1
1TOC	U	4	1	1
1TOC	U	5	1	1
1TOC	U	6	0	−1
1TOC	U	7	1	1
1TOC	U	8	0	−1
1TOC	U	9	1	1
1TOC	U	10	1	−1
1TOC	U	11	1	−1
1TOC	U	12	1	−1
1TOC	U	13	1	−1
1TOC	U	14	0	−1
1TOC	U	15	1	1
1TOC	U	16	1	1
1TOC	U	17	1	−1
1TOC	U	18	1	−1
1TOC	U	19	1	−1
1TOC	U	20	1	−1
1TOC	U	21	1	−1
1TOC	U	22	0	−1
1TOC	U	23	0	−1
1TOC	U	24	0	−1
1TOC	U	25	1	1
1TOC	U	26	1	1
1TOC	U	27	1	1
1TOC	U	28	1	1
1TOC	U	29	1	−1
1TOC	U	30	0	−1
1TOC	U	31	1	−1
1TOC	U	32	1	−1
1TOC	U	33	0	−1
1TOC	U	34	1	−1
1TOC	U	35	0	−1
1TOC	U	36	0	−1
1TOC	U	37	1	−1
1TOC	U	38	1	1
1TOC	U	39	0	−1
1TOC	U	40	0	−1
1TOC	U	41	0	−1
1TOC	U	42	1	−1
1TOC	U	43	1	−1
1TOC	U	44	1	−1
1TOC	U	45	1	1
1TOC	U	46	1	1
1TOC	U	47	0	−1
1TOC	U	48	1	−1
1TOC	U	49	1	1

1TOC	U	50	1	1
1TOC	U	51	0	−1
1TOC	U	52	1	1
1TOC	U	53	1	1
1TOC	U	54	1	1
1TOC	U	55	1	1
1TOC	U	56	1	−1
1TOC	U	57	1	1
1TOC	U	58	1	1
1TOC	U	59	1	1
1TOC	U	60	1	1
1TOC	U	61	1	1
1TOC	U	62	1	−1
1TOC	U	63	1	1
1TOC	U	64	1	−1
1TOC	U	65	0	−1
1TOC	U	66	0	−1
1TOC	U	67	1	−1
1TOC	U	68	1	−1
1TOC	U	69	1	−1
1TOC	U	70	1	−1
1TOC	U	71	0	−1
1TOC	U	72	1	−1
1TOC	U	73	1	−1
1TOC	U	74	1	−1
1TOC	U	75	1	−1
1TOC	U	76	1	−1
1TOC	U	77	1	−1
1TOC	U	78	1	−1
1TOC	U	79	1	−1
1TOC	U	80	0	−1
1TOC	U	81	0	−1
1TOC	U	82	0	−1
1TOC	U	83	0	−1
1TOC	U	84	0	−1
1TOC	U	85	1	−1
1TOC	U	86	1	−1
1TOC	U	87	1	−1
1TOC	U	88	1	−1
1TOC	U	89	1	−1
1TOC	U	90	0	−1
1TOC	U	91	1	−1
1TOC	U	92	1	−1
1TOC	U	93	1	−1
1TOC	U	94	1	−1
1TOC	U	95	1	−1
1TOC	U	96	0	−1
1TOC	U	97	0	−1
1TOC	U	98	1	−1
1TOC	U	99	1	−1
1TOC	U	100	1	−1
1TOC	U	101	1	1

1TOC	U	102	0	−1
1TOC	U	103	1	1
1TOC	U	104	0	−1
1TOC	U	105	1	1
1TOC	U	106	1	−1
1TOC	U	107	1	−1
1TOC	U	108	1	1
1TOC	U	109	1	1
1TOC	U	110	0	−1
1TOC	U	111	0	−1
1TOC	U	112	1	1
1TOC	U	113	0	−1
1TOC	U	114	0	−1
1TOC	U	115	1	1
1TOC	U	116	0	−1
1TOC	U	117	1	1
1TOC	U	118	1	1
1TOC	U	119	1	1
1TOC	U	120	1	1
1TX4	A	3	1	−1
1TX4	A	4	1	−1
1TX4	A	5	1	−1
1TX4	A	6	1	−1
1TX4	A	7	0	−1
1TX4	A	8	0	−1
1TX4	A	9	0	−1
1TX4	A	10	1	−1
1TX4	A	11	0	−1
1TX4	A	12	1	−1
1TX4	A	13	0	−1
1TX4	A	14	1	−1
1TX4	A	15	1	−1
1TX4	A	16	0	−1
1TX4	A	17	0	−1
1TX4	A	18	1	−1
1TX4	A	19	1	−1
1TX4	A	20	0	−1
1TX4	A	21	1	−1
1TX4	A	22	1	−1
1TX4	A	23	1	−1
1TX4	A	24	1	−1
1TX4	A	25	1	−1
1TX4	A	26	0	−1
1TX4	A	27	0	−1
1TX4	A	28	1	−1
1TX4	A	29	0	−1
1TX4	A	30	0	−1
1TX4	A	31	1	−1
1TX4	A	32	1	−1
1TX4	A	33	0	−1
1TX4	A	34	0	−1
1TX4	A	35	1	−1

1TX4	A	36	0	−1
1TX4	A	37	0	−1
1TX4	A	38	1	−1
1TX4	A	39	1	−1
1TX4	A	40	1	−1
1TX4	A	41	0	−1
1TX4	A	42	0	−1
1TX4	A	43	1	−1
1TX4	A	44	0	−1
1TX4	A	45	1	1
1TX4	A	46	0	−1
1TX4	A	47	0	−1
1TX4	A	48	0	−1
1TX4	A	49	1	1
1TX4	A	50	1	1
1TX4	A	51	1	1
1TX4	A	52	1	1
1TX4	A	53	1	1
1TX4	A	54	1	1
1TX4	A	55	1	1
1TX4	A	56	1	−1
1TX4	A	57	0	−1
1TX4	A	58	1	1
1TX4	A	59	1	−1
1TX4	A	60	0	−1
1TX4	A	61	0	−1
1TX4	A	62	1	−1
1TX4	A	63	0	−1
1TX4	A	64	0	−1
1TX4	A	65	0	−1
1TX4	A	66	1	−1
1TX4	A	67	1	−1
1TX4	A	68	1	−1
1TX4	A	69	1	−1
1TX4	A	70	0	−1
1TX4	A	71	1	−1
1TX4	A	72	0	−1
1TX4	A	73	1	−1
1TX4	A	74	1	−1
1TX4	A	75	0	−1
1TX4	A	76	1	1
1TX4	A	77	1	1
1TX4	A	78	1	−1
1TX4	A	79	0	−1
1TX4	A	80	0	−1
1TX4	A	81	0	−1
1TX4	A	82	0	−1
1TX4	A	83	0	−1
1TX4	A	84	0	−1
1TX4	A	85	0	−1
1TX4	A	86	0	−1
1TX4	A	87	0	−1

1TX4	A	88	0	−1
1TX4	A	89	0	−1
1TX4	A	90	1	1
1TX4	A	91	1	−1
1TX4	A	92	0	−1
1TX4	A	93	1	−1
1TX4	A	94	1	−1
1TX4	A	95	0	−1
1TX4	A	96	0	−1
1TX4	A	97	0	−1
1TX4	A	98	0	−1
1TX4	A	99	1	−1
1TX4	A	100	1	−1
1TX4	A	101	0	−1
1TX4	A	102	0	−1
1TX4	A	103	1	−1
1TX4	A	104	0	−1
1TX4	A	105	0	−1
1TX4	A	106	1	−1
1TX4	A	107	1	−1
1TX4	A	108	0	−1
1TX4	A	109	1	−1
1TX4	A	110	1	−1
1TX4	A	111	0	−1
1TX4	A	112	1	−1
1TX4	A	113	1	−1
1TX4	A	114	1	−1
1TX4	A	115	1	−1
1TX4	A	116	0	−1
1TX4	A	117	0	−1
1TX4	A	118	1	−1
1TX4	A	119	1	−1
1TX4	A	120	0	−1
1TX4	A	121	1	−1
1TX4	A	122	1	−1
1TX4	A	123	0	−1
1TX4	A	124	0	−1
1TX4	A	125	1	−1
1TX4	A	126	1	−1
1TX4	A	127	0	−1
1TX4	A	128	1	−1
1TX4	A	129	1	−1
1TX4	A	130	0	−1
1TX4	A	131	0	−1
1TX4	A	132	1	−1
1TX4	A	133	0	−1
1TX4	A	134	0	−1
1TX4	A	135	0	−1
1TX4	A	136	1	−1
1TX4	A	137	1	−1
1TX4	A	138	0	−1
1TX4	A	139	0	−1

1TX4	A	140	1	−1
1TX4	A	141	0	−1
1TX4	A	142	0	−1
1TX4	A	143	1	−1
1TX4	A	144	1	−1
1TX4	A	145	0	−1
1TX4	A	146	0	−1
1TX4	A	147	1	−1
1TX4	A	148	1	−1
1TX4	A	149	1	−1
1TX4	A	150	1	−1
1TX4	A	151	1	−1
1TX4	A	152	0	−1
1TX4	A	153	1	1
1TX4	A	154	0	−1
1TX4	A	155	1	−1
1TX4	A	156	0	−1
1TX4	A	157	1	1
1TX4	A	158	1	1
1TX4	A	159	0	−1
1TX4	A	160	0	−1
1TX4	A	161	1	1
1TX4	A	162	0	−1
1TX4	A	163	0	−1
1TX4	A	164	0	−1
1TX4	A	165	1	1
1TX4	A	166	0	−1
1TX4	A	167	0	−1
1TX4	A	168	0	−1
1TX4	A	169	0	−1
1TX4	A	170	0	−1
1TX4	A	171	1	−1
1TX4	A	172	1	−1
1TX4	A	173	1	1
1TX4	A	174	1	1
1TX4	A	175	1	−1
1TX4	A	176	0	−1
1TX4	A	177	1	1
1TX4	A	178	1	−1
1TX4	A	179	0	−1
1TX4	A	180	1	1
1TX4	A	181	1	−1
1TX4	A	182	0	−1
1TX4	A	183	0	−1
1TX4	A	184	0	−1
1TX4	A	185	1	−1
1TX4	A	186	0	−1
1TX4	A	187	0	−1
1TX4	A	188	1	−1
1TX4	A	189	0	−1
1TX4	A	190	0	−1
1TX4	A	191	0	−1

1TX4	A	192	1	−1
1TX4	A	193	1	−1
1TX4	A	194	0	−1
1TX4	A	195	1	−1
1TX4	A	196	1	−1
1TX4	A	197	0	−1
1TX4	A	198	1	−1
1TX4	B	1	1	−1
1TX4	B	2	1	−1
1TX4	B	3	1	−1
1TX4	B	4	0	−1
1TX4	B	5	0	−1
1TX4	B	6	0	−1
1TX4	B	7	0	−1
1TX4	B	8	0	−1
1TX4	B	9	0	−1
1TX4	B	10	0	−1
1TX4	B	11	0	−1
1TX4	B	12	1	1
1TX4	B	13	1	1
1TX4	B	14	0	−1
1TX4	B	15	0	−1
1TX4	B	16	0	−1
1TX4	B	17	1	1
1TX4	B	18	1	−1
1TX4	B	19	0	−1
1TX4	B	20	0	−1
1TX4	B	21	0	−1
1TX4	B	22	0	−1
1TX4	B	23	0	−1
1TX4	B	24	1	−1
1TX4	B	25	1	−1
1TX4	B	26	1	−1
1TX4	B	27	1	−1
1TX4	B	28	1	−1
1TX4	B	32	1	1
1TX4	B	33	1	1
1TX4	B	34	1	1
1TX4	B	35	0	−1
1TX4	B	36	1	1
1TX4	B	37	1	1
1TX4	B	38	1	−1
1TX4	B	39	1	−1
1TX4	B	40	0	−1
1TX4	B	41	1	−1
1TX4	B	42	0	−1
1TX4	B	43	1	−1
1TX4	B	44	0	−1
1TX4	B	45	1	−1
1TX4	B	46	0	−1
1TX4	B	47	1	−1
1TX4	B	48	1	−1

1TX4	B	49	1	−1
1TX4	B	50	1	−1
1TX4	B	51	0	−1
1TX4	B	52	1	−1
1TX4	B	53	0	−1
1TX4	B	54	1	−1
1TX4	B	55	0	−1
1TX4	B	56	1	−1
1TX4	B	57	0	−1
1TX4	B	58	0	−1
1TX4	B	59	0	−1
1TX4	B	60	0	−1
1TX4	B	61	1	1
1TX4	B	62	1	1
1TX4	B	63	1	1
1TX4	B	64	1	1
1TX4	B	65	1	1
1TX4	B	66	1	1
1TX4	B	67	1	1
1TX4	B	68	0	−1
1TX4	B	69	0	−1
1TX4	B	70	1	1
1TX4	B	71	0	−1
1TX4	B	72	0	−1
1TX4	B	73	1	−1
1TX4	B	74	1	−1
1TX4	B	75	0	−1
1TX4	B	76	1	−1
1TX4	B	77	0	−1
1TX4	B	78	0	−1
1TX4	B	79	0	−1
1TX4	B	80	0	−1
1TX4	B	81	0	−1
1TX4	B	82	0	−1
1TX4	B	83	0	−1
1TX4	B	84	0	−1
1TX4	B	85	1	1
1TX4	B	86	1	1
1TX4	B	87	0	−1
1TX4	B	88	1	1
1TX4	B	89	0	−1
1TX4	B	90	0	−1
1TX4	B	91	1	1
1TX4	B	92	1	1
1TX4	B	93	0	−1
1TX4	B	94	0	−1
1TX4	B	95	1	1
1TX4	B	96	0	−1
1TX4	B	97	0	−1
1TX4	B	98	0	−1
1TX4	B	99	1	−1
1TX4	B	100	0	−1

1TX4	B	101	0	−1
1TX4	B	102	1	−1
1TX4	B	103	1	−1
1TX4	B	104	1	−1
1TX4	B	105	0	−1
1TX4	B	106	1	−1
1TX4	B	107	1	−1
1TX4	B	108	0	−1
1TX4	B	109	0	−1
1TX4	B	110	0	−1
1TX4	B	111	0	−1
1TX4	B	112	0	−1
1TX4	B	113	0	−1
1TX4	B	114	0	−1
1TX4	B	115	0	−1
1TX4	B	116	1	−1
1TX4	B	117	1	−1
1TX4	B	118	1	−1
1TX4	B	119	0	−1
1TX4	B	120	0	−1
1TX4	B	121	1	−1
1TX4	B	122	1	−1
1TX4	B	123	1	−1
1TX4	B	124	1	−1
1TX4	B	125	0	−1
1TX4	B	126	1	−1
1TX4	B	127	1	−1
1TX4	B	128	1	1
1TX4	B	129	0	−1
1TX4	B	130	1	−1
1TX4	B	131	1	−1
1TX4	B	132	1	1
1TX4	B	133	1	1
1TX4	B	134	1	1
1TX4	B	135	1	−1
1TX4	B	136	0	−1
1TX4	B	137	0	−1
1TX4	B	138	1	−1
1TX4	B	139	1	−1
1TX4	B	140	1	−1
1TX4	B	141	1	−1
1TX4	B	142	0	−1
1TX4	B	143	1	−1
1TX4	B	144	1	−1
1TX4	B	145	0	−1
1TX4	B	146	0	−1
1TX4	B	147	1	−1
1TX4	B	148	1	−1
1TX4	B	149	0	−1
1TX4	B	150	1	−1
1TX4	B	151	0	−1
1TX4	B	152	1	−1

1TX4	B	153	0	−1
1TX4	B	154	0	−1
1TX4	B	155	0	−1
1TX4	B	156	0	−1
1TX4	B	157	0	−1
1TX4	B	158	0	−1
1TX4	B	159	0	−1
1TX4	B	160	1	−1
1TX4	B	161	1	−1
1TX4	B	162	1	−1
1TX4	B	163	1	−1
1TX4	B	164	0	−1
1TX4	B	165	0	−1
1TX4	B	166	1	−1
1TX4	B	167	1	−1
1TX4	B	168	0	−1
1TX4	B	169	0	−1
1TX4	B	170	1	−1
1TX4	B	171	1	−1
1TX4	B	172	0	−1
1TX4	B	173	0	−1
1TX4	B	174	1	−1
1TX4	B	175	0	−1
1TX4	B	176	0	−1
1TX4	B	177	1	−1
1UDI	E	18	1	−1
1UDI	E	19	0	−1
1UDI	E	20	1	−1
1UDI	E	21	1	−1
1UDI	E	22	0	−1
1UDI	E	23	1	−1
1UDI	E	24	1	−1
1UDI	E	25	1	−1
1UDI	E	26	0	−1
1UDI	E	27	1	−1
1UDI	E	28	0	−1
1UDI	E	29	1	−1
1UDI	E	30	1	−1
1UDI	E	31	1	−1
1UDI	E	32	0	−1
1UDI	E	33	1	−1
1UDI	E	34	1	−1
1UDI	E	35	0	−1
1UDI	E	36	0	−1
1UDI	E	37	1	−1
1UDI	E	38	1	−1
1UDI	E	39	0	−1
1UDI	E	40	0	−1
1UDI	E	41	1	−1
1UDI	E	42	1	−1
1UDI	E	43	1	−1
1UDI	E	44	1	−1

1UDI	E	45	0	−1
1UDI	E	46	1	−1
1UDI	E	47	1	−1
1UDI	E	48	0	−1
1UDI	E	49	0	−1
1UDI	E	50	1	−1
1UDI	E	51	1	−1
1UDI	E	52	0	−1
1UDI	E	53	1	−1
1UDI	E	54	1	−1
1UDI	E	55	1	−1
1UDI	E	56	1	−1
1UDI	E	57	1	−1
1UDI	E	58	1	−1
1UDI	E	59	0	−1
1UDI	E	60	1	−1
1UDI	E	61	0	−1
1UDI	E	62	0	−1
1UDI	E	63	0	−1
1UDI	E	64	1	−1
1UDI	E	65	1	−1
1UDI	E	66	1	−1
1UDI	E	67	0	−1
1UDI	E	68	0	−1
1UDI	E	69	0	−1
1UDI	E	70	0	−1
1UDI	E	71	0	−1
1UDI	E	72	0	−1
1UDI	E	73	1	−1
1UDI	E	74	0	−1
1UDI	E	75	0	−1
1UDI	E	76	1	−1
1UDI	E	77	0	−1
1UDI	E	78	1	−1
1UDI	E	79	1	−1
1UDI	E	80	0	−1
1UDI	E	81	0	−1
1UDI	E	82	0	−1
1UDI	E	83	0	−1
1UDI	E	84	0	−1
1UDI	E	85	0	−1
1UDI	E	86	0	−1
1UDI	E	87	1	1
1UDI	E	88	0	−1
1UDI	E	89	0	−1
1UDI	E	90	0	−1
1UDI	E	91	1	1
1UDI	E	92	1	1
1UDI	E	93	1	−1
1UDI	E	94	1	−1
1UDI	E	95	0	−1
1UDI	E	96	0	−1

1UDI	E	97	0	−1
1UDI	E	98	0	−1
1UDI	E	99	0	−1
1UDI	E	100	0	−1
1UDI	E	101	0	−1
1UDI	E	102	0	−1
1UDI	E	103	0	−1
1UDI	E	104	1	−1
1UDI	E	105	1	−1
1UDI	E	106	1	−1
1UDI	E	107	0	−1
1UDI	E	108	1	1
1UDI	E	109	0	−1
1UDI	E	110	0	−1
1UDI	E	111	1	1
1UDI	E	112	0	−1
1UDI	E	113	0	−1
1UDI	E	114	1	1
1UDI	E	115	0	−1
1UDI	E	116	0	−1
1UDI	E	117	0	−1
1UDI	E	118	1	−1
1UDI	E	119	0	−1
1UDI	E	120	0	−1
1UDI	E	121	1	−1
1UDI	E	122	1	−1
1UDI	E	123	0	−1
1UDI	E	124	1	−1
1UDI	E	125	1	−1
1UDI	E	126	1	−1
1UDI	E	127	0	−1
1UDI	E	128	1	−1
1UDI	E	129	0	−1
1UDI	E	130	1	−1
1UDI	E	131	1	−1
1UDI	E	132	1	−1
1UDI	E	133	0	−1
1UDI	E	134	0	−1
1UDI	E	135	0	−1
1UDI	E	136	1	−1
1UDI	E	137	1	−1
1UDI	E	138	0	−1
1UDI	E	139	0	−1
1UDI	E	140	1	−1
1UDI	E	141	1	−1
1UDI	E	142	0	−1
1UDI	E	143	0	−1
1UDI	E	144	0	−1
1UDI	E	145	0	−1
1UDI	E	146	0	−1
1UDI	E	147	0	−1
1UDI	E	148	0	−1

1UDI	E	149	0	−1
1UDI	E	150	0	−1
1UDI	E	151	0	−1
1UDI	E	152	0	−1
1UDI	E	153	1	−1
1UDI	E	154	1	−1
1UDI	E	155	1	−1
1UDI	E	156	1	1
1UDI	E	157	1	1
1UDI	E	158	1	1
1UDI	E	159	0	−1
1UDI	E	160	0	−1
1UDI	E	161	1	1
1UDI	E	162	1	1
1UDI	E	163	0	−1
1UDI	E	164	0	−1
1UDI	E	165	0	−1
1UDI	E	166	1	−1
1UDI	E	167	1	−1
1UDI	E	168	0	−1
1UDI	E	169	0	−1
1UDI	E	170	1	−1
1UDI	E	171	0	−1
1UDI	E	172	0	−1
1UDI	E	173	0	−1
1UDI	E	174	1	−1
1UDI	E	175	0	−1
1UDI	E	176	0	−1
1UDI	E	177	0	−1
1UDI	E	178	1	−1
1UDI	E	179	1	−1
1UDI	E	180	1	−1
1UDI	E	181	1	−1
1UDI	E	182	0	−1
1UDI	E	183	0	−1
1UDI	E	184	0	−1
1UDI	E	185	0	−1
1UDI	E	186	0	−1
1UDI	E	187	0	−1
1UDI	E	188	0	−1
1UDI	E	189	0	−1
1UDI	E	190	1	1
1UDI	E	191	1	1
1UDI	E	192	0	−1
1UDI	E	193	0	−1
1UDI	E	194	1	1
1UDI	E	195	1	−1
1UDI	E	196	0	−1
1UDI	E	197	1	−1
1UDI	E	198	0	−1
1UDI	E	199	1	−1
1UDI	E	200	1	−1

1UDI	E	201	1	−1
1UDI	E	202	1	−1
1UDI	E	203	0	−1
1UDI	E	204	0	−1
1UDI	E	205	1	−1
1UDI	E	206	0	−1
1UDI	E	207	1	−1
1UDI	E	208	0	−1
1UDI	E	209	1	1
1UDI	E	210	0	−1
1UDI	E	211	0	−1
1UDI	E	212	1	1
1UDI	E	213	1	1
1UDI	E	214	1	1
1UDI	E	215	1	1
1UDI	E	216	1	1
1UDI	E	217	1	−1
1UDI	E	218	1	−1
1UDI	E	219	0	−1
1UDI	E	220	0	−1
1UDI	E	221	1	−1
1UDI	E	222	0	−1
1UDI	E	223	1	−1
1UDI	E	224	0	−1
1UDI	E	225	0	−1
1UDI	E	226	0	−1
1UDI	E	227	1	−1
1UDI	E	228	0	−1
1UDI	E	229	0	−1
1UDI	E	230	1	−1
1UDI	E	231	0	−1
1UDI	E	232	0	−1
1UDI	E	233	1	−1
1UDI	E	234	1	−1
1UDI	E	235	1	−1
1UDI	E	236	1	−1
1UDI	E	237	1	−1
1UDI	E	238	1	−1
1UDI	E	239	1	−1
1UDI	E	240	0	−1
1UDI	E	241	1	−1
1UDI	E	242	0	−1
1UDI	E	243	0	−1
1UDI	E	244	0	−1
1UDI	I	1	1	−1
1UDI	I	2	1	−1
1UDI	I	3	0	−1
1UDI	I	4	1	−1
1UDI	I	5	1	−1
1UDI	I	6	0	−1
1UDI	I	7	0	−1
1UDI	I	8	1	−1

1UDI	I	9	1	−1
1UDI	I	10	1	−1
1UDI	I	11	1	−1
1UDI	I	12	1	−1
1UDI	I	13	1	−1
1UDI	I	14	1	−1
1UDI	I	15	0	−1
1UDI	I	16	1	1
1UDI	I	17	1	1
1UDI	I	18	1	1
1UDI	I	19	1	1
1UDI	I	20	1	1
1UDI	I	21	1	1
1UDI	I	22	1	1
1UDI	I	23	1	1
1UDI	I	24	1	1
1UDI	I	25	0	−1
1UDI	I	26	1	1
1UDI	I	27	1	1
1UDI	I	28	0	−1
1UDI	I	29	1	−1
1UDI	I	30	1	1
1UDI	I	31	1	1
1UDI	I	32	0	−1
1UDI	I	33	1	−1
1UDI	I	34	1	−1
1UDI	I	35	1	−1
1UDI	I	36	0	−1
1UDI	I	37	1	−1
1UDI	I	38	1	−1
1UDI	I	39	1	−1
1UDI	I	40	0	−1
1UDI	I	41	0	−1
1UDI	I	42	0	−1
1UDI	I	43	0	−1
1UDI	I	44	0	−1
1UDI	I	45	0	−1
1UDI	I	46	1	1
1UDI	I	47	0	−1
1UDI	I	48	1	−1
1UDI	I	49	1	−1
1UDI	I	50	1	−1
1UDI	I	51	1	−1
1UDI	I	52	0	−1
1UDI	I	53	1	1
1UDI	I	54	0	−1
1UDI	I	55	0	−1
1UDI	I	56	0	−1
1UDI	I	57	0	−1
1UDI	I	58	0	−1
1UDI	I	59	0	−1
1UDI	I	60	1	1

1UDI	I	61	1	1
1UDI	I	62	1	1
1UDI	I	63	1	−1
1UDI	I	64	1	1
1UDI	I	65	1	−1
1UDI	I	66	1	−1
1UDI	I	67	0	−1
1UDI	I	68	0	−1
1UDI	I	69	0	−1
1UDI	I	70	0	−1
1UDI	I	71	0	−1
1UDI	I	72	1	1
1UDI	I	73	0	−1
1UDI	I	74	1	−1
1UDI	I	75	1	−1
1UDI	I	76	1	−1
1UDI	I	77	1	−1
1UDI	I	78	1	1
1UDI	I	79	1	−1
1UDI	I	80	1	−1
1UDI	I	81	1	−1
1UDI	I	82	1	−1
1UDI	I	83	1	−1
1YCS	A	4	1	−1
1YCS	A	5	1	−1
1YCS	A	6	1	−1
1YCS	A	7	1	−1
1YCS	A	8	1	−1
1YCS	A	9	1	−1
1YCS	A	10	1	−1
1YCS	A	11	1	−1
1YCS	A	12	1	−1
1YCS	A	13	1	−1
1YCS	A	14	0	−1
1YCS	A	15	0	−1
1YCS	A	16	0	−1
1YCS	A	17	1	−1
1YCS	A	18	0	−1
1YCS	A	19	0	−1
1YCS	A	20	0	−1
1YCS	A	21	1	−1
1YCS	A	22	1	−1
1YCS	A	23	1	−1
1YCS	A	24	1	−1
1YCS	A	25	0	−1
1YCS	A	26	1	−1
1YCS	A	27	1	−1
1YCS	A	28	1	−1
1YCS	A	29	0	−1
1YCS	A	30	1	−1
1YCS	A	31	0	−1
1YCS	A	32	0	−1

1YCS	A	33	0	−1
1YCS	A	34	0	−1
1YCS	A	35	1	−1
1YCS	A	36	1	−1
1YCS	A	37	1	−1
1YCS	A	38	1	−1
1YCS	A	39	0	−1
1YCS	A	40	0	−1
1YCS	A	41	0	−1
1YCS	A	42	0	−1
1YCS	A	43	0	−1
1YCS	A	44	1	−1
1YCS	A	45	1	−1
1YCS	A	46	1	−1
1YCS	A	47	1	−1
1YCS	A	48	0	−1
1YCS	A	49	0	−1
1YCS	A	50	0	−1
1YCS	A	51	0	−1
1YCS	A	52	0	−1
1YCS	A	53	1	−1
1YCS	A	54	0	−1
1YCS	A	55	1	−1
1YCS	A	56	1	−1
1YCS	A	57	1	−1
1YCS	A	58	0	−1
1YCS	A	59	1	−1
1YCS	A	60	1	−1
1YCS	A	61	1	−1
1YCS	A	62	0	−1
1YCS	A	63	0	−1
1YCS	A	64	0	−1
1YCS	A	65	0	−1
1YCS	A	66	0	−1
1YCS	A	67	0	−1
1YCS	A	68	0	−1
1YCS	A	69	0	−1
1YCS	A	70	0	−1
1YCS	A	71	1	−1
1YCS	A	72	1	1
1YCS	A	73	1	−1
1YCS	A	74	1	1
1YCS	A	75	1	1
1YCS	A	76	0	−1
1YCS	A	77	1	−1
1YCS	A	78	1	1
1YCS	A	79	0	−1
1YCS	A	80	0	−1
1YCS	A	81	1	−1
1YCS	A	82	0	−1
1YCS	A	83	0	−1
1YCS	A	84	1	−1

1YCS	A	85	1	1
1YCS	A	86	0	−1
1YCS	A	87	1	−1
1YCS	A	88	1	1
1YCS	A	89	1	1
1YCS	A	90	1	1
1YCS	A	91	1	−1
1YCS	A	92	1	−1
1YCS	A	93	1	−1
1YCS	A	94	1	−1
1YCS	A	95	1	−1
1YCS	A	96	0	−1
1YCS	A	97	0	−1
1YCS	A	98	1	−1
1YCS	A	99	0	−1
1YCS	A	100	0	−1
1YCS	A	101	0	−1
1YCS	A	102	0	−1
1YCS	A	103	0	−1
1YCS	A	104	0	−1
1YCS	A	105	1	−1
1YCS	A	106	1	−1
1YCS	A	107	0	−1
1YCS	A	108	1	−1
1YCS	A	109	1	−1
1YCS	A	110	0	−1
1YCS	A	111	1	−1
1YCS	A	112	0	−1
1YCS	A	113	1	−1
1YCS	A	114	1	−1
1YCS	A	115	1	−1
1YCS	A	116	1	−1
1YCS	A	117	1	−1
1YCS	A	118	1	−1
1YCS	A	119	1	−1
1YCS	A	120	0	−1
1YCS	A	121	0	−1
1YCS	A	122	0	−1
1YCS	A	123	0	−1
1YCS	A	124	0	−1
1YCS	A	125	0	−1
1YCS	A	126	0	−1
1YCS	A	127	0	−1
1YCS	A	128	1	−1
1YCS	A	129	1	−1
1YCS	A	130	1	−1
1YCS	A	131	1	−1
1YCS	A	132	1	−1
1YCS	A	133	1	−1
1YCS	A	134	1	−1
1YCS	A	135	1	−1
1YCS	A	136	0	−1

1YCS	A	137	0	−1
1YCS	A	138	1	−1
1YCS	A	139	0	−1
1YCS	A	140	1	−1
1YCS	A	141	0	−1
1YCS	A	142	0	−1
1YCS	A	143	0	−1
1YCS	A	144	0	−1
1YCS	A	145	0	−1
1YCS	A	146	1	1
1YCS	A	147	0	−1
1YCS	A	148	1	1
1YCS	A	149	0	−1
1YCS	A	150	1	1
1YCS	A	151	1	−1
1YCS	A	152	0	−1
1YCS	A	153	0	−1
1YCS	A	154	1	1
1YCS	A	155	1	1
1YCS	A	156	0	−1
1YCS	A	157	1	1
1YCS	A	158	0	−1
1YCS	A	159	0	−1
1YCS	A	160	0	−1
1YCS	A	161	0	−1
1YCS	A	162	0	−1
1YCS	A	163	0	−1
1YCS	A	164	0	−1
1YCS	A	165	0	−1
1YCS	A	166	0	−1
1YCS	A	167	1	−1
1YCS	A	168	1	−1
1YCS	A	169	1	−1
1YCS	A	170	1	−1
1YCS	A	171	1	−1
1YCS	A	172	0	−1
1YCS	A	173	0	−1
1YCS	A	174	0	−1
1YCS	A	175	0	−1
1YCS	A	176	0	−1
1YCS	A	177	0	−1
1YCS	A	178	0	−1
1YCS	A	179	0	−1
1YCS	A	180	1	1
1YCS	A	181	0	−1
1YCS	A	182	0	−1
1YCS	A	183	1	−1
1YCS	A	184	0	−1
1YCS	A	185	0	−1
1YCS	A	186	0	−1
1YCS	A	187	1	1
1YCS	A	188	0	−1

1YCS	A	189	0	−1
1YCS	A	190	1	−1
1YCS	A	191	1	−1
1YCS	A	192	1	−1
1YCS	A	193	1	−1
1YCS	A	194	1	−1
1YCS	B	37	1	−1
1YCS	B	38	1	−1
1YCS	B	39	1	−1
1YCS	B	40	1	−1
1YCS	B	41	0	−1
1YCS	B	42	0	−1
1YCS	B	43	1	−1
1YCS	B	44	0	−1
1YCS	B	45	0	−1
1YCS	B	46	1	−1
1YCS	B	47	1	−1
1YCS	B	48	1	−1
1YCS	B	49	1	−1
1YCS	B	50	1	−1
1YCS	B	51	1	−1
1YCS	B	52	1	−1
1YCS	B	53	0	−1
1YCS	B	54	1	−1
1YCS	B	55	1	−1
1YCS	B	56	0	−1
1YCS	B	57	0	−1
1YCS	B	58	1	−1
1YCS	B	59	1	−1
1YCS	B	60	1	−1
1YCS	B	61	1	−1
1YCS	B	62	1	−1
1YCS	B	63	0	−1
1YCS	B	64	1	−1
1YCS	B	65	0	−1
1YCS	B	66	1	−1
1YCS	B	67	0	−1
1YCS	B	68	1	−1
1YCS	B	69	1	−1
1YCS	B	70	0	−1
1YCS	B	71	0	−1
1YCS	B	72	0	−1
1YCS	B	73	0	−1
1YCS	B	74	0	−1
1YCS	B	75	0	−1
1YCS	B	76	0	−1
1YCS	B	77	0	−1
1YCS	B	78	0	−1
1YCS	B	79	1	−1
1YCS	B	80	1	−1
1YCS	B	81	1	−1
1YCS	B	82	0	−1

1YCS	B	83	1	−1
1YCS	B	84	1	−1
1YCS	B	85	0	−1
1YCS	B	86	0	−1
1YCS	B	87	1	−1
1YCS	B	88	0	−1
1YCS	B	89	0	−1
1YCS	B	90	0	−1
1YCS	B	91	1	−1
1YCS	B	92	1	−1
1YCS	B	93	1	−1
1YCS	B	94	0	−1
1YCS	B	95	1	−1
1YCS	B	96	0	−1
1YCS	B	97	0	−1
1YCS	B	98	0	−1
1YCS	B	99	1	−1
1YCS	B	100	0	−1
1YCS	B	101	1	−1
1YCS	B	102	1	−1
1YCS	B	103	0	−1
1YCS	B	104	1	−1
1YCS	B	105	0	−1
1YCS	B	106	0	−1
1YCS	B	107	0	−1
1YCS	B	108	0	−1
1YCS	B	109	0	−1
1YCS	B	110	0	−1
1YCS	B	111	0	−1
1YCS	B	112	1	−1
1YCS	B	113	1	−1
1YCS	B	114	0	−1
1YCS	B	115	1	−1
1YCS	B	116	1	−1
1YCS	B	117	1	−1
1YCS	B	118	0	−1
1YCS	B	119	0	−1
1YCS	B	120	1	−1
1YCS	B	121	0	−1
1YCS	B	122	0	−1
1YCS	B	123	0	−1
1YCS	B	124	1	−1
1YCS	B	125	0	−1
1YCS	B	126	0	−1
1YCS	B	127	0	−1
1YCS	B	128	0	−1
1YCS	B	129	0	−1
1YCS	B	130	0	−1
1YCS	B	131	0	−1
1YCS	B	132	1	1
1YCS	B	133	0	−1
1YCS	B	134	1	1

1YCS	B	135	1	1
1YCS	B	136	1	1
1YCS	B	137	1	1
1YCS	B	138	1	−1
1YCS	B	139	0	−1
1YCS	B	140	0	−1
1YCS	B	141	0	−1
1YCS	B	142	1	−1
1YCS	B	143	0	−1
1YCS	B	144	0	−1
1YCS	B	145	0	−1
1YCS	B	146	1	−1
1YCS	B	147	1	−1
1YCS	B	148	1	−1
1YCS	B	149	1	−1
1YCS	B	150	1	−1
1YCS	B	151	1	−1
1YCS	B	152	0	−1
1YCS	B	153	1	−1
1YCS	B	154	0	−1
1YCS	B	155	0	−1
1YCS	B	156	1	−1
1YCS	B	157	0	−1
1YCS	B	158	0	−1
1YCS	B	159	1	−1
1YCS	B	160	0	−1
1YCS	B	161	0	−1
1YCS	B	162	0	−1
1YCS	B	163	1	−1
1YCS	B	164	1	−1
1YCS	B	165	0	−1
1YCS	B	166	0	−1
1YCS	B	167	1	−1
1YCS	B	168	0	−1
1YCS	B	169	1	−1
1YCS	B	170	1	−1
1YCS	B	171	0	−1
1YCS	B	172	1	−1
1YCS	B	173	0	−1
1YCS	B	174	1	−1
1YCS	B	175	0	−1
1YCS	B	176	0	−1
1YCS	B	177	1	−1
1YCS	B	178	1	−1
1YCS	B	179	0	−1
1YCS	B	180	1	−1
1YCS	B	181	1	−1
1YCS	B	182	1	1
1YCS	B	183	1	1
1YCS	B	184	1	1
1YCS	B	185	1	1
1YCS	B	186	0	−1

1YCS	B	187	0	−1
1YCS	B	188	1	−1
1YCS	B	189	0	−1
1YCS	B	190	1	−1
1YCS	B	191	1	−1
1YCS	B	192	1	−1
1YCS	B	193	1	−1
1YCS	B	194	1	−1
1YCS	B	195	0	−1
1YCS	B	196	1	−1
1YCS	B	197	0	−1
1YCS	B	198	1	−1
1YCS	B	199	1	−1
1YCS	B	200	0	−1
1YCS	B	201	1	−1
1YCS	B	202	1	1
1YCS	B	203	1	1
1YCS	B	204	1	1
1YCS	B	205	1	1
1YCS	B	206	1	1
1YCS	B	207	1	1
1YCS	B	208	1	1
1YCS	B	209	0	−1
1YCS	B	210	1	−1
1YCS	B	211	0	−1
1YCS	B	212	1	−1
1YCS	B	213	0	−1
1YCS	B	214	1	−1
1YCS	B	215	1	−1
1YCS	B	216	1	−1
1YCS	B	217	1	−1
1YCS	B	218	0	−1
1YCS	B	219	0	−1
1YCS	B	220	0	−1
1YCS	B	221	0	−1
1YCS	B	222	0	−1
1YCS	B	223	1	1
1YCS	B	224	0	−1
1YCS	B	225	0	−1
1YCS	B	226	0	−1
1YCS	B	227	0	−1
1YCS	B	228	1	−1
1YCS	B	229	1	−1
1YDR	E	15	1	−1
1YDR	E	16	1	−1
1YDR	E	17	1	−1
1YDR	E	18	1	−1
1YDR	E	19	0	−1
1YDR	E	20	1	−1
1YDR	E	21	1	−1
1YDR	E	22	0	−1
1YDR	E	23	1	−1

1YDR	E	24	1	−1
1YDR	E	25	1	−1
1YDR	E	26	0	−1
1YDR	E	27	1	−1
1YDR	E	28	1	−1
1YDR	E	29	1	−1
1YDR	E	30	0	−1
1YDR	E	31	1	−1
1YDR	E	32	1	−1
1YDR	E	33	1	−1
1YDR	E	34	1	−1
1YDR	E	35	1	−1
1YDR	E	36	1	−1
1YDR	E	37	1	−1
1YDR	E	38	0	−1
1YDR	E	39	1	−1
1YDR	E	40	1	−1
1YDR	E	41	1	−1
1YDR	E	42	0	−1
1YDR	E	43	0	−1
1YDR	E	44	1	−1
1YDR	E	45	1	−1
1YDR	E	46	1	−1
1YDR	E	47	0	−1
1YDR	E	48	0	−1
1YDR	E	49	0	−1
1YDR	E	50	0	−1
1YDR	E	51	1	−1
1YDR	E	52	1	1
1YDR	E	53	1	1
1YDR	E	54	0	−1
1YDR	E	55	1	−1
1YDR	E	56	0	−1
1YDR	E	57	1	−1
1YDR	E	58	0	−1
1YDR	E	59	0	−1
1YDR	E	60	0	−1
1YDR	E	61	1	−1
1YDR	E	62	0	−1
1YDR	E	63	1	−1
1YDR	E	64	1	−1
1YDR	E	65	1	−1
1YDR	E	66	1	−1
1YDR	E	67	1	−1
1YDR	E	68	1	−1
1YDR	E	69	0	−1
1YDR	E	70	0	−1
1YDR	E	71	0	−1
1YDR	E	72	0	−1
1YDR	E	73	0	−1
1YDR	E	74	0	−1
1YDR	E	75	0	−1

1YDR	E	76	0	−1
1YDR	E	77	1	−1
1YDR	E	78	1	−1
1YDR	E	79	0	−1
1YDR	E	80	1	−1
1YDR	E	81	1	−1
1YDR	E	82	1	1
1YDR	E	83	1	1
1YDR	E	84	0	−1
1YDR	E	85	1	−1
1YDR	E	86	1	−1
1YDR	E	87	0	−1
1YDR	E	88	0	−1
1YDR	E	89	0	−1
1YDR	E	90	0	−1
1YDR	E	91	0	−1
1YDR	E	92	0	−1
1YDR	E	93	1	−1
1YDR	E	94	0	−1
1YDR	E	95	0	−1
1YDR	E	96	0	−1
1YDR	E	97	0	−1
1YDR	E	98	0	−1
1YDR	E	99	1	−1
1YDR	E	100	0	−1
1YDR	E	101	0	−1
1YDR	E	102	0	−1
1YDR	E	103	0	−1
1YDR	E	104	0	−1
1YDR	E	105	1	−1
1YDR	E	106	0	−1
1YDR	E	107	1	−1
1YDR	E	108	0	−1
1YDR	E	109	0	−1
1YDR	E	110	0	−1
1YDR	E	111	0	−1
1YDR	E	112	0	−1
1YDR	E	113	1	−1
1YDR	E	114	0	−1
1YDR	E	115	0	−1
1YDR	E	116	0	−1
1YDR	E	117	0	−1
1YDR	E	118	0	−1
1YDR	E	119	0	−1
1YDR	E	120	0	−1
1YDR	E	121	1	−1
1YDR	E	122	0	−1
1YDR	E	123	0	−1
1YDR	E	124	0	−1
1YDR	E	125	0	−1
1YDR	E	126	0	−1
1YDR	E	127	0	−1

1YDR	E	128	0	−1
1YDR	E	129	1	1
1YDR	E	130	0	−1
1YDR	E	131	0	−1
1YDR	E	132	0	−1
1YDR	E	133	1	1
1YDR	E	134	1	−1
1YDR	E	135	1	−1
1YDR	E	136	1	1
1YDR	E	137	1	−1
1YDR	E	138	0	−1
1YDR	E	139	1	−1
1YDR	E	140	0	−1
1YDR	E	141	1	−1
1YDR	E	142	0	−1
1YDR	E	143	0	−1
1YDR	E	144	0	−1
1YDR	E	145	0	−1
1YDR	E	146	0	−1
1YDR	E	147	0	−1
1YDR	E	148	0	−1
1YDR	E	149	0	−1
1YDR	E	150	0	−1
1YDR	E	151	0	−1
1YDR	E	152	0	−1
1YDR	E	153	0	−1
1YDR	E	154	0	−1
1YDR	E	155	0	−1
1YDR	E	156	0	−1
1YDR	E	157	0	−1
1YDR	E	158	0	−1
1YDR	E	159	1	−1
1YDR	E	160	0	−1
1YDR	E	161	0	−1
1YDR	E	162	0	−1
1YDR	E	163	0	−1
1YDR	E	164	0	−1
1YDR	E	165	0	−1
1YDR	E	166	0	−1
1YDR	E	167	0	−1
1YDR	E	168	0	−1
1YDR	E	169	0	−1
1YDR	E	170	1	1
1YDR	E	171	0	−1
1YDR	E	172	0	−1
1YDR	E	173	0	−1
1YDR	E	174	0	−1
1YDR	E	175	0	−1
1YDR	E	176	1	−1
1YDR	E	177	1	−1
1YDR	E	178	0	−1
1YDR	E	179	0	−1

1YDR	E	180	0	−1
1YDR	E	181	0	−1
1YDR	E	182	0	−1
1YDR	E	183	0	−1
1YDR	E	184	1	−1
1YDR	E	185	0	−1
1YDR	E	186	0	−1
1YDR	E	187	1	1
1YDR	E	188	0	−1
1YDR	E	189	0	−1
1YDR	E	190	0	−1
1YDR	E	191	0	−1
1YDR	E	192	1	−1
1YDR	E	193	1	−1
1YDR	E	194	1	−1
1YDR	E	195	0	−1
1YDR	E	196	1	−1
1YDR	E	198	1	1
1YDR	E	199	0	−1
1YDR	E	200	1	1
1YDR	E	201	0	−1
1YDR	E	202	0	−1
1YDR	E	203	1	1
1YDR	E	204	0	−1
1YDR	E	205	0	−1
1YDR	E	206	0	−1
1YDR	E	207	0	−1
1YDR	E	208	0	−1
1YDR	E	209	0	−1
1YDR	E	210	1	−1
1YDR	E	211	1	−1
1YDR	E	212	1	−1
1YDR	E	213	1	−1
1YDR	E	214	0	−1
1YDR	E	215	0	−1
1YDR	E	216	1	−1
1YDR	E	217	0	−1
1YDR	E	218	0	−1
1YDR	E	219	0	−1
1YDR	E	220	0	−1
1YDR	E	221	0	−1
1YDR	E	222	0	−1
1YDR	E	223	0	−1
1YDR	E	224	0	−1
1YDR	E	225	0	−1
1YDR	E	226	0	−1
1YDR	E	227	0	−1
1YDR	E	228	0	−1
1YDR	E	229	0	−1
1YDR	E	230	0	−1
1YDR	E	231	0	−1
1YDR	E	232	0	−1

1YDR	E	233	0	−1
1YDR	E	234	0	−1
1YDR	E	235	1	1
1YDR	E	236	0	−1
1YDR	E	237	0	−1
1YDR	E	238	0	−1
1YDR	E	239	1	1
1YDR	E	240	0	−1
1YDR	E	241	1	1
1YDR	E	242	1	−1
1YDR	E	243	1	1
1YDR	E	244	1	−1
1YDR	E	245	1	−1
1YDR	E	246	0	−1
1YDR	E	247	1	1
1YDR	E	248	1	−1
1YDR	E	249	1	−1
1YDR	E	250	0	−1
1YDR	E	251	1	−1
1YDR	E	252	1	−1
1YDR	E	253	1	−1
1YDR	E	254	1	−1
1YDR	E	255	1	−1
1YDR	E	256	1	−1
1YDR	E	257	0	−1
1YDR	E	258	0	−1
1YDR	E	259	1	−1
1YDR	E	260	1	−1
1YDR	E	261	0	−1
1YDR	E	262	1	−1
1YDR	E	263	1	−1
1YDR	E	264	1	−1
1YDR	E	265	0	−1
1YDR	E	266	0	−1
1YDR	E	267	1	−1
1YDR	E	268	0	−1
1YDR	E	269	0	−1
1YDR	E	270	1	−1
1YDR	E	271	0	−1
1YDR	E	272	0	−1
1YDR	E	273	0	−1
1YDR	E	274	0	−1
1YDR	E	275	1	−1
1YDR	E	276	1	−1
1YDR	E	277	0	−1
1YDR	E	278	1	−1
1YDR	E	279	1	−1
1YDR	E	280	0	−1
1YDR	E	281	0	−1
1YDR	E	282	0	−1
1YDR	E	283	1	−1
1YDR	E	284	0	−1

1YDR	E	285	1	−1
1YDR	E	286	1	−1
1YDR	E	287	1	−1
1YDR	E	288	0	−1
1YDR	E	289	1	−1
1YDR	E	290	0	−1
1YDR	E	291	0	−1
1YDR	E	292	0	−1
1YDR	E	293	1	−1
1YDR	E	294	0	−1
1YDR	E	295	1	−1
1YDR	E	296	0	−1
1YDR	E	297	0	−1
1YDR	E	298	1	−1
1YDR	E	299	1	−1
1YDR	E	300	0	−1
1YDR	E	301	1	−1
1YDR	E	302	0	−1
1YDR	E	303	1	−1
1YDR	E	304	0	−1
1YDR	E	305	0	−1
1YDR	E	306	1	−1
1YDR	E	307	1	−1
1YDR	E	308	0	−1
1YDR	E	309	1	−1
1YDR	E	310	0	−1
1YDR	E	311	1	−1
1YDR	E	312	0	−1
1YDR	E	313	1	−1
1YDR	E	314	0	−1
1YDR	E	315	1	−1
1YDR	E	316	0	−1
1YDR	E	317	1	−1
1YDR	E	318	0	−1
1YDR	E	319	1	−1
1YDR	E	320	1	−1
1YDR	E	321	1	−1
1YDR	E	322	0	−1
1YDR	E	323	0	−1
1YDR	E	324	0	−1
1YDR	E	325	1	−1
1YDR	E	326	0	−1
1YDR	E	327	0	−1
1YDR	E	328	1	1
1YDR	E	329	1	−1
1YDR	E	330	1	1
1YDR	E	331	1	−1
1YDR	E	332	1	−1
1YDR	E	333	1	−1
1YDR	E	334	1	−1
1YDR	E	335	1	−1
1YDR	E	336	1	−1

1YDR	E	337	1	−1
1YDR	E	339	1	−1
1YDR	E	340	1	−1
1YDR	E	341	1	−1
1YDR	E	342	1	−1
1YDR	E	343	0	−1
1YDR	E	344	0	−1
1YDR	E	345	1	−1
1YDR	E	346	1	−1
1YDR	E	347	0	−1
1YDR	E	348	1	−1
1YDR	E	349	1	−1
1YDR	E	350	0	−1
1YDR	I	1	1	−1
1YDR	I	2	1	1
1YDR	I	3	1	1
1YDR	I	4	1	−1
1YDR	I	5	1	1
1YDR	I	6	1	1
1YDR	I	7	1	−1
1YDR	I	8	1	1
1YDR	I	9	1	1
1YDR	I	10	1	1
1YDR	I	11	1	1
1YDR	I	12	1	1
1YDR	I	13	1	1
1YDR	I	14	1	1
1YDR	I	15	1	1
1YDR	I	16	1	1
1YDR	I	17	1	1
1YDR	I	18	1	1
1YDR	I	19	1	1
1YDR	I	20	1	1
1YQV	L	1	1	−1
1YQV	L	2	1	−1
1YQV	L	3	0	−1
1YQV	L	4	1	−1
1YQV	L	5	0	−1
1YQV	L	6	1	−1
1YQV	L	7	0	−1
1YQV	L	8	1	−1
1YQV	L	9	1	−1
1YQV	L	10	1	−1
1YQV	L	11	1	−1
1YQV	L	12	0	−1
1YQV	L	13	1	−1
1YQV	L	14	0	−1
1YQV	L	15	0	−1
1YQV	L	16	1	−1
1YQV	L	17	1	−1
1YQV	L	18	1	−1
1YQV	L	19	1	−1

1YQV	L	20	0	−1
1YQV	L	21	1	−1
1YQV	L	22	0	−1
1YQV	L	23	1	−1
1YQV	L	24	0	−1
1YQV	L	25	1	−1
1YQV	L	26	0	−1
1YQV	L	27	1	−1
1YQV	L	28	1	−1
1YQV	L	29	1	−1
1YQV	L	30	0	−1
1YQV	L	31	1	1
1YQV	L	32	1	1
1YQV	L	33	0	−1
1YQV	L	34	0	−1
1YQV	L	35	0	−1
1YQV	L	36	0	−1
1YQV	L	37	0	−1
1YQV	L	38	0	−1
1YQV	L	39	1	−1
1YQV	L	40	1	−1
1YQV	L	41	1	−1
1YQV	L	42	1	−1
1YQV	L	43	0	−1
1YQV	L	44	0	−1
1YQV	L	45	1	−1
1YQV	L	46	0	−1
1YQV	L	47	0	−1
1YQV	L	48	0	−1
1YQV	L	49	0	−1
1YQV	L	50	0	−1
1YQV	L	51	0	−1
1YQV	L	52	1	−1
1YQV	L	53	1	−1
1YQV	L	54	1	−1
1YQV	L	55	0	−1
1YQV	L	56	1	−1
1YQV	L	57	1	−1
1YQV	L	58	0	−1
1YQV	L	59	1	−1
1YQV	L	60	1	−1
1YQV	L	61	0	−1
1YQV	L	62	0	−1
1YQV	L	63	1	−1
1YQV	L	64	0	−1
1YQV	L	65	1	−1
1YQV	L	66	1	−1
1YQV	L	67	1	−1
1YQV	L	68	1	−1
1YQV	L	69	1	−1
1YQV	L	70	1	−1
1YQV	L	71	0	−1

1YQV	L	72	0	−1
1YQV	L	73	0	−1
1YQV	L	74	0	−1
1YQV	L	75	0	−1
1YQV	L	76	1	−1
1YQV	L	77	1	−1
1YQV	L	78	0	−1
1YQV	L	79	1	−1
1YQV	L	80	1	−1
1YQV	L	81	1	−1
1YQV	L	82	0	−1
1YQV	L	83	0	−1
1YQV	L	84	0	−1
1YQV	L	85	0	−1
1YQV	L	86	0	−1
1YQV	L	87	0	−1
1YQV	L	88	0	−1
1YQV	L	89	0	−1
1YQV	L	90	0	−1
1YQV	L	91	1	1
1YQV	L	92	1	1
1YQV	L	93	1	1
1YQV	L	94	0	−1
1YQV	L	95	0	−1
1YQV	L	96	0	−1
1YQV	L	97	0	−1
1YQV	L	98	0	−1
1YQV	L	99	1	−1
1YQV	L	100	0	−1
1YQV	L	101	0	−1
1YQV	L	102	1	−1
1YQV	L	103	0	−1
1YQV	L	104	0	−1
1YQV	L	105	0	−1
1YQV	L	106	1	−1
1YQV	L	107	1	−1
1YQV	L	108	1	−1
1YQV	L	109	1	−1
1YQV	L	110	0	−1
1YQV	L	111	1	−1
1YQV	L	112	0	−1
1YQV	L	113	1	−1
1YQV	L	114	0	−1
1YQV	L	115	0	−1
1YQV	L	116	0	−1
1YQV	L	117	0	−1
1YQV	L	118	0	−1
1YQV	L	119	0	−1
1YQV	L	120	0	−1
1YQV	L	121	1	−1
1YQV	L	122	1	−1
1YQV	L	123	0	−1

1YQV	L	124	0	−1
1YQV	L	125	1	−1
1YQV	L	126	1	−1
1YQV	L	127	1	−1
1YQV	L	128	0	−1
1YQV	L	129	0	−1
1YQV	L	130	0	−1
1YQV	L	131	0	−1
1YQV	L	132	0	−1
1YQV	L	133	0	−1
1YQV	L	134	0	−1
1YQV	L	135	0	−1
1YQV	L	136	0	−1
1YQV	L	137	1	−1
1YQV	L	138	0	−1
1YQV	L	139	0	−1
1YQV	L	140	0	−1
1YQV	L	141	1	−1
1YQV	L	142	1	−1
1YQV	L	143	0	−1
1YQV	L	144	1	−1
1YQV	L	145	0	−1
1YQV	L	146	1	−1
1YQV	L	147	0	−1
1YQV	L	148	0	−1
1YQV	L	149	0	−1
1YQV	L	150	1	−1
1YQV	L	151	1	−1
1YQV	L	152	1	−1
1YQV	L	153	1	−1
1YQV	L	154	0	−1
1YQV	L	155	1	−1
1YQV	L	156	1	−1
1YQV	L	157	1	−1
1YQV	L	158	1	−1
1YQV	L	159	1	−1
1YQV	L	160	1	−1
1YQV	L	161	0	−1
1YQV	L	162	1	−1
1YQV	L	163	0	−1
1YQV	L	164	1	−1
1YQV	L	165	0	−1
1YQV	L	166	0	−1
1YQV	L	167	1	−1
1YQV	L	168	1	−1
1YQV	L	169	0	−1
1YQV	L	170	0	−1
1YQV	L	171	0	−1
1YQV	L	172	0	−1
1YQV	L	173	0	−1
1YQV	L	174	0	−1
1YQV	L	175	0	−1

1YQV	L	176	0	−1
1YQV	L	177	0	−1
1YQV	L	178	0	−1
1YQV	L	179	1	−1
1YQV	L	180	0	−1
1YQV	L	181	1	−1
1YQV	L	182	1	−1
1YQV	L	183	1	−1
1YQV	L	184	0	−1
1YQV	L	185	0	−1
1YQV	L	186	1	−1
1YQV	L	187	1	−1
1YQV	L	188	0	−1
1YQV	L	189	1	−1
1YQV	L	190	0	−1
1YQV	L	191	0	−1
1YQV	L	192	0	−1
1YQV	L	193	0	−1
1YQV	L	194	0	−1
1YQV	L	195	0	−1
1YQV	L	196	1	−1
1YQV	L	197	0	−1
1YQV	L	198	1	−1
1YQV	L	199	1	−1
1YQV	L	200	1	−1
1YQV	L	201	1	−1
1YQV	L	202	1	−1
1YQV	L	203	1	−1
1YQV	L	204	1	−1
1YQV	L	205	1	−1
1YQV	L	206	1	−1
1YQV	L	207	1	−1
1YQV	L	208	0	−1
1YQV	L	211	1	−1
1YQV	L	213	1	−1
1YQV	L	217	1	−1
1YQV	H	1	1	−1
1YQV	H	2	1	−1
1YQV	H	3	1	−1
1YQV	H	4	0	−1
1YQV	H	5	1	−1
1YQV	H	6	0	−1
1YQV	H	7	0	−1
1YQV	H	8	1	−1
1YQV	H	9	1	−1
1YQV	H	10	1	−1
1YQV	H	11	1	−1
1YQV	H	12	1	−1
1YQV	H	13	1	−1
1YQV	H	14	1	−1
1YQV	H	15	1	−1
1YQV	H	16	1	−1

1YQV	H	17	1	−1
1YQV	H	18	0	−1
1YQV	H	19	1	−1
1YQV	H	20	0	−1
1YQV	H	21	1	−1
1YQV	H	22	0	−1
1YQV	H	23	1	−1
1YQV	H	24	0	−1
1YQV	H	25	1	−1
1YQV	H	26	1	−1
1YQV	H	27	0	−1
1YQV	H	28	1	−1
1YQV	H	29	0	−1
1YQV	H	30	1	1
1YQV	H	31	1	1
1YQV	H	32	0	−1
1YQV	H	33	1	1
1YQV	H	34	0	−1
1YQV	H	35	0	−1
1YQV	H	36	0	−1
1YQV	H	37	0	−1
1YQV	H	38	0	−1
1YQV	H	39	0	−1
1YQV	H	40	0	−1
1YQV	H	41	1	−1
1YQV	H	42	1	−1
1YQV	H	43	1	−1
1YQV	H	44	0	−1
1YQV	H	45	0	−1
1YQV	H	46	0	−1
1YQV	H	47	0	−1
1YQV	H	48	0	−1
1YQV	H	49	0	−1
1YQV	H	50	0	−1
1YQV	H	51	0	−1
1YQV	H	52	0	−1
1YQV	H	53	0	−1
1YQV	H	54	1	−1
1YQV	H	55	1	1
1YQV	H	56	1	1
1YQV	H	57	1	1
1YQV	H	58	1	1
1YQV	H	59	1	1
1YQV	H	60	0	−1
1YQV	H	61	0	−1
1YQV	H	62	1	−1
1YQV	H	63	1	−1
1YQV	H	64	0	−1
1YQV	H	65	1	−1
1YQV	H	66	1	−1
1YQV	H	67	0	−1
1YQV	H	68	0	−1

1YQV	H	69	1	−1
1YQV	H	70	0	−1
1YQV	H	71	1	−1
1YQV	H	72	1	−1
1YQV	H	73	1	−1
1YQV	H	74	1	−1
1YQV	H	75	1	−1
1YQV	H	76	1	−1
1YQV	H	77	1	−1
1YQV	H	78	0	−1
1YQV	H	79	0	−1
1YQV	H	80	0	−1
1YQV	H	81	0	−1
1YQV	H	82	1	−1
1YQV	H	83	0	−1
1YQV	H	84	0	−1
1YQV	H	85	1	−1
1YQV	H	86	0	−1
1YQV	H	87	1	−1
1YQV	H	88	1	−1
1YQV	H	89	1	−1
1YQV	H	90	0	−1
1YQV	H	91	1	−1
1YQV	H	92	0	−1
1YQV	H	93	1	−1
1YQV	H	94	0	−1
1YQV	H	95	0	−1
1YQV	H	96	0	−1
1YQV	H	97	0	−1
1YQV	H	98	0	−1
1YQV	H	99	0	−1
1YQV	H	100	0	−1
1YQV	H	101	1	1
1YQV	H	102	1	1
1YQV	H	103	0	−1
1YQV	H	104	0	−1
1YQV	H	105	1	−1
1YQV	H	106	0	−1
1YQV	H	107	0	−1
1YQV	H	108	1	−1
1YQV	H	109	0	−1
1YQV	H	110	0	−1
1YQV	H	111	1	−1
1YQV	H	112	0	−1
1YQV	H	113	0	−1
1YQV	H	114	0	−1
1YQV	H	115	0	−1
1YQV	H	116	1	−1
1YQV	H	117	0	−1
1YQV	H	118	1	−1
1YQV	H	119	1	−1
1YQV	H	120	1	−1

1YQV	H	121	1	−1
1YQV	H	122	0	−1
1YQV	H	123	1	−1
1YQV	H	124	0	−1
1YQV	H	125	0	−1
1YQV	H	126	0	−1
1YQV	H	127	0	−1
1YQV	H	128	0	−1
1YQV	H	129	1	−1
1YQV	H	130	1	−1
1YQV	H	131	1	−1
1YQV	H	132	1	−1
1YQV	H	133	1	−1
1YQV	H	134	1	−1
1YQV	H	135	1	−1
1YQV	H	136	1	−1
1YQV	H	137	1	−1
1YQV	H	138	1	−1
1YQV	H	139	0	−1
1YQV	H	140	0	−1
1YQV	H	141	0	−1
1YQV	H	142	0	−1
1YQV	H	143	0	−1
1YQV	H	144	0	−1
1YQV	H	145	0	−1
1YQV	H	146	1	−1
1YQV	H	147	0	−1
1YQV	H	148	0	−1
1YQV	H	149	0	−1
1YQV	H	150	0	−1
1YQV	H	151	1	−1
1YQV	H	152	1	−1
1YQV	H	153	0	−1
1YQV	H	154	1	−1
1YQV	H	155	0	−1
1YQV	H	156	1	−1
1YQV	H	157	0	−1
1YQV	H	158	1	−1
1YQV	H	159	1	−1
1YQV	H	160	1	−1
1YQV	H	161	1	−1
1YQV	H	162	0	−1
1YQV	H	163	1	−1
1YQV	H	164	1	−1
1YQV	H	165	1	−1
1YQV	H	166	0	−1
1YQV	H	167	0	−1
1YQV	H	168	1	−1
1YQV	H	169	0	−1
1YQV	H	170	1	−1
1YQV	H	171	0	−1
1YQV	H	172	1	−1

1YQV	H	173	1	−1
1YQV	H	174	1	−1
1YQV	H	175	1	−1
1YQV	H	176	1	−1
1YQV	H	177	1	−1
1YQV	H	178	0	−1
1YQV	H	179	0	−1
1YQV	H	180	0	−1
1YQV	H	181	0	−1
1YQV	H	182	0	−1
1YQV	H	183	0	−1
1YQV	H	184	0	−1
1YQV	H	185	0	−1
1YQV	H	186	0	−1
1YQV	H	187	1	−1
1YQV	H	188	0	−1
1YQV	H	189	1	−1
1YQV	H	190	0	−1
1YQV	H	191	0	−1
1YQV	H	192	1	−1
1YQV	H	193	1	−1
1YQV	H	194	1	−1
1YQV	H	195	1	−1
1YQV	H	196	0	−1
1YQV	H	197	0	−1
1YQV	H	198	0	−1
1YQV	H	199	0	−1
1YQV	H	200	0	−1
1YQV	H	201	0	−1
1YQV	H	202	0	−1
1YQV	H	203	1	−1
1YQV	H	204	1	−1
1YQV	H	205	0	−1
1YQV	H	206	1	−1
1YQV	H	207	1	−1
1YQV	H	208	1	−1
1YQV	H	209	1	−1
1YQV	H	210	1	−1
1YQV	H	211	0	−1
1YQV	H	212	1	−1
1YQV	H	213	0	−1
1YQV	H	214	1	−1
1YQV	H	215	1	−1
2BTF	A	2	1	−1
2BTF	A	3	1	−1
2BTF	A	4	1	−1
2BTF	A	5	1	−1
2BTF	A	6	0	−1
2BTF	A	7	0	−1
2BTF	A	8	0	−1
2BTF	A	9	0	−1
2BTF	A	10	0	−1

2BTF	A	11	0	−1
2BTF	A	12	0	−1
2BTF	A	13	0	−1
2BTF	A	14	0	−1
2BTF	A	15	0	−1
2BTF	A	16	1	−1
2BTF	A	17	0	−1
2BTF	A	18	0	−1
2BTF	A	19	0	−1
2BTF	A	20	0	−1
2BTF	A	21	0	−1
2BTF	A	22	0	−1
2BTF	A	23	1	−1
2BTF	A	24	1	−1
2BTF	A	25	1	−1
2BTF	A	26	1	−1
2BTF	A	27	0	−1
2BTF	A	28	1	−1
2BTF	A	29	0	−1
2BTF	A	30	1	−1
2BTF	A	31	0	−1
2BTF	A	32	0	−1
2BTF	A	33	0	−1
2BTF	A	34	0	−1
2BTF	A	35	0	−1
2BTF	A	36	0	−1
2BTF	A	37	1	−1
2BTF	A	38	0	−1
2BTF	A	39	1	−1
2BTF	A	40	1	−1
2BTF	A	41	1	−1
2BTF	A	42	1	−1
2BTF	A	43	1	−1
2BTF	A	44	1	−1
2BTF	A	45	1	−1
2BTF	A	46	1	−1
2BTF	A	47	1	−1
2BTF	A	48	1	−1
2BTF	A	49	1	−1
2BTF	A	50	1	−1
2BTF	A	51	0	−1
2BTF	A	52	0	−1
2BTF	A	53	0	−1
2BTF	A	54	0	−1
2BTF	A	55	0	−1
2BTF	A	56	1	−1
2BTF	A	57	1	−1
2BTF	A	58	0	−1
2BTF	A	59	1	−1
2BTF	A	60	1	−1
2BTF	A	61	1	−1
2BTF	A	62	0	−1

2BTF	A	63	1	−1
2BTF	A	64	1	−1
2BTF	A	65	0	−1
2BTF	A	66	1	−1
2BTF	A	67	0	−1
2BTF	A	68	1	−1
2BTF	A	69	1	−1
2BTF	A	70	0	−1
2BTF	A	71	0	−1
2BTF	A	72	1	−1
2BTF	A	74	1	−1
2BTF	A	75	0	−1
2BTF	A	76	0	−1
2BTF	A	77	0	−1
2BTF	A	78	1	−1
2BTF	A	79	1	−1
2BTF	A	80	0	−1
2BTF	A	81	1	−1
2BTF	A	82	0	−1
2BTF	A	83	0	−1
2BTF	A	84	1	−1
2BTF	A	85	1	−1
2BTF	A	86	0	−1
2BTF	A	87	0	−1
2BTF	A	88	1	−1
2BTF	A	89	0	−1
2BTF	A	90	0	−1
2BTF	A	91	0	−1
2BTF	A	92	1	−1
2BTF	A	93	1	−1
2BTF	A	94	1	−1
2BTF	A	95	0	−1
2BTF	A	96	1	−1
2BTF	A	97	0	−1
2BTF	A	98	0	−1
2BTF	A	99	0	−1
2BTF	A	100	1	−1
2BTF	A	101	1	−1
2BTF	A	102	0	−1
2BTF	A	103	0	−1
2BTF	A	104	0	−1
2BTF	A	105	0	−1
2BTF	A	106	0	−1
2BTF	A	107	0	−1
2BTF	A	108	0	−1
2BTF	A	109	0	−1
2BTF	A	110	0	−1
2BTF	A	111	1	−1
2BTF	A	112	0	−1
2BTF	A	113	1	−1
2BTF	A	114	1	1
2BTF	A	115	1	−1

2BTF	A	116	0	−1
2BTF	A	117	0	−1
2BTF	A	118	1	−1
2BTF	A	119	1	−1
2BTF	A	120	0	−1
2BTF	A	121	0	−1
2BTF	A	122	1	−1
2BTF	A	123	0	−1
2BTF	A	124	0	−1
2BTF	A	125	0	−1
2BTF	A	126	1	−1
2BTF	A	127	1	−1
2BTF	A	128	0	−1
2BTF	A	129	1	−1
2BTF	A	130	0	−1
2BTF	A	131	1	−1
2BTF	A	132	0	−1
2BTF	A	133	0	−1
2BTF	A	134	0	−1
2BTF	A	135	0	−1
2BTF	A	136	0	−1
2BTF	A	137	0	−1
2BTF	A	138	0	−1
2BTF	A	139	0	−1
2BTF	A	140	0	−1
2BTF	A	141	0	−1
2BTF	A	142	0	−1
2BTF	A	143	0	−1
2BTF	A	144	1	−1
2BTF	A	145	1	−1
2BTF	A	146	1	−1
2BTF	A	147	1	−1
2BTF	A	148	0	−1
2BTF	A	149	1	−1
2BTF	A	150	0	−1
2BTF	A	151	0	−1
2BTF	A	152	0	−1
2BTF	A	153	0	−1
2BTF	A	154	0	−1
2BTF	A	155	0	−1
2BTF	A	156	0	−1
2BTF	A	157	1	−1
2BTF	A	158	1	−1
2BTF	A	159	0	−1
2BTF	A	160	0	−1
2BTF	A	161	0	−1
2BTF	A	162	0	−1
2BTF	A	163	0	−1
2BTF	A	164	0	−1
2BTF	A	165	0	−1
2BTF	A	166	0	−1
2BTF	A	167	1	1

2BTF	A	168	1	1
2BTF	A	169	1	−1
2BTF	A	170	1	1
2BTF	A	171	0	−1
2BTF	A	172	0	−1
2BTF	A	173	1	1
2BTF	A	174	1	1
2BTF	A	175	0	−1
2BTF	A	176	0	−1
2BTF	A	177	1	−1
2BTF	A	178	1	−1
2BTF	A	179	0	−1
2BTF	A	180	1	−1
2BTF	A	181	0	−1
2BTF	A	182	0	−1
2BTF	A	183	0	−1
2BTF	A	184	1	−1
2BTF	A	185	0	−1
2BTF	A	186	0	−1
2BTF	A	187	0	−1
2BTF	A	188	1	−1
2BTF	A	189	0	−1
2BTF	A	190	0	−1
2BTF	A	191	0	−1
2BTF	A	192	1	−1
2BTF	A	193	0	−1
2BTF	A	194	0	−1
2BTF	A	195	1	−1
2BTF	A	196	1	−1
2BTF	A	197	1	−1
2BTF	A	198	1	−1
2BTF	A	199	0	−1
2BTF	A	200	1	−1
2BTF	A	201	0	−1
2BTF	A	202	1	−1
2BTF	A	203	1	−1
2BTF	A	204	1	−1
2BTF	A	205	1	−1
2BTF	A	206	0	−1
2BTF	A	207	1	−1
2BTF	A	208	0	−1
2BTF	A	209	0	−1
2BTF	A	210	0	−1
2BTF	A	211	1	−1
2BTF	A	212	0	−1
2BTF	A	213	0	−1
2BTF	A	214	0	−1
2BTF	A	215	1	−1
2BTF	A	216	1	−1
2BTF	A	217	0	−1
2BTF	A	218	0	−1
2BTF	A	219	1	−1

2BTF	A	220	0	−1
2BTF	A	221	0	−1
2BTF	A	222	1	−1
2BTF	A	223	1	−1
2BTF	A	224	0	−1
2BTF	A	225	1	−1
2BTF	A	226	1	−1
2BTF	A	227	1	−1
2BTF	A	228	1	−1
2BTF	A	229	1	−1
2BTF	A	230	1	−1
2BTF	A	231	0	−1
2BTF	A	232	1	−1
2BTF	A	233	1	−1
2BTF	A	234	1	−1
2BTF	A	235	1	−1
2BTF	A	236	1	−1
2BTF	A	237	0	−1
2BTF	A	238	1	−1
2BTF	A	239	1	−1
2BTF	A	240	1	−1
2BTF	A	241	0	−1
2BTF	A	242	1	−1
2BTF	A	243	0	−1
2BTF	A	244	1	−1
2BTF	A	245	1	−1
2BTF	A	246	1	−1
2BTF	A	247	1	−1
2BTF	A	248	1	−1
2BTF	A	249	0	−1
2BTF	A	250	1	−1
2BTF	A	251	0	−1
2BTF	A	252	0	−1
2BTF	A	253	1	−1
2BTF	A	254	0	−1
2BTF	A	255	0	−1
2BTF	A	256	0	−1
2BTF	A	257	0	−1
2BTF	A	258	0	−1
2BTF	A	259	0	−1
2BTF	A	260	0	−1
2BTF	A	261	0	−1
2BTF	A	262	0	−1
2BTF	A	263	0	−1
2BTF	A	264	1	−1
2BTF	A	265	0	−1
2BTF	A	266	1	−1
2BTF	A	267	1	−1
2BTF	A	268	1	−1
2BTF	A	269	1	−1
2BTF	A	270	1	−1
2BTF	A	271	1	−1

2BTF	A	272	1	−1
2BTF	A	273	1	−1
2BTF	A	274	0	−1
2BTF	A	275	0	−1
2BTF	A	276	0	−1
2BTF	A	277	1	−1
2BTF	A	278	0	−1
2BTF	A	279	0	−1
2BTF	A	280	0	−1
2BTF	A	281	1	−1
2BTF	A	282	0	−1
2BTF	A	283	0	−1
2BTF	A	284	1	1
2BTF	A	285	1	1
2BTF	A	286	0	−1
2BTF	A	287	1	1
2BTF	A	288	1	1
2BTF	A	289	1	1
2BTF	A	290	0	−1
2BTF	A	291	0	−1
2BTF	A	292	1	−1
2BTF	A	293	1	−1
2BTF	A	294	0	−1
2BTF	A	295	0	−1
2BTF	A	296	0	−1
2BTF	A	297	0	−1
2BTF	A	298	0	−1
2BTF	A	299	0	−1
2BTF	A	300	0	−1
2BTF	A	301	0	−1
2BTF	A	302	0	−1
2BTF	A	303	1	−1
2BTF	A	304	0	−1
2BTF	A	305	0	−1
2BTF	A	306	1	−1
2BTF	A	307	0	−1
2BTF	A	308	1	−1
2BTF	A	309	1	−1
2BTF	A	310	0	−1
2BTF	A	311	1	−1
2BTF	A	312	1	−1
2BTF	A	313	0	−1
2BTF	A	314	0	−1
2BTF	A	315	1	−1
2BTF	A	316	1	−1
2BTF	A	317	0	−1
2BTF	A	318	0	−1
2BTF	A	319	1	−1
2BTF	A	320	1	−1
2BTF	A	321	1	−1
2BTF	A	322	0	−1
2BTF	A	323	1	−1

2BTF	A	324	1	−1
2BTF	A	325	1	−1
2BTF	A	326	1	−1
2BTF	A	327	1	−1
2BTF	A	328	0	−1
2BTF	A	329	1	−1
2BTF	A	330	0	−1
2BTF	A	331	0	−1
2BTF	A	332	1	−1
2BTF	A	333	0	−1
2BTF	A	334	1	−1
2BTF	A	335	1	−1
2BTF	A	336	0	−1
2BTF	A	337	1	−1
2BTF	A	338	1	−1
2BTF	A	339	0	−1
2BTF	A	340	0	−1
2BTF	A	341	0	−1
2BTF	A	342	0	−1
2BTF	A	343	0	−1
2BTF	A	344	0	−1
2BTF	A	345	0	−1
2BTF	A	346	1	−1
2BTF	A	347	0	−1
2BTF	A	348	0	−1
2BTF	A	349	1	−1
2BTF	A	350	1	−1
2BTF	A	351	1	−1
2BTF	A	352	1	−1
2BTF	A	353	0	−1
2BTF	A	354	1	−1
2BTF	A	355	1	−1
2BTF	A	356	1	1
2BTF	A	357	0	−1
2BTF	A	358	0	−1
2BTF	A	359	0	−1
2BTF	A	360	1	−1
2BTF	A	361	1	−1
2BTF	A	362	0	−1
2BTF	A	363	0	−1
2BTF	A	364	1	−1
2BTF	A	365	1	1
2BTF	A	366	1	1
2BTF	A	367	0	−1
2BTF	A	368	0	−1
2BTF	A	369	1	1
2BTF	A	370	0	−1
2BTF	A	371	0	−1
2BTF	A	372	0	−1
2BTF	A	373	1	1
2BTF	A	374	1	1
2BTF	A	375	0	−1

2BTF	P	2	1	−1
2BTF	P	3	1	−1
2BTF	P	4	1	−1
2BTF	P	5	1	−1
2BTF	P	6	1	−1
2BTF	P	7	0	−1
2BTF	P	8	0	−1
2BTF	P	9	1	−1
2BTF	P	10	1	−1
2BTF	P	11	0	−1
2BTF	P	12	1	−1
2BTF	P	13	1	−1
2BTF	P	14	1	−1
2BTF	P	15	1	−1
2BTF	P	16	0	−1
2BTF	P	17	0	−1
2BTF	P	18	1	−1
2BTF	P	19	0	−1
2BTF	P	20	0	−1
2BTF	P	21	0	−1
2BTF	P	22	0	−1
2BTF	P	23	0	−1
2BTF	P	24	0	−1
2BTF	P	25	0	−1
2BTF	P	26	1	−1
2BTF	P	27	1	−1
2BTF	P	28	1	−1
2BTF	P	29	0	−1
2BTF	P	30	1	−1
2BTF	P	31	1	−1
2BTF	P	32	1	−1
2BTF	P	33	0	−1
2BTF	P	34	0	−1
2BTF	P	35	0	−1
2BTF	P	36	1	−1
2BTF	P	37	1	−1
2BTF	P	38	1	−1
2BTF	P	39	1	−1
2BTF	P	40	0	−1
2BTF	P	41	1	−1
2BTF	P	42	1	−1
2BTF	P	43	0	−1
2BTF	P	44	1	−1
2BTF	P	45	1	−1
2BTF	P	46	1	−1
2BTF	P	47	0	−1
2BTF	P	48	0	−1
2BTF	P	49	1	−1
2BTF	P	50	1	−1
2BTF	P	51	0	−1
2BTF	P	52	1	−1
2BTF	P	53	0	−1

2BTF	P	54	1	−1
2BTF	P	55	1	−1
2BTF	P	56	0	−1
2BTF	P	57	1	−1
2BTF	P	58	1	−1
2BTF	P	59	0	−1
2BTF	P	60	1	1
2BTF	P	61	1	1
2BTF	P	62	1	1
2BTF	P	63	1	1
2BTF	P	64	0	−1
2BTF	P	65	1	−1
2BTF	P	66	0	−1
2BTF	P	67	0	−1
2BTF	P	68	1	−1
2BTF	P	69	1	−1
2BTF	P	70	1	1
2BTF	P	71	0	−1
2BTF	P	72	1	1
2BTF	P	73	0	−1
2BTF	P	74	1	1
2BTF	P	75	1	1
2BTF	P	76	0	−1
2BTF	P	77	0	−1
2BTF	P	78	0	−1
2BTF	P	79	1	−1
2BTF	P	80	1	−1
2BTF	P	81	1	−1
2BTF	P	82	1	1
2BTF	P	83	1	1
2BTF	P	84	0	−1
2BTF	P	85	0	−1
2BTF	P	86	0	−1
2BTF	P	87	0	−1
2BTF	P	88	0	−1
2BTF	P	89	1	1
2BTF	P	90	0	−1
2BTF	P	91	1	1
2BTF	P	92	1	1
2BTF	P	93	1	−1
2BTF	P	94	1	−1
2BTF	P	95	1	−1
2BTF	P	96	1	−1
2BTF	P	97	1	−1
2BTF	P	98	0	−1
2BTF	P	99	0	−1
2BTF	P	100	0	−1
2BTF	P	101	0	−1
2BTF	P	102	0	−1
2BTF	P	103	0	−1
2BTF	P	104	0	−1
2BTF	P	105	0	−1

2BTF	P	106	0	−1
2BTF	P	107	1	−1
2BTF	P	108	1	−1
2BTF	P	109	0	−1
2BTF	P	110	0	−1
2BTF	P	111	0	−1
2BTF	P	112	0	−1
2BTF	P	113	0	−1
2BTF	P	114	0	−1
2BTF	P	115	0	−1
2BTF	P	116	1	−1
2BTF	P	117	1	−1
2BTF	P	118	1	−1
2BTF	P	119	0	−1
2BTF	P	120	1	1
2BTF	P	121	0	−1
2BTF	P	122	1	1
2BTF	P	123	1	1
2BTF	P	124	0	−1
2BTF	P	125	0	−1
2BTF	P	126	1	1
2BTF	P	127	0	−1
2BTF	P	128	0	−1
2BTF	P	129	1	1
2BTF	P	130	1	1
2BTF	P	131	0	−1
2BTF	P	132	0	−1
2BTF	P	133	0	−1
2BTF	P	134	1	−1
2BTF	P	135	0	−1
2BTF	P	136	1	−1
2BTF	P	137	1	−1
2BTF	P	138	1	−1
2BTF	P	139	1	−1
2BTF	P	140	1	−1
2JEL	L	1	1	−1
2JEL	L	2	0	−1
2JEL	L	3	1	−1
2JEL	L	4	0	−1
2JEL	L	5	1	−1
2JEL	L	6	0	−1
2JEL	L	7	1	−1
2JEL	L	8	1	−1
2JEL	L	9	1	−1
2JEL	L	10	1	−1
2JEL	L	11	0	−1
2JEL	L	12	1	−1
2JEL	L	13	0	−1
2JEL	L	14	1	−1
2JEL	L	15	1	−1
2JEL	L	16	1	−1
2JEL	L	17	1	−1

2JEL	L	18	1	−1
2JEL	L	19	0	−1
2JEL	L	20	1	−1
2JEL	L	21	0	−1
2JEL	L	22	1	−1
2JEL	L	23	0	−1
2JEL	L	24	1	−1
2JEL	L	25	0	−1
2JEL	L	26	1	−1
2JEL	L	27	1	−1
2JEL	L	28	1	−1
2JEL	L	29	0	−1
2JEL	L	30	1	−1
2JEL	L	31	1	1
2JEL	L	32	1	1
2JEL	L	33	1	1
2JEL	L	34	1	−1
2JEL	L	35	1	1
2JEL	L	36	0	−1
2JEL	L	37	0	−1
2JEL	L	38	0	−1
2JEL	L	39	0	−1
2JEL	L	40	0	−1
2JEL	L	41	0	−1
2JEL	L	42	0	−1
2JEL	L	43	0	−1
2JEL	L	44	1	−1
2JEL	L	45	1	−1
2JEL	L	46	1	−1
2JEL	L	47	1	−1
2JEL	L	48	1	−1
2JEL	L	49	0	−1
2JEL	L	50	1	−1
2JEL	L	51	0	−1
2JEL	L	52	0	−1
2JEL	L	53	0	−1
2JEL	L	54	0	−1
2JEL	L	55	0	−1
2JEL	L	56	0	−1
2JEL	L	57	1	−1
2JEL	L	58	1	−1
2JEL	L	59	1	−1
2JEL	L	60	0	−1
2JEL	L	61	1	−1
2JEL	L	62	1	−1
2JEL	L	63	0	−1
2JEL	L	64	1	−1
2JEL	L	65	1	−1
2JEL	L	66	0	−1
2JEL	L	67	0	−1
2JEL	L	68	1	−1
2JEL	L	69	0	−1

2JEL	L	70	1	−1
2JEL	L	71	1	−1
2JEL	L	72	1	−1
2JEL	L	73	1	−1
2JEL	L	74	1	−1
2JEL	L	75	1	−1
2JEL	L	76	0	−1
2JEL	L	77	0	−1
2JEL	L	78	0	−1
2JEL	L	79	1	−1
2JEL	L	80	0	−1
2JEL	L	81	1	−1
2JEL	L	82	1	−1
2JEL	L	83	0	−1
2JEL	L	84	1	−1
2JEL	L	85	1	−1
2JEL	L	86	1	−1
2JEL	L	87	0	−1
2JEL	L	88	0	−1
2JEL	L	89	0	−1
2JEL	L	90	1	−1
2JEL	L	91	0	−1
2JEL	L	92	0	−1
2JEL	L	93	0	−1
2JEL	L	94	0	−1
2JEL	L	95	0	−1
2JEL	L	96	0	−1
2JEL	L	97	0	−1
2JEL	L	98	1	−1
2JEL	L	99	1	1
2JEL	L	100	0	−1
2JEL	L	101	0	−1
2JEL	L	102	0	−1
2JEL	L	103	0	−1
2JEL	L	104	0	−1
2JEL	L	105	1	−1
2JEL	L	106	0	−1
2JEL	L	107	0	−1
2JEL	L	108	1	−1
2JEL	L	109	0	−1
2JEL	L	110	0	−1
2JEL	L	111	0	−1
2JEL	L	112	1	−1
2JEL	L	113	1	−1
2JEL	L	114	1	−1
2JEL	L	115	1	−1
2JEL	L	116	0	−1
2JEL	L	117	1	−1
2JEL	L	118	0	−1
2JEL	L	119	1	−1
2JEL	L	120	0	−1
2JEL	L	121	0	−1

2JEL	L	122	0	−1
2JEL	L	123	0	−1
2JEL	L	124	0	−1
2JEL	L	125	0	−1
2JEL	L	126	0	−1
2JEL	L	127	1	−1
2JEL	L	128	1	−1
2JEL	L	129	0	−1
2JEL	L	130	0	−1
2JEL	L	131	1	−1
2JEL	L	132	1	−1
2JEL	L	133	1	−1
2JEL	L	134	0	−1
2JEL	L	135	0	−1
2JEL	L	136	0	−1
2JEL	L	137	0	−1
2JEL	L	138	0	−1
2JEL	L	139	0	−1
2JEL	L	140	0	−1
2JEL	L	141	0	−1
2JEL	L	142	0	−1
2JEL	L	143	1	−1
2JEL	L	144	0	−1
2JEL	L	145	0	−1
2JEL	L	146	0	−1
2JEL	L	147	1	−1
2JEL	L	148	1	−1
2JEL	L	149	0	−1
2JEL	L	150	1	−1
2JEL	L	151	0	−1
2JEL	L	152	1	−1
2JEL	L	153	0	−1
2JEL	L	154	0	−1
2JEL	L	155	0	−1
2JEL	L	156	1	−1
2JEL	L	157	1	−1
2JEL	L	158	1	−1
2JEL	L	159	1	−1
2JEL	L	160	1	−1
2JEL	L	161	1	−1
2JEL	L	162	1	−1
2JEL	L	163	1	−1
2JEL	L	164	1	−1
2JEL	L	165	0	−1
2JEL	L	166	1	−1
2JEL	L	167	0	−1
2JEL	L	168	1	−1
2JEL	L	169	0	−1
2JEL	L	170	1	−1
2JEL	L	171	0	−1
2JEL	L	172	1	−1
2JEL	L	173	1	−1

2JEL	L	174	1	−1
2JEL	L	175	1	−1
2JEL	L	176	0	−1
2JEL	L	177	0	−1
2JEL	L	178	0	−1
2JEL	L	179	0	−1
2JEL	L	180	0	−1
2JEL	L	181	0	−1
2JEL	L	182	0	−1
2JEL	L	183	0	−1
2JEL	L	184	0	−1
2JEL	L	185	1	−1
2JEL	L	186	0	−1
2JEL	L	187	1	−1
2JEL	L	188	1	−1
2JEL	L	189	1	−1
2JEL	L	190	1	−1
2JEL	L	191	0	−1
2JEL	L	192	1	−1
2JEL	L	193	1	−1
2JEL	L	194	0	−1
2JEL	L	195	0	−1
2JEL	L	196	0	−1
2JEL	L	197	0	−1
2JEL	L	198	0	−1
2JEL	L	199	0	−1
2JEL	L	200	0	−1
2JEL	L	201	0	−1
2JEL	L	202	1	−1
2JEL	L	203	0	−1
2JEL	L	204	1	−1
2JEL	L	205	1	−1
2JEL	L	206	1	−1
2JEL	L	207	1	−1
2JEL	L	208	1	−1
2JEL	L	209	1	−1
2JEL	L	210	0	−1
2JEL	L	211	1	−1
2JEL	L	212	1	−1
2JEL	L	213	1	−1
2JEL	L	214	0	−1
2JEL	L	215	1	−1
2JEL	L	216	1	−1
2JEL	L	217	1	−1
2JEL	H	1	1	−1
2JEL	H	2	1	−1
2JEL	H	3	1	−1
2JEL	H	4	0	−1
2JEL	H	5	1	−1
2JEL	H	6	0	−1
2JEL	H	7	0	−1
2JEL	H	8	1	−1

2JEL	H	9	1	−1
2JEL	H	10	1	−1
2JEL	H	11	1	−1
2JEL	H	12	0	−1
2JEL	H	13	1	−1
2JEL	H	14	1	−1
2JEL	H	15	1	−1
2JEL	H	16	0	−1
2JEL	H	17	1	−1
2JEL	H	18	0	−1
2JEL	H	19	1	−1
2JEL	H	20	0	−1
2JEL	H	21	1	−1
2JEL	H	22	0	−1
2JEL	H	23	1	−1
2JEL	H	24	0	−1
2JEL	H	25	1	−1
2JEL	H	26	1	−1
2JEL	H	27	0	−1
2JEL	H	28	1	−1
2JEL	H	29	0	−1
2JEL	H	30	1	1
2JEL	H	31	1	1
2JEL	H	32	0	−1
2JEL	H	33	0	−1
2JEL	H	34	0	−1
2JEL	H	35	0	−1
2JEL	H	36	0	−1
2JEL	H	37	0	−1
2JEL	H	38	0	−1
2JEL	H	39	0	−1
2JEL	H	40	0	−1
2JEL	H	41	1	−1
2JEL	H	42	1	−1
2JEL	H	43	1	−1
2JEL	H	44	1	−1
2JEL	H	45	0	−1
2JEL	H	46	1	−1
2JEL	H	47	0	−1
2JEL	H	48	0	−1
2JEL	H	49	0	−1
2JEL	H	50	0	−1
2JEL	H	51	0	−1
2JEL	H	52	0	−1
2JEL	H	53	0	−1
2JEL	H	54	1	1
2JEL	H	55	1	1
2JEL	H	56	1	−1
2JEL	H	57	1	1
2JEL	H	58	1	−1
2JEL	H	59	1	1
2JEL	H	60	0	−1

2JEL	H	61	0	−1
2JEL	H	62	1	−1
2JEL	H	63	1	−1
2JEL	H	64	0	−1
2JEL	H	65	1	−1
2JEL	H	66	1	−1
2JEL	H	67	0	−1
2JEL	H	68	0	−1
2JEL	H	69	1	−1
2JEL	H	70	0	−1
2JEL	H	71	1	−1
2JEL	H	72	1	−1
2JEL	H	73	1	−1
2JEL	H	74	1	−1
2JEL	H	75	1	−1
2JEL	H	76	1	−1
2JEL	H	77	1	−1
2JEL	H	78	0	−1
2JEL	H	79	0	−1
2JEL	H	80	1	−1
2JEL	H	81	0	−1
2JEL	H	82	1	−1
2JEL	H	83	0	−1
2JEL	H	84	0	−1
2JEL	H	85	1	−1
2JEL	H	86	0	−1
2JEL	H	87	1	−1
2JEL	H	88	1	−1
2JEL	H	89	1	−1
2JEL	H	90	0	−1
2JEL	H	91	1	−1
2JEL	H	92	0	−1
2JEL	H	93	1	−1
2JEL	H	94	0	−1
2JEL	H	95	0	−1
2JEL	H	96	0	−1
2JEL	H	97	0	−1
2JEL	H	98	0	−1
2JEL	H	99	0	−1
2JEL	H	100	1	1
2JEL	H	101	1	1
2JEL	H	102	1	1
2JEL	H	103	0	−1
2JEL	H	104	0	−1
2JEL	H	105	0	−1
2JEL	H	106	0	−1
2JEL	H	107	0	−1
2JEL	H	108	0	−1
2JEL	H	109	0	−1
2JEL	H	110	1	−1
2JEL	H	111	0	−1
2JEL	H	112	0	−1

2JEL	H	113	1	−1
2JEL	H	114	0	−1
2JEL	H	115	0	−1
2JEL	H	116	0	−1
2JEL	H	117	1	−1
2JEL	H	118	1	−1
2JEL	H	119	0	−1
2JEL	H	120	1	−1
2JEL	H	121	1	−1
2JEL	H	122	1	−1
2JEL	H	123	1	−1
2JEL	H	124	0	−1
2JEL	H	125	1	−1
2JEL	H	126	0	−1
2JEL	H	127	0	−1
2JEL	H	128	0	−1
2JEL	H	129	0	−1
2JEL	H	130	0	−1
2JEL	H	131	1	−1
2JEL	H	132	1	−1
2JEL	H	133	1	−1
2JEL	H	134	1	−1
2JEL	H	135	1	−1
2JEL	H	136	1	−1
2JEL	H	137	1	−1
2JEL	H	138	1	−1
2JEL	H	139	1	−1
2JEL	H	140	1	−1
2JEL	H	141	0	−1
2JEL	H	142	0	−1
2JEL	H	143	0	−1
2JEL	H	144	0	−1
2JEL	H	145	0	−1
2JEL	H	146	0	−1
2JEL	H	147	0	−1
2JEL	H	148	1	−1
2JEL	H	149	0	−1
2JEL	H	150	0	−1
2JEL	H	151	0	−1
2JEL	H	152	0	−1
2JEL	H	153	1	−1
2JEL	H	154	1	−1
2JEL	H	155	0	−1
2JEL	H	156	1	−1
2JEL	H	157	0	−1
2JEL	H	158	1	−1
2JEL	H	159	0	−1
2JEL	H	160	1	−1
2JEL	H	161	1	−1
2JEL	H	162	1	−1
2JEL	H	163	1	−1
2JEL	H	164	1	−1

2JEL	H	165	1	−1
2JEL	H	166	1	−1
2JEL	H	167	1	−1
2JEL	H	168	1	−1
2JEL	H	169	0	−1
2JEL	H	170	1	−1
2JEL	H	171	0	−1
2JEL	H	172	1	−1
2JEL	H	173	0	−1
2JEL	H	174	1	−1
2JEL	H	175	1	−1
2JEL	H	176	1	−1
2JEL	H	177	1	−1
2JEL	H	178	1	−1
2JEL	H	179	1	−1
2JEL	H	180	0	−1
2JEL	H	181	0	−1
2JEL	H	182	0	−1
2JEL	H	183	0	−1
2JEL	H	184	0	−1
2JEL	H	185	0	−1
2JEL	H	186	0	−1
2JEL	H	187	0	−1
2JEL	H	188	0	−1
2JEL	H	189	1	−1
2JEL	H	190	0	−1
2JEL	H	191	1	−1
2JEL	H	192	0	−1
2JEL	H	193	0	−1
2JEL	H	194	1	−1
2JEL	H	195	1	−1
2JEL	H	196	1	−1
2JEL	H	197	1	−1
2JEL	H	198	0	−1
2JEL	H	199	0	−1
2JEL	H	200	0	−1
2JEL	H	201	0	−1
2JEL	H	202	0	−1
2JEL	H	203	0	−1
2JEL	H	204	0	−1
2JEL	H	205	1	−1
2JEL	H	206	1	−1
2JEL	H	207	0	−1
2JEL	H	208	1	−1
2JEL	H	209	0	−1
2JEL	H	210	1	−1
2JEL	H	211	0	−1
2JEL	H	212	1	−1
2JEL	H	213	1	−1
2JEL	H	214	1	−1
2JEL	H	215	0	−1
2JEL	H	216	1	−1

2JEL	H	217	1	−1
2JEL	H	218	1	−1
2JEL	P	1	1	1
2JEL	P	2	1	1
2JEL	P	3	1	1
2JEL	P	4	1	1
2JEL	P	5	1	1
2JEL	P	6	0	−1
2JEL	P	7	1	−1
2JEL	P	8	0	−1
2JEL	P	9	1	−1
2JEL	P	10	0	−1
2JEL	P	11	1	−1
2JEL	P	12	1	−1
2JEL	P	13	0	−1
2JEL	P	14	0	−1
2JEL	P	15	1	−1
2JEL	P	16	1	−1
2JEL	P	17	1	−1
2JEL	P	18	0	−1
2JEL	P	19	0	−1
2JEL	P	20	1	−1
2JEL	P	21	1	−1
2JEL	P	22	0	−1
2JEL	P	23	0	−1
2JEL	P	24	1	−1
2JEL	P	25	1	−1
2JEL	P	26	0	−1
2JEL	P	27	1	−1
2JEL	P	28	1	−1
2JEL	P	29	0	−1
2JEL	P	30	1	−1
2JEL	P	31	0	−1
2JEL	P	32	1	−1
2JEL	P	33	0	−1
2JEL	P	34	1	1
2JEL	P	35	0	−1
2JEL	P	36	1	1
2JEL	P	37	0	−1
2JEL	P	38	1	−1
2JEL	P	39	1	−1
2JEL	P	40	1	−1
2JEL	P	41	1	1
2JEL	P	42	0	−1
2JEL	P	43	1	−1
2JEL	P	44	0	−1
2JEL	P	45	1	−1
2JEL	P	46	1	−1
2JEL	P	47	1	−1
2JEL	P	48	1	−1
2JEL	P	49	1	−1
2JEL	P	50	0	−1

2JEL	P	51	1	−1
2JEL	P	52	1	−1
2JEL	P	53	0	−1
2JEL	P	54	1	−1
2JEL	P	55	0	−1
2JEL	P	56	1	−1
2JEL	P	57	1	−1
2JEL	P	58	1	−1
2JEL	P	59	0	−1
2JEL	P	60	1	−1
2JEL	P	61	0	−1
2JEL	P	62	0	−1
2JEL	P	63	0	−1
2JEL	P	64	0	−1
2JEL	P	65	0	−1
2JEL	P	66	1	1
2JEL	P	67	1	1
2JEL	P	68	1	1
2JEL	P	69	0	−1
2JEL	P	70	0	−1
2JEL	P	71	1	1
2JEL	P	72	1	1
2JEL	P	73	0	−1
2JEL	P	74	0	−1
2JEL	P	75	1	1
2JEL	P	76	1	1
2JEL	P	77	0	−1
2JEL	P	78	0	−1
2JEL	P	79	1	1
2JEL	P	80	0	−1
2JEL	P	81	0	−1
2JEL	P	82	1	−1
2JEL	P	83	1	−1
2JEL	P	84	1	−1
2JEL	P	85	1	−1
2SIC	I	1	1	−1
2SIC	I	2	1	−1
2SIC	I	3	1	−1
2SIC	I	4	0	−1
2SIC	I	5	1	−1
2SIC	I	6	0	−1
2SIC	I	7	1	−1
2SIC	I	8	0	−1
2SIC	I	9	0	−1
2SIC	I	10	0	−1
2SIC	I	11	0	−1
2SIC	I	12	1	−1
2SIC	I	13	0	−1
2SIC	I	14	1	−1
2SIC	I	15	1	−1
2SIC	I	16	1	−1
2SIC	I	17	1	−1

2SIC	I	18	1	−1
2SIC	I	19	0	−1
2SIC	I	20	1	−1
2SIC	I	21	1	−1
2SIC	I	22	1	−1
2SIC	I	23	1	−1
2SIC	I	24	1	−1
2SIC	I	25	0	−1
2SIC	I	26	1	−1
2SIC	I	27	0	−1
2SIC	I	28	1	−1
2SIC	I	29	0	−1
2SIC	I	30	0	−1
2SIC	I	31	1	−1
2SIC	I	32	1	−1
2SIC	I	33	1	−1
2SIC	I	34	1	−1
2SIC	I	35	1	−1
2SIC	I	36	1	−1
2SIC	I	37	0	−1
2SIC	I	38	1	−1
2SIC	I	39	0	−1
2SIC	I	40	0	−1
2SIC	I	41	1	−1
2SIC	I	42	1	−1
2SIC	I	43	0	−1
2SIC	I	44	0	−1
2SIC	I	45	1	−1
2SIC	I	46	0	−1
2SIC	I	47	0	−1
2SIC	I	48	1	−1
2SIC	I	49	1	−1
2SIC	I	50	1	−1
2SIC	I	51	1	−1
2SIC	I	52	0	−1
2SIC	I	53	1	−1
2SIC	I	54	0	−1
2SIC	I	55	1	−1
2SIC	I	56	1	−1
2SIC	I	57	0	−1
2SIC	I	58	1	−1
2SIC	I	59	1	1
2SIC	I	60	1	−1
2SIC	I	61	1	1
2SIC	I	62	1	1
2SIC	I	63	1	1
2SIC	I	64	1	1
2SIC	I	65	0	−1
2SIC	I	66	1	1
2SIC	I	67	1	1
2SIC	I	68	1	1
2SIC	I	69	1	1

2SIC	I	70	1	−1
2SIC	I	71	0	−1
2SIC	I	72	0	−1
2SIC	I	73	0	−1
2SIC	I	74	0	−1
2SIC	I	75	0	−1
2SIC	I	76	0	−1
2SIC	I	77	1	−1
2SIC	I	78	1	−1
2SIC	I	79	0	−1
2SIC	I	80	1	−1
2SIC	I	81	1	−1
2SIC	I	82	1	−1
2SIC	I	83	1	−1
2SIC	I	84	1	−1
2SIC	I	85	0	−1
2SIC	I	86	1	−1
2SIC	I	87	0	−1
2SIC	I	88	1	−1
2SIC	I	89	1	−1
2SIC	I	90	1	−1
2SIC	I	91	0	−1
2SIC	I	92	1	1
2SIC	I	93	0	−1
2SIC	I	94	1	−1
2SIC	I	95	1	1
2SIC	I	96	0	−1
2SIC	I	97	0	−1
2SIC	I	98	1	1
2SIC	I	99	0	−1
2SIC	I	100	0	−1
2SIC	I	101	0	−1
2SIC	I	102	1	−1
2SIC	I	103	0	−1
2SIC	I	104	0	−1
2SIC	I	105	0	−1
2SIC	I	106	1	−1
2SIC	I	107	0	−1
2SNI	E	1	1	−1
2SNI	E	2	0	−1
2SNI	E	3	1	−1
2SNI	E	4	1	−1
2SNI	E	5	0	−1
2SNI	E	6	1	−1
2SNI	E	7	0	−1
2SNI	E	8	0	−1
2SNI	E	9	1	−1
2SNI	E	10	0	−1
2SNI	E	11	0	−1
2SNI	E	12	1	−1
2SNI	E	13	0	−1
2SNI	E	14	1	−1

2SNI	E	15	0	−1
2SNI	E	16	0	−1
2SNI	E	17	0	−1
2SNI	E	18	1	−1
2SNI	E	19	1	−1
2SNI	E	20	1	−1
2SNI	E	21	1	−1
2SNI	E	22	0	−1
2SNI	E	23	0	−1
2SNI	E	24	1	−1
2SNI	E	25	1	−1
2SNI	E	26	0	−1
2SNI	E	27	1	−1
2SNI	E	28	0	−1
2SNI	E	29	0	−1
2SNI	E	30	0	−1
2SNI	E	31	0	−1
2SNI	E	32	0	−1
2SNI	E	33	0	−1
2SNI	E	34	0	−1
2SNI	E	35	0	−1
2SNI	E	36	0	−1
2SNI	E	37	1	−1
2SNI	E	38	1	−1
2SNI	E	39	0	−1
2SNI	E	40	1	−1
2SNI	E	41	0	−1
2SNI	E	42	0	−1
2SNI	E	43	1	−1
2SNI	E	44	0	−1
2SNI	E	45	1	−1
2SNI	E	46	0	−1
2SNI	E	47	1	−1
2SNI	E	48	0	−1
2SNI	E	49	0	−1
2SNI	E	50	0	−1
2SNI	E	51	0	−1
2SNI	E	52	1	−1
2SNI	E	53	1	−1
2SNI	E	54	0	−1
2SNI	E	55	1	−1
2SNI	E	56	1	−1
2SNI	E	57	0	−1
2SNI	E	58	1	−1
2SNI	E	59	1	−1
2SNI	E	60	0	−1
2SNI	E	61	1	−1
2SNI	E	62	1	1
2SNI	E	63	0	−1
2SNI	E	64	0	−1
2SNI	E	65	0	−1
2SNI	E	66	0	−1

2SNI	E	67	0	−1
2SNI	E	68	0	−1
2SNI	E	69	0	−1
2SNI	E	70	0	−1
2SNI	E	71	0	−1
2SNI	E	72	0	−1
2SNI	E	73	0	−1
2SNI	E	74	0	−1
2SNI	E	75	1	−1
2SNI	E	76	1	−1
2SNI	E	77	1	−1
2SNI	E	78	1	−1
2SNI	E	79	1	−1
2SNI	E	80	0	−1
2SNI	E	81	0	−1
2SNI	E	82	0	−1
2SNI	E	83	0	−1
2SNI	E	84	0	−1
2SNI	E	85	0	−1
2SNI	E	86	0	−1
2SNI	E	87	1	−1
2SNI	E	88	0	−1
2SNI	E	89	1	−1
2SNI	E	90	0	−1
2SNI	E	91	0	−1
2SNI	E	92	0	−1
2SNI	E	93	0	−1
2SNI	E	94	0	−1
2SNI	E	95	0	−1
2SNI	E	96	0	−1
2SNI	E	97	0	−1
2SNI	E	98	1	−1
2SNI	E	99	1	1
2SNI	E	100	1	1
2SNI	E	101	1	1
2SNI	E	102	1	1
2SNI	E	103	1	1
2SNI	E	104	1	1
2SNI	E	105	1	−1
2SNI	E	106	0	−1
2SNI	E	107	0	−1
2SNI	E	108	0	−1
2SNI	E	109	1	−1
2SNI	E	110	0	−1
2SNI	E	111	0	−1
2SNI	E	112	1	−1
2SNI	E	113	0	−1
2SNI	E	114	0	−1
2SNI	E	115	0	−1
2SNI	E	116	1	−1
2SNI	E	117	1	−1
2SNI	E	118	1	−1

2SNI	E	119	0	−1
2SNI	E	120	1	−1
2SNI	E	121	0	−1
2SNI	E	122	0	−1
2SNI	E	123	0	−1
2SNI	E	124	0	−1
2SNI	E	125	0	−1
2SNI	E	126	0	−1
2SNI	E	127	1	1
2SNI	E	128	0	−1
2SNI	E	129	1	1
2SNI	E	130	1	1
2SNI	E	131	1	−1
2SNI	E	132	1	−1
2SNI	E	133	1	−1
2SNI	E	134	1	−1
2SNI	E	135	0	−1
2SNI	E	136	1	−1
2SNI	E	137	1	−1
2SNI	E	138	0	−1
2SNI	E	139	0	−1
2SNI	E	140	1	−1
2SNI	E	141	1	−1
2SNI	E	142	0	−1
2SNI	E	143	1	−1
2SNI	E	144	1	−1
2SNI	E	145	1	−1
2SNI	E	146	1	−1
2SNI	E	147	0	−1
2SNI	E	148	0	−1
2SNI	E	149	0	−1
2SNI	E	150	0	−1
2SNI	E	151	0	−1
2SNI	E	152	0	−1
2SNI	E	153	0	−1
2SNI	E	154	0	−1
2SNI	E	155	1	1
2SNI	E	156	1	1
2SNI	E	157	0	−1
2SNI	E	158	1	−1
2SNI	E	159	1	−1
2SNI	E	160	1	−1
2SNI	E	161	1	−1
2SNI	E	162	1	−1
2SNI	E	163	0	−1
2SNI	E	164	0	−1
2SNI	E	165	0	−1
2SNI	E	166	0	−1
2SNI	E	167	0	−1
2SNI	E	168	0	−1
2SNI	E	169	0	−1
2SNI	E	170	0	−1

2SNI	E	171	0	−1
2SNI	E	172	1	−1
2SNI	E	173	0	−1
2SNI	E	174	0	−1
2SNI	E	175	0	−1
2SNI	E	176	0	−1
2SNI	E	177	0	−1
2SNI	E	178	0	−1
2SNI	E	179	0	−1
2SNI	E	180	0	−1
2SNI	E	181	1	−1
2SNI	E	182	1	−1
2SNI	E	183	1	−1
2SNI	E	184	1	−1
2SNI	E	185	1	−1
2SNI	E	186	0	−1
2SNI	E	187	0	−1
2SNI	E	188	1	1
2SNI	E	189	1	1
2SNI	E	190	0	−1
2SNI	E	191	0	−1
2SNI	E	192	0	−1
2SNI	E	193	0	−1
2SNI	E	194	1	−1
2SNI	E	195	0	−1
2SNI	E	196	0	−1
2SNI	E	197	0	−1
2SNI	E	198	0	−1
2SNI	E	199	0	−1
2SNI	E	200	0	−1
2SNI	E	201	0	−1
2SNI	E	202	0	−1
2SNI	E	203	1	−1
2SNI	E	204	1	−1
2SNI	E	205	0	−1
2SNI	E	206	1	−1
2SNI	E	207	0	−1
2SNI	E	208	0	−1
2SNI	E	209	0	−1
2SNI	E	210	0	−1
2SNI	E	211	1	−1
2SNI	E	212	1	−1
2SNI	E	213	1	−1
2SNI	E	214	1	−1
2SNI	E	215	0	−1
2SNI	E	216	1	−1
2SNI	E	217	1	1
2SNI	E	218	1	1
2SNI	E	219	0	−1
2SNI	E	220	0	−1
2SNI	E	221	0	−1
2SNI	E	222	0	−1

2SNI	E	223	0	−1
2SNI	E	224	0	−1
2SNI	E	225	0	−1
2SNI	E	226	0	−1
2SNI	E	227	0	−1
2SNI	E	228	0	−1
2SNI	E	229	0	−1
2SNI	E	230	0	−1
2SNI	E	231	0	−1
2SNI	E	232	0	−1
2SNI	E	233	0	−1
2SNI	E	234	0	−1
2SNI	E	235	0	−1
2SNI	E	236	0	−1
2SNI	E	237	1	−1
2SNI	E	238	0	−1
2SNI	E	239	1	−1
2SNI	E	240	1	−1
2SNI	E	241	1	−1
2SNI	E	242	1	−1
2SNI	E	243	0	−1
2SNI	E	244	1	−1
2SNI	E	245	1	−1
2SNI	E	246	0	−1
2SNI	E	247	0	−1
2SNI	E	248	1	−1
2SNI	E	249	0	−1
2SNI	E	250	0	−1
2SNI	E	251	0	−1
2SNI	E	252	1	−1
2SNI	E	253	1	−1
2SNI	E	254	0	−1
2SNI	E	255	1	−1
2SNI	E	256	1	−1
2SNI	E	257	1	−1
2SNI	E	258	1	−1
2SNI	E	259	1	−1
2SNI	E	260	1	−1
2SNI	E	261	0	−1
2SNI	E	262	1	−1
2SNI	E	263	0	−1
2SNI	E	264	0	−1
2SNI	E	265	1	−1
2SNI	E	266	0	−1
2SNI	E	267	0	−1
2SNI	E	268	0	−1
2SNI	E	269	0	−1
2SNI	E	270	0	−1
2SNI	E	271	1	−1
2SNI	E	272	0	−1
2SNI	E	273	0	−1
2SNI	E	274	0	−1

2SNI	E	275	1	−1
2SNI	I	20	1	−1
2SNI	I	21	1	−1
2SNI	I	22	1	−1
2SNI	I	23	1	−1
2SNI	I	24	0	−1
2SNI	I	25	1	−1
2SNI	I	26	1	−1
2SNI	I	27	0	−1
2SNI	I	28	1	−1
2SNI	I	29	1	−1
2SNI	I	30	1	−1
2SNI	I	31	0	−1
2SNI	I	32	0	−1
2SNI	I	33	1	−1
2SNI	I	34	1	−1
2SNI	I	35	0	−1
2SNI	I	36	1	−1
2SNI	I	37	1	−1
2SNI	I	38	1	−1
2SNI	I	39	0	−1
2SNI	I	40	1	−1
2SNI	I	41	1	−1
2SNI	I	42	1	−1
2SNI	I	43	0	−1
2SNI	I	44	1	−1
2SNI	I	45	1	−1
2SNI	I	46	0	−1
2SNI	I	47	1	−1
2SNI	I	48	1	−1
2SNI	I	49	1	1
2SNI	I	50	1	1
2SNI	I	51	0	−1
2SNI	I	52	1	1
2SNI	I	53	1	1
2SNI	I	54	1	1
2SNI	I	55	1	1
2SNI	I	56	1	1
2SNI	I	57	1	1
2SNI	I	58	1	1
2SNI	I	59	1	1
2SNI	I	60	1	1
2SNI	I	61	1	1
2SNI	I	62	1	1
2SNI	I	63	1	−1
2SNI	I	64	1	−1
2SNI	I	65	0	−1
2SNI	I	66	0	−1
2SNI	I	67	0	−1
2SNI	I	68	0	−1
2SNI	I	69	0	−1
2SNI	I	70	0	−1

2SNI	I	71	1	−1
2SNI	I	72	1	−1
2SNI	I	73	1	−1
2SNI	I	74	1	−1
2SNI	I	75	1	−1
2SNI	I	76	0	−1
2SNI	I	77	1	−1
2SNI	I	78	1	1
2SNI	I	79	1	−1
2SNI	I	80	0	−1
2SNI	I	81	1	1
2SNI	I	82	0	−1
2SNI	I	83	0	−1
2TRC	P	1	1	1
2TRC	P	2	1	1
2TRC	P	3	1	1
2TRC	P	4	1	1
2TRC	P	5	1	1
2TRC	P	6	1	1
2TRC	P	7	1	1
2TRC	P	8	1	1
2TRC	P	9	1	1
2TRC	P	10	1	1
2TRC	P	11	0	−1
2TRC	P	12	1	1
2TRC	P	13	0	−1
2TRC	P	14	1	−1
2TRC	P	15	1	1
2TRC	P	16	0	−1
2TRC	P	17	0	−1
2TRC	P	18	1	−1
2TRC	P	19	0	−1
2TRC	P	20	0	−1
2TRC	P	21	1	−1
2TRC	P	22	1	−1
2TRC	P	23	0	−1
2TRC	P	24	1	−1
2TRC	P	25	1	−1
2TRC	P	26	1	−1
2TRC	P	27	1	−1
2TRC	P	28	1	−1
2TRC	P	29	1	−1
2TRC	P	30	1	−1
2TRC	P	31	1	−1
2TRC	P	32	0	−1
2TRC	P	33	1	−1
2TRC	P	34	1	−1
2TRC	P	35	1	−1
2TRC	P	36	1	−1
2TRC	P	37	1	−1
2TRC	P	38	0	−1
2TRC	P	39	1	−1

2TRC	P	40	0	−1
2TRC	P	41	1	−1
2TRC	P	42	1	−1
2TRC	P	43	0	−1
2TRC	P	44	0	−1
2TRC	P	45	1	−1
2TRC	P	46	1	−1
2TRC	P	47	1	−1
2TRC	P	48	1	−1
2TRC	P	49	1	1
2TRC	P	50	1	1
2TRC	P	51	1	1
2TRC	P	52	1	1
2TRC	P	53	0	−1
2TRC	P	54	1	1
2TRC	P	56	1	1
2TRC	P	57	1	1
2TRC	P	58	0	−1
2TRC	P	60	0	−1
2TRC	P	61	1	−1
2TRC	P	62	1	−1
2TRC	P	63	0	−1
2TRC	P	64	1	1
2TRC	P	65	1	−1
2TRC	P	66	1	−1
2TRC	P	67	1	1
2TRC	P	68	1	−1
2TRC	P	69	1	−1
2TRC	P	70	0	−1
2TRC	P	71	1	−1
2TRC	P	72	1	1
2TRC	P	73	1	−1
2TRC	P	74	1	−1
2TRC	P	75	1	−1
2TRC	P	76	1	−1
2TRC	P	77	0	−1
2TRC	P	78	1	−1
2TRC	P	79	1	−1
2TRC	P	80	0	−1
2TRC	P	81	1	1
2TRC	P	82	1	−1
2TRC	P	83	1	−1
2TRC	P	84	1	1
2TRC	P	86	1	−1
2TRC	P	87	1	−1
2TRC	P	89	1	1
2TRC	P	90	1	−1
2TRC	P	91	1	−1
2TRC	P	92	1	1
2TRC	P	93	1	−1
2TRC	P	94	1	−1
2TRC	P	95	1	−1

2TRC	P	96	1	−1
2TRC	P	97	1	−1
2TRC	P	98	0	−1
2TRC	P	99	0	−1
2TRC	P	100	1	−1
2TRC	P	101	0	−1
2TRC	P	102	1	−1
2TRC	P	103	1	−1
2TRC	P	104	0	−1
2TRC	P	105	1	−1
2TRC	P	106	1	−1
2TRC	P	107	0	−1
2TRC	P	108	1	−1
2TRC	P	109	1	−1
2TRC	P	110	0	−1
2TRC	P	111	1	−1
2TRC	P	112	1	−1
2TRC	P	113	0	−1
2TRC	P	114	0	−1
2TRC	P	115	1	−1
2TRC	P	116	1	−1
2TRC	P	117	0	−1
2TRC	P	118	1	−1
2TRC	P	119	1	1
2TRC	P	120	1	−1
2TRC	P	121	0	−1
2TRC	P	122	0	−1
2TRC	P	123	0	−1
2TRC	P	124	0	−1
2TRC	P	125	0	−1
2TRC	P	126	0	−1
2TRC	P	127	0	−1
2TRC	P	128	0	−1
2TRC	P	129	1	−1
2TRC	P	130	1	−1
2TRC	P	131	1	−1
2TRC	P	132	0	−1
2TRC	P	133	1	−1
2TRC	P	134	0	−1
2TRC	P	135	0	−1
2TRC	P	136	1	−1
2TRC	P	137	1	−1
2TRC	P	138	0	−1
2TRC	P	139	0	−1
2TRC	P	140	1	−1
2TRC	P	141	0	−1
2TRC	P	142	0	−1
2TRC	P	143	1	−1
2TRC	P	144	1	−1
2TRC	P	145	0	−1
2TRC	P	146	0	−1
2TRC	P	147	1	−1

2TRC	P	148	1	−1
2TRC	P	149	0	−1
2TRC	P	150	0	−1
2TRC	P	152	0	−1
2TRC	P	153	0	−1
2TRC	P	154	0	−1
2TRC	P	155	0	−1
2TRC	P	156	0	−1
2TRC	P	157	0	−1
2TRC	P	158	1	−1
2TRC	P	159	0	−1
2TRC	P	160	1	−1
2TRC	P	161	1	−1
2TRC	P	162	0	−1
2TRC	P	163	1	−1
2TRC	P	164	1	−1
2TRC	P	165	1	−1
2TRC	P	166	1	−1
2TRC	P	167	1	−1
2TRC	P	168	1	−1
2TRC	P	169	1	−1
2TRC	P	170	1	−1
2TRC	P	171	1	−1
2TRC	P	172	1	−1
2TRC	P	173	0	−1
2TRC	P	174	0	−1
2TRC	P	175	0	−1
2TRC	P	176	0	−1
2TRC	P	177	0	−1
2TRC	P	178	0	−1
2TRC	P	179	0	−1
2TRC	P	180	1	1
2TRC	P	181	1	1
2TRC	P	182	1	−1
2TRC	P	183	1	1
2TRC	P	184	1	1
2TRC	P	185	1	1
2TRC	P	186	0	−1
2TRC	P	187	1	1
2TRC	P	188	0	−1
2TRC	P	189	1	−1
2TRC	P	190	0	−1
2TRC	P	191	0	−1
2TRC	P	192	0	−1
2TRC	P	193	1	−1
2TRC	P	194	1	1
2TRC	P	195	0	−1
2TRC	P	196	1	−1
2TRC	P	197	1	−1
2TRC	P	198	1	−1
2TRC	P	199	0	−1
2TRC	P	200	1	−1

2TRC	P	201	0	−1
2TRC	P	202	1	−1
2TRC	P	203	1	−1
2TRC	P	204	0	−1
2TRC	P	205	0	−1
2TRC	P	206	1	1
2TRC	P	207	0	−1
2TRC	P	208	0	−1
2TRC	P	209	1	1
2TRC	P	210	1	1
2TRC	P	211	1	1
2TRC	P	212	1	1
2TRC	P	213	0	−1
2TRC	P	214	0	−1
2TRC	P	215	0	−1
2TRC	P	216	1	1
2TRC	P	217	1	1
3HFM	L	1	1	−1
3HFM	L	2	0	−1
3HFM	L	3	1	−1
3HFM	L	4	0	−1
3HFM	L	5	1	−1
3HFM	L	6	0	−1
3HFM	L	7	1	−1
3HFM	L	8	1	−1
3HFM	L	9	1	−1
3HFM	L	10	1	−1
3HFM	L	11	1	−1
3HFM	L	12	1	−1
3HFM	L	13	0	−1
3HFM	L	14	1	−1
3HFM	L	15	1	−1
3HFM	L	16	1	−1
3HFM	L	17	1	−1
3HFM	L	18	1	−1
3HFM	L	19	0	−1
3HFM	L	20	1	−1
3HFM	L	21	0	−1
3HFM	L	22	1	−1
3HFM	L	23	0	−1
3HFM	L	24	1	−1
3HFM	L	25	0	−1
3HFM	L	26	1	−1
3HFM	L	27	1	−1
3HFM	L	28	1	−1
3HFM	L	29	0	−1
3HFM	L	30	1	1
3HFM	L	31	1	1
3HFM	L	32	0	−1
3HFM	L	33	0	−1
3HFM	L	34	0	−1
3HFM	L	35	0	−1

3HFM	L	36	0	−1
3HFM	L	37	0	−1
3HFM	L	38	0	−1
3HFM	L	39	0	−1
3HFM	L	40	1	−1
3HFM	L	41	1	−1
3HFM	L	42	1	−1
3HFM	L	43	0	−1
3HFM	L	44	0	−1
3HFM	L	45	1	−1
3HFM	L	46	0	−1
3HFM	L	47	0	−1
3HFM	L	48	0	−1
3HFM	L	49	0	−1
3HFM	L	50	1	1
3HFM	L	51	0	−1
3HFM	L	52	1	−1
3HFM	L	53	1	1
3HFM	L	54	1	−1
3HFM	L	55	0	−1
3HFM	L	56	1	−1
3HFM	L	57	1	−1
3HFM	L	58	0	−1
3HFM	L	59	0	−1
3HFM	L	60	1	−1
3HFM	L	61	0	−1
3HFM	L	62	0	−1
3HFM	L	63	1	−1
3HFM	L	64	0	−1
3HFM	L	65	1	−1
3HFM	L	66	1	−1
3HFM	L	67	1	1
3HFM	L	68	1	−1
3HFM	L	69	1	−1
3HFM	L	70	1	−1
3HFM	L	71	0	−1
3HFM	L	72	1	−1
3HFM	L	73	0	−1
3HFM	L	74	0	−1
3HFM	L	75	0	−1
3HFM	L	76	1	−1
3HFM	L	77	1	−1
3HFM	L	78	0	−1
3HFM	L	79	1	−1
3HFM	L	80	1	−1
3HFM	L	81	1	−1
3HFM	L	82	0	−1
3HFM	L	83	0	−1
3HFM	L	84	0	−1
3HFM	L	85	0	−1
3HFM	L	86	0	−1
3HFM	L	87	0	−1

3HFM	L	88	0	−1
3HFM	L	89	0	−1
3HFM	L	90	0	−1
3HFM	L	91	0	−1
3HFM	L	92	1	1
3HFM	L	93	1	1
3HFM	L	94	1	1
3HFM	L	95	0	−1
3HFM	L	96	0	−1
3HFM	L	97	0	−1
3HFM	L	98	0	−1
3HFM	L	99	0	−1
3HFM	L	100	1	−1
3HFM	L	101	0	−1
3HFM	L	102	0	−1
3HFM	L	103	0	−1
3HFM	L	104	0	−1
3HFM	L	105	0	−1
3HFM	L	106	0	−1
3HFM	L	107	1	−1
3HFM	L	108	1	−1
3HFM	L	109	1	−1
3HFM	L	110	1	−1
3HFM	L	111	0	−1
3HFM	L	112	1	−1
3HFM	L	113	0	−1
3HFM	L	114	1	−1
3HFM	L	115	0	−1
3HFM	L	116	0	−1
3HFM	L	117	0	−1
3HFM	L	118	0	−1
3HFM	L	119	1	−1
3HFM	L	120	0	−1
3HFM	L	121	0	−1
3HFM	L	122	1	−1
3HFM	L	123	0	−1
3HFM	L	124	0	−1
3HFM	L	125	0	−1
3HFM	L	126	1	−1
3HFM	L	127	1	−1
3HFM	L	128	1	−1
3HFM	L	129	0	−1
3HFM	L	130	0	−1
3HFM	L	131	0	−1
3HFM	L	132	0	−1
3HFM	L	133	0	−1
3HFM	L	134	0	−1
3HFM	L	135	0	−1
3HFM	L	136	0	−1
3HFM	L	137	0	−1
3HFM	L	138	1	−1
3HFM	L	139	0	−1

3HFM	L	140	0	−1
3HFM	L	141	0	−1
3HFM	L	142	1	−1
3HFM	L	143	1	−1
3HFM	L	144	0	−1
3HFM	L	145	1	−1
3HFM	L	146	0	−1
3HFM	L	147	1	−1
3HFM	L	148	0	−1
3HFM	L	149	0	−1
3HFM	L	150	0	−1
3HFM	L	151	1	−1
3HFM	L	152	1	−1
3HFM	L	153	1	−1
3HFM	L	154	1	−1
3HFM	L	155	1	−1
3HFM	L	156	1	−1
3HFM	L	157	1	−1
3HFM	L	158	1	−1
3HFM	L	159	1	−1
3HFM	L	160	0	−1
3HFM	L	161	1	−1
3HFM	L	162	0	−1
3HFM	L	163	1	−1
3HFM	L	164	0	−1
3HFM	L	165	1	−1
3HFM	L	166	0	−1
3HFM	L	167	1	−1
3HFM	L	168	1	−1
3HFM	L	169	1	−1
3HFM	L	170	1	−1
3HFM	L	171	0	−1
3HFM	L	172	0	−1
3HFM	L	173	0	−1
3HFM	L	174	0	−1
3HFM	L	175	0	−1
3HFM	L	176	0	−1
3HFM	L	177	0	−1
3HFM	L	178	0	−1
3HFM	L	179	0	−1
3HFM	L	180	1	−1
3HFM	L	181	0	−1
3HFM	L	182	1	−1
3HFM	L	183	1	−1
3HFM	L	184	1	−1
3HFM	L	185	1	−1
3HFM	L	186	0	−1
3HFM	L	187	1	−1
3HFM	L	188	1	−1
3HFM	L	189	1	−1
3HFM	L	190	1	−1
3HFM	L	191	1	−1

3HFM	L	192	0	−1
3HFM	L	193	0	−1
3HFM	L	194	0	−1
3HFM	L	195	0	−1
3HFM	L	196	0	−1
3HFM	L	197	1	−1
3HFM	L	198	0	−1
3HFM	L	199	1	−1
3HFM	L	200	1	−1
3HFM	L	201	1	−1
3HFM	L	202	1	−1
3HFM	L	203	1	−1
3HFM	L	204	1	−1
3HFM	L	205	1	−1
3HFM	L	206	1	−1
3HFM	L	207	0	−1
3HFM	L	208	1	−1
3HFM	L	209	0	−1
3HFM	L	210	1	−1
3HFM	L	211	1	−1
3HFM	L	212	1	−1
3HFM	L	213	1	−1
3HFM	L	214	1	−1
3HFM	H	1	1	−1
3HFM	H	2	0	−1
3HFM	H	3	1	−1
3HFM	H	4	0	−1
3HFM	H	5	1	−1
3HFM	H	6	0	−1
3HFM	H	7	1	−1
3HFM	H	8	1	−1
3HFM	H	9	1	−1
3HFM	H	10	1	−1
3HFM	H	11	1	−1
3HFM	H	12	0	−1
3HFM	H	13	1	−1
3HFM	H	14	1	−1
3HFM	H	15	1	−1
3HFM	H	16	1	−1
3HFM	H	17	1	−1
3HFM	H	18	0	−1
3HFM	H	19	1	−1
3HFM	H	20	0	−1
3HFM	H	21	0	−1
3HFM	H	22	0	−1
3HFM	H	23	1	−1
3HFM	H	24	0	−1
3HFM	H	25	1	−1
3HFM	H	26	1	−1
3HFM	H	27	1	1
3HFM	H	28	1	−1
3HFM	H	29	0	−1

3HFM	H	30	1	1
3HFM	H	31	1	1
3HFM	H	32	1	1
3HFM	H	33	1	1
3HFM	H	34	0	−1
3HFM	H	35	0	−1
3HFM	H	36	0	−1
3HFM	H	37	0	−1
3HFM	H	38	0	−1
3HFM	H	39	0	−1
3HFM	H	40	0	−1
3HFM	H	41	1	−1
3HFM	H	42	1	−1
3HFM	H	43	1	−1
3HFM	H	44	1	−1
3HFM	H	45	0	−1
3HFM	H	46	0	−1
3HFM	H	47	0	−1
3HFM	H	48	0	−1
3HFM	H	49	0	−1
3HFM	H	50	0	−1
3HFM	H	51	0	−1
3HFM	H	52	0	−1
3HFM	H	53	1	1
3HFM	H	54	1	1
3HFM	H	55	1	−1
3HFM	H	56	1	1
3HFM	H	57	1	−1
3HFM	H	58	1	1
3HFM	H	59	1	−1
3HFM	H	60	0	−1
3HFM	H	61	1	−1
3HFM	H	62	1	−1
3HFM	H	63	0	−1
3HFM	H	64	1	−1
3HFM	H	65	1	−1
3HFM	H	66	0	−1
3HFM	H	67	0	−1
3HFM	H	68	1	−1
3HFM	H	69	0	−1
3HFM	H	70	1	−1
3HFM	H	71	1	−1
3HFM	H	72	1	−1
3HFM	H	73	1	−1
3HFM	H	74	1	−1
3HFM	H	75	1	−1
3HFM	H	76	1	−1
3HFM	H	77	0	−1
3HFM	H	78	0	−1
3HFM	H	79	1	−1
3HFM	H	80	0	−1
3HFM	H	81	1	−1

3HFM	H	82	0	−1
3HFM	H	83	1	−1
3HFM	H	84	1	−1
3HFM	H	85	0	−1
3HFM	H	86	1	−1
3HFM	H	87	1	−1
3HFM	H	88	1	−1
3HFM	H	89	0	−1
3HFM	H	90	1	−1
3HFM	H	91	0	−1
3HFM	H	92	0	−1
3HFM	H	93	0	−1
3HFM	H	94	0	−1
3HFM	H	95	0	−1
3HFM	H	96	0	−1
3HFM	H	97	0	−1
3HFM	H	98	1	1
3HFM	H	99	0	−1
3HFM	H	100	0	−1
3HFM	H	101	1	1
3HFM	H	102	1	1
3HFM	H	103	0	−1
3HFM	H	104	0	−1
3HFM	H	105	1	−1
3HFM	H	106	0	−1
3HFM	H	107	0	−1
3HFM	H	108	1	−1
3HFM	H	109	0	−1
3HFM	H	110	0	−1
3HFM	H	111	0	−1
3HFM	H	112	0	−1
3HFM	H	113	1	−1
3HFM	H	114	1	−1
3HFM	H	115	1	−1
3HFM	H	116	1	−1
3HFM	H	117	1	−1
3HFM	H	118	1	−1
3HFM	H	119	0	−1
3HFM	H	120	1	−1
3HFM	H	121	0	−1
3HFM	H	122	0	−1
3HFM	H	123	0	−1
3HFM	H	124	0	−1
3HFM	H	125	0	−1
3HFM	H	126	0	−1
3HFM	H	127	1	−1
3HFM	H	128	1	−1
3HFM	H	129	1	−1
3HFM	H	130	1	−1
3HFM	H	131	1	−1
3HFM	H	132	1	−1
3HFM	H	133	1	−1

3HFM	H	134	1	−1
3HFM	H	135	1	−1
3HFM	H	136	0	−1
3HFM	H	137	0	−1
3HFM	H	138	0	−1
3HFM	H	139	0	−1
3HFM	H	140	0	−1
3HFM	H	141	0	−1
3HFM	H	142	0	−1
3HFM	H	143	1	−1
3HFM	H	144	0	−1
3HFM	H	145	0	−1
3HFM	H	146	0	−1
3HFM	H	147	0	−1
3HFM	H	148	0	−1
3HFM	H	149	1	−1
3HFM	H	150	0	−1
3HFM	H	151	1	−1
3HFM	H	152	0	−1
3HFM	H	153	1	−1
3HFM	H	154	0	−1
3HFM	H	155	0	−1
3HFM	H	156	1	−1
3HFM	H	157	1	−1
3HFM	H	158	1	−1
3HFM	H	159	1	−1
3HFM	H	160	1	−1
3HFM	H	161	1	−1
3HFM	H	162	1	−1
3HFM	H	163	1	−1
3HFM	H	164	0	−1
3HFM	H	165	1	−1
3HFM	H	166	0	−1
3HFM	H	167	1	−1
3HFM	H	168	0	−1
3HFM	H	169	1	−1
3HFM	H	170	1	−1
3HFM	H	171	1	−1
3HFM	H	172	1	−1
3HFM	H	173	1	−1
3HFM	H	174	1	−1
3HFM	H	175	0	−1
3HFM	H	176	0	−1
3HFM	H	177	0	−1
3HFM	H	178	0	−1
3HFM	H	179	0	−1
3HFM	H	180	0	−1
3HFM	H	181	0	−1
3HFM	H	182	0	−1
3HFM	H	183	0	−1
3HFM	H	184	1	−1
3HFM	H	185	1	−1

3HFM	H	186	1	−1
3HFM	H	187	1	−1
3HFM	H	188	0	−1
3HFM	H	189	1	−1
3HFM	H	190	1	−1
3HFM	H	191	1	−1
3HFM	H	192	1	−1
3HFM	H	193	0	−1
3HFM	H	194	0	−1
3HFM	H	195	0	−1
3HFM	H	196	0	−1
3HFM	H	197	0	−1
3HFM	H	198	0	−1
3HFM	H	199	0	−1
3HFM	H	200	1	−1
3HFM	H	201	1	−1
3HFM	H	202	0	−1
3HFM	H	203	1	−1
3HFM	H	204	1	−1
3HFM	H	205	1	−1
3HFM	H	206	1	−1
3HFM	H	207	1	−1
3HFM	H	208	1	−1
3HFM	H	209	1	−1
3HFM	H	210	0	−1
3HFM	H	211	1	−1
3HFM	H	212	0	−1
3HFM	H	213	1	−1
3HFM	H	214	1	−1
3HFM	H	215	1	−1
3SGB	E	1	0	−1
3SGB	E	2	0	−1
3SGB	E	3	0	−1
3SGB	E	4	0	−1
3SGB	E	5	1	−1
3SGB	E	6	0	−1
3SGB	E	7	0	−1
3SGB	E	8	0	−1
3SGB	E	9	0	−1
3SGB	E	10	1	−1
3SGB	E	11	1	1
3SGB	E	12	1	1
3SGB	E	13	1	1
3SGB	E	14	0	−1
3SGB	E	15	0	−1
3SGB	E	16	0	−1
3SGB	E	17	0	−1
3SGB	E	18	0	−1
3SGB	E	19	0	−1
3SGB	E	20	0	−1
3SGB	E	21	1	−1
3SGB	E	22	1	−1

3SGB	E	23	1	−1
3SGB	E	24	1	−1
3SGB	E	25	1	−1
3SGB	E	26	1	−1
3SGB	E	27	0	−1
3SGB	E	28	0	−1
3SGB	E	29	0	−1
3SGB	E	30	0	−1
3SGB	E	31	0	−1
3SGB	E	32	0	−1
3SGB	E	33	1	1
3SGB	E	34	0	−1
3SGB	E	35	0	−1
3SGB	E	36	1	−1
3SGB	E	37	1	−1
3SGB	E	38	0	−1
3SGB	E	39	1	−1
3SGB	E	40	1	−1
3SGB	E	41	0	−1
3SGB	E	42	0	−1
3SGB	E	43	1	−1
3SGB	E	44	1	−1
3SGB	E	45	1	−1
3SGB	E	46	1	−1
3SGB	E	47	1	−1
3SGB	E	48	1	−1
3SGB	E	49	1	−1
3SGB	E	50	1	−1
3SGB	E	51	0	−1
3SGB	E	52	0	−1
3SGB	E	53	1	−1
3SGB	E	54	1	−1
3SGB	E	55	1	−1
3SGB	E	56	0	−1
3SGB	E	57	1	−1
3SGB	E	58	0	−1
3SGB	E	59	0	−1
3SGB	E	60	0	−1
3SGB	E	61	1	−1
3SGB	E	62	1	−1
3SGB	E	63	0	−1
3SGB	E	64	0	−1
3SGB	E	65	0	−1
3SGB	E	66	0	−1
3SGB	E	67	0	−1
3SGB	E	68	1	−1
3SGB	E	69	0	−1
3SGB	E	70	1	−1
3SGB	E	71	0	−1
3SGB	E	72	1	−1
3SGB	E	73	1	−1
3SGB	E	74	0	−1

3SGB	E	75	1	−1
3SGB	E	76	0	−1
3SGB	E	77	1	−1
3SGB	E	78	0	−1
3SGB	E	79	0	−1
3SGB	E	80	0	−1
3SGB	E	81	0	−1
3SGB	E	82	1	−1
3SGB	E	83	1	−1
3SGB	E	84	1	−1
3SGB	E	85	0	−1
3SGB	E	86	1	−1
3SGB	E	87	1	−1
3SGB	E	88	1	−1
3SGB	E	89	0	−1
3SGB	E	90	1	−1
3SGB	E	91	0	−1
3SGB	E	92	1	−1
3SGB	E	93	1	−1
3SGB	E	94	1	−1
3SGB	E	95	0	−1
3SGB	E	96	1	−1
3SGB	E	97	0	−1
3SGB	E	98	0	−1
3SGB	E	99	0	−1
3SGB	E	100	0	−1
3SGB	E	101	0	−1
3SGB	E	102	1	−1
3SGB	E	103	1	1
3SGB	E	104	0	−1
3SGB	E	105	1	−1
3SGB	E	106	1	−1
3SGB	E	107	1	−1
3SGB	E	108	1	−1
3SGB	E	109	1	−1
3SGB	E	110	1	−1
3SGB	E	111	0	−1
3SGB	E	112	1	−1
3SGB	E	113	1	−1
3SGB	E	114	1	−1
3SGB	E	115	1	−1
3SGB	E	116	0	−1
3SGB	E	117	1	−1
3SGB	E	118	0	−1
3SGB	E	119	1	1
3SGB	E	120	1	1
3SGB	E	121	1	1
3SGB	E	122	1	1
3SGB	E	123	1	−1
3SGB	E	124	1	−1
3SGB	E	125	0	−1
3SGB	E	126	0	−1

3SGB	E	127	1	−1
3SGB	E	128	0	−1
3SGB	E	129	0	−1
3SGB	E	130	0	−1
3SGB	E	131	1	−1
3SGB	E	132	0	−1
3SGB	E	133	1	−1
3SGB	E	134	0	−1
3SGB	E	135	0	−1
3SGB	E	136	0	−1
3SGB	E	137	1	1
3SGB	E	138	1	1
3SGB	E	139	0	−1
3SGB	E	140	0	−1
3SGB	E	141	0	−1
3SGB	E	142	0	−1
3SGB	E	143	0	−1
3SGB	E	144	0	−1
3SGB	E	145	0	−1
3SGB	E	146	0	−1
3SGB	E	147	0	−1
3SGB	E	148	1	−1
3SGB	E	149	1	−1
3SGB	E	150	1	−1
3SGB	E	151	0	−1
3SGB	E	152	0	−1
3SGB	E	153	0	−1
3SGB	E	154	0	−1
3SGB	E	155	0	−1
3SGB	E	156	0	−1
3SGB	E	157	0	−1
3SGB	E	158	1	1
3SGB	E	159	1	1
3SGB	E	160	1	1
3SGB	E	161	1	1
3SGB	E	162	1	−1
3SGB	E	163	1	−1
3SGB	E	164	1	−1
3SGB	E	165	1	−1
3SGB	E	166	0	−1
3SGB	E	167	0	−1
3SGB	E	168	0	−1
3SGB	E	169	0	−1
3SGB	E	170	0	−1
3SGB	E	171	0	−1
3SGB	E	172	0	−1
3SGB	E	173	0	−1
3SGB	E	174	0	−1
3SGB	E	175	1	−1
3SGB	E	176	0	−1
3SGB	E	177	0	−1
3SGB	E	178	1	−1

3SGB	E	179	1	−1
3SGB	E	180	1	−1
3SGB	E	181	1	−1
3SGB	E	182	0	−1
3SGB	E	183	1	−1
3SGB	E	184	0	−1
3SGB	E	185	1	−1
4CPA	I	3	1	−1
4CPA	I	4	1	−1
4CPA	I	5	0	−1
4CPA	I	6	1	−1
4CPA	I	7	0	−1
4CPA	I	8	0	−1
4CPA	I	9	1	−1
4CPA	I	10	1	−1
4CPA	I	11	1	−1
4CPA	I	12	0	−1
4CPA	I	13	1	−1
4CPA	I	14	1	−1
4CPA	I	15	1	1
4CPA	I	16	1	−1
4CPA	I	17	1	−1
4CPA	I	18	0	−1
4CPA	I	19	1	−1
4CPA	I	20	1	−1
4CPA	I	21	0	−1
4CPA	I	22	1	1
4CPA	I	23	1	1
4CPA	I	24	0	−1
4CPA	I	25	1	1
4CPA	I	26	0	−1
4CPA	I	27	0	−1
4CPA	I	28	1	1
4CPA	I	29	1	1
4CPA	I	30	1	1
4CPA	I	31	1	−1
4CPA	I	32	1	−1
4CPA	I	33	1	−1
4CPA	I	34	0	−1
4CPA	I	35	0	−1
4CPA	I	36	0	−1
4CPA	I	37	1	1
4CPA	I	38	1	1
4HTC	I	1	1	1
4HTC	I	2	1	1
4HTC	I	3	1	1
4HTC	I	4	1	1
4HTC	I	5	1	1
4HTC	I	6	0	−1
4HTC	I	7	1	−1
4HTC	I	8	1	−1
4HTC	I	9	1	−1

4HTC	I	10	1	−1
4HTC	I	11	0	−1
4HTC	I	12	0	−1
4HTC	I	13	1	1
4HTC	I	14	0	−1
4HTC	I	15	0	−1
4HTC	I	16	0	−1
4HTC	I	17	1	1
4HTC	I	18	1	1
4HTC	I	19	1	1
4HTC	I	20	1	1
4HTC	I	21	1	1
4HTC	I	22	0	−1
4HTC	I	23	1	−1
4HTC	I	24	1	1
4HTC	I	25	1	−1
4HTC	I	26	1	−1
4HTC	I	27	1	−1
4HTC	I	28	0	−1
4HTC	I	29	0	−1
4HTC	I	30	1	−1
4HTC	I	31	1	−1
4HTC	I	36	1	−1
4HTC	I	37	1	−1
4HTC	I	38	1	−1
4HTC	I	39	1	1
4HTC	I	40	0	−1
4HTC	I	41	1	−1
4HTC	I	42	1	−1
4HTC	I	43	1	−1
4HTC	I	44	0	−1
4HTC	I	45	1	−1
4HTC	I	46	1	1
4HTC	I	47	1	1
4HTC	I	48	1	1
4HTC	I	49	1	1
4HTC	I	50	1	1
4HTC	I	51	1	1
4HTC	I	52	1	1
4HTC	I	53	1	1
4HTC	I	54	1	1
4HTC	I	55	1	1
4HTC	I	56	1	1
4HTC	I	57	1	1
4HTC	I	58	1	1
4HTC	I	59	1	1
4HTC	I	60	1	1
4HTC	I	61	1	−1
4HTC	I	62	1	1
4HTC	I	63	1	1
4HTC	I	64	1	1
4HTC	I	65	1	1
